# The Mechanism of Ginseng and Astragalus Decoction in the Treatment of Malignant Pleural Effusion Based on Network Pharmacology and Molecular Docking Technology

**DOI:** 10.1155/2022/7731402

**Published:** 2022-03-15

**Authors:** Fengying Gong, Rongmei Qu, Yuchao Yang, Yongchun Li, Yunshui Cheng, Qiang Zhang, Ronglv Huang, Qin Fan, Jingxing Dai, Ying Lv

**Affiliations:** ^1^Nanfang Hospital of Southern Medical University, Guangzhou 510515, China; ^2^Guangdong Provincial Key Laboratory of Medical Biomechanics & Guangdong Engineering Research Center for Translation of Medical 3D Printing Application & National Key Discipline of Human Anatomy, School of Basic Medical Science, Southern Medical University, Guangzhou, China; ^3^School of Traditional Chinese Medicine, Southern Medical University, Guangzhou 510515, China

## Abstract

**Introduction:**

The objective of our study is to explore the potential active ingredients and activity of Ginseng and Astragalus decoction (GAD) in the treatment of malignant pleural effusion (MPE) by using network pharmacology and molecular docking technologies.

**Methods:**

The active ingredients and corresponding targets of Ginseng and Astragalus were extracted from the Traditional Chinese Medicine System Pharmacology Database and Analysis Platform. The relevant targets of malignant pleural effusion (MPE) were searched in the disease databases. Overlapping targets of Ginseng and Astragalus and the corresponding targets of MPE were obtained to define the effective target of GAD for the treatment of MPE. The STRING database was applied to construct a predicted protein-protein interaction network for intersected targets. The Cytoscape software was used to screen key targets with a therapeutic potential. Using the Metascape database, we performed Gene Ontology and Kyoto Encyclopedia of Genes and Genomes functional enrichment analysis on the targets identified in the study. PyMOL and AutoDock Vina were used to molecularly dock the selected key components to their respective key targets for MPE treatment.

**Results:**

The core target network revealed 22 main active ingredients, 26 main targets, and 16 signaling pathways in GAD. Molecular docking revealed 6 targets (AKT serine/threonine kinase 1, intercellular adhesion molecule, Jun proto-oncogene, peroxisome proliferator activated receptor gamma, prostaglandin-endoperoxide synthase 2, and tumor necrosis factor) that could partially dock with kaempferol, frutinone A, ginsenoside RH2, formononetin, and quercetin.

**Conclusions:**

Several components, targets, and signaling pathways of GAD contribute to the treatment of MPE, which suggests a rationale for further investigation on GAD's active molecule and mechanism of action in the clinical application of MPE.

## 1. Introduction

Malignant pleural effusion (MPE) is one of the most common adverse consequences of lung cancer in the advanced stages of the disease [1]. It was reported that MPE is primarily associated with malignant tumors, and lung carcinoma is the most common type [2]. Interestingly, lung carcinoma is the most prevalent cancer concern in most studies representing one-third of all malignant effusions, followed by breast carcinoma. Moreover, there are other significant reasons for MPE, namely, Hodgkin's and non-Hodgkin's lymphoma. Several types of tumors are associated with less MPE including ovarian and gastrointestinal cancers [3]. Most patients present with signs of advanced cachexia, such as weight loss, emaciation, and anemia and symptoms including progressive dyspnea, chest pain, and dry cough. Several factors contribute to the dyspnea level, including the amount of pleural effusion, its formation rate, and the functional status of the patient's lung [4]. At present, modern medicine mainly treats MPE with the intrathoracic infusion of chemotherapy drugs, pleurodesis, and pleurocentesis, but the overall efficacy is limited, and treatments are not suitable for advanced and weak elderly patients with poor physical strength [5].

In clinical practice, traditional Chinese medicine (TCM) has been considered to be effective in the clinical treatment of MPE [6, 7], but the rationale for its use is mainly based on clinical observation and the administration of multiple medicines, and there is a lack of research about single drugs and prescriptions. The Ginseng and Astragalus decoction (GAD) are composed of Astragalus and Ginseng to strengthen qi and help yang [8]. Astragalus and Ginseng are the top medicines described in the Shennong Materia Medica; they taste sweet and have the effect of tonifying qi [9]. In the “Elbow Reserve Urgent Prescription” written by Gehong in the Eastern Jin Dynasty, records of Ginseng and Astragalus were shared and then they subsequently appeared in the classic prescriptions for treating abnormal water metabolism, such as Baoyuan Decoction, Buzhong Yiqi Decoction, and Guipi Decoction. Modern pharmacological studies have shown that both Astragalus and Ginseng exert antitumor and immunological effects.

Network pharmacology is a new network model of multitarget, multipathway, and multiple associations that combines biological and pharmacological knowledge with a modern data analysis technology to reveal the mechanisms of GAD through multimolecule synergistic effects on diseases. This study aimed to explore potential active ingredients of the GAD, its targets, and mechanisms of action on MPE to understand the clinical application of MPE with GAD.

## 2. Materials and Methods

### 2.1. Related Databases

The Systematic Pharmacology database of Traditional Chinese medicine (TCMSP, https://tcmspw.com/tcmsp.php) [10]; Protein database, UniProt (https://www.uniprot.org/) [11]; Human Gene Database, GeneCards (https://www.genecards.org/); Online Mendelian Inheritance in Man (OMIM, https://omim.org/) [12]; and Drug database, DrugBank (https://go.drugbank.com/) [13] were used for this analysis. The protein-protein interaction analysis platform, STRING (https://www.string-db.org); Network analysis and mapping software, Cytoscape 3.8.2 (National Institute of General Medical Sciences, Bethesda, MD, USA); the Gene function analysis database, Metascape (https://metascape.org) [13]; Protein structure database, PDB (http://www1.rcsb.org/) [14]; Compound structure database, PubChem (https://pubchem.ncbi.nlm.nih.gov/); 3D structure display software, PyMOL (Schrödinger, Portland, OR, USA) [15]; molecular docking software, AutoDock Vina 1.1.2 (http://vina.scripps.edu/)[16] and bioinformatics mapping platform (http://www.bioinformatics.com.cn/) were used as bioinformatics tools.

### 2.2. Screening and Target Determination of Main Active Components of Ginseng and Astragalus

The main active components of Ginseng and Astragalus were retrieved from the systematic pharmacology analysis platform TCMSP. Oral bioavailability (OB) ≥ 30% and drug-like (DL) ≥ 0.18 were selected as the screening conditions for active likeness. The targets of active ingredient were retrieved from the TCMSP database, and the information of target proteins were standardized and converted through UniProt (Supplemental Table 1).

### 2.3. Prediction of Disease Targets of MPE

MPE was used as the search term to retrieve MPE disease targets in GeneCards, OMIM, and DrugBank. Duplicate genes were deleted.

### 2.4. Analysis of Interaction between Effective Targets and Common Targets of MPE in the GAD

Venny analysis was performed to seek out the intersecting targets between the effective target of GAD and the disease target of MPE. A protein-protein interaction (PPI) network was raised for the intersecting targets using STRING database.

### 2.5. Core Network Screening and Target Function Enrichment Analysis

Cytoscape was applied to analyze the PPI network, to analyze closeness, betweenness, and degree of nodes in the network and to screen according to the median of the three parameters. Metascape was applied to conduct Gene Ontology (GO) and Kyoto Encyclopedia of Genes and Genomes (KEGG) functional enrichment analysis for the screened core network proteins, and the biological processes and pathways with *P*  <  0.05, were screened out. The sequence of the enriched genes was carried out, and the pathway information was imported into the bioinformatics tool to draw the bubble map and bar chart.

### 2.6. Molecular Docking Verification

Core network proteins were carefully chosen, and the corresponding active components were determined according to the relationship between the active components and the corresponding targets. The three-dimensional structure (3D) was downloaded and established using the “.PDB” file of the core protein in the PDB database, and the corresponding 3D “.SDF” file of the structure of the active ingredient in the PubChem database. With the PyMOL software, the 3D structure files were processed by water removal, hydrogenation, and charge distribution. Molecular docking was executed out using the software AutoDock Vina 1.1.2 [16]. Binding energy (affinity) was applied to estimate the binding affinity between the active components and the target proteins, and visual processing was carried out using the software PyMOL (supplemental Table 2) [15].

## 3. Results

### 3.1. Screening of Main Active Components of the GAD

**Figure 1:**
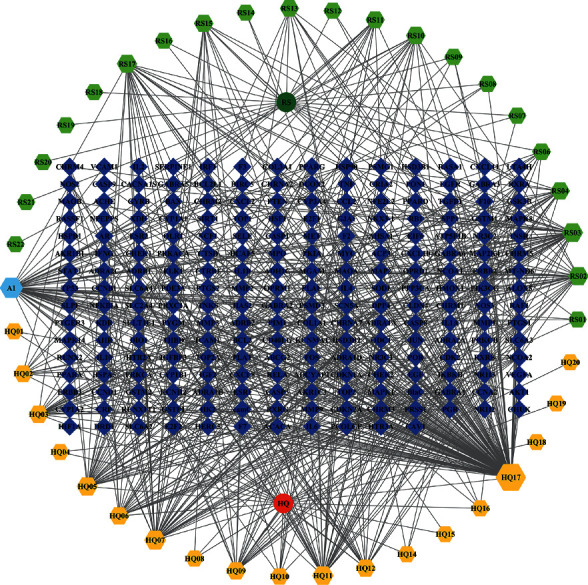
Interaction network map between active ingredients and targets of the GAD.

In total, 190 compounds were identified in Ginseng and 87 compounds were identified in Astragalus from the TCMSP platform. Based on the selection criteria (OB ≥ 30% and DL ≥ 0.18), the core active ingredients were further filtered to 22 in Ginseng and 20 in Astragalus, among which kaempferol (KPL) was a compound shared by both drugs (Table 1). Next, 222 active component-related targets were located in the TCMSP database. Cytoscape 3.8.1 was used to construct the TCM-component-target network map (Figure 1).

### 3.2. Screening of MPE Disease Targets

**Figure 2:**
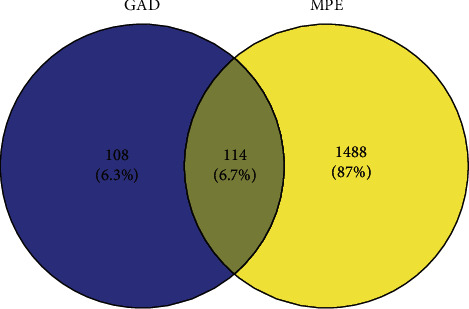
Venn map of GAD and MPE targets.

**Figure 3:**
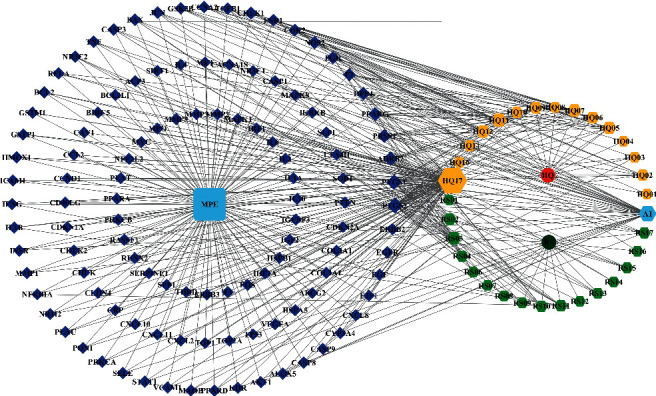
Network map of GAD-component-target disease.

By searching GeneCards, OMIM, and DrugBank databases, 1499, 315, and 124 target genes related to MPE were collected, respectively. We derived a list of 1602 genes that may have been responsible for the MPE, considering only the genes that were not repeated. It was determined through Venn analysis that 114 intersection genes (potential targets) might be regulated by the active ingredient of GAD treatment on MPE (Figure 2). A network diagram consisting of GAD components, target diseases, and targets was constructed in the Cytoscape 3.8.2 software (Figure 3).

### 3.3. Potential Targets of the GAD to Treat MPE Based on PPI Analysis

**Figure 4:**
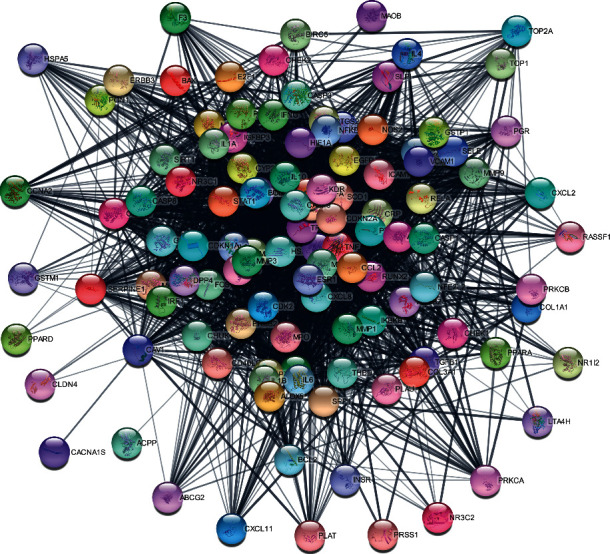
PPI network map of the intersection targets.

**Figure 5:**
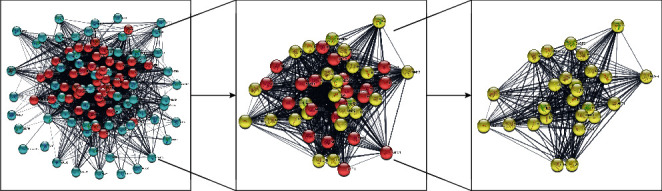
PPI network construction and core target screening process diagram.

To analyze interaction between proteins, 114 potential targets were added to the STRING database (Figure 4). There were 114 nodes and 2324 variations in PPI interactions, and the average node level was 40.77. CytoNCA was used to calculate the tightness, betweenness, and degree of each node. The PPI network was further screened to define elements ≥ the median value of the three parameters. Twenty-six core targets were finally screened (Figure 5).

### 3.4. Interaction Target Pathway and Functional Analysis

**Figure 6:**
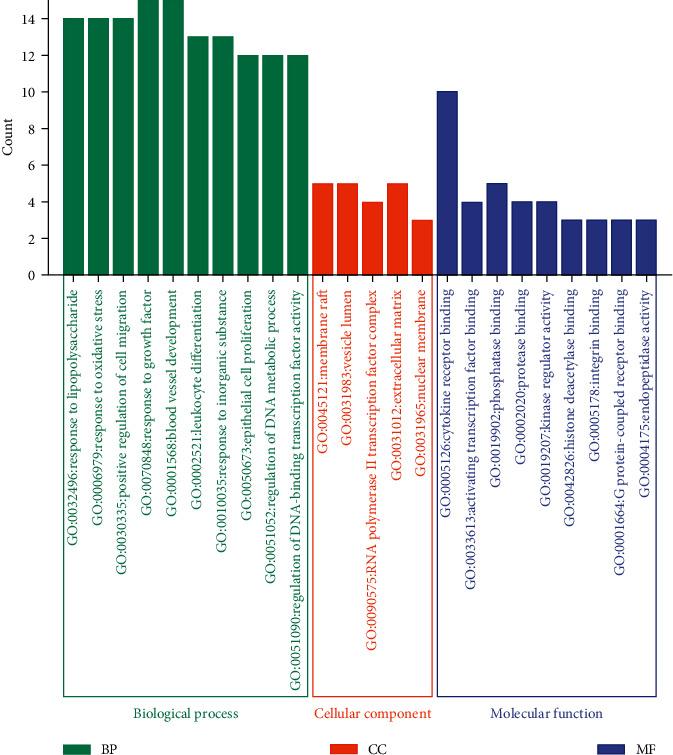
GO enrichment analysis.

GO functional analysis and signaling pathway analysis were performed with the 26 targets in Metascape and KEGG, respectively. GO function enrichment analysis identified the following processes: biological processes mainly concentrated in the lipopolysaccharide response, oxidative stress response, regulation of cell migration, response to growth factors, angiogenesis, leukocyte differentiation, response to inorganic substances, epithelial cell proliferation, DNA metabolism, and DNA-binding transcription factor activity regulation (Figure 6). Cellular components were principally concentrated in membrane rafts, intracellular cavities, the RNA polymerase II transcription factor complex, the extracellular matrix, and the nuclear membrane. Molecular functions were mainly concentrated in cytokine receptor binding, activated transcription factor binding, phosphatase binding, protease binding, kinase regulatory activity, histone deacetylase binding, integrin binding, *G* protein coupled receptor binding, and endopeptidase activities.

**Figure 7:**
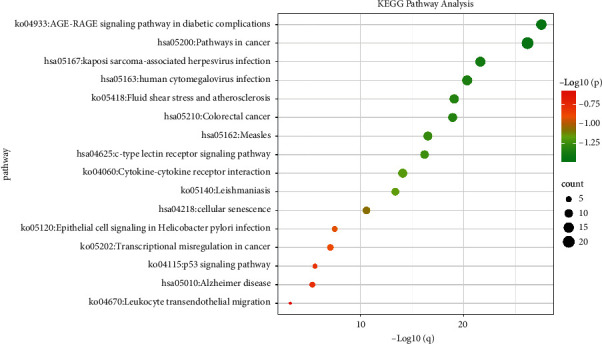
KEGG signal pathway analysis.

The enrichment analysis of the KEGG signaling pathway is shown in Figure 7. The results demonstrate that the 26 potential targets are mainly involved in the following signaling pathways: age/rage signaling, cancer, human cytomegalovirus infection, interaction between cytokines and cytokine receptors, transcriptional imbalance in cancer, and p53 signaling. These results indicated that the GAD may act on multiple biological processes, molecular functions, and signaling pathways to exert therapeutic effects on MPE.

### 3.5. Molecular Docking

**Figure 8:**
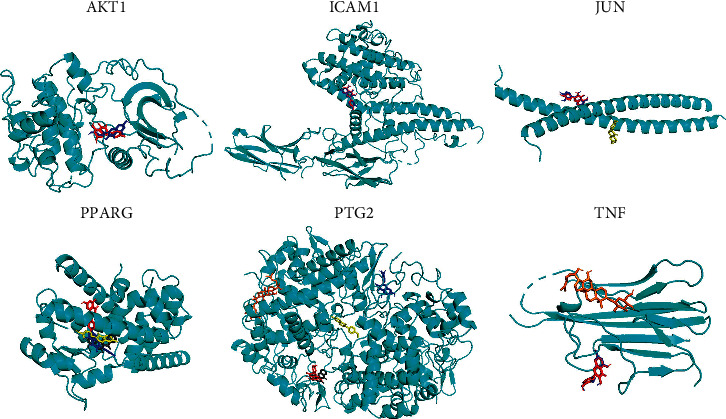
Molecular docking pattern.

Molecular docking was used to predict the binding pattern and affinity of drugs and proteins through the characteristics of the receptor, as defined by the interaction between the receptor and the drug molecule. The conformational stability of the ligand binding to the receptor is defined as by its binding energy (kcal/mol): lower binding energy means the higher stabilization of the ligand. We performed a molecular docking simulation between the top 6 targets AKT serine/threonine kinase 1 (AKT1), intercellular adhesion molecule 1 (ICAM1), Jun proto-oncogene (JUN), peroxisome proliferator-activated receptor gamma (PPARG), prostaglandin-endoperoxide synthase 2 (PTGS2), and tumor necrosis factor (TNF) in the core target network with kaempferol (KPL), frutinone A (FNA), ginsenoside RH2 (GRH2), formononetin (FNN), and quercetin (QCN), respectively (Figure 8 and Table 2). The results showed that many active components had a strong binding ability with the target, and the conformation of the combination was stable. Comprehensive analysis showed that KPL, FNA, GRH2, FNN, and QCN might be the key components of the GAD in the treatment of MPE.

## 4. Discussion

Most MPE patients present with systemic symptoms such as inappetence, weight loss, and discomfort because the condition is usually diagnosed at an advanced stage. There is no clear understanding of how large pleural effusions cause breathing difficulties. However, various factors could include reduced chest wall compliance, contralateral mediastinal displacement, ipsilateral lung volume reduction, and pain from lung and chest wall stimulation [17]. The presence of MPE in patients is an indication that the cancer has spread throughout the body and predicts a decline in the patient's life expectancy and quality of life [18].

The relationships between drug-compound-target-disease resulting from the treatment of GAD were explored using a network pharmacology approach and 41 compounds and 114 targets were identified from the study. Among these, KPL, FNA, GRH2, FNN, and QCN were identified as the main potential active ingredients. The most common flavonoid in vegetables and fruit is KPL, a natural ingredient commonly found in many foods. It has been described to have a variety of anticancer activities, against tumors of the breast, prostate, bladder, cervical, colon, liver, lung, ovarian, and leukemia [19]. Studies have reported eating a diet rich in KPL to reduce the threats of certain kinds of cancer, including cutaneum carcinoma, hepatoma, and colorectal carcinoma. Mechanisms of its effect include apoptosis, cell cycle stagnated in the G2/M phase, downregulation of epithelial-mesenchymal transition-related markers, and phosphoinositol 3-kinase/protein kinase B signaling [20]. FNA is obtained from the extraction of wild fruits with lipophilic grade. Recent studies have found that FNA has a variety of antibacterial and antifungal properties [21], but there have been no antitumor studies. A wide range of pharmacological properties are attributed to its main component, ginsenosides, including strengthening the immune system, building stress resistance, enhancing cardiovascular function, improving mental health, and reducing chemotherapy complications. GRH2, one of the key bioactive ginsenosides of Ginseng, exerts its antitumor action by preventing tumor migration, proliferation, invasion, and metastasis. Aside from this, it also controls the cell cycle, encourages differentiation, reverses the effects of multidrug resistance and is known to reduce the effects of chemotherapy or radiation therapy on tumorigenesis [22].

FNN is an isoflavone derived from numerous therapeutic herbs and plants, such as Astragalus and red clover. FNN has been studied extensively in the past decade because of strong evidence that it promotes apoptosis and inhibits proliferation, suggesting that it can be used as an anticancer drug against various cancer types. Breast, prostatic carcinoma, and colorectal tumors are among the tumor models in which the anticancer properties have been observed. The ability of FNN to inhibit tumor growth and metastasis has also been demonstrated in various in vivo studies [23, 24]. The helpful results from FNN can be ascribed to its properties of inhibiting tumor cell proliferation and arresting or delaying cell cycle. FNN adjusts oncogenic pathways mediated by various transcription and growth factors, thus reducing the probable reasons of chronic inflammation associated with cancer survival of inflammation cells and tolerance to the chemical therapy [25]. Quercetin is a subgroup of the flavonoid family that functions as a representative, and it is known to be one of the most familiar eating flavonols in western culture. As part of its antineoplastic properties, QCN can reduce cellular activity, induce apoptosis, and activate autophagy through adjustments in the PI3K/Akt, Wnt/*β*-catenin, and MAPK/ERK signaling pathways [26].

According to the core target network, GAD activity is exerted mainly via activation of AKT1, ICAM1, JUN, PPARG, PTGS2, and TNF and other targets in MPE. Among these, AKT1 is an important signaling molecule that plays a necessary effect in cell cycle, growth, survival, metabolism, and immune response. AKT1 is highly activated in a variety of human cancers. AKT1 is phosphorylated at Thr308 and Ser473, two important modified sites. Active AKT1 could activate numerous downstream proteins in cytoplasm and/or nucleus [27]. In the advanced cancer, AKT1 plays a specific effect in mediating tumor cell-vascular interoperability and regulates cancer metastasis through a mechanism that differs from its function of promoting tumorigenesis [28]. In addition, point mutations of AKT1 and internal tandem repeats of AKT1 have been observed in a study of sclerosing pneumocytoma, suggesting that AKT1 mutations are a genetic marker of sclerosing pneumocytoma [29]. Studies have shown that knockdown of AKT1 stimulates the nuclear translocation of *β*-catenin, leading to breast cancer cell infiltration. AKT1 inhibition induces nuclear accumulation of *β*-catenin and depends on extended stimulation of the EGFR signaling pathway in mammary gland tumor cells. Downregulation of AKT1 in mechanistic experiments dephosphorylate PIKfyve on Ser and inactivate the PIKfyve site, resulting in mammary cancer metastasis through EGFR signaling pathways [30].

As a member of the immunoglobulin superfamily of adhesion molecules, ICAM1 is an important adhesion molecule that mediates adhesion reactions. ICAM1 significantly encourages the adhesion of inflammatory sites, regulates cancer development and metastasis, and modifies the immune response. ICAM1, ICAM4, and ICAM5 are associated with breast cancer susceptibility loci as indicators of disease severity [31]. The expression of ICAM1 and its soluble part is more obvious during the inflammatory response, chronic diseases, and many malignant tumors. Moreover, the role of this hormone in the progression and prognosis of lung tumors cannot be underestimated [32]. Furthermore, ICAM1 was associated with poor survival in gastric cancer by Chen et al. [33].

C-Jun is an element of transcription factor activator protein 1, which associates to and stimulates transcription on the TRE/AP-1 element. C-Jun-dependent transcription is activated when phosphorylation of C-Jun at serene 63/73 is stimulated by various growth factor, cellular signaling, oncoproteins, and ultraviolet radiations. So, phosphorylated C-Jun may significantly affect tumorigenesis and cancer development [34]. C-Jun is a proto-oncogene that encourages cell proliferation during ectopic accumulation but can be ubiquitinated by SCFs (SKP1-cullin-F-box) (FBXW7, resulting in its degradation. FBXW7 (F-Box and WD repeat domain containing 7, E3 ubiquitin-protein ligase of C-Jun) has been reported to be carcinogenic in colon cancer. Lin et al. [35] reported that overexpression of lysine demethylase 5C reduced FBXW7 transcription and c-Jun protein accumulation, resulting in increased proliferation of human colon tumor cells.

Few publications have demonstrated that peroxisome proliferator-activated receptors impact the progression of a variety of tumors. Zhao et al. [36] reported that the enlarged expression of PPARG can trigger the activation of a variety of molecular factors, to combat the pathological development and prognosis of lung adenocarcinoma, indicating that PPARG is an important therapeutic target treatment. Studies have shown that PPARG may increase the chemical sensitivity of pharyngeal squamous cell carcinoma [37, 38]. Studies have also shown that prognosis in the Chinese Han population is related to PPARG gene polymorphism [39].

PTGS2 is the important enzyme in prostaglandin biosynthesis, which differs in regulating expression and tissue distribution. It is controlled by precise stimuli, suggesting that it is involved in prostaglandin biosynthesis-related with inflammation and mitosis. It has been suggested that tumor recurrence and prognosis of colon tumor is related to PTGS2 expression [40]. Furthermore, mutations in microRNA binding sites in the 3'UTR of the PTGS2 gene are significantly associated with cancer risk [41]. Chen et al. [42] found that PTGS2 was linked to mitochondrial mechanisms that regulate the growth of cancer cells. Cruceriu et al. [43] confirmed the molecular role of TNF-*α* signaling in mammary gland cancer cells, including clinical observations related to TNF-*α*. Møller et al. [44] found that mir-21 was involved in cancer amplification in preinvasion colon cancer by inhibiting TNF-*α* expression. Further, TNF-*α* paracrine and autocrine activity induced cell death in colorectal cancer. Thus, as can be seen from the above, the core targets of GAD defined in this study mainly involve biological processes, for example, cell migration, proliferation, cell survival, inflammatory response, and cell death. Moreover, it shows that the GAD plays an important part in the treatment of MPE through multiple targets.

## 5. Conclusions

In our study, network pharmacology and molecular docking techniques were applied to preliminary explore the key compounds, action targets, biological functions, signaling pathways, and potential mechanisms of the GAD in the treatment of MPE. The results showed that KPL, FNA, and GRH2, FNN, QCN, and other major active ingredients might play an important part in inhibiting apoptosis and regulating cell metabolism through multiple signaling pathways. The treatment effect on MPE by the GAD is not mediated by a single component but involves a collaborative effect of numerous components and targets. The results indicate the potential effect of the GAD on MPE and provide a direction and rationale for further studies involving the GAD on MPE.

## Figures and Tables

**Table 1:** Basic information of active ingredients in Ginseng and Astragalus decoction.

Number	Compound name	OB%	DL	Component source
A1	Kaempferol	41.88	0.24	Ginseng, Astragalus
RS01	Diop	43.59	0.39	Ginseng
RS02	Stigmasterol	43.83	0.76	Ginseng
RS03	Beta-sitosterol	36.91	0.75	Ginseng
RS04	Inermin	65.83	0.54	Ginseng
RS06	Aposiopolamine	66.65	0.22	Ginseng
RS07	Deoxyharringtonine	39.27	0.81	Ginseng
RS08	Dianthramine	40.45	0.2	Ginseng
RS09	Arachidonate	45.57	0.2	Ginseng
RS10	Frutinone A	65.9	0.34	Ginseng
RS11	Ginsenoside RH2	36.32	0.56	Ginseng
RS12	Ginsenoside-Rh4_qt	31.11	0.78	Ginseng
RS13	Girinimbine	61.22	0.31	Ginseng
RS14	Panaxadiol	33.09	0.79	Ginseng
RS15	Suchilactone	57.52	0.56	Ginseng
RS16	Alexandrine_qt	36.91	0.75	Ginseng
RS17	Fumarine	59.26	0.83	Ginseng
RS18	Chrysanthemaxanthin	38.72	0.58	Ginseng
RS19	Celabenzine	101.88	0.49	Ginseng
RS20	Gomisin B	31.99	0.83	Ginseng
RS21	Malkangunin	57.71	0.63	Ginseng
RS22	Ginsenoside Rg5_qt	39.56	0.79	Ginseng
HQ01	Mairin	55.38	0.78	Astragalus
HQ02	Jaranol	50.83	0.29	Astragalus
HQ03	Hederagenin	36.91	0.75	Astragalus
HQ04	(3S,8S,9S,10R,13R,14S,17R)-10,13-Dimethyl-17-[(2R,5S)-5-propan-2-yloctan-2-yl]-2,3,4,7,8,9,11,12,14,15,16,17-dodecahydro-1H-cyclopenta[a] phenanthren-3-ol	36.23	0.78	Astragalus
HQ05	Isorhamnetin	49.6	0.31	Astragalus
HQ06	3,9-di-O-Methylnissolin	53.74	0.48	Astragalus
HQ07	7-O-Methylisomucronulatol	74.69	0.3	Astragalus
HQ08	9,10-Dimethoxypterocarpan-3-O-*β*-D-glucoside	36.74	0.92	Astragalus
HQ09	(6aR,11aR)-9,10-Dimethoxy-6a,11a-dihydro-6H-benzofurano[3,2-c] chromen-3-ol	64.26	0.42	Astragalus
HQ10	Bifendate	31.1	0.67	Astragalus
HQ11	Formononetin	69.67	0.21	Astragalus
HQ12	Calycosin	47.75	0.24	Astragalus
HQ14	FA	68.96	0.71	Astragalus
HQ15	Isomucronulatol-7,2′-di-O-glucosiole	49.28	0.62	Astragalus
HQ16	1,7-Dihydroxy-3,9-dimethoxy pterocarpene	39.05	0.48	Astragalus
HQ17	Quercetin	46.43	0.28	Astragalus
HQ18	Isoflavanone	109.99	0.3	Astragalus
HQ19	5′-Hydroxyiso-muronulatol-2′,5′-di-O-glucoside	41.72	0.69	Astragalus
HQ20	(3R)-3-(2-Hydroxy-3,4-dimethoxyphenyl) chroman-7-ol	67.67	0.26	Astragalus

**Table 2:** Results of molecular docking between active components of the GAD and potential targets of MPE.

Target	Binding energy (kcal/mol)				
	Kaempferol (A1)	Frutinone A (RS10)	Ginsenoside Rh2 (RS11)	Formononetin (HQ11)	Quercetin (HQ17)
AKT1	−7.7	—	—	—	−8.0
ICAM1	−10.0	—	—	—	−10.0
JUN	−5.8	—	—	−5.5	−5.7
PPARG	−8.1	−9.2	—	−7.4	−8.4
PTGS2	−9.0	−9.1	−8.5	−7.4	−9.1
TNF	−6.6	—	−6.6	—	−6.9

## Data Availability

All the supporting data can be found in the supplemental information.

## Abbreviations

MPE: Malignant pleural effusion
GAD: Ginseng and Astragalus decoction
TCMSP: Traditional Chinese Medicine Systems Pharmacology Database Analysis Platform
OMIM: Online Mendelian Inheritance in Man
GO: Gene Ontology
KEGG: Kyoto Encyclopedia of Genes and Genomes
TCM: Traditional Chinese medicine
OB: Oral bioavailability
DL: Druglikeness
AKT1: AKT serine/threonine kinase 1
ICAM1: Intercellular adhesion molecule 1
JUN: Jun proto-oncogene
PPARG: Peroxisome proliferator-activated receptor gamma
PTGS2: Prostaglandin-endoperoxide synthase 2
TNF: Tumor necrosis factor
FBXW7: F-Box and WD repeat domain containing 7
QCN: Quercetin
KPL: Kaempferol
FNA: Frutinone A
GRH2: Ginsenoside RH2
FNN: Formononetin.

## Supplementary Materials

Supplementary Materials Supplementary Table 1: UniPort gene protein name. Supplemental Table 2: the docking setting.
