# Supplementary material for: The Mechanism of Ginseng and Astragalus Decoction in the Treatment of Malignant Pleural Effusion Based on Network Pharmacology and Molecular Docking Technology
Source: Evid Based Complement Alternat Med. 2022 Mar 15;2022:7731402. doi: 10.1155/2022/7731402 (PMC8941536; doi:10.1155/2022/7731402)
Supplement: Supplementary Materials — Supplementary Table 1: UniPort gene protein name. Supplemental Table 2: the docking setting. [file 7731402.f1.pdf]

Supplementary table 1: uniport gene protein name

Entry	Protein names	Gene names			
Q96R72	Olfactory receptor 4K3	OR4K3	Progesterone receptor	PGR	PGR
Q9UKL2	Olfactory receptor 52A1	OR52A1	Nitric oxide synthase, inducible	NOS2	NOS2
Q9H205	Olfactory receptor 2AG1	OR2AG1	Prostaglandin G/H synthase 1	PTGS1	PTGS1
Q8NGN2	Olfactory receptor 10S1	OR10S1	Androgen receptor	AR	AR
Q8NGC1	Olfactory receptor 11G2	OR11G2	Sodium channel protein type 5 subunit alpha	SCN5A	SCN5A
O60502	Protein O-GlcNAcase	OGA	Prostaglandin G/H synthase 2	PTGS2	PTGS2
Q8NGX2	Olfactory receptor 2T35	OR2T35	Estrogen receptor beta	ESR2	ESR2
A6NGY5	Olfactory receptor 51F1	OR51F1	Dipeptidyl peptidase IV	#N/A	#N/A
Q8NGE3	Olfactory receptor 10P1	OR10P1	Heat shock protein HSP 90	#N/A	#N/A
Q6IF63	Olfactory receptor 52W1	OR52W1	Cell division protein kinase 2	#N/A	#N/A
Q9BXB4	Oxysterol-binding protein-related protein 11	OSBPL11	Serine/threonine-protein kinase Chk1	CHEK1	CHEK1
Q9UJX0	Oxidative stress-induced growth inhibitor 1	OSGIN1	Trypsin-1	PRSS1	PRSS1
Q6A1A2	Putative 3-phosphoinositide-dependent protein kinase 2	PDPK2P	Nuclear receptor coactivator 2	NCOA2	NCOA2
Q96S52	GPI transamidase component PIG-S	PIGS	Calmodulin	#N/A	#N/A
P13686	Tartrate-resistant acid phosphatase type 5	ACP5	Progesterone receptor	PGR	PGR
P17980	26S proteasome regulatory subunit 6A	PSMC3	Nuclear receptor coactivator 2	NCOA2	NCOA2
Q5THK1	Protein PRR14L	PRR14L	Muscarinic acetylcholine receptor M3	CHRM3	CHRM3
P0CG20	Proline-rich protein 35	PRR35	Muscarinic acetylcholine receptor M1	CHRM1	CHRM1
Q9ULL5	Proline-rich protein 12	PRR12	Gamma-aminobutyric-acid receptor alpha-2 subunit	#N/A	#N/A
Q6NWY9	Pre-mRNA-processing factor 40 homolog B	PRPF40B	Gamma-aminobutyric-acid receptor alpha-3 subunit	MAOB	MAOB
Q5TYX0	PRAME family member 5	PRAMEF5	Muscarinic acetylcholine receptor M2	MAOA	MAOA
Q5VTA0	PRAME family member 17	PRAMEF17	Alpha-1B adrenergic receptor	PRKACA	PRKACA
Q00LT1	Photoreceptor disk component PRCD	PRCD	Gamma-aminobutyric acid receptor subunit alpha-1	GABRA1	GABRA1
Q9UMR5	Lysosomal thioesterase PPT2	PPT2	Glutamate receptor 2	GRIA2	GRIA2
P0DJ07	Protein PET100 homolog, mitochondrial	PET100	Gamma-aminobutyric-acid receptor subunit alpha-6	#N/A	#N/A
Q8WV60	Pentatricopeptide repeat-containing protein 2, mitochondrial	PTCD2	Gamma-aminobutyric-acid receptor alpha-5 subunit	#N/A	#N/A
Q96EY7	Pentatricopeptide repeat domain-containing protein 3, mitochondrial	PTCD3	Ig gamma-1 chain C region	#N/A	#N/A
Q7RTS3	Pancreas transcription factor 1 subunit alpha	PTF1A	Alcohol dehydrogenase 1B	HTR2A	HTR2A
P20962	Parathymosin	PTMS	Alcohol dehydrogenase 1C	ADH1C	ADH1C
Q92530	Proteasome inhibitor PI31 subunit	PSMF1	Lysozyme	GABRA3	GABRA3
O14957	Cytochrome b-c1 complex subunit 10	UQCR11	Nicotinate-nucleotide--dimethylbenzimidazole phosphoribosyltransferase	#N/A	#N/A
Q6ZRP7	Sulfhydryl oxidase 2	QSOX2	Prostaglandin G/H synthase 1	PTGS1	PTGS1
Q8TBF2	Prostamide/prostaglandin F synthase	PRXL2B	Sodium channel protein type 5 subunit alpha	SCN5A	SCN5A
Q86YV0	RAS protein activator like-3	RASAL3	Prostaglandin G/H synthase 2	**CHRNA7**	CHRNA7
Q8NFJ5	Retinoic acid-induced protein 3	GPRC5A	Retinoic acid receptor RXR-alpha	RXRA	RXRA
Q14206	Calcipressin-2	RCAN2	CGMP-inhibited 3',5'-cyclic phosphodiesterase A	PDE3A	PDE3A
Q9Y2P8	RNA 3'-terminal phosphate cyclase-like protein	RCL1	Sodium-dependent noradrenaline transporter	SLC6A2	SLC6A2
A4D1S5	Ras-related protein Rab-19	RAB19	Cytochrome P450-cam	#N/A	#N/A
P61106	Ras-related protein Rab-14	RAB14	Progesterone receptor	PGR	PGR
O95716	Ras-related protein Rab-3D	RAB3D	Nitric oxide synthase, inducible	**PIK3CG**	PIK3CG
Q8N4Z0	Ras-related protein Rab-42	RAB42	Prostaglandin G/H synthase 1	PTGS1	PTGS1
P49795	Regulator of G-protein signaling 19	RGS19	Estrogen receptor	ESR1	ESR1
Q7L0Q8	Rho-related GTP-binding protein RhoU	RHOU	Androgen receptor	**DRD1**	DRD1

Q9BQ08	Resistin-like beta	RETNLB	Peroxisome proliferator activated receptor gamma	#N/A	#N/A
Q9HB40	Retinoid-inducible serine carboxypeptidase	SCPEP1	Prostaglandin G/H synthase 2	PTGS2	PTGS2
Q8NHV9	Rhox homeobox family member 1	RHOXF1	mRNA of Protein-tyrosine phosphatase, non-receptor type 1	#N/A	#N/A
P07949	Proto-oncogene tyrosine-protein kinase receptor Ret	RET	Estrogen receptor beta	**GABRA2**	GABRA2
Q8TAC1	Rieske domain-containing protein	RFESD	Dipeptidyl peptidase IV	#N/A	#N/A
Q9UJD0	Regulating synaptic membrane exocytosis protein 3	RIMS3	Mitogen-activated protein kinase 14	MAPK14	MAPK14
P50914	60S ribosomal protein L14	RPL14	Glycogen synthase kinase-3 beta	**HTR2A**	HTR2A
Q969Q0	60S ribosomal protein L36a-like	RPL36AL	Heat shock protein HSP 90	**GABRA5**	GABRA5
Q9NSD7	Relaxin-3 receptor 1	RXFP3	Cell division protein kinase 2	#N/A	#N/A
Q9HCK4	Roundabout homolog 2	ROBO2	Phosphatidylinositol-4,5-bisphosphate 3-kinase catalytic subunit, gamma isoform	**GABRA3**	GABRA3
Q9H1D9	DNA-directed RNA polymerase III subunit RPC6	POLR3F	mRNA of PKA Catalytic Subunit C-alpha	#N/A	#N/A
P35372	Mu-type opioid receptor	OPRM1	Trypsin-1	PRSS1	PRSS1
O14581	Olfactory receptor 7A17	OR7A17	Proto-oncogene serine/threonine-protein kinase Pim-1	#N/A	#N/A
Q99489	D-aspartate oxidase	DDO	Cyclin-A2	CCNA2	CCNA2
Q8NGM8	Olfactory receptor 6M1	OR6M1	Nuclear receptor coactivator 2	NCOA2	NCOA2
Q9GZK6	Olfactory receptor 2J1	OR2J1	Calmodulin	#N/A	#N/A
Q149M9	NACHT domain- and WD repeat-containing protein 1	NWD1	Glycogen phosphorylase, muscle form	PYGM	PYGM
Q8NGE7	Olfactory receptor 9K2	OR9K2	Peroxisome proliferator activated receptor delta	#N/A	#N/A
Q6N063	2-oxoglutarate and iron-dependent oxygenase domain-containing protein 2	OGFOD2	Serine/threonine-protein kinase Chk1	CHEK1	CHEK1
O95158	Neurexophilin-4	NXPH4	Aldose reductase	#N/A	#N/A
A6NMZ5	Olfactory receptor 4C45	OR4C45	Nuclear receptor coactivator 1	NCOA1	NCOA1
Q8NGL6	Olfactory receptor 4A15	OR4A15	Coagulation factor VII	F7	F7
Q8NGC8	Olfactory receptor 11H7	OR11H7	Thrombin	#N/A	#N/A
Q06190	Serine/threonine-protein phosphatase 2A regulatory subunit B'' subunit alpha	PPP2R3A	Nitric-oxide synthase, endothelial	#N/A	#N/A
A6NJZ3	Olfactory receptor 6C65	OR6C65	Acetylcholinesterase	ACHE	ACHE
Q3SX64	Outer dense fiber protein 3-like protein 2	ODF3L2	Gamma-aminobutyric acid receptor subunit alpha-1	GABRA1	GABRA1
P00480	Ornithine transcarbamylase, mitochondrial	OTC	Amine oxidase [flavin-containing] B	**TGFB1**	TGFB1
Q9NVV4	Poly	MTPAP	Glutamate receptor 2	GRIA2	GRIA2
Q9NPH6	Odorant-binding protein 2b	OBP2B	Cytochrome P450-cam	#N/A	#N/A
Q8NDF8	Terminal nucleotidyltransferase 4B	TENT4B	Transcription factor p65	RELA	RELA
P0DN37	Peptidyl-prolyl cis-trans isomerase A-like 4G	PPIAL4G	Xanthine dehydrogenase/oxidase	XDH	XDH
O15381	Nuclear valosin-containing protein-like	NVL	Neutrophil cytosol factor 1	NCF1	NCF1
A8MZH6	Putative oocyte-secreted protein 1 homolog	OOSP1	Oxidized low-density lipoprotein receptor 1	OLR1	OLR1
Q8NGH7	Olfactory receptor 52L1	OR52L1	Nitric oxide synthase, inducible	NOS2	NOS2
O95340	Bifunctional 3'-phosphoadenosine 5'-phosphosulfate synthase 2	PAPSS2	Prostaglandin G/H synthase 1	PTGS1	PTGS1
Q6ZVX9	Membrane progestin receptor epsilon	PAQR9	Muscarinic acetylcholine receptor M3	CHRM3	CHRM3
P57723	Poly	PCBP4	Thrombin	#N/A	#N/A
Q8TEZ7	Membrane progestin receptor beta	PAQR8	Muscarinic acetylcholine receptor M1	CHRM1	CHRM1
Q460N5	Protein mono-ADP-ribosyltransferase PARP14	PARP14	Estrogen receptor	ESR1	ESR1
Q96QU1	Protocadherin-15	PCDH15	Beta-1 adrenergic receptor	ADRB1	ADRB1
Q6P474	Putative pyridoxal-dependent decarboxylase domain-containing protein 2	PDXDC2P	Sodium channel protein type 5 subunit alpha	**PIK3CG**	PIK3CG
P13667	Protein disulfide-isomerase A4	PDIA4	Prostaglandin G/H synthase 2	PTGS2	PTGS2
P09466	Glycodelin	PAEP	Nitric-oxide synthase, endothelial	#N/A	#N/A

Q13087	Protein disulfide-isomerase A2	PDIA2	5-hydroxytryptamine receptor 3A	HTR3A	HTR3A
Q15084	Protein disulfide-isomerase A6	PDIA6	Alpha-2C adrenergic receptor	ADRA2C	ADRA2C
Q53GL7	Protein mono-ADP-ribosyltransferase PARP10	PARP10	Retinoic acid receptor RXR-alpha	RXRA	RXRA
Q5MIZ7	Serine/threonine-protein phosphatase 4 regulatory subunit 3B	PPP4R3B	Acetylcholinesterase	ACHE	ACHE
A4QPH2	Putative phosphatidylinositol 4-kinase alpha-like protein P2	PI4KAP2	CGMP-inhibited 3',5'-cyclic phosphodiesterase A	PDE3A	PDE3A
Q92561	Phytanoyl-CoA hydroxylase-interacting protein	PHYHIP	Alpha-1B adrenergic receptor	ADRA1B	ADRA1B
Q8IYJ0	PILR alpha-associated neural protein	PIANP	Beta-2 adrenergic receptor	ADRB2	ADRB2
Q8WWW0	Ras association domain-containing protein 5	RASSF5	Alpha-1D adrenergic receptor	**PPARG**	PPARG
Q06609	DNA repair protein RAD51 homolog 1	RAD51	Mu-type opioid receptor	OPRM1	OPRM1
O75452	Retinol dehydrogenase 16	RDH16	Gamma-aminobutyric acid receptor subunit alpha-1	GABRA1	GABRA1
Q9UBK7	Rab-like protein 2A	RABL2A	Trypsin-1	PRSS1	PRSS1
Q9UKM9	RNA-binding protein Raly	RALY	Nuclear receptor coactivator 2	NCOA2	NCOA2
A0AV96	RNA-binding protein 47	RBM47	Calmodulin	#N/A	#N/A
P46060	Ran GTPase-activating protein 1	RANGAP1	Nitric oxide synthase, inducible	**DPP4**	DPP4
Q96IS3	Retina and anterior neural fold homeobox protein 2	RAX2	Prostaglandin G/H synthase 1	PTGS1	PTGS1
P61572	Endogenous retrovirus group K member 19 Rec protein	ERVK-19	Dopamine D1 receptor	#N/A	#N/A
Q9BRK0	Receptor expression-enhancing protein 2	REEP2	Muscarinic acetylcholine receptor M3	CHRM3	CHRM3
A1A4S6	Rho GTPase-activating protein 10	ARHGAP10	Thrombin	#N/A	#N/A
P61020	Ras-related protein Rab-5B	RAB5B	Potassium voltage-gated channel subfamily H member 2	KCNH2	KCNH2
Q6ZS17	Rho family-interacting cell polarization regulator 1	RIPOR1	Muscarinic acetylcholine receptor M1	CHRM1	CHRM1
P15927	Replication protein A 32 kDa subunit	RPA2	Estrogen receptor	ESR1	ESR1
P62888	60S ribosomal protein L30	RPL30	Androgen receptor	AR	AR
Q9P015	39S ribosomal protein L15, mitochondrial	MRPL15	Beta-1 adrenergic receptor	ADRB1	ADRB1
Q9HAT0	Ropporin-1A	ROPN1	Sodium channel protein type 5 subunit alpha	SCN5A	SCN5A
Q9Y6N7	Roundabout homolog 1	ROBO1	Peroxisome proliferator activated receptor gamma	#N/A	#N/A
Q13151	Heterogeneous nuclear ribonucleoprotein A0	HNRNPA0	Coagulation factor Xa	#N/A	#N/A
P60602	Reactive oxygen species modulator 1	ROMO1	Muscarinic acetylcholine receptor M5	CHRM5	CHRM5
Q13464	Rho-associated protein kinase 1	ROCK1	Prostaglandin G/H synthase 2	PTGS2	PTGS2
Q9P1U0	DNA-directed RNA polymerase I subunit RPA12	POLR1H	Nitric-oxide synthase, endothelial	#N/A	#N/A
Q3B726	DNA-directed RNA polymerase I subunit RPA43	POLR1F	Alpha-2C adrenergic receptor	ADRA2C	ADRA2C
Q9NV58	E3 ubiquitin-protein ligase RNF19A	RNF19A	Muscarinic acetylcholine receptor M4	CHRM4	CHRM4
P78346	Ribonuclease P protein subunit p30	RPP30	Retinoic acid receptor RXR-alpha	RXRA	RXRA
Q9BUL9	Ribonuclease P protein subunit p25	RPP25	Delta-type opioid receptor	OPRD1	OPRD1
Q8N6L0	Protein KASH5	KASH5	CGMP-inhibited 3',5'-cyclic phosphodiesterase A	PDE3A	PDE3A
Q8IUC0	Keratin-associated protein 13-1	KRTAP13-1	5-hydroxytryptamine 2A receptor	#N/A	#N/A
P60368	Keratin-associated protein 10-2	KRTAP10-2	Alpha-1A adrenergic receptor	ADRA1A	ADRA1A
P43629	Killer cell immunoglobulin-like receptor 3DL1	KIR3DL1	Muscarinic acetylcholine receptor M2	CHRM2	CHRM2
Q6P1M3	LLGL scribble cell polarity complex component 2	LLGL2	Alpha-1B adrenergic receptor	ADRA1B	ADRA1B
O14910	Protein lin-7 homolog A	LIN7A	Sodium-dependent dopamine transporter	SLC6A3	SLC6A3

Q6ZMV7	Leucine-, glutamate- and lysine-rich protein 1	LEKR1	Beta-2 adrenergic receptor	ADRB2	ADRB2
P31025	Lipocalin-1	LCN1	Alpha-1D adrenergic receptor	ADRA1D	ADRA1D
Q6UWP7	Lysocardiolipin acyltransferase 1	LCLAT1	Sodium-dependent serotonin transporter	SLC6A4	SLC6A4
P48163	NADP-dependent malic enzyme	ME1	Estrogen receptor beta	ESR2	ESR2
O60942	mRNA-capping enzyme	RNGTT	Gamma-aminobutyric acid receptor subunit alpha-1	GABRA1	GABRA1
A0A075B6I0	Immunoglobulin lambda variable 8-61	IGLV8-61	Dipeptidyl peptidase IV	#N/A	#N/A
Q8IW41	MAP kinase-activated protein kinase 5	MAPKAPK5	Mitogen-activated protein kinase 14	MAPK14	MAPK14
Q5TCX8	Mitogen-activated protein kinase kinase kinase 21	MAP3K21	Glycogen synthase kinase-3 beta	GSK3B	GSK3B
Q86YJ5	E3 ubiquitin-protein ligase MARCHF9	MARCHF9	Heat shock protein HSP 90	#N/A	#N/A
Q8NFP4	MAM domain-containing glycosylphosphatidylinositol anchor protein 1	MDGA1	Cell division protein kinase 2	#N/A	#N/A
Q7KZI7	Serine/threonine-protein kinase MARK2	MARK2	Serine/threonine-protein kinase Chk1	CHEK1	CHEK1
Q68D85	Natural cytotoxicity triggering receptor 3 ligand 1	NCR3LG1	mRNA of PKA Catalytic Subunit C-alpha	#N/A	#N/A
Q96KX0	Lysozyme-like protein 4	LYZL4	Retinoic acid receptor RXR-beta	RXRB	RXRB
Q9NXC5	GATOR complex protein MIOS	MIOS	Trypsin-1	PRSS1	PRSS1
Q96PC5	Melanoma inhibitory activity protein 2	MIA2	Proto-oncogene serine/threonine-protein kinase Pim-1	#N/A	#N/A
Q96MC6	Hippocampus abundant transcript 1 protein	MFSD14A	Cyclin-A2	CCNA2	CCNA2
Q09327	Beta-1,4-mannosyl-glycoprotein 4-beta-N-acetylglucosaminyltransferase	MGAT3	Nuclear receptor coactivator 2	NCOA2	NCOA2
Q86XE3	Calcium uptake protein 3, mitochondrial	MICU3	Calcium-activated potassium channel subunit alpha 1	#N/A	#N/A
Q9Y605	MORF4 family-associated protein 1	MRFAP1	Calmodulin	#N/A	#N/A
Q96H12	Myb/SANT-like DNA-binding domain-containing protein 3	MSANTD3	Prostaglandin G/H synthase 2	PTGS2	PTGS2
O95563	Mitochondrial pyruvate carrier 2	MPC2	DNA topoisomerase II	#N/A	#N/A
P49006	MARCKS-related protein	MARCKSL1	Nuclear receptor coactivator 2	NCOA2	NCOA2
Q14149	MORC family CW-type zinc finger protein 3	MORC3	Nitric oxide synthase, inducible	NOS2	NOS2
Q9NV56	MRG/MORF4L-binding protein	MRGBP	Prostaglandin G/H synthase 1	PTGS1	PTGS1
Q8TDS7	Mas-related G-protein coupled receptor member D	MRGPRD	Muscarinic acetylcholine receptor M3	CHRM3	CHRM3
Q8N5Y2	Male-specific lethal 3 homolog	MSL3	Thrombin	#N/A	#N/A
O96033	Molybdopterin synthase sulfur carrier subunit	MOCS2	Muscarinic acetylcholine receptor M1	CHRM1	CHRM1
Q8NCK7	Monocarboxylate transporter 11	SLC16A11	Estrogen receptor	ESR1	ESR1
Q99551	Transcription termination factor 1, mitochondrial	MTERF1	Sodium channel protein type 5 subunit alpha	SCN5A	SCN5A
B1AL46	NUT family member 2E	NUTM2E	Prostaglandin G/H synthase 2	PTGS2	PTGS2
Q8NGS3	Olfactory receptor 1J1	OR1J1	5-hydroxytryptamine receptor 3A	HTR3A	HTR3A
P03886	NADH-ubiquinone oxidoreductase chain 1	MT-ND1	Retinoic acid receptor RXR-alpha	RXRA	RXRA
O95006	Olfactory receptor 2F2	OR2F2	Acetylcholinesterase	ACHE	ACHE
A6NHN0	Otolin-1	OTOL1	Alpha-1B adrenergic receptor	ADRA1B	ADRA1B
Q8WV22	Non-structural maintenance of chromosomes element 1 homolog	NSMCE1	Beta-2 adrenergic receptor	ADRB2	ADRB2
Q96R09	Olfactory receptor 5B2	OR5B2	Alpha-1D adrenergic receptor	ADRA1D	ADRA1D
Q8NGA0	Olfactory receptor 7G1	OR7G1	Gamma-aminobutyric acid receptor subunit alpha-1	GABRA1	GABRA1
Q8NH18	Olfactory receptor 5J2	OR5J2	Heat shock protein HSP 90	#N/A	#N/A
Q8NGK1	Olfactory receptor 51G1	OR51G1	Neuronal acetylcholine receptor protein, alpha-7 chain	#N/A	#N/A
Q8NH55	Olfactory receptor 52E5	OR52E5	Trypsin-1	PRSS1	PRSS1
Q8NGZ0	Olfactory receptor 2AJ1	OR2AJ1	Nuclear receptor coactivator 2	NCOA2	NCOA2
Q8NGR1	Olfactory receptor 13A1	OR13A1	Nuclear receptor coactivator 1	NCOA1	NCOA1

Q96SU4	Oxysterol-binding protein-related protein 9	OSBPL9	Calmodulin	#N/A	#N/A
Q8WWB5	PIH1 domain-containing protein 2	PIH1D2	Muscarinic acetylcholine receptor M4	CHRM4	CHRM4
Q9NRD5	PRKCA-binding protein	PICK1	Prostaglandin G/H synthase 2	PTGS2	PTGS2
P50542	Peroxisomal targeting signal 1 receptor	PEX5	Vascular endothelial growth factor receptor 2	KDR	KDR
O14494	Phospholipid phosphatase 1	PLPP1	Hepatocyte growth factor receptor	MET	MET
P55058	Phospholipid transfer protein	PLTP	Heat shock protein HSP 90	#N/A	#N/A
Q9UIG5	Psoriasis susceptibility 1 candidate gene 1 protein	PSORS1C1	Calcium-activated potassium channel subunit alpha 1	#N/A	#N/A
P23469	Receptor-type tyrosine-protein phosphatase epsilon	PTPRE	Prostaglandin G/H synthase 1	PTGS1	PTGS1
O95456	Proteasome assembly chaperone 1	PSMG1	DNA topoisomerase II	#N/A	#N/A
Q9Y3U8	60S ribosomal protein L36	RPL36	Nitric oxide synthase, inducible	NOS2	NOS2
Q969G2	LIM/homeobox protein Lhx4	LHX4	Prostaglandin G/H synthase 1	PTGS1	PTGS1
A8MVA2	Keratin-associated protein 9-6	KRTAP9-6	Muscarinic acetylcholine receptor M1	CHRM1	CHRM1
P58180	Olfactory receptor 4D2	OR4D2	Estrogen receptor	ESR1	ESR1
Q8NGP3	Olfactory receptor 5M9	OR5M9	Androgen receptor	AR	AR
P0DN80	Olfactory receptor 5H8	OR5H8	Peroxisome proliferator activated receptor gamma	#N/A	#N/A
Q99623	Prohibitin-2	PHB2	Prostaglandin G/H synthase 2	PTGS2	PTGS2
O43688	Phospholipid phosphatase 2	PLPP2	Retinoic acid receptor RXR-alpha	RXRA	RXRA
P63132	Endogenous retrovirus group K member 113 Pol protein	HERVK_113	CGMP-inhibited 3',5'-cyclic phosphodiesterase A	PDE3A	PDE3A
Q9Y3C6	Peptidyl-prolyl cis-trans isomerase-like 1	PPIL1	Alpha-1A adrenergic receptor	ADRA1A	ADRA1A
P20264	POU domain, class 3, transcription factor 3	POU3F3	Sodium-dependent dopamine transporter	SLC6A3	SLC6A3
O00764	Pyridoxal kinase	PDXK	Beta-2 adrenergic receptor	ADRB2	ADRB2
P11086	Phenylethanolamine N-methyltransferase	PNMT	Sodium-dependent serotonin transporter	SLC6A4	SLC6A4
Q9P212	1-phosphatidylinositol 4,5-bisphosphate phosphodiesterase epsilon-1	PLCE1	Estrogen receptor beta	ESR2	ESR2
Q8NI37	Protein phosphatase PTC7 homolog	PPTC7	Dipeptidyl peptidase IV	#N/A	#N/A
Q00889	Pregnancy-specific beta-1-glycoprotein 6	PSG6	Mitogen-activated protein kinase 14	MAPK14	MAPK14
O95997	Securin	PTTG1	Glycogen synthase kinase-3 beta	GSK3B	GSK3B
Q13332	Receptor-type tyrosine-protein phosphatase S	PTPRS	Heat shock protein HSP 90	#N/A	#N/A
P0DJH9	Protein RD3-like	RD3L	Cell division protein kinase 2	#N/A	#N/A
Q9HBD1	Roquin-2	RC3H2	Amine oxidase [flavin-containing] B	#N/A	#N/A
Q9UL26	Ras-related protein Rab-22A	RAB22A	Serine/threonine-protein kinase Chk1	CHEK1	CHEK1
Q9H2T7	Ran-binding protein 17	RANBP17	mRNA of PKA Catalytic Subunit C-alpha	#N/A	#N/A
Q6IQ22	Ras-related protein Rab-12	RAB12	Trypsin-1	PRSS1	PRSS1
P61019	Ras-related protein Rab-2A	RAB2A	Proto-oncogene serine/threonine-protein kinase Pim-1	#N/A	#N/A
Q7Z6E9	E3 ubiquitin-protein ligase RBBP6	RBBP6	Cyclin-A2	CCNA2	CCNA2
Q9NS91	E3 ubiquitin-protein ligase RAD18	RAD18	Calmodulin	#N/A	#N/A
P61006	Ras-related protein Rab-8A	RAB8A	cAMP-dependent protein kinase inhibitor alpha	PKIA	PKIA
Q68EM7	Rho GTPase-activating protein 17	ARHGAP17	Thrombin	#N/A	#N/A
Q9NWB1	RNA binding protein fox-1 homolog 1	RBFOX1	Nitric-oxide synthase, endothelial	#N/A	#N/A
Q6PCD5	E3 ubiquitin-protein ligase RFWD3	RFWD3	Acetylcholinesterase	ACHE	ACHE
P35250	Replication factor C subunit 2	RFC2	Beta-lactamase	#N/A	#N/A
P50120	Retinol-binding protein 2	RBP2	Transcription factor AP-1	JUN	JUN
Q9BZI7	Regulator of nonsense transcripts 3B	UPF3B	Peroxisome proliferator-activated receptor gamma	PPARG	PPARG
Q9H920	RING finger protein 121	RNF121	Interleukin-4	IL4	IL4
Q9Y508	E3 ubiquitin-protein ligase	RNF114	NAD-dependent deacetylase sirtuin-1	#N/A	#N/A
Q96MT1	RING finger protein 145	RNF145	ATP synthase subunit beta, mitochondrial	ATP5F1B	ATP5F1B
P36578	60S ribosomal protein L4	RPL4	NADH-ubiquinone oxidoreductase chain 6	MT-ND6	MT-ND6

P12271	Retinaldehyde-binding protein 1	RLBP1	3 beta-hydroxysteroid dehydrogenase/Delta 5-->4-isomerase type 2	HSD3B2	HSD3B2
Q68DV7	E3 ubiquitin-protein ligase RNF43	RNF43	3 beta-hydroxysteroid dehydrogenase/Delta 5-->4-isomerase type 1	HSD3B1	HSD3B1
P47736	Rap1 GTPase-activating protein 1	RAP1GAP	Nitric oxide synthase, inducible	NOS2	NOS2
P14678	Small nuclear ribonucleoprotein-associated proteins B and B'	SNRPB	Prostaglandin G/H synthase 1	PTGS1	PTGS1
P62081	40S ribosomal protein S7	RPS7	Estrogen receptor	ESR1	ESR1
P82650	28S ribosomal protein S22, mitochondrial	MRPS22	Androgen receptor	AR	AR
Q8N4F4	Steroid transmembrane transporter SLC22A24	SLC22A24	Peroxisome proliferator activated receptor gamma	#N/A	#N/A
Q8TBP6	Solute carrier family 25 member 40	SLC25A40	Prostaglandin G/H synthase 2	PTGS2	PTGS2
O95156	Neurexophilin-2	NXPH2	Retinoic acid receptor RXR-alpha	RXRA	RXRA
Q8N146	Olfactory receptor 8H3	OR8H3	CGMP-inhibited 3',5'-cyclic phosphodiesterase A	PDE3A	PDE3A
Q9UKK6	NTF2-related export protein 1	NXT1	Estrogen receptor beta	ESR2	ESR2
P0C628	Olfactory receptor 5AC1	OR5AC1	Dipeptidyl peptidase IV	#N/A	#N/A
Q8NGK0	Olfactory receptor 51G2	OR51G2	Mitogen-activated protein kinase 14	MAPK14	MAPK14
Q6ZVC0	Neuronal tyrosine-phosphorylated phosphoinositide-3-kinase adaptor 1	NYAP1	Glycogen synthase kinase-3 beta	GSK3B	GSK3B
Q96R47	Olfactory receptor 2A14	OR2A14	Heat shock protein HSP 90	#N/A	#N/A
Q8NGN4	Olfactory receptor 10G9	OR10G9	Cell division protein kinase 2	#N/A	#N/A
Q8NH73	Olfactory receptor 4S2	OR4S2	Serine/threonine-protein kinase Chk1	CHEK1	CHEK1
Q9ULC6	Protein-arginine deiminase type-	PADI1	mRNA of PKA Catalytic Subunit C-alpha	#N/A	#N/A
Q9Y5G2	Protocadherin gamma-B2	PCDHGB2	Trypsin-1	PRSS1	PRSS1
P0DN26	Peptidyl-prolyl cis-trans isomerase A-like 4F	PPIAL4F	Proto-oncogene serine/threonine-protein kinase Pim-1	#N/A	#N/A
Q4G0U5	Cilia- and flagella-associated protein 221	CFAP221	Cyclin-A2	CCNA2	CCNA2
Q8IY49	Monocyte to macrophage differentiation factor 2	MMD2	Nuclear receptor coactivator 2	NCOA2	NCOA2
O95263	High affinity cAMP-specific and IBMX-insensitive 3',5'-cyclic phosphodiesterase 8B	PDE8B	Calmodulin	#N/A	#N/A
P51160	Cone cGMP-specific 3',5'-cyclic phosphodiesterase subunit alpha'	PDE6C	Beta-2 adrenergic receptor	ADRB2	ADRB2
Q9NPG4	Protocadherin-12	PCDH12	Nitric oxide synthase, inducible	NOS2	NOS2
Q9Y5E2	Protocadherin beta-7	PCDHB7	Prostaglandin G/H synthase 1	PTGS1	PTGS1
O15350	Tumor protein p73	TP73	Androgen receptor	AR	AR
Q15120		PDK3	Peroxisome proliferator activated receptor gamma	#N/A	#N/A
P23219	Prostaglandin G/H synthase 1	PTGS1	Prostaglandin G/H synthase 2	PTGS2	PTGS2
Q9UKK3	Protein mono-ADP-ribosyltransferase PARP4	PARP4	Heat shock protein HSP 90	#N/A	#N/A
Q86V59	Paraneoplastic antigen-like protein 8A	PNMA8A	Phosphatidylinositol-4,5-bisphosphate 3-kinase catalytic subunit, gamma isoform	#N/A	#N/A
O43316	Paired box protein Pax-4	PAX4	mRNA of PKA Catalytic Subunit C-alpha	#N/A	#N/A
Q8N4L2	Type 2 phosphatidylinositol 4,5-bisphosphate 4-phosphatase	PIP4P2	Nuclear receptor coactivator 2	NCOA2	NCOA2
Q15124	Phosphoglucomutase-like protein 5	PGM5	Dipeptidyl peptidase IV	#N/A	#N/A
Q8N414	PiggyBac transposable element-derived protein 5	PGBD5	Trypsin-1	PRSS1	PRSS1
P15309	Prostatic acid phosphatase	ACP3	Progesterone receptor	PGR	PGR
Q9P1A2	Putative serine/threonine-protein phosphatase 4 regulatory subunit 1-like	PPP4R1L	Thrombin	#N/A	#N/A
Q13401	Putative postmeiotic segregation increased 2-like protein 3	PMS2P3	Muscarinic acetylcholine receptor M1	CHRM1	CHRM1
Q96BZ4	5'-3' exonuclease PLD4	PLD4	Nitric-oxide synthase, endothelial	#N/A	#N/A
P40967	Melanocyte protein PMEL	PMEL	Gamma-aminobutyric-acid receptor alpha-2 subunit	#N/A	#N/A
O15297	Protein phosphatase 1D	PPM1D	Acetylcholinesterase	ACHE	ACHE

Q02325	Plasminogen-like protein B	PLGLB1	Sodium-dependent noradrenaline transporter	SLC6A2	SLC6A2
P26678	Cardiac phospholamban	PLN	Muscarinic acetylcholine receptor M2	CHRM2	CHRM2
P13796	Plastin-2	LCP1	Alpha-1B adrenergic receptor	ADRA1B	ADRA1B
O14818	Proteasome subunit alpha type-7	PSMA7	Gamma-aminobutyric acid receptor subunit alpha-1	GABRA1	GABRA1
P27918	Properdin	CFP	DNA topoisomerase II	#N/A	#N/A
Q8N8Z3	Putative uncharacterized protein DIP2C-AS1	DIP2C-AS1	Coagulation factor VII	F7	F7
Q13046	Putative pregnancy-specific beta-1-glycoprotein 7	PSG7	Calmodulin	#N/A	#N/A
O75832	26S proteasome non-ATPase regulatory subunit 10	PSMD10	Transcription factor p65	RELA	RELA
Q15262	Receptor-type tyrosine-protein phosphatase kappa	PTPRK	Inhibitor of nuclear factor kappa-B kinase subunit beta	IKBKB	IKBKB
Q59GN2	Putative 60S ribosomal protein L39-like 5	RPL39P5	RAC-alpha serine/threonine-protein kinase	AKT1	AKT1
A8MTL3	RING finger protein 212B	RNF212B	Apoptosis regulator Bcl-2	BCL2	BCL2
Q6NUJ5	PWWP domain-containing protein 2B	PWWP2B	Apoptosis regulator BAX	BAX	BAX
P01116	GTPase KRas	KRAS	Tumor necrosis factor	TNF	TNF
P48304	Lithostathine-1-beta	REG1B	Transcription factor AP-1	JUN	JUN
Q69383	Endogenous retrovirus group K member 6 Rec protein	ERVK-6	Activator of 90 kDa heat shock protein ATPase homolog 1	AHSA1	AHSA1
P04090	Prorelaxin H2	RLN2	Caspase-3	CASP3	CASP3
Q5U651	Ras-interacting protein 1	RASIP1	Mitogen-activated protein kinase 8	MAPK8	MAPK8
Q15291	Retinoblastoma-binding protein 5	RBBP5	Xanthine dehydrogenase/oxidase	XDH	XDH
Q9NYW8	RB-associated KRAB zinc finger protein	RBAK	Interstitial collagenase	MMP1	MMP1
P0C0E4	Ras-related protein Rab-40A-like	RAB40AL	Signal transducer and activator of transcription 1-alpha/beta	STAT1	STAT1
Q13123	Protein Red	IK	Cell division control protein 2 homolog	#N/A	#N/A
Q7Z6P3	Ras-related protein Rab-44	RAB44	Peroxisome proliferator-activated receptor gamma	PPARG	PPARG
Q53QZ3	Rho GTPase-activating protein 15	ARHGAP15	Heme oxygenase 1	HMOX1	HMOX1
Q96IC2	RNA exonuclease 5	REXO5	Cytochrome P450 3A4	CYP3A4	CYP3A4
O14827	Ras-specific guanine nucleotide-releasing factor 2	RASGRF2	Cytochrome P450 1A2	CYP1A2	CYP1A2
Q9H0H5	Rac GTPase-activating protein 1	RACGAP1	Cytochrome P450 1A1	CYP1A1	CYP1A1
Q5W5W9	Regulated endocrine-specific protein 18	RESP18	Intercellular adhesion molecule 1	ICAM1	ICAM1
P10745	Retinol-binding protein 3	RBP3	E-selectin	SELE	SELE
P52758	2-iminobutanoate/2-iminopropanoate deaminase	RIDA	Vascular cell adhesion protein 1	VCAM1	VCAM1
Q96L33	Rho-related GTP-binding protein RhoV	RHOV	Nuclear receptor subfamily 1 group I member 2	NR1I2	NR1I2
P18621	60S ribosomal protein L17	RPL17	Cytochrome P450 1B1	CYP1B1	CYP1B1
Q96EL3	39S ribosomal protein L53, mitochondrial	MRPL53	Arachidonate 5-lipoxygenase	#N/A	#N/A
P62945	60S ribosomal protein L41	RPL41	Hyaluronan synthase 2	HAS2	HAS2
O15514	DNA-directed RNA polymerase II subunit RPB4	POLR2D	Glutathione S-transferase P	GSTP1	GSTP1
Q8NGI6	Olfactory receptor 4D10	OR4D10	Aryl hydrocarbon receptor	AHR	AHR
Q8NGP0	Olfactory receptor 4C13	OR4C13	26S proteasome non-ATPase regulatory subunit 3	PSMD3	PSMD3
Q8NGV6	Olfactory receptor 5H6	OR5H6	Solute carrier family 2, facilitated glucose transporter member 4	SLC2A4	SLC2A4
P48739	Phosphatidylinositol transfer protein beta isoform	PITPNB	Nuclear receptor subfamily 1 group I member 3	NR1I3	NR1I3
O00264	Membrane-associated progesterone receptor component 1	PGRMC1	Insulin receptor	INSR	INSR
Q5SV97	PGC-1 and ERR-induced regulator in muscle protein 1	PERM1	Type I iodothyronine deiodinase	DIO1	DIO1
O15428	Putative PIN1-like protein	PIN1P1	Serine/threonine-protein phosphatase 2B catalytic subunit alpha isoform	PPP3CA	PPP3CA
Q6NUP7	Serine/threonine-protein phosphatase 4 regulatory subunit 4	PPP4R4	Peroxidase C1A	#N/A	#N/A

Q6XQN6	Nicotinate phosphoribosyltransferase	NAPRT	Glutathione S-transferase Mu 1	GSTM1	GSTM1
Q9H792	Inactive tyrosine-protein kinase PEAK1	PEAK1	Glutathione S-transferase Mu 2	GSTM2	GSTM2
Q8N7P1	Inactive phospholipase D5	PLD5	Aldo-keto reductase family 1 member C3	AKR1C3	AKR1C3
Q5VZY2	Phospholipid phosphatase 4	PLPP4	Antileukoproteinase	SLPI	SLPI
Q9Y244	Proteasome maturation protein	POMP	Cell division protein kinase 2	#N/A	#N/A
Q9P0L9	Polycystic kidney disease 2-like 1 protein	PKD2L1	Thrombin	#N/A	#N/A
Q0VAA5	PI-PLC X domain-containing protein 2	PLCXD2	Glycogen synthase kinase-3 beta	GSK3B	GSK3B
Q86YW0	1-phosphatidylinositol 4,5-bisphosphate phosphodiesterase zeta-1	PLCZ1	DNA topoisomerase II	#N/A	#N/A
Q9NRZ7	1-acyl-sn-glycerol-3-phosphate acyltransferase gamma	AGPAT3	Prostaglandin G/H synthase 2	PTGS2	PTGS2
Q3B8N5	Prospero homeobox protein 2	PROX2	Retinoic acid receptor RXR-alpha	RXRA	RXRA
Q8N271	Prominin-2	PROM2	Heat shock protein HSP 90	#N/A	#N/A
P21108	Ribose-phosphate pyrophosphokinase 3	PRPS1L1	Trypsin-1	PRSS1	PRSS1
P28072	Proteasome subunit beta type-6	PSMB6	Prostaglandin G/H synthase 1	PTGS1	PTGS1
Q9BRX5	DNA replication complex GINS protein PSF3	GINS3	Androgen receptor	AR	AR
O00232	26S proteasome non-ATPase regulatory subunit 12	PSMD12	Peroxisome proliferator activated receptor gamma	#N/A	#N/A
Q9Y3L5	Ras-related protein Rap-2c	RAP2C	Prostaglandin G/H synthase 2	PTGS2	PTGS2
Q6UW15	Regenerating islet-derived protein 3-gamma	REG3G	Heat shock protein HSP 90	#N/A	#N/A
A0A1B0GV85	Reelin domain-containing protein 1	REELD1	Phosphatidylinositol-4,5-bisphosphate 3-kinase catalytic subunit, gamma isoform	#N/A	#N/A
Q9P260	RAB11-binding protein RELCH	RELCH	Nuclear receptor coactivator 2	NCOA2	NCOA2
Q7Z6M1	Rab9 effector protein with kelch motifs	RABEPK	Dipeptidyl peptidase IV	#N/A	#N/A
Q14964	Ras-related protein Rab-39A	RAB39A	Aldose reductase	#N/A	#N/A
Q6ZRY4	RNA-binding protein with multiple splicing 2	RBPMS2	Trypsin-1	PRSS1	PRSS1
P20339	Ras-related protein Rab-5A	RAB5A	DNA topoisomerase II	#N/A	#N/A
O14559	Rho GTPase-activating protein 33	ARHGAP33	Thrombin	#N/A	#N/A
A6NI28	Rho GTPase-activating protein 42	ARHGAP42	Potassium voltage-gated channel subfamily H member 2	KCNH2	KCNH2
Q96EV2	RNA-binding protein 33	RBM33	Sodium channel protein type 5 subunit alpha	SCN5A	SCN5A
O14924	Regulator of G-protein signaling 12	RGS12	Coagulation factor Xa	#N/A	#N/A
O94955	Rho-related BTB domain-containing protein 3	RHOBTB3	Beta-2 adrenergic receptor	ADRB2	ADRB2
Q92963	GTP-binding protein Rit1	RIT1	Stromelysin-1	MMP3	MMP3
Q96L21	60S ribosomal protein L10-like	RPL10L	mRNA of PKA Catalytic Subunit C-alpha	#N/A	#N/A
Q96DV4	39S ribosomal protein L38, mitochondrial	MRPL38	Coagulation factor VII	F7	F7
Q7L0R7	RING finger protein 44	RNF44	Nitric-oxide synthase, endothelial	#N/A	#N/A
Q969K3	E3 ubiquitin-protein ligase RNF34	RNF34	Retinoic acid receptor RXR-alpha	RXRA	RXRA
Q96GF1	E3 ubiquitin-protein ligase	RNF185	Acetylcholinesterase	ACHE	ACHE
Q96T23	Remodeling and spacing factor 1	RSF1	Gamma-aminobutyric acid receptor subunit alpha-1	GABRA1	GABRA1
Q9NPB1	5'	NT5M	Amine oxidase [flavin-containing] B	#N/A	#N/A
Q9NZP2	Olfactory receptor 6C2	OR6C2	Transcription factor p65	RELA	RELA
Q8NGR9	Olfactory receptor 1N2	OR1N2	Epidermal growth factor receptor	EGFR	EGFR
Q8NGL3	Olfactory receptor 5D14	OR5D14	RAC-alpha serine/threonine-protein kinase	AKT1	AKT1
P47883	Putative olfactory receptor 3A4	OR3A4P	Vascular endothelial growth factor A	VEGFA	VEGFA
Q9P121	Neurotrimin	NTM	G1/S-specific cyclin-D1	CCND1	CCND1
Q13492	Phosphatidylinositol-binding clathrin assembly protein	PICALM	Apoptosis regulator Bcl-2	BCL2	BCL2
P78356	Phosphatidylinositol 5-phosphate 4-kinase type-2 beta	PIP4K2B	Bcl-2-like protein 1	BCL2L1	BCL2L1

Q8TCD6	Pyridoxal phosphate phosphatase PHOSPHO2	PHOSPHO2	Proto-oncogene c-Fos	FOS	FOS
Q9BSJ6	Protein PIMREG	PIMREG	Cyclin-dependent kinase inhibitor 1	CDKN1A	CDKN1A
Q5SRE7	Phytanoyl-CoA dioxygenase domain-containing protein 1	PHYHD1	Eukaryotic translation initiation factor 6	EIF6	EIF6
E7EU14	Protein PPP5D1	PPP5D1	Apoptosis regulator BAX	BAX	BAX
O60486	Plexin-C1	PLXNC1	Caspase-9	CASP9	CASP9
P16112	Aggrecan core protein	ACAN	Urokinase-type plasminogen activator	PLAU	PLAU
P30086	Phosphatidylethanolamine-binding protein 1	PEBP1	72 kDa type IV collagenase	MMP2	MMP2
Q8IXQ8	PDZ domain-containing protein 9	PDZD9	Matrix metalloproteinase-9	MMP9	MMP9
O15031	Plexin-B2	PLXNB2	Mitogen-activated protein kinase 1	MAPK1	MAPK1
O95744	Putative postmeiotic segregation increased 2-like protein 2	PMS2P2	Interleukin-10	IL10	IL10
Q8TCS8	Polyribonucleotide nucleotidyltransferase 1, mitochondrial	PNPT1	Pro-epidermal growth factor	EGF	EGF
Q9UG56	Phosphatidylserine decarboxylase proenzyme, mitochondrial	PISD	Retinoblastoma-associated protein	RB1	RB1
Q8NG27	E3 ubiquitin-protein ligase Praja-1	PJA1	Tumor necrosis factor	TNF	TNF
Q6MZQ0	Proline-rich protein 5-like	PRR5L	Transcription factor AP-1	JUN	JUN
Q15147	1-phosphatidylinositol 4,5-bisphosphate phosphodiesterase beta-4	PLCB4	Interleukin-6	IL6	IL6
Q86WR7	Proline and serine-rich protein 2	PROSER2	Cyclin-dependent kinase inhibitor 2A, isoforms 1/2/3	#N/A	#N/A
Q6ZRP0	Proline-rich protein 23C	PRR23C	Activator of 90 kDa heat shock protein ATPase homolog 1	AHSA1	AHSA1
O75360	Homeobox protein prophet of Pit-1	PROP1	Caspase-3	CASP3	CASP3
B1ATL7	Proline-rich protein 32	PRR32	Cellular tumor antigen p53	TP53	TP53
P79522	Proline-rich protein 3	PRR3	ETS domain-containing protein Elk-1	ELK1	ELK1
O60828	Polyglutamine-binding protein 1	PQBP1	NF-kappa-B inhibitor alpha	NFKBIA	NFKBIA
Q9NQV6	PR domain zinc finger protein 10	PRDM10	NADPH--cytochrome P450 reductase	POR	POR
Q5VV67	Peroxisome proliferator-activated receptor gamma coactivator-related protein 1	PPRC1	Ornithine decarboxylase	ODC1	ODC1
P57071	PR domain zinc finger protein 15	PRDM15	Xanthine dehydrogenase/oxidase	XDH	XDH
Q9NYI0	PH and SEC7 domain-containing protein 3	PSD3	Caspase-8	CASP8	CASP8
P22234	Multifunctional protein ADE2	PAICS	DNA topoisomerase 1	TOP1	TOP1
Q96A99	Pentraxin-4	PTX4	RAF proto-oncogene serine/threonine-protein kinase	RAF1	RAF1
Q06323	Proteasome activator complex subunit 1	PSME1	Superoxide dismutase [Cu-Zn]	#N/A	#N/A
A0A096LP55	Cytochrome b-c1 complex subunit 6-like, mitochondrial	UQCRHL	Protein kinase C alpha type	PRKCA	PRKCA
Q14644	Ras GTPase-activating protein 3	RASA3	Interstitial collagenase	MMP1	MMP1
O14807	Ras-related protein M-Ras	MRAS	Hypoxia-inducible factor 1-alpha	HIF1A	HIF1A
Q15415	RNA-binding motif protein, Y chromosome, family 1 member [illegible]	RBMY1F	Signal transducer and activator of transcription 1-alpha/beta	STAT1	STAT1
P04808	Prorelaxin H1	RLN1	Protein CBFA2T1	RUNX1T1	RUNX1T1
P61574	Endogenous retrovirus group K member 113 Rec protein	HERVK_113	Probable E3 ubiquitin-protein ligase HERC5	#N/A	#N/A
Q96S59	Ran-binding protein 9	RANBP9	Cell division control protein 2 homolog	#N/A	#N/A
Q9NX57	Ras-related protein Rab-20	RAB20	78 kDa glucose-regulated protein	#N/A	#N/A
Q9UNA1	Rho GTPase-activating protein 26	ARHGAP26	Receptor tyrosine-protein kinase erbB-2	ERBB2	ERBB2
O75677	Ret finger protein-like 1	RFPL1	Peroxisome proliferator-activated receptor gamma	PPARG	PPARG
P49796	Regulator of G-protein signaling 3	RGS3	Acetyl-CoA carboxylase 1	ACACA	ACACA
Q9UFD9	RIMS-binding protein 3A	RIMBP3	Heme oxygenase 1	HMOX1	HMOX1
Q8NFH8	RalBP1-associated Eps domain-containing protein 2	REPS2	Cytochrome P450 3A4	CYP3A4	CYP3A4
Q9BYD3	39S ribosomal protein L4, mitochondrial	MRPL4	Cytochrome P450 1A2	CYP1A2	CYP1A2
O75792	Ribonuclease H2 subunit A	RNASEH2A	Caveolin-1	CAV1	CAV1
Q6ZMZ0	E3 ubiquitin-protein ligase	RNF19B	Myc proto-oncogene protein	MYC	MYC
P07998	Ribonuclease pancreatic	RNASE1	Tissue factor	F3	F3

Q86VF7	Nebulin-related-anchoring protein	NRAP	Gap junction alpha-1 protein	GJA1	GJA1
Q8NGN1	Olfactory receptor 6T1	OR6T1	Cytochrome P450 1A1	CYP1A1	CYP1A1
Q9GZY6	Linker for activation of T-cells family member 2	LAT2	Intercellular adhesion molecule 1	ICAM1	ICAM1
Q8NH64	Olfactory receptor 51A7	OR51A7	Interleukin-1 beta	IL1B	IL1B
A6NHA9	Olfactory receptor 4C46	OR4C46	C-C motif chemokine 2	CCL2	CCL2
Q9H255	Olfactory receptor 51E2	OR51E2	E-selectin	SELE	SELE
P78380	Oxidized low-density lipoprotein receptor 1	OLR1	Vascular cell adhesion protein 1	VCAM1	VCAM1
Q9H341	Olfactory receptor 51M1	OR51M1	Prostaglandin E2 receptor EP3 subtype	PTGER3	PTGER3
Q8IWE2	Protein NOXP20	FAM114A1	Interleukin-8	CXCL8	CXCL8
Q86UW2	Organic solute transporter subunit beta	SLC51B	Protein kinase C beta type	PRKCB	PRKCB
Q8NGF3	Olfactory receptor 51D1	OR51D1	Baculoviral IAP repeat-containing protein 5	BIRC5	BIRC5
Q8N2R0	Protein odd-skipped-related 2	OSR2	Dual oxidase 2	DUOX2	DUOX2
P56373	P2X purinoceptor 3	P2RX3	Nitric oxide synthase, endothelial	NOS3	NOS3
Q8NGI3	Olfactory receptor 56B1	OR56B1	Heat shock protein beta-1	HSPB1	HSPB1
Q8TCB6	Olfactory receptor 51E1	OR51E1	Transforming growth factor beta-1	#N/A	#N/A
A6NIJ9	Olfactory receptor 6C70	OR6C70	Estrogen sulfotransferase	#N/A	#N/A
P02818	Osteocalcin	BGLAP	Maltase-glucoamylase, intestinal	MGAM	MGAM
A6ND48	Olfactory receptor 14I1	OR14I1	Interleukin-2	IL2	IL2
Q8NGF9	Olfactory receptor 4X2	OR4X2	Nuclear receptor subfamily 1 group I member 2	NR1I2	NR1I2
Q9HBE1	POZ-, AT hook-, and zinc finger-containing protein 1	PATZ1	Cytochrome P450 1B1	CYP1B1	CYP1B1
Q92535	Phosphatidylinositol N-acetylglucosaminyltransferase subunit C	PIGC	G2/mitotic-specific cyclin-B1	CCNB1	CCNB1
Q8N6C7	Putative uncharacterized protein encoded by MIR7-3HG	MIR7-3HG	Tissue-type plasminogen activator	PLAT	PLAT
Q02962	Paired box protein Pax-2	PAX2	Thrombomodulin	THBD	THBD
Q9Y6X2	E3 SUMO-protein ligase PIAS3	PIAS3	Plasminogen activator inhibitor 1	SERPINE1	SERPINE1
Q14863	POU domain, class 6, transcription factor 1	POU6F1	Collagen alpha-1(I) chain	#N/A	#N/A
Q6P3X8	PiggyBac transposable element-derived protein 2	PGBD2	Interferon gamma	IFNG	IFNG
P20618	Proteasome subunit beta type-1	PSMB1	Arachidonate 5-lipoxygenase	#N/A	#N/A
P60673	Profilin-3	PFN3	Phosphatidylinositol-3,4,5-trisphosphate 3-phosphatase and dual-specificity protein phosphatase PTEN	#N/A	#N/A
Q99946	Proline-rich transmembrane protein 1	PRRT1	Interleukin-1 alpha	IL1A	IL1A
Q16849	Receptor-type tyrosine-protein phosphatase-like N	PTPRN	Myeloperoxidase	MPO	MPO
P10586	Receptor-type tyrosine-protein phosphatase F	PTPRF	DNA topoisomerase 2-alpha	TOP2A	TOP2A
Q15008	26S proteasome non-ATPase regulatory subunit 6	PSMD6	Neutrophil cytosol factor 1	NCF1	NCF1
P11217	Glycogen phosphorylase, muscle form	PYGM	ATP-binding cassette sub-family G member 2	#N/A	#N/A
P06737	Glycogen phosphorylase, liver form	PYGL	Hyaluronan synthase 2	HAS2	HAS2
A1KZ92	Peroxidasin-like protein	PXDNL	Glutathione S-transferase P	GSTP1	GSTP1
P18754	Regulator of chromosome condensation	RCC1	Nuclear factor erythroid 2-related factor 2	NFE2L2	NFE2L2
Q15293	Reticulocalbin-1	RCN1	NAD(P)H dehydrogenase [quinone] 1	#N/A	#N/A
Q15382	GTP-binding protein Rheb	RHEB	Poly [ADP-ribose] polymerase 1	#N/A	#N/A
O14966	Ras-related protein Rab-7L1	RAB29	Aryl hydrocarbon receptor	AHR	AHR
Q15771	Ras-related protein Rab-30	RAB30	26S proteasome non-ATPase regulatory subunit 3	PSMD3	PSMD3
Q17R89	Rho GTPase-activating protein 44	ARHGAP44	Solute carrier family 2, facilitated glucose transporter member 4	SLC2A4	SLC2A4
P51149	Ras-related protein Rab-7a	RAB7A	Collagen alpha-1(III) chain	#N/A	#N/A
Q5JT25	Ras-related protein Rab-41	RAB41	DNA gyrase subunit B	#N/A	#N/A
P0DJD1	RANBP2-like and GRIP domain-containing protein 2	RGPD2	C-X-C motif chemokine 11	CXCL11	CXCL11
Q6ZWI9	Ret finger protein-like 4B	RFPL4B	C-X-C motif chemokine 2	CXCL2	CXCL2

Q5UIP0	Telomere-associated protein RIF1	RIF1	DDB1- and CUL4-associated factor 5	DCAF5	DCAF5
P26373	60S ribosomal protein L13	RPL13	Nuclear receptor subfamily 1 group I member 3	NR1I3	NR1I3
P61353	60S ribosomal protein L27	RPL27	Serine/threonine-protein kinase Chk2	CHEK2	CHEK2
Q9Y535	DNA-directed RNA polymerase III subunit RPC8	POLR3H	Insulin receptor	INSR	INSR
Q9UHV5	Rap guanine nucleotide exchange factor-like 1	RAPGEFL1	Claudin-4	CLDN4	CLDN4
A6NCQ9	RING finger protein 222	RNF222	Peroxisome proliferator-activated receptor alpha	PPARA	PPARA
P32242	Homeobox protein OTX1	OTX1	Peroxisome proliferator-activated receptor delta	PPARD	PPARD
Q15620	Olfactory receptor 8B8	OR8B8	Heat shock factor protein 1	HSF1	HSF1
O00628	Peroxisomal targeting signal 2 receptor	PEX7	C-reactive protein	CRP	CRP
Q6UXB8	Peptidase inhibitor 16	PI16	C-X-C motif chemokine 10	CXCL10	CXCL10
Q96RG2	PAS domain-containing serine/threonine-protein kinase	PASK	Inhibitor of nuclear factor kappa-B kinase subunit alpha	CHUK	CHUK
Q9NQ66	1-phosphatidylinositol 4,5-bisphosphate phosphodiesterase beta-1	PLCB1	Osteopontin	SPP1	SPP1
Q99633	Pre-mRNA-splicing factor 18	PRPF18	Runt-related transcription factor 2	RUNX2	RUNX2
Q92786	Prospero homeobox protein 1	PROX1	Ras association domain-containing protein 1	RASSF1	RASSF1
Q86XR5	Proline-rich membrane anchor 1	PRIMA1	Transcription factor E2F1	E2F1	E2F1
Q96QR8	Transcriptional activator protein Pur-beta	PURB	Transcription factor E2F2	E2F2	E2F2
P06401	Progesterone receptor	PGR	Prostatic acid phosphatase	ACP3	ACP3
O15067	Phosphoribosylformylglycinamidine synthase	PFAS	Cathepsin D	CTSD	CTSD
Q14914	Prostaglandin reductase 1	PTGR1	Insulin-like growth factor-binding protein 3	IGFBP3	IGFBP3
Q9UMZ3	Phosphatidylinositol phosphatase PTPRQ	PTPRQ	Insulin-like growth factor II	IGF2	IGF2
P35236	Tyrosine-protein phosphatase non-receptor type 7	PTPN7	CD40 ligand	CD40LG	CD40LG
P01270	Parathyroid hormone	PTH	Interferon regulatory factor 1	IRF1	IRF1
Q6ISU1	Pre T-cell antigen receptor alpha	PTCRA	Receptor tyrosine-protein kinase erbB-3	ERBB3	ERBB3
Q3MIT2	tRNA pseudouridine synthase Pus10	PUS10	Serum paraoxonase/arylesterase 1	PON1	PON1
Q2TAK8	PWWP domain-containing DNA repair factor 3A	PWWP3A	Type I iodothyronine deiodinase	DIO1	DIO1
Q7Z4M0	Meiotic recombination protein REC114	REC114	Procollagen C-endopeptidase enhancer 1	PCOLCE	PCOLCE
Q9H6Z4	Ran-binding protein 3	RANBP3	Puromycin-sensitive aminopeptidase	NPEPPS	NPEPPS
P60763	Ras-related C3 botulinum toxin substrate 3	RAC3	Hexokinase-2	HK2	HK2
Q9NRW1	Ras-related protein Rab-6B	RAB6B	Homeobox protein Nkx-3.1	NKX3-1	NKX3-1
Q9GZR2	RNA exonuclease 4	REXO4	Ras GTPase-activating protein 1	RASA1	RASA1
Q6ZVN8	Hemojuvelin	HJV	Peroxidase C1A	#N/A	#N/A
Q7Z5H3	Rho GTPase-activating protein 22	ARHGAP22	Glutathione S-transferase Mu 1	GSTM1	GSTM1
P41220	Regulator of G-protein signaling 2	RGS2	Glutathione S-transferase Mu 2	GSTM2	GSTM2
P48378	DNA-binding protein RFX2	RFX2			
A6NFN3	RNA binding protein fox-1 homolog 3	RBFOX3			
O00212	Rho-related GTP-binding protein RhoD	RHOD			
Q6BDI9	Rab15 effector protein	REP15			
P61586	Transforming protein RhoA	RHOA			
Q6NUM9	All-trans-retinol 13,14-reductase	RETSAT			
Q9BWE0	Replication initiator 1	REPIN1			
P61254	60S ribosomal protein L26	RPL26			
Q9BYC8	39S ribosomal protein L32, mitochondrial	MRPL32			
Q9BYD6	39S ribosomal protein L1, mitochondrial	MRPL1			
Q15070	Mitochondrial inner membrane protein OXA1L	OXA1L			

Q96R48	Olfactory receptor 2A5	OR2A5			
Q8NGG7	Olfactory receptor 8A1	OR8A1			
O76000	Putative olfactory receptor 2B3	OR2B3			
O00168	Phospholemman	FXYD1			
Q13670	Putative postmeiotic segregation increased 2-like protein 11	PMS2P11			
Q96Q06	Perilipin-4	PLIN4			
P53041	Serine/threonine-protein phosphatase 5	PPP5C			
O75817	Ribonuclease P protein subunit p20	POP7			
O43660	Pleiotropic regulator 1	PLRG1			
Q8NEB9	Phosphatidylinositol 3-kinase catalytic subunit type 3	PIK3C3			
P16298	Serine/threonine-protein phosphatase 2B catalytic subunit beta isoform	PPP3CB			
Q15435	Protein phosphatase 1 regulatory subunit 7	PPP1R7			
Q99943	1-acyl-sn-glycerol-3-phosphate acyltransferase alpha	AGPAT1			
Q9NWT1	p21-activated protein kinase-interacting protein 1	PAK1IP1			
P81277	Prolactin-releasing peptide	PRLH			
Q7Z5A4	Putative serine protease 42	PRSS42P			
Q8IV56	Proline-rich protein 15	PRR15			
Q9NRI7	Putative pancreatic polypeptide 2	PPY2P			
A8MZF0	Proline-rich protein 33	PRR33			
Q99436	Proteasome subunit beta type-7	PSMB7			
Q9BXM0	Periaxin	PRX			
Q9NQV8	PR domain zinc finger protein 8	PRDM8			
Q96I23	Protein preY, mitochondrial	PYURF			
Q13635	Protein patched homolog 1	PTCH1			
Q13882	Protein-tyrosine kinase 6	PTK6			
Q9Y272	Dexamethasone-induced Ras-related protein 1	RASD1			
Q92698	DNA repair and recombination protein RAD54-like	RAD54L			
Q8IZ40	REST corepressor 2	RCOR2			
Q9NW64	Pre-mRNA-splicing factor RBM22	RBM22			
Q13972	Ras-specific guanine nucleotide-releasing factor 1	RASGRF1			
Q9NS28	Regulator of G-protein signaling 18	RGS18			
Q9BWJ2	Putative uncharacterized protein encoded by RHPN1-AS1	RHPN1-AS1			
Q8WZ73	E3 ubiquitin-protein ligase rififylin	RFFL			
Q9UBD6	Ammonium transporter Rh type C	RHCG			
O43566	Regulator of G-protein signaling 14	RGS14			
Q9BSD3	RAD9, HUS1, RAD1-interacting nuclear orphan protein 1	RHNO1			
Q5TAB7	Protein ripply2	RIPPLY2			
A6NNM3	RIMS-binding protein 3B	RIMBP3B			
Q8TEB7	E3 ubiquitin-protein ligase	RNF128			
P36954	DNA-directed RNA polymerase II subunit RPB9	POLR2I			
Q9NW08	DNA-directed RNA polymerase III subunit RPC2	POLR3B			
O15446	DNA-directed RNA polymerase I subunit RPA34	POLR1G			
O15318	DNA-directed RNA polymerase III subunit RPC7	POLR3G			
Q5GAN4	Probable inactive ribonuclease-like protein 12	RNASE12			
Q6ZRF8	RING finger protein 207	RNF207			
Q9Y3C5	RING finger protein 11	RNF11			
Q07001	Acetylcholine receptor subunit delta	CHRND			

Q9Y615	Actin-like protein 7A	ACTL7A			
P41595	5-hydroxytryptamine receptor 2B	HTR2B			
Q15172	Serine/threonine-protein phosphatase 2A 56 kDa regulatory subunit alpha isoform	PPP2R5A			
P28566	5-hydroxytryptamine receptor 1E	HTR1E			
Q8N9L9	Peroxisomal succinyl-coenzyme A thioesterase	ACOT4			
P41238	C->U-editing enzyme APOBEC-1	APOBEC1			
O95870	Phosphatidylserine lipase ABHD16A	ABHD16A			
Q9H7C9	Mth938 domain-containing protein	AAMDC			
Q9UBJ2	ATP-binding cassette sub-family D member 2	ABCD2			
Q15758	Neutral amino acid transporter B	SLC1A5			
A9YTQ3	Aryl hydrocarbon receptor repressor	AHRR			
Q5JTC6	APC membrane recruitment protein 1	AMER1			
Q9UKV5	E3 ubiquitin-protein ligase AMFR	AMFR			
P30838	Aldehyde dehydrogenase, dimeric NADP-preferring	ALDH3A1			
P51857	Aldo-keto reductase family 1 member D1	AKR1D1			
P27338	Amine oxidase	MAOB			
P42568	Protein AF-9	MLLT3			
O60488	Long-chain-fatty-acid--CoA ligase 4	ACSL4			
Q9BZ11	Disintegrin and metalloproteinase domain-containing protein 33	ADAM33			
Q8N7Z5	Ankyrin repeat domain-containing protein 31	ANKRD31			
Q9Y672	Dolichyl pyrophosphate Man9GlcNAc2 alpha-1,3-glucosyltransferase	ALG6			
P62330	ADP-ribosylation factor 6	ARF6			
Q13794	Phorbol-12-myristate-13-acetate-induced protein 1	PMAIP1			
Q9Y576	Ankyrin repeat and SOCS box protein 1	ASB1			
O95260	Arginyl-tRNA--protein transferase 1	ATE1			
Q8WWH4	Ankyrin repeat, SAM and basic leucine zipper domain-containing protein 1	ASZ1			
P15848	Arylsulfatase B	ARSB			
O14983	Sarcoplasmic/endoplasmic reticulum calcium ATPase 1	ATP2A1			
Q674R7	Autophagy-related protein 9B	ATG9B			
Q13489	Baculoviral IAP repeat-containing protein 3	BIRC3			
Q8TDH9	Biogenesis of lysosome-related organelles complex 1 subunit 5	BLOC1S5			
Q9BXK5	Bcl-2-like protein 13	BCL2L13			
P46736	Lys-63-specific deubiquitinase BRCC36	BRCC3			
Q6UXG8	Butyrophilin-like protein 9	BTNL9			
P59827	BPI fold-containing family B member 4	BPIFB4			
Q9NPI1	Bromodomain-containing protein 7	BRD7			
P51451	Tyrosine-protein kinase Blk	BLK			
Q15059	Bromodomain-containing protein 3	BRD3			
Q6UXY1	Brain-specific angiogenesis inhibitor 1-associated protein 2-like protein 2	BAIAP2L2			

Q6ZNE5	Beclin 1-associated autophagy-related key regulator	ATG14			
Q9HBU1	Homeobox protein BarH-like 1	BARX1			
Q9UIG0	Tyrosine-protein kinase BAZ1B	BAZ1B			
Q9HD36	Bcl-2-like protein 10	BCL2L10			
Q9Y2D0	Carbonic anhydrase 5B, mitochondrial	CA5B			
Q6ZV80	Uncharacterized protein C2orf91	C2orf91			
O95810	Caveolae-associated protein 2	CAVIN2			
Q9H7X2	Uncharacterized protein C1orf115	C1orf115			
P40123	Adenylyl cyclase-associated protein 2	CAP2			
P02745	Complement C1q subcomponent subunit A	C1QA			
Q6P1W5	Uncharacterized protein C1orf94	C1orf94			
Q8N6N3	UPF0690 protein C1orf52	C1orf52			
O43570	Carbonic anhydrase 12	CA12			
Q4G0X9	Coiled-coil domain-containing protein 40	CCDC40			
Q86Z23	Complement C1q-like protein 4	C1QL4			
Q6DHV5	Protein CC2D2B	CC2D2B			
Q8N6G1	Putative uncharacterized protein encoded by LINC00337	LINC00337			
Q8ND61	Uncharacterized protein C3orf20	C3orf20			
Q494R4	Coiled-coil domain-containing protein 153	CCDC153			
P22676	Calretinin	CALB2			
Q8WXS5	Voltage-dependent calcium channel gamma-8 subunit	CACNG8			
A0A1B0GVG4	Coiled-coil domain-containing protein 194	CCDC194			
Q66K79	Carboxypeptidase Z	CPZ			
Q4G0S7	Coiled-coil domain-containing protein 152	CCDC152			
A6NKD9	Coiled-coil domain-containing protein 85C	CCDC85C			
P27701	CD82 antigen	CD82			
Q5U5Z8	Cytosolic carboxypeptidase 2	AGBL2			
Q6ZTR5	Cilia- and flagella-associated protein 47	CFAP47			
Q9BXF3	Cat eye syndrome critical region protein 2	CECR2			
Q9P2X8	Putative uncharacterized protein encoded by LINC00474	LINC00474			
Q8N865	Uncharacterized protein C7orf31	C7orf31			
Q6UXB4	C-type lectin domain family 4 member G	CLEC4G			
Q13057	Bifunctional coenzyme A synthase	COASY			
Q8IYT2	Cap-specific mRNA	CMTR2			
Q96NU0	Contactin-associated protein-like 3B	CNTNAP3B			
Q02388	Collagen alpha-1	COL7A1			
Q6IPW1	Uncharacterized protein C11orf71	C11orf71			
Q5SWW7	Uncharacterized protein C10orf55	C10orf55			
Q8TDQ1	CMRF35-like molecule 1	CD300LF			
Q8TCG5	Carnitine O-palmitoyltransferase 1, brain isoform	CPT1C			
Q96L46	Calpain small subunit 2	CAPNS2			
P41240	Tyrosine-protein kinase CSK	CSK			
P16220	Cyclic AMP-responsive element-binding protein 1	CREB1			
Q96A83	Collagen alpha-1	COL26A1			
Q9NRD9	Dual oxidase 1	DUOX1			
Q14181	DNA polymerase alpha subunit B	POLA2			
Q9H6B9	Epoxide hydrolase 3	EPHX3			
Q14576	ELAV-like protein 3	ELAVL3			
O60739	Eukaryotic translation initiation factor 1b	EIF1B			
P11474	Steroid hormone receptor ERR1	ESRRA			

P41162	ETS translocation variant 3	ETV3			
Q5RGS3	Protein FAM74A1	FAM74A1			
Q13158	FAS-associated death domain protein	FADD			
Q8IYT1	Protein FAM71A	FAM71A			
P23141	Liver carboxylesterase 1	CES1			
A0A1B0GVM5	Embryonic testis differentiation protein homolog C	ETDC			
Q9BZJ8	G-protein coupled receptor 61	GPR61			
P00451	Coagulation factor VIII	F8			
P01588	Erythropoietin	EPO			
P81408	Protein FAM189B	FAM189B			
Q96MZ4	Protein FAM218A	FAM218A			
P00488	Coagulation factor XIII A chain	F13A1			
Q9H0X4	Protein FAM234A	FAM234A			
Q96MY7	Protein FAM161B	FAM161B			
Q15910	Histone-lysine N-methyltransferase EZH2	EZH2			
Q92637	High affinity immunoglobulin gamma Fc receptor IB	FCGR1B			
Q8NEA4	F-box only protein 36	FBXO36			
Q8N539	Fibrinogen C domain-containing protein 1	FIBCD1			
P11362	Fibroblast growth factor receptor 1	FGFR1			
Q9UKA2	F-box/LRR-repeat protein 4	FBXL4			
Q6UN15	Pre-mRNA 3'-end-processing factor FIP1	FIP1L1			
Q99689	Fasciculation and elongation protein zeta-1	FEZ1			
Q96M96	FYVE, RhoGEF and PH domain-containing protein 4	FGD4			
Q8N461	F-box/LRR-repeat protein 16	FBXL16			
P63130	Endogenous retrovirus group K member 7 Gag polyprotein	ERVK-7			
P0DTW1	G antigen 1	GAGE1			
P0DSO3	G antigen 4	GAGE4			
Q6ZUU3	FOXL2 neighbor protein	FOXL2NB			
Q12951	Forkhead box protein I1	FOXI1			
O95749	Geranylgeranyl pyrophosphate synthase	GGPS1			
P13284	Gamma-interferon-inducible lysosomal thiol reductase	IFI30			
Q14390	Glutathione hydrolase light chain	GGTLC2			
A6NER3	G antigen 12J	GAGE12J			
Q6ZNI0	Beta-1,3-galactosyl-O-glycosyl-glycoprotein beta-1,6-N-acetylglucosaminyltransferase 7	GCNT7			
A0A1W2PPG7	Putative guanine nucleotide-binding protein G	GNG14			
O14764	Gamma-aminobutyric acid receptor subunit delta	GABRD			
Q8IX06	Putative exonuclease GOR	REXO1L1P			
Q3T8J9	GON-4-like protein	GON4L			
P0CG33	Golgin subfamily A member 6D	GOLGA6D			
Q13322	Growth factor receptor-bound protein 10	GRB10			
Q5VY09	Immediate early response gene 5 protein	IER5			
O95872	G patch domain and ankyrin repeat-containing protein 1	GPANK1			
P07359	Platelet glycoprotein Ib alpha chain	GP1BA			
P35052	Glypican-1	GPC1			
P46695	Radiation-inducible immediate-early gene IEX-1	IER3			
P31249	Homeobox protein Hox-D3	HOXD3			
P09016	Homeobox protein Hox-D4	HOXD4			
P13378	Homeobox protein Hox-D8	HOXD8			

Q86YW7	Glycoprotein hormone beta-5	GPHB5			
Q9Y4H4	G-protein-signaling modulator 3	GPSM3			
Q5T6X5	G-protein coupled receptor family C group 6 member A	GPRC6A			
B7ZAQ6	Golgi pH regulator A	GPR89A			
Q8N954	G patch domain-containing protein 11	GPATCH11			
P0CG08	Golgi pH regulator B	GPR89B			
Q2TAA2	Isoamyl acetate-hydrolyzing esterase 1 homolog	IAH1			
P35557	Hexokinase-4	GCK			
P48735	Isocitrate dehydrogenase	IDH2			
Q15053	Uncharacterized protein KIAA0040	KIAA0040			
Q2M1V0	Intestine-specific homeobox	ISX			
Q14695	Uncharacterized protein KIAA0087	KIAA0087			
Q5T013	Putative hydroxypyruvate isomerase	HYI			
A2RU49	Hydroxylysine kinase	HYKK			
P10997	Islet amyloid polypeptide	IAPP			
Q96M11	Hydrolethalus syndrome protein 1	HYLS1			
Q9Y4L1	Hypoxia up-regulated protein 1	HYOU1			
Q9BYI3	Hyccin	FAM126A			
Q14667	Protein KIAA0100	KIAA0100			
Q6NUN7	Jhy protein homolog	JHY			
Q92628	Uncharacterized protein KIAA0232	KIAA0232			
Q12794	Hyaluronidase-1	HYAL1			
Q12891	Hyaluronidase-2	HYAL2			
O43820	Hyaluronidase-3	HYAL3			
Q2M3T9	Hyaluronidase-4	HYAL4			
P38567	Hyaluronidase PH-20	SPAM1			
Q92839	Hyaluronan synthase 1	HAS1			
Q92819	Hyaluronan synthase 2	HAS2			
O00219	Hyaluronan synthase 3	HAS3			
Q86SR1	Polypeptide N-acetylgalactosaminyltransferase 10	GALNT10			
Q5T3I0	G patch domain-containing protein 4	GPATCH4			
Q9Y4A0	Jerky protein homolog-like	JRKL			
P53990	IST1 homolog	IST1			
O75564	Jerky protein homolog	JRK			
P18283	Glutathione peroxidase 2	GPX2			
Q92917	G-patch domain and KOW motifs-containing protein	GPKOW			
Q96SL4	Glutathione peroxidase 7	GPX7			
P52948	Nuclear pore complex protein Nup98-Nup96	NUP98			
Q5XKR4	Homeobox protein orthopedia	OTP			
O00459	Phosphatidylinositol 3-kinase regulatory subunit beta	PIK3R2			
Q9Y6F1	Protein mono-ADP-ribosyltransferase PARP3	PARP3			
O14683	Tumor protein p53-inducible protein 11	TP53I11			
P43115	Prostaglandin E2 receptor EP3 subtype	PTGER3			
Q9Y3B1	PRELI domain containing protein 3B	PRELID3B			
Q9NY27	Serine/threonine-protein phosphatase 4 regulatory subunit 2	PPP4R2			
Q7RTY9	Serine protease 41	PRSS41			
P28074	Proteasome subunit beta type-5	PSMB5			
P0DPQ3	Proline-rich protein 20G	PRR20G			
Q96LQ0	Protein phosphatase 1 regulatory subunit 36	PPP1R36			
O75626	PR domain zinc finger protein 1	PRDM1			
P32119	Peroxiredoxin-2	PRDX2			
Q6UWB4	Serine protease 55	PRSS55			
O95758	Polypyrimidine tract-binding protein 3	PTBP3			

Q16401	26S proteasome non-ATPase regulatory subunit 5	PSMD5			
Q13200	26S proteasome non-ATPase regulatory subunit 2	PSMD2			
P30566	Adenylosuccinate lyase	ADSL			
P27708	CAD protein	CAD			
Q8IZV5	Retinol dehydrogenase 10	RDH10			
Q13702	43 kDa receptor-associated protein of the synapse	RAPSN			
Q9Y5P3	Retinoic acid-induced protein 2	RAI2			
O15539	Regulator of G-protein signaling 5	RGS5			
Q8WVD3	E3 ubiquitin-protein ligase	RNF138			
Q9P0M9	39S ribosomal protein L27, mitochondrial	MRPL27			
Q6P5S7	Ribonuclease kappa	RNASEK			
Q99729	Heterogeneous nuclear ribonucleoprotein A/B	HNRNPAB			
Q96EX2	RING finger and transmembrane domain-containing protein 2	RNFT2			
Q9HAU8	Aminopeptidase RNPEPL1	RNPEPL1			
Q8N4F7	RING finger protein 175	RNF175			
P62280	40S ribosomal protein S11	RPS11			
Q16533	snRNA-activating protein complex subunit 1	SNAPC1			
Q9BQ83	Structure-specific endonuclease subunit SLX1	SLX1A			
Q5VYV7	Protein SLX4IP	SLX4IP			
P49903	Selenide, water dikinase 1	SEPHS1			
Q15036	Sorting nexin-17	SNX17			
Q8N196	Homeobox protein SIX5	SIX5			
O15389	Sialic acid-binding Ig-like lectin 5	SIGLEC5			
Q8TEQ0	Sorting nexin-29	SNX29			
K7EJ46	Small integral membrane protein 22	SMIM22			
P21549	Serine--pyruvate aminotransferase	AGXT			
Q96EA4	Protein Spindly	SPDL1			
Q9HB58	Sp110 nuclear body protein	SP110			
P57073	Transcription factor SOX-8	SOX8			
Q9Y4P9	Sperm flagellar protein 1	SPEF1			
P84022	Mothers against decapentaplegic homolog 3	SMAD3			
O43255	E3 ubiquitin-protein ligase SIAH2	SIAH2			
H3BQB6	Stathmin domain-containing protein 1	STMND1			
Q9NZ72	Stathmin-3	STMN3			
Q9NP77	RNA polymerase II subunit A C-terminal domain phosphatase SSU72	SSU72			
Q9P246	Stromal interaction molecule 2	STIM2			
P02808	Statherin	STATH			
Q9H5I1	Histone-lysine N-methyltransferase SUV39H2	SUV39H2			
Q9UGU0	Transcription factor 20	TCF20			
Q92526	T-complex protein 1 subunit zeta-2	CCT6B			
P0DPI4	T cell receptor beta diversity 1	TRBD1			
O43581	Synaptotagmin-7	SYT7			
Q9BQG1	Synaptotagmin-3	SYT3			
Q9Y5J6	Mitochondrial import inner membrane translocase subunit Tim10 B	TIMM10B			
Q15399	Toll-like receptor 1	TLR1			
Q03403	Trefoil factor 2	TFF2			
P19484	Transcription factor EB	TFEB			
Q99973	Telomerase protein component 1	TEP1			
Q92748	Thyroid hormone-inducible hepatic protein	THRSP			
Q9NUH8	Transmembrane protein 14B	TMEM14B			

Q13009	T-lymphoma invasion and metastasis-inducing protein 1	TIAM1			
P10600	Transforming growth factor beta-3 proprotein	TGFB3			
A2RRL7	Transmembrane protein 213	TMEM213			
Q8WUU8	Transmembrane protein 174	TMEM174			
O75762	Transient receptor potential cation channel subfamily A member 1	TRPA1			
P00439	Phenylalanine-4-hydroxylase	PAH			
Q9H307	Pinin	PNN			
Q86TG7	Retrotransposon-derived protein PEG10	PEG10			
Q494U1	Pleckstrin homology domain-containing family N member 1	PLEKHN1			
O43930	Putative serine/threonine-protein kinase PRKY	PRKY			
Q7RTY5	Serine protease 48	PRSS48			
O60508	Pre-mRNA-processing factor 17	CDC40			
P49642	DNA primase small subunit	PRIM1			
Q58EX7	Puratrophin-1	PLEKHG4			
P58294	Prokineticin-1	PROK1			
P28070	Proteasome subunit beta type-4	PSMB4			
Q8IZ63	Proline-rich protein 22	PRR22			
Q86WC6	Protein phosphatase 1 regulatory subunit 27	PPP1R27			
O43663	Protein regulator of cytokinesis 1	PRC1			
H0Y7S4	Putative PRAME family member	PRAMEF26			
P0DTF9	PTTG1IP family member 2	PTTG1IP2			
P0C881	Radial spoke head 10 homolog B	RSPH10B			
Q96R05	Retinoid-binding protein 7	RBP7			
P78317	E3 ubiquitin-protein ligase RNF4	RNF4			
Q9H9Y6	DNA-directed RNA polymerase I subunit RPA2	POLR1B			
Q92565	Rap guanine nucleotide exchange factor 5	RAPGEF5			
Q8N8N0	E3 ubiquitin-protein ligase	RNF152			
Q8NC42	E3 ubiquitin-protein ligase	RNF149			
Q9H6Y7	E3 ubiquitin-protein ligase	RNF167			
Q9H0F5	E3 ubiquitin-protein ligase RNF38	RNF38			
Q96EU6	Ribosomal RNA processing protein 36 homolog	RRP36			
Q86T96	E3 ubiquitin-protein ligase	RNF180			
Q9H0A6	RING finger protein 32	RNF32			
Q9NYV6	RNA polymerase I-specific transcription initiation factor RRN3	RRN3			
Q7L523	Ras-related GTP-binding protein A	RRAGA			
P62273	40S ribosomal protein S29	RPS29			
A4D0T7	Small integral membrane protein 30	SMIM30			
Q13485	Mothers against decapentaplegic homolog 4	SMAD4			
P35326	Small proline-rich protein 2A	SPRR2A			
Q9GZT3	SRA stem-loop-interacting RNA-binding protein, mitochondrial	SLIRP			
Q5MJ07	Sperm protein associated with the nucleus on the X chromosome N5	SPANXN5			
P0DL12	Small integral membrane protein 17	SMIM17			
O94956	Solute carrier organic anion transporter family member 2B1	SLCO2B1			
Q5MJ68	Speedy protein C	SPDYC			
P48431	Transcription factor SOX-2	SOX2			
Q495Y7	Putative speedy protein E7	SPDYE7P			
Q96GM5	SWI/SNF-related matrix-associated actin-dependent regulator of chromatin subfamily D member 1	SMARCD1			
Q9C004	Protein sprouty homolog 4	SPRY4			

Q15475	Homeobox protein SIX1	SIX1			
Q68D06	Schlafen family member 13	SLFN13			
Q01892	Transcription factor Spi-B	SPIB			
Q969X2	Alpha-N-acetylgalactosaminide alpha-2,6-sialyltransferase 6	ST6GALNAC6			
Q6ZMC9	Sialic acid-binding Ig-like lectin	SIGLEC15			
Q11201	CMP-N-acetylneuraminate-beta-galactosamide-alpha-2,3-sialyltransferase 1	ST3GAL1			
Q8TAV4	Stomatin-like protein 3	STOML3			
Q9H6Q3	Src-like-adapter 2	SLA2			
P58511	Small integral membrane protein 11A	SMIM11A			
Q9UEW8	STE20/SPS1-related proline-alanine-rich protein kinase	STK39			
Q9BWW4	Single-stranded DNA-binding protein 3	SSBP3			
Q9P2R7	Succinate--CoA ligase	SUCLA2			
Q96A44	SPRY domain-containing SOCS box protein 4	SPSB4			
Q8IWZ8	SURP and G-patch domain-containing protein 1	SUGP1			
Q14BN4	Sarcolemmal membrane-associated protein	SLMAP			
Q5VT97	Rho GTPase-activating protein SYDE2	SYDE2			
Q96M53	Protein TBATA	TBATA			
Q96RJ0	Trace amine-associated receptor 1	TAAR1			
P17542	T-cell acute lymphocytic leukemia protein 1	TAL1			
O76070	Gamma-synuclein	SNCG			
O15273	Telethonin	TCAP			
Q4W5G0	Tigger transposable element-derived protein 2	TIGD2			
P35269	General transcription factor IIF subunit 1	GTF2F1			
Q8IY51	Tigger transposable element-derived protein 4	TIGD4			
Q9UL52	Transmembrane protease serine 11E	TMPRSS11E			
Q9BT49	THAP domain-containing protein 7	THAP7			
O75204	Transmembrane protein 127	TMEM127			
Q8N6G2	Testis-expressed protein 26	TEX26			
P13385	Teratocarcinoma-derived growth factor 1	TDGF1			
Q9NYB0	Telomeric repeat-binding factor 2-interacting protein 1	TERF2IP			
Q6UWW9	Transmembrane protein 207	TMEM207			
Q96HV5	Transmembrane protein 41A	TMEM41A			
Q8WY98	Transmembrane protein 234	TMEM234			
Q8NBN3	Transmembrane protein 87A	TMEM87A			
Q9H147	Deoxynucleotidyltransferase terminal-interacting protein 1	DNTTIP1			
A6NK02	Putative tripartite motif-containing protein 75	TRIM75P			
O60858	E3 ubiquitin-protein ligase	TRIM13			
Q9C019	Tripartite motif-containing protein 15	TRIM15			
O00300	Tumor necrosis factor receptor superfamily member 11B	TNFRSF11B			
O94826	Mitochondrial import receptor subunit TOM70	TOMM70			
Q96A61	E3 ubiquitin-protein ligase	TRIM52			
Q7Z403	Transmembrane channel-like protein 6	TMC6			
Q9Y5R8	Trafficking protein particle complex subunit 1	TRAPPC1			

Q9Y5U5	Tumor necrosis factor receptor superfamily member 18	TNFRSF18			
Q96NL1	Transmembrane protein 74	TMEM74			
Q8TBN0	Guanine nucleotide exchange factor for Rab-3A	RAB3IL1			
Q96P65	Pyroglutamylated RF-amide peptide receptor	QRFPR			
Q7Z7A4	PX domain-containing protein kinase-like protein	PXK			
P78406	mRNA export factor	RAE1			
Q9BQ04	RNA-binding protein 4B	RBM4B			
P61224	Ras-related protein Rap-1b	RAP1B			
P51159	Ras-related protein Rab-27A	RAB27A			
P51157	Ras-related protein Rab-28	RAB28			
P15153	Ras-related C3 botulinum toxin substrate 2	RAC2			
Q92928	Putative Ras-related protein Rab-1C	RAB1C			
Q13017	Rho GTPase-activating protein 5	ARHGAP5			
P57729	Ras-related protein Rab-38	RAB38			
Q9P227	Rho GTPase-activating protein 23	ARHGAP23			
Q92930	Ras-related protein Rab-8B	RAB8B			
Q5TG30	Rho GTPase-activating protein 40	ARHGAP40			
Q86YS6	Ras-related protein Rab-43	RAB43			
Q86UA6	RPA-interacting protein	RPAIN			
Q9HD89	Resistin	RETN			
Q99578	GTP-binding protein Rit2	RIT2			
Q9ULI2	Beta-citrylglutamate synthase B	RIMKLB			
O75394	39S ribosomal protein L33, mitochondrial	MRPL33			
P05386	60S acidic ribosomal protein P1	RPLP1			
Q9H7B2	Ribosome production factor 2 homolog	RPF2			
Q8IXW5	Putative RNA polymerase II subunit B1 CTD phosphatase RPAP2	RPAP2			
Q9H1E1	Ribonuclease 7	RNASE7			
Q96EP0	E3 ubiquitin-protein ligase RNF31	RNF31			
A6NIE6	Putative RRN3-like protein RRN3P2	RRN3P2			
Q5GAN3	Probable inactive ribonuclease-like protein 13	RNASE13			
Q6T310	Ras-like protein family member 11A	RASL11A			
Q8IUI4	Putative protein SNX29P2	SNX29P2			
Q9Y3D3	28S ribosomal protein S16, mitochondrial	MRPS16			
Q9UHR5	SAP30-binding protein	SAP30BP			
Q96K37	Solute carrier family 35 member E1	SLC35E1			
P51398	28S ribosomal protein S29, mitochondrial	DAP3			
Q8N488	RING1 and YY1-binding protein	RYBP			
Q9Y467	Sal-like protein 2	SALL2			
P0CK97	Solute carrier family 35 member E2A	SLC35E2A			
Q5VXD3	Sterile alpha motif domain-containing protein 13	SAMD13			
P40879	Chloride anion exchanger	SLC26A3			
P23297	Protein S100-A1	S100A1			
Q6ZMH5	Zinc transporter ZIP5	SLC39A5			
Q01196	Runt-related transcription factor 1	RUNX1			
Q6XE38	Secretoglobin family 1D member	SCGB1D4			
Q86V20	Shieldin complex subunit 2	SHLD2			
P22307	Sterol carrier protein 2	SCP2			
Q9P0V9	Septin-10	SEPTIN10			
P53992	Protein transport protein Sec24C	SEC24C			
Q9Y617	Phosphoserine aminotransferase	PSAT1			
Q5PRF9	Protein Smaug homolog 2	SAMD4B			
Q9NRG4	N-lysine methyltransferase	SMYD2			

Q9GZW5	Putative SCAN domain-containing protein SCAND2P	SCAND2P			
Q8NDV3	Structural maintenance of chromosomes protein 1B	SMC1B			
Q9UHJ3	Scm-like with four MBT domains protein 1	SFMBT1			
Q9H0E3	Histone deacetylase complex subunit SAP130	SAP130			
Q8N4H0	Spermatogenesis associated 6-like protein	SPATA6L			
P48764	Sodium/hydrogen exchanger 3	SLC9A3			
O43699	Sialic acid-binding Ig-like lectin 6	SIGLEC6			
Q8TEV9	Guanine nucleotide exchange protein SMCR8	SMCR8			
Q5VWJ9	Sorting nexin-30	SNX30			
A0A096LP01	Small integral membrane protein 26	SMIM26			
A7XYQ1	Sine oculis-binding protein homolog	SOBP			
O14543	Suppressor of cytokine signaling 3	SOCS3			
Q96FS4	Signal-induced proliferation-associated protein 1	SIPA1			
Q2MJR0	Sprouty-related, EVH1 domain-containing protein 3	SPRED3			
O75830	Serpin I2	SERPINI2			
Q8TB22	Spermatogenesis-associated protein 20	SPATA20			
A0A087WV53	SPEG neighbor protein	SPEGNB			
Q8NBJ9	SID1 transmembrane family member 2	SIDT2			
Q5TH74	O	STPG1			
Q9NRC1	Suppressor of tumorigenicity 7 protein	ST7			
Q8TDW4	Suppressor of tumorigenicity 7 protein-like	ST7L			
Q8WU08	Serine/threonine-protein kinase 32A	STK32A			
Q7Z7C7	Stimulated by retinoic acid gene 8 protein homolog	STRA8			
Q9BXU2	Testis-expressed protein 13B	TEX13B			
A7MD48	Serine/arginine repetitive matrix protein 4	SRRM4			
Q9UHV2	SERTA domain-containing protein 1	SERTAD1			
P49458	Signal recognition particle 9 kDa protein	SRP9			
P17987	T-complex protein 1 subunit	TCP1			
P0C7N4	Transmembrane protein 191B	TMEM191B			
Q9UJW2	Tubulointerstitial nephritis antigen	TINAG			
O43763	T-cell leukemia homeobox protein 2	TLX2			
Q9UP52	Transferrin receptor protein 2	TFR2			
P04155	Trefoil factor 1	TFF1			
A0A2R8Y7D0	Putative ubiquitin domain-containing protein TINCR	TINCR			
Q8TBM7	Transmembrane protein 254	TMEM254			
Q587J7	Putative ATP-dependent RNA helicase TDRD12	TDRD12			
P59922	Putative olfactory receptor 2B8	OR2B8P			
Q9Y3N9	Olfactory receptor 2W1	OR2W1			
Q8NGF7	Olfactory receptor 5B17	OR5B17			
Q15617	Olfactory receptor 8G1	OR8G1			
Q6UWY5	Olfactomedin-like protein 1	OLFML1			
Q8TE04	Pantothenate kinase 1	PANK1			
Q86YC2	Partner and localizer of BRCA2	PALB2			
Q8TEW8	Partitioning defective 3 homolog	PARD3B			
Q29RF7	Sister chromatid cohesion protein PDS5 homolog A	PDS5A			

Q15391	P2Y purinoceptor 14	P2RY14			
P09131	P3 protein	SLC10A3			
P15863	Paired box protein Pax-1	PAX1			
P49585	Choline-phosphate cytidylyltransferase A	PCYT1A			
P43119	Prostacyclin receptor	PTGIR			
Q9H2J4	Phosducin-like protein 3	PDCL3			
Q5JQD4	Putative peptide YY-3	PYY3			
P50876	E3 ubiquitin-protein ligase RNF144A	RNF144A			
O95199	RCC1 and BTB domain-containing protein 2	RCBTB2			
Q9BWF3	RNA-binding protein 4	RBM4			
P62491	Ras-related protein Rab-11A	RAB11A			
P62834	Ras-related protein Rap-1A	RAP1A			
P49792	E3 SUMO-protein ligase RanBP2	RANBP2			
P29762	Cellular retinoic acid-binding protein 1	CRABP1			
Q9P1G2	Putative uncharacterized protein encoded by RBM12B-AS1	RBM12B-AS1			
Q96EN9	Required for excision 1-B domain-containing protein	REX1BD			
O60216	Double-strand-break repair protein rad21 homolog	RAD21			
A7KAX9	Rho GTPase-activating protein 32	ARHGAP32			
Q9NX52	Rhomboid-related protein 2	RHBDL2			
Q6ZUB1	Spermatogenesis-associated protein 31E1	SPATA31E1			
Q5VYP0	Spermatogenesis-associated protein 31A3	SPATA31A3			
P82921	28S ribosomal protein S21, mitochondrial	MRPS21			
Q5M8T2	Solute carrier family 35 member D3	SLC35D3			
P82673	28S ribosomal protein S35, mitochondrial	MRPS35			
Q86VD7	Mitochondrial coenzyme A transporter SLC25A42	SLC25A42			
Q92736	Ryanodine receptor 2	RYR2			
P60903	Protein S100-A10	S100A10			
Q92504	Zinc transporter SLC39A7	SLC39A7			
Q9NP59	Solute carrier family 40 member 1	SLC40A1			
Q99584	Protein S100-A13	S100A13			
Q96JT2	Solute carrier family 45 member 3	SLC45A3			
P55011	Solute carrier family 12 member 2	SLC12A2			
Q9Y666	Solute carrier family 12 member 7	SLC12A7			
P78381	UDP-galactose translocator	SLC35A2			
Q9Y3D5	28S ribosomal protein S18c, mitochondrial	MRPS18C			
Q8NET4	Retrotransposon Gag-like protein 9	RTL9			
Q9UKG4	Solute carrier family 13 member 4	SLC13A4			
Q9HAB3	Solute carrier family 52, riboflavin transporter, member 2	SLC52A2			
Q2I0M5	R-spondin-4	RSPO4			
P62306	Small nuclear ribonucleoprotein F	SNRPF			
Q8N1H7	Protein SIX6OS1	SIX6OS1			
Q9UN76	Sodium- and chloride-dependent neutral and basic amino acid transporter B	SLC6A14			
Q16799	Reticulon-1	RTN1			
Q96NL0	RUN domain-containing protein 3B	RUNDC3B			
Q9BVN2	RUN and SH3 domain-containing protein 1	RUSC1			
Q96C34	RUN domain-containing protein 1	RUNDC1			
Q8N5C8	TGF-beta-activated kinase 1 and MAP3K7-binding protein 3	TAB3			

Q5TGI4	Sterile alpha motif domain-containing protein 5	SAMD5			
Q9NSI8	SAM domain-containing protein SAMSN-1	SAMSN1			
Q8WWX8	Sodium/myo-inositol cotransporter 2	SLC5A11			
P60468	Protein transport protein Sec61 subunit beta	SEC61B			
Q01826	DNA-binding protein SATB1	SATB1			
Q0VDG4	Secernin-3	SCRN3			
O15126	Secretory carrier-associated membrane protein 1	SCAMP1			
Q9NVA2	Septin-11	SEPTIN11			
Q9BWG6	Sodium channel modifier 1	SCNM1			
P0DPE8	Small integral membrane protein 34B	SMIM34B			
Q13884	Beta-1-syntrophin	SNTB1			
Q5SQN1	Synaptosomal-associated protein 47	SNAP47			
Q8WVK2	U4/U6.U5 small nuclear ribonucleoprotein 27 kDa protein	SNRNP27			
Q6UXR4	Putative serpin A13	SERPINA13P			
P30825	High affinity cationic amino acid transporter 1	SLC7A1			
A8MWL6	Putative synaptogyrin-2 like protein				
Q9NPB0	SAYSvFN domain-containing protein 1	SAYSD1			
Q96RM1	Small proline-rich protein 2F	SPRR2F			
Q2TAY7	WD40 repeat-containing protein SMU1	SMU1			
O94972	E3 ubiquitin-protein ligase	TRIM37			
O43597	Protein sprouty homolog 2	SPRY2			
Q9H9C1	Spermatogenesis-defective protein 39 homolog	VIPAS39			
Q14159	DNA repair-scaffolding protein	SPIDR			
Q9P270	SLAIN motif-containing protein 2	SLAIN2			
Q8IW03	Seven in absentia homolog 3	SIAH3			
Q96P15	Serpin B11	SERPINB11			
P21815	Bone sialoprotein 2	IBSP			
Q96RL6	Sialic acid-binding Ig-like lectin	SIGLEC11			
O43704	Sulfotransferase family cytosolic 1B member 1	SULT1B1			
Q93045	Stathmin-2	STMN2			
B8ZZ34	Protein shisa-8	SHISA8			
O94768	Serine/threonine-protein kinase 17B	STK17B			
Q8N412	Sperm-tail PG-rich repeat-containing protein 2	STPG2			
Q9UH99	SUN domain-containing protein 2	SUN2			
Q99469	SH3 and cysteine-rich domain-containing protein	STAC			
O75683	Surfeit locus protein 6	SURF6			
P0CW00	Testis-specific Y-encoded protein 8	TSPY8			
Q92537	Sushi domain-containing protein	SUSD6			
O75044	SLIT-ROBO Rho GTPase-activating protein 2	SRGAP2			
Q5VVX9	Ubiquitin-conjugating enzyme E2 U	UBE2U			
A6NNA2	Serine/arginine repetitive matrix protein 3	SRRM3			
B0FP48	Uroplakin-3b-like protein 1	UPK3BL1			
Q9UH65	Switch-associated protein 70	SWAP70			
Q8WUJ0	Serine/threonine/tyrosine-interacting protein	STYX			
P37288	Vasopressin V1a receptor	AVPR1A			
Q6SJ96	TATA box-binding protein-like 2	TBPL2			

P20062	Transcobalamin-2	TCN2			
Q86XR7	TIR domain-containing adapter molecule 2	TICAM2			
P0CG35	Thymosin beta-15B	TMSB15B			
Q9H853	Putative tubulin-like protein alpha-4B	TUBA4B			
Q9NQC1	E3 ubiquitin-protein ligase Jade-2	JADE2			
P05106	Integrin beta-3	ITGB3			
Q96HW7	Integrator complex subunit 4	INTS4			
C9JVW0	Putative transmembrane protein INAFM1	INAFM1			
O15225	Putative inactivation escape 1 protein	INE1			
Q1MX18	Protein inscuteable homolog	INSC			
O15503	Insulin-induced gene 1 protein	INSIG1			
Q96PR1	Potassium voltage-gated channel subfamily C member 2	KCNC2			
Q8NHM5	Lysine-specific demethylase 2B	KDM2B			
Q8N6M8	IQ domain-containing protein F1	IQCF1			
Q8NHK3	Killer cell immunoglobulin-like receptor 2DL5B	KIR2DL5B			
Q13601	KRR1 small subunit processome component homolog	KRR1			
Q6P2M8	Calcium/calmodulin-dependent protein kinase type 1B	PNCK			
Q5JQS5	Olfactory receptor 2B11	OR2B11			
P0DPK3	Notch homolog 2 N-terminal-like protein B	NOTCH2NL B			
Q8NGG6	Olfactory receptor 8B12	OR8B12			
Q9BXM7	Serine/threonine-protein kinase PINK1, mitochondrial	PINK1			
Q9H611	ATP-dependent DNA helicase	PIF1			
P14927	Cytochrome b-c1 complex subunit 7	UQCRB			
Q5H9M0	PWWP domain-containing DNA repair factor 3B	PWWP3B			
Q9H2L5	Ras association domain-containing protein 4	RASSF4			
P01111	GTPase NRas	NRAS			
Q7Z444	GTPase ERas	ERAS			
Q5TC82	Roquin-1	RC3H1			
P06400	Retinoblastoma-associated protein	RB1			
Q8IYK8	GTP-binding protein REM 2	REM2			
P98171	Rho GTPase-activating protein 4	ARHGAP4			
Q96QF0	Rab-3A-interacting protein	RAB3IP			
P0DJD0	RANBP2-like and GRIP domain-containing protein 1	RGPD1			
P63244	Receptor of activated protein C kinase 1	RACK1			
Q4ADV7	Guanine nucleotide exchange factor subunit RIC1	RIC1			
Q2M5E4	Regulator of G-protein signaling 21	RGS21			
F8VTS6	Ret finger protein-like 4A-like protein 1	RFPL4AL1			
Q8N431	Ras-GEF domain-containing family member 1C	RASGEF1C			
Q8TB24	Ras and Rab interactor 3	RIN3			
P62829	60S ribosomal protein L23	RPL23			
P61927	60S ribosomal protein L37	RPL37			
Q9BV68	E3 ubiquitin-protein ligase	RNF126			
O00584	Ribonuclease T2	RNASET2			
P60866	40S ribosomal protein S20	RPS20			
Q92681	Regulatory solute carrier protein family 1 member 1	RSC1A1			
O60687	Sushi repeat-containing protein SRPX2	SRPX2			

Q1RMZ1	S-adenosylmethionine sensor upstream of mTORC1	BMT2			
Q6UWV7	Protein shisa-like-2A	SHISAL2A			
O95968	Secretoglobin family 1D member	SCGB1D1			
O95025	Semaphorin-3D	SEMA3D			
Q9UJQ7	SCP2 sterol-binding domain-containing protein 1	SCP2D1			
Q9UPW6	DNA-binding protein SATB2	SATB2			
Q1EHB4	Sodium-coupled monocarboxylate transporter 2	SLC5A12			
A0A1B0GTR4	Putative small proline-rich protein 5	SPRR5			
O60613	Selenoprotein F	SELENOF			
O00767	Stearoyl-CoA desaturase	SCD			
Q2VWA4	SKI family transcriptional corepressor 2	SKOR2			
Q6IEE8	Schlafen family member 12-like	SLFN12L			
O95863	Zinc finger protein SNAI1	SNAI1			
Q9Y5W8	Sorting nexin-13	SNX13			
Q13342	Nuclear body protein SP140	SP140			
A6NFE2	Single-pass membrane and coiled-coil domain-containing protein 2	SMCO2			
Q15797	Mothers against decapentaplegic homolog 1	SMAD1			
O43761	Synaptogyrin-3	SYNGR3			
Q9H3E2	Sorting nexin-25	SNX25			
Q969W0	Serine palmitoyltransferase small subunit A	SPTSSA			
Q5STR5	Small integral membrane protein 40	SMIM40			
Q9BXG8	Spermatogenic leucine zipper protein 1	SPZ1			
Q2TAL5	Smoothelin-like protein 2	SMTNL2			
Q7Z7L1	Schlafen family member 11	SLFN11			
Q8N6T7	NAD-dependent protein deacetylase sirtuin-6	SIRT6			
Q92187	CMP-N-acetylneuraminate-poly-alpha-2,8-sialyltransferase	ST8SIA4			
Q56A73	Spindlin-4	SPIN4			
O60902	Short stature homeobox protein 2	SHOX2			
Q149N8	E3 ubiquitin-protein ligase SHPRH	SHPRH			
Q9NX95	Syntabulin	SYBU			
Q6UWI4	Protein shisa-2 homolog	SHISA2			
Q8TC36	SUN domain-containing protein 5	SUN5			
Q9NY15	Stabilin-1	STAB1			
O75558	Syntaxin-11	STX11			
Q08170	Serine/arginine-rich splicing factor 4	SRSF4			
Q66K14	TBC1 domain family member 9B	TBC1D9B			
A0A087WXS9	TBC1 domain family member 3I	TBC1D3I			
Q96DZ7	Transmembrane 4 L6 family member 19	TM4SF19			
A0A087X1G2	TBC1 domain family member 3K	TBC1D3K			
Q15813	Tubulin-specific chaperone E	TBCE			
Q5ST30	Valine--tRNA ligase, mitochondrial	VARS2			
Q7RTU1	Transcription factor 23	TCF23			
Q8NB59	Synaptotagmin-14	SYT14			
Q9NYW0	Taste receptor type 2 member 10	TAS2R10			
Q9H1K6	Talin rod domain-containing protein 1	TLNRD1			
P59536	Taste receptor type 2 member 41	TAS2R41			
Q9NWX6	Probable tRNA	THG1L			
Q9UKU6	Thyrotropin-releasing hormone-degrading ectoenzyme	TRHDE			
Q9BZY9	E3 ubiquitin-protein ligase	TRIM31			

Q6ZNR0	Transmembrane protein 91	TMEM91			
Q8IZ21	Phosphatase and actin regulator 4	PHACTR4			
Q9NXJ5	Pyroglutamyl-peptidase 1	PGPEP1			
Q9UGN5	Poly	PARP2			
O43189	PHD finger protein 1	PHF1			
Q9Y3A3	MOB-like protein phocein	MOB4			
Q9BWX1	PHD finger protein 7	PHF7			
P17858	ATP-dependent 6-phosphofructokinase, liver type	PFKL			
O43157	Plexin-B1	PLXNB1			
Q9NYY3	Serine/threonine-protein kinase PLK2	PLK2			
Q9BX97	Plasmalemma vesicle-associated protein	PLVAP			
Q12796	Proline-rich nuclear receptor coactivator 1	PNRC1			
P49593	Protein phosphatase 1F	PPM1F			
Q32ZL2	Phospholipid phosphatase-related protein type 5	PLPPR5			
P0C851	Phosphoinositide-interacting protein	PIRT			
O00625	Pirin	PIR			
Q3KR16	Pleckstrin homology domain-containing family G member 6	PLEKHG6			
Q9BZ71	Membrane-associated phosphatidylinositol transfer protein 3	PITPNM3			
Q6ZWE6	Pleckstrin homology domain-containing family M member 3	PLEKHM3			
P51817	cAMP-dependent protein kinase catalytic subunit PRKX	PRKX			
Q8TB68	Proline-rich protein 7	PRR7			
Q86Y79	Probable peptidyl-tRNA hydrolase	PTRH1			
Q3KNS1	Patched domain-containing protein 3	PTCHD3			
Q2TAL8	Glutamine-rich protein 1	QRICH1			
Q96HA9	Peroxisomal membrane protein 11C	PEX11G			
Q8TC12	Retinol dehydrogenase 11	RDH11			
Q7Z3Z2	Protein RD3	RD3			
A6NDE4	RNA-binding motif protein, Y chromosome, family 1 member B	RBMY1B			
Q86SE5	RNA-binding Raly-like protein	RALYL			
Q96DA2	Ras-related protein Rab-39B	RAB39B			
Q53S08	Ras-related protein Rab-6D	RAB6D			
Q9P2F6	Rho GTPase-activating protein 20	ARHGAP20			
Q9NRY4	Rho GTPase-activating protein 35	ARHGAP35			
Q99638	Cell cycle checkpoint control protein RAD9A	RAD9A			
Q96HR9	Receptor expression-enhancing protein 6	REEP6			
O15492	Regulator of G-protein signaling 16	RGS16			
O75678	Ret finger protein-like 2	RFPL2			
Q9H1J1	Regulator of nonsense transcripts 3A	UPF3A			
Q0VAM2	Ras-GEF domain-containing family member 1B	RASGEF1B			
Q96E11	Ribosome-recycling factor, mitochondrial	MRRF			
Q5JNZ5	Putative 40S ribosomal protein S26-like 1	RPS26P11			
Q5VWQ0	Lysine-specific demethylase 9	RSBN1			
Q92737	Ras-like protein family member 10A	RASL10A			
Q9P2J5	Leucine--tRNA ligase, cytoplasmic	LARS1			
P82914	28S ribosomal protein S15, mitochondrial	MRPS15			

Q9Y676	28S ribosomal protein S18b, mitochondrial	MRPS18B			
Q9UJQ4	Sal-like protein 4	SALL4			
P50443	Sulfate transporter	SLC26A2			
P82909	28S ribosomal protein S36, mitochondrial	MRPS36			
Q9BQC6	Ribosomal protein 63, mitochondrial	MRPL57			
Q9BXA9	Sal-like protein 3	SALL3			
Q8IVJ1	Solute carrier family 41 member 1	SLC41A1			
Q8TE82	SH3 domain and tetratricopeptide repeat-containing protein 1	SH3TC1			
Q15404	Ras suppressor protein 1	RSU1			
Q7L099	Protein RUFY3	RUFY3			
Q16586	Alpha-sarcoglycan	SGCA			
Q92629	Delta-sarcoglycan	SGCD			
P63302	Selenoprotein W	SELENOW			
Q9BQI5	SH3-containing GRB2-like protein 3-interacting protein 1	SGIP1			
Q5FBB7	Shugoshin 1	SGO1			
O00141	Serine/threonine-protein kinase Sgk1	SGK1			
P0DPB3	Schwannomin-interacting protein 1	SCHIP1			
Q96GD3	Polycomb protein SCMH1	SCMH1			
Q9BV35	Calcium-binding mitochondrial carrier protein SCaMC-3	SLC25A23			
Q9P0U3	Sentrin-specific protease 1	SENP1			
Q9BWW7	Transcriptional repressor scratch 1	SCRT1			
P23975	Sodium-dependent noradrenaline transporter	SLC6A2			
Q8WTV0	Scavenger receptor class B member 1	SCARB1			
Q8NB12	Histone-lysine N-methyltransferase SMYD1	SMYD1			
A8MWV9	Small integral membrane protein 34A	SMIM34A			
P54920	Alpha-soluble NSF attachment protein	NAPA			
Q496A3	Spermatogenesis-associated serine-rich protein 1	SPATS1			
A0A0B4J2F2	Probable serine/threonine-protein kinase SIK1B	SIK1B			
Q5TAH2	Sodium/hydrogen exchanger 11	SLC9C2			
Q8IX90	Spindle and kinetochore-associated protein 3	SKA3			
A0A286YFK9	Small integral membrane protein 38	SMIM38			
O43760	Synaptogyrin-2	SYNGR2			
Q96QK8	Small integral membrane protein 14	SMIM14			
Q15506	Sperm surface protein Sp17	SPA17			
P30626	Sorcin	SRI			
Q9H156	SLIT and NTRK-like protein 2	SLITRK2			
Q9NRY2	SOSS complex subunit C	INIP			
Q7Z698	Sprouty-related, EVH1 domain-containing protein 2	SPRED2			
P0CW21	Putative uncharacterized protein SPART-AS1	SPART-AS1			
A0A1B0GUW6	Uncharacterized protein SPEM3	SPEM3			
P61009	Signal peptidase complex subunit 3	SPCS3			
P00441	Superoxide dismutase	SOD1			
P53597	Succinate--CoA ligase	SUCLG1			
Q16514	Transcription initiation factor TFIID subunit 12	TAF12			
Q8NI27	THO complex subunit 2	THOC2			
Q9NT68	Teneurin-2	TENM2			

O15164	Transcription intermediary factor 1-alpha	TRIM24			
P22105	Tenascin-X	TNXB			
Q9Y5Q9	General transcription factor 3C polypeptide 3	GTF3C3			
Q96IP4	Terminal nucleotidyltransferase 5A	TENT5A			
Q5VZQ5	Testis-expressed protein 36	TEX36			
Q8IUE1	Homeobox protein TGIF2LX	TGIF2LX			
Q6ZWK6	Transmembrane protease serine 11F	TMPRSS11F			
Q86WS3	Oocyte-secreted protein 2	OOSP2			
P26367	Paired box protein Pax-6	PAX6			
P53611	Geranylgeranyl transferase type-2 subunit beta	RABGGTB			
P82980	Retinol-binding protein 5	RBP5			
P84098	60S ribosomal protein L19	RPL19			
P42766	60S ribosomal protein L35	RPL35			
Q9NYK5	39S ribosomal protein L39, mitochondrial	MRPL39			
Q92834	X-linked retinitis pigmentosa GTPase regulator	RPGR			
Q9Y3B9	RRP15-like protein	RRP15			
O94941	RING finger protein 37	UBOX5			
Q9NQG5	Regulation of nuclear pre-mRNA domain-containing protein 1B	RPRD1B			
Q5VZM2	Ras-related GTP-binding protein	RRAGB			
P62277	40S ribosomal protein S13	RPS13			
Q96S37	Solute carrier family 22 member 12	SLC22A12			
Q9BSK2	Solute carrier family 25 member 33	SLC25A33			
Q6PIS1	Solute carrier family 23 member 3	SLC23A3			
Q76EJ3	UDP-N-acetylglucosamine/UDP-glucose/GDP-mannose transporter	SLC35D2			
Q9Y2V3	Retinal homeobox protein Rx	RAX			
P0C7Q5	Putative solute carrier family 35 member G4	SLC35G4			
Q9Y2H2	Phosphatidylinositide phosphatase SAC2	INPP5F			
Q9Y2P5	Bile acyl-CoA synthetase	SLC27A5			
Q9C0K1	Metal cation symporter ZIP8	SLC39A8			
P05109	Protein S100-A8	S100A8			
Q8N1F8	Serine/threonine-protein kinase 11-interacting protein	STK11IP			
Q92503	SEC14-like protein 1	SEC14L1			
O15061	Synemin	SYNM			
Q4U2R8	Solute carrier family 22 member 6	SLC22A6			
Q99500	Sphingosine 1-phosphate receptor 3	S1PR3			
P82932	28S ribosomal protein S6, mitochondrial	MRPS6			
Q96BY9	Store-operated calcium entry-associated regulatory factor	SARAF			
Q9Y512	Sorting and assembly machinery component 50 homolog	SAMM50			
Q92622	Run domain Beclin-1-interacting and cysteine-rich domain-containing protein	RUBCN			
P07602	Prosaposin	PSAP			
O75556	Mammaglobin-B	SCGB2A1			
Q58EX2	Protein sidekick-2	SDK2			
Q96QR1	Secretoglobin family 3A member	SCGB3A1			
Q01105	Protein SET	SET			
Q9UGP8	Translocation protein SEC63 homolog	SEC63			
Q96HU1	Small G protein signaling modulator 3	SGSM3			

O60880	SH2 domain-containing protein 1A	SH2D1A			
Q6ZV89	SH2 domain-containing protein 5	SH2D5			
Q9NP31	SH2 domain-containing protein 2A	SH2D2A			
Q8N228	Sex comb on midleg-like protein	SCML4			
Q9UPX8	SH3 and multiple ankyrin repeat domains protein 2	SHANK2			
Q9Y566	SH3 and multiple ankyrin repeat domains protein 1	SHANK1			
Q7RTU7	Basic helix-loop-helix transcription factor scleraxis	SCX			
Q9Y2G9	Protein strawberry notch homolog 2	SBNO2			
P57086	SCAN domain-containing protein 1	SCAND1			
Q3SY56	Transcription factor Sp6	SP6			
O75971	snRNA-activating protein complex subunit 5	SNAPC5			
O95391	Pre-mRNA-splicing factor SLU7	SLU7			
Q8IY92	Structure-specific endonuclease subunit SLX4	SLX4			
O95473	Synaptogyrin-4	SYNGR4			
P0DKX4	Small integral membrane protein 18	SMIM18			
Q8N7X8	SIGLEC family-like protein 1	SIGLECL1			
Q96HG1	Small integral membrane protein 10	SMIM10			
Q86XE0	Sorting nexin-32	SNX32			
Q96PQ0	VPS10 domain-containing receptor SorCS2	SORCS2			
P0DTA3	Putative speedy protein E11	SPDYE11			
Q07890	Son of sevenless homolog 2	SOS2			
Q5VSR9	Sperm protein associated with the nucleus on the X chromosome N1	SPANXN1			
O95416	Transcription factor SOX-14	SOX14			
Q3KNW5	Solute carrier family 10 member 6	SLC10A6			
O75934	Pre-mRNA-splicing factor SPF27	BCAS2			
Q96N96	Spermatogenesis-associated protein 13	SPATA13			
Q9Y6A9	Signal peptidase complex subunit 1	SPCS1			
A0PJX4	Protein shisa-3 homolog	SHISA3			
O60224	Protein SSX4	SSX4			
P49842	Serine/threonine-protein kinase	STK19			
Q7RTN6	STE20-related kinase adapter protein alpha	STRADA			
P08842	Steryl-sulfatase	STS			
Q5JTZ9	Alanine--tRNA ligase, mitochondrial	AARS2			
Q96A49	Synapse-associated protein 1	SYAP1			
Q9BXP5	Serrate RNA effector molecule homolog	SRRT			
P59542	Taste receptor type 2 member 19	TAS2R19			
Q6IE38	Serine protease inhibitor Kazal-type 14	SPINK14			
Q01546	Keratin, type II cytoskeletal 2 oral	KRT76			
P16144	Integrin beta-4	ITGB4			
P01308	Insulin	INS			
Q9C075	Keratin, type I cytoskeletal 23	KRT23			
Q1ZYL8	Izumo sperm-egg fusion protein 4	IZUMO4			
Q9Y6K8	Adenylate kinase isoenzyme 5	AK5			
Q9UBS0	Ribosomal protein S6 kinase beta-2	RPS6KB2			
Q9P2J3	Kelch-like protein 9	KLHL9			
Q9H2R5	Kallikrein-15	KLK15			
Q9Y5W3	Krueppel-like factor 2	KLF2			
Q4G0X4	BTB/POZ domain-containing protein KCTD21	KCTD21			

P30613	Pyruvate kinase PKLR	PKLR			
Q07866	Kinesin light chain 1	KLC1			
P60412	Keratin-associated protein 10-11	KRTAP10-11			
A0A075B6S5	Immunoglobulin kappa variable 1-27	IGKV1-27			
Q15012	Lysosomal-associated transmembrane protein 4A	LAPTM4A			
Q68G74	LIM/homeobox protein Lhx8	LHX8			
A0A183	Late cornified envelope protein 6A	LCE6A			
O43561	Linker for activation of T-cells family member 1	LAT			
Q96AG4	Leucine-rich repeat-containing protein 59	LRRC59			
A4QPB2	Low-density lipoprotein receptor-related protein 5-like protein	LRP5L			
Q9Y2Y9	Krueppel-like factor 13	KLF13			
Q9Y664	KICSTOR complex protein kaptin	KPTN			
P18054	Polyunsaturated fatty acid lipoxygenase ALOX12	ALOX12			
Q9C099	Leucine-rich repeat and coiled-coil domain-containing protein 1	LRRCC1			
Q6XZB0	Lipase member I	LIPI			
A6NI73	Leukocyte immunoglobulin-like receptor subfamily A member 5	LILRA5			
Q86W92	Liprin-beta-1	PPFIBP1			
P06858	Lipoprotein lipase	LPL			
Q86UE4	Protein LYRIC	MTDH			
Q93074	Mediator of RNA polymerase II transcription subunit 12	MED12			
Q9H9K5	Endogenous retroviral envelope protein HEMO	ERVMER34-1			
Q96A32	Myosin regulatory light chain 2, skeletal muscle isoform	MYLPF			
Q8N6F8	Methyltransferase-like protein 27	METTL27			
Q13434	Putative E3 ubiquitin-protein ligase makorin-4	MKRN4P			
Q9BWT6	Meiotic nuclear division protein 1 homolog	MND1			
Q9UBM8	Alpha-1,3-mannosyl-glycoprotein 4-beta-N-acetylglucosaminyltransferase C	MGAT4C			
Q9H019	Mitochondrial fission regulator 1-like	MTFR1L			
Q86VD1	MORC family CW-type zinc finger protein 1	MORC1			
Q6NTE8	MRN complex-interacting protein	MRNIP			
Q13508	Ecto-ADP-ribosyltransferase 3	ART3			
Q92832	Protein kinase C-binding protein NELL1	NELL1			
O60524	Nuclear export mediator factor NEMF	NEMF			
Q5JS37	NHL repeat-containing protein 3	NHLRC3			
Q13563	Polycystin-2	PKD2			
Q14249	Endonuclease G, mitochondrial	ENDOG			
Q9BQG2	NAD-capped RNA hydrolase NUDT12	NUDT12			
Q9BZD4	Kinetochore protein Nuf2	NUF2			
Q8NGS0	Olfactory receptor 1N1	OR1N1			
P47874	Olfactory marker protein	OMP			
P47884	Olfactory receptor 1D4	OR1D4			
Q8NHA6	Putative olfactory receptor 2W6	OR2W6P			
P04000	Long-wave-sensitive opsin 1	OPN1LW			
Q5W0B1	ORC ubiquitin ligase 1	OBI1			
Q9UBM1	Phosphatidylethanolamine N-methyltransferase	PEMT			
P21810	Biglycan	BGN			

Q9NWQ8	Phosphoprotein associated with glycosphingolipid-enriched microdomains 1	PAG1			
Q969N2	GPI transamidase component PIG-T	PIGT			
P12955	Xaa-Pro dipeptidase	PEPD			
A0A1B0GUW7	Small integral membrane protein 27	SMIM27			
P22532	Small proline-rich protein 2D	SPRR2D			
Q9H6I2	Transcription factor SOX-17	SOX17			
P61647	Alpha-2,8-sialyltransferase 8F	ST8SIA6			
Q9NSC7	Alpha-N-acetylgalactosaminide alpha-2,6-sialyltransferase 1	ST6GALNAC1			
Q2M3G4	Protein Shroom1	SHROOM1			
Q8NBJ7	Inactive C-alpha-formylglycine-generating enzyme 2	SUMF2			
Q9BXU1	Serine/threonine-protein kinase	STK31			
P40763	Signal transducer and activator of transcription 3	STAT3			
Q9BYT3	Serine/threonine-protein kinase	STK33			
Q14849	StAR-related lipid transfer protein 3	STARD3			
Q6NZ63	STEAP family member 1B	STEAP1B			
A6NEL2	Ankyrin repeat domain-containing protein SOWAHB	SOWAHB			
Q96JX3	Protein SERAC1	SERAC1			
Q9C0H9	SRC kinase signaling inhibitor 1	SRCIN1			
Q9UL17	T-box transcription factor TBX21	TBX21			
Q9HCD6	Protein TANC2	TANC2			
Q01995	Transgelin	TAGLN			
O60603	Toll-like receptor 2	TLR2			
Q12800	Alpha-globin transcription factor CP2	TFCP2			
Q8NE00	Transmembrane protein 104	TMEM104			
P0DPH7	Tubulin alpha-3C chain	TUBA3C			
Q8N4L1	Transmembrane protein 151A	TMEM151A			
A0A1B0GTD5	Testis-expressed protein 49	TEX49			
Q9NPI0	Transmembrane protein 138	TMEM138			
Q8NCL8	Transmembrane protein 116	TMEM116			
P20366	Protachykinin-1	TAC1			
Q9Y490	Talin-1	TLN1			
Q8NHU6	Tudor domain-containing protein 7	TDRD7			
Q8NAT2	Tudor domain-containing protein 5	TDRD5			
Q9H330	Transmembrane protein 245	TMEM245			
Q5T292	Transmembrane protein 273	TMEM273			
P36897	TGF-beta receptor type-1	TGFBR1			
Q15583	Homeobox protein TGIF1	TGIF1			
Q9UPN9	E3 ubiquitin-protein ligase	TRIM33			
Q9UJV3	Probable E3 ubiquitin-protein ligase MID2	MID2			
Q9Y275	Tumor necrosis factor ligand superfamily member 13B	TNFSF13B			
B7Z8K6	T cell receptor delta constant	TRDC			
Q7Z2T5	TRMT1-like protein	TRMT1L			
Q8N9V7	Protein TOPAZ1	TOPAZ1			
Q8NGG2	Olfactory receptor 5T2	OR5T2			
Q9NRC9	Otoraplin	OTOR			
P0C604	Olfactory receptor 4A8	OR4A8			
O76100	Olfactory receptor 7A10	OR7A10			
Q96P11	28S rRNA	NSUN5			
Q8NGR3	Olfactory receptor 1K1	OR1K1			
Q99567	Nuclear pore complex protein Nup88	NUP88			
Q96RC9	Olfactory receptor 8B4	OR8B4			
Q5SRE5	Nucleoporin NUP188	NUP188			

Q6PK18	2-oxoglutarate and iron-dependent oxygenase domain-containing protein 3	OGFOD3			
P41146	Nociceptin receptor	OPRL1			
P42857	Neuronal vesicle trafficking-associated protein 1	NSG1			
P0CE71	Putative oncomodulin-2	OCM2			
O60404	Olfactory receptor 10H3	OR10H3			
Q6IF99	Olfactory receptor 10K2	OR10K2			
Q8NH04	Olfactory receptor 2T27	OR2T27			
P39656	Dolichyl-diphosphooligosaccharide--protein glycosyltransferase 48 kDa subunit	DDOST			
Q8NGF1	Olfactory receptor 52R1	OR52R1			
Q9H2C8	Olfactory receptor 51V1	OR51V1			
P30559	Oxytocin receptor	OXTR			
Q8NGZ2	Olfactory receptor 14K1	OR14K1			
Q15072	Zinc finger protein OZF	ZNF146			
Q96M63	Outer dynein arm-docking complex subunit 1	ODAD1			
Q8NGT2	Olfactory receptor 13J1	OR13J1			
P11182	Lipoamide acyltransferase component of branched-chain alpha-keto acid dehydrogenase complex, mitochondrial	DBT			
E9PI22	Proline-rich protein 23D1	PRR23D1			
Q92569	Phosphatidylinositol 3-kinase regulatory subunit gamma	PIK3R3			
Q8IZP6	RING finger protein 113B	RNF113B			
Q8NC44	Reticulophagy regulator 2	RETREG2			
Q07020	60S ribosomal protein L18	RPL18			
P08708	40S ribosomal protein S17	RPS17			
Q9HBX9	Relaxin receptor 1	RXFP1			
Q9Y6Y8	SEC23-interacting protein	SEC23IP			
Q969S0	UDP-xylose and UDP-N-acetylglucosamine transporter	SLC35B4			
P28702	Retinoic acid receptor RXR-beta	RXRB			
Q9BYN8	28S ribosomal protein S26, mitochondrial	MRPS26			
Q9H1U9	Mitochondrial nicotinamide adenine dinucleotide transporter SLC25A51	SLC25A51			
Q8WUX1	Sodium-coupled neutral amino acid transporter 5	SLC38A5			
O00442	RNA 3'-terminal phosphate cyclase	RTCA			
Q96GZ6	Solute carrier family 41 member 3	SLC41A3			
Q5BKX6	Solute carrier family 45 member 4	SLC45A4			
Q8NBS3	Sodium bicarbonate transporter-like protein 11	SLC4A11			
Q9NQ40	Solute carrier family 52, riboflavin transporter, member 3	SLC52A3			
Q9BZV2	Thiamine transporter 2	SLC19A3			
Q16348	Solute carrier family 15 member 2	SLC15A2			
Q9H7V2	Synapse differentiation-inducing gene protein 1	SYNDIG1			
O60779	Thiamine transporter 1	SLC19A2			
Q9H2J7	Sodium-dependent neutral amino acid transporter B	SLC6A15			
O43865	S-adenosylhomocysteine hydrolase-like protein 1	AHCYL1			
Q96CW6	Probable RNA polymerase II nuclear localization protein SLC7A6OS	SLC7A6OS			
O15244	Solute carrier family 22 member 2	SLC22A2			
Q9NQZ2	Something about silencing protein 10	UTP3			

Q9NV23	S-acyl fatty acid synthase thioesterase, medium chain	OLAH			
Q9BZR6	Reticulon-4 receptor	RTN4R			
Q7Z3H4	Sterile alpha motif domain-containing protein 7	SAMD7			
P08865	40S ribosomal protein SA	RPSA			
Q9Y6B6	GTP-binding protein SAR1b	SAR1B			
Q9Y6X0	SET-binding protein	SETBP1			
Q9H5K3	Protein O-mannose kinase	POMK			
O43819	Protein SCO2 homolog, mitochondrial	SCO2			
O43556	Epsilon-sarcoglycan	SGCE			
Q86TU7	Actin-histidine N-methyltransferase	SETD3			
Q15465	Sonic hedgehog protein	SHH			
Q8WXD2	Secretogranin-3	SCG3			
Q9NZV5	Selenoprotein N	SELENON			
Q6NUK1	Calcium-binding mitochondrial carrier protein SCaMC-1	SLC25A24			
Q6KCM7	Calcium-binding mitochondrial carrier protein SCaMC-2	SLC25A25			
Q9H0W7	THAP domain-containing protein 2	THAP2			
Q4G0G5	Secretoglobin family 2B member	SCGB2B2			
Q9GZV3	High affinity choline transporter 1	SLC5A7			
Q86VE3	Spermidine/spermine N	SATL1			
Q8IXZ3	Transcription factor Sp8	SP8			
O95295	SNARE-associated protein Snapin	SNAPIN			
Q9Y336	Sialic acid-binding Ig-like lectin 9	SIGLEC9			
O95475	Homeobox protein SIX6	SIX6			
Q96SB8	Structural maintenance of chromosomes protein 6	SMC6			
Q9H3U7	SPARC-related modular calcium-binding protein 2	SMOC2			
Q8NEM7	Transcription factor SPT20 homolog	SUPT20H			
Q8IUH8	Signal peptide peptidase-like 2C	SPPL2C			
Q9Y448	Small kinetochore-associated protein	KNSTRN			
O75563	Src kinase-associated phosphoprotein 2	SKAP2			
Q9Y5X3	Sorting nexin-5	SNX5			
Q96KF7	Small integral membrane protein	SMIM8			
Q9BQ15	SOSS complex subunit B1	NABP2			
Q5MJ08	Sperm protein associated with the nucleus on the X chromosome N4	SPANXN4			
Q7Z699	Sprouty-related, EVH1 domain-containing protein 1	SPRED1			
Q86VZ5	Phosphatidylcholine:ceramide cholinephosphotransferase 1	SGMS1			
P52788	Spermine synthase	SMS			
Q6RVD6	Spermatogenesis-associated protein 8	SPATA8			
Q7RTT5	Protein SSX7	SSX7			
Q9BZQ2	Testicular spindle-associated protein SHCBP1L	SHCBP1L			
Q15468	SCL-interrupting locus protein	STIL			
Q9NRP7	Serine/threonine-protein kinase	STK36			
P81877	Single-stranded DNA-binding protein 2	SSBP2			
Q9NQZ5	StAR-related lipid transfer protein 7, mitochondrial	STARD7			
Q8NEF9	Serum response factor-binding protein 1	SRFBP1			
Q9UNK0	Syntaxin-8	STX8			
Q1ZZU3	DNA repair protein SWI5 homolog	SWI5			
Q9HCS7	Pre-mRNA-splicing factor SYF1	XAB2			
Q96BW9	Phosphatidate cytidylyltransferase, mitochondrial	TAMM41			

P40200	T-cell surface protein tactile	CD96			
Q9BTW9	Tubulin-specific chaperone D	TBCD			
P56279	T-cell leukemia/lymphoma protein 1A	TCL1A			
Q8IXH6	Tumor protein p53-inducible nuclear protein 2	TP53INP2			
Q9BYX2	TBC1 domain family member 2A	TBC1D2			
P02786	Transferrin receptor protein 1	TFRC			
Q17RP2	Tigger transposable element-derived protein 6	TIGD6			
Q8WWR9	Pancreatic progenitor cell differentiation and proliferation factor-like protein	PPDPFL			
Q02548	Paired box protein Pax-5	PAX5			
P68402	Platelet-activating factor acetylhydrolase IB subunit alpha2	PAFAH1B2			
P78364	Polyhomeotic-like protein 1	PHC1			
Q9Y233	cAMP and cAMP-inhibited cGMP 3',5'-cyclic phosphodiesterase 10A	PDE10A			
Q06416	Putative POU domain, class 5, transcription factor 1B	POU5F1B			
Q2NL67	Protein mono-ADP-ribosyltransferase PARP6	PARP6			
Q13519	Prepronociceptin	PNOC			
P24666	Low molecular weight phosphotyrosine protein phosphatase	ACP1			
A0A0J9YX9	Paraneoplastic antigen Ma6F	PNMA6F			
Q15121	Astrocytic phosphoprotein PEA-	PEA15			
A8MTI9	Putative serine protease 47	PRSS47			
P22891	Vitamin K-dependent protein Z	PROZ			
O75400	Pre-mRNA-processing factor 40 homolog A	PRPF40A			
Q8NAV1	Pre-mRNA-splicing factor 38A	PRPF38A			
Q5VWM3	PRAME family member 18	PRAMEF18			
P50897	Palmitoyl-protein thioesterase 1	PPT1			
Q5SWL8	PRAME family member 19	PRAMEF19			
P26022	Pentraxin-related protein PTX3	PTX3			
Q16769	Glutaminyl-peptide cyclotransferase	QPCT			
Q99496	E3 ubiquitin-protein ligase RING2	RNF2			
Q86UR5	Regulating synaptic membrane exocytosis protein 1	RIMS1			
P62906	60S ribosomal protein L10a	RPL10A			
Q5XPI4	E3 ubiquitin-protein ligase	RNF123			
Q16540	39S ribosomal protein L23, mitochondrial	MRPL23			
Q96LZ7	Regulator of microtubule dynamics protein 2	RMDN2			
P35398	Nuclear receptor ROR-alpha	RORA			
P22626	Heterogeneous nuclear ribonucleoproteins A2/B1	HNRNPA2B1			
P19388	DNA-directed RNA polymerases I, II, and III subunit RPABC1	POLR2E			
P60153	Inactive ribonuclease-like protein 9	RNASE9			
Q9P0P0	E3 ubiquitin-protein ligase	RNF181			
Q14690	Protein RRP5 homolog	PDCD11			
Q5VT52	Regulation of nuclear pre-mRNA domain-containing protein 2	RPRD2			
P62861	40S ribosomal protein S30	FAU			
Q92552	28S ribosomal protein S27, mitochondrial	MRPS27			
Q96EL2	28S ribosomal protein S24, mitochondrial	MRPS24			
Q8IWB4	Spermatogenesis-associated protein 31A7	SPATA31A7			
Q6ICL7	Solute carrier family 35 member E4	SLC35E4			

Q9NSA0	Solute carrier family 22 member 11	SLC22A11			
Q9Y2Q9	28S ribosomal protein S28, mitochondrial	MRPS28			
Q9NP94	Zinc transporter ZIP2	SLC39A2			
Q6ZT89	Solute carrier family 25 member 48	SLC25A48			
P06703	Protein S100-A6	S100A6			
Q9UNT1	Rab-like protein 2B	RABL2B			
Q96NR8	Retinol dehydrogenase 12	RDH12			
Q9Y5S9	RNA-binding protein 8A	RBM8A			
P54725	UV excision repair protein RAD23 homolog A	RAD23A			
Q9H0T7	Ras-related protein Rab-17	RAB17			
P78563	Double-stranded RNA-specific editase 1	ADARB1			
Q969Q5	Ras-related protein Rab-24	RAB24			
P62820	Ras-related protein Rab-1A	RAB1A			
O75943	Cell cycle checkpoint protein RAD17	RAD17			
Q9C0H5	Rho GTPase-activating protein 39	ARHGAP39			
Q9BXF6	Rab11 family-interacting protein 5	RAB11FIP5			
O00287	Regulatory factor X-associated protein	RFXAP			
P49207	60S ribosomal protein L34	RPL34			
P62424	60S ribosomal protein L7a	RPL7A			
Q96EQ8	E3 ubiquitin-protein ligase	RNF125			
Q96PX1	E3 ubiquitin ligase RNF157	RNF157			
Q9NS64	Protein reprimo	RPRM			
P12755	Ski oncogene	SKI			
Q14524	Sodium channel protein type 5 subunit alpha	SCN5A			
Q8WVM8	Sec1 family domain-containing protein 1	SCFD1			
Q8TDD2	Transcription factor Sp7	SP7			
O95721	Synaptosomal-associated protein 29	SNAP29			
Q86WD7	Serpin A9	SERPINA9			
Q6IEG0	U11/U12 small nuclear ribonucleoprotein 48 kDa protein	SNRNP48			
Q9Y675	SNRPN upstream reading frame protein	SNURF			
Q9NPC8	Homeobox protein SIX2	SIX2			
O43759	Synaptogyrin-1	SYNGR1			
H3BMG3	Small lysine-rich protein 1	SMKR1			
O15020	Spectrin beta chain, non-erythrocytic 2	SPTBN2			
A6NIY4	Speedy protein E5	SPDYE5			
Q8NCC5	Sugar phosphate exchanger 3	SLC37A3			
Q9BT81	Transcription factor SOX-7	SOX7			
Q5JUK2	Spermatogenesis- and oogenesis-specific basic helix-loop-helix-containing protein 1	SOHLH1			
Q9BT56	Spexin	SPX			
O00267	Transcription elongation factor SPT5	SUPT5H			
Q96LK8	Spermatogenesis-associated protein 32	SPATA32			
Q11206	CMP-N-acetylneuraminate-beta-galactosamide-alpha-2,3-sialyltransferase 4	ST3GAL4			
P50226	Sulfotransferase 1A2	SULT1A2			
Q0VAF6	Syncollin	SYCN			
O43166	Signal-induced proliferation-associated 1-like protein 1	SIPA1L1			
P63165	Small ubiquitin-related modifier 1	SUMO1			
Q05952	Nuclear transition protein 2	TNP2			
Q9Y5Y6	Suppressor of tumorigenicity 14 protein	ST14			

P0CL84	Putative STAG3-like protein 2	STAG3L2			
Q04837	Single-stranded DNA-binding protein, mitochondrial	SSBP1			
O94804	Serine/threonine-protein kinase	STK10			
A6NJG2	Ankyrin repeat domain-containing protein SOWAHD	SOWAHD			
Q92844	TRAF family member-associated NF-kappa-B activator	TANK			
Q9NVR7	TBCC domain-containing protein	TBCCD1			
Q8TBP0	TBC1 domain family member 16	TBC1D16			
O60347	TBC1 domain family member 12	TBC1D12			
A0AVI4	E3 ubiquitin-protein ligase TM129	TMEM129			
P35625	Metalloproteinase inhibitor 3	TIMP3			
Q96DX7	Tripartite motif-containing protein 44	TRIM44			
Q9Y2L5	Trafficking protein particle complex subunit 8	TRAPPC8			
Q9Y2S6	Translation machinery-associated protein 7	TMA7			
Q7RTY8	Transmembrane protease serine 7	TMPRSS7			
P50616	Protein Tob1	TOB1			
Q9H4I3	TraB domain-containing protein	TRABD			
Q7Z404	Transmembrane channel-like protein 4	TMC4			
Q9BUB7	Transmembrane protein 70, mitochondrial	TMEM70			
Q86X19	Transmembrane protein 17	TMEM17			
Q9ULS5	Transmembrane and coiled-coil domain protein 3	TMCC3			
Q9Y210	Short transient receptor potential channel 6	TRPC6			
O14604	Thymosin beta-4, Y-chromosomal	TMSB4Y			
P63313	Thymosin beta-10	TMSB10			
Q9Y4R7	Tubulin monoglycylase TTLL3	TTLL3			
A6NH52	Golgi apparatus membrane protein TVP23 homolog A	TVP23A			
Q14679	Tubulin polyglutamylase TTLL4	TTLL4			
P61088	Ubiquitin-conjugating enzyme E2 N	UBE2N			
Q9UHF7	Zinc finger transcription factor Trps1	TRPS1			
Q6SA08	Testis-specific serine/threonine-protein kinase 4	TSSK4			
A0A0K0K1E9	T cell receptor beta variable 7-7	TRBV7-7			
A0AVT1	Ubiquitin-like modifier-activating enzyme 6	UBA6			
Q9Y5Z9	UbiA prenyltransferase domain-containing protein 1	UBIAD1			
Q53HI1	Protein unc-50 homolog	UNC50			
Q96RU2	Ubiquitin carboxyl-terminal hydrolase 28	USP28			
Q8IX04	Ubiquitin-conjugating enzyme E2 variant 3	UEVLD			
P17948	Vascular endothelial growth factor receptor 1	FLT1			
Q6UX71	Plexin domain-containing protein 2	PLXDC2			
P43351	DNA repair protein RAD52 homolog	RAD52			
Q7Z419	E3 ubiquitin-protein ligase RNF144B	RNF144B			
Q02833	Ras association domain-containing protein 7	RASSF7			
Q15109	Advanced glycosylation end product-specific receptor	AGER			
P78332	RNA-binding protein 6	RBM6			
Q6XE24	RNA-binding motif, single-stranded-interacting protein 3	RBMS3			

Q9UBG7	Recombining binding protein suppressor of hairless-like protein	RBPJL			
P98179	RNA-binding protein 3	RBM3			
Q99666	RANBP2-like and GRIP domain-containing protein 5/6	RGPD5			
Q86X27	Ras-specific guanine nucleotide-releasing factor RalGPS2	RALGPS2			
Q0D2K3	Protein ripply1	RIPPLY1			
O94844	Rho-related BTB domain-containing protein 1	RHOBTB1			
O43251	RNA binding protein fox-1 homolog 2	RBFOX2			
Q9BQY4	Rhox homeobox family member 2	RHOXF2			
Q8WYP3	Ras and Rab interactor 2	RIN2			
Q9H6W3	Ribosomal oxygenase 1	RIOX1			
Q4U2R6	39S ribosomal protein L51, mitochondrial	MRPL51			
Q8TEU7	Rap guanine nucleotide exchange factor 6	RAPGEF6			
Q8TA86	Retinitis pigmentosa 9 protein	RP9			
P04843	Dolichyl-diphosphooligosaccharide--protein glycosyltransferase subunit 1	RPN1			
Q9H0X6	RING finger protein 208	RNF208			
M0QZC1	RING finger protein 225	RNF225			
Q14108	Lysosome membrane protein 2	SCARB2			
O95104	SR-related and CTD-associated factor 4	SCAF4			
Q8WU79	Stromal membrane-associated protein 2	SMAP2			
Q9Y6J3	SMAD5 antisense gene protein 1	SMAD5-AS1			
P0CG40	Transcription factor Sp9	SP9			
Q8ND04	Protein SMG8	SMG8			
Q86UD5	Sodium/hydrogen exchanger 9B2	SLC9B2			
Q13596	Sorting nexin-1	SNX1			
Q96H20	Vacuolar-sorting protein SNF8	SNF8			
Q86WV1	Src kinase-associated phosphoprotein 1	SKAP1			
Q9UM82	Spermatogenesis-associated protein 2	SPATA2			
Q9H254	Spectrin beta chain, non-erythrocytic 4	SPTBN4			
Q8WVK7	Spindle and kinetochore-associated protein 2	SKA2			
Q6IA17	Single Ig IL-1-related receptor	SIGIRR			
A6NLE4	Small integral membrane protein 23	SMIM23			
G3V0H7	Putative solute carrier organic anion transporter family member 1B7	SLCO1B7			
O75264	Small integral membrane protein 24	SMIM24			
Q92925	SWI/SNF-related matrix-associated actin-dependent regulator of chromatin subfamily D member 2	SMARCD2			
Q96I25	Splicing factor 45	RBM17			
Q16384	Protein SSX1	SSX1			
P42229	Signal transducer and activator of transcription 5A	STAT5A			
Q13188	Serine/threonine-protein kinase 3	STK3			
O94901	SUN domain-containing protein 1	SUN1			
Q9ULZ2	Signal-transducing adaptor protein 1	STAP1			
Q6NXT6	Transmembrane anterior posterior transformation protein 1 homolog	TAPT1			
Q69YZ2	Transmembrane protein 200B	TMEM200B			

Q9NYW2	Taste receptor type 2 member 8	TAS2R8			
Q99576	TSC22 domain family protein 3	TSC22D3			
Q8N2H4	Protein SYS1 homolog	SYS1			
P15923	Transcription factor E2-alpha	TCF3			
Q86TG1	Transmembrane protein 150A	TMEM150A			
Q9BQ16	Testican-3	SPOCK3			
Q96I45	Transmembrane protein 141	TMEM141			
Q9BTD3	Transmembrane protein 121	TMEM121			
O14746	Telomerase reverse transcriptase	TERT			
P05452	Tetranectin	CLEC3B			
Q7Z2Z1	Treslin	TICRR			
A0A0J9YWX3	T cell receptor beta joining 1-6	TRBJ1-6			
Q9BXT5	Testis-expressed protein 15	TEX15			
A0A0A0MT94	T cell receptor beta joining 2-2	TRBJ2-2			
A0A0A0MT78	T cell receptor beta joining 2-7	TRBJ2-7			
Q8N6I4	Transmembrane protein 251	TMEM251			
Q86SX3	Tubulin epsilon and delta complex protein 1	TEDC1			
Q9Y2W6	Tudor and KH domain-containing protein	TDRKH			
Q9BX74	TM2 domain-containing protein 1	TM2D1			
Q96SK2	Transmembrane protein 209	TMEM209			
P28347	Transcriptional enhancer factor TEF-1	TEAD1			
Q14656	Transmembrane protein 187	TMEM187			
Q7L0Y3	tRNA methyltransferase 10 homolog C	TRMT10C			
P01137	Transforming growth factor beta-1 proprotein	TGFB1			
Q9C035	Tripartite motif-containing protein 5	TRIM5			
P20396	Pro-thyrotropin-releasing hormone	TRH			
Q6ZTA4	Tripartite motif-containing protein 67	TRIM67			
O14656	Torsin-1A	TOR1A			
Q495X7	Tripartite motif-containing protein 60	TRIM60			
A0A577	T cell receptor beta variable 4-1	TRBV4-1			
A0A0B4J24	T cell receptor alpha variable 12-1	TRAV12-1			
A0A087WSZ9	T cell receptor alpha variable 30	TRAV30			
O95801	Tetratricopeptide repeat protein 4	TTC4			
A0A087X0K7	Probable non-functional T cell receptor beta variable 17	TRBV17			
Q712K3	Ubiquitin-conjugating enzyme E2 R2	UBE2R2			
P68036	Ubiquitin-conjugating enzyme E2 L3	UBE2L3			
Q96HA8	Protein N-terminal glutamine amidohydrolase	NTAQ1			
Q8NGA1	Olfactory receptor 1M1	OR1M1			
Q8TBF5	Phosphatidylinositol-glycan biosynthesis class X protein	PIGX			
Q5H8A4	GPI ethanolamine phosphate transferase 2	PIGG			
Q86TB9	Protein PAT1 homolog 1	PATL1			
Q99497	Parkinson disease protein 7	PARK7			
O15055	Period circadian protein homolog 2	PER2			
Q6NSJ2	Pleckstrin homology-like domain family B member 3	PHLDB3			
P18669	Phosphoglycerate mutase 1	PGAM1			
Q9UIW2	Plexin-A1	PLXNA1			
P62937	Peptidyl-prolyl cis-trans isomerase A	PPIA			

Q13356	RING-type E3 ubiquitin-protein ligase PPIL2	PPIL2			
Q99541	Perilipin-2	PLIN2			
Q99959	Plakophilin-2	PKP2			
Q6UW63	Protein O-glucosyltransferase 2	POGLUT2			
Q9NRY6	Phospholipid scramblase 3	PLSCR3			
Q8N131	Porimin	TMEM123			
P62714	Serine/threonine-protein phosphatase 2A catalytic subunit beta isoform	PPP2CB			
Q6P1J6	Phospholipase B1, membrane-associated	PLB1			
Q9HB19	Pleckstrin homology domain-containing family A member 2	PLEKHA2			
Q9HB20	Pleckstrin homology domain-containing family A member 3	PLEKHA3			
Q96JA3	Pleckstrin homology domain-containing family A member 8	PLEKHA8			
Q9BZ72	Membrane-associated phosphatidylinositol transfer protein 2	PITPNM2			
Q99935	Opiorphin prepropeptide	OPRPN			
P28062	Proteasome subunit beta type-8	PSMB8			
P0C7W0	Proline-rich protein 29	PRR29			
O60831	PRA1 family protein 2	PRAF2			
O95685	Protein phosphatase 1 regulatory subunit 3D	PPP1R3D			
Q04118	Basic salivary proline-rich protein	PRB3			
P30041	Peroxiredoxin-6	PRDX6			
P30048	Thioredoxin-dependent peroxide reductase, mitochondrial	PRDX3			
P07737	Profilin-1	PFN1			
P41222	Prostaglandin-H2 D-isomerase	PTGDS			
O14522	Receptor-type tyrosine-protein phosphatase T	PTPRT			
Q9BT73	Proteasome assembly chaperone 3	PSMG3			
P55036	26S proteasome non-ATPase regulatory subunit 4	PSMD4			
Q15397	Pumilio homolog 3	PUM3			
B2RC85	Radial spoke head 10 homolog B2	RSPH10B2			
P62851	40S ribosomal protein S25	RPS25			
Q9BUD6	Spondin-2	SPON2			
P0DMW5	Small integral membrane protein 10-like protein 2B	SMIM10L2B			
A2RU48	Single-pass membrane and coiled-coil domain-containing protein 3	SMCO3			
Q9NPD5	Solute carrier organic anion transporter family member 1B3	SLCO1B3			
H3BR10	Small leucine-rich protein 1	SMLR1			
Q13573	SNW domain-containing protein	SNW1			
A0A5F9ZH02	Small integral membrane protein 42	SMIM42			
P0DUD2	Putative speedy protein E17	SPDYE17			
Q96P63	Serpin B12	SERPINB12			
Q9HCE7	E3 ubiquitin-protein ligase SMURF1	SMURF1			
P17947	Transcription factor PU.1	SPI1			
P48436	Transcription factor SOX-9	SOX9			
Q8NHX4	Spermatogenesis-associated protein 3	SPATA3			
Q8N7U7	Tetra-peptide repeat homeobox protein 1	TPRX1			
P0DMM9	Sulfotransferase 1A3	SULT1A3			
Q13043	Serine/threonine-protein kinase 4	STK4			
Q9P2P6	StAR-related lipid transfer protein 9	STARD9			
A0A0B4J27	T cell receptor alpha variable 12-3	TRAV12-3			

Q8N300	Small vasohibin-binding protein	SVBP			
P07814	Bifunctional glutamate/proline--tRNA ligase	EPRS1			
Q9Y4P3	Transducin beta-like protein 2	TBL2			
Q9HAU6	Putative translationally-controlled tumor protein-like protein TPT1P8	TPT1P8			
P48230	Transmembrane 4 L6 family member 4	TM4SF4			
Q9Y242	Transcription factor 19	TCF19			
Q8N9U0	Tandem C2 domains nuclear protein	TC2N			
Q9BSH4	Translational activator of cytochrome c oxidase 1	TACO1			
Q03518	Antigen peptide transporter 1	TAP1			
P52657	Transcription initiation factor IIA subunit 2	GTF2A2			
Q9BSW7	Synaptotagmin-17	SYT17			
Q14186	Transcription factor Dp-1	TFDP1			
O43151	Methylcytosine dioxygenase TET3	TET3			
A0A1B0GU33	Testis-expressed protein 53	TEX53			
O95411	TGFB1-induced anti-apoptotic factor 1	TIAF1			
Q15569	Dual specificity testis-specific protein kinase 1	TESK1			
A0A0J9YXM7	T cell receptor beta joining 1-5	TRBJ1-5			
Q08629	Testican-1	SPOCK1			
Q9BXU0	Testis-expressed protein 12	TEX12			
Q86UU9	Tachykinin-4	TAC4			
A0A0A0MTA7	T cell receptor beta joining 2-1	TRBJ2-1			
Q9BU70	tRNA	TRMO			
I1YAP6	Tripartite motif-containing protein 77	TRIM77			
Q6NT89	TMF-regulated nuclear protein 1	TRNP1			
Q629K1	Triple QxxK/R motif-containing protein	TRIQK			
O14717	tRNA	TRDMT1			
Q63HR2	Tensin-2	TNS2			
Q9NYL9	Tropomodulin-3	TMOD3			
Q12888	TP53-binding protein 1	TP53BP1			
P19075	Tetraspanin-8	TSPAN8			
B1AH88	Putative peripheral benzodiazepine receptor-related protein	TSPO			
P35443	Thrombospondin-4	THBS4			
O75694	Nuclear pore complex protein Nup155	NUP155			
Q7Z3S9	Notch homolog 2 N-terminal-like protein A	NOTCH2NLA			
Q8NGY2	Olfactory receptor 6K2	OR6K2			
O95222	Olfactory receptor 6A2	OR6A2			
Q7Z3T1	Olfactory receptor 2W3	OR2W3			
Q5UE93	Phosphoinositide 3-kinase regulatory subunit 6	PIK3R6			
Q9Y5Y5	Peroxisomal membrane protein PEX16	PEX16			
Q8N2H9	E3 ubiquitin-protein ligase pellino homolog 3	PELI3			
Q6PEW0	Inactive serine protease 54	PRSS54			
Q5FWE3	Proline-rich transmembrane protein 3	PRRT3			
Q5SWA1	Protein phosphatase 1 regulatory subunit 15B	PPP1R15B			
Q00888	Pregnancy-specific beta-1-glycoprotein 4	PSG4			
P11465	Pregnancy-specific beta-1-glycoprotein 2	PSG2			

Q92932	Receptor-type tyrosine-protein phosphatase N2	PTPRN2			
P23396	40S ribosomal protein S3	RPS3			
P62701	40S ribosomal protein S4, X isoform	RPS4X			
Q96DW6	Mitochondrial glycine transporter	SLC25A38			
Q6Q0C1	Solute carrier family 25 member 47	SLC25A47			
Q96NU1	Sterile alpha motif domain-containing protein 11	SAMD11			
Q8IZD0	Sterile alpha motif domain-containing protein 14	SAMD14			
P35542	Serum amyloid A-4 protein	SAA4			
Q86WA9	Sodium-independent sulfate anion transporter	SLC26A11			
Q8WXG8	Protein S100-Z	S100Z			
Q5H9E4	Solute carrier family 25 member 53	SLC25A53			
A6NNN8	Putative sodium-coupled neutral amino acid transporter 8	SLC38A8			
O43868	Sodium/nucleoside cotransporter 2	SLC28A2			
P29034	Protein S100-A2	S100A2			
Q9UMX9	Membrane-associated transporter protein	SLC45A2			
Q9Y3I0	RNA-splicing ligase RtcB homolog	RTCB			
Q6U841	Sodium-driven chloride bicarbonate exchanger	SLC4A10			
Q9NZ71	Regulator of telomere elongation helicase 1	RTEL1			
Q9C0A6	Histone-lysine N-methyltransferase SETD5	SETD5			
Q9NVD3	SET domain-containing protein 4	SETD4			
O14796	SH2 domain-containing protein	SH2D1B			
Q9HB31	Homeobox protein SEBOX	SEBOX			
Q7Z4S9	SH2 domain-containing protein 6	SH2D6			
P18827	Syndecan-1	SDC1			
Q9NVU7	Protein SDA1 homolog	SDAD1			
P09683	Secretin	SCT			
O15047	Histone-lysine N-methyltransferase SETD1A	SETD1A			
P31431	Syndecan-4	SDC4			
Q9H4I8	Serine hydrolase-like protein 2	SERHL2			
O75326	Semaphorin-7A	SEMA7A			
Q8NFY4	Semaphorin-6D	SEMA6D			
Q9H3T3	Semaphorin-6B	SEMA6B			
O43175	D-3-phosphoglycerate dehydrogenase	PHGDH			
Q6ZVN7	Putative protein SEM1, isoform 2	SEM1			
Q9NRX5	Serine incorporator 1	SERINC1			
Q9H4L4	Sentrin-specific protease 3	SENP3			
Q99884	Sodium-dependent proline transporter	SLC6A7			
Q9Y289	Sodium-dependent multivitamin transporter	SLC5A6			
Q96HI0	Sentrin-specific protease 5	SENP5			
Q96SA4	Serine incorporator 2	SERINC2			
Q9UI33	Sodium channel protein type 11 subunit alpha	SCN11A			
Q9Y5K1	Meiotic recombination protein SPO11	SPO11			
Q96EX1	Small integral membrane protein 12	SMIM12			
Q969T3	Sorting nexin-21	SNX21			
Q4ZJI4	Sodium/hydrogen exchanger 9B1	SLC9B1			
Q8TEV8	Smith-Magenis syndrome chromosomal region candidate gene 5 protein	SMCR5			

A0A1B0GW54	Small integral membrane protein 39	SMIM39			
Q9UBP0	Spastin	SPAST			
P46721	Solute carrier organic anion transporter family member 1A2	SLCO1A2			
Q02446	Transcription factor Sp4	SP4			
A0A494C0Z2	Putative speedy protein E13	SPDYE13			
Q92673	Sortilin-related receptor	SORL1			
O00570	Transcription factor SOX-1	SOX1			
Q14493	Histone RNA hairpin-binding protein	SLBP			
O15466	Alpha-2,8-sialyltransferase 8E	ST8SIA5			
Q9NZQ3	NCK-interacting protein with SH3 domain	NCKIPSD			
Q8IWU6	Extracellular sulfatase Sulf-1	SULF1			
Q86UX6	Serine/threonine-protein kinase 32C	STK32C			
Q9Y274	Type 2 lactosamine alpha-2,3-sialyltransferase	ST3GAL6			
E0CX11	Short transmembrane mitochondrial protein 1	STMP1			
P09430	Spermatid nuclear transition protein 1	TNP1			
Q8IUC6	TIR domain-containing adapter molecule 1	TICAM1			
P49848	Transcription initiation factor TFIID subunit 6	TAF6			
Q9BT92	Trichoplein keratin filament-binding protein	TCHP			
Q8WWU5	T-complex protein 11 homolog	TCP11			
Q9UKN8	General transcription factor 3C polypeptide 4	GTF3C4			
Q5TEJ8	Protein THEMIS2	THEMIS2			
Q6PF06	tRNA methyltransferase 10 homolog B	TRMT10B			
P58753	Toll/interleukin-1 receptor domain-containing adapter protein	TIRAP			
Q6ZNM6	Testis-expressed protein 43	TEX43			
Q5T1C6	Acyl-coenzyme A thioesterase THEM4	THEM4			
Q12789	General transcription factor 3C polypeptide 1	GTF3C1			
P47974	mRNA decay activator protein ZFP36L2	ZFP36L2			
Q3Y452	Testis development-related protein 1	TDRG1			
A6NGB7	Transmembrane protein 221	TMEM221			
Q6NUQ4	Transmembrane protein 214	TMEM214			
Q14142	Tripartite motif-containing protein 14	TRIM14			
Q12899	Tripartite motif-containing protein 26	TRIM26			
Q9BZR9	E3 ubiquitin-protein ligase TRIM8	TRIM8			
Q96PN7	Transcriptional-regulating factor 1	TRERF1			
A6NGJ6	Tripartite motif-containing protein 64	TRIM64			
Q8NDV7	Trinucleotide repeat-containing gene 6A protein	TNRC6A			
Q8N584	Tetratricopeptide repeat protein 39C	TTC39C			
Q6ZMV5	Protein PPP4R3C	PPP4R3C			
Q96C36	Pyrroline-5-carboxylate reductase 2	PYCR2			
Q86T03	Type 1 phosphatidylinositol 4,5-bisphosphate 4-phosphatase	PIP4P1			
P00491	Purine nucleoside phosphorylase	PNP			

O75170	Serine/threonine-protein phosphatase 6 regulatory subunit 2	PPP6R2			
Q9NRY7	Phospholipid scramblase 2	PLSCR2			
Q9H8P0	Polyprenol reductase	SRD5A3			
Q9BZL4	Protein phosphatase 1 regulatory subunit 12C	PPP1R12C			
A1L390	Pleckstrin homology domain-containing family G member 3	PLEKHG3			
Q96S79	Ras-like protein family member 10B	RASL10B			
P22090	40S ribosomal protein S4, Y isoform 1	RPS4Y1			
Q96G79	Probable UDP-sugar transporter protein SLC35A4	SLC35A4			
Q8TBE7	Solute carrier family 35 member G2	SLC35G2			
Q96BI1	Solute carrier family 22 member 18	SLC22A18			
P17900	Ganglioside GM2 activator	GM2A			
P55809	Succinyl-CoA:3-ketoacid coenzyme A transferase 1, mitochondrial	OXCT1			
Q8IWX5	Sphingosine-1-phosphate phosphatase 2	SGPP2			
P59797	Selenoprotein V	SELENOV			
A6NJ88	Putative SAGE1-like protein	SAGE2P			
Q15436	Protein transport protein Sec23A	SEC23A			
Q86SQ7	Serologically defined colon cancer antigen 8	SDCCAG8			
O94855	Protein transport protein Sec24D	SEC24D			
P13866	Sodium/glucose cotransporter 1	SLC5A1			
Q9NQ03	Transcriptional repressor scratch 2	SCRT2			
Q9NQ36	Signal peptide, CUB and EGF-like domain-containing protein 2	SCUBE2			
Q8IVN8	Somatomedin-B and thrombospondin type-1 domain-containing protein	SBSPON			
Q9BSF0	Small membrane A-kinase anchor protein	C2orf88			
Q9Y343	Sorting nexin-24	SNX24			
P51531	Probable global transcription activator SNF2L2	SMARCA2			
O43791	Speckle-type POZ protein	SPOP			
Q8NEY3	Spermatogenesis-associated protein 4	SPATA4			
P81133	Single-minded homolog 1	SIM1			
Q7Z3B0	Small integral membrane protein 15	SMIM15			
Q9UNH6	Sorting nexin-7	SNX7			
Q9NRA0	Sphingosine kinase 2	SPHK2			
Q8IYM2	Schlafen family member 12	SLFN12			
Q6UDR6	Kunitz-type protease inhibitor 4	SPINT4			
O43278	Kunitz-type protease inhibitor 1	SPINT1			
Q8N5J4	Transcription factor Spi-C	SPIC			
Q9NXL6	SID1 transmembrane family member 1	SIDT1			
Q16842	CMP-N-acetylneuraminate-beta-galactosamide-alpha-2,3-sialyltransferase 2	ST3GAL2			
Q9BSE5	Agmatinase, mitochondrial	AGMAT			
Q9NSN8	Gamma-1-syntrophin	SNTG1			
Q6EEV6	Small ubiquitin-related modifier 4	SUMO4			
O75486	Transcription initiation protein SPT3 homolog	SUPT3H			
A2RUT3	Transmembrane protein 89	TMEM89			
Q8N2I9	Serine/threonine-protein kinase	STK40			
Q8N3U4	Cohesin subunit SA-2	STAG2			

O95210	Starch-binding domain-containing protein 1	STBD1			
Q8WVM7	Cohesin subunit SA-1	STAG1			
P51649	Succinate-semialdehyde dehydrogenase, mitochondrial	ALDH5A1			
Q9Y2K9	Syntaxin-binding protein 5-like	STXBP5L			
A6NGB0	Transmembrane protein 191C	TMEM191C			
Q15572	TATA box-binding protein-associated factor RNA polymerase I subunit C	TAF1C			
Q9UGM6	Tryptophan--tRNA ligase, mitochondrial	WARS2			
P40227	T-complex protein 1 subunit zeta	CCT6A			
Q7Z5A7	Chemokine-like protein TAFA-5	TAFA5			
Q9NVA4	Transmembrane protein 184C	TMEM184C			
Q5BJH2	Transmembrane protein 128	TMEM128			
Q5T7P8	Synaptotagmin-6	SYT6			
P0DPH8	Tubulin alpha-3D chain	TUBA3D			
Q96M34	Testis-specific expressed protein 55	TEX55			
Q01085	Nucleolysin TIAR	TIAL1			
P10827	Thyroid hormone receptor alpha	THRA			
Q71RH2	Ceramide synthase	TLCD3B			
Q8IWB9	Testis-expressed protein 2	TEX2			
O43897	Tolloid-like protein 1	TLL1			
A0PJW6	Transmembrane protein 223	TMEM223			
Q6ICI0	Transmembrane protein 211	TMEM211			
Q9H1E5	Thioredoxin-related transmembrane protein 4	TMX4			
Q9C029	E3 ubiquitin-protein ligase TRIM7	TRIM7			
Q9NRS4	Transmembrane protease serine 4	TMPRSS4			
O00220	Tumor necrosis factor receptor superfamily member 10A	TNFRSF10A			
Q15633	RISC-loading complex subunit TARBP2	TARBP2			
A0A075B6T6	T cell receptor alpha variable 12-2	TRAV12-2			
A0A075B6T7	T cell receptor alpha variable 6	TRAV6			
A0A0J9YXY	T cell receptor beta variable 6-2	TRBV6-2			
O75841	Uroplakin-1b	UPK1B			
Q86UP9	LHFPL tetraspan subfamily member 3 protein	LHFPL3			
P01601	Immunoglobulin kappa variable 1D-16	IGKV1D-16			
Q9H1Y3	Opsin-3	OPN3			
P30953	Olfactory receptor 1E1	OR1E1			
Q8NGR5	Olfactory receptor 1L4	OR1L4			
Q8NE18	Putative methyltransferase NSUN7	NSUN7			
Q8NGP2	Olfactory receptor 8J1	OR8J1			
Q8NGT5	Olfactory receptor 9A2	OR9A2			
Q8NH01	Olfactory receptor 2T11	OR2T11			
Q9H207	Olfactory receptor 10A5	OR10A5			
Q6IFN5	Olfactory receptor 7E24	OR7E24			
Q93086	P2X purinoceptor 5	P2RX5			
Q8NGJ2	Olfactory receptor 52H1	OR52H1			
A6NDL8	Olfactory receptor 6C68	OR6C68			
Q8NGS6	Olfactory receptor 13C3	OR13C3			
Q8NGI1	Putative olfactory receptor 56B2	OR56B2P			
Q17RF5	Odontogenesis associated phosphoprotein	ODAPH			
Q9BXB5	Oxysterol-binding protein-related protein 10	OSBPL10			
Q9BZF1	Oxysterol-binding protein-related protein 8	OSBPL8			
Q96R54	Olfactory receptor 14A2	OR14A2			
Q9BQT8	Mitochondrial 2-oxodicarboxylate carrier	SLC25A21			
A0A2R8Y4Y8	Oocyte-secreted protein 4B	OOSP4B			

Q9H158	Protocadherin alpha-C1	PCDHAC1			
Q9Y2B2	N-acetylglucosaminyl-phosphatidylinositol de-N-acetylase	PIGL			
Q92968	Peroxisomal membrane protein PEX13	PEX13			
P43116	Prostaglandin E2 receptor EP2 subtype	PTGER2			
Q8NEN9	PDZ domain-containing protein 8	PDZD8			
Q8N8W4	Omega-hydroxyceramide transacylase	PNPLA1			
P62333	26S proteasome regulatory subunit 10B	PSMC6			
Q16378	Proline-rich protein 4	PRR4			
P23942	Peripherin-2	PRPH2			
O43395	U4/U6 small nuclear ribonucleoprotein Prp3	PRPF3			
O75864	Protein phosphatase 1 regulatory subunit 37	PPP1R37			
Q53SZ7	Proline-rich protein 30	PRR30			
Q99952	Tyrosine-protein phosphatase non-receptor type 18	PTPN18			
Q09MP3	RAD51-associated protein 2	RAD51AP2			
P05451	Lithostathine-1-alpha	REG1A			
P51151	Ras-related protein Rab-9A	RAB9A			
Q8WUD1	Ras-related protein Rab-2B	RAB2B			
Q9NTZ6	RNA-binding protein 12	RBM12			
P49798	Regulator of G-protein signaling 4	RGS4			
P18577	Blood group Rh	RHCE			
Q8HWS3	DNA-binding protein RFX6	RFX6			
O94810	Regulator of G-protein signaling 11	RGS11			
O14593	DNA-binding protein RFXANK	RFXANK			
Q9H9A7	RecQ-mediated genome instability protein 1	RMI1			
P02814	Submaxillary gland androgen-regulated protein 3B	SMR3B			
Q8IVW8	Protein spinster homolog 2	SPNS2			
Q9Y3P8	Signaling threshold-regulating transmembrane adapter 1	SIT1			
Q8N0Z3	Spindle and centriole-associated protein 1	SPICE1			
Q96N06	Spermatogenesis-associated protein 33	SPATA33			
Q9NR45	Sialic acid synthase	NANS			
Q8TF72	Protein Shroom3	SHROOM3			
Q9UHJ6	Sedoheptulokinase	SHPK			
Q9NQ25	SLAM family member 7	SLAMF7			
Q13425	Beta-2-syntrophin	SNTB2			
Q9UBY0	Sodium/hydrogen exchanger 2	SLC9A2			
O60284	Suppression of tumorigenicity 18 protein	ST18			
Q9UEE5	Serine/threonine-protein kinase 17A	STK17A			
Q9BVH7	Alpha-N-acetylgalactosaminide alpha-2,6-sialyltransferase 5	ST6GALNAC5			
Q9Y365	START domain-containing protein 10	STARD10			
P0CL83	Putative STAG3-like protein 1	STAG3L1			
P27105	Stomatin	STOM			
P31948	Stress-induced-phosphoprotein 1	STIP1			
O75494	Serine/arginine-rich splicing factor 10	SRSF10			
Q5VZP5	Serine/threonine/tyrosine-interacting-like protein 2	STYXL2			
Q13428	Treacle protein	TCOF1			
A2RTX5	Threonine--tRNA ligase 2, cytoplasmic	TARS3			
Q8N9I0	Synaptotagmin-2	SYT2			

Q8WUA7	TBC1 domain family member 22A	TBC1D22A			
Q9NYV9	Taste receptor type 2 member 13	TAS2R13			
Q9C026	E3 ubiquitin-protein ligase TRIM9	TRIM9			
Q9BVK6	Transmembrane emp24 domain-containing protein 9	TMED9			
P0DTU4	T cell receptor beta chain MC.7.G5	TRB			
Q9UBN6	Tumor necrosis factor receptor superfamily member 10D	TNFRSF10D			
Q8N4H5	Mitochondrial import receptor subunit TOM5 homolog	TOMM5			
Q6ZUK4	Transmembrane protein 26	TMEM26			
A0A0J9YY5	Testis-expressed protein 13D	TEX13D			
Q5T9Z0	Transmembrane epididymal protein 1	TEDDM1			
Q5TGY1	Transmembrane and coiled-coil domain-containing protein 4	TMCO4			
P11388	DNA topoisomerase 2-alpha	TOP2A			
Q9P2J2	Protein turtle homolog A	IGSF9			
Q6PID6	Tetratricopeptide repeat protein 33	TTC33			
Q96AY4	Tetratricopeptide repeat protein 28	TTC28			
O95859	Tetraspanin-12	TSPAN12			
Q96B01	RAD51-associated protein 1	RAD51AP1			
P0C7P2	Putative protein RFPL3S	RFPL3S			
O60673	DNA polymerase zeta catalytic subunit	REV3L			
Q52LD8	Raftlin-2	RFTN2			
Q9HB90	Ras-related GTP-binding protein C	RRAGC			
Q9H1N7	Adenosine 3'-phospho 5'-phosphosulfate transporter 2	SLC35B3			
Q5VVP1	Spermatogenesis-associated protein 31A6	SPATA31A6			
Q8IXU6	Solute carrier family 35 member	SLC35F2			
P48443	Retinoic acid receptor RXR-gamma	RXRG			
Q6YBV0	Proton-coupled amino acid transporter 4	SLC36A4			
Q92777	Synapsin-2	SYN2			
O95197	Reticulon-3	RTN3			
Q6P087	Mitochondrial mRNA pseudouridine synthase RPUSD3	RPUSD3			
O75995	SAM and SH3 domain-containing protein 3	SASH3			
P09234	U1 small nuclear ribonucleoprotein C	SNRPC			
Q9NX18	Succinate dehydrogenase assembly factor 2, mitochondrial	SDHAF2			
Q9NUL5	Shiftless antiviral inhibitor of ribosomal frameshifting protein	SHFL			
P02743	Serum amyloid P-component	APCS			
Q16585	Beta-sarcoglycan	SGCB			
Q8TBC3	SH3KBP1-binding protein 1	SHKBP1			
Q9UPN6	SR-related and CTD-associated factor 8	SCAF8			
Q9NUQ6	SPATS2-like protein	SPATS2L			
Q6AI14	Sodium/hydrogen exchanger 4	SLC9A4			
Q9NY99	Gamma-2-syntrophin	SNTG2			
Q07617	Sperm-associated antigen 1	SPAG1			
Q96KW9	Sperm acrosome-associated protein 7	SPACA7			
Q8WV41	Sorting nexin-33	SNX33			
Q6P435	Putative uncharacterized SMG1-like protein				
Q99717	Mothers against decapentaplegic homolog 5	SMAD5			
P08047	Transcription factor Sp1	SP1			
Q9UNH7	Sorting nexin-6	SNX6			

Q15477	Helicase SKI2W	SKIV2L			
O14512	Suppressor of cytokine signaling 7	SOCS7			
P01242	Growth hormone variant	GH2			
Q495Y8	Speedy protein E2	SPDYE2			
Q14515	SPARC-like protein 1	SPARCL1			
Q9Y651	Transcription factor SOX-21	SOX21			
Q8NFV5	Speedy protein E1	SPDYE1			
Q5TFQ8	Signal-regulatory protein beta-1 isoform 3	SIRPB1			
Q9Y657	Spindlin-1	SPIN1			
Q9P0Z9	Peroxisomal sarcosine oxidase	PIPOX			
Q9Y6H5	Synphilin-1	SNCAIP			
Q9BQI6	SMC5-SMC6 complex localization factor protein 1	SLF1			
Q13796	Protein Shroom2	SHROOM2			
Q9BUA3	Spindlin interactor and repressor of chromatin-binding protein	SPINDOC			
Q8N801	Protein STPG4	STPG4			
Q9BX79	Receptor for retinol uptake STRA6	STRA6			
Q9UBS9	SUN domain-containing ossification factor	SUCO			
O75886	Signal transducing adapter molecule 2	STAM2			
Q9NUL3	Double-stranded RNA-binding protein Staufen homolog 2	STAU2			
A0A0J9YWL9	Putative testis-expressed protein 13C	TEX13C			
Q9UPE1	SRSF protein kinase 3	SRPK3			
P0CV99	Testis-specific Y-encoded protein 4	TSPY4			
Q16623	Syntaxin-1A	STX1A			
Q9Y5M8	Signal recognition particle receptor subunit beta	SRPRB			
Q6ZVD7	Storkhead-box protein 1	STOX1			
Q6UWL2	Sushi domain-containing protein	SUSD1			
Q9UGT4	Sushi domain-containing protein	SUSD2			
Q96L08	Sushi domain-containing protein	SUSD3			
Q9GZT4	Serine racemase	SRR			
Q8NFX7	Syntaxin-binding protein 6	STXBP6			
Q96PV0	Ras/Rap GTPase-activating protein SynGAP	SYNGAP1			
Q9UKP6	Urotensin-2 receptor	UTS2R			
Q9NUC0	SERTA domain-containing protein 4	SERTAD4			
Q13247	Serine/arginine-rich splicing factor 6	SRSF6			
Q14146	Unhealthy ribosome biogenesis protein 2 homolog	URB2			
Q7L3T8	Probable proline--tRNA ligase, mitochondrial	PARS2			
Q9Y5K5	Ubiquitin carboxyl-terminal hydrolase isozyme L5	UCHL5			
P56192	Methionine--tRNA ligase, cytoplasmic	MARS1			
A6NNZ2	Tubulin beta 8B	TUBB8B			
P59538	Taste receptor type 2 member 31	TAS2R31			
Q71U36	Tubulin alpha-1A chain	TUBA1A			
P0C1Z6	TCF3 fusion partner	TFPT			
Q96IK0	Transmembrane protein 101	TMEM101			
Q9NPL8	Complex I assembly factor TIMMDC1, mitochondrial	TIMMDC1			
Q5SRD1	Mitochondrial import inner membrane translocase subunit Tim23B	TIMM23B			
Q92664	Transcription factor IIIA	GTF3A			
Q9Y6L7	Tolloid-like protein 2	TLL2			
O43294	Transforming growth factor beta-1-induced transcript 1 protein	TGFB1I1			
Q5W0B7	Transmembrane protein 236	TMEM236			

Q6ZNB5	Putative short transient receptor potential channel 2-like protein				
Q9NS56	E3 ubiquitin-protein ligase Topors	TOPORS			
Q92547	DNA topoisomerase 2-binding protein 1	TOPBP1			
P67936	Tropomyosin alpha-4 chain	TPM4			
A6NI03	Putative tripartite motif-containing protein 64B	TRIM64B			
A6NLI5	Tripartite motif-containing protein 64C	TRIM64C			
Q9Y3C4	EKC/KEOPS complex subunit TPRKB	TPRKB			
Q6ZVM7	TOM1-like protein 2	TOM1L2			
Q9NX01	Thioredoxin-like protein 4B	TXNL4B			
Q3SXZ7	Probable tubulin polyglutamylase TTLL9	TTLL9			
Q5W5X9	Tetratricopeptide repeat protein 23	TTC23			
P46782	40S ribosomal protein S5	RPS5			
Q6PIV7	Solute carrier family 25 member 34	SLC25A34			
P19793	Retinoic acid receptor RXR-alpha	RXRA			
Q9NSC2	Sal-like protein 1	SALL1			
Q495M3	Proton-coupled amino acid transporter 2	SLC36A2			
Q8N808	Solute carrier family 35 member G3	SLC35G3			
Q15413	Ryanodine receptor 3	RYR3			
Q9BYL1	Sterile alpha motif domain-containing protein 10	SAMD10			
P58743	Prestin	SLC26A5			
Q8TDM5	Sperm acrosome membrane-associated protein 4	SPACA4			
Q08AI6	Putative sodium-coupled neutral amino acid transporter 11	SLC38A11			
Q9NTJ5	Phosphatidylinositol-3-phosphatase SAC1	SACM1L			
Q504Y0	Zinc transporter ZIP12	SLC39A12			
Q9Y2P4	Long-chain fatty acid transport protein 6	SLC27A6			
Q96FQ6	Protein S100-A16	S100A16			
Q8N8U3	Retrotransposon Gag-like protein 3	RTL3			
Q5HYW3	Retrotransposon Gag-like protein 5	RTL5			
Q6ICC9	Retrotransposon Gag-like protein 6	RTL6			
A0AV02	Solute carrier family 12 member 8	SLC12A8			
Q9BY07	Electrogenic sodium bicarbonate cotransporter 4	SLC4A5			
Q9Y2C5	Probable small intestine urate exporter	SLC17A4			
Q96IZ7	Serine/Arginine-related protein 53	RSRC1			
O95136	Sphingosine 1-phosphate receptor 2	S1PR2			
O00631	Sarcolipin	SLN			
Q9BW04	Specifically androgen-regulated gene protein	SARG			
Q96N87	Inactive sodium-dependent neutral amino acid transporter B	SLC6A18			
Q8IVG5	Sterile alpha motif domain-containing protein 9-like	SAMD9L			
Q96CM3	Mitochondrial RNA pseudouridine synthase RPUSD4	RPUSD4			
Q8N2Y8	Iporin	RUSC2			
Q14D33	Receptor-transporting protein 5	RTP5			
Q12788	Transducin beta-like protein 3	TBL3			
P08579	U2 small nuclear ribonucleoprotein B''	SNRPB2			

O00422	Histone deacetylase complex subunit SAP18	SAP18			
Q9BYW2	Histone-lysine N-methyltransferase SETD2	SETD2			
A0A286YF01	Small cysteine and glycine repeat-containing protein 7	SCYGR7			
A0A286YEY9	Small cysteine and glycine repeat-containing protein 1	SCYGR1			
A0A286YF60	Small cysteine and glycine repeat-containing protein 3	SCYGR3			
Q9H7L9	Sin3 histone deacetylase corepressor complex component SDS3	SUDS3			
O15041	Semaphorin-3E	SEMA3E			
Q92854	Semaphorin-4D	SEMA4D			
Q9Y6D0	Selenoprotein K	SELENOK			
A8K8P3	Protein SFI1 homolog	SFI1			
Q9BRG2	SH2 domain-containing protein 3A	SH2D3A			
Q9BVL4	Protein adenylyltransferase SelO, mitochondrial	SELENOO			
P35499	Sodium channel protein type 4 subunit alpha	SCN4A			
Q96HF1	Secreted frizzled-related protein 2	SFRP2			
Q00403	Transcription initiation factor IIB	GTF2B			
O94979	Protein transport protein Sec31A	SEC31A			
P30531	Sodium- and chloride-dependent GABA transporter 1	SLC6A1			
Q15858	Sodium channel protein type 9 subunit alpha	SCN9A			
P84550	SKI family transcriptional corepressor 1	SKOR1			
P48029	Sodium- and chloride-dependent creatine transporter 1	SLC6A8			
Q96BD8	Spindle and kinetochore-associated protein 1	SKA1			
Q147U7	Single-pass membrane and coiled-coil domain-containing protein 1	SMCO1			
Q5TF21	Protein SOGA3	SOGA3			
Q92959	Solute carrier organic anion transporter family member 2A1	SLCO2A1			
P04179	Superoxide dismutase	SOD2			
Q02447	Transcription factor Sp3	SP3			
Q9NX45	Spermatogenesis- and oogenesis-specific basic helix-loop-helix-containing protein 2	SOHLH2			
Q9UPU3	VPS10 domain-containing receptor SorCS3	SORCS3			
Q9P0W8	Spermatogenesis-associated protein 7	SPATA7			
Q5MJ70	Speedy protein A	SPDYA			
P35713	Transcription factor SOX-18	SOX18			
Q7Z572	Spermatogenesis-associated protein 21	SPATA21			
Q13813	Spectrin alpha chain, non-erythrocytic 1	SPTAN1			
Q6PI26	Protein SHQ1 homolog	SHQ1			
O60225	Protein SSX5	SSX5			
Q86WV6	Stimulator of interferon genes protein	STING1			
Q8N0S2	Synaptonemal complex central element protein 1	SYCE1			
P49589	Cysteine--tRNA ligase, cytoplasmic	CARS1			
M5A8F1	Suppressyn	ERVH48-1			
P35610	Sterol O-acyltransferase 1	SOAT1			
P42224	Signal transducer and activator of transcription 1-alpha/beta	STAT1			

Q8NFT2	Metalloreductase STEAP2	STEAP2			
Q08945	FACT complex subunit SSRP1	SSRP1			
Q8IYB3	Serine/arginine repetitive matrix protein 1	SRRM1			
Q9BT76	Uroplakin-3b	UPK3B			
Q8WTU2	Scavenger receptor cysteine-rich domain-containing group B protein	SSC4D			
Q13243	Serine/arginine-rich splicing factor 5	SRSF5			
Q496J9	Synaptic vesicle glycoprotein 2C	SV2C			
Q12772	Sterol regulatory element-binding protein 2	SREBF2			
Q5JPH6	Probable glutamate--tRNA ligase, mitochondrial	EARS2			
Q96I59	Probable asparagine--tRNA ligase, mitochondrial	NARS2			
P57738	T-cell leukemia translocation-altered gene protein	TCTA			
Q9H5J8	TATA box-binding protein-associated factor RNA polymerase I subunit D	TAF1D			
P50991	T-complex protein 1 subunit delta	CCT4			
Q15170	Transcription elongation factor A protein-like 1	TCEAL1			
Q9BUR4	Telomerase Cajal body protein 1	WRAP53			
Q5T160	Probable arginine--tRNA ligase, mitochondrial	RARS2			
Q92519	Tribbles homolog 2	TRIB2			
P0DTU3	T cell receptor alpha chain MC.7.G5	TRA			
Q96RU7	Tribbles homolog 3	TRIB3			
Q5EBN2	Putative tripartite motif-containing protein 61	TRIM61			
Q12974	Protein tyrosine phosphatase type IVA 2	PTP4A2			
Q5JTV8	Torsin-1A-interacting protein 1	TOR1AIP1			
Q15631	Translin	TSN			
P02766	Transthyretin	TTR			
Q9BZW7	Testis-specific gene 10 protein	TSGA10			
A0A578	T cell receptor beta variable 5-1	TRBV5-1			
A0A0K0K1C0	T cell receptor beta variable 11-1	TRBV11-1			
P30530	Tyrosine-protein kinase receptor UFO	AXL			
Q9NPG3	Ubinuclein-1	UBN1			
Q9GZP7	Vomeronasal type-1 receptor 1	VN1R1			
O60437	Periplakin	PPL			
Q96GW7	Brevican core protein	BCAN			
P16234	Platelet-derived growth factor receptor alpha	PDGFRA			
Q13393	Phospholipase D1	PLD1			
Q9UPR0	Inactive phospholipase C-like protein 2	PLCL2			
P78527	DNA-dependent protein kinase catalytic subunit	PRKDC			
P25788	Proteasome subunit alpha type-3	PSMA3			
P55786	Puromycin-sensitive aminopeptidase	NPEPPS			
O43900	Prickle planar cell polarity protein	PRICKLE3			
P86480	Proline-rich protein 20D	PRR20D			
Q92733	Proline-rich protein PRCC	PRCC			
O94906	Pre-mRNA-processing factor 6	PRPF6			
P28065	Proteasome subunit beta type-9	PSMB9			
Q9UI14	Prenylated Rab acceptor protein 1	RABAC1			
Q9BVG9	Phosphatidylserine synthase 2	PTDSS2			
Q9Y620	DNA repair and recombination protein RAD54B	RAD54B			

Q96JH8	Ras-associating and dilute domain-containing protein	RADIL			
O75154	Rab11 family-interacting protein 3	RAB11FIP3			
A6NKH3	Putative 60S ribosomal protein L37a-like protein	RPL37AP8			
Q9NTX7	E3 ubiquitin-protein ligase	RNF146			
Q8WZ75	Roundabout homolog 4	ROBO4			
Q9H1A7	DNA-directed RNA polymerase II subunit RPB11-b2	POLR2J3			
Q5GAN6	Inactive ribonuclease-like protein 10	RNASE10			
Q9NQL2	Ras-related GTP-binding protein D	RRAGD			
P25398	40S ribosomal protein S12	RPS12			
Q71UM5	40S ribosomal protein S27-like	RPS27L			
Q8IX30	Signal peptide, CUB and EGF-like domain-containing protein 3	SCUBE3			
O00193	Small acidic protein	SMAP			
Q96DI7	U5 small nuclear ribonucleoprotein 40 kDa protein	SNRNP40			
Q8TER0	Sushi, nidogen and EGF-like domain-containing protein 1	SNED1			
O43290	U4/U6.U5 tri-snRNP-associated protein 1	SART1			
Q9NY87	Sperm protein associated with the nucleus on the X chromosome C	SPANXC			
Q96BD6	SPRY domain-containing SOCS box protein 1	SPSB1			
Q96R06	Sperm-associated antigen 5	SPAG5			
Q9H173	Nucleotide exchange factor SIL1	SIL1			
Q86XZ4	Spermatogenesis-associated serine-rich protein 2	SPATS2			
Q96RF0	Sorting nexin-18	SNX18			
P19634	Sodium/hydrogen exchanger 1	SLC9A1			
Q9NYZ4	Sialic acid-binding Ig-like lectin 8	SIGLEC8			
O60749	Sorting nexin-2	SNX2			
Q7Z614	Sorting nexin-20	SNX20			
Q96ST3	Paired amphipathic helix protein Sin3a	SIN3A			
Q9H741	SREBP regulating gene protein	SPRING1			
Q07889	Son of sevenless homolog 1	SOS1			
Q8WXH5	Suppressor of cytokine signaling 4	SOCS4			
Q9BXB7	Spermatogenesis-associated protein 16	SPATA16			
P48594	Serpin B4	SERPINB4			
O00241	Signal-regulatory protein beta-1	SIRPB1			
Q9H2G2	STE20-like serine/threonine-protein kinase	SLK			
Q9UQ13	Leucine-rich repeat protein SHOC-2	SHOC2			
Q6ZSJ9	Protein shisa-6	SHISA6			
Q15714	TSC22 domain family protein 1	TSC22D1			
Q9NYV8	Taste receptor type 2 member 14	TAS2R14			
P35442	Thrombospondin-2	THBS2			
Q8WUA8	Tsukushin	TSKU			
A0A0B4J24	T cell receptor alpha variable 5	TRAV5			
Q49AM3	Tetratricopeptide repeat protein 31	TTC31			
Q8NHM4	Putative trypsin-6	PRSS3P2			
Q8NBM4	Ubiquitin-associated domain-containing protein 2	UBAC2			
Q9HAW8	UDP-glucuronosyltransferase	UGT1A10			
Q53H54	tRNA wybutosine-synthesizing protein 2 homolog	TRMT12			
Q9P2K3	REST corepressor 3	RCOR3			
O75901	Ras association domain-containing protein 9	RASSF9			
Q6WBX8	Cell cycle checkpoint control protein RAD9B	RAD9B			

P61578	Endogenous retrovirus group K member 16 Rec protein	ERVK-16			
O75884	Serine hydrolase RBBP9	RBBP9			
Q96AX2	Ras-related protein Rab-37	RAB37			
Q96B86	Repulsive guidance molecule A	RGMA			
P98175	RNA-binding protein 10	RBM10			
P47804	RPE-retinal G protein-coupled receptor	RGR			
Q6NTF9	Rhomboid domain-containing protein 2	RHBDD2			
Q02094	Ammonium transporter Rh type A	RHAG			
Q33E94	Transcription factor RFX4	RFX4			
Q9H4K1	RIB43A-like with coiled-coils protein 2	RIBC2			
Q9P0J6	39S ribosomal protein L36, mitochondrial	MRPL36			
Q96G75	E3 ubiquitin-protein transferase RMND5B	RMND5B			
Q01974	Tyrosine-protein kinase transmembrane receptor ROR2	ROR2			
Q92753	Nuclear receptor ROR-beta	RORB			
P52434	DNA-directed RNA polymerases I, II, and III subunit RPABC3	POLR2H			
Q86VV4	Ran-binding protein 3-like	RANBP3L			
Q9Y4G8	Rap guanine nucleotide exchange factor 2	RAPGEF2			
P0DH78	RING finger protein 224	RNF224			
Q8NBX0	Saccharopine dehydrogenase-like oxidoreductase	SCCPDH			
O00161	Synaptosomal-associated protein 23	SNAP23			
Q92966	snRNA-activating protein complex subunit 3	SNAPC3			
Q13424	Alpha-1-syntrophin	SNTA1			
Q3B7S5	Small integral membrane protein 21	SMIM21			
Q9Y286	Sialic acid-binding Ig-like lectin 7	SIGLEC7			
Q9NRS6	Sorting nexin-15	SNX15			
P0DMW3	Small integral membrane protein 10-like protein 1	SMIM10L1			
Q9UHP9	Small muscular protein	SMPX			
Q9Y5X1	Sorting nexin-9	SNX9			
A0A0U1RRN3	Putative transmembrane protein SPTY2D1OS	SPTY2D1OS			
Q53HV7	Single-strand selective monofunctional uracil DNA glycosylase	SMUG1			
Q8TAQ2	SWI/SNF complex subunit SMARCC2	SMARCC2			
P0DUD4	Putative speedy protein E15	SPDYE15			
O43609	Protein sprouty homolog 1	SPRY1			
Q8IY81	pre-rRNA 2'-O-ribose RNA methyltransferase FTSJ3	FTSJ3			
Q13291	Signaling lymphocytic activation molecule	SLAMF1			
Q8NHS9	Spermatogenesis-associated protein 22	SPATA22			
Q9NTJ3	Structural maintenance of chromosomes protein 4	SMC4			
Q8N4L4	Spermatid maturation protein 1	SPEM1			
Q9BX26	Synaptonemal complex protein 2	SYCP2			
P61956	Small ubiquitin-related modifier 2	SUMO2			
Q8TBR4	Putative STAG3-like protein 4	STAG3L4			
Q9UJ98	Cohesin subunit SA-3	STAG3			
Q9Y2H1	Serine/threonine-protein kinase 38-like	STK38L			
Q96DR4	StAR-related lipid transfer protein 4	STARD4			

Q8NBB2	Putative uncharacterized protein ST20-AS1	ST20-AS1			
Q9HAC7	Succinate--hydroxymethylglutarate CoA-transferase	SUGCT			
Q9UHE8	Metalloreductase STEAP1	STEAP1			
Q658P3	Metalloreductase STEAP3	STEAP3			
O76094	Signal recognition particle subunit SRP72	SRP72			
Q9UNL2	Translocon-associated protein subunit gamma	SSR3			
Q7Z6B7	SLIT-ROBO Rho GTPase-activating protein 1	SRGAP1			
P35346	Somatostatin receptor type 5	SSTR5			
O43752	Syntaxin-6	STX6			
Q6UW78	Ubiquinol-cytochrome-c reductase complex assembly factor 3	UQCC3			
Q9UGK8	Secretion-regulating guanine nucleotide exchange factor	SERGEF			
O94875	Sorbin and SH3 domain-containing protein 2	SORBS2			
O95497	Pantetheinase	VNN1			
A6NER0	TBC1 domain family member 3F	TBC1D3F			
Q86VY9	Transmembrane protein 200A	TMEM200A			
Q5R3I4	Tetratricopeptide repeat protein 38	TTC38			
Q9NUW8	Tyrosyl-DNA phosphodiesterase 1	TDP1			
A0A0A6YYK4	Probable non-functional T cell receptor beta variable 7-1	TRBV7-1			
P20231	Tryptase beta-2	TPSB2			
Q9NYH9	U3 small nucleolar RNA-associated protein 6 homolog	UTP6			
Q6NUM6	S-adenosyl-L-methionine-dependent tRNA 4-demethylwyosine synthase TYW1B	TYW1B			
Q9H320	Variable charge X-linked protein 1	VCX			
P11117	Lysosomal acid phosphatase	ACP2			
P51805	Plexin-A3	PLXNA3			
A0A1B0GWB2	Proline rich transmembrane protein 1B	PRRT1B			
P35998	26S proteasome regulatory subunit 7	PSMC2			
A6NEV1	Proline-rich protein 23A	PRR23A			
O60810	PRAME family member 4	PRAMEF4			
Q7Z4L9	Protein phosphatase 1 regulatory subunit 42	PPP1R42			
O60927	E3 ubiquitin-protein ligase PPP1R11	PPP1R11			
P30044	Peroxiredoxin-5, mitochondrial	PRDX5			
E9PB15	Putative protein PTGES3L	PTGES3L			
Q8TB72	Pumilio homolog 2	PUM2			
O00487	26S proteasome non-ATPase regulatory subunit 14	PSMD14			
P13631	Retinoic acid receptor gamma	RARG			
O00559	Receptor-binding cancer antigen expressed on SiSo cells	EBAG9			
P29373	Cellular retinoic acid-binding protein 2	CRABP2			
O00194	Ras-related protein Rab-27B	RAB27B			
Q9UL25	Ras-related protein Rab-21	RAB21			
P85298	Rho GTPase-activating protein 8	ARHGAP8			
Q9P2N5	RNA-binding protein 27	RBM27			
Q9NW13	RNA-binding protein 28	RBM28			
Q6P4F7	Rho GTPase-activating protein 11A	ARHGAP11A			
O14715	RANBP2-like and GRIP domain-containing protein 8	RGPD8			
P57771	Regulator of G-protein signaling 8	RGS8			

Q8NE09	Regulator of G-protein signaling 22	RGS22			
Q8N9B8	Ras-GEF domain-containing family member 1A	RASGEF1A			
Q96NA2	Rab-interacting lysosomal protein	RILP			
Q13278	Putative protein RIG	RIG			
Q13671	Ras and Rab interactor 1	RIN1			
P35268	60S ribosomal protein L22	RPL22			
P39023	60S ribosomal protein L3	RPL3			
Q96DM3	Regulator of MON1-CCZ1 complex	RMC1			
P51449	Nuclear receptor ROR-gamma	RORC			
P84103	Serine/arginine-rich splicing factor 3	SRSF3			
Q5VWG9	Transcription initiation factor TFIID subunit 3	TAF3			
Q9NYW5	Taste receptor type 2 member 4	TAS2R4			
Q96S53	Dual specificity testis-specific protein kinase 2	TESK2			
Q86X45	Protein tilB homolog	LRRC6			
E9PQX1	Transmembrane protein 262	TMEM262			
A6NKF7	Transmembrane protein 88B	TMEM88B			
Q6GV28	Transmembrane protein 225	TMEM225			
Q96QE5	Transcription elongation factor, mitochondrial	TEFM			
Q5HYL7	Transmembrane protein 196	TMEM196			
A0A0A0MS02	Probable non-functional T cell receptor gamma variable	TRGV1			
O95407	Tumor necrosis factor receptor superfamily member 6B	TNFRSF6B			
Q9NQ34	Transmembrane protein 9B	TMEM9B			
Q7Z402	Transmembrane channel-like protein 7	TMC7			
Q5GJ75	Tumor necrosis factor alpha-induced protein 8-like protein 3	TNFAIP8L3			
Q13445	Transmembrane emp24 domain-containing protein 1	TMED1			
Q969K7	Transmembrane protein 54	TMEM54			
Q9UM00	Calcium load-activated calcium channel	TMCO1			
Q9BXS4	Transmembrane protein 59	TMEM59			
P08138	Tumor necrosis factor receptor superfamily member 16	NGFR			
Q9GZX9	Twisted gastrulation protein homolog 1	TWSG1			
Q6DKK2	Tetratricopeptide repeat protein 19, mitochondrial	TTC19			
Q6UWH6	Protein TEX261	TEX261			
A6NLP5	Tetratricopeptide repeat protein 36	TTC36			
A2A3L6	Tetratricopeptide repeat protein 24	TTC24			
O00294	Tubby-related protein 1	TULP1			
Q9NRR2	Tryptase gamma	TPSG1			
A0A0A6YYG2	T cell receptor beta variable 6-6	TRBV6-6			
P07478	Trypsin-2	PRSS2			
A0A0K0K1G8	T cell receptor beta variable 10-2	TRBV10-2			
Q96BW1	Uracil phosphoribosyltransferase homolog	UPRT			
O95551	Tyrosyl-DNA phosphodiesterase 2	TDP2			
Q8NB66	Protein unc-13 homolog C	UNC13C			
Q9H9P5	Putative E3 ubiquitin-protein ligase UNKL	UNKL			
O75643	U5 small nuclear ribonucleoprotein 200 kDa helicase	SNRNP200			
Q96PU4	E3 ubiquitin-protein ligase UHRF2	UHRF2			

Q5W0Q7	SUMO-specific isopeptidase USPL1	USPL1			
Q3LFD5	Putative ubiquitin carboxyl-terminal hydrolase 41	USP41			
Q9H322	Variable charge X-linked protein 2	VCX2			
O95183	Vesicle-associated membrane protein 5	VAMP5			
Q8NEY4	V-type proton ATPase subunit C 2	ATP6V1C2			
Q658N2	WSC domain-containing protein 1	WSCD1			
Q8NGP4	Olfactory receptor 5M3	OR5M3			
Q8NGR6	Olfactory receptor 1B1	OR1B1			
O43913	Origin recognition complex subunit 5	ORC5			
Q96FX8	p53 apoptosis effector related to PMP-22	PERP			
O60925	Prefoldin subunit 1	PFDN1			
Q96S96	Phosphatidylethanolamine-binding protein 4	PEBP4			
P49190	Parathyroid hormone 2 receptor	PTH2R			
P51665	26S proteasome non-ATPase regulatory subunit 7	PSMD7			
P20742	Pregnancy zone protein	PZP			
Q04864	Proto-oncogene c-Rel	REL			
Q8NDT2	Putative RNA-binding protein 15B	RBM15B			
Q93062	RNA-binding protein with multiple splicing	RBPMS			
Q9NP72	Ras-related protein Rab-18	RAB18			
P59190	Ras-related protein Rab-15	RAB15			
O43182	Rho GTPase-activating protein 6	ARHGAP6			
P61018	Ras-related protein Rab-4B	RAB4B			
Q96QB1	Rho GTPase-activating protein 7	DLC1			
P0C7M4	Rhox homeobox family member 2B	RHOXF2B			
Q8N392	Rho GTPase-activating protein 18	ARHGAP18			
Q9NVN3	Synembryn-B	RIC8B			
Q2KHR2	DNA-binding protein RFX7	RFX7			
Q86VR2	Reticulophagy regulator 3	RETREG3			
P62891	60S ribosomal protein L39	RPL39			
Q92901	60S ribosomal protein L3-like	RPL3L			
Q9BZE1	39S ribosomal protein L37, mitochondrial	MRPL37			
Q02878	60S ribosomal protein L6	RPL6			
Q86TS9	39S ribosomal protein L52, mitochondrial	MRPL52			
P05423	DNA-directed RNA polymerase III subunit RPC4	POLR3D			
P61587	Rho-related GTP-binding protein RhoE	RND3			
Q8NCN4	E3 ubiquitin-protein ligase	RNF169			
Q5VTB9	E3 ubiquitin-protein ligase	RNF220			
Q14684	Ribosomal RNA processing protein 1 homolog B	RRP1B			
P62263	40S ribosomal protein S14	RPS14			
Q14765	Signal transducer and activator of transcription 4	STAT4			
Q6ZW31	Rho GTPase-activating protein SYDE1	SYDE1			
O95363	Phenylalanine--tRNA ligase, mitochondrial	FARS2			
O43426	Synaptojanin-1	SYNJ1			
Q9H987	Synaptopodin 2-like protein	SYNPO2L			
Q9NYJ8	TGF-beta-activated kinase 1 and MAP3K7-binding protein 2	TAB2			
Q9UFV1	Putative TBC1 domain family member 29	TBC1D29P			
A6NFQ2	TRPM8 channel-associated factor 2	TCAF2			
Q9ULP9	TBC1 domain family member 24	TBC1D24			

P23193	Transcription elongation factor A protein 1	TCEA1			
Q9Y6A5	Transforming acidic coiled-coil-containing protein 3	TACC3			
Q7L8C5	Synaptotagmin-13	SYT13			
Q17RD7	Synaptotagmin-16	SYT16			
Q9NP81	Serine--tRNA ligase, mitochondrial	SARS2			
Q6NT04	Tigger transposable element-derived protein 7	TIGD7			
B9EJG8	Transmembrane protein 150C	TMEM150C			
Q8N841	Tubulin polyglutamylase TTLL6	TTLL6			
Q99816	Tumor susceptibility gene 101 protein	TSG101			
Q9NQE7	Thymus-specific serine protease	PRSS16			
Q8IWX7	Protein unc-45 homolog B	UNC45B			
O75310	UDP-glucuronosyltransferase	UGT2B11			
Q70CQ4	Ubiquitin carboxyl-terminal hydrolase 31	USP31			
P38606	V-type proton ATPase catalytic subunit A	ATP6V1A			
Q6Q795	Putative viral protein-binding protein C1				
Q7L8A9	Tubulinyl-Tyr carboxypeptidase 1	VASH1			
P36543	V-type proton ATPase subunit E 1	ATP6V1E1			
Q5GH72	XK-related protein 7	XKR7			
Q702N8	Xin actin-binding repeat-containing protein 1	XIRP1			
O75379	Vesicle-associated membrane protein 4	VAMP4			
P04628	Proto-oncogene Wnt-1	WNT1			
Q9Y4P8	WD repeat domain phosphoinositide-interacting protein 2	WIPI2			
Q8WZA6	Olfactory receptor 1E3	OR1E3			
Q8NGG8	Olfactory receptor 8B3	OR8B3			
Q7Z3B4	Nucleoporin p54	NUP54			
Q14982	Opioid-binding protein/cell adhesion molecule	OPCML			
Q96M98	Parkin coregulated gene protein	PACRG			
Q9P0S3	ORM1-like protein 1	ORMDL1			
P0C869	Cytosolic phospholipase A2 beta	PLA2G4B			
O75781	Paralemmin-1	PALM			
Q07343	cAMP-specific 3',5'-cyclic phosphodiesterase 4B	PDE4B			
Q96A54	Adiponectin receptor protein 1	ADIPOR1			
Q08499	cAMP-specific 3',5'-cyclic phosphodiesterase 4D	PDE4D			
P04085	Platelet-derived growth factor subunit A	PDGFA			
Q9Y5H8	Protocadherin alpha-3	PCDHA3			
Q9UQ80	Proliferation-associated protein 2G4	PA2G4			
Q9UN72	Protocadherin alpha-7	PCDHA7			
O14917	Protocadherin-17	PCDH17			
P05166	Propionyl-CoA carboxylase beta chain, mitochondrial	PCCB			
Q9UN66	Protocadherin beta-8	PCDHB8			
Q63HM2	Pecanex-like protein 4	PCNX4			
Q8N807	Protein disulfide-isomerase-like protein of the testis	PDILT			
Q9Y5B6	PAX3- and PAX7-binding protein 1	PAXBP1			
Q9H814	Phosphorylated adapter RNA export protein	PHAX			
P0DJD7	Pepsin A-4	PGA4			
Q9H3S5	GPI mannosyltransferase 1	PIGM			
Q8N3S3	Protein PHTF2	PHTF2			
Q8WWQ0	PH-interacting protein	PHIP			

Q8N3A8	Protein mono-ADP-ribosyltransferase PARP8	PARP8			
Q8IXQ6	Protein mono-ADP-ribosyltransferase PARP9	PARP9			
Q9NUG6	p53 and DNA damage-regulated protein 1	PDRG1			
Q9UHG2	ProSAAS	PCSK1N			
P20941	Phosducin	PDC			
P15259	Phosphoglycerate mutase 2	PGAM2			
Q9NQP4	Prefoldin subunit 4	PFDN4			
Q12837	POU domain, class 4, transcription factor 2	POU4F2			
P54277	PMS1 protein homolog 1	PMS1			
P05187	Alkaline phosphatase, placental type	ALPP			
P30405	Peptidyl-prolyl cis-trans isomerase F, mitochondrial	PPIF			
O95428	Papilin	PAPLN			
P50336	Protoporphyrinogen oxidase	PPOX			
P08567	Pleckstrin	PLEK			
Q8N3J5	Protein phosphatase 1K, mitochondrial	PPM1K			
Q12972	Nuclear inhibitor of protein phosphatase 1	PPP1R8			
Q9H8W4	Pleckstrin homology domain-containing family F member 2	PLEKHF2			
Q99697	Pituitary homeobox 2	PITX2			
Q9BRC7	1-phosphatidylinositol 4,5-bisphosphate phosphodiesterase delta-4	PLCD4			
Q96S99	Pleckstrin homology domain-containing family F member 1	PLEKHF1			
Q7Z736	Pleckstrin homology domain-containing family H member 3	PLEKHH3			
P04280	Basic salivary proline-rich protein	PRB1			
Q6UWY2	Serine protease 57	PRSS57			
P16885	1-phosphatidylinositol 4,5-bisphosphate phosphodiesterase gamma-2	PLCG2			
P10163	Basic salivary proline-rich protein	PRB4			
Q9GZV8	PR domain zinc finger protein 14	PRDM14			
Q92954	Proteoglycan 4	PRG4			
Q16557	Pregnancy-specific beta-1-glycoprotein 3	PSG3			
I3L0S3	Putative uncharacterized protein PYCARD-AS1	PYCARD-AS1			
Q16851	UTP--glucose-1-phosphate uridylyltransferase	UGP2			
Q9UHP3	Ubiquitin carboxyl-terminal hydrolase 25	USP25			
Q9H7C4	Syncoilin	SYNC			
Q9UMS6	Synaptopodin-2	SYNPO2			
Q5QJ38	Trichohyalin-like protein 1	TCHHL1			
Q9H4B7	Tubulin beta-1 chain	TUBB1			
Q8N103	T-cell activation Rho GTPase-activating protein	TAGAP			
Q8N3G9	Transmembrane protein 130	TMEM130			
Q96C24	Synaptotagmin-like protein 4	SYTL4			
Q6N022	Teneurin-4	TENM4			
O75865	Trafficking protein particle complex subunit 6A	TRAPPC6A			
Q969P6	DNA topoisomerase I, mitochondrial	TOP1MT			
P49755	Transmembrane emp24 domain-containing protein 10	TMED10			
Q7Z5M5	Transmembrane channel-like protein 3	TMC3			
Q6P2H8	Transmembrane protein 53	TMEM53			
Q13077	TNF receptor-associated factor 1	TRAF1			

Q2T9K0	Transmembrane protein 44	TMEM44			
O43396	Thioredoxin-like protein 1	TXNL1			
A0A0A6YYK6	T cell receptor alpha variable 16	TRAV16			
Q5VTQ0	Tetratricopeptide repeat protein 39B	TTC39B			
Q9Y5S1	Transient receptor potential cation channel subfamily V member 2	TRPV2			
Q9H1Z9	Tetraspanin-10	TSPAN10			
O60636	Tetraspanin-2	TSPAN2			
Q96PN8	Testis-specific serine/threonine-protein kinase 3	TSSK3			
Q9C0C9		UBE2O			
P16662	UDP-glucuronosyltransferase 2B7	UGT2B7			
O60656	UDP-glucuronosyltransferase 1A9	UGT1A9			
P26368	Splicing factor U2AF 65 kDa subunit	U2AF2			
Q15853	Upstream stimulatory factor 2	USF2			
P11684	Uteroglobin	SCGB1A1			
Q96JH7	Deubiquitinating protein VCPIP1	VCPIP1			
Q9Y4E6	WD repeat-containing protein 7	WDR7			
O15537	Retinoschisin	RS1			
O43543	DNA repair protein XRCC2	XRCC2			
P04004	Vitronectin	VTN			
Q9UIA9	Exportin-7	XPO7			
Q8N3P4	Vacuolar protein sorting-associated protein 8 homolog	VPS8			
Q9HAV4	Exportin-5	XPO5			
Q6IFH4	Olfactory receptor 6B2	OR6B2			
Q9UHM6	Melanopsin	OPN4			
Q6U736	Opsin-5	OPN5			
P18509	Pituitary adenylate cyclase-activating polypeptide	ADCYAP1			
Q969R2	Oxysterol-binding protein 2	OSBP2			
Q9ULJ1	Protein BCAP	ODF2L			
Q99650	Oncostatin-M-specific receptor subunit beta	OSMR			
Q8N138	ORM1-like protein 3	ORMDL3			
P04054	Phospholipase A2	PLA2G1B			
P47900	P2Y purinoceptor 1	P2RY1			
Q9P286	Serine/threonine-protein kinase PAK 5	PAK5			
O00750	Phosphatidylinositol 4-phosphate 3-kinase C2 domain-containing subunit beta	PIK3C2B			
O00443	Phosphatidylinositol 4-phosphate 3-kinase C2 domain-containing subunit alpha	PIK3C2A			
P07237	Protein disulfide-isomerase	P4HB			
O95427	GPI ethanolamine phosphate transferase 1	PIGN			
Q32NB8	CDP-diacylglycerol--glycerol-3-phosphate 3-phosphatidyltransferase, mitochondrial	PGS1			
P00558	Phosphoglycerate kinase 1	PGK1			
P07205	Phosphoglycerate kinase 2	PGK2			
Q8N7G0	POU domain, class 5, transcription factor 2	POU5F2			
O94903	Pyridoxal phosphate homeostasis protein	PLPBP			
Q9H7Z7	Prostaglandin E synthase 2	PTGES2			
O43447	Peptidyl-prolyl cis-trans isomerase H	PPIH			
Q9NP80	Calcium-independent phospholipase A2-gamma	PNPLA8			
Q86YR6	POTE ankyrin domain family member D	POTED			
O15355	Protein phosphatase 1G	PPM1G			
Q99569	Plakophilin-4	PKP4			

Q6S5H5	POTE ankyrin domain family member G	POTEG			
O43586	Proline-serine-threonine phosphatase-interacting protein 1	PSTPIP1			
Q9HBL7	Plasminogen receptor	PLGRKT			
Q9ULL1	Pleckstrin homology domain-containing family G member 1	PLEKHG1			
Q8TDX9	Polycystic kidney disease protein 1-like 1	PKD1L1			
A0A1B0GVH4	Serine protease-like protein 51	PRSS51			
O75807	Protein phosphatase 1 regulatory subunit 15A	PPP1R15A			
Q9NQW5	Probable histone-lysine N-methyltransferase PRDM7	PRDM7			
Q9NQX1	PR domain zinc finger protein 5	PRDM5			
O75127	Pentatricopeptide repeat-containing protein 1, mitochondrial	PTCD1			
Q8IV42	L-seryl-tRNA	PSTK			
P17812	CTP synthase 1	CTPS1			
P62857	40S ribosomal protein S28	RPS28			
Q9BZZ2	Sialoadhesin	SIGLEC1			
Q53GS9	U4/U6.U5 tri-snRNP-associated protein 2	USP39			
O60248	Protein SOX-15	SOX15			
Q6UW49	Sperm equatorial segment protein 1	SPESP1			
Q9BPZ2	Spindlin-2B	SPIN2B			
P35237	Serpin B6	SERPINB6			
Q96JF0	Beta-galactoside alpha-2,6-sialyltransferase 2	ST6GAL2			
Q5VXU9	Protein shortage in chiasmata 1 ortholog	SHOC1			
Q15431	Synaptonemal complex protein 1	SYCP1			
Q9P2F8	Signal-induced proliferation-associated 1-like protein 2	SIPA1L2			
O60292	Signal-induced proliferation-associated 1-like protein 3	SIPA1L3			
Q9BR01	Sulfotransferase 4A1	SULT4A1			
Q8IWL8	Saitohin	STH			
Q9H6E5	Speckle targeted PIP5K1A-regulated poly	TUT1			
Q8TDR2	Serine/threonine-protein kinase	STK35			
P32856	Syntaxin-2	STX2			
Q7Z3V4	Ubiquitin-protein ligase E3B	UBE3B			
A6NHL2	Tubulin alpha chain-like 3	TUBAL3			
O43680	Transcription factor 21	TCF21			
Q5H9L2	Transcription elongation factor A protein-like 5	TCEAL5			
Q6IPX3	Transcription elongation factor A protein-like 6	TCEAL6			
Q15542	Transcription initiation factor TFIID subunit 5	TAF5			
O60806	T-box transcription factor TBX19	TBX19			
P53999	Activated RNA polymerase II transcriptional coactivator p15	SUB1			
O14894	Transmembrane 4 L6 family member 5	TM4SF5			
Q9H2D6	TRIO and F-actin-binding protein	TRIOBP			
O15040	Tectonin beta-propeller repeat-containing protein 2	TECPR2			
Q8IV01	Synaptotagmin-12	SYT12			
O43156	TELO2-interacting protein 1 homolog	TTI1			
Q9Y4A5	Transformation/transcription domain-associated protein	TRRAP			
Q9BY64	UDP-glucuronosyltransferase	UGT2B28			

Q9GZZ9	Ubiquitin-like modifier-activating enzyme 5	UBA5			
Q709C8	Vacuolar protein sorting-associated protein 13C	VPS13C			
Q8NFA0	Ubiquitin carboxyl-terminal hydrolase 32	USP32			
Q86TI4	WD repeat-containing protein 86	WDR86			
A0A1B0GUX0	Protein ATP6V1FNB	ATP6V1FNB			
A6NLU5	V-set and transmembrane domain-containing protein 2B	VSTM2B			
P49754	Vacuolar protein sorting-associated protein 41 homolog	VPS41			
O43542	DNA repair protein XRCC3	XRCC3			
Q8TCV5	WAP four-disulfide core domain protein 5	WFDC5			
Q1A5X7	Putative WASP homolog-associated protein with actin, membranes and microtubules-like protein 1	WHAMMP3			
Q8N1B4	Vacuolar protein sorting-associated protein 52 homolog	VPS52			
A2RRH5	WD repeat-containing protein 27	WDR27			
P42768	Wiskott-Aldrich syndrome protein	WAS			
Q9NRI6	Putative peptide YY-2	PYY2			
Q01201	Transcription factor RelB	RELB			
Q15907	Ras-related protein Rab-11B	RAB11B			
Q8N0V3	Putative ribosome-binding factor A, mitochondrial	RBFA			
P42696	RNA-binding protein 34	RBM34			
Q8N1G1	RNA exonuclease 1 homolog	REXO1			
Q3MIN7	Ral guanine nucleotide dissociation stimulator-like 3	RGL3			
Q52LW3	Rho GTPase-activating protein 29	ARHGAP29			
Q9BYZ6	Rho-related BTB domain-containing protein 2	RHOBTB2			
O75783	Rhomboid-related protein 1	RHBDL1			
Q9H628	Ras-related and estrogen-regulated growth inhibitor-like protein	RERGL			
Q7Z5B4	Protein RIC-3	RIC3			
P08134	Rho-related GTP-binding protein RhoC	RHOC			
A6NNX1	RIIa domain-containing protein 1	RIIAD1			
P46778	60S ribosomal protein L21	RPL21			
P49406	39S ribosomal protein L19, mitochondrial	MRPL19			
Q9H9V4	RING finger protein 122	RNF122			
Q8N7C7	RING finger protein 148	RNF148			
Q96TC7	Regulator of microtubule dynamics protein 3	RMDN3			
Q6P161	39S ribosomal protein L54, mitochondrial	MRPL54			
Q13129	Zinc finger protein Rlf	RLF			
Q96MS0	Roundabout homolog 3	ROBO3			
O75575	DNA-directed RNA polymerase III subunit RPC9	CRCP			
Q9BUI4	DNA-directed RNA polymerase III subunit RPC3	POLR3C			
Q8TDE3	Ribonuclease 8	RNASE8			
Q9NXI6	E3 ubiquitin-protein ligase	RNF186			
Q9H668	CST complex subunit STN1	STN1			
Q9UJ37	Alpha-N-acetylgalactosaminide alpha-2,6-sialyltransferase 2	ST6GALNAC2			
Q8NBK3	Formylglycine-generating enzyme	SUMF1			
Q8IWU5	Extracellular sulfatase Sulf-2	SULF2			
P49223	Kunitz-type protease inhibitor 3	SPINT3			
Q9UBI4	Stomatin-like protein 1	STOML1			
O00506	Serine/threonine-protein kinase	STK25			

O75716	Serine/threonine-protein kinase	STK16			
A6NDV4	Transmembrane protein 8B	TMEM8B			
Q13033	Striatin-3	STRN3			
Q9H7F4	Transmembrane protein 185B	TMEM185B			
P09758	Tumor-associated calcium signal transducer 2	TACSTD2			
Q9Y2B4	TP53-target gene 5 protein	TP53TG5			
Q96SF2	T-complex protein 1 subunit theta-like 2	CCT8L2			
P0CG34	Thymosin beta-15A	TMSB15A			
Q9NY65	Tubulin alpha-8 chain	TUBA8			
Q6N021	Methylcytosine dioxygenase TET2	TET2			
Q10587	Thyrotroph embryonic factor	TEF			
P03986	T cell receptor gamma constant 2	TRGC2			
Q9Y577	E3 ubiquitin-protein ligase	TRIM17			
Q9NXH9	tRNA	TRMT1			
P48553	Trafficking protein particle complex subunit 10	TRAPPC10			
O75888	Tumor necrosis factor ligand superfamily member 13	TNFSF13			
Q56UQ5	TPT1-like protein				
Q14106	Protein Tob2	TOB2			
Q96KP6	TNFAIP3-interacting protein 3	TNIP3			
Q8NBS9	Thioredoxin domain-containing protein 5	TXNDC5			
Q5T7P6	Transmembrane protein 78	TMEM78			
Q06418	Tyrosine-protein kinase receptor TYRO3	TYRO3			
Q96HH6	Transmembrane protein 19	TMEM19			
Q9NX07	tRNA selenocysteine 1-associated protein 1	TRNAU1AP			
Q9NRE2	Teashirt homolog 2	TSHZ2			
A0A0K0K1C4	T cell receptor beta variable 27	TRBV27			
P49815	Tuberin	TSC2			
A0A5A6	T cell receptor beta variable 11-3	TRBV11-3			
Q6UWM9	UDP-glucuronosyltransferase 2A3	UGT2A3			
C9JVI0	Ubiquitin carboxyl-terminal hydrolase 17-like protein 11	USP17L11			
Q765I0	Urotensin-2B	UTS2B			
Q9UIW0	Ventral anterior homeobox 2	VAX2			
Q3MJ13	WD repeat-containing protein 72	WDR72			
P63119	Endogenous retrovirus group K member 21 Pro protein	ERVK-21			
Q9HBG4	V-type proton ATPase 116 kDa subunit a isoform 4	ATP6V0A4			
P63125	Endogenous retrovirus group K member 25 Pro protein	ERVK-25			
P23025	DNA repair protein complementing XP-A cells	XPA			
O14980	Exportin-1	XPO1			
Q9H270	Vacuolar protein sorting-associated protein 11 homolog	VPS11			
O76024	Wolframin	WFS1			
Q96S15	GATOR complex protein WDR24	WDR24			
Q9H1Z4	WD repeat-containing protein 13	WDR13			
Q8NEZ3	WD repeat-containing protein 19	WDR19			
Q15061	WD repeat-containing protein 43	WDR43			
Q9NQA3	WAS protein family homolog 6	WASH6P			
Q8TAF8	LHFPL tetraspan subfamily member 5 protein	LHFPL5			
Q659C4	La-related protein 1B	LARP1B			
O75334	Liprin-alpha-2	PPFIA2			
P98164	Low-density lipoprotein receptor-related protein 2	LRP2			
Q8N309	Leucine-rich repeat-containing protein 43	LRRC43			
Q8WV35	Leucine-rich repeat-containing protein 29	LRRC29			

Q9BY71	Leucine-rich repeat-containing protein 3	LRRC3			
Q7Z2Q7	Leucine-rich repeat-containing protein 70	LRRC70			
Q96JA1	Leucine-rich repeats and immunoglobulin-like domains protein 1	LRIG1			
Q8IVB5	LIX1-like protein	LIX1L			
Q9BYJ1	Hydroperoxide isomerase ALOXE3	ALOXE3			
Q9UBY5	Lysophosphatidic acid receptor 3	LPAR3			
Q9H3W5	Leucine-rich repeat neuronal protein 3	LRRN3			
Q8NDX9	Lymphocyte antigen 6 complex locus protein G5b	LY6G5B			
Q58DX5	Inactive N-acetylated-alpha-linked acidic dipeptidase-like protein 2	NAALADL2			
O75608	Acyl-protein thioesterase 1	LYPLA1			
Q02078	Myocyte-specific enhancer factor 2A	MEF2A			
O95372	Acyl-protein thioesterase 2	LYPLA2			
Q02080	Myocyte-specific enhancer factor 2B	MEF2B			
Q96PE7	Methylmalonyl-CoA epimerase, mitochondrial	MCEE			
Q96AQ8	Mitochondrial calcium uniporter regulator 1	MCUR1			
O00470	Homeobox protein Meis1	MEIS1			
P51948	CDK-activating kinase assembly factor MAT1	MNAT1			
Q56UN5	Mitogen-activated protein kinase kinase kinase 19	MAP3K19			
Q9Y6R4	Mitogen-activated protein kinase kinase kinase 4	MAP3K4			
O15151	Protein Mdm4	MDM4			
Q5VZF2	Muscleblind-like protein 2	MBNL2			
Q9Y2H9	Microtubule-associated serine/threonine-protein kinase 1	MAST1			
Q9BXY0	Protein MAK16 homolog	MAK16			
Q9BRQ6	MICOS complex subunit MIC25	CHCHD6			
H3BPM6	MKRN2 opposite strand protein	MKRN2OS			
Q8N108	Mesoderm induction early response protein 1	MIER1			
Q9BRT3	Migration and invasion enhancer	MIEN1			
P0C024	Peroxisomal coenzyme A diphosphatase NUDT7	NUDT7			
O95140	Mitofusin-2	MFN2			
Q8N5J2	Ubiquitin carboxyl-terminal hydrolase MINDY-1	MINDY1			
Q8N4C8	Misshapen-like kinase 1	MINK1			
Q4G0A6	Probable ubiquitin carboxyl-terminal hydrolase MINDY-4	MINDY4			
A6NI15	Mesogenin-1	MSGN1			
O75970	Multiple PDZ domain protein	MPDZ			
P30305	M-phase inducer phosphatase 2	CDC25B			
O95907	Monocarboxylate transporter 3	SLC16A8			
Q8NGS4	Olfactory receptor 13F1	OR13F1			
A6NCE7	Microtubule-associated proteins 1A/1B light chain 3 beta 2	MAP1LC3B2			
Q5TGP6	Maestro heat-like repeat-containing protein family member 9	MROH9			
P36507	Dual specificity mitogen-activated protein kinase kinase 2	MAP2K2			
O43193	Motilin receptor	MLNR			
Q9Y6R0	Numb-like protein	NUMBL			
Q15612	Olfactory receptor 1Q1	OR1Q1			
Q8WXC3	Pyrin domain-containing protein 1	PYDC1			

P54750	Calcium/calmodulin-dependent 3',5'-cyclic nucleotide phosphodiesterase 1A	PDE1A			
Q9Y2J8	Protein-arginine deiminase type-	PADI2			
Q6TCH4	Membrane progestin receptor delta	PAQR6			
Q6TCH7	Progestin and adipoQ receptor family member 3	PAQR3			
Q13946	High affinity cAMP-specific 3',5'-cyclic phosphodiesterase 7A	PDE7A			
Q9ULR5	Polyadenylate-binding protein-interacting protein 2B	PAIP2B			
Q8N6Y1	Protocadherin-20	PCDH20			
Q9H244	P2Y purinoceptor 12	P2RY12			
Q9Y5E6	Protocadherin beta-3	PCDHB3			
Q9BPZ3	Polyadenylate-binding protein-interacting protein 2	PAIP2			
A0A087WWA1	PIK3R3 upstream open reading frame protein	P3R3URF			
A6NC86	phospholipase A2 inhibitor and Ly6/PLAUR domain-containing protein	PINLYP			
Q9NRF8	CTP synthase 2	CTPS2			
Q08257	Quinone oxidoreductase	CRYZ			
Q7L1I2	Synaptic vesicle glycoprotein 2B	SV2B			
P37108	Signal recognition particle 14 kDa protein	SRP14			
Q9NRH3	Tubulin gamma-2 chain	TUBG2			
Q12870	Transcription factor 15	TCF15			
Q9HCH5	Synaptotagmin-like protein 2	SYTL2			
O43615	Mitochondrial import inner membrane translocase subunit TIM44	TIMM44			
Q8NDZ6	Transmembrane protein 161B	TMEM161B			
P0CAT3	Putative TLX1 neighbor protein	TLX1NB			
Q8WVE7	Transmembrane protein 170A	TMEM170A			
P16035	Metalloproteinase inhibitor 2	TIMP2			
Q9BSN7	Transmembrane protein 204	TMEM204			
Q9UDY6	Tripartite motif-containing protein 10	TRIM10			
O14788	Tumor necrosis factor ligand superfamily member 11	TNFSF11			
Q86WS5	Transmembrane protease serine 12	TMPRSS12			
O75962	Triple functional domain protein	TRIO			
Q9BSJ1	Tripartite motif-containing protein 51	TRIM51			
Q9Y320	Thioredoxin-related transmembrane protein 2	TMX2			
O14530	Thioredoxin domain-containing protein 9	TXNDC9			
Q8TAI1	TYMS opposite strand protein	TYMSOS			
Q7RTX1	Taste receptor type 1 member 1	TAS1R1			
Q9BTX7	Alpha-tocopherol transfer protein-like	TTPAL			
A0A0B4J1U6	T cell receptor beta variable 9	TRBV9			
A0A0C4DH59	T cell receptor beta variable 5-4	TRBV5-4			
D6RJB6	Ubiquitin carboxyl-terminal hydrolase 17-like protein 20	USP17L20			
Q8WVF2	Unique cartilage matrix-associated protein	UCMA			
Q96RL1	BRCA1-A complex subunit RAP80	UIMC1			
Q96KR7	Phosphatase and actin regulator 3	PHACTR3			
Q01860	POU domain, class 5, transcription factor 1	POU5F1			
Q496M5	Inactive serine/threonine-protein kinase PLK5	PLK5			

Q8IXY8	Probable inactive peptidyl-prolyl cis-trans isomerase-like 6	PPIL6			
A8MQ11	Postmeiotic segregation increased 2-like protein 5	PMS2P5			
P0CG38	POTE ankyrin domain family member I	POTEI			
Q13835	Plakophilin-1	PKP1			
Q4J6C6	Prolyl endopeptidase-like	PREPL			
Q9HBU9	Popeye domain-containing protein 2	POPDC2			
Q96C90	Protein phosphatase 1 regulatory subunit 14B	PPP1R14B			
A1L4L8	PLAC8-like protein 1	PLAC8L1			
Q9UM63	Zinc finger protein PLAGL1	PLAGL1			
Q6ZS11	Ras and Rab interactor-like protein	RINL			
P04156	Major prion protein	PRNP			
Q96J94	Piwi-like protein 1	PIWIL1			
Q9Y253	DNA polymerase eta	POLH			
O94763	Unconventional prefoldin RPB5 interactor 1	URI1			
O75526	RNA-binding motif protein, X-linked-like-2	RBMXL2			
Q9H871	E3 ubiquitin-protein transferase RMND5A	RMND5A			
P0DPB5	Protein POLR1D, isoform 2	POLR1D			
Q13905	Rap guanine nucleotide exchange factor 1	RAPGEF1			
P56182	Ribosomal RNA processing protein 1 homolog A	RRP1			
Q8TC41	Probable E3 ubiquitin-protein ligase RNF217	RNF217			
P62070	Ras-related protein R-Ras2	RRAS2			
Q9H633	Ribonuclease P protein subunit p21	RPP21			
A6NMZ2	Sentan	SNTN			
Q9Y5X0	Sorting nexin-10	SNX10			
Q9NWM0	Spermine oxidase	SMOX			
P0C7V6	Putative transcription factor SPT20 homolog-like 2	SUPT20HL2			
Q8NDZ2	SUMO-interacting motif-containing protein 1	SIMC1			
Q9UIG8	Solute carrier organic anion transporter family member 3A1	SLCO3A1			
Q9H2Y9	Solute carrier organic anion transporter family member 5A1	SLCO5A1			
Q96PX8	SLIT and NTRK-like protein 1	SLITRK1			
A0A1B0GW64	Small integral membrane protein 33	SMIM33			
Q8IXJ6	NAD-dependent protein deacetylase sirtuin-2	SIRT2			
P63272	Transcription elongation factor SPT4	SUPT4H1			
Q9BQB4	Sclerostin	SOST			
Q7Z5L4	Spermatogenesis-associated protein 19, mitochondrial	SPATA19			
Q9UIV8	Serpin B13	SERPINB13			
Q9UN79	Transcription factor SOX-13	SOX13			
Q96DU3	SLAM family member 6	SLAMF6			
P02549	Spectrin alpha chain, erythrocytic	SPTA1			
Q15005	Signal peptidase complex subunit 2	SPCS2			
Q9Y2M2	Protein SSUH2 homolog	SSUH2			
Q8TE77	Protein phosphatase Slingshot homolog 3	SSH3			
Q7RTT3	Putative protein SSX9	SSX9P			
Q08ET2	Sialic acid-binding Ig-like lectin	SIGLEC14			
P49888	Sulfotransferase 1E1	SULT1E1			
O95772	STARD3 N-terminal-like protein	STARD3NL			

P51692	Signal transducer and activator of transcription 5B	STAT5B			
Q96MF2	SH3 and cysteine-rich domain-containing protein 3	STAC3			
Q6NVH7	ATPase SWSAP1	SWSAP1			
Q86TI0	TBC1 domain family member 1	TBC1D1			
Q96SF7	T-box transcription factor TBX15	TBX15			
B6A8C7	T-cell-interacting, activating receptor on myeloid cells protein 1	TARM1			
Q9BXR5	Toll-like receptor 10	TLR10			
P10646	Tissue factor pathway inhibitor	TFPI			
Q9NR97	Toll-like receptor 8	TLR8			
Q8IW70	Transmembrane protein 151B	TMEM151B			
O14948	Transcription factor EC	TFEC			
Q6B0B8	Tigger transposable element-derived protein 3	TIGD3			
P02787	Serotransferrin	TF			
Q14134	Tripartite motif-containing protein 29	TRIM29			
Q13049	E3 ubiquitin-protein ligase	TRIM32			
A0A075B6S0	T cell receptor gamma joining 1	TRGJ1			
Q8IUR5	Protein O-mannosyl-transferase TMTC1	TMTC1			
Q9H0E2	Toll-interacting protein	TOLLIP			
P0CI25	Tripartite motif-containing protein 49	TRIM49			
O43399	Tumor protein D54	TPD52L2			
Q59H18	Serine/threonine-protein kinase TNNI3K	TNNI3K			
A0A075B6U4	T cell receptor alpha variable 7	TRAV7			
Q9HBA0	Transient receptor potential cation channel subfamily V member 4	TRPV4			
A0A589	T cell receptor beta variable 4-3	TRBV4-3			
A0A0B4J24	T cell receptor alpha variable 10	TRAV10			
Q03405	Urokinase plasminogen activator surface receptor	PLAUR			
Q2NL82	Pre-rRNA-processing protein TSR1 homolog	TSR1			
P49638	Alpha-tocopherol transfer protein	TTPA			
P07477	Trypsin-1	PRSS1			
Q9NVA1	Ubiquinol-cytochrome-c reductase complex assembly factor 1	UQCC1			
Q9NZI7	Upstream-binding protein 1	UBP1			
A6NDN8	Putative ubiquitin-like protein FUBI-like protein ENSP00000310146				
Q9NZ09	Ubiquitin-associated protein 1	UBAP1			
Q70J99	Protein unc-13 homolog D	UNC13D			
Q16560	U11/U12 small nuclear ribonucleoprotein 35 kDa protein	SNRNP35			
Q8NB14	Ubiquitin carboxyl-terminal hydrolase 38	USP38			
Q8TED0	U3 small nucleolar RNA-associated protein 15 homolog	UTP15			
Q13415	Origin recognition complex subunit 1	ORC1			
Q8NGR8	Olfactory receptor 1L8	OR1L8			
Q8NGZ6	Olfactory receptor 6F1	OR6F1			
Q96FW1	Ubiquitin thioesterase OTUB1	OTUB1			
Q99983	Osteomodulin	OMD			
Q9BSM1	Polycomb group RING finger protein 1	PCGF1			
Q08174	Protocadherin-1	PCDH1			
A6NGQ2	Oocyte-expressed protein homolog	OOEP			
P04637	Cellular tumor antigen p53	TP53			

Q8N328	PiggyBac transposable element-derived protein 3	PGBD3			
Q96NR3	Patched domain-containing protein 1	PTCHD1			
Q16825	Tyrosine-protein phosphatase non-receptor type 21	PTPN21			
O43502	DNA repair protein RAD51 homolog 3	RAD51C			
P22695	Cytochrome b-c1 complex subunit 2, mitochondrial	UQCRC2			
Q8TDP1	Ribonuclease H2 subunit C	RNASEH2C			
Q96LT9	RNA-binding region-containing protein 3	RNPC3			
Q9BXT8	RING finger protein 17	RNF17			
A6NIN4	RING finger protein 227	RNF227			
P62249	40S ribosomal protein S16	RPS16			
Q17RH7	Putative protein TPRXL	TPRXL			
Q6IMI4	Sulfotransferase 6B1	SULT6B1			
Q9ULQ0	Striatin-interacting protein 2	STRIP2			
P08240	Signal recognition particle receptor subunit alpha	SRPRA			
Q687X5	Metalloreductase STEAP4	STEAP4			
P0CV98	Testis-specific Y-encoded protein 3	TSPY3			
Q15527	Surfeit locus protein 2	SURF2			
P61764	Syntaxin-binding protein 1	STXBP1			
O15042	U2 snRNP-associated SURP motif-containing protein	U2SURP			
Q4G0T1	Scavenger receptor cysteine-rich domain-containing protein SCART1				
Q8NFB2	Transmembrane protein 185A	TMEM185A			
Q9NYW3	Taste receptor type 2 member 7	TAS2R7			
Q02763	Angiopoietin-1 receptor	TEK			
Q96H15	T-cell immunoglobulin and mucin domain-containing protein 4	TIMD4			
P21579	Synaptotagmin-1	SYT1			
P59537	Taste receptor type 2 member 43	TAS2R43			
Q96CG3	TRAF-interacting protein with FHA domain-containing protein A	TIFA			
P68363	Tubulin alpha-1B chain	TUBA1B			
Q99595	Mitochondrial import inner membrane translocase subunit Tim17-A	TIMM17A			
Q9BVV7	Mitochondrial import inner membrane translocase subunit Tim21	TIMM21			
A0A075B6Y9	T cell receptor alpha joining 42	TRAJ42			
Q9NV12	Transmembrane protein 140	TMEM140			
A0PJX2	TLD domain-containing protein 2	TLDC2			
Q86YL5	Testis development-related protein	TDRP			
Q13569	G/T mismatch-specific thymine DNA glycosylase	TDG			
Q9BZW4	Transmembrane 6 superfamily member 2	TM6SF2			
Q8N661	Lysoplasmalogenase	TMEM86B			
Q9BZW5	Transmembrane 6 superfamily member 1	TM6SF1			
Q96PL2	Beta-tectorin	TECTB			
O15417	Trinucleotide repeat-containing gene 18 protein	TNRC18			
Q03169	Tumor necrosis factor alpha-induced protein 2	TNFAIP2			
Q9BVG3	E3 ubiquitin-protein ligase	TRIM62			
A0A5B9	T cell receptor beta constant 2	TRBC2			
Q6ZXV5	Protein O-mannosyl-transferase TMTC3	TMTC3			

P50591	Tumor necrosis factor ligand superfamily member 10	TNFSF10			
Q86TZ1	Tetratricopeptide repeat protein 6	TTC6			
Q86VY4	Testis-specific Y-encoded-like protein 5	TSPYL5			
A0A0A6YYC5	T cell receptor alpha variable 14/delta variable 4	TRAV14DV4			
O60637	Tetraspanin-3	TSPAN3			
A0A087WV62	T cell receptor beta variable 16	TRBV16			
Q86TN4	tRNA 2'-phosphotransferase 1	TRPT1			
Q9BZ97	Putative transcript Y 13 protein	TTTY13			
E5RIL1	Uroplakin-3b-like protein 2	UPK3BL2			
Q05086	Ubiquitin-protein ligase E3A	UBE3A			
Q9UK80	Ubiquitin carboxyl-terminal hydrolase 21	USP21			
Q8TBC4	NEDD8-activating enzyme E1 catalytic subunit	UBA3			
Q969T4	Ubiquitin-conjugating enzyme E2 E3	UBE2E3			
Q6EMK4	Vasorin	VASN			
O75191	Xylulose kinase	XYLB			
Q2TBF2	WSC domain-containing protein 2	WSCD2			
P56703	Proto-oncogene Wnt-3	WNT3			
Q7Z3J2	VPS35 endosomal protein-sorting factor-like	VPS35L			
P58304	Visual system homeobox 2	VSX2			
Q15269	Periodic tryptophan protein 2 homolog	PWP2			
P15151	Poliovirus receptor	PVR			
Q6ZTQ3	Ras association domain-containing protein 6	RASSF6			
P01112	GTPase HRas	HRAS			
P62877	E3 ubiquitin-protein ligase RBX1	RBX1			
Q8NC24	RELT-like protein 2	RELL2			
Q3YEC7	Rab-like protein 6	RABL6			
O95755	Ras-related protein Rab-36	RAB36			
Q9BTD8	RNA-binding protein 42	RBM42			
Q13637	Ras-related protein Rab-32	RAB32			
Q9NZL6	Ral guanine nucleotide dissociation stimulator-like 1	RGL1			
Q7L804	Rab11 family-interacting protein 2	RAB11FIP2			
Q9UGC6	Regulator of G-protein signaling 17	RGS17			
Q6R327	Rapamycin-insensitive companion of mTOR	RICTOR			
Q96AA3	Protein RFT1 homolog	RFT1			
Q96K30	RBPJ-interacting and tubulin-associated protein 1	RITA1			
O95707	Ribonuclease P protein subunit p29	POP4			
O95059	Ribonuclease P protein subunit p14	RPP14			
Q9BRL7	Vesicle-trafficking protein SEC22c	SEC22C			
Q9H115	Beta-soluble NSF attachment protein	NAPB			
Q8WW59	SPRY domain-containing protein	SPRYD4			
O95347	Structural maintenance of chromosomes protein 2	SMC2			
O43623	Zinc finger protein SNAI2	SNAI2			
Q9Y5W9	Sorting nexin-11	SNX11			
Q8NCJ5	SPRY domain-containing protein	SPRYD3			
Q99835	Smoothened homolog	SMO			
Q8TAD8	Smad nuclear-interacting protein 1	SNIP1			
Q6ZMY3	SPOC domain-containing protein 1	SPOCD1			
A0A1B0GVT2	Small integral membrane protein 36	SMIM36			

P51532	Transcription activator BRG1	SMARCA4			
P35270	Sepiapterin reductase	SPR			
Q9Y6E7	NAD-dependent protein lipoamidase sirtuin-4, mitochondrial	SIRT4			
O95238	SAM pointed domain-containing Ets transcription factor	SPDEF			
Q9NTG7	NAD-dependent protein deacetylase sirtuin-3, mitochondrial	SIRT3			
P23497	Nuclear autoantigen Sp-100	SP100			
Q13239	Src-like-adapter	SLA			
Q8N114	Protein shisa-5	SHISA5			
O43291	Kunitz-type protease inhibitor 2	SPINT2			
Q8IZP2	Putative protein FAM10A4	ST13P4			
A8MT33	Synaptonemal complex central element protein 1-like	SYCE1L			
A6NL88	Protein shisa-7	SHISA7			
Q9H0A9	Speriolin-like protein	SPATC1L			
Q9H169	Stathmin-4	STMN4			
O75897	Sulfotransferase 1C4	SULT1C4			
A1L190	Synaptonemal complex central element protein 3	SYCE3			
P0CL85	STAG3-like protein 3	STAG3L3			
Q58G82	Putative synaptotagmin-14-like protein	SYT14P1			
Q9P2W9	Syntaxin-18	STX18			
A0A0B4J24	T cell receptor alpha variable 13-1	TRAV13-1			
Q8N4C7	Syntaxin-19	STX19			
P61266	Syntaxin-1B	STX1B			
P10124	Serglycin	SRGN			
P30872	Somatostatin receptor type 1	SSTR1			
Q12846	Syntaxin-4	STX4			
P55854	Small ubiquitin-related modifier 3	SUMO3			
B3KS81	Serine/arginine repetitive matrix protein 5	SRRM5			
P43308	Translocon-associated protein subunit beta	SSR2			
Q6J9G0	Tyrosine-protein kinase STYK1	STYK1			
Q96LR4	Chemokine-like protein TAFA-4	TAFA4			
B2RXF0	Transmembrane protein 229A	TMEM229A			
Q9Y5Q8	General transcription factor 3C polypeptide 5	GTF3C5			
O75663	TIP41-like protein	TIPRL			
Q6IEE7	Transmembrane protein 132E	TMEM132E			
Q3ZCQ8	Mitochondrial import inner membrane translocase subunit TIM50	TIMM50			
Q07352	mRNA decay activator protein ZFP36L1	ZFP36L1			
A0A1B0GV G6	Testis-expressed protein 54	TEX54			
Q9NQB0	Transcription factor 7-like 2	TCF7L2			
Q8IVF5	T-lymphoma invasion and metastasis-inducing protein 2	TIAM2			
Q8WUH6	Transmembrane protein 263	TMEM263			
P13726	Tissue factor	F3			
O75674	TOM1-like protein 1	TOM1L1			
Q8WW34	Transmembrane protein 239	TMEM239			
O95455	dTDP-D-glucose 4,6-dehydratase	TGDS			
Q53S58	Transmembrane protein 177	TMEM177			
Q9BSA9	Endosomal/lysosomal potassium channel TMEM175	TMEM175			
Q6UXN2	Trem-like transcript 4 protein	TREML4			
Q9NQ86	E3 ubiquitin-protein ligase	TRIM36			
Q5T4D3	Protein O-mannosyl-transferase TMTC4	TMTC4			
Q7Z410	Transmembrane protease serine 9	TMPRSS9			
Q96GM8	Target of EGR1 protein 1	TOE1			
Q8N0Z6	Tetratricopeptide repeat protein 5	TTC5			

Q6ZSZ6	Teashirt homolog 1	TSHZ1			
P48995	Short transient receptor potential channel 1	TRPC1			
A0A0A0MS03	Probable non-functional T cell receptor beta variable 5-3	TRBV5-3			
A0A1B0GX95	T cell receptor beta variable 7-4	TRBV7-4			
A0A584	T cell receptor beta variable 11-2	TRBV11-2			
Q9Y2K6	Ubiquitin carboxyl-terminal hydrolase 20	USP20			
P0CB47	Upstream-binding factor 1-like protein 1	UBTFL1			
D6R901	Ubiquitin carboxyl-terminal hydrolase 17-like protein 21	USP17L21			
P25874	Mitochondrial brown fat uncoupling protein 1	UCP1			
Q6R6M4	Ubiquitin carboxyl-terminal hydrolase 17	USP17L2			
Q69YN4	Protein virilizer homolog	VIRMA			
B2RUY7	von Willebrand factor C domain-containing protein 2-like	VWC2L			
Q8N752	Casein kinase I isoform alpha-like	CSNK1A1L			
P56470	Galectin-4	LGALS4			
Q86VQ0	Lebercilin	LCA5			
Q6B0I6	Lysine-specific demethylase 4D	KDM4D			
Q9NT99	Leucine-rich repeat-containing protein 4B	LRRC4B			
Q14392	Transforming growth factor beta activator LRRC32	LRRC32			
Q86X29	Lipolysis-stimulated lipoprotein receptor	LSR			
O75581	Low-density lipoprotein receptor-related protein 6	LRP6			
A6NDA9	Leucine-rich repeat, immunoglobulin-like domain and transmembrane domain-containing protein 2	LRIT2			
Q86VH5	Leucine-rich repeat transmembrane neuronal protein 3	LRRTM3			
Q38SD2	Leucine-rich repeat serine/threonine-protein kinase 1	LRRK1			
Q9Y234	Lipoyltransferase 1, mitochondrial	LIPT1			
P12980	Protein lyl-1	LYL1			
Q9BRT6	Protein LLP homolog	LLPH			
P83369	U7 snRNA-associated Sm-like protein LSm11	LSM11			
P43034	Platelet-activating factor acetylhydrolase IB subunit beta	PAFAH1B1			
Q9NU23	LYR motif-containing protein 2	LYRM2			
Q9UHV7	Mediator of RNA polymerase II transcription subunit 13	MED13			
P55145	Mesencephalic astrocyte-derived neurotrophic factor	MANF			
A4D2B0	Metallo-beta-lactamase domain-containing protein 1	MBLAC1			
Q969L2	Protein MAL2	MAL2			
Q9H0U3	Magnesium transporter protein 1	MAGT1			
Q7Z304	MAM domain-containing protein 2	MAMDC2			
Q13394	Putative nucleotidyltransferase MAB21L1	MAB21L1			
Q13477	Mucosal addressin cell adhesion molecule 1	MADCAM1			
P45984	Mitogen-activated protein kinase 9	MAPK9			
Q6NUT3	Major facilitator superfamily domain-containing protein 12	MFSD12			
Q13201	Multimerin-1	MMRN1			

Q96T76	MMS19 nucleotide excision repair protein homolog	MMS19			
Q5SR56	Hippocampus abundant transcript-like protein 1	MFSD14B			
Q96LA9	Mas-related G-protein coupled receptor member X4	MRGPRX4			
Q86SM8	Mas-related G-protein coupled receptor member E	MRGPRE			
P33527	Multidrug resistance-associated protein 1	ABCC1			
Q4VC12	Putative protein MSS51 homolog, mitochondrial	MSS51			
Q6P444	Mitochondrial fission regulator 2	MTFR2			
P13640	Metallothionein-1G	MT1G			
Q8NGC6	Olfactory receptor 4K17	OR4K17			
Q8NG81	Olfactory receptor 2M7	OR2M7			
Q8N628	Olfactory receptor 2C3	OR2C3			
Q8NGZ4	Olfactory receptor 2G3	OR2G3			
O14753	Putative transcription factor Ovo-like 1	OVOL1			
P22059	Oxysterol-binding protein 1	OSBP			
P49810	Presenilin-2	PSEN2			
Q96E09	PPP2R1A-PPP2R2A-interacting phosphatase regulator 1	PABIR1			
Q96PD5	N-acetylmuramoyl-L-alanine amidase	PGLYRP2			
Q9GZU8	PSME3-interacting protein	PSME3IP1			
Q9H5I5	Piezo-type mechanosensitive ion channel component 2	PIEZO2			
Q8NA58	Poly	PNLDC1			
Q9Y4D7	Plexin-D1	PLXND1			
Q9HAT8	E3 ubiquitin-protein ligase pellino homolog 2	PELI2			
Q03431	Parathyroid hormone/parathyroid hormone-related peptide receptor	PTH1R			
Q9HD43	Receptor-type tyrosine-protein phosphatase H	PTPRH			
Q6NVV1	Putative 60S ribosomal protein L13a protein RPL13AP3	RPL13AP3			
Q8WU10	Pyridine nucleotide-disulfide oxidoreductase domain-containing protein 1	PYROXD1			
Q96N64	PWWP domain-containing protein 2A	PWWP2A			
Q2KHR3	Glutamine and serine-rich protein 1	QSER1			
Q9BRX8	Peroxiredoxin-like 2A	PRXL2A			
P50749	Ras association domain-containing protein 2	RASSF2			
C9J798	Ras GTPase-activating protein 4B	RASA4B			
Q9NR77	Peroxisomal membrane protein 2	PXMP2			
Q8IZ41	Ras and EF-hand domain-containing protein	RASEF			
Q9UBF6	RING-box protein 2	RNF7			
P61571	Endogenous retrovirus group K member 21 Rec protein	ERVK-21			
Q9Y388	RNA-binding motif protein, X-linked 2	RBMX2			
Q8NDN9	RCC1 and BTB domain-containing protein 1	RCBTB1			
O60896	Receptor activity-modifying protein 3	RAMP3			
Q9NP90	Ras-related protein Rab-9B	RAB9B			
Q15042	Rab3 GTPase-activating protein catalytic subunit	RAB3GAP1			
Q5T481	RNA-binding protein 20	RBM20			
Q6ZTI6	Refilin-A	RFLNA			

Q2PPJ7	Ral GTPase-activating protein subunit alpha-2	RALGAPA2			
Q6PJF5	Inactive rhomboid protein 2	RHBDF2			
O76081	Regulator of G-protein signaling 20	RGS20			
P58872	Rhomboid-related protein 3	RHBDL3			
Q969G6	Riboflavin kinase	RFK			
P02753	Retinol-binding protein 4	RBP4			
P62913	60S ribosomal protein L11	RPL11			
O95948	One cut domain family member 2	ONECUT2			
O95221	Olfactory receptor 5F1	OR5F1			
Q96RA2	Olfactory receptor 7D2	OR7D2			
Q8NGL0	Olfactory receptor 5L2	OR5L2			
A0A075B759	Peptidyl-prolyl cis-trans isomerase A-like 4E	PPIAL4E			
Q16654		PDK4			
Q9H477	Ribokinase	RBKS			
Q9BUL8	Programmed cell death protein 10	PDCD10			
C9JFL3	Proline, histidine and glycine-rich protein 1	PHGR1			
Q96PV4	Paraneoplastic antigen-like protein 5	PNMA5			
O14829	Serine/threonine-protein phosphatase with EF-hands 1	PPEF1			
Q8NA72	Centrosomal protein POC5	POC5			
Q6P4A8	Phospholipase B-like 1	PLBD1			
Q5T870	Proline-rich protein 9	PRR9			
Q8N945	PRELI domain-containing protein 2	PRELID2			
P78337	Pituitary homeobox 1	PITX1			
Q53GL0	Pleckstrin homology domain-containing family O member 1	PLEKHO1			
P25789	Proteasome subunit alpha type-4	PSMA4			
Q2VWP7	Protogenin	PRTG			
Q7RTY3	Putative serine protease 45	PRSS45P			
Q9HC23	Prokineticin-2	PROK2			
Q9Y2Y8	Proteoglycan 3	PRG3			
Q9HAZ2	Histone-lysine N-methyltransferase PRDM16	PRDM16			
Q86YN6	Peroxisome proliferator-activated receptor gamma coactivator 1-beta	PPARGC1B			
P48556	26S proteasome non-ATPase regulatory subunit 8	PSMD8			
Q9Y3E5	Peptidyl-tRNA hydrolase 2, mitochondrial	PTRH2			
Q15256	Receptor-type tyrosine-protein phosphatase R	PTPRR			
Q9Y3Y4	Pygopus homolog 1	PYGO1			
P07919	Cytochrome b-c1 complex subunit 6, mitochondrial	UQCRH			
Q8NBN7	Retinol dehydrogenase 13	RDH13			
P0C7P1	RNA-binding motif protein, Y chromosome, family 1 member D	RBMY1D			
P61573	Endogenous retrovirus group K member 9 Rec protein	ERVK-9			
P04049	RAF proto-oncogene serine/threonine-protein kinase	RAF1			
Q9H1K0	Rabenosyn-5	RBSN			
Q13636	Ras-related protein Rab-31	RAB31			
O75628	GTP-binding protein REM 1	REM1			
Q9H082	Ras-related protein Rab-33B	RAB33B			
Q9BZG1	Ras-related protein Rab-34	RAB34			
Q9BX46	RNA-binding protein 24	RBM24			
Q8IWW6	Rho GTPase-activating protein 12	ARHGAP12			
Q07960	Rho GTPase-activating protein 1	ARHGAP1			
P27694	Replication protein A 70 kDa DNA-binding subunit	RPA1			

Q3KRB8	Rho GTPase-activating protein 11B	ARHGAP11B			
A6NLU0	Ret finger protein-like 4A	RFPL4A			
P49758	Regulator of G-protein signaling 6	RGS6			
P40937	Replication factor C subunit 5	RFC5			
Q5JTH9	RRP12-like protein	RRP12			
P46783	40S ribosomal protein S10	RPS10			
P62269	40S ribosomal protein S18	RPS18			
Q9HA92	Radical S-adenosyl methionine domain-containing protein 1, mitochondrial	RSAD1			
P62244	40S ribosomal protein S15a	RPS15A			
P46781	40S ribosomal protein S9	RPS9			
Q8TD47	40S ribosomal protein S4, Y isoform 2	RPS4Y2			
Q6NW29	RWD domain-containing protein	RWDD4			
Q9Y2R5	28S ribosomal protein S17, mitochondrial	MRPS17			
A6NK97	Solute carrier family 22 member 20	SLC22A20P			
Q9BZD2	Equilibrative nucleoside transporter 3	SLC29A3			
Q9Y3D9	28S ribosomal protein S23, mitochondrial	MRPS23			
Q86UD0	Suppressor APC domain-containing protein 2	SAPCD2			
P0DMR2	Secretoglobin family 1C member	SCGB1C2			
O15357	Phosphatidylinositol 3,4,5-trisphosphate 5-phosphatase 2	INPPL1			
P31645	Sodium-dependent serotonin transporter	SLC6A4			
O15127	Secretory carrier-associated membrane protein 2	SCAMP2			
Q8IWY4	Signal peptide, CUB and EGF-like domain-containing protein 1	SCUBE1			
Q9UBC9	Small proline-rich protein 3	SPRR3			
Q13487	snRNA-activating protein complex subunit 2	SNAPC2			
O95343	Homeobox protein SIX3	SIX3			
Q9H0W8	Protein SMG9	SMG9			
O43541	Mothers against decapentaplegic homolog 6	SMAD6			
Q3ZLR7	Transcription factor SPT20 homolog-like 1	SUPT20HL1			
Q16637	Survival motor neuron protein	SMN1			
P62318	Small nuclear ribonucleoprotein Sm D3	SNRPD3			
Q9BPZ7	Target of rapamycin complex 2 subunit MAPKAP1	MAPKAP1			
O15370	Transcription factor SOX-12	SOX12			
Q8ND83	SLAIN motif-containing protein 1	SLAIN1			
Q15532	Protein SSXT	SS18			
Q8TCY0	Small integral membrane protein 11B	SMIM11B			
Q5T4T6	Synaptonemal complex protein 2-like	SYCP2L			
P82094	TATA element modulatory factor	TMF1			
P59095	StAR-related lipid transfer protein 6	STARD6			
Q96SI9	Spermatid perinuclear RNA-binding protein	STRBP			
P09132	Signal recognition particle 19 kDa protein	SRP19			
Q13242	Serine/arginine-rich splicing factor 9	SRSF9			
P23327	Sarcoplasmic reticulum histidine-rich calcium-binding protein	HRC			
Q96RI8	Trace amine-associated receptor 6	TAAR6			

Q9Y6J9	TAF6-like RNA polymerase II p300/CBP-associated factor-associated factor 65 kDa subunit 6L	TAF6L			
O75764	Transcription elongation factor A protein 3	TCEA3			
P50990	T-complex protein 1 subunit theta	CCT8			
Q86UD7	TBC1 domain family member 26	TBC1D26			
Q9C0C2	182 kDa tankyrase-1-binding protein	TNKS1BP1			
Q6YHU6	Thyroid adenoma-associated protein	THADA			
P59543	Taste receptor type 2 member 20	TAS2R20			
P62987	Ubiquitin-60S ribosomal protein L40	UBA52			
P09651	Heterogeneous nuclear ribonucleoprotein A1	HNRNPA1			
O14802	DNA-directed RNA polymerase III subunit RPC1	POLR3A			
Q93091	Ribonuclease K6	RNASE6			
Q96KN7	X-linked retinitis pigmentosa GTPase regulator-interacting protein 1	RPGRIP1			
Q8TAA1	Probable ribonuclease 11	RNASE11			
Q8N122	Regulatory-associated protein of mTOR	RPTOR			
Q6PCB5	Lysine-specific demethylase RSBN1L	RSBN1L			
P62753	40S ribosomal protein S6	RPS6			
Q5TD94	Radial spoke head protein 4 homolog A	RSPH4A			
Q9Y694	Solute carrier family 22 member 7	SLC22A7			
Q96CQ1	Solute carrier family 25 member 36	SLC25A36			
Q8TE54	Anion exchange transporter	SLC26A7			
Q8IXA5	Sperm acrosome membrane-associated protein 3	SPACA3			
Q9NZJ4	Sacsin	SACS			
O14975	Very long-chain acyl-CoA synthetase	SLC27A2			
Q9BY42	Replication termination factor 2	RTF2			
Q13621	Solute carrier family 12 member 1	SLC12A1			
Q9BST9	Rhotekin	RTKN			
P55017	Solute carrier family 12 member 3	SLC12A3			
O76054	SEC14-like protein 2	SEC14L2			
P62304	Small nuclear ribonucleoprotein E	SNRPE			
Q8IY34	Solute carrier family 15 member 3	SLC15A3			
Q9Y265	RuvB-like 1	RUVBL1			
P41440	Reduced folate transporter	SLC19A1			
Q9BUV0	Arginine/serine-rich protein 1	RSRP1			
Q8WYR4	Radial spoke head 1 homolog	RSPH1			
P48066	Sodium- and chloride-dependent GABA transporter 3	SLC6A11			
Q9GZN6	Orphan sodium- and chloride-dependent neurotransmitter transporter NTT5	SLC6A16			
Q9H228	Sphingosine 1-phosphate receptor 5	S1PR5			
P0DJI9	Serum amyloid A-2 protein	SAA2			
Q08357	Sodium-dependent phosphate transporter 2	SLC20A2			
Q9H015	Solute carrier family 22 member 4	SLC22A4			
O75298	Reticulon-2	RTN2			
Q96LT4	Sphingomyelin synthase-related protein 1	SAMD8			
O75446	Histone deacetylase complex subunit SAP30	SAP30			
Q8NE22	SET domain-containing protein 9	SETD9			
Q9BWJ5	Splicing factor 3B subunit 5	SF3B5			

A0A286YF46	Small cysteine and glycine repeat-containing protein 5	SCYGR5			
Q5TG53	Putative uncharacterized protein SERTAD4-AS1	SERTAD4-AS1			
Q99961	Endophilin-A2	SH3GL1			
Q5TEA6	Protein sel-1 homolog 2	SEL1L2			
Q9UQQ2	SH2B adapter protein 3	SH2B3			
Q96I15	Selenocysteine lyase	SCLY			
Q9P0V3	SH3 domain-binding protein 4	SH3BP4			
Q8NEM2	SHC SH2 domain-binding protein 1	SHCBP1			
Q9Y6X1	Stress-associated endoplasmic reticulum protein 1	SERP1			
Q96KG9	N-terminal kinase-like protein	SCYL1			
P58004	Sestrin-2	SESN2			
Q12884	Prolyl endopeptidase FAP	FAP			
P34741	Syndecan-2	SDC2			
O76038	Secretagogin	SCGN			
Q8N4B1	Sesquipedalian-1	PHETA1			
P04279	Semenogelin-1	SEMG1			
Q8NCE0	tRNA-splicing endonuclease subunit Sen2	TSEN2			
Q52WX2	Serine/threonine-protein kinase SBK1	SBK1			
Q5BIV9	Shadow of prion protein	SPRN			
O75711	Scrapie-responsive protein 1	SCRG1			
B1AK76	Putative SNURF-like protein	SNURFL			
P62314	Small nuclear ribonucleoprotein Sm D1	SNRPD1			
Q9UIU6	Homeobox protein SIX4	SIX4			
A6NGZ8	Small integral membrane protein	SMIM9			
Q8NHU3	Phosphatidylcholine:ceramide cholinephosphotransferase 2	SGMS2			
Q9HD40	O-phosphoseryl-tRNA	SEPSECS			
Q8NFR3	Serine palmitoyltransferase small subunit B	SPTSSB			
A0A494C086	Putative speedy protein E21	SPDYE21			
P35712	Transcription factor SOX-6	SOX6			
Q06945	Transcription factor SOX-4	SOX4			
Q9P0V8	SLAM family member 8	SLAMF8			
P29508	Serpin B3	SERPINB3			
O95149	Snurportin-1	SNUPN			
P19623	Spermidine synthase	SRM			
Q07837	Neutral and basic amino acid transport protein rBAT	SLC3A1			
Q9ULL8	Protein Shroom4	SHROOM4			
O15266	Short stature homeobox protein	SHOX			
O00338	Sulfotransferase 1C2	SULT1C2			
Q6PEY1	Transmembrane protein 88	TMEM88			
Q99523	Sortilin	SORT1			
Q8NE28	Serine/threonine kinase-like domain-containing protein STKLD1	STKLD1			
P78362	SRSF protein kinase 2	SRPK2			
P78539	Sushi repeat-containing protein SRPX	SRPX			
P51571	Translocon-associated protein subunit delta	SSR4			
Q8WXA9	Splicing regulatory glutamine/lysine-rich protein 1	SREK1			
A2A2V5	Serine-rich and transmembrane domain-containing protein 1	SERTM1			
Q9NUY8	TBC1 domain family member 23	TBC1D23			
Q96EI5	Transcription elongation factor A protein-like 4	TCEAL4			
Q9UJT0	Tubulin epsilon chain	TUBE1			
Q96DN5	TBC1 domain family member 31	TBC1D31			
P0C7X1	TBC1 domain family member 3H	TBC1D3H			
Q1AE95	Transmembrane protein 183B	TMEM183B			

Q9Y2B9	cAMP-dependent protein kinase inhibitor gamma	PKIG			
Q9NVM9	Integrator complex subunit 13	INTS13			
Q2VIQ3	Chromosome-associated kinesin KIF4B	KIF4B			
Q13241	Natural killer cells antigen CD94	KLRD1			
Q92806	G protein-activated inward rectifier potassium channel 3	KCNJ9			
A1A580	Keratin-associated protein 23-1	KRTAP23-1			
Q6L8G5	Keratin-associated protein 5-10	KRTAP5-10			
Q3LI58	Keratin-associated protein 21-1	KRTAP21-1			
Q8N743	Killer cell immunoglobulin-like receptor 3DL3	KIR3DL3			
P23443	Ribosomal protein S6 kinase beta-1	RPS6KB1			
O75449	Katanin p60 ATPase-containing subunit A1	KATNA1			
P24723	Protein kinase C eta type	PRKCH			
O95751	Protein LDOC1	LDOC1			
Q16288	NT-3 growth factor receptor	NTRK3			
Q8NGC2	Olfactory receptor 4E2	OR4E2			
P61457	Pterin-4-alpha-carbinolamine dehydratase	PCBD1			
Q9UBZ9	DNA repair protein REV1	REV1			
P57078	Receptor-interacting serine/threonine-protein kinase 4	RIPK4			
P62899	60S ribosomal protein L31	RPL31			
Q6ZNA4	E3 ubiquitin-protein ligase	RNF111			
Q6P1L8	39S ribosomal protein L14, mitochondrial	MRPL14			
Q99942	E3 ubiquitin-protein ligase RNF5	RNF5			
Q96FB5	Protein RRNAD1	RRNAD1			
P0DKV0	Putative spermatogenesis-associated protein 31C1	SPATA31C1			
P0CK96	Solute carrier family 35 member E2B	SLC35E2B			
Q9Y267	Solute carrier family 22 member 14	SLC22A14			
Q5T1Q4	Solute carrier family 35 member	SLC35F1			
Q6ZQQ2	Spermatogenesis-associated protein 31D1	SPATA31D1			
A4IF30	Solute carrier family 35 member	SLC35F4			
Q6NUJ1	Proactivator polypeptide-like 1	PSAPL1			
Q9BYC2	Succinyl-CoA:3-ketoacid coenzyme A transferase 2, mitochondrial	OXCT2			
Q9NRP4	Succinate dehydrogenase assembly factor 3, mitochondrial	SDHAF3			
Q6ZNX1	Shieldin complex subunit 3	SHLD3			
Q9NRF2	SH2B adapter protein 1	SH2B1			
Q9BX95	Sphingosine-1-phosphate phosphatase 1	SGPP1			
Q5BJF2	Sigma intracellular receptor 2	TMEM97			
Q13591	Semaphorin-5A	SEMA5A			
Q9H299	SH3 domain-binding glutamic acid-rich-like protein 3	SH3BGRL3			
P35498	Sodium channel protein type 1 subunit alpha	SCN1A			
Q8IWL2	Pulmonary surfactant-associated protein A1	SFTPA1			
Q8IWL1	Pulmonary surfactant-associated protein A2	SFTPA2			
P50454	Serpin H1	SERPINH1			
P53794	Sodium/myo-inositol cotransporter	SLC5A3			
Q9HC62	Sentrin-specific protease 2	SENP2			
Q9Y6X3	MAU2 chromatid cohesion factor homolog	MAU2			

Q9UQD0	Sodium channel protein type 8 subunit alpha	SCN8A			
P05060	Secretogranin-1	CHGB			
Q9NRH2	SNF-related serine/threonine-protein kinase	SNRK			
Q8WVI0	Small integral membrane protein	SMIM4			
P0DMW4	Small integral membrane protein 10-like protein 2A	SMIM10L2A			
Q9UMY4	Sorting nexin-12	SNX12			
Q8IVB4	Sodium/hydrogen exchanger 9	SLC9A9			
Q6STE5	SWI/SNF-related matrix-associated actin-dependent regulator of chromatin subfamily D member 3	SMARCD3			
Q96BD0	Solute carrier organic anion transporter family member 4A1	SLCO4A1			
O94933	SLIT and NTRK-like protein 3	SLITRK3			
P08294	Extracellular superoxide dismutase	SOD3			
O75093	Slit homolog 1 protein	SLIT1			
Q96L03	Spermatogenesis-associated protein 17	SPATA17			
Q08AE8	Protein spire homolog 1	SPIRE1			
Q96A28	SLAM family member 9	SLAMF9			
O15269	Serine palmitoyltransferase 1	SPTLC1			
Q9H0F6	Sharpin	SHARPIN			
Q96PQ1	Sialic acid-binding Ig-like lectin	SIGLEC12			
O15270	Serine palmitoyltransferase 2	SPTLC2			
P15907	Beta-galactoside alpha-2,6-sialyltransferase 1	ST6GAL1			
Q9H4F1	Alpha-N-acetyl-neuraminyl-2,3-beta-galactosyl-1,3-N-acetyl-galactosaminide alpha-2,6-sialyltransferase	ST6GALNAC4			
O94864	STAGA complex 65 subunit gamma	SUPT7L			
Q9NY57	Serine/threonine-protein kinase 32B	STK32B			
P46977	Dolichyl-diphosphooligosaccharide--protein glycosyltransferase subunit STT3A	STT3A			
P56962	Syntaxin-17	STX17			
Q86Y82	Syntaxin-12	STX12			
P61011	Signal recognition particle 54 kDa protein	SRP54			
Q06330	Recombining binding protein suppressor of hairless	RBPJ			
Q16629	Serine/arginine-rich splicing factor 7	SRSF7			
Q15046	Lysine--tRNA ligase	KARS1			
O43776	Asparagine--tRNA ligase, cytoplasmic	NARS1			
O95359	Transforming acidic coiled-coil-containing protein 2	TACC2			
Q9NYS0	NF-kappa-B inhibitor-interacting Ras-like protein 1	NKIRAS1			
Q13303	Voltage-gated potassium channel subunit beta-2	KCNAB2			
A0A075B6H7	Probable non-functional immunoglobulin kappa variable 3-7	IGKV3-7			
Q9HBH9	MAP kinase-interacting serine/threonine-protein kinase 2	MKNK2			
Q5VZ52	MORN repeat-containing protein 5	MORN5			
Q8NDC4	MORN repeat-containing protein 4	MORN4			
O15427	Monocarboxylate transporter 4	SLC16A3			

Q96J66	ATP-binding cassette sub-family C member 11	ABCC11			
Q96DY7	Mdm2-binding protein	MTBP			
P43121	Cell surface glycoprotein MUC18	MCAM			
Q9HDB5	Neurexin-3-beta	NRXN3			
Q14980	Nuclear mitotic apparatus protein 1	NUMA1			
Q8NGX9	Olfactory receptor 6P1	OR6P1			
Q7Z3H0	Photoreceptor ankyrin repeat protein	ANKRD33			
Q9Y5H4	Protocadherin gamma-A1	PCDHGA1			
Q9Y5G7	Protocadherin gamma-A6	PCDHGA6			
Q06710	Paired box protein Pax-8	PAX8			
P20142	Gastricsin	PGC			
O75594	Peptidoglycan recognition protein 1	PGLYRP1			
Q9UPV7	PHD finger protein 24	PHF24			
Q04671	P protein	OCA2			
P35244	Replication protein A 14 kDa subunit	RPA3			
Q9Y572	Receptor-interacting serine/threonine-protein kinase 3	RIPK3			
P52815	39S ribosomal protein L12, mitochondrial	MRPL12			
Q96A35	39S ribosomal protein L24, mitochondrial	MRPL24			
P62487	DNA-directed RNA polymerase II subunit RPB7	POLR2G			
P19387	DNA-directed RNA polymerase II subunit RPB3	POLR2C			
Q9GZN7	Protein rogdi homolog	ROGDI			
Q16518	Retinoid isomerohydrolase	RPE65			
P49247	Ribose-5-phosphate isomerase	RPIA			
O60518	Ran-binding protein 6	RANBP6			
Q96A37	E3 ubiquitin-protein ligase	RNF166			
Q96BH1	E3 ubiquitin-protein ligase RNF25	RNF25			
Q96D59	E3 ubiquitin-protein ligase	RNF183			
Q9Y3A4	Ribosomal RNA-processing protein 7 homolog A	RRP7A			
Q53EL9	Seizure protein 6 homolog	SEZ6			
Q99985	Semaphorin-3C	SEMA3C			
Q9Y2Z0	Protein SGT1 homolog	SUGT1			
Q13275	Semaphorin-3F	SEMA3F			
Q6ZPB5	Stress-responsive DNAJB4-interacting membrane protein 1	SDIM1			
O75533	Splicing factor 3B subunit 1	SF3B1			
Q8WWX9	Selenoprotein M	SELENOM			
Q9BTD1	Putative uncharacterized protein SHANK2-AS3	SHANK2-AS3			
P58005	Sestrin-3	SESN3			
O75056	Syndecan-3	SDC3			
Q6IQ49	Replication stress response regulator SDE2	SDE2			
Q8WW01	tRNA-splicing endonuclease subunit Sen15	TSEN15			
Q14160	Protein scribble homolog	SCRIB			
Q99747	Gamma-soluble NSF attachment protein	NAPG			
Q5VX52	Spermatogenesis-associated protein 1	SPATA1			
O15198	Mothers against decapentaplegic homolog 9	SMAD9			
Q6IQ16	Speckle-type POZ protein-like	SPOPL			
Q9BZL3	Small integral membrane protein	SMIM3			
Q9Y2K2	Serine/threonine-protein kinase SIK3	SIK3			
Q99954	Submaxillary gland androgen-regulated protein 3A	SMR3A			

Q9Y6L6	Solute carrier organic anion transporter family member 1B1	SLCO1B1			
O94813	Slit homolog 2 protein	SLIT2			
O14544	Suppressor of cytokine signaling 6	SOCS6			
O15304	Apoptosis regulatory protein Siva	SIVA1			
Q5MJ09	Sperm protein associated with the nucleus on the X chromosome N3	SPANXN3			
O43173	Sia-alpha-2,3-Gal-beta-1,4-GlcNAc-R:alpha 2,8-sialyltransferase	ST8SIA3			
Q9Y6N5	Sulfide:quinone oxidoreductase, mitochondrial	SQOR			
Q9UNP4	Lactosylceramide alpha-2,3-sialyltransferase	ST3GAL5			
Q9BRT9	DNA replication complex GINS protein SLD5	GINS4			
Q99909	Protein SSX3	SSX3			
P16949	Stathmin	STMN1			
Q9UJZ1	Stomatin-like protein 2, mitochondrial	STOML2			
Q9UGK3	Signal-transducing adaptor protein 2	STAP2			
Q7RTU9	Stereocilin	STRC			
P41252	Isoleucine--tRNA ligase, cytoplasmic	IARS1			
Q9UMZ2	Synergin gamma	SYNRG			
Q8IZX4	Transcription initiation factor TFIID subunit 1-like	TAF1L			
Q8NG95	Olfactory receptor 7G3	OR7G3			
Q9NZP0	Olfactory receptor 6C3	OR6C3			
Q15646	2'-5'-oligoadenylate synthase-like protein	OASL			
Q7RTM1	Proton channel OTOP1	OTOP1			
Q86YG4	5'-nucleotidase domain-containing protein 4	NT5DC4			
Q8NG99	Olfactory receptor 7G2	OR7G2			
Q8NGB4	Olfactory receptor 4S1	OR4S1			
Q8NG85	Olfactory receptor 2L3	OR2L3			
Q5VU65	Nuclear pore membrane glycoprotein 210-like	NUP210L			
Q9NVD7	Alpha-parvin	PARVA			
Q53H96	Pyrroline-5-carboxylate reductase 3	PYCR3			
P11309	Serine/threonine-protein kinase pim-1	PIM1			
Q5SXH7	Pleckstrin homology domain-containing family S member 1	PLEKHS1			
P60900	Proteasome subunit alpha type-6	PSMA6			
Q5VT98	PRAME family member 20	PRAMEF20			
Q6UWS5	Protein PET117 homolog, mitochondrial	PET117			
Q8WUK0	Phosphatidylglycerophosphatase and protein-tyrosine phosphatase 1	PTPMT1			
Q9NYN1	Ras-like protein family member	RASL12			
Q9HCM1	Retroelement silencing factor 1	RESF1			
O14921	Regulator of G-protein signaling 13	RGS13			
Q96CC6	Inactive rhomboid protein 1	RHBDF1			
P17081	Rho-related GTP-binding protein RhoQ	RHOQ			
Q13156	Replication protein A 30 kDa subunit	RPA4			
Q13546	Receptor-interacting serine/threonine-protein kinase 1	RIPK1			
P46779	60S ribosomal protein L28	RPL28			
Q13405	39S ribosomal protein L49, mitochondrial	MRPL49			

Q9UHA3	Probable ribosome biogenesis protein RLP24	RSL24D1			
Q9BRJ2	39S ribosomal protein L45, mitochondrial	MRPL45			
Q96DB5	Regulator of microtubule dynamics protein 1	RMDN1			
Q8N5L8	Ribonuclease P protein subunit p25-like protein	RPP25L			
Q9BS91	Probable UDP-sugar transporter protein SLC35A5	SLC35A5			
Q9Y3V2	RWD domain-containing protein	RWDD3			
Q63ZE4	Solute carrier family 22 member 10	SLC22A10			
Q9NVS2	39S ribosomal protein S18a, mitochondrial	MRPS18A			
Q6ZUB0	Spermatogenesis-associated protein 31D4	SPATA31D4			
P33764	Protein S100-A3	S100A3			
Q13433	Zinc transporter ZIP6	SLC39A6			
O15235	28S ribosomal protein S12, mitochondrial	MRPS12			
Q6PDA7	Sperm-associated antigen 11A	SPAG11A			
Q92835	Phosphatidylinositol 3,4,5-trisphosphate 5-phosphatase 1	INPP5D			
A0A286YEX9	Small cysteine and glycine repeat-containing protein 10	SCYGR10			
O95171	Sciellin	SCEL			
Q96IW7	Vesicle-trafficking protein SEC22a	SEC22A			
P51170	Amiloride-sensitive sodium channel subunit gamma	SCNN1G			
Q92911	Sodium/iodide cotransporter	SLC5A5			
Q01118	Sodium channel protein type 7 subunit alpha	SCN7A			
Q6AZY7	Scavenger receptor class A member 3	SCARA3			
Q6UX34	Protein SNORC	SNORC			
O15105	Mothers against decapentaplegic homolog 7	SMAD7			
O75908	Sterol O-acyltransferase 2	SOAT2			
P22531	Small proline-rich protein 2E	SPRR2E			
Q68CJ6	Nuclear GTPase SLIP-GC	NUGGC			
Q8N0X2	Sperm-associated antigen 16 protein	SPAG16			
O14508	Suppressor of cytokine signaling 2	SOCS2			
Q9H106	Signal-regulatory protein delta	SIRPD			
Q3SXP7	Protein shisa-like-1	SHISAL1			
Q76I76	Protein phosphatase Slingshot homolog 2	SSH2			
O76061	Stanniocalcin-2	STC2			
Q9UHB9	Signal recognition particle subunit SRP68	SRP68			
Q8NEQ6	Steroid receptor-associated and regulated protein	SRARP			
Q9UKJ1	Paired immunoglobulin-like type 2 receptor alpha	PILRA			
Q9BY49	Peroxisomal trans-2-enoyl-CoA reductase	PECR			
Q96GM1	Phospholipid phosphatase-related protein type 2	PLPPR2			
Q9UKY4	Protein O-mannosyl-transferase 2	POMT2			
A6NI47	Putative POTE ankyrin domain family member M	POTEM			
Q96MI6	Protein phosphatase 1M	PPM1M			
Q96A00	Protein phosphatase 1 regulatory subunit 14A	PPP1R14A			
Q9Y4G2	Pleckstrin homology domain-containing family M member 1	PLEKHM1			
Q00577	Transcriptional activator protein Pur-alpha	PURA			

Q06124	Tyrosine-protein phosphatase non-receptor type 11	PTPN11			
Q05209	Tyrosine-protein phosphatase non-receptor type 12	PTPN12			
W6CW81	Pyrin domain-containing protein 5	PYDC5			
P46776	60S ribosomal protein L27a	RPL27A			
Q8WU17	E3 ubiquitin-protein ligase	RNF139			
P32969	60S ribosomal protein L9	RPL9			
Q5VYX0	Renalase	RNLS			
O15160	DNA-directed RNA polymerases I and III subunit RPAC1	POLR1C			
Q9Y2J0	Rabphilin-3A	RPH3A			
Q9BQ52	Zinc phosphodiesterase ELAC protein 2	ELAC2			
Q9Y6S9	Ribosomal protein S6 kinase-like	RPS6KL1			
P10153	Non-secretory ribonuclease	RNASE2			
Q9UIL1	Short coiled-coil protein	SCOC			
Q15047	Histone-lysine N-methyltransferase SETDB1	SETDB1			
Q8TBK2	N-lysine methyltransferase SETD6	SETD6			
Q9NS98	Semaphorin-3G	SEMA3G			
Q9H788	SH2 domain-containing protein 4A	SH2D4A			
Q6S5L8	SHC-transforming protein 4	SHC4			
Q9Y6P5	Sestrin-1	SESN1			
Q9UPS6	Histone-lysine N-methyltransferase SETD1B	SETD1B			
O43236	Septin-4	SEPTIN4			
Q9BY50	Signal peptidase complex catalytic subunit SEC11C	SEC11C			
O75396	Vesicle-trafficking protein SEC22b	SEC22B			
P31641	Sodium- and chloride-dependent taurine transporter	SLC6A6			
P60880	Synaptosomal-associated protein 25	SNAP25			
Q9HBX3	Uncharacterized protein encoded by SND1-IT1	SND1-IT1			
Q14940	Sodium/hydrogen exchanger 5	SLC9A5			
P57059	Serine/threonine-protein kinase SIK1	SIK1			
Q9BYE4	Small proline-rich protein 2G	SPRR2G			
Q92540	Protein SMG7	SMG7			
Q9Y5W7	Sorting nexin-14	SNX14			
Q96L94	Sorting nexin-22	SNX22			
A8MU46	Smoothelin-like protein 1	SMTNL1			
Q76KD6	Speriolin	SPATC1			
A6NNV3	Putative speedy protein E16	SPDYE16			
Q7Z6I5	Spermatogenesis-associated protein 12	SPATA12			
Q5JXA9	Signal-regulatory protein beta-2	SIRPB2			
Q9BR10	Spermatogenesis-associated protein 25	SPATA25			
Q9H5Y7	SLIT and NTRK-like protein 6	SLITRK6			
P35711	Transcription factor SOX-5	SOX5			
O75094	Slit homolog 3 protein	SLIT3			
Q499Z3	Schlafen-like protein 1	SLFNL1			
Q8NBT2	Kinetochore protein Spc24	SPC24			
Q0P670	Uncharacterized protein SPEM2	SPEM2			
A6NMB1	Sialic acid-binding Ig-like lectin	SIGLEC16			
Q99932	Sperm-associated antigen 8	SPAG8			
P78324	Tyrosine-protein phosphatase non-receptor type substrate 1	SIRPA			
Q96JI7	Spatacsin	SPG11			
Q8WXE9	Stonin-2	STON2			
Q9Y3F4	Serine-threonine kinase receptor-associated protein	STRAP			
Q9UBN4	Short transient receptor potential channel 4	TRPC4			

Q8TCJ2	Dolichyl-diphosphooligosaccharide--protein glycosyltransferase subunit STT3B	STT3B			
Q5T5J6	Transcriptional protein SWT1	SWT1			
Q2M3V2	Ankyrin repeat domain-containing protein SOWAHA	SOWAHA			
Q96GP6	Scavenger receptor class F member 2	SCARF2			
O75631	Uroplakin-3a	UPK3A			
O43295	SLIT-ROBO Rho GTPase-activating protein 3	SRGAP3			
Q9NPD8	Ubiquitin-conjugating enzyme E2 T	UBE2T			
O15400	Syntaxin-7	STX7			
A2VEC9	SCO-spondin	SSPOP			
O00186	Syntaxin-binding protein 3	STXBP3			
Q9H3Y6	Tyrosine-protein kinase Srms	SRMS			
Q9BYN0	Sulfiredoxin-1	SRXN1			
Q9BZ95	Histone-lysine N-methyltransferase NSD3	NSD3			
Q8NH72	Olfactory receptor 4C6	OR4C6			
P57054	Phosphatidylinositol N-acetylglucosaminyltransferase subunit P	PIGP			
Q8IXK0	Polyhomeotic-like protein 2	PHC2			
Q03052	POU domain, class 3, transcription factor 1	POU3F1			
O15018	PDZ domain-containing protein 2	PDZD2			
Q13427	Peptidyl-prolyl cis-trans isomerase G	PPIG			
O00469	Procollagen-lysine,2-oxoglutarate 5-dioxygenase 2	PLOD2			
P49683	Prolactin-releasing peptide receptor	PRLHR			
Q6PGN9	Proline/serine-rich coiled-coil protein 1	PSRC1			
O60542	Persephin	PSPN			
Q6ZW05	Patched domain-containing protein 4	PTCHD4			
P29074	Tyrosine-protein phosphatase non-receptor type 4	PTPN4			
Q9H2M9	Rab3 GTPase-activating protein non-catalytic subunit	RAB3GAP2			
O95294	RasGAP-activating-like protein 1	RASAL1			
Q9UKA8	Calcipressin-3	RCAN3			
P42331	Rho GTPase-activating protein 25	ARHGAP25			
Q6GYQ0	Ral GTPase-activating protein subunit alpha-1	RALGAPA1			
P20337	Ras-related protein Rab-3B	RAB3B			
Q2M1Z3	Rho GTPase-activating protein 31	ARHGAP31			
A8MT19	Putative rhophilin-2-like protein RHPN2P1	RHPN2P1			
Q8IUC4	Rhophilin-2	RHPN2			
Q06587	E3 ubiquitin-protein ligase RING1	RING1			
Q9NZE8	39S ribosomal protein L35, mitochondrial	MRPL35			
Q04912	Macrophage-stimulating protein receptor	MST1R			
Q8N4K4	Reprimo-like protein	RPRML			
Q96AG3	Solute carrier family 25 member 46	SLC25A46			
Q9P1V8	Sterile alpha motif domain-containing protein 15	SAMD15			
Q3SY17	Mitochondrial nicotinamide adenine dinucleotide transporter SLC25A52	SLC25A52			
Q9Y291	28S ribosomal protein S33, mitochondrial	MRPS33			

P26447	Protein S100-A4	S100A4			
Q9BRY0	Zinc transporter ZIP3	SLC39A3			
Q9NY26	Zinc transporter ZIP1	SLC39A1			
Q5K4L6	Solute carrier family 27 member 3	SLC27A3			
O43511	Pendrin	SLC26A4			
Q9HAS3	Solute carrier family 28 member 3	SLC28A3			
Q14542	Equilibrative nucleoside transporter 2	SLC29A2			
Q59EK9	RUN domain-containing protein 3A	RUNDC3A			
P82933	28S ribosomal protein S9, mitochondrial	MRPS9			
Q13761	Runt-related transcription factor 3	RUNX3			
Q6P1X5	Transcription initiation factor TFIID subunit 2	TAF2			
Q9Y3Z3	Deoxynucleoside triphosphate triphosphohydrolase SAMHD1	SAMHD1			
O75880	Protein SCO1 homolog, mitochondrial	SCO1			
Q9BWM7	Sideroflexin-3	SFXN3			
Q9NR31	GTP-binding protein SAR1a	SAR1A			
Q15393	Splicing factor 3B subunit 3	SF3B3			
A0A286YEV6	Small cysteine and glycine repeat-containing protein 4	SCYGR4			
Q9H3S1	Semaphorin-4A	SEMA4A			
Q99470	Stromal cell-derived factor 2	SDF2			
Q562F6	Shugoshin 2	SGO2			
Q5SQS7	SH2 domain-containing protein	SH2D4B			
Q86VE9	Serine incorporator 5	SERINC5			
A0PJK1	Sodium/glucose cotransporter 5	SLC5A10			
Q9BVW6	Small integral membrane protein	SMIM2			
Q9NRQ5	Single-pass membrane and coiled-coil domain-containing protein 4	SMCO4			
Q96T83	Sodium/hydrogen exchanger 7	SLC9A7			
Q1XH10	SKI/DACH domain-containing protein 1	SKIDA1			
Q96Q15	Serine/threonine-protein kinase SMG1	SMG1			
Q9UPR3	Protein SMG5	SMG5			
O95219	Sorting nexin-4	SNX4			
A0A2R8YCJ5	Small integral membrane protein 41	SMIM41			
O75159	Suppressor of cytokine signaling 5	SOCS5			
O43610	Protein sprouty homolog 3	SPRY3			
O75940	Survival of motor neuron-related-splicing factor 30	SMNDC1			
P35716	Transcription factor SOX-11	SOX11			
Q9NYA1	Sphingosine kinase 1	SPHK1			
Q9Y6Q2	Stonin-1	STON1			
B4DS77	Protein shisa-9	SHISA9			
P50225	Sulfotransferase 1A1	SULT1A1			
O00204	Sulfotransferase 2B1	SULT2B1			
Q69YW2	Protein stum homolog	STUM			
O14662	Syntaxin-16	STX16			
P14410	Sucrase-isomaltase, intestinal	SI			
Q8N5C6	S1 RNA-binding domain-containing protein 1	SRBD1			
Q13501	Sequestosome-1	SQSTM1			
P36956	Sterol regulatory element-binding protein 1	SREBF1			
Q92696	Geranylgeranyl transferase type-2 subunit alpha	RABGGTA			
Q9NUD9	GPI mannosyltransferase 2	PIGV			
Q8NEY8	Periphilin-1	PPHLN1			
P53801	Pituitary tumor-transforming gene 1 protein-interacting protein	PTTG1IP			
Q16647	Prostacyclin synthase	PTGIS			
Q6DKI7	Transmembrane protein PVRIG	PVRIG			
Q9NYR8	Retinol dehydrogenase 8	RDH8			

Q8N3Y7	Epidermal retinol dehydrogenase 2	SDR16C5			
Q92781	Retinol dehydrogenase 5	RDH5			
Q13127	RE1-silencing transcription factor	REST			
P35251	Replication factor C subunit 1	RFC1			
Q96A58	Ras-related and estrogen-regulated growth inhibitor	RERG			
Q96EH5	60S ribosomal protein L39-like	RPL39L			
P47914	60S ribosomal protein L29	RPL29			
Q7Z7H8	39S ribosomal protein L10, mitochondrial	MRPL10			
Q9BYC9	39S ribosomal protein L20, mitochondrial	MRPL20			
Q9BQ48	39S ribosomal protein L34, mitochondrial	MRPL34			
Q9Y2Y1	DNA-directed RNA polymerase III subunit RPC10	POLR3K			
P08922	Proto-oncogene tyrosine-protein kinase ROS	ROS1			
P61218	DNA-directed RNA polymerases I, II, and III subunit RPABC2	POLR2F			
P30876	DNA-directed RNA polymerase II subunit RPB2	POLR2B			
P63220	40S ribosomal protein S21	RPS21			
Q8IVM8	Solute carrier family 22 member 9	SLC22A9			
Q9NQQ7	Solute carrier family 35 member C2	SLC35C2			
P57060	RWD domain-containing protein 2B	RWDD2B			
Q7Z2H8	Proton-coupled amino acid transporter 1	SLC36A1			
Q9NVC3	Putative sodium-coupled neutral amino acid transporter 7	SLC38A7			
Q9Y226	Solute carrier family 22 member 13	SLC22A13			
Q8NBW4	Sodium-coupled neutral amino acid transporter 9	SLC38A9			
Q6PCB7	Long-chain fatty acid transport protein 1	SLC27A1			
P31151	Protein S100-A7	S100A7			
Q86VL8	Multidrug and toxin extrusion protein 2	SLC47A2			
Q7Z3Q1	Solute carrier family 46 member 3	SLC46A3			
Q17RB0	Retrotransposon Gag-like protein 8B	RTL8B			
O43304	SEC14-like protein 5	SEC14L5			
Q86UC2	Radial spoke head protein 3 homolog	RSPH3			
Q8N755	Solute carrier family 66 member 3	SLC66A3			
Q86SG5	Protein S100-A7A	S100A7A			
Q8N697	Solute carrier family 15 member 4	SLC15A4			
O95977	Sphingosine 1-phosphate receptor 4	S1PR4			
Q9H1V8	Sodium-dependent neutral amino acid transporter SLC6A17	SLC6A17			
Q15020	Squamous cell carcinoma antigen recognized by T-cells 3	SART3			
Q6SZW1	NAD	SARM1			
O75751	Solute carrier family 22 member 3	SLC22A3			
Q13950	Runt-related transcription factor 2	RUNX2			
Q5QGT7	Receptor-transporting protein 2	RTP2			
Q6SPF0	Atherin	SAMD1			
P21673	Diamine acetyltransferase 1	SAT1			
Q9C0D5	Protein TANC1	TANC1			
Q96IW2	SH2 domain-containing adapter protein D	SHD			
Q5VZ18	SH2 domain-containing adapter protein E	SHE			

Q96BR1	Serine/threonine-protein kinase Sgk3	SGK3			
Q96ES7	SAGA-associated factor 29	SGF29			
P43007	Neutral amino acid transporter A	SLC1A4			
Q8WVN6	Secreted and transmembrane protein 1	SECTM1			
Q9NWH9	SAFB-like transcription modulator	SLTM			
Q8IYR2	SET and MYND domain-containing protein 4	SMYD4			
Q96PI1	Small proline-rich protein 4	SPRR4			
Q5T5P2	Sickle tail protein homolog	KIAA1217			
Q4G0N8	Sodium/hydrogen exchanger 10	SLC9C1			
Q5W111	SPRY domain-containing protein	SPRYD7			
Q9NWH7	Spermatogenesis-associated protein 6	SPATA6			
Q8N0X7	Spartin	SPART			
Q8IY18	Structural maintenance of chromosomes protein 5	SMC5			
P35321	Cornifin-A	SPRR1A			
O60264	SWI/SNF-related matrix-associated actin-dependent regulator of chromatin subfamily A member 5	SMARCA5			
Q9NRC6	Spectrin beta chain, non-erythrocytic 5	SPTBN5			
O94991	SLIT and NTRK-like protein 5	SLITRK5			
Q8NCR6	Spermatid-specific manchette-related protein 1	SMRP1			
Q92186	Alpha-2,8-sialyltransferase 8B	ST8SIA2			
Q99865	Spindlin-2A	SPIN2A			
Q13285	Steroidogenic factor 1	NR5A1			
Q8WWL2	Protein spire homolog 2	SPIRE2			
Q6PIF2	Synaptonemal complex central element protein 2	SYCE2			
Q9P2F5	Storkhead-box protein 2	STOX2			
Q8NG80	Olfactory receptor 2L5	OR2L5			
O96028	Histone-lysine N-methyltransferase NSD2	NSD2			
P58417	Neurexophilin-1	NXPH1			
Q6UX06	Olfactomedin-4	OLFM4			
Q9H342	Olfactory receptor 51J1	OR51J1			
Q9NX40	OCIA domain-containing protein	OCIAD1			
Q8NGX6	Olfactory receptor 10R2	OR10R2			
Q8NGE5	Olfactory receptor 10A7	OR10A7			
Q8NGT1	Olfactory receptor 2K2	OR2K2			
Q96JY6	PDZ and LIM domain protein 2	PDLIM2			
P42356	Phosphatidylinositol 4-kinase alpha	PI4KA			
Q9BX93	Group XIIB secretory phospholipase A2-like protein	PLA2G12B			
Q96DM1	PiggyBac transposable element-derived protein 4	PGBD4			
O15305	Phosphomannomutase 2	PMM2			
P49335	POU domain, class 3, transcription factor 4	POU3F4			
Q9Y342	Plasmolipin	PLLP			
O60568	Multifunctional procollagen lysine hydroxylase and glycosyltransferase LH3	PLOD3			
Q8N2A8	Mitochondrial cardiolipin hydrolase	PLD6			
Q9BXR3	Endogenous retrovirus group K member 6 Pol protein	ERVK-6			
Q6NYC8	Phostensin	PPP1R18			
Q8TCE9	Placental protein 13-like	LGALS14			
Q96KN3	Homeobox protein PKNOX2	PKNOX2			
Q8NHP8	Putative phospholipase B-like 2	PLBD2			
O15120	1-acyl-sn-glycerol-3-phosphate acyltransferase beta	AGPAT2			

P28069	Pituitary-specific positive transcription factor 1	POU1F1			
O43164	E3 ubiquitin-protein ligase Praja-2	PJA2			
Q8NF86	Serine protease 33	PRSS33			
Q9H606	Proline-rich protein, Y-linked	PRORY			
Q86TP1	Exopolyphosphatase PRUNE1	PRUNE1			
A6NIE9	Putative serine protease 29	PRSS29P			
O60813	PRAME family member 11	PRAMEF11			
Q8TB61	Adenosine 3'-phospho 5'-phosphosulfate transporter 1	SLC35B2			
Q8IY50	Putative thiamine transporter SLC35F3	SLC35F3			
Q7RTT9	Equilibrative nucleoside transporter 4	SLC29A4			
Q8WV83	Solute carrier family 35 member	SLC35F5			
L0R6Q1	SLC35A4 upstream open reading frame protein	SLC35A4			
Q2M3R5	Solute carrier family 35 member G1	SLC35G1			
Q9H2H9	Sodium-coupled neutral amino acid transporter 1	SLC38A1			
P82930	28S ribosomal protein S34, mitochondrial	MRPS34			
Q8IZM9	Probable sodium-coupled neutral amino acid transporter 6	SLC38A6			
Q8N8I0	Sterile alpha motif domain-containing protein 12	SAMD12			
P34925	Tyrosine-protein kinase RYK	RYK			
P04271	Protein S100-B	S100B			
Q96FL8	Multidrug and toxin extrusion protein 1	SLC47A1			
A6NKG5	Retrotransposon-like protein 1	RTL1			
Q8IZC4	Rhotekin-2	RTKN2			
Q9H2X9	Solute carrier family 12 member 5	SLC12A5			
Q9BZW2	Solute carrier family 13 member 1	SLC13A1			
Q92541	RNA polymerase-associated protein RTF1 homolog	RTF1			
Q13183	Solute carrier family 13 member 2	SLC13A2			
Q9BWD3	Retrotransposon Gag-like protein 8A	RTL8A			
Q86YT5	Solute carrier family 13 member 5	SLC13A5			
Q9H9S3	Protein transport protein Sec61 subunit alpha isoform 2	SEC61A2			
P18405	3-oxo-5-alpha-steroid 4-dehydrogenase 1	SRD5A1			
Q6NT16	MFS-type transporter SLC18B1	SLC18B1			
Q7L4I2	Arginine/serine-rich coiled-coil protein 2	RSRC2			
P31213	3-oxo-5-alpha-steroid 4-dehydrogenase 2	SRD5A2			
Q9NSD5	Sodium- and chloride-dependent GABA transporter 2	SLC6A13			
Q8TCU3	Solute carrier family 7 member 13	SLC7A13			
Q96HN2	Adenosylhomocysteinase 3	AHCYL2			
Q9BQQ7	Receptor-transporting protein 3	RTP3			
Q96NB2	Sideroflexin-2	SFXN2			
Q9HBY8	Serine/threonine-protein kinase Sgk2	SGK2			
Q9NTN9	Semaphorin-4G	SEMA4G			
Q6UWF3	SLP adapter and CSK-interacting membrane protein	SCIMP			
Q9NPR2	Semaphorin-4B	SEMA4B			
A6NKC9	SH2 domain-containing protein 7	SH2D7			
Q86UB9	Transmembrane protein 135	TMEM135			
Q9P283	Semaphorin-5B	SEMA5B			
Q16181	Septin-7	SEPTIN7			
O95486	Protein transport protein Sec24A	SEC24A			
P78330	Phosphoserine phosphatase	PSPH			

Q8IYX7	Stabilizer of axonemal microtubules 1	SAXO1			
P0C7V7	Putative signal peptidase complex catalytic subunit SEC11B	SEC11B			
Q9BR46	Putative uncharacterized protein SCP2D1-AS1	SCP2D1-AS1			
P13521	Secretogranin-2	SCG2			
Q9BV90	U11/U12 small nuclear ribonucleoprotein 25 kDa protein	SNRNP25			
Q9NZC9	SWI/SNF-related matrix-associated actin-dependent regulator of chromatin subfamily A-like protein 1	SMARCAL1			
Q96E16	Small integral membrane protein 19	SMIM19			
Q9BQ49	Small integral membrane protein	SMIM7			
P35325	Small proline-rich protein 2B	SPRR2B			
A0A1B0GVV1	Small integral membrane protein 35	SMIM35			
Q9BRV8	Suppressor of IKBKE 1	SIKE1			
A0A1B0GVQ0	Small regulatory polypeptide of amino acid response	SPAAR			
O75182	Paired amphipathic helix protein Sin3b	SIN3B			
B2RUZ4	Small integral membrane protein	SMIM1			
P22528	Cornifin-B	SPRR1B			
A0A1B0GUA5	Small integral membrane protein 32	SMIM32			
A6NKU9	Speedy protein E3	SPDYE3			
O94964	Protein SOGA1	SOGA1			
A6NHP3	Speedy protein E2B	SPDYE2B			
O15524	Suppressor of cytokine signaling 1	SOCS1			
Q9H4L7	SWI/SNF-related matrix-associated actin-dependent regulator of chromatin subfamily A containing DEAD/H box 1	SMARCAD1			
Q5MJ10	Sperm protein associated with the nucleus on the X chromosome N2	SPANXN2			
Q9HAT2	Sialate O-acetylesterase	SIAE			
Q15831	Serine/threonine-protein kinase STK11	STK11			
Q9HA77	Probable cysteine--tRNA ligase, mitochondrial	CARS2			
Q86TL2	Store-operated calcium entry regulator STIMATE	STIMATE			
Q9Y3M8	StAR-related lipid transfer protein 13	STARD13			
P49675	Steroidogenic acute regulatory protein, mitochondrial	STAR			
O95425	Supervillin	SVIL			
Q6IE37	Ovostatin homolog 1	OVOS1			
P0DPK4	Notch homolog 2 N-terminal-like protein C	NOTCH2NLC			
Q15619	Olfactory receptor 1C1	OR1C1			
O60313	Dynamin-like 120 kDa protein, mitochondrial	OPA1			
Q8N543	Prolyl 3-hydroxylase OGFOD1	OGFOD1			
Q8NGQ5	Olfactory receptor 9Q1	OR9Q1			
Q9NZT2	Opioid growth factor receptor	OGFR			
Q96MG7	Non-structural maintenance of chromosomes element 3 homolog	NSMCE3			
Q68BL7	Olfactomedin-like protein 2A	OLFML2A			
A6NND4	Olfactory receptor 2AT4	OR2AT4			
Q96SN7	Protein orai-2	ORAI2			
Q9BSG0	Protease-associated domain-containing protein 1	PRADC1			
Q9C0D0	Phosphatase and actin regulator 1	PHACTR1			
Q9UMS5	Protein PHTF1	PHTF1			

Q8TBX8	Phosphatidylinositol 5-phosphate 4-kinase type-2 gamma	PIP4K2C			
Q9H490	Phosphatidylinositol glycan anchor biosynthesis class U protein	PIGU			
O00151	PDZ and LIM domain protein 1	PDLIM1			
Q9H1Q7	PC-esterase domain-containing protein 1A	PCED1A			
O00743	Serine/threonine-protein phosphatase 6 catalytic subunit	PPP6C			
Q08209	Serine/threonine-protein phosphatase 2B catalytic subunit alpha isoform	PPP3CA			
Q8TAE6	Protein phosphatase 1 regulatory subunit 14C	PPP1R14C			
P48454	Serine/threonine-protein phosphatase 2B catalytic subunit gamma isoform	PPP3CC			
Q9NRQ2	Phospholipid scramblase 4	PLSCR4			
O75038	1-phosphatidylinositol 4,5-bisphosphate phosphodiesterase eta-2	PLCH2			
Q8IWE5	Pleckstrin homology domain-containing family M member 2	PLEKHM2			
Q16513	Serine/threonine-protein kinase N2	PKN2			
A1L453	Serine protease 38	PRSS38			
Q5VXH5	PRAME family member 7	PRAMEF7			
Q9H4Q3	PR domain zinc finger protein 13	PRDM13			
Q5SWL7	PRAME family member 14	PRAMEF14			
Q13308	Inactive tyrosine-protein kinase 7	PTK7			
Q86YD1	Prostate tumor-overexpressed gene 1 protein	PTOV1			
Q9H3S7	Tyrosine-protein phosphatase non-receptor type 23	PTPN23			
P61513	60S ribosomal protein L37a	RPL37A			
Q9H0U6	39S ribosomal protein L18, mitochondrial	MRPL18			
Q8WXG1	Radical S-adenosyl methionine domain-containing protein 2	RSAD2			
P62308	Small nuclear ribonucleoprotein G	SNRPG			
Q5TZJ5	Spermatogenesis-associated protein 31A1	SPATA31A1			
Q9NR83	SLC2A4 regulator	SLC2A4RG			
A8MWD9	Putative small nuclear ribonucleoprotein G-like protein 15	SNRPGP15			
Q6T423	Solute carrier family 22 member 25	SLC22A25			
Q3KQZ1	Solute carrier family 25 member 35	SLC25A35			
Q96H72	Zinc transporter ZIP13	SLC39A13			
A6NKF1	SAC3 domain-containing protein 1	SAC3D1			
P29377	Protein S100-G	S100G			
P25815	Protein S100-P	S100P			
Q9NUM3	Zinc transporter ZIP9	SLC39A9			
Q6ZR62	Retrotransposon Gag-like protein 4	RTL4			
Q9UDX4	SEC14-like protein 3	SEC14L3			
Q14151	Scaffold attachment factor B2	SAFB2			
Q9NWF4	Solute carrier family 52, riboflavin transporter, member 1	SLC52A1			
Q9BXY4	R-spondin-3	RSPO3			
Q9UHA2	SS18-like protein 2	SS18L2			
Q6UXX9	R-spondin-2	RSPO2			
Q9NXZ1	Sarcoma antigen 1	SAGE1			
A1A4F0	Putative uncharacterized protein SLC66A1L	SLC66A1L			
O76082	Solute carrier family 22 member 5	SLC22A5			

O15245	Solute carrier family 22 member 1	SLC22A1			
Q9Y230	RuvB-like 2	RUVBL2			
Q8WXA3	RUN and FYVE domain-containing protein 2	RUFY2			
Q96T51	RUN and FYVE domain-containing protein 1	RUFY1			
Q70HW3	S-adenosylmethionine mitochondrial carrier protein	SLC25A26			
Q8N6K7	Sterile alpha motif domain-containing protein 3	SAMD3			
Q8NGQ3	Olfactory receptor 1S2	OR1S2			
Q8NGY3	Olfactory receptor 6K3	OR6K3			
Q96F24	Nuclear receptor-binding factor 2	NRBF2			
Q7RTY7	Ovochymase-1	OVCH1			
Q8NGJ1	Olfactory receptor 4D6	OR4D6			
Q9BZF2	Oxysterol-binding protein-related protein 7	OSBPL7			
Q9H3D4	Tumor protein 63	TP63			
Q9UBF8	Phosphatidylinositol 4-kinase beta	PI4KB			
Q8IZL8	Proline-, glutamic acid- and leucine-rich protein 1	PELP1			
O15534	Period circadian protein homolog 1	PER1			
Q9UPN7	Serine/threonine-protein phosphatase 6 regulatory subunit 1	PPP6R1			
A8MUH7	Putative PDZ domain-containing protein PDZK1P1	PDZK1P1			
Q9NVS9	Pyridoxine-5'-phosphate oxidase	PNPO			
Q9QC07	Endogenous retrovirus group K member 18 Pol protein	ERVK-18			
P00747	Plasminogen	PLG			
Q8WWY3	U4/U6 small nuclear ribonucleoprotein Prp31	PRPF31			
P11908	Ribose-phosphate pyrophosphokinase 2	PRPS2			
Q5VWM4	PRAME family member 8	PRAMEF8			
P86481	Proline-rich protein 20B	PRR20B			
A6NGN4	PRAME family member 25	PRAMEF25			
O75475	PC4 and SFRS1-interacting	PSIP1			
Q9H2W6	39S ribosomal protein L46, mitochondrial	MRPL46			
Q8IWN7	Retinitis pigmentosa 1-like 1 protein	RP1L1			
Q9GZS1	DNA-directed RNA polymerase I subunit RPA49	POLR1E			
Q13084	39S ribosomal protein L28, mitochondrial	MRPL28			
Q2KHN1	RING finger protein 151	RNF151			
P52435	DNA-directed RNA polymerase II subunit RPB11-a	POLR2J			
P78345	Ribonuclease P protein subunit p38	RPP38			
Q01534	Testis-specific Y-encoded protein 1	TSPY1			
A6NKD2	Testis-specific Y-encoded protein 2	TSPY2			
Q5VX71	Sushi domain-containing protein	SUSD4			
O60279	Sushi domain-containing protein	SUSD5			
P0DMP2	SLIT-ROBO Rho GTPase-activating protein 2B	SRGAP2B			
P0DJJ0	SLIT-ROBO Rho GTPase-activating protein 2C	SRGAP2C			
P43307	Translocon-associated protein subunit alpha	SSR1			
P32745	Somatostatin receptor type 3	SSTR3			
Q9UQF0	Syncytin-1	ERVW-1			
Q13190	Syntaxin-5	STX5			
Q14714	Sarcospan	SSPN			

P30874	Somatostatin receptor type 2	SSTR2			
Q9UQ35	Serine/arginine repetitive matrix protein 2	SRRM2			
Q9BRV3	Sugar transporter SWEET1	SLC50A1			
Q9BRL6	Serine/arginine-rich splicing factor 8	SRSF8			
Q14140	SERTA domain-containing protein 2	SERTAD2			
Q9P1P4	Putative trace amine-associated receptor 3	TAAR3P			
P20226	TATA-box-binding protein	TBP			
B9A6J9	TBC1 domain family member 3L	TBC1D3L			
O43435	T-box transcription factor TBX1	TBX1			
Q9HA65	TBC1 domain family member 17	TBC1D17			
Q2M2D7	TBC1 domain family member 28	TBC1D28			
Q7Z7N9	Transmembrane protein 179B	TMEM179B			
Q9UBK9	Protein UXT	UXT			
Q8IV63	Inactive serine/threonine-protein kinase VRK3	VRK3			
Q9H6D3	XK-related protein 8	XKR8			
P57081	tRNA	WDR4			
Q9H267	Vacuolar protein sorting-associated protein 33B	VPS33B			
Q96IR3	Putative uncharacterized protein MGC15705				
A8MQ14	Zinc finger protein 850	ZNF850			
Q5SVQ8	Zinc finger and BTB domain-containing protein 41	ZBTB41			
O75541	Zinc finger protein 821	ZNF821			
O75467	Zinc finger protein 324A	ZNF324			
Q9UPR6	Zinc finger RNA-binding protein 2	ZFR2			
O96006	E3 SUMO-protein ligase ZBED1	ZBED1			
Q2TB10	Zinc finger protein 800	ZNF800			
Q96NI8	Zinc finger protein 570	ZNF570			
Q5BKZ1	DBIRD complex subunit ZNF326	ZNF326			
A6NGD5	Zinc finger and SCAN domain-containing protein 5C	ZSCAN5C			
Q4V348	Zinc finger protein 658B	ZNF658B			
O15156	Zinc finger and BTB domain-containing protein 7B	ZBTB7B			
Q9NUE0	Palmitoyltransferase ZDHHC18	ZDHHC18			
Q8IYP9	Palmitoyltransferase ZDHHC23	ZDHHC23			
O75290	Zinc finger protein 780A	ZNF780A			
Q9BSK1	Zinc finger protein 577	ZNF577			
Q15940	Putative zinc finger protein 726P1	ZNF726P1			
Q96MV8	Palmitoyltransferase ZDHHC15	ZDHHC15			
P36508	Zinc finger protein 76	ZNF76			
Q15928	Zinc finger protein 141	ZNF141			
O00488	Zinc finger protein 593	ZNF593			
P15622	Zinc finger protein 250	ZNF250			
Q15916	Zinc finger and BTB domain-containing protein 6	ZBTB6			
Q8N554	Zinc finger protein 276	ZNF276			
Q68DY9	Zinc finger protein 772	ZNF772			
Q8WTX9	Palmitoyltransferase ZDHHC1	ZDHHC1			
Q8NCA9	Zinc finger protein 784	ZNF784			
Q9Y2P7	Zinc finger protein 256	ZNF256			
P52737	Zinc finger protein 136	ZNF136			
Q8NH79	Olfactory receptor 6X1	OR6X1			
Q15622	Olfactory receptor 7A5	OR7A5			
Q8NH83	Olfactory receptor 4A5	OR4A5			
Q5VV17	OTU domain-containing protein 1	OTUD1			
Q8NG75	Olfactory receptor 5T1	OR5T1			
Q12889	Oviduct-specific glycoprotein	OVGP1			
Q9BRB3	Phosphatidylinositol N-acetylglucosaminyltransferase subunit Q	PIGQ			
Q9BVI0	PHD finger protein 20	PHF20			
P55771	Paired box protein Pax-9	PAX9			
Q9H720	PGAP2-interacting protein	CWH43			

A0A1W2PR82	Protein PERCC1	PERCC1			
Q9BRX2	Protein pelota homolog	PELO			
Q9ULN7	Paraneoplastic antigen-like protein 8B	PNMA8B			
P37231	Peroxisome proliferator-activated receptor gamma	PPARG			
Q9ULM0	Pleckstrin homology domain-containing family H member 1	PLEKHH1			
P24158	Myeloblastin	PRTN3			
Q9NRZ5	1-acyl-sn-glycerol-3-phosphate acyltransferase delta	AGPAT4			
Q9NUJ7	PI-PLC X domain-containing protein 1	PLCXD1			
Q2L4Q9	Serine protease 53	PRSS53			
Q96HE9	Proline-rich protein 11	PRR11			
Q9UKY0	Prion-like protein doppel	PRND			
Q96BP3	Peptidylprolyl isomerase domain and WD repeat-containing protein 1	PPWD1			
Q8N4B5	Proline-rich protein 18	PRR18			
Q9H6K5	Proline-rich protein 36	PRR36			
P37837	Transaldolase	TALDO1			
Q16650	T-box brain protein 1	TBR1			
Q13769	THO complex subunit 5 homolog	THOC5			
Q53EQ6	Tigger transposable element-derived protein 5	TIGD5			
Q9H5Q4	Dimethyladenosine transferase 2, mitochondrial	TFB2M			
Q8TBZ9	Testis-expressed protein 47	TEX47			
O60235	Transmembrane protease serine 11D	TMPRSS11D			
Q16762	Thiosulfate sulfurtransferase	TST			
P05543	Thyroxine-binding globulin	SERPINA7			
Q9P2T0	Testicular haploid expressed gene protein	THEG			
Q04726	Transducin-like enhancer protein	TLE3			
Q4V9L6	Transmembrane protein 119	TMEM119			
A0A0J9YXG5	T cell receptor beta joining 1-4	TRBJ1-4			
Q8NEK8	Terminal nucleotidyltransferase 5D	TENT5D			
P32780	General transcription factor IIH subunit 1	GTF2H1			
A2RU30	Protein TESPA1	TESPA1			
Q9Y4F4	TOG array regulator of axonemal microtubules protein 1	TOGARAM1			
A0A1W2PR48	Transducin-like enhancer protein 7	TLE7			
A0A286YF58	Transmembrane protein 271	TMEM271			
Q66K66	Transmembrane protein 198	TMEM198			
Q9NZ01	Very-long-chain enoyl-CoA reductase	TECR			
Q96EY4	Translation machinery-associated protein 16	TMA16			
Q92544	Transmembrane 9 superfamily member 4	TM9SF4			
O95932	Protein-glutamine gamma-glutamyltransferase 6	TGM6			
Q5U3C3	Transmembrane protein 164	TMEM164			
Q8N2M4	Lysoplasmalogenase-like protein TMEM86A	TMEM86A			
Q14258	E3 ubiquitin/ISG15 ligase TRIM25	TRIM25			
Q96FX7	tRNA	TRMT61A			
Q13061	Triadin	TRDN			
Q6ZT21	Transmembrane protein with metallophosphoesterase domain	TMPPE			
Q9NUP7	tRNA:m	TRMT13			

Q8WVR3	Trafficking protein particle complex subunit 14	TRAPPC14			
A0A075B706	T cell receptor delta joining 1	TRDJ1			
Q9Y3Q3	Transmembrane emp24 domain-containing protein 3	TMED3			
Q86XT4	E3 ubiquitin-protein ligase	TRIM50			
Q13472	DNA topoisomerase 3-alpha	TOP3A			
Q8WZ04	Transmembrane O-methyltransferase	LRTOMT			
Q9C0B7	Transport and Golgi organization protein 6 homolog	TANGO6			
P12270	Nucleoprotein TPR	TPR			
Q9H3M7	Thioredoxin-interacting protein	TXNIP			
O95881	Thioredoxin domain-containing protein 12	TXNDC12			
P42681	Tyrosine-protein kinase TXK	TXK			
Q6Q0C0	E3 ubiquitin-protein ligase TRAF7	TRAF7			
Q6A555	Thioredoxin domain-containing protein 8	TXNDC8			
A0A0A6YYK7	T cell receptor alpha variable 19	TRAV19			
A0A0A6YYK1	T cell receptor alpha variable 8-1	TRAV8-1			
O00295	Tubby-related protein 2	TULP2			
Q969D9	Thymic stromal lymphopoietin	TSLP			
A0A0B4J27	T cell receptor alpha variable 20	TRAV20			
Q7Z4L5	Tetratricopeptide repeat protein 21B	TTC21B			
Q7Z4N2	Transient receptor potential cation channel subfamily M member 1	TRPM1			
A0A5B0	T cell receptor beta variable 14	TRBV14			
Q9C0H2	Protein tweety homolog 3	TTYH3			
P62256	Ubiquitin-conjugating enzyme E2 H	UBE2H			
Q14694	Ubiquitin carboxyl-terminal hydrolase 10	USP10			
Q9UPT9	Ubiquitin carboxyl-terminal hydrolase 22	USP22			
Q8N806	Putative E3 ubiquitin-protein ligase UBR7	UBR7			
Q969X6	U3 small nucleolar RNA-associated protein 4 homolog	UTP4			
P62068	Ubiquitin carboxyl-terminal hydrolase 46	USP46			
Q99536	Synaptic vesicle membrane protein VAT-1 homolog	VAT1			
Q8IUA0	WAP four-disulfide core domain protein 8	WFDC8			
Q9UPY6	Wiskott-Aldrich syndrome protein family member 3	WASF3			
Q96G27	WW domain-binding protein 1	WBP1			
Q9UNX4	WD repeat-containing protein 3	WDR3			
Q1RN00	Putative uncharacterized protein LOC151760				
Q6ZV60	Putative uncharacterized protein encoded by LINC00173	LINC00173			
Q75L30	Putative uncharacterized protein FLJ92257				
Q86V86	Serine/threonine-protein kinase pim-3	PIM3			
O15541	E3 ubiquitin-protein ligase RNF113A	RNF113A			
P62847	40S ribosomal protein S24	RPS24			
P42677	40S ribosomal protein S27	RPS27			
Q9UHI7	Solute carrier family 23 member 1	SLC23A1			
Q9Y2D2	UDP-N-acetylglucosamine transporter	SLC35A3			

P0C874	Spermatogenesis-associated protein 31D3	SPATA31D3			
Q9Y224	RNA transcription, translation and transport factor protein	RTRAF			
Q8TD33	Secretoglobin family 1C member	SCGB1C1			
Q53H47	Histone-lysine N-methyltransferase SETMAR	SETMAR			
Q96LD1	Zeta-sarcoglycan	SGCZ			
Q9BYH1	Seizure 6-like protein	SEZ6L			
Q68CR1	Protein sel-1 homolog 3	SEL1L3			
O14492	SH2B adapter protein 2	SH2B2			
Q07699	Sodium channel subunit beta-1	SCN1B			
O95562	Vesicle transport protein SFT2B	SFT2D2			
Q92765	Secreted frizzled-related protein 3	FRZB			
Q86XK3	Swi5-dependent recombination DNA repair protein 1 homolog	SFR1			
Q96JE7	Protein transport protein Sec16B	SEC16B			
Q9H4B6	Protein salvador homolog 1	SAV1			
Q9Y3A5	Ribosome maturation protein SBDS	SBDS			
Q2M3M2	Sodium/glucose cotransporter 4	SLC5A9			
Q8WU76	Sec1 family domain-containing protein 2	SCFD2			
P51168	Amiloride-sensitive sodium channel subunit beta	SCNN1B			
Q8IUW3	Spermatogenesis-associated protein 2-like protein	SPATA2L			
P38935	DNA-binding protein SMUBP-2	IGHMBP2			
Q5VVC0	Protein SPO16 homolog	SPO16			
Q8N9U9	Putative uncharacterized protein SPANXA2-OT1	SPANXA2-OT1			
Q6ZMD2	Protein spinster homolog 3	SPNS3			
P09012	U1 small nuclear ribonucleoprotein A	SNRPA			
O60493	Sorting nexin-3	SNX3			
Q96EB6	NAD-dependent protein deacetylase sirtuin-1	SIRT1			
Q9UIB8	SLAM family member 5	CD84			
Q96DD7	Protein shisa-4	SHISA4			
O75391	Sperm-associated antigen 7	SPAG7			
Q9C093	Sperm flagellar protein 2	SPEF2			
Q9HBM1	Kinetochore protein Spc25	SPC25			
Q8WWF3	Serine-rich single-pass membrane protein 1	SSMEM1			
Q92783	Signal transducing adapter molecule 1	STAM			
Q9C0K7	STE20-related kinase adapter protein beta	STRADB			
Q9UMX1	Suppressor of fused homolog	SUFU			
O14804	Trace amine-associated receptor 5	TAAR5			
P48643	T-complex protein 1 subunit epsilon	CCT5			
P49591	Serine--tRNA ligase, cytoplasmic	SARS1			
O75410	Transforming acidic coiled-coil-containing protein 1	TACC1			
Q86V81	THO complex subunit 4	ALYREF			
Q13888	General transcription factor IIH subunit 2	GTF2H2			
Q8NBT3	Transmembrane protein 145	TMEM145			
Q8IV31	Transmembrane protein 139	TMEM139			
A6NCN8	Testis-expressed protein 52	TEX52			
A0A0A0MT87	T cell receptor beta joining 2-4	TRBJ2-4			
Q04725	Transducin-like enhancer protein	TLE2			
A6NEH6	Transmembrane protein 247	TMEM247			
P04053	DNA nucleotidylexotransferase	DNTT			
Q8IYJ3	Synaptotagmin-like protein 1	SYTL1			
Q9P2C4	Transmembrane protein 181	TMEM181			

Q4KMG9	Transmembrane protein 52B	TMEM52B			
A0A0U1RQS6	Transmembrane protein 275	TMEM275			
Q8N8B7	Transcription elongation factor A N-terminal and central domain-containing protein	TCEANC			
Q8IYL2	Probable tRNA	TRMT44			
Q6AZZ1	E3 ubiquitin-protein ligase	TRIM68			
Q6F5E7	Protein TXNRD3NB	TXNRD3NB			
P09493	Tropomyosin alpha-1 chain	TPM1			
Q96RJ3	Tumor necrosis factor receptor superfamily member 13C	TNFRSF13C			
Q96HE8	Transmembrane protein 80	TMEM80			
Q8WVJ9	Twist-related protein 2	TWIST2			
Q9UJ04	Testis-specific Y-encoded-like protein 4	TSPYL4			
A0A0B4J24	T cell receptor alpha variable 1-1	TRAV1-1			
Q2T9J0	Peroxisomal leader peptide-processing protease	TYSND1			
P54219	Chromaffin granule amine transporter	SLC18A1			
Q86UX7	Fermitin family homolog 3	FERMT3			
Q70CQ2	Ubiquitin carboxyl-terminal hydrolase 34	USP34			
P0DN76	Splicing factor U2AF 35 kDa subunit-like protein	U2AF1L5			
Q9GZS3	WD repeat-containing protein 61	WDR61			
Q9H967	WD repeat-containing protein 76	WDR76			
P41221	Protein Wnt-5a	WNT5A			
P46094	Chemokine XC receptor 1	XCR1			
Q8N7P7	Uncharacterized protein FLJ40521				
O43847	Nardilysin	NRDC			
A6NF83	Nuclear protein 2	NUPR2			
Q8NGF8	Olfactory receptor 4B1	OR4B1			
Q8NBP7	Proprotein convertase subtilisin/kexin type 9	PCSK9			
P29122	Proprotein convertase subtilisin/kexin type 6	PCSK6			
O00444	Serine/threonine-protein kinase PLK4	PLK4			
Q9WJR5	Endogenous retrovirus group K member 19 Pol protein	ERVK-19			
P09923	Intestinal-type alkaline phosphatase	ALPI			
Q7Z4H8	Protein O-glucosyltransferase 3	POGLUT3			
P55347	Homeobox protein PKNOX1	PKNOX1			
Q8TAA3	Proteasome subunit alpha-type 8	PSMA8			
P23467	Receptor-type tyrosine-protein phosphatase beta	PTPRB			
Q03393	6-pyruvoyl tetrahydrobiopterin synthase	PTS			
P23471	Receptor-type tyrosine-protein phosphatase zeta	PTPRZ1			
O95153	Peripheral-type benzodiazepine receptor-associated protein 1	TSPOAP1			
P61313	60S ribosomal protein L15	RPL15			
Q9BRS2	Serine/threonine-protein kinase RIO1	RIOK1			
O43353	Receptor-interacting serine/threonine-protein kinase 2	RIPK2			
O00237	E3 ubiquitin-protein ligase	RNF103			
Q9H9Y2	Ribosome production factor 1	RPF1			
Q9H6T3	RNA polymerase II-associated protein 3	RPAP3			
Q8N5U6	RING finger protein 10	RNF10			
P62854	40S ribosomal protein S26	RPS26			
Q8N9S9	Sorting nexin-31	SNX31			
Q8TCT8	Signal peptide peptidase-like 2A	SPPL2A			
P61278	Somatostatin	SST			

P51688	N-sulphoglucosamine sulphohydrolase	SGSH			
Q7KZ85	Transcription elongation factor SPT6	SUPT6H			
Q9UQ90	Paraplegin	SPG7			
Q9BXA5	Succinate receptor 1	SUCNR1			
Q15022	Polycomb protein SUZ12	SUZ12			
Q8N205	Nesprin-4	SYNE4			
Q6IPX1	TBC1 domain family member 3C	TBC1D3C			
Q86TJ2	Transcriptional adapter 2-beta	TADA2B			
A0A087X179	TBC1 domain family member 3E	TBC1D3E			
Q9NS69	Mitochondrial import receptor subunit TOM22 homolog	TOMM22			
Q8NFZ5	TNFAIP3-interacting protein 2	TNIP2			
Q9H2S6	Tenomodulin	TNMD			
O60296	Trafficking kinesin-binding protein 2	TRAK2			
Q6UXZ0	Transmembrane and immunoglobulin domain-containing protein 1	TMIGD1			
Q0P6H9	Transmembrane protein 62	TMEM62			
A0PK05	Transmembrane protein 72	TMEM72			
O14787	Transportin-2	TNPO2			
P14679	Tyrosinase	TYR			
P16473	Thyrotropin receptor	TSHR			
A0A075B6L2	Probable non-functional T cell receptor gamma variable 11	TRGV11			
Q8IYT8	Serine/threonine-protein kinase ULK2	ULK2			
P63146	Ubiquitin-conjugating enzyme E2 B	UBE2B			
Q5JXB2	Putative ubiquitin-conjugating enzyme E2 N-like	UBE2NL			
A0A1B0GUS4	Ubiquitin-conjugating enzyme E2 L5	UBE2L5			
Q9UBW7	Zinc finger MYM-type protein 2	ZMYM2			
Q8N9F8	Zinc finger protein 454	ZNF454			
Q9UDV7	Zinc finger protein 282	ZNF282			
P0DPD5	Zinc finger protein 723	ZNF723			
Q7Z5L2	Coiled-coil domain-containing protein R3HCC1L	R3HCC1L			
P83731	60S ribosomal protein L24	RPL24			
Q6P5R6	60S ribosomal protein L22-like 1	RPL22L1			
Q9Y3B7	39S ribosomal protein L11, mitochondrial	MRPL11			
Q8NHW5	60S acidic ribosomal protein P0-like	RPLP0P6			
P19474	E3 ubiquitin-protein ligase	TRIM21			
Q9Y2L1	Exosome complex exonuclease RRP44	DIS3			
Q9P2E9	Ribosome-binding protein 1	RRBP1			
Q96LC7	Sialic acid-binding Ig-like lectin	SIGLEC10			
Q8N7X2	Protein STPG3	STPG3			
Q6ZMT1	SH3 and cysteine-rich domain-containing protein 2	STAC2			
Q8WWQ8	Stabilin-2	STAB2			
Q9NSY2	StAR-related lipid transfer protein 5	STARD5			
P52630	Signal transducer and activator of transcription 2	STAT2			
P49588	Alanine--tRNA ligase, cytoplasmic	AARS1			
O15260	Surfeit locus protein 4	SURF4			
Q7Z2R9	Putative uncharacterized protein SSBP3-AS1	SSBP3-AS1			
A0A1B0GWG4	Serine-rich and transmembrane domain-containing 2	SERTM2			
O95926	Pre-mRNA-splicing factor SYF2	SYF2			

Q9NSE4	Isoleucine--tRNA ligase, mitochondrial	IARS2			
P08247	Synaptophysin	SYP			
Q7Z7C8	Transcription initiation factor TFIID subunit 8	TAF8			
Q9BQ70	Transcription factor 25	TCF25			
P23258	Tubulin gamma-1 chain	TUBG1			
Q3ZCM7	Tubulin beta-8 chain	TUBB8			
Q9Y4R8	Telomere length regulation protein TEL2 homolog	TELO2			
Q9UKZ4	Teneurin-1	TENM1			
Q24JP5	Transmembrane protein 132A	TMEM132A			
Q96JJ7	Protein disulfide-isomerase TMX3	TMX3			
Q9BY14	Testis-expressed protein 101	TEX101			
Q96NA8	t-SNARE domain-containing protein 1	TSNARE1			
Q5TGU0	Translocator protein 2	TSPO2			
O60635	Tetraspanin-1	TSPAN1			
Q9UKR8	Tetraspanin-16	TSPAN16			
Q12999	Tetraspanin-31	TSPAN31			
A0A0K0K1D8	T cell receptor beta variable 6-1	TRBV6-1			
Q9H1C4	Protein unc-93 homolog B1	UNC93B1			
Q9UPW8	Protein unc-13 homolog A	UNC13A			
Q5TAP6	U3 small nucleolar RNA-associated protein 14 homolog C	UTP14C			
Q9P0L0	Vesicle-associated membrane protein-associated protein A	VAPA			
Q9NNX9	Variable charge X-linked protein 3	VCX3A			
O14599	Testis-specific basic protein Y 2	BPY2			
Q6ZMY6	WD repeat-containing protein 88	WDR88			
Q8WWY7	WAP four-disulfide core domain protein 12	WFDC12			
Q96AJ9	Vesicle transport through interaction with t-SNAREs homolog 1A	VTI1A			
Q5GFL6	von Willebrand factor A domain-containing protein 2	VWA2			
Q9BXE9	Vomeronasal type-1 receptor 3	VN1R3			
Q9H6R7	WD repeat and coiled-coil-containing protein	WDCP			
Q7Z5K2	Wings apart-like protein homolog	WAPL			
Q9HAD4	WD repeat-containing protein 41	WDR41			
Q9H7D7	WD repeat-containing protein 26	WDR26			
A6NIX2	Wilms tumor protein 1-interacting protein	WTIP			
Q9Y5W5	Wnt inhibitory factor 1	WIF1			
O14905	Protein Wnt-9b	WNT9B			
Q96NR7	Putative uncharacterized protein WWC2-AS2	WWC2-AS2			
Q9BYP7	Serine/threonine-protein kinase WNK3	WNK3			
Q9Y2G7	Zinc finger protein 30 homolog	ZFP30			
Q9H171	Z-DNA-binding protein 1	ZBP1			
P24278	Zinc finger and BTB domain-containing protein 25	ZBTB25			
O95159	Zinc finger protein-like 1	ZFPL1			
Q9NQZ6	Zinc finger C4H2 domain-containing protein	ZC4H2			
Q8N5P1	Zinc finger CCCH domain-containing protein 8	ZC3H8			
A6NFI3	Zinc finger protein 316	ZNF316			
Q9UC06	Zinc finger protein 70	ZNF70			
P0CG32	Zinc finger CCHC domain-containing protein 18	ZCCHC18			
A8MVS1	Zinc finger protein 705F	ZNF705F			
Q86UD4	Zinc finger protein 329	ZNF329			
Q8IVQ6	Palmitoyltransferase ZDHHC21	ZDHHC21			

Q96K62	Zinc finger and BTB domain-containing protein 45	ZBTB45			
Q16587	Zinc finger protein 74	ZNF74			
Q8N7K0	Zinc finger protein 433	ZNF433			
Q9ULD9	Zinc finger protein 608	ZNF608			
Q5H9K5	Zinc finger matrin-type protein 1	ZMAT1			
Q9H900	Protein zwilch homolog	ZWILCH			
O75152	Zinc finger CCCH domain-containing protein 11A	ZC3H11A			
Q86XN6	Zinc finger protein 761	ZNF761			
Q96C55	Zinc finger protein 524	ZNF524			
P59923	Zinc finger protein 445	ZNF445			
P28328	Peroxisome biogenesis factor 2	PEX2			
Q8NBL1	Protein O-glucosyltransferase 1	POGLUT1			
Q8ND90	Paraneoplastic antigen Ma1	PNMA1			
Q96FM1	Post-GPI attachment to proteins factor 3	PGAP3			
Q02809	Procollagen-lysine,2-oxoglutarate 5-dioxygenase 1	PLOD1			
Q9H4Q4	PR domain zinc finger protein 12	PRDM12			
Q70Z35	Phosphatidylinositol 3,4,5-trisphosphate-dependent Rac exchanger 2 protein	PREX2			
P30520	Adenylosuccinate synthetase isozyme 2	ADSS2			
P62266	40S ribosomal protein S23	RPS23			
Q13277	Syntaxin-3	STX3			
Q96H78	Solute carrier family 25 member 44	SLC25A44			
Q8TCC7	Solute carrier family 22 member 8	SLC22A8			
Q96GQ5	RUS family member 1	RUSF1			
Q96T68	Histone-lysine N-methyltransferase SETDB2	SETDB2			
A6NKW6	Protein shisa-like-2B	SHISAL2B			
O43147	Small G protein signaling modulator 2	SGSM2			
P0DME0	Protein SETSIP	SETSIP			
Q8WTS6	Histone-lysine N-methyltransferase SETD7	SETD7			
Q6UWP8	Suprabasin	SBSN			
Q93073	Selenocysteine insertion sequence-binding protein 2-like	SECISBP2L			
P37088	Amiloride-sensitive sodium channel subunit alpha	SCNN1A			
Q0VAQ4	Small cell adhesion glycoprotein	SMAGP			
Q5SXM2	snRNA-activating protein complex subunit 4	SNAPC4			
Q14683	Structural maintenance of chromosomes protein 1A	SMC1A			
Q5VUG0	Scm-like with four MBT domains protein 2	SFMBT2			
Q9H040	DNA-dependent metalloprotease SPRTN	SPRTN			
P62316	Small nuclear ribonucleoprotein Sm D2	SNRPD2			
P28370	Probable global transcription activator SNF2L1	SMARCA1			
A6NJY1	Putative SLC9B1-like protein SLC9B1P1	SLC9B1P1			
P09486	SPARC	SPARC			
Q9NS25	Sperm protein associated with the nucleus on the X chromosome B1	SPANXB1			
Q4W5P6	Small integral membrane protein 43	SMIM43			
Q9NYB5	Solute carrier organic anion transporter family member 1C1	SLCO1C1			
P0DUD1	Putative speedy protein E8	SPDYE8			
Q6ZQN7	Solute carrier organic anion transporter family member 4C1	SLCO4C1			

P0DUD3	Putative speedy protein E14	SPDYE14			
O94993	Transcription factor SOX-30	SOX30			
Q9NZD8	Maspardin	SPG21			
Q5TCZ1	SH3 and PX domain-containing protein 2A	SH3PXD2A			
P48595	Serpin B10	SERPINB10			
Q5T0L3	Spermatogenesis-associated protein 46	SPATA46			
Q6Q759	Sperm-associated antigen 17	SPAG17			
Q01082	Spectrin beta chain, non-erythrocytic 1	SPTBN1			
Q8IX21	SMC5-SMC6 complex localization factor protein 2	SLF2			
Q8TAQ9	SUN domain-containing protein 3	SUN3			
Q96N35	Putative uncharacterized protein encoded by LINC00052	LINC00052			
Q15208	Serine/threonine-protein kinase	STK38			
G2XKQ0	Small ubiquitin-related modifier 5	SUMO1P1			
P52823	Stanniocalcin-1	STC1			
Q9NRL3	Striatin-4	STRN4			
Q6ZRS2	Helicase SRCAP	SRCAP			
Q86TD4	Sarcalumenin	SRL			
A7MCY6	TANK-binding kinase 1-binding protein 1	TBKBP1			
Q13395	Probable methyltransferase TARBP1	TARBP1			
Q9Y5J9	Mitochondrial import inner membrane translocase subunit Tim8 B	TIMM8B			
Q9UPQ9	Trinucleotide repeat-containing gene 6B protein	TNRC6B			
Q8N2E6	Prosalusin	TOR2A			
P19429	Troponin I, cardiac muscle	TNNI3			
Q9ULW0	Targeting protein for Xklp2	TPX2			
Q15035	Translocating chain-associated membrane protein 2	TRAM2			
P83876	Thioredoxin-like protein 4A	TXNL4A			
Q9BTV4	Transmembrane protein 43	TMEM43			
Q9BZD7	Transmembrane gamma-carboxyglutamic acid protein 3	PRRG3			
Q86YD3	Transmembrane protein 25	TMEM25			
O14907	Tax1-binding protein 3	TAX1BP3			
P04818	Thymidylate synthase	TYMS			
Q99990	Transcription cofactor vestigial-like protein 1	VGLL1			
Q6UXI7	Vitrin	VIT			
Q9P2U8	Vesicular glutamate transporter 2	SLC17A6			
P41226	Ubiquitin-like modifier-activating enzyme 7	UBA7			
Q562E7	WD repeat-containing protein 81	WDR81			
Q5GH77	XK-related protein 3	XKR3			
P18887	DNA repair protein XRCC1	XRCC1			
Q96QK1	Vacuolar protein sorting-associated protein 35	VPS35			
P0C1S8	Wee1-like protein kinase 2	WEE2			
Q6PCB0	von Willebrand factor A domain-containing protein 1	VWA1			
Q16222	UDP-N-acetylhexosamine pyrophosphorylase	UAP1			
Q2TAL6	Brorin	VWC2			
Q9NQW7	Xaa-Pro aminopeptidase 1	XPNPEP1			
Q641Q2	WASH complex subunit 2A	WASHC2A			
P04275	von Willebrand factor	VWF			
Q9Y2I8	WD repeat-containing protein 37	WDR37			
P47989	Xanthine dehydrogenase/oxidase	XDH			
P56706	Protein Wnt-7b	WNT7B			
Q9GZT5	Protein Wnt-10a	WNT10A			
Q0VG73	Putative uncharacterized protein LOC152225				
Q5BJH7	Protein YIF1B	YIF1B			

Q5PR19	Putative UPF0607 protein LOC392364				
Q8N6K4	Putative uncharacterized protein MGC34800				
Q8NAQ8	Putative uncharacterized protein FLJ34945				
Q6ZN57	Zinc finger protein 2 homolog	ZFP2			
Q9NU63	Zinc finger protein 57 homolog	ZFP57			
Q8IXM3	39S ribosomal protein L41, mitochondrial	MRPL41			
Q2QD12	Ribulose-phosphate 3-epimerase-like protein 1	RPEL1			
Q9H777	Zinc phosphodiesterase ELAC protein 1	ELAC1			
Q9BWH6	RNA polymerase II-associated protein 1	RPAP1			
P62875	DNA-directed RNA polymerases I, II, and III subunit RPABC5	POLR2L			
Q8IYW5	E3 ubiquitin-protein ligase	RNF168			
Q96K19	E3 ubiquitin-protein ligase	RNF170			
Q9H2S5	RING finger protein 39	RNF39			
Q9UBS8	E3 ubiquitin-protein ligase RNF14	RNF14			
Q9NWF9	E3 ubiquitin-protein ligase	RNF216			
O00411	DNA-directed RNA polymerase, mitochondrial	POLRMT			
Q8WXD0	Relaxin receptor 2	RXFP2			
P78382	CMP-sialic acid transporter	SLC35A1			
A6NFY7	Succinate dehydrogenase assembly factor 1, mitochondrial	SDHAF1			
Q8TD22	Sideroflexin-5	SFXN5			
Q96PL1	Secretoglobin family 3A member	SCGB3A2			
Q5VUM1	Succinate dehydrogenase assembly factor 4, mitochondrial	SDHAF4			
Q13326	Gamma-sarcoglycan	SGCG			
Q15427	Splicing factor 3B subunit 4	SF3B4			
Q5JRV8	Transmembrane protein 255A	TMEM255A			
Q96T21	Selenocysteine insertion sequence-binding protein 2	SECISBP2			
Q587I9	Vesicle transport protein SFT2C	SFT2D3			
P35247	Pulmonary surfactant-associated protein D	SFTPD			
P98077	SHC-transforming protein 2	SHC2			
P84101	Small EDRK-rich factor 2	SERF2			
P49908	Selenoprotein P	SELENOP			
P31639	Sodium/glucose cotransporter 2	SLC5A2			
Q13228	Methanethiol oxidase	SELENBP1			
Q9UHD8	Septin-9	SEPTIN9			
A6NH21	Serine incorporator 4	SERINC4			
Q96FV2	Secernin-2	SCRN2			
Q9UPU9	Protein Smaug homolog 1	SAMD4A			
O14828	Secretory carrier-associated membrane protein 3	SCAMP3			
Q71RC9	Small integral membrane protein	SMIM5			
Q02086	Transcription factor Sp2	SP2			
Q9H0K1	Serine/threonine-protein kinase SIK2	SIK2			
P01241	Somatotropin	GH1			
A0A494C191	Putative speedy protein E9	SPDYE9			
Q9BWV2	Spermatogenesis-associated protein 9	SPATA9			
Q96AH0	SOSS complex subunit B2	NABP1			
Q8WY21	VPS10 domain-containing receptor SorCS1	SORCS1			
Q537H7	Spermatogenesis-associated protein 45	SPATA45			
O75635	Serpin B7	SERPINB7			
P03973	Antileukoproteinase	SLPI			
P11277	Spectrin beta chain, erythrocytic	SPTB			

Q68D10	Protein SPT2 homolog	SPTY2D1			
Q9NUV7	Serine palmitoyltransferase 3	SPTLC3			
Q16385	Protein SSX2	SSX2			
Q6IMI6	Sulfotransferase 1C3	SULT1C3			
Q7RTT6	Putative protein SSX6	SSX6P			
Q13586	Stromal interaction molecule 1	STIM1			
O95630	STAM-binding protein	STAMBP			
Q92502	StAR-related lipid transfer protein 8	STARD8			
P42226	Signal transducer and activator of transcription 6	STAT6			
O94888	UBX domain-containing protein 7	UBXN7			
Q9HBJ7	Ubiquitin carboxyl-terminal hydrolase 29	USP29			
P15374	Ubiquitin carboxyl-terminal hydrolase isozyme L3	UCHL3			
Q6DHY5	TBC1 domain family member 3G	TBC1D3G			
Q6ZVK1	Transmembrane protein 179	TMEM179			
Q9NVV9	THAP domain-containing protein 1	THAP1			
Q99598	Translin-associated protein X	TSNAX			
Q96T88	E3 ubiquitin-protein ligase UHRF1	UHRF1			
Q96MU6	Zinc finger protein 778	ZNF778			
Q2VY69	Zinc finger protein 284	ZNF284			
Q9HBT7	Zinc finger protein 287	ZNF287			
Q13398	Zinc finger protein 211	ZNF211			
Q9Y237	Peptidyl-prolyl cis-trans isomerase NIMA-interacting 4	PIN4			
O75151	Lysine-specific demethylase PHF2	PHF2			
P35354	Prostaglandin G/H synthase 2	PTGS2			
P10720	Platelet factor 4 variant	PF4V1			
Q9UHV9	Prefoldin subunit 2	PFDN2			
P08237	ATP-dependent 6-phosphofructokinase, muscle type	PFKM			
Q00169	Phosphatidylinositol transfer protein alpha isoform	PITPNA			
Q01813	ATP-dependent 6-phosphofructokinase, platelet type	PFKP			
P61758	Prefoldin subunit 3	VBP1			
Q9UKJ0	Paired immunoglobulin-like type 2 receptor beta	PILRB			
P15428	15-hydroxyprostaglandin dehydrogenase	HPGD			
Q01851	POU domain, class 4, transcription factor 1	POU4F1			
Q9H2H8	Peptidyl-prolyl cis-trans isomerase-like 3	PPIL3			
Q01453	Peripheral myelin protein 22	PMP22			
Q2NL68	Proline and serine-rich protein 3	PROSER3			
P16471	Prolactin receptor	PRLR			
P02810	Salivary acidic proline-rich phosphoprotein 1/2	PRH1;			
P86496	Proline-rich protein 20A	PRR20A			
D3DTV9	Putative protein PRAC2	PRAC2			
O75915	PRA1 family protein 3	ARL6IP5			
A3QJZ6	PRAME family member 22	PRAMEF22			
Q9UQ74	Pregnancy-specific beta-1-glycoprotein 8	PSG8			
P28066	Proteasome subunit alpha type-5	PSMA5			
P11464	Pregnancy-specific beta-1-glycoprotein 1	PSG1			
Q8N142	Adenylosuccinate synthetase isozyme 1	ADSS1			
Q99460	26S proteasome non-ATPase regulatory subunit 1	PSMD1			
Q92575	UBX domain-containing protein 4	UBXN4			
A6NDD5	Synapse differentiation-inducing gene protein 1-like	SYNDIG1L			
O14994	Synapsin-3	SYN3			

Q99593	T-box transcription factor TBX5	TBX5			
P49368	T-complex protein 1 subunit gamma	CCT3			
O75347	Tubulin-specific chaperone A	TBCA			
Q5H9I0	Transcription factor Dp family member 3	TFDP3			
Q92752	Tenascin-R	TNR			
Q8NBL3	Transmembrane protein 178A	TMEM178A			
Q15654	Thyroid receptor-interacting protein 6	TRIP6			
Q9Y296	Trafficking protein particle complex subunit 4	TRAPPC4			
Q9ULQ1	Two pore calcium channel protein 1	TPCN1			
Q8WW62	Transmembrane emp24 domain-containing protein 6	TMED6			
A0A0B4J23	T cell receptor alpha variable 1-2	TRAV1-2			
Q6EMB2	Tubulin polyglutamylase TTLL5	TTLL5			
Q969E8	Pre-rRNA-processing protein TSR2 homolog	TSR2			
A0A1B0GX68	T cell receptor beta variable 2	TRBV2			
Q6PHR2	Serine/threonine-protein kinase ULK3	ULK3			
Q9BSL1	Ubiquitin-associated domain-containing protein 1	UBAC1			
Q7Z6J8	E3 ubiquitin-protein ligase E3D	UBE3D			
Q70EL3	Inactive ubiquitin carboxyl-terminal hydrolase 50	USP50			
C9JJH3	Ubiquitin carboxyl-terminal hydrolase 17-like protein 10	USP17L10			
A8MUK1	Ubiquitin carboxyl-terminal hydrolase 17-like protein 5	USP17L5			
Q8IYU4	Ubiquilin-like protein	UBQLNL			
Q14119	Vascular endothelial zinc finger 1	VEZF1			
P61077	Ubiquitin-conjugating enzyme E2 D3	UBE2D3			
Q9HCJ6	Synaptic vesicle membrane protein VAT-1 homolog-like	VAT1L			
P63027	Vesicle-associated membrane protein 2	VAMP2			
A4D1P6	WD repeat-containing protein 91	WDR91			
Q6UX68	XK-related protein 5	XKR5			
O14609	Testis-specific XK-related protein, Y-linked	XKRY			
Q6P2D8	X-ray radiation resistance-associated protein 1	XRRA1			
O00401	Neural Wiskott-Aldrich syndrome protein	WASL			
Q8TAF3	WD repeat-containing protein 48	WDR48			
Q9HD64	X antigen family member 1	XAGE1A			
O00744	Protein Wnt-10b	WNT10B			
Q5MNZ9	WD repeat domain phosphoinositide-interacting protein 1	WIPI1			
O15498	Synaptobrevin homolog YKT6	YKT6			
Q9H354	Putative uncharacterized protein PRO1933	PRO1933			
Q6ZQY7	Putative uncharacterized protein FLJ46792				
Q8N6M9	AN1-type zinc finger protein 2A	ZFAND2A			
P10072	Zinc finger protein 875	ZNF875			
Q8N0V1	Putative uncharacterized protein ZNF295-AS1	ZNF295-AS1			
O95405	Zinc finger FYVE domain-containing protein 9	ZFYVE9			
P17017	Zinc finger protein 14	ZNF14			
Q15937	Zinc finger protein 79	ZNF79			

P17040	Zinc finger and SCAN domain-containing protein 20	ZSCAN20			
Q7Z2W4	Zinc finger CCCH-type antiviral protein 1	ZC3HAV1			
Q7Z3V5	Zinc finger protein 571	ZNF571			
A0A0U1RRA0	Putative transmembrane protein ZNF593OS	ZNF593OS			
P43403	Tyrosine-protein kinase ZAP-70	ZAP70			
O95218	Zinc finger Ran-binding domain-containing protein 2	ZRANB2			
Q8TAD4	Zinc transporter 5	SLC30A5			
Q6ZNH5	Zinc finger protein 497	ZNF497			
O95365	Zinc finger and BTB domain-containing protein 7A	ZBTB7A			
Q8TBZ8	Zinc finger protein 564	ZNF564			
Q9P2E3	NFX1-type zinc finger-containing protein 1	ZNFX1			
Q14585	Zinc finger protein 345	ZNF345			
Q9Y6R6	Zinc finger protein 780B	ZNF780B			
Q9H0C1	Zinc finger MYND domain-containing protein 12	ZMYND12			
Q9Y2L8	Zinc finger protein with KRAB and SCAN domains 5	ZKSCAN5			
Q5VZL5	Zinc finger MYM-type protein 4	ZMYM4			
Q96MR9	Zinc finger protein 560	ZNF560			
Q9Y6M5	Zinc transporter 1	SLC30A1			
Q8N782	Zinc finger protein 525	ZNF525			
Q13360	Zinc finger protein 177	ZNF177			
Q8TF20	Zinc finger protein 721	ZNF721			
Q9BS34	Zinc finger protein 670	ZNF670			
Q9HCE3	Zinc finger protein 532	ZNF532			
Q8NGJ0	Olfactory receptor 5A1	OR5A1			
P36957	Dihydrolipoyllysine-residue succinyltransferase component of 2-oxoglutarate dehydrogenase complex, mitochondrial	DLST			
Q01804	OTU domain-containing protein 4	OTUD4			
Q96DC9	Ubiquitin thioesterase OTUB2	OTUB2			
Q13606	Olfactory receptor 5I1	OR5I1			
Q53FV1	ORM1-like protein 2	ORMDL2			
Q96RR1	Twinkle protein, mitochondrial	TWNK			
Q15198	Platelet-derived growth factor receptor-like protein	PDGFRL			
Q8TC44	POC1 centriolar protein homolog B	POC1B			
Q96AD5	Patatin-like phospholipase domain-containing protein 2	PNPLA2			
Q75T13	GPI inositol-deacylase	PGAP1			
P62841	40S ribosomal protein S15	RPS15			
Q86SK9	Stearoyl-CoA desaturase 5	SCD5			
Q969E2	Secretory carrier-associated membrane protein 4	SCAMP4			
Q86W54	Spermatogenesis-associated protein 24	SPATA24			
Q6ZMJ2	Scavenger receptor class A member 5	SCARA5			
Q3KNW1	Zinc finger protein SNAI3	SNAI3			
P0DJ93	Small integral membrane protein 13	SMIM13			
Q9H930	Nuclear body protein SP140-like protein	SP140L			
A0A1B0GU29	Small integral membrane protein 28	SMIM28			
A1X283	SH3 and PX domain-containing protein 2B	SH3PXD2B			
Q9NXA8	NAD-dependent protein deacylase sirtuin-5, mitochondrial	SIRT5			
Q2M3C7	A-kinase anchor protein SPHKAP	SPHKAP			
P36952	Serpin B5	SERPINB5			

P50453	Serpin B9	SERPINB9			
Q8WYL5	Protein phosphatase Slingshot homolog 1	SSH1			
P60508	Syncytin-2	ERVFRD-1			
A6NGW2	Putative stereocilin-like protein	STRCP1			
Q96I99	Succinate--CoA ligase	SUCLG2			
Q8IX01	SURP and G-patch domain-containing protein 2	SUGP2			
Q96SB4	SRSF protein kinase 1	SRPK1			
P14868	Aspartate--tRNA ligase, cytoplasmic	DARS1			
Q8IYB8	ATP-dependent RNA helicase SUPV3L1, mitochondrial	SUPV3L1			
O60602	Toll-like receptor 5	TLR5			
P0DI82	Trafficking protein particle complex subunit 2B	TRAPPC2B			
A0A0B4J27	T cell receptor alpha variable 17	TRAV17			
A0A0A6YYD4	T cell receptor beta variable 13	TRBV13			
P41732	Tetraspanin-7	TSPAN7			
Q9BZM6	UL16-binding protein 1	ULBP1			
Q8NFU3	Thiosulfate:glutathione sulfurtransferase	TSTD1			
Q9Y3C8	Ubiquitin-fold modifier-conjugating enzyme 1	UFC1			
Q8TB05	UBA-like domain-containing protein 1	UBALD1			
Q5T6F2	Ubiquitin-associated protein 2	UBAP2			
P62760	Visinin-like protein 1	VSNL1			
Q9Y3A2	Probable U3 small nucleolar RNA-associated protein 11	UTP11			
Q9Y484	WD repeat domain phosphoinositide-interacting protein 4	WDR45			
O43516	WAS/WASL-interacting protein family member 1	WIPF1			
P67809	Y-box-binding protein 1	YBX1			
Q9H4A3	Serine/threonine-protein kinase WNK1	WNK1			
Q9P1C3	Putative uncharacterized protein PRO2829	PRO2829			
Q15942	Zyxin	ZYX			
Q8ND82	Zinc finger protein 280C	ZNF280C			
Q96KR1	Zinc finger RNA-binding protein	ZFR			
O60290	Zinc finger protein 862	ZNF862			
Q9UGR2	Zinc finger CCCH domain-containing protein 7B	ZC3H7B			
Q9HCK0	Zinc finger and BTB domain-containing protein 26	ZBTB26			
P0CG24	Zinc finger protein 883	ZNF883			
Q9Y3S2	Zinc finger protein 330	ZNF330			
A4D1E1	Zinc finger protein 804B	ZNF804B			
Q6ZN55	Zinc finger protein 574	ZNF574			
Q9NUA8	Zinc finger and BTB domain-containing protein 40	ZBTB40			
Q9BR11	Zinc finger SWIM domain-containing protein 1	ZSWIM1			
Q3ZCX4	Zinc finger protein 568	ZNF568			
Q9ULT6	E3 ubiquitin-protein ligase ZNRF3	ZNRF3			
Q32MQ0	Zinc finger protein 750	ZNF750			
Q8TF68	Zinc finger protein 384	ZNF384			
P17032	Zinc finger protein 37A	ZNF37A			
Q99676	Zinc finger protein 184	ZNF184			
Q9ULC8	Palmitoyltransferase ZDHHC8	ZDHHC8			
Q9HCJ5	Zinc finger SWIM domain-containing protein 6	ZSWIM6			
Q4FZB7	Histone-lysine N-methyltransferase KMT5B	KMT5B			

A0A075B6S2	Immunoglobulin kappa variable 2D-29	IGKV2D-29			
A0A075B6R9	Probable non-functional immunoglobulin kappa variable 2D-24	IGKV2D-24			
A0A087WSY6	Immunoglobulin kappa variable 3D-15	IGKV3D-15			
Q5TGZ0	MICOS complex subunit MIC10	MICOS10			
P53778	Mitogen-activated protein kinase 12	MAPK12			
Q15049	Membrane protein MLC1	MLC1			
Q96PD6	2-acylglycerol O-acyltransferase 1	MOGAT1			
Q8N565	Melanoregulin	MREG			
Q7RTY1	Monocarboxylate transporter 9	SLC16A9			
O15440	Multidrug resistance-associated protein 5	ABCC5			
Q9Y217	Myotubularin-related protein 6	MTMR6			
O95248	Myotubularin-related protein 5	SBF1			
P22033	Methylmalonyl-CoA mutase, mitochondrial	MMUT			
Q9H4K7	Mitochondrial ribosome-associated GTPase 2	MTG2			
O75648	Mitochondrial tRNA-specific 2-thiouridylase 1	TRMU			
P59046	NACHT, LRR and PYD domains-containing protein 12	NLRP12			
Q92614	Unconventional myosin-XVIIIa	MYO18A			
Q9BRK3	Matrix remodeling-associated protein 8	MXRA8			
Q86TC9	Myopalladin	MYPN			
Q9UMX5	Neudesin	NENF			
Q5SWX8	Protein odr-4 homolog	ODR4			
Q7RTS6	Proton channel OTOP2	OTOP2			
P32243	Homeobox protein OTX2	OTX2			
Q9HBI1	Beta-parvin	PARVB			
Q5T6S3	PHD finger protein 19	PHF19			
P0DJD8	Pepsin A-3	PGA3			
Q32M88	Protein-glucosylgalactosylhydroxylysine glucosidase	PGGHG			
P41247	Patatin-like phospholipase domain-containing protein 4	PNPLA4			
P28749	Retinoblastoma-like protein 1	RBL1			
B7ZAP0	Rab GTPase-activating protein 1-like, isoform 10	RABGAP1L			
P61225	Ras-related protein Rap-2b	RAP2B			
Q70E73	Ras-associated and pleckstrin homology domains-containing protein 1	RAPH1			
O75916	Regulator of G-protein signaling 9	RGS9			
Q9HAU5	Regulator of nonsense transcripts 2	UPF2			
P27635	60S ribosomal protein L10	RPL10			
P83881	60S ribosomal protein L36a	RPL36A			
Q9ULX5	RING finger protein 112	RNF112			
Q8IUD6	E3 ubiquitin-protein ligase	RNF135			
Q8TCC3	39S ribosomal protein L30, mitochondrial	MRPL30			
Q8NH48	Olfactory receptor 5B3	OR5B3			
Q6ZRI0	Otogelin	OTOG			
P04001	Medium-wave-sensitive opsin 1	OPN1MW			
Q5BJF6	Outer dense fiber protein 2	ODF2			
P04181	Ornithine aminotransferase, mitochondrial	OAT			
Q8NG78	Olfactory receptor 8G5	OR8G5			
P47712	Cytosolic phospholipase A2	PLA2G4A			
O75340	Programmed cell death protein 6	PDCD6			
Q96G03	Phosphoglucomutase-2	PGM2			
Q6ZV29	Patatin-like phospholipase domain-containing protein 7	PNPLA7			

Q9NPH0	Lysophosphatidic acid phosphatase type 6	ACP6			
Q9Y255	PRELI domain-containing protein 1, mitochondrial	PRELID1			
P60891	Ribose-phosphate pyrophosphokinase 1	PRPS1			
Q9NV39	Proline-rich protein 34	PRR34			
Q5JRX3	Presequence protease, mitochondrial	PITRM1			
Q16821	Protein phosphatase 1 regulatory subunit 3A	PPP1R3A			
P06454	Prothymosin alpha	PTMA			
Q9UDW1	Cytochrome b-c1 complex subunit 9	UQCR10			
Q05519	Serine/arginine-rich splicing factor 11	SRSF11			
Q9BVJ6	U3 small nucleolar RNA-associated protein 14 homolog A	UTP14A			
Q92797	Symplekin	SYMPK			
Q9Y228	TRAF3-interacting JNK-activating modulator	TRAF3IP3			
Q5VWN6	Protein TASOR 2	TASOR2			
P78371	T-complex protein 1 subunit beta	CCT2			
Q8IV04	Carabin	TBC1D10C			
Q9UBB9	Tuftelin-interacting protein 11	TFIP11			
Q5JTD0	Tight junction-associated protein 1	TJAP1			
A0A0J9YXA8	T cell receptor beta joining 1-1	TRBJ1-1			
P29401	Transketolase	TKT			
P51864	Putative teratocarcinoma-derived growth factor 3	TDGF1P3			
Q15562	Transcriptional enhancer factor TEF-4	TEAD2			
Q6P9G4	Transmembrane protein 154	TMEM154			
Q9NV64	Transmembrane protein 39A	TMEM39A			
Q8WVE6	Transmembrane protein 171	TMEM171			
Q9UNG2	Tumor necrosis factor ligand superfamily member 18	TNFSF18			
Q15643	Thyroid receptor-interacting protein 11	TRIP11			
A0A0B4J26	T cell receptor alpha variable 41	TRAV41			
P0C672	Tetraspanin-19	TSPAN19			
Q9ULT0	Tetratricopeptide repeat protein 7A	TTC7A			
A0A0A6YYJ7	T cell receptor alpha variable 8-3	TRAV8-3			
A0A1B0GX51	T cell receptor beta variable 7-8	TRBV7-8			
O95164	Ubiquitin-like protein 3	UBL3			
Q9Y4E8	Ubiquitin carboxyl-terminal hydrolase 15	USP15			
Q8N7F7	Ubiquitin-like protein 4B	UBL4B			
Q9BQ65	U6 snRNA phosphodiesterase	USB1			
P49765	Vascular endothelial growth factor B	VEGFB			
P15313	V-type proton ATPase subunit B, kidney isoform	ATP6V1B1			
P16989	Y-box-binding protein 3	YBX3			
Q8N1X5	Uncharacterized protein FLJ37310				
Q499Y3	Putative uncharacterized protein C10orf88-like				
O43149	Zinc finger ZZ-type and EF-hand domain-containing protein 1	ZZEF1			
Q9HCL3	Zinc finger protein 14 homolog	ZFP14			
O60844	Zymogen granule membrane protein 16	ZG16			
Q08AG5	Zinc finger protein 844	ZNF844			
O95049	Tight junction protein ZO-3	TJP3			

Q96KM6	Zinc finger protein 512B	ZNF512B			
O15209	Zinc finger and BTB domain-containing protein 22	ZBTB22			
P0C7X2	Zinc finger protein 688	ZNF688			
Q9H898	Zinc finger matrin-type protein 4	ZMAT4			
Q5HYM0	Probable ribonuclease ZC3H12B	ZC3H12B			
Q9NXT0	Zinc finger protein 586	ZNF586			
Q6PK81	Zinc finger protein 773	ZNF773			
Q14588	Zinc finger protein 234	ZNF234			
Q9UID6	Zinc finger protein 639	ZNF639			
Q6PI48	Aspartate--tRNA ligase, mitochondrial	DARS2			
P21675	Transcription initiation factor TFIID subunit 1	TAF1			
Q7RTU0	Transcription factor 24	TCF24			
A6NKL6	Transmembrane protein 200C	TMEM200C			
Q9UHD2	Serine/threonine-protein kinase TBK1	TBK1			
Q7Z7G0	Target of Nesh-SH3	ABI3BP			
Q5VWI1	Transcription elongation regulator 1-like protein	TCERG1L			
Q8WV15	Transmembrane protein 255B	TMEM255B			
Q8N3T6	Transmembrane protein 132C	TMEM132C			
Q9BXF9	Tektin-3	TEKT3			
O96008	Mitochondrial import receptor subunit TOM40 homolog	TOMM40			
O14763	Tumor necrosis factor receptor superfamily member 10B	TNFRSF10B			
P20333	Tumor necrosis factor receptor superfamily member 1B	TNFRSF1B			
Q6P2S7	Putative tetratricopeptide repeat protein 41	TTC41P			
P07996	Thrombospondin-1	THBS1			
Q9BZM4	UL16-binding protein 3	ULBP3			
Q6H3X3	UL-16 binding protein 5	RAET1G			
Q9H313	Protein tweety homolog 1	TTYH1			
Q8IU68	Transmembrane channel-like protein 8	TMC8			
A6NMA1	Putative uncharacterized protein TRPC5OS	TRPC5OS			
Q96HZ7	Putative uncharacterized protein URB1-AS1	URB1-AS1			
Q9Y5T5	Ubiquitin carboxyl-terminal hydrolase 16	USP16			
P35503	UDP-glucuronosyltransferase 1A3	UGT1A3			
Q9HA47	Uridine-cytidine kinase 1	UCK1			
A2RUC4	tRNA wybutosine-synthesizing protein 5	TYW5			
P62253	Ubiquitin-conjugating enzyme E2 G1	UBE2G1			
P49427	Ubiquitin-conjugating enzyme E2 R1	CDC34			
P19320	Vascular cell adhesion protein 1	VCAM1			
P52735	Guanine nucleotide exchange factor VAV2	VAV2			
P63123	Endogenous retrovirus group K member 18 Pro protein	ERVK-18			
Q9UM01	Y+L amino acid transporter 1	SLC7A7			
Q86YA3	Protein ZGRF1	ZGRF1			
Q9C0D3	Protein zyg-11 homolog B	ZYG11B			
Q8N8P6	Putative uncharacterized protein FLJ39060				
Q3KP31	Zinc finger protein 791	ZNF791			
P17014	Zinc finger protein 12	ZNF12			
Q96IR2	Zinc finger protein 845	ZNF845			
Q6ZN19	Zinc finger protein 841	ZNF841			
Q96EG3	Zinc finger protein 837	ZNF837			
Q147U1	Zinc finger protein 846	ZNF846			
P17021	Zinc finger protein 17	ZNF17			

Q96BR9	Zinc finger and BTB domain-containing protein 8A	ZBTB8A			
Q19AV6	Zinc finger SWIM domain-containing protein 7	ZSWIM7			
Q5T0B9	Zinc finger protein 362	ZNF362			
O60304	Zinc finger protein 500	ZNF500			
Q9UK10	Zinc finger protein 225	ZNF225			
Q5W0Z9	Palmitoyltransferase ZDHHC20	ZDHHC20			
Q96CX3	Zinc finger protein 501	ZNF501			
Q9BS86	Zona pellucida-binding protein 1	ZPBP			
Q494X3	Zinc finger protein 404	ZNF404			
Q6ZNG0	Zinc finger protein 620	ZNF620			
Q6NXT4	Zinc transporter 6	SLC30A6			
Q8WXB4	Zinc finger protein 606	ZNF606			
Q8NGL2	Olfactory receptor 5L1	OR5L1			
Q92823	Neuronal cell adhesion molecule	NRCAM			
A6NF89	Olfactory receptor 6C6	OR6C6			
Q6ZS82	Regulator of G-protein signaling 9-binding protein	RGS9BP			
Q9Y3T6	R3H and coiled-coil domain-containing protein 1	R3HCC1			
P17600	Synapsin-1	SYN1			
P54136	Arginine--tRNA ligase, cytoplasmic	RARS1			
Q14157	Ubiquitin-associated protein 2-	UBAP2L			
Q9UL54	Serine/threonine-protein kinase TAO2	TAOK2			
Q53T94	TATA box-binding protein-associated factor RNA polymerase I subunit B	TAF1B			
Q5T4T1	Transmembrane protein 170B	TMEM170B			
P0DTE0	Putative taste receptor type 2 member 36	TAS2R36			
Q86V40	Metalloprotease TIKI1	TRABD2A			
Q8NA92	THAP domain-containing protein 8	THAP8			
Q04724	Transducin-like enhancer protein	TLE1			
Q04727	Transducin-like enhancer protein	TLE4			
Q9NX78	Transmembrane protein 260	TMEM260			
Q5BJD5	Transmembrane protein 41B	TMEM41B			
Q9H0V1	Transmembrane protein 168	TMEM168			
A0A1B0GTI	Transmembrane protein 272	TMEM272			
A2RU14	Transmembrane protein 218	TMEM218			
Q8WUH2	Transforming growth factor-beta receptor-associated protein 1	TGFBRAP1			
Q5SNT2	Transmembrane protein 201	TMEM201			
Q9H6L2	Transmembrane protein 231	TMEM231			
Q96F44	E3 ubiquitin-protein ligase	TRIM11			
P36406	E3 ubiquitin-protein ligase	TRIM23			
P56180	Putative tyrosine-protein phosphatase TPTE	TPTE			
Q07011	Tumor necrosis factor receptor superfamily member 9	TNFRSF9			
Q7Z392	Trafficking protein particle complex subunit 11	TRAPPC11			
Q9H892	Tetratricopeptide repeat protein 12	TTC12			
A0A075B6V5	T cell receptor alpha variable 36/delta variable 7	TRAV36DV7			
Q15819	Ubiquitin-conjugating enzyme E2 variant 2	UBE2V2			
D6RCP7	Ubiquitin carboxyl-terminal hydrolase 17-like protein 19	USP17L19			
Q5T230	Undifferentiated embryonic cell transcription factor 1	UTF1			
O95292	Vesicle-associated membrane protein-associated protein B/C	VAPB			
Q9NW82	WD repeat-containing protein 70	WDR70			

Q5MNZ6	WD repeat domain phosphoinositide-interacting protein 3	WDR45B			
O75554	WW domain-binding protein 4	WBP4			
O14904	Protein Wnt-9a	WNT9A			
A8MVM7	Putative uncharacterized protein ENSP00000382790				
Q9UJW7	Zinc finger protein 229	ZNF229			
P0DKX0	Zinc finger protein 728	ZNF728			
Q8TAW3	Zinc finger protein 671	ZNF671			
P57082	T-box transcription factor TBX4	TBX4			
Q7Z5A8	Chemokine-like protein TAFA-3	TAFA3			
P59544	Taste receptor type 2 member 50	TAS2R50			
Q9NQ88	Fructose-2,6-bisphosphatase TIGAR	TIGAR			
Q9NR96	Toll-like receptor 9	TLR9			
P01266	Thyroglobulin	TG			
Q9NXG2	THUMP domain-containing protein 1	THUMPD1			
P31483	Nucleolysin TIA-1 isoform p40	TIA1			
Q9BU02	Thiamine-triphosphatase	THTPA			
Q8NA77	Testis-expressed protein 19	TEX19			
Q7Z5S9	Transmembrane protein 144	TMEM144			
P0DJG4	Testicular haploid expressed gene protein-like	THEGL			
Q9HCS4	Transcription factor 7-like 1	TCF7L1			
H0YL14	Transmembrane protein 250	TMEM250			
P51854	Transketolase-like protein 1	TKTL1			
Q96MN5	Transcription elongation factor A N-terminal and central domain-containing protein 2	TCEANC2			
Q96PF1	Protein-glutamine gamma-glutamyltransferase Z	TGM7			
Q6QAJ8	Transmembrane protein 220	TMEM220			
Q24JQ0	Transmembrane protein 241	TMEM241			
B5MCY1	Tudor domain-containing protein 15	TDRD15			
Q9H6F2	Trimeric intracellular cation channel type A	TMEM38A			
P56557	Transmembrane protein 50B	TMEM50B			
P49221	Protein-glutamine gamma-glutamyltransferase 4	TGM4			
Q9NZC2	Triggering receptor expressed on myeloid cells 2	TREM2			
O75382	Tripartite motif-containing protein 3	TRIM3			
O60507	Protein-tyrosine sulfotransferase 1	TPST1			
O43508	Tumor necrosis factor ligand superfamily member 12	TNFSF12			
Q8NG06	E3 ubiquitin-protein ligase	TRIM58			
Q15650	Activating signal cointegrator 1	TRIP4			
Q9HC21	Mitochondrial thiamine pyrophosphate carrier	SLC25A19			
O95985	DNA topoisomerase 3-beta-1	TOP3B			
O14836	Tumor necrosis factor receptor superfamily member 13B	TNFRSF13B			
Q15363	Transmembrane emp24 domain-containing protein 2	TMED2			
P40238	Thrombopoietin receptor	MPL			
Q8N9X5	Putative transmembrane protein 75	TMEM75			
Q8N3L3	Beta-taxilin	TXLNB			
P22735	Protein-glutamine gamma-glutamyltransferase K	TGM1			
Q96B42	Transmembrane protein 18	TMEM18			
P17643	5,6-dihydroxyindole-2-carboxylic acid oxidase	TYRP1			
O75954	Tetraspanin-9	TSPAN9			
A1L157	Tetraspanin-11	TSPAN11			

P01222	Thyrotropin subunit beta	TSHB			
Q8IV45	UNC5C-like protein	UNC5CL			
Q8N2C7	Protein unc-80 homolog	UNC80			
Q8NBZ7	UDP-glucuronic acid decarboxylase 1	UXS1			
Q2NL98	Vimentin-type intermediate filament-associated coiled-coil protein	VMAC			
P22314	Ubiquitin-like modifier-activating enzyme 1	UBA1			
Q7Z7E8	Ubiquitin-conjugating enzyme E2 Q1	UBE2Q1			
Q9BV40	Vesicle-associated membrane protein 8	VAMP8			
Q8NEX6	Protein WFDC11	WFDC11			
P02774	Vitamin D-binding protein	GC			
Q8TEU8	WAP, Kazal, immunoglobulin, Kunitz and NTR domain-containing protein 2	WFIKKN2			
Q8IWB7	WD repeat and FYVE domain-containing protein 1	WDFY1			
O75717	WD repeat and HMG-box DNA-binding protein 1	WDHD1			
O75063	Glycosaminoglycan xylosylkinase	FAM20B			
Q9Y6F9	Protein Wnt-6	WNT6			
Q6ZS46	Putative uncharacterized protein FLJ45840				
Q969W8	Zinc finger protein 566	ZNF566			
Q8N9Z0	Zinc finger protein 610	ZNF610			
Q6ZR52	Zinc finger protein 493	ZNF493			
Q3MIS6	Zinc finger protein 528	ZNF528			
Q9H7R5	Zinc finger protein 665	ZNF665			
Q6ZMN7	PDZ domain-containing RING finger protein 4	PDZRN4			
Q15031	Probable leucine--tRNA ligase, mitochondrial	LARS2			
O95045	Uridine phosphorylase 2	UPP2			
Q5VXT5	Synaptophysin-like protein 2	SYPL2			
O15178	T-box transcription factor T	TBXT			
Q9NYW7	Taste receptor type 2 member 1	TAS2R1			
P36402	Transcription factor 7	TCF7			
Q15544	Transcription initiation factor TFIID subunit 11	TAF11			
P68371	Tubulin beta-4B chain	TUBB4B			
Q03519	Antigen peptide transporter 2	TAP2			
Q8TDR4	T-complex protein 10A homolog 1	TCP10L			
Q6P1N9	Putative deoxyribonuclease TATDN1	TATDN1			
P68366	Tubulin alpha-4A chain	TUBA4A			
Q9HC07	Transmembrane protein 165	TMEM165			
Q86UV6	Tripartite motif-containing protein 74	TRIM74			
Q9UBP6	tRNA	METTL1			
P01850	T cell receptor beta constant 1	TRBC1			
Q6P9F5	E3 ubiquitin ligase TRIM40	TRIM40			
P57727	Transmembrane protease serine 3	TMPRSS3			
O75365	Protein tyrosine phosphatase type IVA 3	PTP4A3			
A5PLN9	Trafficking protein particle complex subunit 13	TRAPPC13			
A0A1B0GX56	T cell receptor delta variable 1	TRDV1			
P23510	Tumor necrosis factor ligand superfamily member 4	TNFSF4			
Q8N6T0	Type 2 DNA topoisomerase 6 subunit B-like	TOP6BL			
Q96NM4	TOX high mobility group box family member 2	TOX2			

P59282	Tubulin polymerization-promoting protein family member 2	TPPP2			
O43617	Trafficking protein particle complex subunit 3	TRAPPC3			
Q9H497	Torsin-3A	TOR3A			
Q9Y3B3	Transmembrane emp24 domain-containing protein 7	TMED7			
O75509	Tumor necrosis factor receptor superfamily member 21	TNFRSF21			
Q2TAA8	Translin-associated factor X-interacting protein 1	TSNAXIP1			
O95857	Tetraspanin-13	TSPAN13			
A0JNW5	UHRF1-binding protein 1-like	UHRF1BP1L			
A8MV65	Transcription cofactor vestigial-like protein 3	VGLL3			
Q5THJ4	Vacuolar protein sorting-associated protein 13D	VPS13D			
Q52LC2	V-type proton ATPase subunit S1-like protein	ATP6AP1L			
A8MX80	Putative UPF0607 protein ENSP00000383144				
Q8TF47	Zinc finger protein 90 homolog	ZFP90			
Q6ZN06	Zinc finger protein 813	ZNF813			
Q5CZA5	Zinc finger protein 805	ZNF805			
O43298	Zinc finger and BTB domain-containing protein 43	ZBTB43			
Q99592	Zinc finger and BTB domain-containing protein 18	ZBTB18			
P25311	Zinc-alpha-2-glycoprotein	AZGP1			
Q15935	Zinc finger protein 77	ZNF77			
Q8TBK6	Zinc finger CCHC domain-containing protein 10	ZCCHC10			
Q6AHZ1	Zinc finger protein 518A	ZNF518A			
Q15929	Putative zinc finger protein 56	ZNF56			
Q86VK4	Zinc finger protein 410	ZNF410			
Q96SK3	Zinc finger protein 607	ZNF607			
Q96GR4	Palmitoyltransferase ZDHHC12	ZDHHC12			
Q8N823	Zinc finger protein 611	ZNF611			
Q8TAF7	Zinc finger protein 461	ZNF461			
O60293	Zinc finger C3H1 domain-containing protein	ZFC3H1			
Q9UQR1	Zinc finger protein 148	ZNF148			
Q96LW9	Zinc finger and SCAN domain-containing protein 31	ZSCAN31			
Q9H8X9	Palmitoyltransferase ZDHHC11	ZDHHC11			
Q8ND25	E3 ubiquitin-protein ligase ZNRF1	ZNRF1			
O15014	Zinc finger protein 609	ZNF609			
Q14590	Zinc finger protein 235	ZNF235			
Q9BRI3	Zinc transporter 2	SLC30A2			
Q5H9L4	Transcription initiation factor TFIID subunit 7-like	TAF7L			
O75528	Transcriptional adapter 3	TADA3			
P30408	Transmembrane 4 L6 family member 1	TM4SF1			
Q8TEA7	TBC domain-containing protein kinase-like protein	TBCK			
Q9Y4C2	TRPM8 channel-associated factor 1	TCAF1			
Q9BXQ6	Transmembrane protein 121B	TMEM121B			
P59534	Taste receptor type 2 member 39	TAS2R39			
Q14C87	Transmembrane protein 132D	TMEM132D			
Q9NZI6	Transcription factor CP2-like protein 1	TFCP2L1			
Q6ZUX3	TOG array regulator of axonemal microtubules protein 2	TOGARAM2			
Q9H7E2	Tudor domain-containing protein 3	TDRD3			
Q2WGJ8	Transmembrane protein 249	TMEM249			
Q8N2U0	Transmembrane protein 256	TMEM256			

Q8N6L7	Transmembrane protein 252	TMEM252			
P61165	Transmembrane protein 258	TMEM258			
Q8N614	Transmembrane protein 156	TMEM156			
Q5VZ19	Tudor domain-containing protein 10	TDRD10			
Q6UW68	Transmembrane protein 205	TMEM205			
A6NGA9	Transmembrane protein 202	TMEM202			
Q969S6	Transmembrane protein 203	TMEM203			
Q96GX1	Tectonic-2	TCTN2			
P14373	Zinc finger protein RFP	TRIM27			
Q6PIZ9	T-cell receptor-associated transmembrane adapter 1	TRAT1			
O94900	Thymocyte selection-associated high mobility group box protein TOX	TOX			
Q7Z4G4	tRNA	TRMT11			
Q96KB5	Lymphokine-activated killer T-cell-originated protein kinase	PBK			
P29144	Tripeptidyl-peptidase 2	TPP2			
P48788	Troponin I, fast skeletal muscle	TNNI2			
P0DMS9	Transmembrane domain-containing protein TMIGD3	TMIGD3			
Q92956	Tumor necrosis factor receptor superfamily member 14	TNFRSF14			
Q02880	DNA topoisomerase 2-beta	TOP2B			
Q15361	Transcription termination factor 1	TTF1			
A0A0B4J23	T cell receptor alpha variable 8-2	TRAV8-2			
Q96C45	Serine/threonine-protein kinase ULK4	ULK4			
C9J7I0	UBAP1-MVB12-associated	UMAD1			
Q6NVU6	Inactive Ufm1-specific protease 1	UFSP1			
Q8TAG6	Vexin	VXN			
P46939	Utrophin	UTRN			
Q7Z5L0	Vitelline membrane outer layer protein 1 homolog	VMO1			
O00507	Probable ubiquitin carboxyl-terminal hydrolase FAF-Y	USP9Y			
Q96LB4	V-type proton ATPase subunit G 3	ATP6V1G3			
Q9Y2B5	VPS9 domain-containing protein	VPS9D1			
Q7Z5H5	Vomeronasal type-1 receptor 4	VN1R4			
O75083	WD repeat-containing protein 1	WDR1			
Q9BV38	WD repeat-containing protein 18	WDR18			
Q9P2S5	WD repeat-containing protein WRAP73	WRAP73			
O96014	Protein Wnt-11	WNT11			
P56704	Protein Wnt-3a	WNT3A			
Q9NZC7	WW domain-containing oxidoreductase	WWOX			
O95625	Zinc finger and BTB domain-containing protein 11	ZBTB11			
Q9NQX6	Zinc finger protein 331	ZNF331			
Q5FWF4	DNA annealing helicase and endonuclease ZRANB3	ZRANB3			
P0CG23	Zinc finger protein 853	ZNF853			
Q8N446	Zinc finger protein 843	ZNF843			
P0CH99	Zinc finger protein 705D	ZNF705D			
P17029	Zinc finger protein with KRAB and SCAN domains 1	ZKSCAN1			
Q9NR11	Zinc finger protein 302	ZNF302			
Q9HCZ1	Zinc finger protein 334	ZNF334			
Q9Y493	Zonadhesin	ZAN			
Q8NC26	Zinc finger protein 114	ZNF114			
Q68EA5	Zinc finger protein 57	ZNF57			
Q6NSZ9	Zinc finger and SCAN domain-containing protein 25	ZSCAN25			
Q9H7R0	Zinc finger protein 442	ZNF442			
Q86VM9	Zinc finger CCCH domain-containing protein 18	ZC3H18			
Q96SZ4	Zinc finger and SCAN domain-containing protein 10	ZSCAN10			

Q9Y5A6	Zinc finger and SCAN domain-containing protein 21	ZSCAN21			
O15090	Zinc finger protein 536	ZNF536			
P51815	Zinc finger protein 75D	ZNF75D			
Q8NEP9	Zinc finger protein 555	ZNF555			
Q96SQ5	Zinc finger protein 587	ZNF587			
O43345	Zinc finger protein 208	ZNF208			
Q96K75	Zinc finger protein 514	ZNF514			
Q8WW24	Tektin-4	TEKT4			
Q92734	Protein TFG	TFG			
Q9C0I4	Thrombospondin type-1 domain-containing protein 7B	THSD7B			
Q96MV1	TLC domain-containing protein 4	TLCD4			
Q9H0C3	Transmembrane protein 117	TMEM117			
Q96LM6	Testis-expressed sequence 37 protein	TEX37			
Q86T26	Transmembrane protease serine 11B	TMPRSS11B			
P04216	Thy-1 membrane glycoprotein	THY1			
Q9BVK8	Transmembrane protein 147	TMEM147			
A0A075B6Y3	T cell receptor alpha joining 3	TRAJ3			
Q92759	General transcription factor IIH subunit 4	GTF2H4			
A0A0A0MTA4	T cell receptor beta joining 2-5	TRBJ2-5			
Q8N1Q8	Acyl-coenzyme A thioesterase THEM5	THEM5			
Q5VZI3	Transmembrane protein 268	TMEM268			
Q96HH4	Transmembrane protein 169	TMEM169			
Q9GZN2	Homeobox protein TGIF2	TGIF2			
Q15561	Transcriptional enhancer factor TEF-3	TEAD4			
Q9NP99	Triggering receptor expressed on myeloid cells 1	TREM1			
Q8WZ59	Transmembrane protein 190	TMEM190			
P17752	Tryptophan 5-hydroxylase 1	TPH1			
Q9H3S3	Transmembrane protease serine 5	TMPRSS5			
P40225	Thrombopoietin	THPO			
P01374	Lymphotoxin-alpha	LTA			
Q13470	Non-receptor tyrosine-protein kinase TNK1	TNK1			
Q9Y4K3	TNF receptor-associated factor 6	TRAF6			
P40222	Alpha-taxilin	TXLNA			
Q8IYR6	Tomoregulin-1	TMEFF1			
Q15628	Tumor necrosis factor receptor type 1-associated DEATH domain protein	TRADD			
Q9P2K2	Thioredoxin domain-containing protein 16	TXNDC16			
Q5TCY1	Tau-tubulin kinase 1	TTBK1			
P0DPF4	T cell receptor alpha variable 35	TRAV35			
A0A0B4J26	T cell receptor alpha variable 4	TRAV4			
O75436	Vacuolar protein sorting-associated protein 26A	VPS26A			
Q8IWF7	Putative ubiquitin-conjugating enzyme E2 D2-like protein	UBE2DNL			
Q9UI12	V-type proton ATPase subunit H	ATP6V1H			
Q5GH70	XK-related protein 9	XKR9			
Q9NYS7	WD repeat and SOCS box-containing protein 2	WSB2			
A8MWX3	Putative WAS protein family homolog 4	WASH4P			
Q9H6Y2	WD repeat-containing protein 55	WDR55			
P78423	Fractalkine	CX3CL1			
Q9GZV5	WW domain-containing transcription regulator protein 1	WWTR1			
Q9UHU1	Putative uncharacterized protein PRO1716	PRO1716			

Q86TA4	Putative uncharacterized protein FLJ44553				
P49750	YLP motif-containing protein 1	YLPM1			
Q9H4I2	Zinc fingers and homeoboxes protein 3	ZHX3			
Q6ZSA8	Putative uncharacterized protein FLJ45684				
Q86XD8	AN1-type zinc finger protein 4	ZFAND4			
Q8NB50	Zinc finger protein 62 homolog	ZFP62			
Q05516	Zinc finger and BTB domain-containing protein 16	ZBTB16			
Q9HBT8	Zinc finger protein 286A	ZNF286A			
Q06732	Zinc finger protein 33B	ZNF33B			
Q6ZNA1	Zinc finger protein 836	ZNF836			
Q6PDB4	Zinc finger protein 880	ZNF880			
Q9BSG1	Zinc finger protein 2	ZNF2			
Q8NF64	Zinc finger MIZ domain-containing protein 2	ZMIZ2			
Q3MJ62	Zinc finger and SCAN domain-containing protein 23	ZSCAN23			
Q49A33	Putative zinc finger protein 876	ZNF876P			
Q52M93	Zinc finger protein 585B	ZNF585B			
Q96H79	Zinc finger CCCH-type antiviral protein 1-like	ZC3HAV1L			
P17097	Zinc finger protein 7	ZNF7			
Q6X784	Zona pellucida-binding protein 2	ZPBP2			
Q6ZN08	Putative zinc finger protein 66	ZNF66			
Q9BUY5	Zinc finger protein 426	ZNF426			
O15535	Zinc finger and SCAN domain-containing protein 9	ZSCAN9			
Q6XR72	Zinc transporter 10	SLC30A10			
Q6ECI4	Zinc finger protein 470	ZNF470			
B1APH4	Putative zinc finger protein 487	ZNF487			
Q9H091	Zinc finger MYND domain-containing protein 15	ZMYND15			
Q6IQ21	Zinc finger protein 770	ZNF770			
Q8WTR7	Zinc finger protein 473	ZNF473			
Q5MCW4	Zinc finger protein 569	ZNF569			
Q96LX8	Zinc finger protein 597	ZNF597			
Q92618	Zinc finger protein 516	ZNF516			
Q8NH93	Olfactory receptor 1L3	OR1L3			
Q8N148	Olfactory receptor 6V1	OR6V1			
Q53LP3	Ankyrin repeat domain-containing protein SOWAHC	SOWAHC			
Q8N434	Putative transporter SVOPL	SVOPL			
A0A0B4J23	T cell receptor alpha variable 13-2	TRAV13-2			
Q15526	Surfeit locus protein 1	SURF1			
Q9UH36	SRR1-like protein	SRRD			
Q06520	Sulfotransferase 2A1	SULT2A1			
P11831	Serum response factor	SRF			
Q14162	Scavenger receptor class F member 1	SCARF1			
Q5VSL9	Striatin-interacting protein 1	STRIP1			
Q9UJW9	SERTA domain-containing protein 3	SERTAD3			
Q13885	Tubulin beta-2A chain	TUBB2A			
Q9H6P5	Threonine aspartase 1	TASP1			
Q13509	Tubulin beta-3 chain	TUBB3			
Q9Y3F1	Putative TAP2-associated 6.5 kDa polypeptide				
Q99832	T-complex protein 1 subunit eta	CCT7			
Q4KMP7	TBC1 domain family member 10B	TBC1D10B			
P59535	Taste receptor type 2 member 40	TAS2R40			
A6NCK2	Tripartite motif-containing protein 43B	TRIM43B			
Q6UXY8	Transmembrane channel-like protein 5	TMC5			
Q5JXX7	Transmembrane protein 31	TMEM31			
Q9NVH6	Trimethyllysine dioxygenase, mitochondrial	TMLHE			

Q15629	Translocating chain-associated membrane protein 1	TRAM1			
O14669	Transmembrane gamma-carboxyglutamic acid protein 2	PRRG2			
Q96BF3	Transmembrane and immunoglobulin domain-containing protein 2	TMIGD2			
P19971	Thymidine phosphorylase	TYMP			
Q6P3X3	Tetratricopeptide repeat protein 27	TTC27			
A0A075B6T8	T cell receptor alpha variable 9-1	TRAV9-1			
P07101	Tyrosine 3-monooxygenase	TH			
Q96A04	TSSK6-activating co-chaperone protein	TSACC			
Q8IYN6	UBA-like domain-containing protein 2	UBALD2			
Q04323	UBX domain-containing protein 1	UBXN1			
Q9NV66	S-adenosyl-L-methionine-dependent tRNA 4-demethylwyosine synthase TYW1	TYW1			
O95399	Urotensin-2	UTS2			
O95498	Vascular non-inflammatory molecule 2	VNN2			
Q2YD98	UV-stimulated scaffold protein A	UVSSA			
P15692	Vascular endothelial growth factor A	VEGFA			
Q16572	Vesicular acetylcholine transporter	SLC18A3			
P98170	E3 ubiquitin-protein ligase XIAP	XIAP			
Q08AM6	Protein VAC14 homolog	VAC14			
Q96AX1	Vacuolar protein sorting-associated protein 33A	VPS33A			
P0C879	Putative uncharacterized protein FLJ43185				
Q8N814	Putative uncharacterized protein FLJ40140				
Q96EF9	Zinc fingers and homeoboxes protein 1, isoform 2	ZHX1-C8orf76			
Q9UKY1	Zinc fingers and homeoboxes protein 1	ZHX1			
Q6ZRN7	Putative uncharacterized protein FLJ46214				
P61129	Zinc finger CCCH domain-containing protein 6	ZC3H6			
Q6PJT7	Zinc finger CCCH domain-containing protein 14	ZC3H14			
P0C7X5	Zinc finger protein 806	ZNF806			
A6NEH8	Putative uncharacterized protein encoded by ZNF503-AS2	ZNF503-AS2			
Q96MX3	Zinc finger protein 48	ZNF48			
P10073	Zinc finger and SCAN domain-containing protein 22	ZSCAN22			
P51523	Zinc finger protein 84	ZNF84			
P13682	Zinc finger protein 35	ZNF35			
Q03923	Zinc finger protein 85	ZNF85			
Q8IYN0	Zinc finger protein 100	ZNF100			
Q7Z3I7	Zinc finger protein 572	ZNF572			
Q9ULJ6	Zinc finger MIZ domain-containing protein 1	ZMIZ1			
P98182	Zinc finger protein 200	ZNF200			
Q8TCW7	Zona pellucida-like domain-containing protein 1	ZPLD1			
Q8TAU3	Zinc finger protein 417	ZNF417			
Q9NYT6	Zinc finger protein 226	ZNF226			
P51508	Zinc finger protein 81	ZNF81			
Q9UJ78	Zinc finger MYM-type protein 5	ZMYM5			
Q8IYX1	TBC1 domain family member 21	TBC1D21			
Q8NBD8	Transmembrane protein 229B	TMEM229B			

Q15543	Transcription initiation factor TFIID subunit 13	TAF13			
Q9BVW5	TIMELESS-interacting protein	TIPIN			
Q6ZNK6	TRAF-interacting protein with FHA domain-containing protein B	TIFAB			
Q96A98	Tuberoinfundibular peptide of 39 residues	PTH2			
A0A0C4DH28	T cell receptor gamma variable 4	TRGV4			
O94842	TOX high mobility group box family member 4	TOX4			
Q8TDI7	Transmembrane channel-like protein 2	TMC2			
P06753	Tropomyosin alpha-3 chain	TPM3			
Q96Q05	Trafficking protein particle complex subunit 9	TRAPPC9			
Q9BW30	Tubulin polymerization-promoting protein family member [illegible]	TPPP3			
Q8WVT3	Trafficking protein particle complex subunit 12	TRAPPC12			
Q8NFQ8	Torsin-1A-interacting protein 2	TOR1AIP2			
Q9NP84	Tumor necrosis factor receptor superfamily member 12A	TNFRSF12A			
Q5SRN2	Testis-expressed basic protein 1	TSBP1			
Q8NA56	Tetratricopeptide repeat protein 29	TTC29			
Q9NQA5	Transient receptor potential cation channel subfamily V member 5	TRPV5			
Q96PF2	Testis-specific serine/threonine-protein kinase 2	TSSK2			
O00526	Uroplakin-2	UPK2			
P51784	Ubiquitin carboxyl-terminal hydrolase 11	USP11			
P55089	Urocortin	UCN			
Q9P2U7	Vesicular glutamate transporter 1	SLC17A7			
P18206	Vinculin	VCL			
P11473	Vitamin D3 receptor	VDR			
O43379	WD repeat-containing protein 62	WDR62			
Q8IZH2	5'-3' exoribonuclease 1	XRN1			
Q5JSH3	WD repeat-containing protein 44	WDR44			
Q71RG6	Putative chemokine-related protein FP248	FP248			
Q6ZSV7	Putative uncharacterized protein FLJ45177				
A6XGL0	YjeF N-terminal domain-containing protein 3	YJEFN3			
Q6UXP9	Putative uncharacterized protein UNQ9370/PRO34162	UNQ9370/PRO34162			
Q6ZR03	Uncharacterized protein FLJ46757				
Q6ZTK2	Putative uncharacterized protein LOC400499				
Q9BYJ9	YTH domain-containing family protein 1	YTHDF1			
Q49AA0	Zinc finger protein 69 homolog	ZFP69			
Q68DK2	Zinc finger FYVE domain-containing protein 26	ZFYVE26			
Q8NCN2	Zinc finger and BTB domain-containing protein 34	ZBTB34			
Q6U7Q0	Zinc finger protein 322	ZNF322			
Q96JL9	Zinc finger protein 333	ZNF333			
Q9BRR0	Zinc finger protein with KRAB and SCAN domains 3	ZKSCAN3			
Q8WUU4	Zinc finger protein 296	ZNF296			
Q9NQZ8	Endothelial zinc finger protein induced by tumor necrosis factor alpha	ZNF71			
Q9UDW3	Zinc finger matrin-type protein 5	ZMAT5			
Q86W11	Zinc finger and SCAN domain-containing protein 30	ZSCAN30			

P17039	Zinc finger protein 30	ZNF30			
Q9HA38	Zinc finger matrin-type protein 3	ZMAT3			
Q8NCK3	Zinc finger protein 485	ZNF485			
Q86UK7	E3 ubiquitin-protein ligase	ZNF598			
P17098	Zinc finger protein 8	ZNF8			
Q96CK0	Zinc finger protein 653	ZNF653			
Q96CS4	Zinc finger protein 689	ZNF689			
Q9BX82	Zinc finger protein 471	ZNF471			
Q8N720	Zinc finger protein 655	ZNF655			
Q8NGW1	Olfactory receptor 6B3	OR6B3			
Q5T2D3	OTU domain-containing protein 3	OTUD3			
O76099	Olfactory receptor 7C1	OR7C1			
Q9Y5P0	Olfactory receptor 51B4	OR51B4			
Q8NGA8	Olfactory receptor 4F17	OR4F17			
Q8NGJ6	Olfactory receptor 51A4	OR51A4			
Q8IYS5	Osteoclast-associated immunoglobulin-like receptor	OSCAR			
Q96QZ0	Pannexin-3	PANX3			
Q9NR21	Protein mono-ADP-ribosyltransferase PARP11	PARP11			
Q08493	cAMP-specific 3',5'-cyclic phosphodiesterase 4C	PDE4C			
Q8N5Y8	Protein mono-ADP-ribosyltransferase PARP16	PARP16			
P01298	Pancreatic prohormone	PPY			
Q8TAB3	Protocadherin-19	PCDH19			
Q9Y5E8	Protocadherin beta-15	PCDHB15			
O96013	Serine/threonine-protein kinase PAK 4	PAK4			
Q9Y5E9	Protocadherin beta-14	PCDHB14			
Q8TEQ8	GPI ethanolamine phosphate transferase 3	PIGO			
Q86YI8	PHD finger protein 13	PHF13			
Q96AQ6	Pre-B-cell leukemia transcription factor-interacting protein 1	PBXIP1			
O75925	E3 SUMO-protein ligase PIAS1	PIAS1			
P09086	POU domain, class 2, transcription factor 2	POU2F2			
Q8N8N7	Prostaglandin reductase 2	PTGR2			
Q9Y2R2	Tyrosine-protein phosphatase non-receptor type 22	PTPN22			
Q6MZT1	Regulator of G-protein signaling 7-binding protein	RGS7BP			
A6NEQ0	RNA-binding motif protein, Y chromosome, family 1 member E	RBMY1E			
P53805	Calcipressin-1	RCAN1			
Q8TAI7	GTPase RhebL1	RHEBL1			
Q96I51	RCC1-like G exchanging factor-like protein	RCC1L			
Q6VN20	Ran-binding protein 10	RANBP10			
Q96D15	Reticulocalbin-3	RCN3			
Q6ZP01	RNA-binding protein 44	RBM44			
Q8IUH3	RNA-binding protein 45	RBM45			
P49756	RNA-binding protein 25	RBM25			
Q6ZUM4	Rho GTPase-activating protein 27	ARHGAP27			
Q9Y4F9	Rho family-interacting cell polarization regulator 2	RIPOR2			
P25791	Rhombotin-2	LMO2			
Q8N5W9	Refilin-B	RFLNB			
P22670	MHC class II regulatory factor RFX1	RFX1			
A5PLK6	Regulator of G-protein signaling protein-like	RGSL1			
Q8IZJ4	Ral-GDS-related protein	RGL4			
P62750	60S ribosomal protein L23a	RPL23A			
Q5T653	39S ribosomal protein L2, mitochondrial	MRPL2			
Q7Z2W9	39S ribosomal protein L21, mitochondrial	MRPL21			
Q8WVD5	RING finger protein 141	RNF141			

P05388	60S acidic ribosomal protein P0	RPLP0			
Q9BT43	DNA-directed RNA polymerase III subunit RPC7-like	POLR3GL			
Q9Y252	E3 ubiquitin-protein ligase RNF6	RNF6			
Q9UNE2	Rab effector Noc2	RPH3AL			
P53803	DNA-directed RNA polymerases I, II, and III subunit RPABC4	POLR2K			
Q495C1	Probable E3 SUMO-protein ligase RNF212	RNF212			
Q7Z418	Potassium channel subfamily K member 18	KCNK18			
Q96EK5	KIF-binding protein	KIFBP			
Q6L8G9	Keratin-associated protein 5-6	KRTAP5-6			
Q9Y691	Calcium-activated potassium channel subunit beta-2	KCNMB2			
Q5T7N3	KN motif and ankyrin repeat domain-containing protein 4	KANK4			
Q9UQV4	Lysosome-associated membrane glycoprotein 3	LAMP3			
O75665	Oral-facial-digital syndrome 1 protein	OFD1			
P47887	Olfactory receptor 1E2	OR1E2			
O95190	Ornithine decarboxylase antizyme 2	OAZ2			
Q8IXE1	Olfactory receptor 4N5	OR4N5			
A6NF01	Putative nuclear envelope pore membrane protein POM 121B	POM121B			
Q9Y5H0	Protocadherin gamma-A3	PCDHGA3			
Q9HC56	Protocadherin-9	PCDH9			
Q9NPF4	Probable tRNA N6-adenosine threonylcarbamoyltransferase	OSGEP			
Q3MJ16	Cytosolic phospholipase A2 epsilon	PLA2G4E			
Q9NWS0	PIH1 domain-containing protein 1	PIH1D1			
Q53GG5	PDZ and LIM domain protein 3	PDLIM3			
Q6NUR6	Putative protein RNF216-like	RNF216P1			
A0A1B0GWK0	Parvalbumin-like EF-hand-containing protein	PVALEF			
P10826	Retinoic acid receptor beta	RARB			
Q5TGL8	PX domain-containing protein 1	PXDC1			
Q9UKL0	REST corepressor 1	RCOR1			
Q6NSI4	RPA-related protein RADX	RADX			
P55895	V	RAG2			
P24386	Rab proteins geranylgeranyltransferase component A 1	CHM			
P78509	Reelin	RELN			
Q9ULC3	Ras-related protein Rab-23	RAB23			
Q9Y4C8	Probable RNA-binding protein 19	RBM19			
Q15286	Ras-related protein Rab-35	RAB35			
Q8TBY0	Probable RNA-binding protein 46	RBM46			
Q96PK6	RNA-binding protein 14	RBM14			
Q5T5U3	Rho GTPase-activating protein 21	ARHGAP21			
Q86YS3	Rab11 family-interacting protein 4	RAB11FIP4			
Q08116	Regulator of G-protein signaling 1	RGS1			
P62745	Rho-related GTP-binding protein RhoB	RHOB			
Q02161	Blood group Rh	RHD			
Q7LG56	Ribonucleoside-diphosphate reductase subunit M2 B	RRM2B			
Q9H4X1	Regulator of cell cycle RGCC	RGCC			
Q684P5	Rap1 GTPase-activating protein 2	RAP1GAP2			
Q9NRR4	Ribonuclease 3	DROSHA			
Q05823	2-5A-dependent ribonuclease	RNASEL			
Q15050	Ribosome biogenesis regulatory protein homolog	RRS1			
Q6XPR3	Repetin	RPTN			
Q9H9B4	Sideroflexin-1	SFXN1			
P62341	Thioredoxin reductase-like selenoprotein T	SELENOT			

Q14242	P-selectin glycoprotein ligand 1	SELPLG			
Q7Z333	Probable helicase senataxin	SETX			
P20132	L-serine dehydratase/L-threonine deaminase	SDS			
Q14563	Semaphorin-3A	SEMA3A			
A0A286YF77	Small cysteine and glycine repeat-containing protein 6	SCYGR6			
Q96B97	SH3 domain-containing kinase-binding protein 1	SH3KBP1			
Q13435	Splicing factor 3B subunit 2	SF3B2			
Q6UXD5	Seizure 6-like protein 2	SEZ6L2			
O95470	Sphingosine-1-phosphate lyase 1	SGPL1			
O75920	Small EDRK-rich factor 1	SERF1A			
Q8WTV1	THAP domain-containing protein 3	THAP3			
P48061	Stromal cell-derived factor 1	CXCL12			
Q9UH03	Neuronal-specific septin-3	SEPTIN3			
Q9H190	Syntenin-2	SDCBP2			
Q96EE3	Nucleoporin SEH1	SEH1L			
Q9NS62	Thrombospondin type-1 domain-containing protein 1	THSD1			
B3SHH9	Transmembrane protein 114	TMEM114			
O60830	Mitochondrial import inner membrane translocase subunit Tim17-B	TIMM17B			
Q9BTF0	THUMP domain-containing protein 2	THUMPD2			
Q9BV44	THUMP domain-containing protein 3	THUMPD3			
Q92599	Septin-8	SEPTIN8			
Q9BY12	S phase cyclin A-associated protein in the endoplasmic reticulum	SCAPER			
Q12770	Sterol regulatory element-binding protein cleavage-activating protein	SCAP			
O75845	Lathosterol oxidase	SC5D			
P60059	Protein transport protein Sec61 subunit gamma	SEC61G			
Q92791	Endoplasmic reticulum protein SC65	P3H4			
Q9BXW3	Putative uncharacterized protein SNHG12	SNHG12			
P0DPA3	Putative uncharacterized protein SNHG28	SNHG28			
Q99611	Selenide, water dikinase 2	SEPHS2			
Q9NPE6	Sperm-associated antigen 4 protein	SPAG4			
Q96L92	Sorting nexin-27	SNX27			
Q8TCT6	Signal peptide peptidase-like 3	SPPL3			
Q92543	Sorting nexin-19	SNX19			
P53814	Smoothelin	SMTN			
Q9HAJ7	Histone deacetylase complex subunit SAP30L	SAP30L			
Q8IW52	SLIT and NTRK-like protein 4	SLITRK4			
Q6X4U4	Sclerostin domain-containing protein 1	SOSTDC1			
Q99619	SPRY domain-containing SOCS box protein 2	SPSB2			
Q9P1W8	Signal-regulatory protein gamma	SIRPG			
Q9NRC8	NAD-dependent protein deacetylase sirtuin-7	SIRT7			
A4D263	Spermatogenesis-associated protein 48	SPATA48			
Q8IUQ4	E3 ubiquitin-protein ligase SIAH1	SIAH1			
Q8NDV1	Alpha-N-acetylgalactosaminide alpha-2,6-sialyltransferase 3	ST6GALNAC3			
Q8N1F7	Nuclear pore complex protein Nup93	NUP93			

O14841	5-oxoprolinase	OPLAH			
P0C645	Olfactory receptor 4E1	OR4E1			
Q8NG84	Olfactory receptor 2AK2	OR2AK2			
Q9UBU9	Nuclear RNA export factor 1	NXF1			
Q8NGZ9	Olfactory receptor 2T10	OR2T10			
Q8NGH5	Olfactory receptor 56A1	OR56A1			
Q9H209	Olfactory receptor 10A4	OR10A4			
Q8NGC3	Olfactory receptor 10G2	OR10G2			
Q8NH63	Olfactory receptor 51H1	OR51H1			
Q8NH07	Olfactory receptor 11H2	OR11H2			
Q8NGF6	Olfactory receptor 10W1	OR10W1			
P51575	P2X purinoceptor 1	P2RX1			
Q9H607	Occludin/ELL domain-containing protein 1	OCEL1			
P0DN82	Olfactory receptor 12D1	OR12D1			
Q8NGS5	Olfactory receptor 13C4	OR13C4			
Q8N573	Oxidation resistance protein 1	OXR1			
A6NNC1	Putative POM121-like protein 1-like				
P49768	Presenilin-1	PSEN1			
Q96HP4	Oxidoreductase NAD-binding domain-containing protein 1	OXNAD1			
Q86SE9	Polycomb group RING finger protein 5	PCGF5			
Q9Y5G9	Protocadherin gamma-A4	PCDHGA4			
Q9GZY1	Prostate and breast cancer overexpressed gene 1 protein	PBOV1			
A0A0B4J2F0	Protein PIGBOS1	PIGBOS1			
Q92824	Proprotein convertase subtilisin/kexin type 5	PCSK5			
O75928	E3 SUMO-protein ligase PIAS2	PIAS2			
Q92576	PHD finger protein 3	PHF3			
Q7Z5L7	Podocan	PODN			
Q9UBV8	Peflin	PEF1			
O15172	Putative phosphoserine phosphatase-like protein	PSPHP1			
P60484	Phosphatidylinositol 3,4,5-trisphosphate 3-phosphatase and dual-specificity protein phosphatase PTEN	PTEN			
Q8WXF1	Paraspeckle component 1	PSPC1			
Q15678	Tyrosine-protein phosphatase non-receptor type 14	PTPN14			
Q9UNX3	60S ribosomal protein L26-like 1	RPL26L1			
Q01973	Inactive tyrosine-protein kinase transmembrane receptor ROR1	ROR1			
O95398	Rap guanine nucleotide exchange factor 3	RAPGEF3			
Q9H4P4	E3 ubiquitin-protein ligase NRDP1	RNF41			
Q8WZA2	Rap guanine nucleotide exchange factor 4	RAPGEF4			
Q92730	Rho-related GTP-binding protein Rho6	RND1			
Q63HN8	E3 ubiquitin-protein ligase	RNF213			
Q13296	Mammaglobin-A	SCGB2A2			
Q99720	Sigma non-opioid intracellular receptor 1	SIGMAR1			
A0A286YFB4	Small cysteine and glycine repeat-containing protein 2	SCYGR2			
Q5HYK7	SH3 domain-containing protein	SH3D19			
P21912	Succinate dehydrogenase	SDHB			
Q96EQ0	Small glutamine-rich tetratricopeptide repeat-containing protein beta	SGTB			
Q96GA7	Serine dehydratase-like	SDSL			
Q96NL6	Sodium channel and clathrin linker 1	SCLT1			
Q9UJC5	SH3 domain-binding glutamic acid-rich-like protein 2	SH3BGRL2			
Q6UW10	Surfactant-associated protein 2	SFTA2			

Q92529	SHC-transforming protein 3	SHC3			
Q6FHJ7	Secreted frizzled-related protein 4	SFRP4			
Q13530	Serine incorporator 3	SERINC3			
Q9NQW1	Protein transport protein Sec31B	SEC31B			
O75324	Stannin	SNN			
P55000	Secreted Ly-6/uPAR-related protein 1	SLURP1			
Q8IYB5	Stromal membrane-associated protein 1	SMAP1			
P0DP57	Secreted Ly-6/uPAR domain-containing protein 2	SLURP2			
O15079	Syntaphilin	SNPH			
Q9BVQ7	Spermatogenesis-associated protein 5-like protein 1	SPATA5L1			
Q9UQE7	Structural maintenance of chromosomes protein 3	SMC3			
Q9BXN6	Sperm protein associated with the nucleus on the X chromosome D	SPANXD			
Q9HCB6	Spondin-1	SPON1			
Q8N5G0	Small integral membrane protein 20	SMIM20			
Q9H2V7	Protein spinster homolog 1	SPNS1			
Q86T20	Small integral membrane protein 29	SMIM29			
A0A1B0GVY4	Small integral membrane protein 31	SMIM31			
P18583	Protein SON	SON			
P12757	Ski-like protein	SKIL			
Q86UG4	Solute carrier organic anion transporter family member 6A1	SLCO6A1			
P0CI01	Speedy protein E6	SPDYE6			
Q9HAU4	E3 ubiquitin-protein ligase SMURF2	SMURF2			
P41225	Transcription factor SOX-3	SOX3			
Q11203	CMP-N-acetylneuraminate-beta-1,4-galactoside alpha-2,3-sialyltransferase	ST3GAL3			
P0C7P3	Protein SLFN14	SLFN14			
O15173	Membrane-associated progesterone receptor component 2	PGRMC2			
Q32P51	Heterogeneous nuclear ribonucleoprotein A1-like 2	HNRNPA1L2			
Q8TE99	2-phosphoxylose phosphatase 1	PXYLP1			
O96011	Peroxisomal membrane protein 11B	PEX11B			
Q92626	Peroxidasin homolog	PXDN			
Q9Y2K5	R3H domain-containing protein 2	R3HDM2			
Q8N0T1	Ribosomal biogenesis factor	RBIS			
Q15283	Ras GTPase-activating protein 2	RASA2			
A6NJZ7	RIMS-binding protein 3C	RIMBP3C			
Q9H6L5	Reticulophagy regulator 1	RETREG1			
Q8IXN7	N-acetylaspartylglutamate synthase A	RIMKLA			
O15034	RIMS-binding protein 2	RIMBP2			
Q02543	60S ribosomal protein L18a	RPL18A			
P40429	60S ribosomal protein L13a	RPL13A			
P18124	60S ribosomal protein L7	RPL7			
Q9NX20	39S ribosomal protein L16, mitochondrial	MRPL16			
Q7Z7F7	39S ribosomal protein L55, mitochondrial	MRPL55			
Q8N5N7	39S ribosomal protein L50, mitochondrial	MRPL50			
Q9NVU0	DNA-directed RNA polymerase III subunit RPC5	POLR3E			
P56715	Oxygen-regulated protein 1	RP1			
Q9ULK6	RING finger protein 150	RNF150			

Q9NSQ0	Putative ribosomal RNA-processing protein 7 homolog B	RRP7BP			
Q92766	Ras-responsive element-binding protein 1	RREB1			
P61247	40S ribosomal protein S3a	RPS3A			
Q9H0K4	Radial spoke head protein 6 homolog A	RSPH6A			
A1A5C7	Solute carrier family 22 member 23	SLC22A23			
P82663	28S ribosomal protein S25, mitochondrial	MRPS25			
Q9UGH3	Solute carrier family 23 member 2	SLC23A2			
Q96RN1	Testis anion transporter 1	SLC26A8			
Q8WWV3	Reticulon-4-interacting protein 1, mitochondrial	RTN4IP1			
Q7LBE3	Solute carrier family 26 member 9	SLC26A9			
P06702	Protein S100-A9	S100A9			
Q9UBT2	SUMO-activating enzyme subunit 2	UBA2			
P80511	Protein S100-A12	S100A12			
Q9HCY8	Protein S100-A14	S100A14			
Q9BXP2	Solute carrier family 12 member 9	SLC12A9			
Q86VW1	Solute carrier family 22 member 16	SLC22A16			
Q8NF91	Nesprin-1	SYNE1			
Q9UHW9	Solute carrier family 12 member 6	SLC12A6			
Q2Y0W8	Electroneutral sodium bicarbonate exchanger 1	SLC4A8			
Q9UDX3	SEC14-like protein 4	SEC14L4			
B5MCN3	Putative SEC14-like protein 6	SEC14L6			
Q9BYT1	Solute carrier family 17 member 9	SLC17A9			
P82979	SAP domain-containing ribonucleoprotein	SARNP			
P21453	Sphingosine 1-phosphate receptor 1	S1PR1			
P0DJI8	Serum amyloid A-1 protein	SAA1			
Q6UVJ0	Spindle assembly abnormal protein 6 homolog	SASS6			
Q9Y2R9	28S ribosomal protein S7, mitochondrial	MRPS7			
Q86VV8	Rotatin	RTTN			
Q9NQC3	Reticulon-4	RTN4			
P09661	U2 small nuclear ribonucleoprotein A'	SNRPA1			
Q66K80	Putative uncharacterized protein RUSC1-AS1	RUSC1-AS1			
Q5K651	Sterile alpha motif domain-containing protein 9	SAMD9			
O60783	28S ribosomal protein S14, mitochondrial	MRPS14			
Q8TEE9	Histone deacetylase complex subunit SAP25	SAP25			
P82912	28S ribosomal protein S11, mitochondrial	MRPS11			
Q13214	Semaphorin-3B	SEMA3B			
Q9NR46	Endophilin-B2	SH3GLB2			
Q9UBV2	Protein sel-1 homolog 1	SEL1L			
Q9BT88	Synaptotagmin-11	SYT11			
Q96HL8	SH3 domain-containing YSC84-like protein 1	SH3YL1			
Q8N5H7	SH2 domain-containing protein 3C	SH2D3C			
Q8WV19	Vesicle transport protein SFT2A	SFT2D1			
Q12981	Vesicle transport protein SEC20	BNIP1			
Q15464	SH2 domain-containing adapter protein B	SHB			
O60939	Sodium channel subunit beta-2	SCN2B			
P0C7M3	Surfactant-associated protein 3	SFTA3			

Q8N6R1	Stress-associated endoplasmic reticulum protein 2	SERP2			
Q99590	Protein SCAF11	SCAF11			
Q96LD8	Sentrin-specific protease 8	SENP8			
Q12765	Secernin-1	SCRN1			
Q9Y345	Sodium- and chloride-dependent glycine transporter 2	SLC6A5			
Q8IYM1	Septin-12	SEPTIN12			
Q15796	Mothers against decapentaplegic homolog 2	SMAD2			
Q16613	Serotonin N-acetyltransferase	AANAT			
A6NHR9	Structural maintenance of chromosomes flexible hinge domain-containing protein 1	SMCHD1			
O75602	Sperm-associated antigen 6	SPAG6			
Q92922	SWI/SNF complex subunit SMARCC1	SMARCC1			
Q8TCT7	Signal peptide peptidase-like 2B	SPPL2B			
A6NCI5	Putative transmembrane protein encoded by LINC00862	LINC00862			
Q6PJ21	SPRY domain-containing SOCS box protein 3	SPSB3			
P56693	Transcription factor SOX-10	SOX10			
Q13526	Peptidyl-prolyl cis-trans isomerase NIMA-interacting 1	PIN1			
Q13371	Phosducin-like protein	PDCL			
P29590	Protein PML	PML			
Q14761	Protein tyrosine phosphatase receptor type C-associated protein	PTPRCAP			
P23468	Receptor-type tyrosine-protein phosphatase delta	PTPRD			
Q9H0J4	Glutamine-rich protein 2	QRICH2			
Q9BRQ0	Pygopus homolog 2	PYGO2			
Q8TDU9	Relaxin-3 receptor 2	RXFP4			
P62910	60S ribosomal protein L32	RPL32			
P0DPB6	DNA-directed RNA polymerases I and III subunit RPAC2	POLR1D			
Q9GZM3	DNA-directed RNA polymerase II subunit RPB11-b1	POLR2J2			
P52198	Rho-related GTP-binding protein RhoN	RND2			
Q9NVW2	E3 ubiquitin-protein ligase RLIM	RLIM			
Q5TA31	E3 ubiquitin-protein ligase	RNF187			
Q9BY78	E3 ubiquitin-protein ligase RNF26	RNF26			
Q96P16	Regulation of nuclear pre-mRNA domain-containing protein 1A	RPRD1A			
Q9NQ39	Putative 40S ribosomal protein S10-like	RPS10P5			
Q9Y2B1	Ribitol-5-phosphate xylosyltransferase 1	RXYLT1			
Q7Z769	Solute carrier family 35 member E3	SLC35E3			
Q9H2B4	Sulfate anion transporter 1	SLC26A1			
Q8WUT9	Solute carrier family 25 member 43	SLC25A43			
Q96E40	Sperm acrosome-associated protein 9	SPACA9			
Q6P1M0	Long-chain fatty acid transport protein 4	SLC27A4			
Q9Y2W3	Proton-associated sugar transporter A	SLC45A1			
Q8TF17	SH3 domain and tetratricopeptide repeat-containing protein 2	SH3TC2			
Q8NBI5	Solute carrier family 43 member 3	SLC43A3			
Q9UHP6	Radial spoke head 14 homolog	RSPH14			
Q8WWT9	Solute carrier family 13 member 3	SLC13A3			
Q9NRA2	Sialin	SLC17A5			

Q96DX4	RING finger and SPRY domain-containing protein 1	RSPRY1			
Q9UL12	Sarcosine dehydrogenase, mitochondrial	SARDH			
Q8WUM9	Sodium-dependent phosphate transporter 1	SLC20A1			
Q96BU1	S100P-binding protein	S100PBP			
Q6ZNE9	RUN and FYVE domain-containing protein 4	RUFY4			
O94885	SAM and SH3 domain-containing protein 1	SASH1			
P08621	U1 small nuclear ribonucleoprotein 70 kDa	SNRNP70			
Q7M4L6	SH2 domain-containing adapter protein F	SHF			
Q12874	Splicing factor 3A subunit 3	SF3A3			
Q99442	Translocation protein SEC62	SEC62			
Q99963	Endophilin-A3	SH3GL3			
Q9UN30	Sex comb on midleg-like protein	SCML1			
Q9UQR0	Sex comb on midleg-like protein	SCML2			
Q9BYB0	SH3 and multiple ankyrin repeat domains protein 3	SHANK3			
Q86VW0	SEC14 domain and spectrin repeat-containing protein 1	SESTD1			
Q15019	Septin-2	SEPTIN2			
O00560	Syntenin-1	SDCBP			
Q96F10	Thialysine N-epsilon-acetyltransferase	SAT2			
Q9BSV6	tRNA-splicing endonuclease subunit Sen34	TSEN34			
Q8N9R8	Protein SCAI	SCAI			
Q9NY91	Solute carrier family 5 member 4	SLC5A4			
P47872	Secretin receptor	SCTR			
P21583	Kit ligand	KITLG			
Q6GMV2	SET and MYND domain-containing protein 5	SMYD5			
Q9NXX6	Non-structural maintenance of chromosomes element 4 homolog A	NSMCE4A			
Q8NGZ5	Olfactory receptor 2G2	OR2G2			
Q8TE49	OTU domain-containing protein 7A	OTUD7A			
Q8NDX1	PH and SEC7 domain-containing protein 4	PSD4			
Q9Y5G6	Protocadherin gamma-A7	PCDHGA7			
A0A2R8YFL7	Oocyte-secreted protein 4A	OOSP4A			
Q8NH16	Olfactory receptor 2L2	OR2L2			
Q6QHF9	Peroxisomal N	PAOX			
Q5QFB9	Protein PAPPAS	PAPPA-AS1			
O43252	Bifunctional 3'-phosphoadenosine 5'-phosphosulfate synthase 1	PAPSS1			
Q8NGA6	Olfactory receptor 10H5	OR10H5			
Q9BZA7	Protocadherin-11 X-linked	PCDH11X			
Q7L5N7	Lysophosphatidylcholine acyltransferase 2	LPCAT2			
P16499	Rod cGMP-specific 3',5'-cyclic phosphodiesterase subunit alpha	PDE6A			
P0CB38	Polyadenylate-binding protein 4-like	PABPC4L			
Q15365	Poly	PCBP1			
P27815	cAMP-specific 3',5'-cyclic phosphodiesterase 4A	PDE4A			
Q86U42	Polyadenylate-binding protein 2	PABPN1			
Q9BPV8	P2Y purinoceptor 13	P2RY13			
Q13177	Serine/threonine-protein kinase PAK 2	PAK2			
Q9P0J1		PDP1			
Q32P28	Prolyl 3-hydroxylase 1	P3H1			

P30039	Phenazine biosynthesis-like domain-containing protein	PBLD			
Q92508	Piezo-type mechanosensitive ion channel component 1	PIEZO1			
Q53EL6	Programmed cell death protein 4	PDCD4			
Q9H0N5	Pterin-4-alpha-carbinolamine dehydratase 2	PCBD2			
Q8WXI9	Transcriptional repressor p66-	GATAD2B			
Q8N490	Probable hydrolase PNKD	PNKD			
Q6P1K2	Polyamine-modulated factor 1	PMF1			
A0A1W2PS18	Transmembrane protein PMIS2	PMIS2			
P10266	Endogenous retrovirus group K member 10 Pol protein	ERVK-10			
Q9ULR3	Protein phosphatase 1H	PPM1H			
Q9Y446	Plakophilin-3	PKP3			
A0JP26	POTE ankyrin domain family member B3	POTEB3			
Q99471	Prefoldin subunit 5	PFDN5			
P35813	Protein phosphatase 1A	PPM1A			
P42338	Phosphatidylinositol 4,5-bisphosphate 3-kinase catalytic subunit beta isoform	PIK3CB			
A0PG75	Phospholipid scramblase family member 5	PLSCR5			
Q9UHV8	Galactoside-binding soluble lectin 13	LGALS13			
Q63HM9	PI-PLC X domain-containing protein 3	PLCXD3			
P25787	Proteasome subunit alpha type-2	PSMA2			
Q7Z3G6	Prickle-like protein 2	PRICKLE2			
P04554	Protamine-2	PRM2			
Q00887	Pregnancy-specific beta-1-glycoprotein 9	PSG9			
P26599	Polypyrimidine tract-binding protein 1	PTBP1			
Q9NXS2	Glutaminyl-peptide cyclotransferase-like protein	QPCTL			
B4DYI2	Putative spermatogenesis-associated protein 31C2	SPATA31C2			
Q9H446	RWD domain-containing protein	RWDD1			
Q9UIY3	RWD domain-containing protein 2A	RWDD2A			
P0C7Q6	Solute carrier family 35 member G6	SLC35G6			
Q8N413	Solute carrier family 25 member 45	SLC25A45			
Q8N357	Solute carrier family 35 member	SLC35F6			
Q96ER3	Protein SAAL1	SAAL1			
P21817	Ryanodine receptor 1	RYR1			
Q9ULF5	Zinc transporter ZIP10	SLC39A10			
Q96JW4	Solute carrier family 41 member 2	SLC41A2			
Q15424	Scaffold attachment factor B1	SAFB			
P47897	Glutamine--tRNA ligase	QARS1			
Q9UJJ7	RNA pseudouridylate synthase domain-containing protein 1	RPUSD1			
Q96DX8	Receptor-transporting protein 4	RTP4			
Q6P4A7	Sideroflexin-4	SFXN4			
Q08648	Sperm-associated antigen 11B	SPAG11B			
Q9BQE4	Selenoprotein S	SELENOS			
Q8IYI0	Shieldin complex subunit 1	SHLD1			
O95969	Secretoglobin family 1D member	SCGB1D2			
Q15428	Splicing factor 3A subunit 2	SF3A2			
P31040	Succinate dehydrogenase	SDHA			
Q99962	Endophilin-A1	SH3GL2			
Q9Y3B4	Splicing factor 3B subunit 6	SF3B6			
Q8TEJ3	E3 ubiquitin-protein ligase	SH3RF3			
P29353	SHC-transforming protein 1	SHC1			

Q9NY46	Sodium channel protein type 3 subunit alpha	SCN3A			
Q9NY72	Sodium channel subunit beta-3	SCN3B			
Q9NQF3	Serine hydrolase-like protein	SERHL			
A8MV23	Serpin E3	SERPINE3			
Q6ICB4	Sesquipedalian-2	PHETA2			
Q9H2E6	Semaphorin-6A	SEMA6A			
Q02383	Semenogelin-2	SEMG2			
Q7Z6J9	tRNA-splicing endonuclease subunit Sen54	TSEN54			
Q9GZR1	Sentrin-specific protease 6	SENP6			
P0C264	Uncharacterized serine/threonine-protein kinase SBK3	SBK3			
Q6ZU15	Septin-14	SEPTIN14			
P67812	Signal peptidase complex catalytic subunit SEC11A	SEC11A			
O76013	Keratin, type I cuticular Ha6	KRT36			
P48050	Inward rectifier potassium channel 4	KCNJ4			
Q9HB14	Potassium channel subfamily K member 13	KCNK13			
Q9NZI2	Kv channel-interacting protein 1	KCNIP1			
Q04759	Protein kinase C theta type	PRKCQ			
P27361	Mitogen-activated protein kinase 3	MAPK3			
Q7Z6M3	Allergin-1	MILR1			
P14780	Matrix metalloproteinase-9	MMP9			
Q8NGT0	Olfactory receptor 13C9	OR13C9			
A6NES4	Maestro heat-like repeat-containing protein family member 2A	MROH2A			
O95255	Multidrug resistance-associated protein 6	ABCC6			
O95665	Neurotensin receptor type 2	NTSR2			
Q9Y4C0	Neurexin-3	NRXN3			
Q9Y328	Neuronal vesicle trafficking-associated protein 2	NSG2			
Q96P68	2-oxoglutarate receptor 1	OXGR1			
Q8WXA2	Prostate and testis expressed protein 1	PATE1			
P10082	Peptide YY	PYY			
Q9HBH0	Rho-related GTP-binding protein RhoF	RHOF			
Q9H426	Regulating synaptic membrane exocytosis protein 4	RIMS4			
Q5EBL4	RILP-like protein 1	RILPL1			
Q9NWS8	Required for meiotic nuclear division protein 1 homolog	RMND1			
Q9Y4L5	E3 ubiquitin-protein ligase	RNF115			
Q8N7X1	RNA-binding motif protein, X-linked-like-3	RBMXL3			
P62917	60S ribosomal protein L8	RPL8			
Q9Y225	RING finger protein 24	RNF24			
Q15637	Splicing factor 1	SF1			
A0A286YFG1	Small cysteine and glycine repeat-containing protein 8	SCYGR8			
P0DSO2	Small cysteine and glycine repeat-containing protein 9	SCYGR9			
Q15459	Splicing factor 3A subunit 1	SF3A1			
O95754	Semaphorin-4F	SEMA4F			
Q8TEC5	E3 ubiquitin-protein ligase	SH3RF2			
P04278	Sex hormone-binding globulin	SHBG			
Q6P3W7	SCY1-like protein 2	SCYL2			
Q8IWT1	Sodium channel subunit beta-4	SCN4B			
Q5T4F7	Secreted frizzled-related protein 5	SFRP5			
Q8N474	Secreted frizzled-related protein 1	SFRP1			
Q8WYJ6	Septin-1	SEPTIN1			
Q9H3T2	Semaphorin-6C	SEMA6C			
Q99719	Septin-5	SEPTIN5			
Q14141	Septin-6	SEPTIN6			

Q15437	Protein transport protein Sec23B	SEC23B			
P0C263	Serine/threonine-protein kinase SBK2	SBK2			
Q9BQF6	Sentrin-specific protease 7	SENP7			
O95487	Protein transport protein Sec24B	SEC24B			
P57772	Selenocysteine-specific elongation factor	EEFSEC			
Q8TAC9	Secretory carrier-associated membrane protein 5	SCAMP5			
Q658L1	Stabilizer of axonemal microtubules 2	SAXO2			
Q9Y5Y9	Sodium channel protein type 10 subunit alpha	SCN10A			
Q8N695	Sodium-coupled monocarboxylate transporter 1	SLC5A8			
P48067	Sodium- and chloride-dependent glycine transporter 1	SLC6A9			
Q01959	Sodium-dependent dopamine transporter	SLC6A3			
Q6R2W3	SCAN domain-containing protein 3	ZBED9			
P63208	S-phase kinase-associated protein 1	SKP1			
Q8IW75	Serpin A12	SERPINA12			
Q13309	S-phase kinase-associated protein 2	SKP2			
Q969G3	SWI/SNF-related matrix-associated actin-dependent regulator of chromatin subfamily E member 1	SMARCE1			
Q9Y5B9	FACT complex subunit SPT16	SUPT16H			
Q9H4F8	SPARC-related modular calcium-binding protein 1	SMOC1			
Q9H210	Olfactory receptor 2D2	OR2D2			
Q9UNZ2	NSFL1 cofactor p47	NSFL1C			
P51003	Poly	PAPOLA			
Q9H714	Protein associated with UVRAG as autophagy enhancer	RUBCNL			
Q5JUK9	P antigen family member 3	PAGE3			
O43924	Retinal rod rhodopsin-sensitive cGMP 3',5'-cyclic phosphodiesterase subunit delta	PDE6D			
Q9H074	Polyadenylate-binding protein-interacting protein 1	PAIP1			
O75914	Serine/threonine-protein kinase PAK 3	PAK3			
O00623	Peroxisome assembly protein 12	PEX12			
C9JE40	Protein PAT1 homolog 2	PATL2			
Q9NST1	1-acylglycerol-3-phosphate O-acyltransferase PNPLA3	PNPLA3			
P0CW24	Paraneoplastic antigen-like protein 6A	PNMA6A			
Q86XI6	Protein phosphatase 1 regulatory subunit 3B	PPP1R3B			
Q9BRP8	Partner of Y14 and mago	PYM1			
O76021	Ribosomal L1 domain-containing protein 1	RSL1D1			
P46777	60S ribosomal protein L5	RPL5			
O60930	Ribonuclease H1	RNASEH1			
A6NIZ1	Ras-related protein Rap-1b-like protein				
Q6ZSG1	E3 ubiquitin-protein ligase	RNF165			
Q15287	RNA-binding protein with serine-rich domain 1	RNPS1			
Q9Y6U7	RING finger protein 215	RNF215			
Q969S9	Ribosome-releasing factor 2, mitochondrial	GFM2			
P15880	40S ribosomal protein S2	RPS2			

Q5VU36	Spermatogenesis-associated protein 31A5	SPATA31A5			
Q8IZD6	Solute carrier family 22 member 15	SLC22A15			
Q9BZJ4	Solute carrier family 25 member 39	SLC25A39			
Q92665	28S ribosomal protein S31, mitochondrial	MRPS31			
Q9HBV2	Sperm acrosome membrane-associated protein 1	SPACA1			
Q6P5W5	Zinc transporter ZIP4	SLC39A4			
Q99808	Equilibrative nucleoside transporter 1	SLC29A1			
Q15043	Metal cation symporter ZIP14	SLC39A14			
P31949	Protein S100-A11	S100A11			
Q9UBE0	SUMO-activating enzyme subunit 1	SAE1			
O43765	Small glutamine-rich tetratricopeptide repeat-containing protein alpha	SGTA			
Q9C0C4	Semaphorin-4C	SEMA4C			
Q9HCN8	Stromal cell-derived factor 2-like protein 1	SDF2L1			
P55822	SH3 domain-binding glutamic acid-rich protein	SH3BGR			
A4FU49	SH3 domain-containing protein	SH3D21			
O75368	SH3 domain-binding glutamic acid-rich-like protein	SH3BGRL			
Q8IZQ5	Selenoprotein H	SELENOH			
Q7Z6J0	E3 ubiquitin-protein ligase	SH3RF1			
P55735	Protein SEC13 homolog	SEC13			
Q12872	Splicing factor, suppressor of white-apricot homolog	SFSWAP			
Q99250	Sodium channel protein type 2 subunit alpha	SCN2A			
Q9NP50	SIN3-HDAC complex-associated factor	SINHCAF			
P23246	Splicing factor, proline- and glutamine-rich	SFPQ			
Q9H7N4	Splicing factor, arginine/serine-rich 19	SCAF1			
Q9H1H9	Kinesin-like protein KIF13A	KIF13A			
O00142	Thymidine kinase 2, mitochondrial	TK2			
Q9HA64	Ketosamine-3-kinase	FN3KRP			
B2CW77	Killin	KLLN			
O60341	Lysine-specific histone demethylase 1A	KDM1A			
Q3MIV0	Keratin-associated protein 22-1	KRTAP22-1			
O60938	Keratocan	KERA			
Q86WI0	LHFPL tetraspan subfamily member 1 protein	LHFPL1			
P05771	Protein kinase C beta type	PRKCB			
C9JBD0	KRAB domain-containing protein 1	KRBOX1			
Q96MC5	bMERB domain-containing protein 1	BMERB1			
Q5EB52	Mesoderm-specific transcript homolog protein	MEST			
P03956	Interstitial collagenase	MMP1			
Q2M2H8	Probable maltase-glucoamylase 2	MGAM2			
O60487	Myelin protein zero-like protein 2	MPZL2			
Q96HJ5	Membrane-spanning 4-domains subfamily A member 3	MS4A3			
Q99572	P2X purinoceptor 7	P2RX7			
Q14872	Metal regulatory transcription factor 1	MTF1			
Q15390	Mitochondrial fission regulator 1	MTFR1			
O15146	Muscle, skeletal receptor tyrosine-protein kinase	MUSK			

P11055	Myosin-3	MYH3			
Q3BBV2	Putative neuroblastoma breakpoint family member 8	NBPF8			
Q9BXD5	N-acetylneuraminate lyase	NPL			
Q13823	Nucleolar GTP-binding protein 2	GNL2			
Q92979	Ribosomal RNA small subunit methyltransferase NEP1	EMG1			
Q9HBJ0	Placenta-specific protein 1	PLAC1			
Q86UY8	5'-nucleotidase domain-containing protein 3	NT5DC3			
Q5TFE4	5'-nucleotidase domain-containing protein 1	NT5DC1			
O60356	Nuclear protein 1	NUPR1			
Q8NH09	Olfactory receptor 8S1	OR8S1			
P58173	Olfactory receptor 2B6	OR2B6			
Q8NGT9	Olfactory receptor 2A1/2A42	OR2A1;			
Q969Y0	NXPE family member 3	NXPE3			
Q8NG76	Olfactory receptor 2T33	OR2T33			
Q9P242	Neuronal tyrosine-phosphorylated phosphoinositide-3-kinase adaptor 2	NYAP2			
O95013	Olfactory receptor 4F21	OR4F21			
Q13093	Platelet-activating factor acetylhydrolase	PLA2G7			
Q99487	Platelet-activating factor acetylhydrolase 2, cytoplasmic	PAFAH2			
Q8WX93	Palladin	PALLD			
Q9H999	Pantothenate kinase 3	PANK3			
P35913	Rod cGMP-specific 3',5'-cyclic phosphodiesterase subunit beta	PDE6B			
P55085	Proteinase-activated receptor 2	F2RL1			
P05165	Propionyl-CoA carboxylase alpha chain, mitochondrial	PCCA			
Q9H0J9	Protein mono-ADP-ribosyltransferase PARP12	PARP12			
P57721	Poly	PCBP3			
Q9UN73	Protocadherin alpha-6	PCDHA6			
Q9NZK7	Group IIE secretory phospholipase A2	PLA2G2E			
Q7Z2X4	PTB-containing, cubilin and LRP1-interacting protein	PID1			
O75167	Phosphatase and actin regulator 2	PHACTR2			
P78562	Phosphate-regulating neutral endopeptidase PHEX	PHEX			
Q9BUL5	PHD finger protein 23	PHF23			
Q16816	Phosphorylase b kinase gamma catalytic chain, skeletal muscle/heart isoform	PHKG1			
Q9NRX4	14 kDa phosphohistidine phosphatase	PHPT1			
P14222	Perforin-1	PRF1			
Q6UWI2	Prostate androgen-regulated mucin-like protein 1	PARM1			
P20265	POU domain, class 3, transcription factor 2	POU3F2			
P45877	Peptidyl-prolyl cis-trans isomerase C	PPIC			
Q8IV08	5'-3' exonuclease PLD3	PLD3			
P48147	Prolyl endopeptidase	PREP			
Q96LW4	DNA-directed primase/polymerase protein	PRIMPOL			
Q86XN7	Proline and serine-rich protein 1	PROSER1			
Q12913	Receptor-type tyrosine-protein phosphatase eta	PTPRJ			
P22102	Trifunctional purine biosynthetic protein adenosine-3	GART			
Q969U7	Proteasome assembly chaperone 2	PSMG2			
Q8IY67	Ribonucleoprotein PTB-binding 1	RAVER1			

Q5RL73	RNA-binding protein 48	RBM48			
Q9Y580	RNA-binding protein 7	RBM7			
P25800	Rhombotin-1	LMO1			
P10276	Retinoic acid receptor alpha	RARA			
P46063	ATP-dependent DNA helicase Q1	RECQL			
A6NED2	RCC1 domain-containing protein 1	RCCD1			
Q9H0U4	Ras-related protein Rab-1B	RAB1B			
Q96BD5	PHD finger protein 21A	PHF21A			
Q6ZS72	Protein PEAK3	PEAK3			
O60664	Perilipin-3	PLIN3			
Q15165	Serum paraoxonase/arylesterase 2	PON2			
P27169	Serum paraoxonase/arylesterase 1	PON1			
P07738	Bisphosphoglycerate mutase	BPGM			
Q6ZRT6	Proline-rich protein 23B	PRR23B			
P28827	Receptor-type tyrosine-protein phosphatase mu	PTPRM			
P54829	Tyrosine-protein phosphatase non-receptor type 5	PTPN5			
P43378	Tyrosine-protein phosphatase non-receptor type 9	PTPN9			
P11498	Pyruvate carboxylase, mitochondrial	PC			
O15315	DNA repair protein RAD51 homolog 2	RAD51B			
Q9HD33	39S ribosomal protein L47, mitochondrial	MRPL47			
Q96GC5	39S ribosomal protein L48, mitochondrial	MRPL48			
Q96C74	Ropporin-1-like protein	ROPN1L			
O95602	DNA-directed RNA polymerase I subunit RPA1	POLR1A			
Q8ND24	RING finger protein 214	RNF214			
Q96KT7	Solute carrier family 35 member G5	SLC35G5			
Q8N5S1	Mitochondrial carrier protein SCaMC-3L	SLC25A41			
P78383	Solute carrier family 35 member B1	SLC35B1			
A6NKX4	Putative solute carrier family 22 member 31	SLC22A31			
P23526	Adenosylhomocysteinase	AHCY			
Q969I6	Sodium-coupled neutral amino acid transporter 4	SLC38A4			
Q96QD8	Sodium-coupled neutral amino acid transporter 2	SLC38A2			
Q9BXS9	Solute carrier family 26 member 6	SLC26A6			
Q99624	Sodium-coupled neutral amino acid transporter 3	SLC38A3			
Q9HBR0	Putative sodium-coupled neutral amino acid transporter 10	SLC38A10			
Q9BSG5	Retbindin	RTBDN			
O00337	Sodium/nucleoside cotransporter 1	SLC28A1			
W5XKT8	Sperm acrosome membrane-associated protein 6	SPACA6			
P33763	Protein S100-A5	S100A5			
Q8N1S5	Zinc transporter ZIP11	SLC39A11			
Q6UXD7	Solute carrier family 49 member A3	SLC49A3			
Q9UP95	Solute carrier family 12 member 4	SLC12A4			
A6ZKI3	Retrotransposon Gag-like protein 8C	RTL8C			
Q9Y6M7	Sodium bicarbonate cotransporter 3	SLC4A7			
Q9Y6R1	Electrogenic sodium bicarbonate cotransporter 1	SLC4A4			
Q9Y285	Phenylalanine--tRNA ligase alpha subunit	FARSA			

Q9Y399	28S ribosomal protein S2, mitochondrial	MRPS2			
A6NIM6	Solute carrier family 15 member 5	SLC15A5			
Q8N2U9	Solute carrier family 66 member 2	SLC66A2			
Q2MKA7	R-spondin-1	RSPO1			
P46059	Solute carrier family 15 member 1	SLC15A1			
Q5SY68	Protein S100-A7-like 2	S100A7L2			
P61619	Protein transport protein Sec61 subunit alpha isoform 1	SEC61A1			
Q9H1X1	Radial spoke head protein 9 homolog	RSPH9			
P48065	Sodium- and chloride-dependent betaine transporter	SLC6A12			
Q8TBB6	Probable cationic amino acid transporter	SLC7A14			
Q695T7	Sodium-dependent neutral amino acid transporter B	SLC6A19			
P82675	28S ribosomal protein S5, mitochondrial	MRPS5			
Q9NP91	Sodium- and chloride-dependent transporter XTRP3	SLC6A20			
P63162	Small nuclear ribonucleoprotein-associated protein N	SNRPN			
P59025	Receptor-transporting protein 1	RTP1			
P82664	28S ribosomal protein S10, mitochondrial	MRPS10			
Q7Z5N4	Protein sidekick-1	SDK1			
P83859	Orexigenic neuropeptide QRFP	QRFP			
Q9NQN1	Olfactory receptor 2S2	OR2S2			
Q9H7Z3	Nuclear exosome regulator NRDE2	NRDE2			
Q9Y5F7	Protocadherin gamma-C4	PCDHGC4			
Q9Y5I4	Protocadherin alpha-C2	PCDHAC2			
Q8NGA5	Olfactory receptor 10H4	OR10H4			
O60658	High affinity cAMP-specific and IBMX-insensitive 3',5'-cyclic phosphodiesterase 8A	PDE8A			
Q9HCL0	Protocadherin-18	PCDH18			
P12004	Proliferating cell nuclear antigen	PCNA			
Q8WW12	PEST proteolytic signal-containing nuclear protein	PCNP			
B3GLJ2	Prostate and testis expressed protein 3	PATE3			
Q86WH2	Ras association domain-containing protein 3	RASSF3			
Q9NS23	Ras association domain-containing protein 1	RASSF1			
Q8TD07	Retinoic acid early transcript 1E	RAET1E			
P62826	GTP-binding nuclear protein Ran	RAN			
P48380	Transcription factor RFX3	RFX3			
Q9P2R6	Arginine-glutamic acid dipeptide repeats protein	RERE			
Q6ZV50	DNA-binding protein RFX8	RFX8			
Q96D71	RalBP1-associated Eps domain-containing protein 1	REPS1			
Q9BVS4	Serine/threonine-protein kinase RIO2	RIOK2			
P30050	60S ribosomal protein L12	RPL12			
Q03395	Rod outer segment membrane protein 1	ROM1			
Q96E14	RecQ-mediated genome instability protein 2	RMI2			
O75116	Rho-associated protein kinase 2	ROCK2			
O76064	E3 ubiquitin-protein ligase RNF8	RNF8			
Q5TBB1	Ribonuclease H2 subunit B	RNASEH2B			
P04844	Dolichyl-diphosphooligosaccharide--protein glycosyltransferase subunit 2	RPN2			

O43159	Ribosomal RNA-processing protein 8	RRP8			
P10301	Ras-related protein R-Ras	RRAS			
P62241	40S ribosomal protein S8	RPS8			
Q9BPW5	Ras-like protein family member 11B	RASL11B			
Q8WUG5	Solute carrier family 22 member 17	SLC22A17			
Q9NTN3	UDP-glucuronic acid/UDP-N-acetylgalactosamine transporter	SLC35D1			
Q495N2	Proton-coupled amino acid transporter 3	SLC36A3			
Q9NP92	39S ribosomal protein S30, mitochondrial	MRPS30			
Q8NG04	Solute carrier family 26 member 10	SLC26A10			
Q8IZ73	RNA pseudouridylate synthase domain-containing protein 2	RPUSD2			
Q5SSQ6	Suppressor APC domain-containing protein 1	SAPCD1			
P60896	26S proteasome complex subunit SEM1	SEM1			
Q9Y371	Endophilin-B1	SH3GLB1			
Q2NKQ1	Small G protein signaling modulator 1	SGSM1			
P17482	Homeobox protein Hox-B9	HOXB9			
A0A0B4J1Y9	Immunoglobulin heavy variable 3-72	IGHV3-72			
Q9NWY4	Histone PARylation factor 1	HPF1			
P0CJ69	Humanin-like 2	MTRNR2L2			
P40305	Interferon alpha-inducible protein 27, mitochondrial	IFI27			
P01579	Interferon gamma	IFNG			
Q9NPH3	Interleukin-1 receptor accessory protein	IL1RAP			
Q8N9C0	Immunoglobulin superfamily member 22	IGSF22			
Q8NFM7	Interleukin-17 receptor D	IL17RD			
Q9NSI5	Immunoglobulin superfamily member 5	IGSF5			
O95760	Interleukin-33	IL33			
Q96BM0	Interferon alpha-inducible protein 27-like protein 1	IFI27L1			
Q5T953	Immediate early response gene 5-like protein	IER5L			
P98153	Integral membrane protein DGCR2/IDD	DGCR2			
Q5TF58	Intermediate filament family orphan 2	IFFO2			
Q96RY7	Intraflagellar transport protein 140 homolog	IFT140			
P50221	Homeobox protein MOX-1	MEOX1			
P30301	Lens fiber major intrinsic protein	MIP			
P50222	Homeobox protein MOX-2	MEOX2			
Q16819	Meprin A subunit alpha	MEP1A			
Q9HAP2	MLX-interacting protein	MLXIP			
Q16820	Meprin A subunit beta	MEP1B			
Q9UH92	Max-like protein X	MLX			
Q8IXI2	Mitochondrial Rho GTPase 1	RHOT1			
Q8IXI1	Mitochondrial Rho GTPase 2	RHOT2			
Q9H081	Protein MIS12 homolog	MIS12			
Q9ULR0	Pre-mRNA-splicing factor ISY1 homolog	ISY1			
O75578	Integrin alpha-10	ITGA10			
Q96DU7	Inositol-trisphosphate 3-kinase C	ITPKC			
Q8IV33	Uncharacterized protein KIAA0825	KIAA0825			
Q5TA45	Integrator complex subunit 11	INTS11			
Q96L93	Kinesin-like protein KIF16B	KIF16B			

P43630	Killer cell immunoglobulin-like receptor 3DL2	KIR3DL2			
P0DP09	Immunoglobulin kappa variable 1-13	IGKV1-13			
P42166	Lamina-associated polypeptide 2, isoform alpha	TMPO			
P11279	Lysosome-associated membrane glycoprotein 1	LAMP1			
Q53QV2	Protein LBH	LBH			
Q9BUB5	MAP kinase-interacting serine/threonine-protein kinase 1	MKNK1			
O43934	UNC93-like protein MFSD11	MFSD11			
Q9Y6X9	ATPase MORC2	MORC2			
Q96DH6	RNA-binding protein Musashi homolog 2	MSI2			
P25713	Metallothionein-3	MT3			
P42898	Methylenetetrahydrofolate reductase	MTHFR			
Q8IVL0	Neuron navigator 3	NAV3			
Q96H55	Unconventional myosin-XIX	MYO19			
Q5VVJ2	Deubiquitinase MYSM1	MYSM1			
Q9ULV0	Unconventional myosin-Vb	MYO5B			
Q9Y623	Myosin-4	MYH4			
P02144	Myoglobin	MB			
P59044	NACHT, LRR and PYD domains-containing protein 6	NLRP6			
Q9NZ94	Neuroligin-3	NLGN3			
Q12986	Transcriptional repressor NF-X1	NFX1			
P28336	Neuromedin-B receptor	NMBR			
A6NFF2	Putative nucleosome assembly protein 1-like 6	NAP1L6P			
Q5HYW2	NHS-like protein 2	NHSL2			
Q96TA1	Protein Niban 2	NIBAN2			
Q14112	Nidogen-2	NID2			
Q13253	Noggin	NOG			
Q14934	Nuclear factor of activated T-cells, cytoplasmic 4	NFATC4			
Q9NX24	H/ACA ribonucleoprotein complex subunit 2	NHP2			
P56556	NADH dehydrogenase	NDUFA6			
Q9UKK9	ADP-sugar pyrophosphatase	NUDT5			
P54845	Neural retina-specific leucine zipper protein	NRL			
O14786	Neuropilin-1	NRP1			
Q8WWG1	Pro-neuregulin-4, membrane-bound isoform	NRG4			
Q9NY59	Sphingomyelin phosphodiesterase 3	SMPD3			
Q9BV86	N-terminal Xaa-Pro-Lys N-methyltransferase 1	NTMT1			
Q96E93	Killer cell lectin-like receptor subfamily G member 1	KLRG1			
Q5T7B8	Kinesin-like protein KIF24	KIF24			
Q8IUC1	Keratin-associated protein 11-1	KRTAP11-1			
Q9H0B6	Kinesin light chain 2	KLC2			
P60371	Keratin-associated protein 10-6	KRTAP10-6			
O43526	Potassium voltage-gated channel subfamily KQT member 2	KCNQ2			
Q9NS87	Kinesin-like protein KIF15	KIF15			
P31323	cAMP-dependent protein kinase type II-beta regulatory subunit	PRKAR2B			
Q9NPI7	Lysine-rich coiled-coil protein 1	KRCC1			
P22001	Potassium voltage-gated channel subfamily A member 3	KCNA3			
Q9NVX7	Kelch repeat and BTB domain-containing protein 4	KBTBD4			
P04430	Immunoglobulin kappa variable 1-16	IGKV1-16			

Q9H9P8	L-2-hydroxyglutarate dehydrogenase, mitochondrial	L2HGDH			
Q96MP8	BTB/POZ domain-containing protein KCTD7	KCTD7			
A2NJV5	Immunoglobulin kappa variable 2-29	IGKV2-29			
Q9H825	mRNA N	METTL8			
Q8TDZ2		MICAL1			
Q8NHS3	Major facilitator superfamily domain-containing protein 8	MFSD8			
Q96EN8	Molybdenum cofactor sulfurase	MOCOS			
O15442	Metallophosphoesterase domain-containing protein 1	MPPED1			
P26038	Moesin	MSN			
Q5T3U5	ATP-binding cassette sub-family C member 10	ABCC10			
Q8WXB1	Protein N-lysine methyltransferase METTL21A	METTL21A			
Q8N9H8	Exonuclease mut-7 homolog	EXD3			
Q8N6N6	Protein NATD1	NATD1			
Q9UHF3	Putative N-acetyltransferase 8B	NAT8B			
P49321	Nuclear autoantigenic sperm protein	NASP			
Q9UKN7	Unconventional myosin-XV	MYO15A			
Q6PIF6	Unconventional myosin-VIIb	MYO7B			
P49821	NADH dehydrogenase	NDUFV1			
Q8NI08	Nuclear receptor coactivator 7	NCOA7			
P61583	Endogenous retrovirus group K member 5 Np9 protein	ERVK-5			
P78316	Nucleolar protein 14	NOP14			
Q9UM47	Neurogenic locus notch homolog protein 3	NOTCH3			
P46087	Probable 28S rRNA	NOP2			
Q92570	Nuclear receptor subfamily 4 group A member 3	NR4A3			
Q9HC29	Nucleotide-binding oligomerization domain-containing protein 2	NOD2			
Q9GZQ6	Neuropeptide FF receptor 1	NPFFR1			
P56730	Neurotrypsin	PRSS12			
Q16236	Nuclear factor erythroid 2-related factor 2	NFE2L2			
Q969S2	Endonuclease 8-like 2	NEIL2			
Q6VVB1	E3 ubiquitin-protein ligase NHLRC1	NHLRC1			
Q16795	NADH dehydrogenase	NDUFA9			
Q8NGD5	Olfactory receptor 4K14	OR4K14			
P0C626	Olfactory receptor 5G3	OR5G3			
Q9GZK3	Olfactory receptor 2B2	OR2B2			
P41145	Kappa-type opioid receptor	OPRK1			
Q96R67	Olfactory receptor 4C12	OR4C12			
Q8NGG4	Olfactory receptor 8H1	OR8H1			
Q96CB9	5-methylcytosine rRNA methyltransferase NSUN4	NSUN4			
O60890	Oligophrenin-1	OPHN1			
P03999	Short-wave-sensitive opsin 1	OPN1SW			
Q6UWF7	NXPE family member 4	NXPE4			
Q96DL1	NXPE family member 2	NXPE2			
Q8NH05	Olfactory receptor 4Q3	OR4Q3			
Q8IXS6	Paralemmin-2	PALM2			
Q9P1W9	Serine/threonine-protein kinase pim-2	PIM2			
Q8WV24	Pleckstrin homology-like domain family A member 1	PHLDA1			
Q68D20	Protein PMS2CL	PMS2CL			
Q8TBJ4	Phospholipid phosphatase-related protein type 1	PLPPR1			

Q8WZA1	Protein O-linked-mannose beta-1,2-N-acetylglucosaminyltransferase 1	POMGNT1			
Q7Z6K3	Protein prenyltransferase alpha subunit repeat-containing protein 1	PTAR1			
Q9UNM6	26S proteasome non-ATPase regulatory subunit 13	PSMD13			
Q92613	Protein Jade-3	JADE3			
Q9NQ38	Serine protease inhibitor Kazal-type 5	SPINK5			
Q86UX2	Inter-alpha-trypsin inhibitor heavy chain H5	ITIH5			
Q5JYT7	Uncharacterized protein KIAA1755	KIAA1755			
Q9Y624	Junctional adhesion molecule A	F11R			
O00410	Importin-5	IPO5			
O14896	Interferon regulatory factor 6	IRF6			
Q9UNL4	Inhibitor of growth protein 4	ING4			
O94953	Lysine-specific demethylase 4B	KDM4B			
Q96Q89	Kinesin-like protein KIF20B	KIF20B			
Q3LI67	Keratin-associated protein 6-3	KRTAP6-3			
A0A0C4DH26	Probable non-functional immunoglobulin kappa variable 6D-41	IGKV6D-41			
Q96ES6	Major facilitator superfamily domain-containing protein 3	MFSD3			
Q9UI43	rRNA methyltransferase 2, mitochondrial	MRM2			
Q7Z745	Maestro heat-like repeat-containing protein family member 2B	MROH2B			
Q8NEH6	Meiosis-specific nuclear structural protein 1	MNS1			
Q9H579	Protein MROH8	MROH8			
Q96QG7	Myotubularin-related protein 9	MTMR9			
Q9UHG3	Prenylcysteine oxidase 1	PCYOX1			
Q13562	Neurogenic differentiation factor	NEUROD1			
Q9NPC7	Myoneurin	MYNN			
Q9Y6X6	Unconventional myosin-XVI	MYO16			
Q13232	Nucleoside diphosphate kinase 3	NME3			
Q9NP98	Myozenin-1	MYOZ1			
A7E2Y1	Myosin-7B	MYH7B			
Q6P3W6	Neuroblastoma breakpoint family member 10	NBPF10			
Q02045	Myosin light chain 5	MYL5			
O14931	Natural cytotoxicity triggering receptor 3	NCR3			
Q8NCW5	NAD	NAXE			
Q7RTP0	Magnesium transporter NIPA1	NIPA1			
Q9Y618	Nuclear receptor corepressor 2	NCOR2			
P51513	RNA-binding protein Nova-1	NOVA1			
P35228	Nitric oxide synthase, inducible	NOS2			
Q9NPE2	Neugrin	NGRN			
Q14957	Glutamate receptor ionotropic, NMDA 2C	GRIN2C			
Q9H6R4	Nucleolar protein 6	NOL6			
Q12980	GATOR complex protein NPRL3	NPRL3			
O75380	NADH dehydrogenase	NDUFS6			
Q9BSC4	Nucleolar protein 10	NOL10			
Q8N9A8	Nuclear envelope phosphatase-regulatory subunit 1	CNEP1R1			
O75469	Nuclear receptor subfamily 1 group I member 2	NR1I2			
Q92617	Nuclear pore complex-interacting protein family member B3	NPIPB3			
Q6UXI9	Nephronectin	NPNT			
P52848	Bifunctional heparan sulfate N-deacetylase/N-sulfotransferase 1	NDST1			
Q7Z417	Nuclear fragile X mental retardation-interacting protein 2	NUFIP2			

Q8IVF1	NUT family member 2A	NUTM2A			
Q8NFH3	Nucleoporin Nup43	NUP43			
Q8WUM0	Nuclear pore complex protein Nup133	NUP133			
Q15615	Olfactory receptor 4D1	OR4D1			
P0C7N1	Olfactory receptor 8U8	OR8U8			
P35558	Phosphoenolpyruvate carboxykinase, cytosolic	PCK1			
Q8IY26	Phospholipid phosphatase 6	PLPP6			
P22103	Pneumadin				
Q14651	Plastin-1	PLS1			
Q96CS7	Pleckstrin homology domain-containing family B member 2	PLEKHB2			
P11686	Pulmonary surfactant-associated protein C	SFTPC			
Q3SYG4	Protein PTHB1	BBS9			
P46940	Ras GTPase-activating-like protein IQGAP1	IQGAP1			
Q107X0	Putative protein KRIP1	KLKP1			
Q96AC6	Kinesin-like protein KIFC2	KIFC2			
Q02241	Kinesin-like protein KIF23	KIF23			
Q86W47	Calcium-activated potassium channel subunit beta-4	KCNMB4			
Q7Z3B3	KAT8 regulatory NSL complex subunit 1	KANSL1			
Q9BV57	1,2-dihydroxy-3-keto-5-methylthiopentene dioxygenase	ADI1			
Q05655	Protein kinase C delta type	PRKCD			
Q3ZCV2	Lymphocyte expansion molecule	LEXM			
Q8N2G4	Ly6/PLAUR domain-containing protein 1	LYPD1			
Q96JK9	Mastermind-like protein 3	MAML3			
O60299	Leucine zipper putative tumor suppressor 3	LZTS3			
Q8WZA0	Protein LZIC	LZIC			
Q9H8H3	Methyltransferase-like protein 7A	METTL7A			
Q9NRE1	Matrix metalloproteinase-26	MMP26			
A0A0U1RRL7	Protein MMP24OS	MMP24OS			
P22897	Macrophage mannose receptor 1	MRC1			
Q96AM1	Mas-related G-protein coupled receptor member F	MRGPRF			
Q7L9L4	MOB kinase activator 1B	MOB1B			
Q8NCU8	Mitoregulin	MTLN			
P48039	Melatonin receptor type 1A	MTNR1A			
P57103	Sodium/calcium exchanger 3	SLC8A3			
P60323	Nanos homolog 3	NANOS3			
Q9BWU0	Kanadaptin	SLC4A1AP			
P22392	Nucleoside diphosphate kinase B	NME2			
P51970	NADH dehydrogenase	NDUFA8			
O00483	Cytochrome c oxidase subunit NDUFA4	NDUFA4			
Q02818	Nucleobindin-1	NUCB1			
Q6IQ23	Pleckstrin homology domain-containing family A member 7	PLEKHA7			
O95848	Uridine diphosphate glucose pyrophosphatase NUDT14	NUDT14			
Q5VVY1	Alpha N-terminal protein methyltransferase 1B	METTL11B			
Q8WZ94	Olfactory receptor 5P3	OR5P3			
Q8NGR2	Olfactory receptor 1L6	OR1L6			
Q15738	Sterol-4-alpha-carboxylate 3-dehydrogenase, decarboxylating	NSDHL			
Q8NGN0	Olfactory receptor 4D5	OR4D5			
Q8NH21	Olfactory receptor 4F5	OR4F5			
Q9P1Q5	Olfactory receptor 1A1	OR1A1			
Q9H4D5	Nuclear RNA export factor 3	NXF3			
Q8NGJ5	Olfactory receptor 51L1	OR51L1			
Q6NXP6	NADP-dependent oxidoreductase domain-containing protein 1	NOXRED1			

A6NHG9	Olfactory receptor 5H14	OR5H14			
Q8NGE0	Olfactory receptor 10AD1	OR10AD1			
Q9Y4A9	Olfactory receptor 10H1	OR10H1			
Q8NGK6	Olfactory receptor 52I1	OR52I1			
Q8NHC5	Olfactory receptor 14A16	OR14A16			
Q9UP65	Cytosolic phospholipase A2 gamma	PLA2G4C			
Q9Y619	Mitochondrial ornithine transporter 1	SLC25A15			
Q9Y5F8	Protocadherin gamma-B7	PCDHGB7			
Q9UNF0	Protein kinase C and casein kinase substrate in neurons protein 2	PACSIN2			
Q86YL7	Podoplanin	PDPN			
Q8WXW3	Progesterone-induced-blocking factor 1	PIBF1			
Q86YP4	Transcriptional repressor p66-alpha	GATAD2A			
Q13258	Prostaglandin D2 receptor	PTGDR			
O15530	3-phosphoinositide-dependent protein kinase 1	PDPK1			
Q9UL41	Paraneoplastic antigen Ma3	PNMA3			
O00592	Podocalyxin	PODXL			
P63133	Endogenous retrovirus group K member 8 Pol protein	ERVK-8			
Q96M27	Protein PRRC1	PRRC1			
Q8TC59	Piwi-like protein 2	PIWIL2			
P04070	Vitamin K-dependent protein C	PROC			
P07988	Pulmonary surfactant-associated protein B	SFTPB			
P12272	Parathyroid hormone-related protein	PTHLH			
Q5JS54	Proteasome assembly chaperone 4	PSMG4			
Q6GMV3	Putative peptidyl-tRNA hydrolase PTRHD1	PTRHD1			
Q92729	Receptor-type tyrosine-protein phosphatase U	PTPRU			
P0C7L1	Serine protease inhibitor Kazal-type 8	SPINK8			
P18084	Integrin beta-5	ITGB5			
Q0VF49	Uncharacterized protein KIAA2012	KIAA2012			
Q14532	Keratin, type I cuticular Ha2	KRT32			
P28290	Protein ITPRID2	ITPRID2			
P49441	Inositol polyphosphate 1-phosphatase	INPP1			
O15327	Inositol polyphosphate 4-phosphatase type II	INPP4B			
Q9BY32	Inosine triphosphate pyrophosphatase	ITPA			
P05111	Inhibin alpha chain	INHA			
A1A4Y4	Immunity-related GTPase family M protein	IRGM			
Q96NJ5	Kelch-like protein 32	KLHL32			
Q7Z7F0	KH homology domain-containing protein 4	KHDC4			
Q13557	Calcium/calmodulin-dependent protein kinase type II subunit delta	CAMK2D			
Q86SY8	Putative uncharacterized protein KTN1-AS1	KTN1-AS1			
Q96L42	Potassium voltage-gated channel subfamily H member 8	KCNH8			
P33176	Kinesin-1 heavy chain	KIF5B			
Q14012	Calcium/calmodulin-dependent protein kinase type 1	CAMK1			
Q9P2N6	KAT8 regulatory NSL complex subunit 3	KANSL3			
A5PL33	Protein KRBA1	KRBA1			
P30085	UMP-CMP kinase	CMPK1			

Q6ZNG9	KRAB-A domain-containing protein 2	KRBA2			
P0DSN7	Probable non-functional immunoglobulinn kappa variable 1D-37	IGKV1D-37			
P01611	Immunoglobulin kappa variable 1D-12	IGKV1D-12			
Q6UYE1	Leukemia-associated protein 7	DLEU7			
Q86YQ2	Putative BPIFA4P protein	BPIFA4P			
Q6DKI2	Galectin-9C	LGALS9C			
O75610	Left-right determination factor 1	LEFTY1			
Q9BWQ8	Protein lifeguard 2	FAIM2			
O95202	Mitochondrial proton/calcium exchanger protein	LETM1			
Q8IX19	Mast cell-expressed membrane protein 1	MCEMP1			
O43708	Maleylacetoacetate isomerase	GSTZ1			
Q49MG5	Microtubule-associated protein 9	MAP9			
Q13233	Mitogen-activated protein kinase kinase kinase 1	MAP3K1			
Q9BY19	Membrane-spanning 4-domains subfamily A member 8	MS4A8			
P57077	MAP3K7 C-terminal-like protein	MAP3K7CL			
Q9NX47	E3 ubiquitin-protein ligase MARCHF5	MARCHF5			
Q96JG8	Melanoma-associated antigen D4	MAGED4			
Q5J8X5	Membrane-spanning 4-domains subfamily A member 13	MS4A13			
P61244	Protein max	MAX			
P21145	Myelin and lymphocyte protein	MAL			
A6NDZ8	Putative methyl-CpG-binding domain protein 3-like 4	MBD3L4			
Q8IZL2	Mastermind-like protein 2	MAML2			
Q13495	Mastermind-like domain-containing protein 1	MAMLD1			
Q96EY5	Multivesicular body subunit 12A	MVB12A			
Q9H1A3	Methyltransferase-like protein 9	METTL9			
Q96QH8	Sperm acrosome-associated protein 5	SPACA5			
P43355	Melanoma-associated antigen 1	MAGEA1			
P43356	Melanoma-associated antigen 2	MAGEA2			
P20700	Lamin-B1	LMNB1			
Q96S90	LysM and putative peptidoglycan-binding domain-containing protein 1	LYSMD1			
O15479	Melanoma-associated antigen B2	MAGEB2			
Q92686	Neurogranin	NRGN			
Q8N912	Nutritionally-regulated adipose and cardiac enriched protein homolog	NRAC			
O75900	Matrix metalloproteinase-23	MMP23B			
P39900	Macrophage metalloelastase	MMP12			
B2RBV5	Putative MORF4 family-associated protein 1-like protein UPP				
Q13614	Myotubularin-related protein 2	MTMR2			
Q8N1T3	Unconventional myosin-Ih	MYO1H			
Q01538	Myelin transcription factor 1	MYT1			
Q8TD19	Serine/threonine-protein kinase Nek9	NEK9			
Q8NBF2	NHL repeat-containing protein 2	NHLRC2			
Q96G61	Diphosphoinositol polyphosphate phosphohydrolase 3-beta	NUDT11			
Q86X67	NAD	NUDT13			
Q0GE19	Sodium/bile acid cotransporter 7	SLC10A7			
P53370	Nucleoside diphosphate-linked moiety X motif 6	NUDT6			
P03915	NADH-ubiquinone oxidoreductase chain 5	MT-ND5			
Q9HC10	Otoferlin	OTOF			

Q86Y26	NUT family member 1	NUTM1			
P0C7N8	Olfactory receptor 9G9	OR9G9			
Q8NH94	Olfactory receptor 1L1	OR1L1			
Q9GZM6	Olfactory receptor 8D2	OR8D2			
Q8NGJ4	Olfactory receptor 52E2	OR52E2			
Q8NGN6	Olfactory receptor 10G7	OR10G7			
Q8NGX3	Olfactory receptor 10T2	OR10T2			
Q8NGK2	Olfactory receptor 52B4	OR52B4			
Q9H208	Olfactory receptor 10A2	OR10A2			
P0C6T2	Dolichyl-diphosphooligosaccharide--protein glycosyltransferase subunit 4	OST4			
Q8NGF4	Olfactory receptor 5AP2	OR5AP2			
Q8NGN3	Olfactory receptor 10G4	OR10G4			
Q9Y5P8	Serine/threonine-protein phosphatase 2A regulatory subunit B'' subunit beta	PPP2R3B			
P0DN81	Olfactory receptor 13C7	OR13C7			
A1E959	Odontogenic ameloblast-associated protein	ODAM			
Q9BYE7	Polycomb group RING finger protein 6	PCGF6			
Q5T2S8	Outer dynein arm-docking complex subunit 2	ODAD2			
Q9UGF5	Olfactory receptor 14J1	OR14J1			
Q8WVF1	Protein OSCP1	OSCP1			
Q8NGY9	Olfactory receptor 2L8	OR2L8			
Q9HCR9	Dual 3',5'-cyclic-AMP and -GMP phosphodiesterase 11A	PDE11A			
Q01064	Calcium/calmodulin-dependent 3',5'-cyclic nucleotide phosphodiesterase 1B	PDE1B			
Q9Y5H2	Protocadherin gamma-A11	PCDHGA11			
A0A0X1KG70	Olfactory receptor 4M2	OR4M2B			
P40855	Peroxisomal biogenesis factor 19	PEX19			
Q14442	Phosphatidylinositol N-acetylglucosaminyltransferase subunit H	PIGH			
Q8TCI5	Protein pitchfork	PIFO			
P54886	Delta-1-pyrroline-5-carboxylate synthase	ALDH18A1			
Q86X59	Putative uncharacterized protein LINC02875	LINC02875			
Q6VVX0	Vitamin D 25-hydroxylase	CYP2R1			
P61566	Endogenous retrovirus group K member 24 Env polyprotein	ERVK-24			
Q9NT22	EMILIN-3	EMILIN3			
Q6EEV4	DNA-directed RNA polymerase II subunit GRINL1A, isoforms 4/5	POLR2M			
Q9NQX3	Gephyrin	GPHN			
Q9NS66	Probable G-protein coupled receptor 173	GPR173			
P63092	Guanine nucleotide-binding protein G	GNAS			
Q7Z5G4	Golgin subfamily A member 7	GOLGA7			
P51810	G-protein coupled receptor 143	GPR143			
P48637	Glutathione synthetase	GSS			
P11169	Solute carrier family 2, facilitated glucose transporter member 3	SLC2A3			
Q71UI9	Histone H2A.V	H2AZ2			
Q9UBI9	Headcase protein homolog	HECA			
Q58FF7	Putative heat shock protein HSP 90-beta-3	HSP90AB3P			
P31269	Homeobox protein Hox-A9	HOXA9			
O60921	Checkpoint protein HUS1	HUS1			
Q99081	Transcription factor 12	TCF12			

P0DTW3	Probable non-functional immunoglobulin heavy variable 1-38-4	IGHV1-38-4			
Q9UJY1	Heat shock protein beta-8	HSPB8			
Q9BUJ2	Heterogeneous nuclear ribonucleoprotein U-like protein 1	HNRNPUL1			
P32881	Interferon alpha-8	IFNA8			
Q14005	Pro-interleukin-16	IL16			
Q9NWZ3	Interleukin-1 receptor-associated kinase 4	IRAK4			
Q8WWU7	Intelectin-2	ITLN2			
Q06033	Inter-alpha-trypsin inhibitor heavy chain H3	ITIH3			
P17275	Transcription factor jun-B	JUNB			
Q8NBE8	Kelch-like protein 23	KLHL23			
Q8N239	Kelch-like protein 34	KLHL34			
P15382	Potassium voltage-gated channel subfamily E member 1	KCNE1			
Q2KJY2	Kinesin-like protein KIF26B	KIF26B			
Q9NSB4	Keratin, type II cuticular Hb2	KRT82			
Q5SVS4	Kidney mitochondrial carrier protein 1	SLC25A30			
B7ZC32	Kinesin-like protein KIF28P	KIF28P			
Q9BYR2	Keratin-associated protein 4-5	KRTAP4-5			
Q9BYQ7	Keratin-associated protein 4-1	KRTAP4-1			
Q9NPI9	Inward rectifier potassium channel 16	KCNJ16			
Q6L8H1	Keratin-associated protein 5-4	KRTAP5-4			
Q3LI64	Keratin-associated protein 6-1	KRTAP6-1			
O60256	Phosphoribosyl pyrophosphate synthase-associated protein 2	PRPSAP2			
P04432	Immunoglobulin kappa variable 1D-39	IGKV1D-39			
P12532	Creatine kinase U-type, mitochondrial	CKMT1A			
Q9UBD5	Origin recognition complex subunit 3	ORC3			
Q9H6K4	Optic atrophy 3 protein	OPA3			
A0A2R8YFM6	Oocyte-secreted protein 3	OOSP3			
Q8NGI9	Olfactory receptor 5A2	OR5A2			
O43614	Orexin receptor type 2	HCRTR2			
Q16620	BDNF/NT-3 growth factors receptor	NTRK2			
P47890	Olfactory receptor 1G1	OR1G1			
P0DMB1	Proline-rich protein 23D2	PRR23D2			
Q96R28	Olfactory receptor 2M2	OR2M2			
Q6ZW49	PAX-interacting protein 1	PAXIP1			
Q96FC7	Phytanoyl-CoA hydroxylase-interacting protein-like	PHYHIPL			
Q8WUA2	Peptidyl-prolyl cis-trans isomerase-like 4	PPIL4			
Q92871	Phosphomannomutase 1	PMM1			
Q9UQG0	Endogenous retrovirus group K member 11 Pol protein	ERVK-11			
O14939	Phospholipase D2	PLD2			
Q96HM7	PC-esterase domain-containing protein 1B	PCED1B			
O95521	PRAME family member 1	PRAMEF1			
O95522	PRAME family member 12	PRAMEF12			
Q6P2Q9	Pre-mRNA-processing-splicing factor 8	PRPF8			
Q9NRG1	Phosphoribosyltransferase domain-containing protein 1	PRTFDC1			
Q9H7J1	Protein phosphatase 1 regulatory subunit 3E	PPP1R3E			
Q9UJV8	Purine-rich element-binding protein gamma	PURG			
O43653	Prostate stem cell antigen	PSCA			

Q9UKA9	Polypyrimidine tract-binding protein 2	PTBP2			
P78395	Melanoma antigen preferentially expressed in tumors	PRAME			
Q14671	Pumilio homolog 1	PUM1			
P21246	Pleiotrophin	PTN			
P0DMW2	NLR family pyrin domain-containing protein 2B	NLRP2B			
Q14807	Kinesin-like protein KIF22	KIF22			
Q9H7L2	Putative killer cell immunoglobulin-like receptor-like protein KIR3DX1	KIR3DX1			
Q03426	Mevalonate kinase	MVK			
A0A075B6S4	Immunoglobulin kappa variable 1D-17	IGKV1D-17			
P48357	Leptin receptor	LEPR			
Q13136	Liprin-alpha-1	PPFIA1			
O95237	Lecithin retinol acyltransferase	LRAT			
P54315	Inactive pancreatic lipase-related protein 1	PNLIPRP1			
Q9Y333	U6 snRNA-associated Sm-like protein LSm2	LSM2			
O95777	U6 snRNA-associated Sm-like protein LSm8	LSM8			
Q86TE4	Leucine zipper protein 2	LUZP2			
A0A075B6J1	Immunoglobulin lambda variable 5-37	IGLV5-37			
Q5TIA1	Meiosis inhibitor protein 1	MEI1			
Q9GZU1	Mucolipin-1	MCOLN1			
P0C7V9	Putative methyltransferase-like protein 15P1	METTL15P1			
Q9BUT9	MAPK regulated corepressor interacting protein 2	MCRIP2			
Q96EH3	Mitochondrial assembly of ribosomal large subunit protein 1	MALSU1			
Q03112	Histone-lysine N-methyltransferase MECOM	MECOM			
P41968	Melanocortin receptor 3	MC3R			
O43462	Membrane-bound transcription factor site-2 protease	MBTPS2			
Q8WZ33	MaFF-interacting protein	MAFIP			
Q8IV50	LysM and putative peptidoglycan-binding domain-containing protein 2	LYSMD2			
Q8WXG6	MAP kinase-activating death domain protein	MADD			
Q9NX63	MICOS complex subunit MIC19	CHCHD3			
P24347	Stromelysin-3	MMP11			
P55081	Microfibrillar-associated protein 1	MFAP1			
Q5T2T1	MAGUK p55 subfamily member 7	MPP7			
Q2M385	Macrophage-expressed gene 1 protein	MPEG1			
Q96LB1	Mas-related G-protein coupled receptor member X2	MRGPRX2			
Q9NRJ1	Protein MOST-1	C8orf17			
Q9HCE1	Helicase MOV-10	MOV10			
Q8NDA8	Maestro heat-like repeat-containing protein family member 1	MROH1			
O15374	Monocarboxylate transporter 5	SLC16A4			
Q6ZUA9	Maestro heat-like repeat family member 5	MROH5			
Q92887	Canalicular multispecific organic anion transporter 1	ABCC2			
Q70IA6	MOB kinase activator 2	MOB2			
Q96EF0	Myotubularin-related protein 8	MTMR8			
Q9Y216	Myotubularin-related protein 7	MTMR7			
Q96DR8	Mucin-like protein 1	MUCL1			
Q9H195	Mucin-3B	MUC3B			

Q9H903	Probable bifunctional methylenetetrahydrofolate dehydrogenase/cyclohydrolase 2	MTHFD2L			
O60934	Nibrin	NBN			
Q8N7R0	Putative homeobox protein NANOG2	NANOGP1			
Q9H009	Nascent polypeptide-associated complex subunit alpha-2	NACA2			
P04198	N-myc proto-oncogene protein	MYCN			
Q9Y5B8	Nucleoside diphosphate kinase 7	NME7			
Q8NEV4	Myosin-IIIa	MYO3A			
Q96S97	Myeloid-associated differentiation marker	MYADM			
O00533	Neural cell adhesion molecule L1-like protein	CHL1			
Q9H2Z4	Homeobox protein Nkx-2.4	NKX2-4			
Q96PY6	Serine/threonine-protein kinase Nek1	NEK1			
Q9UN36	Protein NDRG2	NDRG2			
Q96G74	OTU domain-containing protein 5	OTUD5			
Q16549	Proprotein convertase subtilisin/kexin type 7	PCSK7			
Q15032	R3H domain-containing protein 1	R3HDM1			
Q9H974	Queuine tRNA-ribosyltransferase accessory subunit 2	QTRT2			
O75192	Peroxisomal membrane protein 11A	PEX11A			
Q9H427	Potassium channel subfamily K member 15	KCNK15			
Q9BW19	Kinesin-like protein KIFC1	KIFC1			
A6NMS7	Leucine-rich repeat-containing protein 37A	LRRC37A			
O14960	Leukocyte cell-derived chemotaxin-2	LECT2			
A0A075B6S6	Immunoglobulin kappa variable 2D-30	IGKV2D-30			
Q6UWW0	Lipocalin-15	LCN15			
Q8IYD9	Lung adenoma susceptibility protein 2	LAS2			
Q5TDP6	Lengsin	LGSN			
Q6ZST4	Lipocalin-like 1 protein	LCNL1			
Q96DT0	Galectin-12	LGALS12			
Q5TA77	Late cornified envelope protein 3B	LCE3B			
Q8N967	Leucine-rich repeat and transmembrane domain-containing protein 2	LRTM2			
Q6P1Q0	LETM1 domain-containing protein 1	LETMD1			
P55344	Lens fiber membrane intrinsic protein	LIM2			
P28300	Protein-lysine 6-oxidase	LOX			
Q9BTT4	Mediator of RNA polymerase II transcription subunit 10	MED10			
O60663	LIM homeobox transcription factor 1-beta	LMX1B			
Q96T17	MAP7 domain-containing protein 2	MAP7D2			
P62312	U6 snRNA-associated Sm-like protein LSm6	LSM6			
Q9UK45	U6 snRNA-associated Sm-like protein LSm7	LSM7			
Q14814	Myocyte-specific enhancer factor 2D	MEF2D			
P50579	Methionine aminopeptidase 2	METAP2			
Q8N3Y3	LARGE xylosyl- and glucuronyltransferase 2	LARGE2			
Q6ZN16	Mitogen-activated protein kinase kinase kinase 15	MAP3K15			

Q16644	MAP kinase-activated protein kinase 3	MAPKAPK3			
Q14D04	Ventricular zone-expressed PH domain-containing protein homolog 1	VEPH1			
Q1X8D7	Leucine-rich repeat-containing protein 36	LRRC36			
O15021	Microtubule-associated serine/threonine-protein kinase 4	MAST4			
P35410	Mas-related G-protein coupled receptor MRG	MAS1L			
Q9NXL9	DNA helicase MCM9	MCM9			
Q14703	Membrane-bound transcription factor site-1 protease	MBTPS1			
Q13021	MAL-like protein	MALL			
P09238	Stromelysin-2	MMP10			
Q9BYG3	MKI67 FHA domain-interacting nucleolar phosphoprotein	NIFK			
P20585	DNA mismatch repair protein Msh3	MSH3			
Q29980	MHC class I polypeptide-related sequence B	MICB			
Q13421	Mesothelin	MSLN			
O60906	Sphingomyelin phosphodiesterase 2	SMPD2			
Q8NGC5	Olfactory receptor 6J1	OR6J1			
Q8IZS5	Orofacial cleft 1 candidate gene 1 protein	OFCC1			
Q8TDS5	Oxoeicosanoid receptor 1	OXER1			
Q9Y5N6	Origin recognition complex subunit 6	ORC6			
Q8NH69	Olfactory receptor 5W2	OR5W2			
Q9HBI0	Gamma-parvin	PARVG			
Q5VY43	Platelet endothelial aggregation receptor 1	PEAR1			
Q8TF01	Arginine/serine-rich protein PNISR	PNISR			
O14495	Phospholipid phosphatase 3	PLPP3			
Q96N28	PRELI domain containing protein 3A	PRELID3A			
Q9HBV1	Popeye domain-containing protein 3	POPDC3			
Q9NUX5	Protection of telomeres protein 1	POT1			
O15162	Phospholipid scramblase 1	PLSCR1			
Q9ULU4	Protein kinase C-binding protein 1	ZMYND8			
Q9NUQ2	1-acyl-sn-glycerol-3-phosphate acyltransferase epsilon	AGPAT5			
Q92620	Pre-mRNA-splicing factor ATP-dependent RNA helicase PRP16	DHX38			
Q96KF2	Small nuclear protein PRAC1	PRAC1			
Q8TAP8	Protein phosphatase 1 regulatory subunit 35	PPP1R35			
Q9NQV7	Histone-lysine N-methyltransferase PRDM9	PRDM9			
Q8TCU6	Phosphatidylinositol 3,4,5-trisphosphate-dependent Rac exchanger 1 protein	PREX1			
Q9Y6C5	Protein patched homolog 2	PTCH2			
O43242	26S proteasome non-ATPase regulatory subunit 3	PSMD3			
P18031	Tyrosine-protein phosphatase non-receptor type 1	PTPN1			
P29350	Tyrosine-protein phosphatase non-receptor type 6	PTPN6			
P38571	Lysosomal acid lipase/cholesteryl ester hydrolase	LIPA			
Q49AS3	Putative protein LRRC37A5P	LRRC37A5P			
O75145	Liprin-alpha-3	PPFIA3			
P00338	L-lactate dehydrogenase A chain	LDHA			

Q01650	Large neutral amino acids transporter small subunit 1	SLC7A5			
O95461	LARGE xylosyl- and glucuronyltransferase 1	LARGE1			
Q17RB8	LON peptidase N-terminal domain and RING finger protein 1	LONRF1			
Q15345	Leucine-rich repeat-containing protein 41	LRRC41			
Q14693	Phosphatidate phosphatase LPIN1	LPIN1			
Q96S06	Lipase maturation factor 1	LMF1			
P07948	Tyrosine-protein kinase Lyn	LYN			
O43324	Eukaryotic translation elongation factor 1 epsilon-1	EEF1E1			
Q9H9H5	MAP6 domain-containing protein 1	MAP6D1			
Q9UPN3	Microtubule-actin cross-linking factor 1, isoforms 1/2/3/5	MACF1			
Q99687	Homeobox protein Meis3	MEIS3			
Q14676	Mediator of DNA damage checkpoint protein 1	MDC1			
P49736	DNA replication licensing factor MCM2	MCM2			
Q8TD91	Melanoma-associated antigen C3	MAGEC3			
P33992	DNA replication licensing factor MCM5	MCM5			
O60732	Melanoma-associated antigen C1	MAGEC1			
Q9NUK0	Muscleblind-like protein 3	MBNL3			
Q5I0G3	Putative malate dehydrogenase	MDH1B			
P33993	DNA replication licensing factor MCM7	MCM7			
A6NE82	Putative methyl-CpG-binding domain protein 3-like 3	MBD3L3			
Q05195	Max dimerization protein 1	MXD1			
Q9BQG0	Myb-binding protein 1A	MYBBP1A			
Q5TF39	Sodium-dependent glucose transporter 1	MFSD4B			
Q6N075	Molybdate-anion transporter	MFSD5			
Q8NAN2	Mitoguardin 1	MIGA1			
Q13219	Pappalysin-1	PAPPA			
Q96S19	Methyltransferase-like 26	METTL26			
O15438	ATP-binding cassette sub-family C member 3	ABCC3			
P00387	NADH-cytochrome b5 reductase 3	CYB5R3			
Q7Z5D8	NANOG neighbor homeobox	NANOGNB			
A6NI61	Protein myomaker	MYMK			
Q9UBC5	Unconventional myosin-Ia	MYO1A			
Q9Y6K9	NF-kappa-B essential modulator	IKBKG			
P48552	Nuclear receptor-interacting protein 1	NRIP1			
Q9UHK0	Nuclear fragile X mental retardation-interacting protein 1	NUFIP1			
A1L443	NUT family member 2F	NUTM2F			
Q7L8S5	OTU domain-containing protein 6A	OTUD6A			
Q8WZ92	Olfactory receptor 5P2	OR5P2			
P03897	NADH-ubiquinone oxidoreductase chain 3	MT-ND3			
Q8NGD3	Olfactory receptor 4K5	OR4K5			
Q86W56	Poly	PARG			
Q92521	GPI mannosyltransferase 3	PIGB			
P34995	Prostaglandin E2 receptor EP1 subtype	PTGER1			
Q8TEM1	Nuclear pore membrane glycoprotein 210	NUP210			
Q9Y6A1	Protein O-mannosyl-transferase 1	POMT1			
Q9Y570	Protein phosphatase methylesterase 1	PPME1			

Q6S545	POTE ankyrin domain family member H	POTEH			
Q9H939	Proline-serine-threonine phosphatase-interacting protein 2	PSTPIP2			
B7ZBB8	Protein phosphatase 1 regulatory subunit 3G	PPP1R3G			
Q7Z2D5	2-lysophosphatidate phosphatase PLPPR4	PLPPR4			
O00329	Phosphatidylinositol 4,5-bisphosphate 3-kinase catalytic subunit delta isoform	PIK3CD			
O94827	Pleckstrin homology domain-containing family G member 5	PLEKHG5			
Q6NUQ1	RAD50-interacting protein 1	RINT1			
Q9H7P9	Pleckstrin homology domain-containing family G member 2	PLEKHG2			
Q6NYC1	Bifunctional arginine demethylase and lysyl-hydroxylase JMJD6	JMJD6			
Q7Z3Y9	Keratin, type I cytoskeletal 26	KRT26			
Q13797	Integrin alpha-9	ITGA9			
Q0VD86	Protein INCA1	INCA1			
P35568	Insulin receptor substrate 1	IRS1			
Q9NRX6	Protein kish-B	TMEM167B			
Q92985	Interferon regulatory factor 7	IRF7			
Q01101	Insulinoma-associated protein 1	INSM1			
O95279	Potassium channel subfamily K member 5	KCNK5			
P57682	Krueppel-like factor 3	KLF3			
P17252	Protein kinase C alpha type	PRKCA			
Q05513	Protein kinase C zeta type	PRKCZ			
A8MTY7	Keratin-associated protein 9-7	KRTAP9-7			
P05162	Galectin-2	LGALS2			
Q8WX39	Epididymal-specific lipocalin-9	LCN9			
Q7Z4F1	Low-density lipoprotein receptor-related protein 10	LRP10			
Q6VAB6	Kinase suppressor of Ras 2	KSR2			
Q8TF66	Leucine-rich repeat-containing protein 15	LRRC15			
A6NM36	Leucine-rich repeat-containing protein 30	LRRC30			
Q6NSJ5	Volume-regulated anion channel subunit LRRC8E	LRRC8E			
P09917	Polyunsaturated fatty acid 5-lipoxygenase	ALOX5			
Q6UWE0	E3 ubiquitin-protein ligase LRSAM1	LRSAM1			
P14151	L-selectin	SELL			
Q93052	Lipoma-preferred partner	LPP			
Q9Y4Y9	U6 snRNA-associated Sm-like protein LSm5	LSM5			
Q6ZN17	Protein lin-28 homolog B	LIN28B			
Q5U623	Activating transcription factor 7-interacting protein 2	ATF7IP2			
O15232	Matrilin-3	MATN3			
A0A075B6J6	Immunoglobulin lambda variable 3-22	IGLV3-22			
Q71F56	Mediator of RNA polymerase II transcription subunit 13-like	MED13L			
Q15648	Mediator of RNA polymerase II transcription subunit 1	MED1			
Q8N635	Meiosis-specific with OB domain-containing protein	MEIOB			
P20382	Pro-MCH	PMCH			
Q16626	Male-enhanced antigen 1	MEA1			
Q9Y316	Protein MEMO1	MEMO1			
Q9NZL9	Methionine adenosyltransferase 2 subunit beta	MAT2B			
O95402	Mediator of RNA polymerase II transcription subunit 26	MED26			

Q00987	E3 ubiquitin-protein ligase Mdm2	MDM2			
A6NJ08	Putative methyl-CpG-binding domain protein 3-like 5	MBD3L5			
Q5VYJ5	MAM and LDL-receptor class A domain-containing protein 1	MALRD1			
Q6P0Q8	Microtubule-associated serine/threonine-protein kinase 2	MAST2			
Q8N8X9	Protein mab-21-like 3	MAB21L3			
P53985	Monocarboxylate transporter 1	SLC16A1			
Q86TA1	MOB kinase activator 3B	MOB3B			
Q96RD1	Olfactory receptor 6C1	OR6C1			
Q16656	Nuclear respiratory factor 1	NRF1			
Q8NH40	Olfactory receptor 6S1	OR6S1			
O60683	Peroxisome biogenesis factor 10	PEX10			
Q99755	Phosphatidylinositol 4-phosphate 5-kinase type-1 alpha	PIP5K1A			
Q8TCT1	Phosphoethanolamine/phosphocholine phosphatase	PHOSPHO1			
O14737	Programmed cell death protein 5	PDCD5			
P49023	Paxillin	PXN			
P43088	Prostaglandin F2-alpha receptor	PTGFR			
Q8NBT0	POC1 centriolar protein homolog A	POC1A			
Q13951	Core-binding factor subunit beta	CBFB			
Q9H5P4	PDZ domain-containing protein 7	PDZD7			
Q969W9	Protein TMEPAI	PMEPA1			
Q6T4P5	Phospholipid phosphatase-related protein type 3	PLPPR3			
Q15195	Plasminogen-like protein A	PLGLA			
Q9UKL6	Phosphatidylcholine transfer protein	PCTP			
Q969H6	Ribonuclease P/MRP protein subunit POP5	POP5			
Q00G26	Perilipin-5	PLIN5			
Q9UF11	Pleckstrin homology domain-containing family B member 1	PLEKHB1			
Q9UBT6	DNA polymerase kappa	POLK			
P48651	Phosphatidylserine synthase 1	PTDSS1			
Q96BW5	Phosphotriesterase-related protein	PTER			
P61289	Proteasome activator complex subunit 3	PSME3			
Q12923	Tyrosine-protein phosphatase non-receptor type 13	PTPN13			
P17706	Tyrosine-protein phosphatase non-receptor type 2	PTPN2			
P23470	Receptor-type tyrosine-protein phosphatase gamma	PTPRG			
P53708	Integrin alpha-8	ITGA8			
P35527	Keratin, type I cytoskeletal 9	KRT9			
Q9UPP2	IQ motif and SEC7 domain-containing protein 3	IQSEC3			
P20701	Integrin alpha-L	ITGAL			
Q86VZ6	Juxtaposed with another zinc finger protein 1	JAZF1			
Q96M32	Adenylate kinase 7	AK7			
Q14641	Early placenta insulin-like peptide	INSL4			
P15260	Interferon gamma receptor 1	IFNGR1			
Q9UH77	Kelch-like protein 3	KLHL3			
Q9UJ96	Potassium voltage-gated channel subfamily G member 2	KCNG2			
Q9Y573	Actin-binding protein IPP	IPP			
Q9UQ07	MAPK/MAK/MRK overlapping kinase	MOK			
O75352	Mannose-P-dolichol utilization defect 1 protein	MPDU1			
P19823	Inter-alpha-trypsin inhibitor heavy chain H2	ITIH2			
Q8IX03	Protein KIBRA	WWC1			

P57789	Potassium channel subfamily K member 10	KCNK10			
Q8IUB9	Keratin-associated protein 19-1	KRTAP19-1			
Q04760	Lactoylglutathione lyase	GLO1			
P22612	cAMP-dependent protein kinase catalytic subunit gamma	PRKACG			
A0A0A0MRZ7	Immunoglobulin kappa variable 2D-26	IGKV2D-26			
Q5TA76	Late cornified envelope protein 3A	LCE3A			
Q13094	Lymphocyte cytosolic protein 2	LCP2			
Q9BV99	Leucine-rich repeat-containing protein 61	LRRC61			
Q6P9F7	Volume-regulated anion channel subunit LRRC8B	LRRC8B			
O00453	Leukocyte-specific transcript 1 protein	LST1			
O75325	Leucine-rich repeat neuronal protein 2	LRRN2			
A1A4G5	Leukemia NUP98 fusion partner 1	LNP1			
Q969L4	U7 snRNA-associated Sm-like protein LSm10	LSM10			
O75023	Leukocyte immunoglobulin-like receptor subfamily B member 5	LILRB5			
P0DP58	Ly-6/neurotoxin-like protein 1	LYNX1			
Q9P086	Mediator of RNA polymerase II transcription subunit 11	MED11			
O00754	Lysosomal alpha-mannosidase	MAN2B1			
P01717	Immunoglobulin lambda variable 3-25	IGLV3-25			
Q3KQU3	MAP7 domain-containing protein 1	MAP7D1			
Q9Y6D9	Mitotic spindle assembly checkpoint protein MAD1	MAD1L1			
Q15013	MAD2L1-binding protein	MAD2L1BP			
Q16048	Putative pro-MCH-like protein 1	PMCHL1			
O00255	Menin	MEN1			
Q9H992	E3 ubiquitin-protein ligase MARCHF7	MARCHF7			
Q68CR7	Leucine-rich repeat-containing protein 66	LRRC66			
Q9NXJ0	Membrane-spanning 4-domains subfamily A member 12	MS4A12			
C9JLW8	Mapk-regulated corepressor-interacting protein 1	MCRIP1			
P21439	Phosphatidylcholine translocator ABCB4	ABCB4			
Q4ZIN3	Membralin	TMEM259			
O43513	Mediator of RNA polymerase II transcription subunit 7	MED7			
Q7Z553	MAM domain-containing glycosylphosphatidylinositol anchor protein 2	MDGA2			
Q8N4S9	MARVEL domain-containing protein 2	MARVELD2			
Q8IYB1	Protein MB21D2	MB21D2			
P56270	Myc-associated zinc finger	MAZ			
Q9H7H0	Methyltransferase-like protein 17, mitochondrial	METTL17			
P30304	M-phase inducer phosphatase 1	CDC25A			
Q9NXD2	Myotubularin-related protein 10	MTMR10			
Q9Y483	Metal-response element-binding transcription factor 2	MTF2			
Q13126	S-methyl-5'-thioadenosine phosphorylase	MTAP			
Q6BCY4	NADH-cytochrome b5 reductase 2	CYB5R2			
Q86W28	NACHT, LRR and PYD domains-containing protein 8	NLRP8			
Q6ZNJ1	Neurobeachin-like protein 2	NBEAL2			

Q9GZM8	Nuclear distribution protein nudE-like 1	NDEL1			
Q8TBZ2	MYCBP-associated protein	MYCBPAP			
P43490	Nicotinamide phosphoribosyltransferase	NAMPT			
Q96MN2	NACHT, LRR and PYD domains-containing protein 4	NLRP4			
Q9HD67	Unconventional myosin-X	MYO10			
P56597	Nucleoside diphosphate kinase homolog 5	NME5			
O75592	E3 ubiquitin-protein ligase MYCBP2	MYCBP2			
Q5VZE5	N-alpha-acetyltransferase 35, NatC auxiliary subunit	NAA35			
P61599	N-alpha-acetyltransferase 20	NAA20			
Q8WV74	Nucleoside diphosphate-linked moiety X motif 8	NUDT8			
Q08J23	RNA cytosine C	NSUN2			
Q5VST9	Obscurin	OBSCN			
Q8NHA4	Olfactory receptor 2AE1	OR2AE1			
P30954	Olfactory receptor 10J1	OR10J1			
Q8NH80	Putative olfactory receptor 10D3	OR10D3			
O60403	Olfactory receptor 10H2	OR10H2			
P58181	Olfactory receptor 10A3	OR10A3			
Q8NH53	Olfactory receptor 52N1	OR52N1			
Q8NH08	Olfactory receptor 10AC1	OR10AC1			
Q8NGI7	Olfactory receptor 10V1	OR10V1			
Q96NG3	Outer dynein arm-docking complex subunit 4	ODAD4			
Q13438	Protein OS-9	OS9			
Q9NTK5	Obg-like ATPase 1	OLA1			
O94913	Pre-mRNA cleavage complex 2 protein Pcf11	PCF11			
Q9ULE6	Paladin	PALD1			
Q9NVE7	4'-phosphopantetheine phosphatase	PANK4			
P0C7N5	Olfactory receptor 8U9	OR8U9			
Q8N7H5	RNA polymerase II-associated factor 1 homolog	PAF1			
O76074	cGMP-specific 3',5'-cyclic phosphodiesterase	PDE5A			
Q15546	Monocyte to macrophage differentiation factor	MMD			
Q96DU9	Polyadenylate-binding protein 5	PABPC5			
Q9NZ20	Group 3 secretory phospholipase A2	PLA2G3			
O43422	52 kDa repressor of the inhibitor of the protein kinase	THAP12			
Q9P2E7	Protocadherin-10	PCDH10			
Q9Y5H9	Protocadherin alpha-2	PCDHA2			
Q96FE7	Phosphoinositide-3-kinase-interacting protein 1	PIK3IP1			
Q96MG8	Protein-L-isoaspartate O-methyltransferase domain-containing protein 1	PCMTD1			
Q86U86	Protein polybromo-1	PBRM1			
Q8TCG2	Phosphatidylinositol 4-kinase type 2-beta	PI4K2B			
P14210	Hepatocyte growth factor	HGF			
Q9BQS7	Hephaestin	HEPH			
Q9NYD6	Homeobox protein Hox-C10	HOXC10			
P04792	Heat shock protein beta-1	HSPB1			
P01814	Immunoglobulin heavy variable 2-70	IGHV2-70			
Q12988	Heat shock protein beta-3	HSPB3			
Q86UW8	Hyaluronan and proteoglycan link protein 4	HAPLN4			
C9JCN9	Heat shock factor-binding protein 1-like protein 1	HSBP1L1			

Q86XE5	4-hydroxy-2-oxoglutarate aldolase, mitochondrial	HOGA1			
Q86VS8	Protein Hook homolog 3	HOOK3			
P50135	Histamine N-methyltransferase	HNMT			
P35680	Hepatocyte nuclear factor 1-beta	HNF1B			
Q9H496	Torsin-1A-interacting protein 2, isoform IFRG15	TOR1AIP2			
Q14240	Eukaryotic initiation factor 4A-II	EIF4A2			
Q9H5V7	Zinc finger protein Pegasus	IKZF5			
P42701	Interleukin-12 receptor subunit beta-1	IL12RB1			
Q8IU81	Interferon regulatory factor 2-binding protein 1	IRF2BP1			
Q9BXS1	Isopentenyl-diphosphate delta-isomerase 2	IDI2			
O95256	Interleukin-18 receptor accessory protein	IL18RAP			
P02533	Keratin, type I cytoskeletal 14	KRT14			
P78504	Protein jagged-1	JAG1			
Q13349	Integrin alpha-D	ITGAD			
Q7RTS7	Keratin, type II cytoskeletal 74	KRT74			
Q8TAC2	Josephin-2	JOSD2			
Q9ULD6	Protein inturned	INTU			
Q13572	Inositol-tetrakisphosphate 1-kinase	ITPK1			
Q96PQ5	Putative protein phosphatase inhibitor 2-like protein 1	PPP1R2P1			
Q9Y5Q6	Insulin-like peptide INSL5	INSL5			
F8WCM5	Insulin, isoform 2	INS-IGF2			
P58166	Inhibin beta E chain	INHBE			
Q53HC5	Kelch-like protein 26	KLHL26			
Q14145	Kelch-like ECH-associated protein 1	KEAP1			
Q9UIH9	Krueppel-like factor 15	KLF15			
Q2TAC6	Kinesin-like protein KIF19	KIF19			
Q6JEL2	Kelch-like protein 10	KLHL10			
P60369	Keratin-associated protein 10-3	KRTAP10-3			
P60985	Keratinocyte differentiation-associated protein	KRTDAP			
Q674X7	Kazrin	KAZN			
Q6GTX8	Leukocyte-associated immunoglobulin-like receptor 1	LAIR1			
P25391	Laminin subunit alpha-1	LAMA1			
P24043	Laminin subunit alpha-2	LAMA2			
P19256	Lymphocyte function-associated antigen 3	CD58			
P50851	Lipopolysaccharide-responsive and beige-like anchor protein	LRBA			
Q86YC3	Transforming growth factor beta activator LRRC33	NRROS			
Q6ZSA7	Leucine-rich repeat-containing protein 55	LRRC55			
Q9BTT6	Leucine-rich repeat-containing protein 1	LRRC1			
Q6ZRR7	Leucine-rich repeat-containing protein 9	LRRC9			
P16233	Pancreatic triacylglycerol lipase	PNLIP			
Q8TBB1	E3 ubiquitin-protein ligase LNX	LNX1			
A0A075B6I3	Probable non-functional immunoglobulin lambda variable 11-55	IGLV11-55			
P62310	U6 snRNA-associated Sm-like protein LSm3	LSM3			
P01709	Immunoglobulin lambda variable 2-8	IGLV2-8			
Q96RQ3	Methylcrotonoyl-CoA carboxylase subunit alpha, mitochondrial	MCCC1			
Q86U10	60 kDa lysophospholipase	ASPG			

Q8NE86	Calcium uniporter protein, mitochondrial	MCU			
A0A0G2JS06	Immunoglobulin lambda variable 5-39	IGLV5-39			
O75095	Multiple epidermal growth factor-like domains protein 6	MEGF6			
Q9Y2X0	Mediator of RNA polymerase II transcription subunit 16	MED16			
Q3C1V0	Membrane-spanning 4-domains subfamily A member 18	MS4A18			
Q6P1A2	Lysophospholipid acyltransferase	LPCAT3			
Q567V2	Mpv17-like protein 2	MPV17L2			
P51512	Matrix metalloproteinase-16	MMP16			
Q96NR2	Putative MIR1-1HG-AS1	MIR1-1HG-AS1			
P26572	Alpha-1,3-mannosyl-glycoprotein 2-beta-N-acetylglucosaminyltransferase	MGAT1			
Q29983	MHC class I polypeptide-related sequence A	MICA			
Q16612	Neuronal regeneration-related protein	NREP			
Q1L6U9	Prostate-associated microseminoprotein	MSMP			
Q8NHP6	Motile sperm domain-containing protein 2	MOSPD2			
Q8NGY5	Olfactory receptor 6N1	OR6N1			
Q86UD1	Out at first protein homolog	OAF			
O95047	Olfactory receptor 2A4	OR2A4			
P57740	Nuclear pore complex protein Nup107	NUP107			
Q8NGM9	Olfactory receptor 8D4	OR8D4			
Q8NH51	Olfactory receptor 8K3	OR8K3			
Q9BRQ5	Protein orai-3	ORAI3			
Q8NGH9	Olfactory receptor 52E4	OR52E4			
Q9H1B4	Nuclear RNA export factor 5	NXF5			
Q14654	ATP-sensitive inward rectifier potassium channel 11	KCNJ11			
Q8NC69	BTB/POZ domain-containing protein KCTD6	KCTD6			
Q86V97	Kelch repeat and BTB domain-containing protein 6	KBTBD6			
P01624	Immunoglobulin kappa variable 3-15	IGKV3-15			
Q8N3X6	Ligand-dependent nuclear receptor corepressor-like protein	LCORL			
P09382	Galectin-1	LGALS1			
P17931	Galectin-3	LGALS3			
Q9Y4K0	Lysyl oxidase homolog 2	LOXL2			
P43657	Lysophosphatidic acid receptor 6	LPAR6			
Q6DN14	Multiple C2 and transmembrane domain-containing protein 1	MCTP1			
Q5VWZ2	Lysophospholipase-like protein 1	LYPLAL1			
Q8TE12	LIM homeobox transcription factor 1-alpha	LMX1A			
A0A0A0MRZ9	Immunoglobulin lambda variable 5-52	IGLV5-52			
O00339	Matrilin-2	MATN2			
A6NDP7	Myeloid-associated differentiation marker-like protein 2	MYADML2			
Q9UIS9	Methyl-CpG-binding domain protein 1	MBD1			
Q9UHC7	E3 ubiquitin-protein ligase makorin-1	MKRN1			
Q8NBR6	Ubiquitin carboxyl-terminal hydrolase MINDY-2	MINDY2			
P41218	Myeloid cell nuclear differentiation antigen	MNDA			

Q9UPX6	Major intrinsically disordered Notch2-binding receptor 1	MINAR1			
Q8TC71	Mitochondria-eating protein	SPATA18			
Q8TCY5	Melanocortin-2 receptor accessory protein	MRAP			
Q53F39	Metallophosphoesterase 1	MPPE1			
Q2M3A8	Putative uncharacterized protein MRGPRG-AS1	MRGPRG-AS1			
A8MV57	Putative mucosal pentraxin homolog	MPTX1			
Q15800	Methylsterol monooxygenase 1	MSMO1			
Q96LU7	Myelin regulatory factor-like protein	MYRFL			
Q8TE76	MORC family CW-type zinc finger protein 4	MORC4			
Q5SSG8	Mucin-21	MUC21			
Q04900	Sialomucin core protein 24	CD164			
Q9BV20	Methylthioribose-1-phosphate isomerase	MRI1			
P17050	Alpha-N-acetylgalactosaminidase	NAGA			
Q9NXR1	Nuclear distribution protein nudE homolog 1	NDE1			
Q6IPT4	NADH-cytochrome b5 reductase-like	CYB5RL			
P10242	Transcriptional activator Myb	MYB			
P12524	Protein L-Myc	MYCL			
O94832	Unconventional myosin-Id	MYO1D			
Q9NPC6	Myozenin-2	MYOZ2			
Q6IA69	Glutamine-dependent NAD	NADSYN1			
P0DPF3	Neuroblastoma breakpoint family member 9	NBPF9			
Q6NSJ0	Myogenesis-regulating glycosidase	MYORG			
P15172	Myoblast determination protein 1	MYOD1			
P52952	Homeobox protein Nkx-2.5	NKX2-5			
Q96SB3	Neurabin-2	PPP1R9B			
Q99608	Necdin	NDN			
Q92886	Neurogenin-1	NEUROG1			
Q9NQR4	Omega-amidase NIT2	NIT2			
Q13423	NAD	NNT			
Q9NZP6	Nuclear pore-associated protein 1	NPAP1			
P48645	Neuromedin-U	NMU			
Q9BS92	Protein NipSnap homolog 3B	NIPSNAP3B			
Q9Y6M9	NADH dehydrogenase	NDUFB9			
Q9HCQ7	Pro-FMRFamide-related neuropeptide VF	NPVF			
Q8IVI9	Nostrin	NOSTRIN			
Q9UI09	NADH dehydrogenase	NDUFA12			
P56975	Pro-neuregulin-3, membrane-bound isoform	NRG3			
Q9BQI9	Nuclear receptor-interacting protein 2	NRIP2			
P03923	NADH-ubiquinone oxidoreductase chain 6	MT-ND6			
Q63ZY6	Putative methyltransferase NSUN5C	NSUN5P2			
Q6IEZ7	Olfactory receptor 2T5	OR2T5			
Q9UBM4	Opticin	OPTC			
Q8NH00	Olfactory receptor 2T4	OR2T4			
Q9Y536	Peptidyl-prolyl cis-trans isomerase A-like 4A	PPIAL4A			
Q8N349	Olfactory receptor 2L13	OR2L13			
Q9UKS6	Protein kinase C and casein kinase substrate in neurons protein 3	PACSIN3			
Q9GZP0	Platelet-derived growth factor D	PDGFD			
Q9Y5H6	Protocadherin alpha-8	PCDHA8			
Q8IVL5	Prolyl 3-hydroxylase 2	P3H2			
Q15154	Pericentriolar material 1 protein	PCM1			

Q7Z4N8	Prolyl 4-hydroxylase subunit alpha-3	P4HA3			
Q9BTU6	Phosphatidylinositol 4-kinase type 2-alpha	PI4K2A			
Q6ZVD8	PH domain leucine-rich repeat-containing protein phosphatase 2	PHLPP2			
P01833	Polymeric immunoglobulin receptor	PIGR			
Q76NI1	Kinase non-catalytic C-lobe domain-containing protein 1	KNDC1			
Q9Y6N6	Laminin subunit gamma-3	LAMC3			
Q14739	Delta	LBR			
P00709	Alpha-lactalbumin	LALBA			
O00182	Galectin-9	LGALS9			
P0CW20	LIM and senescent cell antigen-like-containing domain protein 4	LIMS4			
Q8N7C0	Leucine-rich repeat-containing protein 52	LRRC52			
O75074	Low-density lipoprotein receptor-related protein 3	LRP3			
Q5VT99	Leucine-rich repeat-containing protein 38	LRRC38			
Q96KN1	Protein LRATD2	LRATD2			
Q8N9N7	Leucine-rich repeat-containing protein 57	LRRC57			
Q8TD94	Krueppel-like factor 14	KLF14			
O75197	Low-density lipoprotein receptor-related protein 5	LRP5			
O00370	LINE-1 retrotransposable element ORF2 protein				
A0A0A0MT76	Immunoglobulin lambda joining 1	IGLJ1			
Q8N2S1	Latent-transforming growth factor beta-binding protein 4	LTBP4			
O60449	Lymphocyte antigen 75	LY75			
Q9Y5X9	Endothelial lipase	LIPG			
P59901	Leukocyte immunoglobulin-like receptor subfamily A member 4	LILRA4			
Q6P5Q4	Leiomodin-2	LMOD2			
Q8NA19	Lethal	L3MBTL4			
Q8N8R3	Mitochondrial basic amino acids transporter	SLC25A29			
Q9BQD1	Putative pro-MCH-like protein 2	PMCHL2			
Q14244	Ensconsin	MAP7			
P42679	Megakaryocyte-associated tyrosine-protein kinase	MATK			
Q96JE9	Microtubule-associated protein 6	MAP6			
A1Z1Q3	ADP-ribose glycohydrolase MACROD2	MACROD2			
Q5VT66	Mitochondrial amidoxime-reducing component 1	MTARC1			
Q9UJ55	MAGE-like protein 2	MAGEL2			
Q9H8J5	MANSC domain-containing protein 1	MANSC1			
Q99698	Lysosomal-trafficking regulator	LYST			
Q5XG99	LysM and putative peptidoglycan-binding domain-containing protein 4	LYSMD4			
P43361	Melanoma-associated antigen 8	MAGEA8			
P43362	Melanoma-associated antigen 9	MAGEA9			
Q9HCC0	Methylcrotonoyl-CoA carboxylase beta chain, mitochondrial	MCCC2			
Q66K74	Microtubule-associated protein 1S	MAP1S			
O95382	Mitogen-activated protein kinase kinase kinase 6	MAP3K6			
A8MYZ0	Inactive ubiquitin carboxyl-terminal hydrolase MINDY-4B	MINDY4B			
Q96ER9	Mitochondrial potassium channel	CCDC51			
Q8N119	Matrix metalloproteinase-21	MMP21			

Q15759	Mitogen-activated protein kinase 11	MAPK11			
Q9BQP7	Mitochondrial genome maintenance exonuclease 1	MGME1			
Q6ZN04	RNA-binding protein MEX3B	MEX3B			
Q8TF71	Monocarboxylate transporter 10	SLC16A10			
Q6PF18	MORN repeat-containing protein 3	MORN3			
Q70IA8	MOB kinase activator 3C	MOB3C			
P52564	Dual specificity mitogen-activated protein kinase kinase 6	MAP2K6			
Q9BT17	Mitochondrial ribosome-associated GTPase 1	MTG1			
P21757	Macrophage scavenger receptor types I and II	MSR1			
Q99102	Mucin-4	MUC4			
O60682	Musculin	MSC			
Q8N307	Mucin-20	MUC20			
P84157	Matrix-remodeling-associated protein 7	MXRA7			
Q14764	Major vault protein	MVP			
Q9UBB6	Neurochondrin	NCDN			
Q9Y2K3	Myosin-15	MYH15			
P12882	Myosin-1	MYH1			
P14598	Neutrophil cytosol factor 1	NCF1			
O95944	Natural cytotoxicity triggering receptor 2	NCR2			
Q8N4C6	Ninein	NIN			
P08590	Myosin light chain 3	MYL3			
P26717	NKG2-C type II integral membrane protein	KLRC2			
P56181	NADH dehydrogenase	NDUFV3			
Q9NZG7	Ninjurin-2	NINJ2			
O14594	Neurocan core protein	NCAN			
Q0D2K0	Magnesium transporter NIPA4	NIPAL4			
Q9BU61	NADH dehydrogenase	NDUFAF3			
Q9P032	NADH dehydrogenase	NDUFAF4			
O95169	NADH dehydrogenase	NDUFB8			
O00476	Sodium-dependent phosphate transport protein 4	SLC17A3			
P16083	Ribosyldihydronicotinamide dehydrogenase	NQO2			
Q04721	Neurogenic locus notch homolog protein 2	NOTCH2			
Q13469	Nuclear factor of activated T-cells, cytoplasmic 2	NFATC2			
Q9NZF1	Placenta-specific gene 8 protein	PLAC8			
A0A024RBG1	Diphosphoinositol polyphosphate phosphohydrolase NUDT4B	NUDT4B			
Q9NPI5	Nicotinamide riboside kinase 2	NMRK2			
O95989	Diphosphoinositol polyphosphate phosphohydrolase 1	NUDT3			
Q9Y266	Nuclear migration protein nudC	NUDC			
Q6IEY1	Olfactory receptor 4F3/4F16/4F29	OR4F3;			
O14718	Visual pigment-like receptor peropsin	RRH			
Q8NHB7	Olfactory receptor 5K1	OR5K1			
Q8NGI4	Olfactory receptor 4D11	OR4D11			
E2RYF7	Protein PBMUCL2	HCG22			
Q8N1N4	Keratin, type II cytoskeletal 78	KRT78			
P78411	Iroquois-class homeodomain protein IRX-5	IRX5			
Q96EL1	PAK4-inhibitor INKA1	INKA1			
Q96FN5	Kinesin-like protein KIF12	KIF12			
Q9UEF7	Klotho	KL			
Q12840	Kinesin heavy chain isoform 5A	KIF5A			
P23919	Thymidylate kinase	DTYMK			
A4D1S0	Killer cell lectin-like receptor subfamily G member 2	KLRG2			
Q1ED39	Lysine-rich nucleolar protein 1	KNOP1			

Q03164	Histone-lysine N-methyltransferase 2A	KMT2A			
Q9UGP4	LIM domain-containing protein 1	LIMD1			
O00214	Galectin-8	LGALS8			
Q8N370	Large neutral amino acids transporter small subunit 4	SLC43A2			
Q969X1	Protein lifeguard 3	TMBIM1			
Q2Q1W2	E3 ubiquitin-protein ligase	TRIM71			
Q8TD35	Protein LKAAEAR1	LKAAEAR1			
Q0VGL1	Ragulator complex protein LAMTOR4	LAMTOR4			
Q99677	Lysophosphatidic acid receptor 4	LPAR4			
Q96JB6	Lysyl oxidase homolog 4	LOXL4			
P16109	P-selectin	SELP			
Q5W064	Lipase member J	LIPJ			
Q9H0V9	VIP36-like protein	LMAN2L			
A0A075B6K0	Immunoglobulin lambda variable 3-16	IGLV3-16			
P01718	Immunoglobulin lambda variable 3-27	IGLV3-27			
A0A075B6H9	Immunoglobulin lambda variable 4-69	IGLV4-69			
Q13503	Mediator of RNA polymerase II transcription subunit 21	MED21			
Q9UI95	Mitotic spindle assembly checkpoint protein MAD2B	MAD2L2			
P27816	Microtubule-associated protein 4	MAP4			
Q8N5U1	Membrane-spanning 4-domains subfamily A member 15	MS4A15			
Q9HAY2	Melanoma-associated antigen F1	MAGEF1			
Q14566	DNA replication licensing factor MCM6	MCM6			
Q6ZN01	MEF2-activating motif and SAP domain-containing transcriptional regulator	MAMSTR			
Q6ZNC8	Lysophospholipid acyltransferase	MBOAT1			
O60307	Microtubule-associated serine/threonine-protein kinase 3	MAST3			
Q8N653	Leucine-zipper-like transcriptional regulator 1	LZTR1			
Q8WWY6	Methyl-CpG-binding domain protein 3-like 1	MBD3L1			
P45983	Mitogen-activated protein kinase 8	MAPK8			
P53779	Mitogen-activated protein kinase 10	MAPK10			
Q9BY79	Membrane frizzled-related	MFRP			
Q8NGV5	Olfactory receptor 13D1	OR13D1			
O14733	Dual specificity mitogen-activated protein kinase kinase 7	MAP2K7			
Q7Z6M4	Transcription termination factor 4, mitochondrial	MTERF4			
Q02817	Mucin-2	MUC2			
Q8NEY1	Neuron navigator 1	NAV1			
Q8N165	Serine/threonine-protein kinase PDIK1L	PDIK1L			
Q9Y2G1	Myelin regulatory factor	MYRF			
Q14896	Myosin-binding protein C, cardiac-type	MYBPC3			
Q9UL68	Myelin transcription factor 1-like protein	MYT1L			
Q09161	Nuclear cap-binding protein subunit 1	NCBP1			
B4DH59	Neuroblastoma breakpoint family member 26	NBPF26			
P55160	Nck-associated protein 1-like	NCKAP1L			
Q9Y3T9	Nucleolar complex protein 2 homolog	NOC2L			

Q7RTR0	NACHT, LRR and PYD domains-containing protein 9	NLRP9			
O15226	NF-kappa-B-repressing factor	NKRF			
Q8NC60	Nitric oxide-associated protein 1	NOA1			
P61580	Endogenous retrovirus group K member 10 Np9 protein	ERVK-10			
O60936	Nucleolar protein 3	NOL3			
Q96D46	60S ribosomal export protein NMD3	NMD3			
E9PQ53	NADH dehydrogenase	NDUFC2-KCTD14			
Q00653	Nuclear factor NF-kappa-B p100 subunit	NFKB2			
Q8N130	Sodium-dependent phosphate transport protein 2C	SLC34A3			
P69849	Nodal modulator 3	NOMO3			
P13056	Nuclear receptor subfamily 2 group C member 1	NR2C1			
P19838	Nuclear factor NF-kappa-B p105 subunit	NFKB1			
Q8TAT6	Nuclear protein localization protein 4 homolog	NPLOC4			
Q6P3R8	Serine/threonine-protein kinase Nek5	NEK5			
Q99435	Protein kinase C-binding protein NELL2	NELL2			
Q96RB7	Olfactory receptor 5M11	OR5M11			
P0DN77	Medium-wave-sensitive opsin 2	OPN1MW2			
P37198	Nuclear pore glycoprotein p62	NUP62			
A6NMS3	Olfactory receptor 5K4	OR5K4			
Q96RQ9	L-amino-acid oxidase	IL4I1			
Q14123	Calcium/calmodulin-dependent 3',5'-cyclic nucleotide phosphodiesterase 1C	PDE1C			
A0A075B767	Peptidyl-prolyl cis-trans isomerase A-like 4H	PPIAL4H			
Q96RD7	Pannexin-1	PANX1			
Q9UM07	Protein-arginine deiminase type-	PADI4			
O76083	High affinity cGMP-specific 3',5'-cyclic phosphodiesterase 9A	PDE9A			
O00254	Proteinase-activated receptor 3	F2RL2			
Q8NF37	Lysophosphatidylcholine acyltransferase 1	LPCAT1			
Q8N4S7	Progestin and adipoQ receptor family member 4	PAQR4			
Q9UN67	Protocadherin beta-10	PCDHB10			
P01127	Platelet-derived growth factor subunit B	PDGFB			
P48426	Phosphatidylinositol 5-phosphate 4-kinase type-2 alpha	PIP4K2A			
O60346	PH domain leucine-rich repeat-containing protein phosphatase 1	PHLPP1			
Q3MUY2	Phosphatidylinositol N-acetylglucosaminyltransferase subunit Y	PIGY			
Q5T9C9	Phosphatidylinositol 4-phosphate 5-kinase-like protein 1	PIP5KL1			
P50479	PDZ and LIM domain protein 4	PDLIM4			
A2A3N6	Putative PIP5K1A and PSMD4-like protein	PIPSL			
P48547	Potassium voltage-gated channel subfamily C member 1	KCNC1			
Q86WU2	Probable D-lactate dehydrogenase, mitochondrial	LDHD			
Q50LG9	Leucine-rich repeat-containing protein 24	LRRC24			
P02545	Prelamin-A/C	LMNA			
A0A1B0GVX0	LITAF domain-containing protein	LITAFD			

A0A075B6K4	Immunoglobulin lambda variable 3-10	IGLV3-10			
A0A0A0MS00	Probable non-functional immunoglobulin lambda variable 3-32	IGLV3-32			
Q9BV36	Melanophilin	MLPH			
Q6ZN28	Metastasis-associated in colon cancer protein 1	MACC1			
Q9NU22	Midasin	MDN1			
P23490	Loricrin	LORICRIN			
Q9H6Y5	PDZ domain-containing protein MAGIX	MAGIX			
Q9BV79	Enoyl-	MECR			
Q9UDY8	Mucosa-associated lymphoid tissue lymphoma translocation protein 1	MALT1			
P02686	Myelin basic protein	MBP			
Q9BW11	Max dimerization protein 3	MXD3			
Q96GV9	Macrophage immunometabolism regulator	MACIR			
Q12866	Tyrosine-protein kinase Mer	MERTK			
Q9NPJ1	McKusick-Kaufman/Bardet-Biedl syndromes putative chaperonin	MKKS			
Q96IZ6	tRNA N	METTL2A			
Q8IYA7	Homeobox protein Mohawk	MKX			
P0DMT0	Myoregulin	MRLN			
O94851		MICAL2			
P14174	Macrophage migration inhibitory factor	MIF			
Q9H3H1	tRNA dimethylallyltransferase	TRIT1			
Q9GZW8	Membrane-spanning 4-domains subfamily A member 7	MS4A7			
Q2KHM9	Protein moonraker	KIAA0753			
Q9UDX5	Mitochondrial fission process protein 1	MTFP1			
P47224	Guanine nucleotide exchange factor MSS4	RABIF			
Q9NYA4	Myotubularin-related protein 4	MTMR4			
Q9Y3D2	Methionine-R-sulfoxide reductase B2, mitochondrial	MSRB2			
Q13585	Melatonin-related receptor	GPR50			
A8MVU1	Putative neutrophil cytosol factor 1C	NCF1C			
P14649	Myosin light chain 6B	MYL6B			
Q9UNW9	RNA-binding protein Nova-2	NOVA2			
Q96E22	Dehydrodolichyl diphosphate synthase complex subunit NUS1	NUS1			
Q8WTW4	GATOR complex protein NPRL2	NPRL2			
Q8NEJ9	Neuroguidin	NGDN			
P17568	NADH dehydrogenase	NDUFB7			
O95178	NADH dehydrogenase	NDUFB2			
P61916	NPC intracellular cholesterol transporter 2	NPC2			
Q96T66	Nicotinamide/nicotinic acid mononucleotide adenylyltransferase 3	NMNAT3			
Q9Y697	Cysteine desulfurase, mitochondrial	NFS1			
Q86WQ0	Nuclear receptor 2C2-associated protein	NR2C2AP			
Q6NW34	Nucleolus and neural progenitor protein	NEPRO			
Q5QGS0	Neurite extension and migration factor	NEXMIF			
Q9Y6Y0	Influenza virus NS1A-binding protein	IVNS1ABP			
O15239	NADH dehydrogenase	NDUFA1			
Q92597	Protein NDRG1	NDRG1			

P53384	Cytosolic Fe-S cluster assembly factor NUBP1	NUBP1			
Q9Y5A7	NEDD8 ultimate buster 1	NUB1			
P19338	Nucleolin	NCL			
P49757	Protein numb homolog	NUMB			
Q8NGN8	Putative olfactory receptor 4A4	OR4A4P			
O95007	Olfactory receptor 6B1	OR6B1			
Q2M2E3	Outer dense fiber protein 4	ODF4			
P47881	Olfactory receptor 3A1	OR3A1			
P04629	High affinity nerve growth factor receptor	NTRK1			
P54368	Ornithine decarboxylase antizyme 1	OAZ1			
Q9H488	GDP-fucose protein O-fucosyltransferase 1	POFUT1			
Q8NH81	Olfactory receptor 10G6	OR10G6			
A6NCV1	Olfactory receptor 6C74	OR6C74			
Q8NGX1	Olfactory receptor 2T34	OR2T34			
P0C629	Olfactory receptor 10J4	OR10J4			
Q8NGN5	Olfactory receptor 10G8	OR10G8			
A6NDH6	Olfactory receptor 5H15	OR5H15			
Q9H340	Olfactory receptor 51B6	OR51B6			
Q8NGJ9	Olfactory receptor 51T1	OR51T1			
Q8NGC9	Olfactory receptor 11H4	OR11H4			
Q8NGC7	Olfactory receptor 11H6	OR11H6			
Q9NRP0	Oligosaccharyltransferase complex subunit OSTC	OSTC			
Q9Y3B8	Oligoribonuclease, mitochondrial	REXO2			
Q9Y5G3	Protocadherin gamma-B1	PCDHGB1			
Q9Y5G5	Protocadherin gamma-A8	PCDHGA8			
Q8NH49	Olfactory receptor 4X1	OR4X1			
Q9Y5G0	Protocadherin gamma-B5	PCDHGB5			
P11940	Polyadenylate-binding protein 1	PABPC1			
Q9H8K7	ATPase PAAT	PAAT			
Q9BTK6	PAXIP1-associated glutamate-rich protein 1	PAGR1			
Q96RI0	Proteinase-activated receptor 4	F2RL3			
Q9BRP4	Proteasomal ATPase-associated factor 1	PAAF1			
Q13153	Serine/threonine-protein kinase PAK 1	PAK1			
Q9NTI5	Sister chromatid cohesion protein PDS5 homolog B	PDS5B			
O00398	Putative P2Y purinoceptor 10	P2RY10			
Q9Y5E3	Protocadherin beta-6	PCDHB6			
Q6V1P9	Protocadherin-23	DCHS2			
P0C8F1	Prostate and testis expressed protein 4	PATE4			
Q9NUP9	Protein lin-7 homolog C	LIN7C			
Q7Z7J7	LHFPL tetraspan subfamily member 4 protein	LHFPL4			
A0A0A0MRZ8	Immunoglobulin kappa variable 3D-11	IGKV3D-11			
O43261	Leukemia-associated protein 1	DLEU1			
Q5TA82	Late cornified envelope protein	LCE2D			
Q9BT23	LIM domain-containing protein 2	LIMD2			
P62502	Epididymal-specific lipocalin-6	LCN6			
O95214	Leptin receptor overlapping transcript-like 1	LEPROTL1			
Q5XKP0	MICOS complex subunit MIC13	MICOS13			
P35240	Merlin	NF2			
Q5JXM2	Methyltransferase-like protein 24	METTL24			
P08493	Matrix Gla protein	MGP			
P51511	Matrix metalloproteinase-15	MMP15			
Q8N4Q1	Mitochondrial intermembrane space import and assembly protein 40	CHCHD4			
Q7L4E1	Mitoguardin 2	MIGA2			

Q09328	Alpha-1,6-mannosylglycoprotein 6-beta-N-acetylglucosaminyltransferase A	MGAT5			
Q86V88	Magnesium-dependent phosphatase 1	MDP1			
P22894	Neutrophil collagenase	MMP8			
Q8IWA4	Mitofusin-1	MFN1			
P52701	DNA mismatch repair protein Msh6	MSH6			
Q3SYC2	2-acylglycerol O-acyltransferase 2	MOGAT2			
Q13724	Mannosyl-oligosaccharide glucosidase	MOGS			
P02795	Metallothionein-2	MT2A			
Q765P7	Protein MTSS 2	MTSS2			
Q8WUY8	N-acetyltransferase 14	NAT14			
P59047	NACHT, LRR and PYD domains-containing protein 5	NLRP5			
A0A1B0GUC4	Myocilin opposite strand protein	MYOCOS			
Q9GZZ1	N-alpha-acetyltransferase 50	NAA50			
O14974	Protein phosphatase 1 regulatory subunit 12A	PPP1R12A			
Q8TDC0	Myozenin-3	MYOZ3			
P28698	Myeloid zinc finger 1	MZF1			
Q9HD90	Neurogenic differentiation factor	NEUROD4			
Q9HC58	Sodium/potassium/calcium exchanger 3	SLC24A3			
Q9UQQ1	Aminopeptidase NAALADL1	NAALADL1			
Q8NFF2	Sodium/potassium/calcium exchanger 4	SLC24A4			
P15173	Myogenin	MYOG			
P16519	Neuroendocrine convertase 2	PCSK2			
Q9UFN0	Protein NipSnap homolog 3A	NIPSNAP3A			
F8W0I5	Nuclear pore complex-interacting protein family member B12	NPIPB12			
Q76FK4	Nucleolar protein 8	NOL8			
Q15063	Periostin	POSTN			
P28331	NADH-ubiquinone oxidoreductase 75 kDa subunit, mitochondrial	NDUFS1			
P01178	Oxytocin-neurophysin 1	OXT			
Q9UHB4	NADPH-dependent diflavin oxidoreductase 1	NDOR1			
Q9H093	NUAK family SNF1-like kinase 2	NUAK2			
O95299	NADH dehydrogenase	NDUFA10			
P18615	Negative elongation factor E	NELFE			
P62136	Serine/threonine-protein phosphatase PP1-alpha catalytic subunit	PPP1CA			
P12273	Prolactin-inducible protein	PIP			
Q8TB37	Iron-sulfur protein NUBPL	NUBPL			
P62140	Serine/threonine-protein phosphatase PP1-beta catalytic subunit	PPP1CB			
Q9HDD0	Phospholipase A and acyltransferase 1	PLAAT1			
Q9NV35	Nucleotide triphosphate diphosphatase NUDT15	NUDT15			
Q8IVD9	NudC domain-containing protein 3	NUDCD3			
Q5VT03	NUT family member 2D	NUTM2D			
Q8NG97	Olfactory receptor 2Z1	OR2Z1			
Q68BL8	Olfactomedin-like protein 2B	OLFML2B			
Q96CM4	Nucleoredoxin-like protein 1	NXNL1			
Q9Y2G5	GDP-fucose protein O-fucosyltransferase 2	POFUT2			
O95157	Neurexophilin-3	NXPH3			
Q8NGJ3	Olfactory receptor 52E1	OR52E1			
Q8NGC0	Olfactory receptor 5AU1	OR5AU1			

Q9BY11	Protein kinase C and casein kinase substrate in neurons protein 1	PACSIN1			
Q9NZ42	Gamma-secretase subunit PEN-2	PSENEN			
Q6UY27	Prostate and testis expressed protein 2	PATE2			
Q15102	Platelet-activating factor acetylhydrolase IB subunit alpha1	PAFAH1B3			
Q9P1Y6	PHD and RING finger domain-containing protein 1	PHRF1			
O14986	Phosphatidylinositol 4-phosphate 5-kinase type-1 beta	PIP5K1B			
Q9HCN3	Post-GPI attachment to proteins factor 6	PGAP6			
Q9BQS6	Heat shock protein beta-9	HSPB9			
A0A0A0MS15	Immunoglobulin heavy variable 3-49	IGHV3-49			
A8MVJ9	Putative histone PARylation factor 1-like				
Q9GZV7	Hyaluronan and proteoglycan link protein 2	HAPLN2			
Q6UXD1	Histidine-rich carboxyl terminus protein 1	HRCT1			
Q96LI6	Heat shock transcription factor, Y-linked	HSFY1			
P01889	HLA class I histocompatibility antigen, B alpha chain	HLA-B			
P29218	Inositol monophosphatase 1	IMPA1			
Q8TDY8	Immunoglobulin superfamily DCC subclass member 4	IGDCC4			
Q96G21	U3 small nucleolar ribonucleoprotein protein IMP4	IMP4			
Q04637	Eukaryotic translation initiation factor 4 gamma 1	EIF4G1			
Q6UX52	Protein IL-40	C17orf99			
Q659A1	Little elongation complex subunit 2	ICE2			
P05112	Interleukin-4	IL4			
P54105	Methylosome subunit pICln	CLNS1A			
P38570	Integrin alpha-E	ITGAE			
P04264	Keratin, type II cytoskeletal 1	KRT1			
Q8N201	Integrator complex subunit 1	INTS1			
Q9Y5U9	Immediate early response 3-interacting protein 1	IER3IP1			
Q15323	Keratin, type I cuticular Ha1	KRT31			
Q14624	Inter-alpha-trypsin inhibitor heavy chain H4	ITIH4			
P41236	Protein phosphatase inhibitor 2	PPP1R2			
Q8IWB1	Inositol 1,4,5-trisphosphate receptor-interacting protein	ITPRIP			
Q6NXS1	Protein phosphatase inhibitor 2 family member B	PPP1R2B			
Q8IU57	Interferon lambda receptor 1	IFNLR1			
Q96T55	Potassium channel subfamily K member 16	KCNK16			
P11801	Serine/threonine-protein kinase H1	PSKH1			
P0C5Y4	Keratin-associated protein 1-4	KRTAP1-4			
Q14533	Keratin, type II cuticular Hb1	KRT81			
A8MUX0	Keratin-associated protein 16-1	KRTAP16-1			
P07288	Prostate-specific antigen	KLK3			
Q8N6L1	Keratinocyte-associated protein 2	KRTCAP2			
Q07627	Keratin-associated protein 1-1	KRTAP1-1			
Q92953	Potassium voltage-gated channel subfamily B member 2	KCNB2			
P46020	Phosphorylase b kinase regulatory subunit alpha, skeletal muscle isoform	PHKA1			
O75690	Keratin-associated protein 5-8	KRTAP5-8			

O94806	Serine/threonine-protein kinase D3	PRKD3			
Q16719	Kynureninase	KYNU			
Q9H2C1	LIM/homeobox protein Lhx5	LHX5			
Q53H82	Endoribonuclease LACTB2	LACTB2			
Q96FE5	Leucine-rich repeat and immunoglobulin-like domain-containing nogo receptor-interacting protein 1	LINGO1			
O75112	LIM domain-binding protein 3	LDB3			
Q496Y0	LON peptidase N-terminal domain and RING finger protein 3	LONRF3			
Q8N5H3	Leucine repeat adapter protein 25	FAM89B			
Q9UHA4	Ragulator complex protein LAMTOR3	LAMTOR3			
Q8IVV2	Lipoxygenase homology domain-containing protein 1	LOXHD1			
Q5VUJ6	Leucine-rich repeat and calponin homology domain-containing protein 2	LRCH2			
Q6UWN5	Ly6/PLAUR domain-containing protein 5	LYPD5			
Q9NTJ4	Alpha-mannosidase 2C1	MAN2C1			
P01721	Immunoglobulin lambda variable 6-57	IGLV6-57			
O60336	Mitogen-activated protein kinase-binding protein 1	MAPKBP1			
Q9BTE3	Mini-chromosome maintenance complex-binding protein	MCMBP			
Q96PG1	Putative membrane-spanning 4-domains subfamily A member 4E	MS4A4E			
Q5VSG8	Glycoprotein endo-alpha-1,2-mannosidase-like protein	MANEAL			
Q8NHZ7	Methyl-CpG-binding domain protein 3-like 2	MBD3L2			
Q6P0N0	Mis18-binding protein 1	MIS18BP1			
Q8N5G2	Macoilin	MACO1			
P43363	Melanoma-associated antigen 10	MAGEA10			
P43364	Melanoma-associated antigen 11	MAGEA11			
A8MXT2	Melanoma-associated antigen B17	MAGEB17			
Q9H3L0	Cobalamin trafficking protein	MMADHC			
Q8N3F8	MICAL-like protein 1	MICALL1			
A6NG13	Alpha-1,3-mannosyl-glycoprotein 4-beta-N-acetylglucosaminyltransferase-like protein MGAT4D	MGAT4D			
Q02252	Methylmalonate-semialdehyde dehydrogenase	ALDH6A1			
Q9NYZ2	Mitoferrin-1	SLC25A37			
Q14CX5	Transmembrane protein 180	MFSD13A			
Q14168	MAGUK p55 subfamily member 2	MPP2			
P10515	Dihydrolipoyllysine-residue acetyltransferase component of pyruvate dehydrogenase complex, mitochondrial	DLAT			
Q9NUU6	Inactive ubiquitin thioesterase OTULINL	OTULINL			
P29728	2'-5'-oligoadenylate synthase 2	OAS2			
Q6IEV9	Olfactory receptor 4C11	OR4C11			
Q9BVL2	Nucleoporin p58/p45	NUP58			
Q15116	Programmed cell death protein 1	PDCD1			
Q14943	Killer cell immunoglobulin-like receptor 3DS1	KIR3DS1			
A0A0B4J1Z2	Immunoglobulin kappa variable 1D-43	IGKV1D-43			
Q86VI4	Lysosomal-associated transmembrane protein 4B	LAPTM4B			

Q8N145	Leucine-rich repeat LGI family member 3	LGI3			
Q6JVE6	Epididymal-specific lipocalin-10	LCN10			
Q9HC24	Protein lifeguard 4	TMBIM4			
Q9H9A6	Leucine-rich repeat-containing protein 40	LRRC40			
Q96II8	DISP complex protein LRCH3	LRCH3			
Q16873	Leukotriene C4 synthase	LTC4S			
A6NJW4	Leucine-rich repeat-containing protein 3C	LRRC3C			
O94822	E3 ubiquitin-protein ligase listerin	LTN1			
O75022	Leukocyte immunoglobulin-like receptor subfamily B member 3	LILRB3			
A0A075B6J9	Immunoglobulin lambda variable 2-18	IGLV2-18			
Q96G25	Mediator of RNA polymerase II transcription subunit 8	MED8			
A0A075B6K6	Immunoglobulin lambda variable 4-3	IGLV4-3			
A0A075B6I1	Immunoglobulin lambda variable 4-60	IGLV4-60			
Q9P2G4	Microtubule-associated protein	MAP10			
Q8N8E1	Putative uncharacterized protein encoded by MAPKAPK5-AS1	MAPKAPK5-AS1			
Q8TC05	Nuclear protein MDM1	MDM1			
Q9Y2U5	Mitogen-activated protein kinase kinase kinase 2	MAP3K2			
Q96EZ8	Microspherule protein 1	MCRS1			
O60337	E3 ubiquitin-protein ligase MARCHF6	MARCHF6			
Q8TD90	Melanoma-associated antigen E2	MAGEE2			
Q9P0N8	E3 ubiquitin-protein ligase MARCHF2	MARCHF2			
P40926	Malate dehydrogenase, mitochondrial	MDH2			
P32245	Melanocortin receptor 4	MC4R			
Q9Y250	Leucine zipper putative tumor suppressor 1	LZTS1			
Q7Z3D4	LysM and putative peptidoglycan-binding domain-containing protein 3	LYSMD3			
P59773	Major intrinsically disordered NOTCH2-binding receptor 1-like	MINAR2			
Q9UHC1	DNA mismatch repair protein	MLH3			
O75425	Motile sperm domain-containing protein 3	MOSPD3			
O75081	Protein CBFA2T3	CBFA2T3			
Q96GX9	Methylthioribulose-1-phosphate dehydratase	APIP			
Q06455	Protein CBFA2T1	RUNX1T1			
Q93083	Metallothionein-1L	MT1L			
P11137	Microtubule-associated protein 2	MAP2			
P58546	Myotrophin	MTPN			
Q9HC84	Mucin-5B	MUC5B			
Q147X3	N-alpha-acetyltransferase 30	NAA30			
Q99972	Myocilin	MYOC			
P54296	Myomesin-2	MYOM2			
Q9UBF9	Myotilin	MYOT			
Q14686	Nuclear receptor coactivator 6	NCOA6			
Q9Y2I6	Ninein-like protein	NINL			
P25208	Nuclear transcription factor Y subunit beta	NFYB			
Q8WTT2	Nucleolar complex protein 3 homolog	NOC3L			
Q9NVX2	Notchless protein homolog 1	NLE1			
Q8N0W4	Neuroligin-4, X-linked	NLGN4X			
Q9HAN9	Nicotinamide/nicotinic acid mononucleotide adenylyltransferase 1	NMNAT1			

Q13224	Glutamate receptor ionotropic, NMDA 2B	GRIN2B			
Q96NT1	Nucleosome assembly protein 1-like 5	NAP1L5			
P29475	Nitric oxide synthase, brain	NOS1			
Q9GZQ4	Neuromedin-U receptor 2	NMUR2			
A6NJU9	Nuclear pore complex-interacting protein family member B13	NPIPB13			
Q9BZQ8	Protein Niban 1	NIBAN1			
Q5T2W1	Na	PDZK1			
Q15233	Non-POU domain-containing octamer-binding protein	NONO			
Q92542	Nicastrin	NCSTN			
O43674	NADH dehydrogenase	NDUFB5			
Q9Y6K5	2'-5'-oligoadenylate synthase 3	OAS3			
Q8NGU4	Putative olfactory receptor 2I1	OR2I1P			
Q96AB6	Protein N-terminal asparagine amidohydrolase	NTAN1			
Q9UN70	Protocadherin gamma-C3	PCDHGC3			
Q9NY56	Odorant-binding protein 2a	OBP2A			
Q9Y5H3	Protocadherin gamma-A10	PCDHGA10			
Q9NX31	Oxidative stress-responsive serine-rich protein 1	OSER1			
Q9Y5H1	Protocadherin gamma-A2	PCDHGA2			
Q13370	cGMP-inhibited 3',5'-cyclic phosphodiesterase B	PDE3B			
P35232	Prohibitin	PHB			
Q8WUB8	PHD finger protein 10	PHF10			
Q9UPP1	Histone lysine demethylase PHF8	PHF8			
Q9UKI9	POU domain, class 2, transcription factor 3	POU2F3			
Q9NQR1	N-lysine methyltransferase	KMT5A			
Q96MU8	Kremen protein 1	KREMEN1			
Q9UJU2	Lymphoid enhancer-binding factor 1	LEF1			
A0A075B6S9	Probable non-functional immunoglobulinn kappa variable 1-37	IGKV1-37			
Q4G0J3	La-related protein 7	LARP7			
P42702	Leukemia inhibitory factor receptor	LIFR			
Q7Z4I7	LIM and senescent cell antigen-like-containing domain protein 2	LIMS2			
P48059	LIM and senescent cell antigen-like-containing domain protein 1	LIMS1			
Q8NES3	Beta-1,3-N-acetylglucosaminyltransferase lunatic fringe	LFNG			
Q8MH63	Putative L-type amino acid transporter 1-like protein MLAS	SLC7A5P1			
Q2VPJ9	Leucine-rich repeat-containing protein 75B	LRRC75B			
Q9BQK8	Phosphatidate phosphatase LPIN3	LPIN3			
A0A1B0GTW7	Leishmanolysin-like peptidase 2	LMLN2			
P10253	Lysosomal alpha-glucosidase	GAA			
P01699	Immunoglobulin lambda variable 1-44	IGLV1-44			
P08235	Mineralocorticoid receptor	NR3C2			
A8K0S8	Putative homeobox protein Meis3-like 2	MEIS3P2			
Q9Y2Q5	Ragulator complex protein LAMTOR2	LAMTOR2			
Q9NYL2	Mitogen-activated protein kinase kinase kinase 20	MAP3K20			
Q8NA82	Probable E3 ubiquitin-protein ligase MARCHF10	MARCHF10			
Q9NR56	Muscleblind-like protein 1	MBNL1			

Q96QZ7	Membrane-associated guanylate kinase, WW and PDZ domain-containing protein 1	MAGI1			
P20794	Serine/threonine-protein kinase MAK	MAK			
Q9UBB5	Methyl-CpG-binding domain protein 2	MBD2			
Q96A59	MARVEL domain-containing protein 3	MARVELD3			
Q86XA0	Methyltransferase-like protein 23	METTL23			
P19105	Myosin regulatory light chain 12A	MYL12A			
Q502X0	MORN repeat-containing protein 2	MORN2			
O15091	Mitochondrial ribonuclease P catalytic subunit	PRORP			
Q5HYI7	Metaxin-3	MTX3			
P59045	NACHT, LRR and PYD domains-containing protein 11	NLRP11			
Q8N8M0	Probable N-acetyltransferase 16	NAT16			
A0A1B0GTQ4	Protein myomixer	MYMX			
P01106	Myc proto-oncogene protein	MYC			
Q5TBK1	NEDD4-binding protein 2-like 1	N4BP2L1			
O75414	Nucleoside diphosphate kinase 6	NME6			
Q12965	Unconventional myosin-Ie	MYO1E			
Q9H6N6	Putative uncharacterized protein MYH16	MYH16			
P0DPF2	Neuroblastoma breakpoint family member 20	NBPF20			
Q9BUA6	Myosin regulatory light chain 10	MYL10			
Q9UKX2	Myosin-2	MYH2			
Q5T1S8	Noncompact myelin-associated protein	NCMAP			
Q8IY84	Serine/threonine-protein kinase NIM1	NIM1K			
P20929	Nebulin	NEB			
Q9H841	NIPA-like protein 2	NIPAL2			
P55769	NHP2-like protein 1	SNU13			
Q99742	Neuronal PAS domain-containing protein 1	NPAS1			
P29474	Nitric oxide synthase, endothelial	NOS3			
Q9NQX5	Neural proliferation differentiation and control protein 1	NPDC1			
Q14596	Next to BRCA1 gene 1 protein	NBR1			
Q9NPE3	H/ACA ribonucleoprotein complex subunit 3	NOP10			
O15130	Pro-FMRFamide-related neuropeptide FF	NPFF			
Q8NFW8	N-acylneuraminate cytidylyltransferase	CMAS			
O00482	Nuclear receptor subfamily 5 group A member 2	NR5A2			
Q9UQ49	Sialidase-3	NEU3			
O00634	Netrin-3	NTN3			
Q9HB63	Netrin-4	NTN4			
P51955	Serine/threonine-protein kinase Nek2	NEK2			
P58400	Neurexin-1-beta	NRXN1			
P03901	NADH-ubiquinone oxidoreductase chain 4L	MT-ND4L			
Q8NGG3	Olfactory receptor 5T3	OR5T3			
P13725	Oncostatin-M	OSM			
Q9ULI1	NACHT and WD repeat domain-containing protein 2	NWD2			
P58182	Olfactory receptor 12D2	OR12D2			
Q8NH59	Olfactory receptor 51Q1	OR51Q1			
Q9Y5G1	Protocadherin gamma-B3	PCDHGB3			
Q9ULW8	Protein-arginine deiminase type-	PADI3			
P56589	Peroxisomal biogenesis factor 3	PEX3			

Q15126	Phosphomevalonate kinase	PMVK			
Q7Z3K3	Pogo transposable element with ZNF domain	POGZ			
Q9BZG2	Testicular acid phosphatase	ACP4			
O14730	Serine/threonine-protein kinase RIO3	RIOK3			
Q6DKI1	60S ribosomal protein L7-like 1	RPL7L1			
Q9NWU5	39S ribosomal protein L22, mitochondrial	MRPL22			
P24928	DNA-directed RNA polymerase II subunit RPB1	POLR2A			
O43567	E3 ubiquitin-protein ligase RNF13	RNF13			
O75818	Ribonuclease P protein subunit p40	RPP40			
P62979	Ubiquitin-40S ribosomal protein S27a	RPS27A			
O60499	Syntaxin-10	STX10			
Q14247	Src substrate cortactin	CTTN			
O15056	Synaptojanin-2	SYNJ2			
Q96GW9	Methionine--tRNA ligase, mitochondrial	MARS2			
Q8N5T2	TBC1 domain family member 19	TBC1D19			
P04350	Tubulin beta-4A chain	TUBB4A			
Q0IIM8	TBC1 domain family member 8B	TBC1D8B			
Q15545	Transcription initiation factor TFIID subunit 7	TAF7			
Q5T011	KICSTOR complex protein SZT2	SZT2			
Q9UPU7	TBC1 domain family member 2B	TBC1D2B			
Q9NYV7	Taste receptor type 2 member 16	TAS2R16			
D6RBQ6	Ubiquitin carboxyl-terminal hydrolase 17-like protein 17	USP17L17			
P98155	Very low-density lipoprotein receptor	VLDLR			
Q9H347	Ubiquilin-3	UBQLN3			
P45880	Voltage-dependent anion-selective channel protein 2	VDAC2			
Q0P140	Putative uncharacterized protein HSD52	HSD52			
A8MTW9	Putative uncharacterized protein ENSP00000380674				
Q6ZVU0	Putative uncharacterized protein FLJ42102				
Q8N5A5	Zinc finger CCCH-type with G patch domain-containing protein	ZGPAT			
A8MWP6	Uncharacterized protein ENSP00000382042				
Q9Y6Q3	Zinc finger protein 37 homolog	ZFP37			
A8MT70	Zinc finger B-box domain-containing protein 1	ZBBX			
Q9H869	YY1-associated protein 1	YY1AP1			
Q8N8Y5	Zinc finger protein 41 homolog	ZFP41			
Q3KNS6	Zinc finger protein 829	ZNF829			
Q7Z570	Zinc finger protein 804A	ZNF804A			
P17024	Zinc finger protein 20	ZNF20			
A6NJL1	Zinc finger and SCAN domain-containing protein 5B	ZSCAN5B			
O43257	Zinc finger HIT domain-containing protein 1	ZNHIT1			
Q96C00	Zinc finger and BTB domain-containing protein 9	ZBTB9			
Q9UGI0	Ubiquitin thioesterase ZRANB1	ZRANB1			
Q96SE7	Zinc finger protein 347	ZNF347			
Q96IQ9	Zinc finger protein 414	ZNF414			
Q96K83	Zinc finger protein 521	ZNF521			
Q8TD17	Zinc finger protein 398	ZNF398			
Q9NX65	Zinc finger and SCAN domain-containing protein 32	ZSCAN32			
Q9H707	Zinc finger protein 552	ZNF552			
Q8IYX0	Zinc finger protein 679	ZNF679			

O14863	Zinc transporter 4	SLC30A4			
Q96JF6	Zinc finger protein 594	ZNF594			
O43670	BUB3-interacting and GLEBS motif-containing protein ZNF207	ZNF207			
Q6ZMS7	Protein ZNF783	ZNF783			
Q9H0M5	Zinc finger protein 700	ZNF700			
Q9NVG8	TBC1 domain family member 13	TBC1D13			
Q9BXJ8	Ion channel TACAN	TMEM120A			
Q9UHQ7	Transcription elongation factor A protein-like 9	TCEAL9			
Q4VX76	Synaptotagmin-like protein 3	SYTL3			
Q96DA6	Mitochondrial import inner membrane translocase subunit TIM14	DNAJC19			
Q8IUX1	Complex I assembly factor TMEM126B, mitochondrial	TMEM126B			
Q9NUJ3	T-complex protein 11-like protein 1	TCP11L1			
Q9H2B2	Synaptotagmin-4	SYT4			
Q9BQS2	Synaptotagmin-15	SYT15			
Q16473	Putative tenascin-XA	TNXA			
P13984	General transcription factor IIF subunit 2	GTF2F2			
Q8TB96	T-cell immunomodulatory protein	ITFG1			
Q9BRJ7	Tudor-interacting repair regulator protein	NUDT16L1			
Q96A09	Terminal nucleotidyltransferase 5B	TENT5B			
Q9Y5L4	Mitochondrial import inner membrane translocase subunit Tim13	TIMM13			
Q8WY91	Peroxynitrite isomerase THAP4	THAP4			
Q8NHR7	Telomere repeats-binding bouquet formation protein 2	TERB2			
Q8WUY1	Protein THEM6	THEM6			
A6NFA1	Metalloprotease TIKI2	TRABD2B			
Q3LXA3	Triokinase/FMN cyclase	TKFC			
Q9H0I9	Transketolase-like protein 2	TKTL2			
O15482	Testis-specific protein TEX28	TEX28			
Q8IZJ6	Inactive L-threonine 3-dehydrogenase, mitochondrial	TDH			
Q96B21	Transmembrane protein 45B	TMEM45B			
Q8N511	Transmembrane protein 199	TMEM199			
Q8TC26	Transmembrane protein 163	TMEM163			
O15321	Transmembrane 9 superfamily member 1	TM9SF1			
P08582	Melanotransferrin	MELTF			
Q9UPQ4	E3 ubiquitin-protein ligase	TRIM35			
Q9NZR1	Tropomodulin-2	TMOD2			
Q93096	Protein tyrosine phosphatase type IVA 1	PTP4A1			
P28289	Tropomodulin-1	TMOD1			
Q8IWR1	Tripartite motif-containing protein 59	TRIM59			
P0DKB5	Trophoblast glycoprotein-like	TPBGL			
Q86WT1	Tetratricopeptide repeat protein 30A	TTC30A			
Q13114	TNF receptor-associated factor 3	TRAF3			
Q8NG68	Tubulin--tyrosine ligase	TTL			
Q5VYS8	Terminal uridylyltransferase 7	TUT7			
A0A0B4J27	T cell receptor alpha variable 21	TRAV21			
Q86TV6	Tetratricopeptide repeat protein 7B	TTC7B			
Q92623	Tetratricopeptide repeat protein 9A	TTC9			
Q96N46	Tetratricopeptide repeat protein 14	TTC14			
Q9HCF6	Transient receptor potential cation channel subfamily M member 3	TRPM3			

A0A0K0K1G6	T cell receptor beta variable 10-3	TRBV10-3			
P35544	Ubiquitin-like protein FUBI	FAU			
Q9HAW7	UDP-glucuronosyltransferase 1A7	UGT1A7			
Q92995	Ubiquitin carboxyl-terminal hydrolase 13	USP13			
Q13404	Ubiquitin-conjugating enzyme E2 variant 1	UBE2V1			
Q9Y385	Ubiquitin-conjugating enzyme E2 J1	UBE2J1			
O43314	Inositol hexakisphosphate and diphosphoinositol-pentakisphosphate kinase 2	PPIP5K2			
Q8NDX2	Vesicular glutamate transporter 3	SLC17A8			
Q96RL7	Vacuolar protein sorting-associated protein 13A	VPS13A			
Q8TCY9	Up-regulator of cell proliferation	URGCP			
Q6P4I2	WD repeat-containing protein 73	WDR73			
A2RUG3	Testis-specific XK-related protein, Y-linked 2	XKRY2			
Q9HAW9	UDP-glucuronosyltransferase 1A8	UGT1A8			
Q9BQY6	WAP four-disulfide core domain protein 6	WFDC6			
Q8IUB5	WAP four-disulfide core domain protein 13	WFDC13			
Q9H269	Vacuolar protein sorting-associated protein 16 homolog	VPS16			
A6NCI4	von Willebrand factor A domain-containing protein 3A	VWA3A			
Q8N2E2	von Willebrand factor D and EGF domain-containing protein	VWDE			
Q86Y38	Xylosyltransferase 1	XYLT1			
Q6VEQ5	WAS protein family homolog 2	WASH2P			
Q8NA23	WD repeat-containing protein 31	WDR31			
Q9H9H4	Vacuolar protein sorting-associated protein 37B	VPS37B			
Q6UXR6	Putative uncharacterized protein UNQ6494/PRO21346	UNQ6494/PRO21346			
Q9NZV7	Zinc finger imprinted 2	ZIM2			
Q3SY52	Zinc finger protein interacting with ribonucleoprotein K	ZIK1			
Q96QA6	Protein yippee-like 2	YPEL2			
P37275	Zinc finger E-box-binding homeobox 1	ZEB1			
Q9H8U3	AN1-type zinc finger protein 3	ZFAND3			
Q6PG37	Zinc finger protein 790	ZNF790			
P51504	Zinc finger protein 80	ZNF80			
Q8N1W2	Zinc finger protein 710	ZNF710			
Q9H5J0	Zinc finger and BTB domain-containing protein 3	ZBTB3			
Q68DI1	Zinc finger protein 776	ZNF776			
Q96QT6	PHD finger protein 12	PHF12			
Q8NDH3	Probable aminopeptidase NPEPL1	NPEPL1			
P22061	Protein-L-isoaspartate	PCMT1			
Q7RTV0	PHD finger-like domain-containing protein 5A	PHF5A			
P36955	Pigment epithelium-derived factor	SERPINF1			
Q53FA7	Quinone oxidoreductase PIG3	TP53I3			
P39019	40S ribosomal protein S19	RPS19			
Q9BWG4	Single-stranded DNA-binding protein 4	SSBP4			
Q9NQ55	Suppressor of SWI4 1 homolog	PPAN			
O95793	Double-stranded RNA-binding protein Staufen homolog 1	STAU1			
Q8NHG7	Small VCP/p97-interacting protein	SVIP			
P0CW01	Testis-specific Y-encoded protein 10	TSPY10			

Q9BRT2	Ubiquinol-cytochrome-c reductase complex assembly factor 2	UQCC2			
P31391	Somatostatin receptor type 4	SSTR4			
Q9HBF5	Suppressor of tumorigenicity 20 protein	ST20			
Q9BX66	Sorbin and SH3 domain-containing protein 1	SORBS1			
O43463	Histone-lysine N-methyltransferase SUV39H1	SUV39H1			
Q9NX61	Transmembrane protein 161A	TMEM161A			
Q6URK8	Testis, prostate and placenta-expressed protein	TEPP			
Q9Y2C9	Toll-like receptor 6	TLR6			
Q9Y6I9	Testis-expressed protein 264	TEX264			
Q9BZJ3	Tryptase delta	TPSD1			
Q16763	Ubiquitin-conjugating enzyme E2 S	UBE2S			
Q13564	NEDD8-activating enzyme E1 regulatory subunit	NAE1			
Q15386	Ubiquitin-protein ligase E3C	UBE3C			
P36537	UDP-glucuronosyltransferase	UGT2B10			
Q8TEY7	Ubiquitin carboxyl-terminal hydrolase 33	USP33			
D6RA61	Ubiquitin carboxyl-terminal hydrolase 17-like protein 22	USP17L22			
Q9Y5J1	U3 small nucleolar RNA-associated protein 18 homolog	UTP18			
Q8N8Y2	V-type proton ATPase subunit d 2	ATP6V0D2			
Q6BDS2	UHRF1-binding protein 1	UHRF1BP1			
Q6RFH5	WD repeat-containing protein 74	WDR74			
Q93050	V-type proton ATPase 116 kDa subunit a1	ATP6V0A1			
Q86XK7	V-set and immunoglobulin domain-containing protein 1	VSIG1			
B1ANS9	WD repeat-containing protein 64	WDR64			
Q8TAA9	Vang-like protein 1	VANGL1			
Q9Y279	V-set and immunoglobulin domain-containing protein 4	VSIG4			
Q9BZH6	WD repeat-containing protein 11	WDR11			
Q9H1B5	Xylosyltransferase 2	XYLT2			
Q9Y3C0	WASH complex subunit 3	WASHC3			
Q9Y6W5	Wiskott-Aldrich syndrome protein family member 2	WASF2			
Q6AWC2	Protein WWC2	WWC2			
Q6XCG6	Putative uncharacterized protein PP632	PP632			
Q8N2B8	Putative uncharacterized protein FLJ33534				
Q8N1Y9	Putative uncharacterized protein FLJ37218				
Q9UI72	Putative uncharacterized protein PRO0255	PRO0255			
Q5VUA4	Zinc finger protein 318	ZNF318			
Q9ULJ3	Zinc finger and BTB domain-containing protein 21	ZBTB21			
Q7Z3T8	Zinc finger FYVE domain-containing protein 16	ZFYVE16			
C9JN71	Zinc finger protein 878	ZNF878			
Q9UEG4	Zinc finger protein 629	ZNF629			
Q96C28	Zinc finger protein 707	ZNF707			
Q8TF45	Zinc finger protein 418	ZNF418			
Q2M1K9	Zinc finger protein 423	ZNF423			
Q8TCN5	Zinc finger protein 507	ZNF507			
Q8TF32	Zinc finger protein 431	ZNF431			
Q9Y2H8	Zinc finger protein 510	ZNF510			
Q8IVP9	Zinc finger protein 547	ZNF547			
Q9UL36	Zinc finger protein 236	ZNF236			

Q9BV97	KRAB domain-containing protein ZNF747	ZNF747			
Q2M3X9	Zinc finger protein 674	ZNF674			
Q92750	Transcription initiation factor TFIID subunit 4B	TAF4B			
P21731	Thromboxane A2 receptor	TBXA2R			
Q9NYW6	Taste receptor type 2 member 3	TAS2R3			
Q7Z6L1	Tectonin beta-propeller repeat-containing protein 1	TECPR1			
Q8TDW5	Synaptotagmin-like protein 5	SYTL5			
Q13263	Transcription intermediary factor 1-beta	TRIM28			
Q9BWD1	Acetyl-CoA acetyltransferase, cytosolic	ACAT2			
Q6ZMR5	Transmembrane protease serine 11A	TMPRSS11 A			
O14925	Mitochondrial import inner membrane translocase subunit Tim23	TIMM23			
P09110	3-ketoacyl-CoA thiolase, peroxisomal	ACAA1			
Q8TBR7	TLC domain-containing protein	TLCD3A			
Q8N6K0	Testis-expressed protein 29	TEX29			
Q9UHF0	Tachykinin-3	TAC3			
Q08117	TLE family member 5	TLE5			
O60522	Tudor domain-containing protein 6	TDRD6			
P42680	Tyrosine-protein kinase Tec	TEC			
Q5VVB8	Transmembrane protein 244	TMEM244			
A6NLX4	Transmembrane protein 210	TMEM210			
Q8NCS4	Transmembrane protein 35B	TMEM35B			
Q9NSU2	Three-prime repair exonuclease 1	TREX1			
Q12815	Tastin	TROAP			
O00635	E3 ubiquitin-protein ligase	TRIM38			
O95361	Tripartite motif-containing protein 16	TRIM16			
Q8IWZ4	Tripartite motif-containing protein 48	TRIM48			
Q8WV44	E3 ubiquitin-protein ligase	TRIM41			
O15405	TOX high mobility group box family member 3	TOX3			
A0JD36	T cell receptor delta variable 2	TRDV2			
O43280	Trehalase	TREH			
Q8NER1	Transient receptor potential cation channel subfamily V member 1	TRPV1			
A0A0A0MS 01	Probable non-functional T cell receptor gamma variable 10	TRGV10			
A0A0K0K1B 3	T cell receptor beta variable 30	TRBV30			
Q15661	Tryptase alpha/beta-1	TPSAB1			
P61960	Ubiquitin-fold modifier 1	UFM1			
O94966	Ubiquitin carboxyl-terminal hydrolase 19	USP19			
Q15695	Putative U2 small nuclear ribonucleoprotein auxiliary factor 35 kDa subunit-related protein 1	ZRSR2P1			
Q495M9	Usher syndrome type-1G protein	USH1G			
Q9P2Y5	UV radiation resistance-associated gene protein	UVRAG			
Q96MX6	WD repeat-containing protein 92	WDR92			
P63129	Endogenous retrovirus group K member 24 Pro protein	ERVK-24			
Q92738	USP6 N-terminal-like protein	USP6NL			
Q8TF30	WASP homolog-associated protein with actin, membranes and microtubules	WHAMM			
A3KMH1	von Willebrand factor A domain-containing protein 8	VWA8			
Q8TBZ3	WD repeat-containing protein 20	WDR20			

O94967	WD repeat-containing protein 47	WDR47			
Q9H0M0	NEDD4-like E3 ubiquitin-protein ligase WWP1	WWP1			
Q06250	Putative Wilms tumor upstream neighbor 1 gene protein	WT1-AS			
Q6ICG8	Postacrosomal sheath WW domain-binding protein	WBP2NL			
O95785	Protein Wiz	WIZ			
Q3C1V9	Putative uncharacterized protein ENSP00000334305				
Q6ZS52	Putative uncharacterized protein FLJ45825				
Q9UFV3	Putative uncharacterized protein DKFZp434L187				
P98169	Zinc finger X-linked protein ZXDB	ZXDB			
Q6UXR8	Putative uncharacterized protein UNQ6493/PRO21345	UNQ6493/ PRO21345			
Q6P2D0	Zinc finger protein 1 homolog	ZFP1			
Q9C0A1	Zinc finger homeobox protein 2	ZFHX2			
P17026	Zinc finger protein 22	ZNF22			
Q8NAP3	Zinc finger and BTB domain-containing protein 38	ZBTB38			
Q9Y2P0	Zinc finger protein 835	ZNF835			
Q86YH2	Zinc finger protein 280B	ZNF280B			
Q9BQ24	Zinc finger FYVE domain-containing protein 21	ZFYVE21			
Q6N043	Zinc finger protein 280D	ZNF280D			
Q96PQ6	Zinc finger protein 317	ZNF317			
P52740	Zinc finger protein 132	ZNF132			
Q5T7W0	Zinc finger protein 618	ZNF618			
Q969S3	Zinc finger protein 622	ZNF622			
O15062	Zinc finger and BTB domain-containing protein 5	ZBTB5			
Q8N9K5	Zinc finger protein 565	ZNF565			
Q5D1E8	Endoribonuclease ZC3H12A	ZC3H12A			
Q9HAH1	Zinc finger protein 556	ZNF556			
Q5EBM4	Putative zinc finger protein 542	ZNF542P			
Q08ER8	Zinc finger protein 543	ZNF543			
Q9C0B5	Palmitoyltransferase ZDHHC5	ZDHHC5			
Q86SH2	Zygote arrest protein 1	ZAR1			
P01210	Proenkephalin-A	PENK			
Q8WUM4	Programmed cell death 6-interacting protein	PDCD6IP			
Q86UU1	Pleckstrin homology-like domain family B member 1	PHLDB1			
Q8N4E4	Phosducin-like protein 2	PDCL2			
Q13113	PDZK1-interacting protein 1	PDZK1IP1			
Q5K4E3	Polyserase-2	PRSS36			
P63135	Endogenous retrovirus group K member 7 Pol protein	ERVK-7			
A4D1T9	Probable inactive serine protease 37	PRSS37			
Q8N3Z0	Inactive serine protease 35	PRSS35			
P01236	Prolactin	PRL			
P20472	Parvalbumin alpha	PVALB			
P49643	DNA primase large subunit	PRIM2			
Q7Z6L0	Proline-rich transmembrane protein 2	PRRT2			
P43686	26S proteasome regulatory subunit 6B	PSMC4			
Q7Z3Z3	Piwi-like protein 3	PIWIL3			
Q8NHR9	Profilin-4	PFN4			
Q9NZ81	Proline-rich protein 13	PRR13			
Q9NZH5	Securin-2	PTTG2			
Q05066	Sex-determining region Y protein	SRY			
Q8IZP1	TBC1 domain family member 3	TBC1D3			
Q96CE8	Transmembrane 4 L6 family member 18	TM4SF18			

Q15573	TATA box-binding protein-associated factor RNA polymerase I subunit A	TAF1A			
A6NDS4	TBC1 domain family member 3B	TBC1D3B			
Q9UMR3	T-box transcription factor TBX20	TBX20			
P20061	Transcobalamin-1	TCN1			
P37802	Transgelin-2	TAGLN2			
O75157	TSC22 domain family protein 2	TSC22D2			
Q16594	Transcription initiation factor TFIID subunit 9	TAF9			
O95759	TBC1 domain family member 8	TBC1D8			
Q15560	Transcription elongation factor A protein 2	TCEA2			
A2VDJ0	Transmembrane protein 131-like	TMEM131L			
P48307	Tissue factor pathway inhibitor 2	TFPI2			
Q8IWZ5	Tripartite motif-containing protein 42	TRIM42			
Q6PL24	Protein TMED8	TMED8			
Q6PF05	Tetratricopeptide repeat protein 23-like	TTC23L			
Q96J42	Thioredoxin domain-containing protein 15	TXNDC15			
Q9BSE2	Transmembrane protein 79	TMEM79			
Q9NW97	Transmembrane protein 51	TMEM51			
A0A0A0MS05	Probable non-functional T cell receptor beta variable 5-7	TRBV5-7			
P29597	Non-receptor tyrosine-protein kinase TYK2	TYK2			
Q14CS0	UBX domain-containing protein 2B	UBXN2B			
Q6UXZ4	Netrin receptor UNC5D	UNC5D			
Q8WUN7	Ubiquitin domain-containing protein 2	UBTD2			
Q9UNY4	Transcription termination factor 2	TTF2			
Q9NRR5	Ubiquilin-4	UBQLN4			
Q96RP3	Urocortin-2	UCN2			
Q99437	V-type proton ATPase 21 kDa proteolipid subunit	ATP6V0B			
A4UGR9	Xin actin-binding repeat-containing protein 2	XIRP2			
O95619	YEATS domain-containing protein 4	YEATS4			
Q6ZSN1	Putative uncharacterized protein FLJ45355				
Q68DY1	Zinc finger protein 626	ZNF626			
P51786	Zinc finger protein 157	ZNF157			
Q5TYW1	Zinc finger protein 658	ZNF658			
Q96NJ3	Zinc finger protein 285	ZNF285			
Q8NE65	Protein ZNF738	ZNF738			
Q8NDX6	Zinc finger protein 740	ZNF740			
Q01130	Serine/arginine-rich splicing factor 2	SRSF2			
Q6ZWJ1	Syntaxin-binding protein 4	STXBP4			
Q9HD15	Steroid receptor RNA activator 1	SRA1			
Q9NSD9	Phenylalanine--tRNA ligase beta subunit	FARSB			
Q8TBG9	Synaptoporin	SYNPR			
Q16563	Synaptophysin-like protein 1	SYPL1			
O60907	F-box-like/WD repeat-containing protein TBL1X	TBL1X			
P07437	Tubulin beta chain	TUBB			
Q7Z5A9	Chemokine-like protein TAFA-1	TAFA1			
Q16559	T-cell acute lymphocytic leukemia protein 2	TAL2			
Q13148	TAR DNA-binding protein 43	TARDBP			
Q3YBR2	Transforming growth factor beta regulator 1	TBRG1			
Q7Z422	SUZ domain-containing protein 1	SZRD1			
Q96M29	Tektin-5	TEKT5			

P59541	Taste receptor type 2 member 30	TAS2R30			
Q96J01	THO complex subunit 3	THOC3			
Q9NXF1	Testis-expressed protein 10	TEX10			
Q8WVM0	Dimethyladenosine transferase 1, mitochondrial	TFB1M			
Q9UKI8	Serine/threonine-protein kinase tousled-like 1	TLK1			
Q9H061	Transmembrane protein 126A	TMEM126A			
A0A087WTH1	Transmembrane protein 265	TMEM265			
Q8N7C4	Transmembrane protein 217	TMEM217			
A0A1B0GVZ9	Transmembrane protein 269	TMEM269			
B4DJY2	Transmembrane protein 233	TMEM233			
A6NFC5	Transmembrane protein 235	TMEM235			
Q99594	Transcriptional enhancer factor TEF-5	TEAD3			
A0A075B6R0	T cell receptor gamma variable 2	TRGV2			
P0CF51	T cell receptor gamma constant 1	TRGC1			
Q6ZTW0	Tubulin polyglutamylase complex subunit 1	TPGS1			
Q9C030	Tripartite motif-containing protein 6	TRIM6			
O14798	Tumor necrosis factor receptor superfamily member 10C	TNFRSF10C			
Q14669	E3 ubiquitin-protein ligase TRIP12	TRIP12			
P04437	T cell receptor alpha variable 29/delta variable 5	TRAV29DV5			
O95900	Mitochondrial mRNA pseudouridine synthase TRUB2	TRUB2			
Q9BWV7	Probable tubulin polyglutamylase TTLL2	TTLL2			
A6NJT0	Homeobox protein unc-4 homolog	UNCX			
O95155	Ubiquitin conjugation factor E4 B	UBE4B			
O94782	Ubiquitin carboxyl-terminal hydrolase 1	USP1			
Q9P275	Ubiquitin carboxyl-terminal hydrolase 36	USP36			
O60294	tRNA wybutosine-synthesizing protein 4	LCMT2			
O75385	Serine/threonine-protein kinase ULK1	ULK1			
O95258	Brain mitochondrial carrier protein 1	SLC25A14			
P47901	Vasopressin V1b receptor	AVPR1B			
Q8TCH9	Putative uncharacterized protein FLJ23865				
Q8N7Q3	Zinc finger protein 676	ZNF676			
Q8NDQ6	Zinc finger protein 540	ZNF540			
Q9P255	Zinc finger protein 492	ZNF492			
Q9H3H9	Transcription elongation factor A protein-like 2	TCEAL2			
Q9H2K8	Serine/threonine-protein kinase TAO3	TAOK3			
Q96A56	Tumor protein p53-inducible nuclear protein 1	TP53INP1			
Q969E4	Transcription elongation factor A protein-like 3	TCEAL3			
H3BS89	Transmembrane protein 178B	TMEM178B			
Q9Y458	T-box transcription factor TBX22	TBX22			
Q9P031	Thyroid transcription factor 1-associated protein 26	CCDC59			
Q92545	Transmembrane protein 131	TMEM131			
Q86SS6	Synaptotagmin-9	SYT9			
Q96FV9	THO complex subunit 1	THOC1			
Q96CP7	TLC domain-containing protein 1	TLCD1			

Q8TBB0	THAP domain-containing protein 6	THAP6			
Q8NA69	Testis-expressed protein 45	TEX45			
Q8NFU7	Methylcytosine dioxygenase TET1	TET1			
Q9UGI8	Testin	TES			
A6NML5	Transmembrane protein 212	TMEM212			
Q9P0N5	Transmembrane protein 216	TMEM216			
P49788	Retinoic acid receptor responder protein 1	RARRES1			
Q9NS93	Transmembrane 7 superfamily member 3	TM7SF3			
P03979	T cell receptor gamma variable 3	TRGV3			
Q12816	Trophinin	TRO			
P43489	Tumor necrosis factor receptor superfamily member 4	TNFRSF4			
Q71RG4	Transmembrane and ubiquitin-like domain-containing protein 2	TMUB2			
Q9H8W5	Tripartite motif-containing protein 45	TRIM45			
Q8IU80	Transmembrane protease serine 6	TMPRSS6			
P26651	mRNA decay activator protein ZFP36	ZFP36			
Q96QT4	Transient receptor potential cation channel subfamily M member 7	TRPM7			
P35030	Trypsin-3	PRSS3			
A0A0J9YX7	T cell receptor beta variable 6-9	TRBV6-9			
A6NDI0	Putative tripartite motif-containing protein 49B	TRIM49B			
Q8TF42	Ubiquitin-associated and SH3 domain-containing protein B	UBASH3B			
Q3SY77	UDP-glucuronosyltransferase 3A2	UGT3A2			
Q70CQ3	Ubiquitin carboxyl-terminal hydrolase 30	USP30			
Q5VV11	Putative UPF0633 protein ENSP00000303136				
Q70CQ1	Ubiquitin carboxyl-terminal hydrolase 49	USP49			
Q9H9J4	Ubiquitin carboxyl-terminal hydrolase 42	USP42			
A6NCW0	Ubiquitin carboxyl-terminal hydrolase 17-like protein 3	USP17L3			
Q9H7M9	V-type immunoglobulin domain-containing suppressor of T-cell activation	VSIR			
O14972	Vacuolar protein sorting-associated protein 26C	VPS26C			
P08670	Vimentin	VIM			
P30518	Vasopressin V2 receptor	AVPR2			
Q15849	Urea transporter 2	SLC14A2			
Q6PP77	XK-related protein 2	XKRX			
Q96JC1	Vam6/Vps39-like protein	VPS39			
Q9P253	Vacuolar protein sorting-associated protein 18 homolog	VPS18			
Q8NBI6	Xyloside xylosyltransferase 1	XXYLT1			
Q9BTA9	WW domain-containing adapter protein with coiled-coil	WAC			
Q96P53	WD repeat and FYVE domain-containing protein 2	WDFY2			
A6NGB9	WAS/WASL-interacting protein family member 3	WIPF3			
Q6ZUG5	Uncharacterized protein FLJ43738				
A6NDX4	Putative transmembrane protein ENSP00000320207				
P31629	Transcription factor HIVEP2	HIVEP2			
O76080	AN1-type zinc finger protein 5	ZFAND5			
Q7Z7L7	Protein zer-1 homolog	ZER1			
Q9NPG8	Palmitoyltransferase ZDHHC4	ZDHHC4			
Q8N8J6	Zinc finger protein 615	ZNF615			
Q86TJ5	Zinc finger protein 554	ZNF554			

Q9Y2X9	Zinc finger protein 281	ZNF281			
P17025	Zinc finger protein 182	ZNF182			
O95825	Quinone oxidoreductase-like protein 1	CRYZL1			
Q4LDE5	Sushi, von Willebrand factor type A, EGF and pentraxin domain-containing protein 1	SVEP1			
O60343	TBC1 domain family member 4	TBC1D4			
Q8IXX5	Transmembrane protein 183A	TMEM183A			
Q8N3R3	T-cell activation inhibitor, mitochondrial	TCAIM			
P19532	Transcription factor E3	TFE3			
Q9BXI6	TBC1 domain family member 10A	TBC1D10A			
Q8N4U5	T-complex protein 11-like protein 2	TCP11L2			
P52888	Thimet oligopeptidase	THOP1			
Q07654	Trefoil factor 3	TFF3			
Q8WWH5	Probable tRNA pseudouridine synthase 1	TRUB1			
Q9Y5U2	Protein TSSC4	TSSC4			
Q5T7W7	Thiosulfate sulfurtransferase/rhodanese-like domain-containing protein 2	TSTD2			
O75896	Tumor suppressor candidate 2	TUSC2			
Q9NNW7	Thioredoxin reductase 2, mitochondrial	TXNRD2			
Q9NZQ8	Transient receptor potential cation channel subfamily M member 5	TRPM5			
Q7Z2W7	Transient receptor potential cation channel subfamily M member 8	TRPM8			
C9JPN9	Ubiquitin carboxyl-terminal hydrolase 17-like protein 12	USP17L12			
Q8N2C9	Uncharacterized protein UMODL1-AS1	UMODL1-AS1			
P0DPF7	T cell receptor beta variable 6-3	TRBV6-3			
A0A597	T cell receptor beta variable 5-5	TRBV5-5			
P0CI26	Tripartite motif-containing protein 49C	TRIM49C			
C9J1S8	Tripartite motif-containing protein 49D	TRIM49D1			
A0A0K0K1A5	T cell receptor beta variable 6-5	TRBV6-5			
Q96HA7	Tonsoku-like protein	TONSL			
A6NGE7	Putative 2-oxo-4-hydroxy-4-carboxy-5-ureidoimidazoline decarboxylase	URAD			
P01848	T cell receptor alpha chain constant	TRAC			
Q8NEW7	Transmembrane inner ear expressed protein	TMIE			
Q8IZJ1	Netrin receptor UNC5B	UNC5B			
Q6ZN44	Netrin receptor UNC5A	UNC5A			
F5GYI3	Ubiquitin-associated protein 1-	UBAP1L			
Q9UMX0	Ubiquilin-1	UBQLN1			
P09327	Villin-1	VIL1			
O75445	Usherin	USH2A			
Q9Y2C2	Uronyl 2-sulfotransferase	UST			
P40337	von Hippel-Lindau disease tumor suppressor	VHL			
Q8NHE4	V-type proton ATPase subunit e 2	ATP6V0E2			
Q96A05	V-type proton ATPase subunit E 2	ATP6V1E2			
Q6ZQQ6	WD repeat-containing protein 87	WDR87			
Q8IUB2	WAP four-disulfide core domain protein 3	WFDC3			
Q01831	DNA repair protein complementing XP-C cells	XPC			
O75351	Vacuolar protein sorting-associated protein 4B	VPS4B			
Q13426	DNA repair protein XRCC4	XRCC4			

Q96QU8	Exportin-6	XPO6			
Q5VIR6	Vacuolar protein sorting-associated protein 53 homolog	VPS53			
Q9H0D6	5'-3' exoribonuclease 2	XRN2			
Q502W6	von Willebrand factor A domain-containing protein 3B	VWA3B			
Q9C0J8	pre-mRNA 3' end processing protein WDR33	WDR33			
P61964	WD repeat-containing protein 5	WDR5			
Q9NP79	Vacuolar protein sorting-associated protein VTA1 homolog	VTA1			
Q6ZQT0	Putative uncharacterized protein FLJ45035				
Q8NBF4	Putative uncharacterized protein FLJ33307				
Q9UI25	Putative uncharacterized protein PRO0461	PRO0461			
Q3KQV3	Zinc finger protein 792	ZNF792			
P17010	Zinc finger X-chromosomal protein	ZFX			
P16415	Zinc finger protein 823	ZNF823			
Q8NEG5	E3 ubiquitin-protein ligase ZSWIM2	ZSWIM2			
Q0D2J5	Zinc finger protein 763	ZNF763			
Q8IUH5	Palmitoyltransferase ZDHHC17	ZDHHC17			
Q6ZNG1	Zinc finger protein 600	ZNF600			
Q15697	Zinc finger protein 174	ZNF174			
Q2M218	Zinc finger protein 630	ZNF630			
Q9UNY5	Zinc finger protein 232	ZNF232			
Q5JVG2	Zinc finger protein 484	ZNF484			
A8MTY0	Zinc finger protein 724	ZNF724			
Q8N3J9	Zinc finger protein 664	ZNF664			
Q499Z4	Zinc finger protein 672	ZNF672			
Q9HAI6	TLR adapter interacting with SLC15A4 on the lysosome	TASL			
Q8TC07	TBC1 domain family member 15	TBC1D15			
A0PK00	Transmembrane protein 120B	TMEM120B			
O00445	Synaptotagmin-5	SYT5			
Q3SY00	Testis-specific protein 10-interacting protein	TSGA10IP			
Q8IYQ7	Threonine synthase-like 1	THNSL1			
Q8N8V8	Transmembrane protein 105	TMEM105			
O15455	Toll-like receptor 3	TLR3			
Q5T0J7	Testis-expressed protein 35	TEX35			
Q92563	Testican-2	SPOCK2			
Q8WZ42	Titin	TTN			
Q92994	Transcription factor IIIB 90 kDa subunit	BRF1			
Q8N616	Putative uncharacterized protein encoded by LINC00311	LINC00311			
Q9BX73	TM2 domain-containing protein 2	TM2D2			
Q96A57	Transmembrane protein 230	TMEM230			
Q9BXR0	Queuine tRNA-ribosyltransferase catalytic subunit 1	QTRT1			
Q07283	Trichohyalin	TCHH			
Q86YW5	Trem-like transcript 1 protein	TREML1			
O95150	Tumor necrosis factor ligand superfamily member 15	TNFSF15			
Q9BVS5	tRNA	TRMT61B			
O94811	Tubulin polymerization-promoting protein	TPPP			
O60784	Target of Myb protein 1	TOM1			
Q7Z6W1	Transmembrane and coiled-coil domain-containing protein 2	TMCO2			
Q9NS68	Tumor necrosis factor receptor superfamily member 19	TNFRSF19			
Q86VQ3	Thioredoxin domain-containing protein 2	TXNDC2			

Q9HCX4	Short transient receptor potential channel 7	TRPC7			
Q6IQ55	Tau-tubulin kinase 2	TTBK2			
A0A087WT01	T cell receptor alpha variable 27	TRAV27			
P62079	Tetraspanin-5	TSPAN5			
Q13454	Tumor suppressor candidate 3	TUSC3			
P11172	Uridine 5'-monophosphate synthase	UMPS			
Q96B02	Ubiquitin-conjugating enzyme E2 W	UBE2W			
Q9NYU1	UDP-glucose:glycoprotein glucosyltransferase 2	UGGT2			
O95071	E3 ubiquitin-protein ligase UBR5	UBR5			
Q16864	V-type proton ATPase subunit F	ATP6V1F			
P22415	Upstream stimulatory factor 1	USF1			
P21796	Voltage-dependent anion-selective channel protein 1	VDAC1			
P63127	Endogenous retrovirus group K member 9 Pro protein	ERVK-9			
Q86XT2	Vacuolar protein sorting-associated protein 37D	VPS37D			
Q9Y3S1	Serine/threonine-protein kinase WNK2	WNK2			
Q5VSD8	Putative uncharacterized protein LOC401522				
Q9Y548	Protein YIPF1	YIPF1			
Q6ZSR3	Putative uncharacterized protein FLJ45275, mitochondrial				
Q8N9W7	Putative transmembrane protein FLJ36131				
Q8WZ26	Putative uncharacterized protein PP6455	PP6455			
P0C880	Putative uncharacterized protein FLJ40606				
Q6DD87	Zinc finger protein 787	ZNF787			
Q13105	Zinc finger and BTB domain-containing protein 17	ZBTB17			
Q7Z739	YTH domain-containing family protein 3	YTHDF3			
A6NDX5	Putative zinc finger protein 840	ZNF840P			
Q8N4W9	Zinc finger protein 808	ZNF808			
O60765	Zinc finger protein 354A	ZNF354A			
A2A288	Probable ribonuclease ZC3H12D	ZC3H12D			
Q9Y2K1	Zinc finger and BTB domain-containing protein 1	ZBTB1			
A8MUZ8	Putative zinc finger protein 705G	ZNF705G			
Q03936	Zinc finger protein 92	ZNF92			
Q6P1L6	Zinc finger protein 343	ZNF343			
O60232	Protein ZNRD2	ZNRD2			
Q8TA94	Zinc finger protein 563	ZNF563			
Q12836	Zona pellucida sperm-binding protein 4	ZP4			
Q9UL59	Zinc finger protein 214	ZNF214			
Q8NDW4	Zinc finger protein 248	ZNF248			
Q9UHR6	Zinc finger HIT domain-containing protein 2	ZNHIT2			
Q5HYK9	Zinc finger protein 667	ZNF667			
Q5SXM1	Zinc finger protein 678	ZNF678			
Q9H2Y7	Zinc finger protein 106	ZNF106			
Q6UX98	Probable palmitoyltransferase ZDHHC24	ZDHHC24			
Q6ZNC4	Zinc finger protein 704	ZNF704			
Q7LBC6	Lysine-specific demethylase 3B	KDM3B			
Q9BXK1	Krueppel-like factor 16	KLF16			
A0A075B6P5	Immunoglobulin kappa variable 2-28	IGKV2-28			
O94819	Kelch repeat and BTB domain-containing protein 11	KBTBD11			

P01594	Immunoglobulin kappa variable 1-33	IGKV1-33			
Q6P1Q9	tRNA N	METTL2B			
Q9UJ68	Mitochondrial peptide methionine sulfoxide reductase	MSRA			
Q9ULB1	Neurexin-1	NRXN1			
Q8NGH3	Olfactory receptor 2D3	OR2D3			
Q96R84	Putative olfactory receptor 1F2	OR1F2P			
P08559	Pyruvate dehydrogenase E1 component subunit alpha, somatic form, mitochondrial	PDHA1			
Q6IE36	Ovostatin homolog 2	OVOS2			
Q8NH03	Olfactory receptor 2T3	OR2T3			
Q9H857	5'-nucleotidase domain-containing protein 2	NT5DC2			
Q8IYB4	PEX5-related protein	PEX5L			
Q8NCN5	Pyruvate dehydrogenase phosphatase regulatory subunit, mitochondrial	PDPR			
Q16342	Programmed cell death protein 2	PDCD2			
P36871	Phosphoglucomutase-1	PGM1			
Q86SQ0	Pleckstrin homology-like domain family B member 2	PHLDB2			
P56645	Period circadian protein homolog 3	PER3			
Q96JS3	PiggyBac transposable element-derived protein 1	PGBD1			
Q04941	Proteolipid protein 2	PLP2			
Q08752	Peptidyl-prolyl cis-trans isomerase D	PPID			
P01213	Proenkephalin-B	PDYN			
Q9GZU2	Paternally-expressed gene 3 protein	PEG3			
A6NEE1	Pleckstrin homology domain-containing family D member 1	PLEKHD1			
P53350	Serine/threonine-protein kinase PLK1	PLK1			
Q6UQ28	Placenta-expressed transcript 1 protein	PLET1			
Q9UIG4	Psoriasis susceptibility 1 candidate gene 2 protein	PSORS1C2			
A6NJB7	Proline-rich protein 19	PRR19			
Q9UQK1	Protein phosphatase 1 regulatory subunit 3C	PPP1R3C			
Q5VXH4	PRAME family member 6	PRAMEF6			
Q7Z5V6	Protein phosphatase 1 regulatory subunit 32	PPP1R32			
Q13029	PR domain zinc finger protein 2	PRDM2			
Q9UQ72	Pregnancy-specific beta-1-glycoprotein 11	PSG11			
Q99969	Retinoic acid receptor responder protein 2	RARRES2			
P55042	GTP-binding protein RAD	RRAD			
P54727	UV excision repair protein RAD23 homolog B	RAD23B			
P52756	RNA-binding protein 5	RBM5			
Q8IXT5	RNA-binding protein 12B	RBM12B			
Q12829	Ras-related protein Rab-40B	RAB40B			
O75771	DNA repair protein RAD51 homolog 4	RAD51D			
P63000	Ras-related C3 botulinum toxin substrate 1	RAC1			
O95980	Reversion-inducing cysteine-rich protein with Kazal motifs	RECK			
Q9ULW5	Ras-related protein Rab-26	RAB26			
Q9UJ41	Rab5 GDP/GTP exchange factor	RABGEF1			
Q9H0N0	Ras-related protein Rab-6C	RAB6C			

Q6ZWK4	Regulator of hemoglobinization and erythroid cell expansion protein	RHEX			
Q15493	Regucalcin	RGN			
A6NK89	Ras association domain-containing protein 10	RASSF10			
Q9Y644	Beta-1,3-N-acetylglucosaminyltransferase radical fringe	RFNG			
P84095	Rho-related GTP-binding protein RhoG	RHOG			
Q8TCX5	Rhophilin-1	RHPN1			
O15258	Protein RER1	RER1			
O75787	Renin receptor	ATP6AP2			
P40938	Replication factor C subunit 3	RFC3			
P13489	Ribonuclease inhibitor	RNH1			
P18077	60S ribosomal protein L35a	RPL35A			
Q9NQ50	39S ribosomal protein L40, mitochondrial	MRPL40			
Q8IZ83	Aldehyde dehydrogenase family 16 member A1	ALDH16A1			
O94823	Phospholipid-transporting ATPase VB	ATP10B			
Q8IXM2	Chromatin complexes subunit BAP18	BAP18			
Q6UWJ8	CD164 sialomucin-like 2 protein	CD164L2			
P27824	Calnexin	CANX			
Q9NY47	Voltage-dependent calcium channel subunit alpha-2/delta-2	CACNA2D2			
P07711	Procathepsin L	CTSL			
Q6ZRK6	Coiled-coil domain-containing protein 73	CCDC73			
Q13166	CATR tumorigenic conversion 1 protein	CATR1			
Q8WTZ4	Putative inactive carbonic anhydrase 5B-like protein	CA5BP1			
A0A1B0GTC6	Uncharacterized protein C3orf85	C3orf85			
P01258	Calcitonin	CALCA			
Q9BXT2	Voltage-dependent calcium channel gamma-6 subunit	CACNG6			
Q96M83	Coiled-coil domain-containing protein 7	CCDC7			
Q99616	C-C motif chemokine 13	CCL13			
P53634	Dipeptidyl peptidase 1	CTSC			
O95503	Chromobox protein homolog 6	CBX6			
Q9NZZ3	Charged multivesicular body protein 5	CHMP5			
Q14011	Cold-inducible RNA-binding protein	CIRBP			
A6NNL5	Uncharacterized protein C15orf61	C15orf61			
Q96MX0	CKLF-like MARVEL transmembrane domain-containing protein 3	CMTM3			
P09543	2',3'-cyclic-nucleotide 3'-phosphodiesterase	CNP			
Q8N1N0	C-type lectin domain family 4 member F	CLEC4F			
Q92478	C-type lectin domain family 2 member B	CLEC2B			
P0C0L4	Complement C4-A	C4A			
O75153	Clustered mitochondria protein homolog	CLUH			
Q17RY0	Cytoplasmic polyadenylation element-binding protein 4	CPEB4			
Q96H96	4-hydroxybenzoate polyprenyltransferase, mitochondrial	COQ2			

Q8N123	CPX chromosomal region candidate gene 1 protein	CPXCR1			
Q13324	Corticotropin-releasing factor receptor 2	CRHR2			
P11844	Gamma-crystallin A	CRYGA			
Q96FQ7	Putative uncharacterized protein encoded by LINC00526	LINC00526			
Q8N8Q8	Cytochrome c oxidase assembly protein COX18, mitochondrial	COX18			
A6NLW8	Double homeobox protein A	DUXA			
P17813	Endoglin	ENG			
Q9GZR5	Elongation of very long chain fatty acids protein 4	ELOVL4			
Q32P44	Echinoderm microtubule-associated protein-like 3	EML3			
O95834	Echinoderm microtubule-associated protein-like 2	EML2			
Q17RC7	Exocyst complex component 3-like protein 4	EXOC3L4			
Q5XUX1	F-box/WD repeat-containing protein 9	FBXW9			
Q9H6D8	Fibronectin type III domain-containing protein 4	FNDC4			
Q96AC1	Fermitin family homolog 2	FERMT2			
P49356	Protein farnesyltransferase subunit beta	FNTB			
Q6DN72	Fc receptor-like protein 6	FCRL6			
Q9HAH7	Probable fibrosin-1	FBRS			
Q9NZU0	Leucine-rich repeat transmembrane protein FLRT3	FLRT3			
Q9Y297	F-box/WD repeat-containing protein 1A	BTRC			
Q9UH90	F-box only protein 40	FBXO40			
Q9Y3I1	F-box only protein 7	FBXO7			
Q8N878	FERM domain-containing protein 1	FRMD1			
Q5CZC0	Fibrous sheath-interacting protein 2	FSIP2			
Q5JV73	FERM and PDZ domain-containing protein 3	FRMPD3			
Q96PS6	Putative uncharacterized protein GAFA-1	GAFA1			
Q99958	Forkhead box protein C2	FOXC2			
O43524	Forkhead box protein O3	FOXO3			
Q7Z7M9	Polypeptide N-acetylgalactosaminyltransferase 5	GALNT5			
P15328	Folate receptor alpha	FOLR1			
Q86XP6	Gastrokine-2	GKN2			
Q8N7Z2	Golgin subfamily A member 6-like protein 1	GOLGA6L1			
P28476	Gamma-aminobutyric acid receptor subunit rho-2	GABRR2			
Q9UBI6	Guanine nucleotide-binding protein G	GNG12			
P52951	Homeobox protein GBX-2	GBX2			
P28472	Gamma-aminobutyric acid receptor subunit beta-3	GABRB3			
P30968	Gonadotropin-releasing hormone receptor	GNRHR			
P49863	Granzyme K	GZMK			
A6NK44	Glyoxalase domain-containing protein 5	GLOD5			
Q9BXV9	EKC/KEOPS complex subunit GON7	GON7			
Q9HC38	Glyoxalase domain-containing protein 4	GLOD4			
A8MQT2	Golgin subfamily A member 8B	GOLGA8B			
Q9UJ42	Probable G-protein coupled receptor 160	GPR160			

Q6P9A2	Polypeptide N-acetylgalactosaminyltransferase 18	GALNT18			
P23416	Glycine receptor subunit alpha-2	GLRA2			
Q8TDB8	Solute carrier family 2, facilitated glucose transporter member 14	SLC2A14			
Q9Y2T6	G-protein coupled receptor 55	GPR55			
O95800	Probable G-protein coupled receptor 75	GPR75			
P11168	Solute carrier family 2, facilitated glucose transporter member 2	SLC2A2			
Q53T59	HCLS1-binding protein 3	HS1BP3			
Q9BTM1	Histone H2A.J	H2AJ			
O14756	17-beta-hydroxysteroid dehydrogenase type 6	HSD17B6			
Q6DN03	Putative histone H2B type 2-C	H2BC20P			
P07686	Beta-hexosaminidase subunit beta	HEXB			
A2RTY3	Protein HEATR9	HEATR9			
Q6AI08	HEAT repeat-containing protein 6	HEATR6			
P31271	Homeobox protein Hox-A13	HOXA13			
P42357	Histidine ammonia-lyase	HAL			
P83105	Serine protease HTRA4	HTRA4			
Q9P2P5	E3 ubiquitin-protein ligase	HECW2			
Q7Z6Z7	E3 ubiquitin-protein ligase	HUWE1			
O60506	Heterogeneous nuclear ribonucleoprotein Q	SYNCRIP			
P01782	Immunoglobulin heavy variable 3-9	IGHV3-9			
P09429	High mobility group protein B1	HMGB1			
S4R3Y5	Humanin-like 11	MTRNR2L11			
Q13099	Intraflagellar transport protein 88 homolog	IFT88			
Q99665	Interleukin-12 receptor subunit beta-2	IL12RB2			
O95976	Immunoglobulin superfamily member 6	IGSF6			
Q9NX62	Golgi-resident adenosine 3',5'-bisphosphate 3'-phosphatase	BPNT2			
Q6ZQW0	Indoleamine 2,3-dioxygenase 2	IDO2			
A6NMX2	Eukaryotic translation initiation factor 4E type 1B	EIF4E1B			
Q8WX77	Insulin-like growth factor-binding protein-like 1	IGFBPL1			
Q4G0P3	Hydrocephalus-inducing protein homolog	HYDIN			
Q9H1K1	Iron-sulfur cluster assembly enzyme ISCU, mitochondrial	ISCU			
Q5VV43	Dyslexia-associated protein KIAA0319	KIAA0319			
P27987	Inositol-trisphosphate 3-kinase B	ITPKB			
Q9UHH9	Inositol hexakisphosphate kinase 2	IP6K2			
P27144	Adenylate kinase 4, mitochondrial	AK4			
Q12912	Inositol 1,4,5-triphosphate receptor associated 2	IRAG2			
P05787	Keratin, type II cytoskeletal 8	KRT8			
P00568	Adenylate kinase isoenzyme 1	AK1			
Q8N8D9	Uncharacterized protein IRF1-AS1	IRF1-AS1			
P05154	Plasma serine protease inhibitor	SERPINA5			
Q96JJ6	Junctophilin-4	JPH4			
O14654	Insulin receptor substrate 4	IRS4			
Q9Y496	Kinesin-like protein KIF3A	KIF3A			
Q12918	Killer cell lectin-like receptor subfamily B member 1	KLRB1			
O75582	Ribosomal protein S6 kinase alpha-5	RPS6KA5			
Q8IYT4	Katanin p60 ATPase-containing subunit A-like 2	KATNAL2			
Q16566	Calcium/calmodulin-dependent protein kinase type IV	CAMK4			

P01614	Immunoglobulin kappa variable 2D-40	IGKV2D-40			
Q13064	Probable E3 ubiquitin-protein ligase makorin-3	MKRN3			
A0A0C5B5G6	Mitochondrial-derived peptide MOTS-c	MT-RNR1			
Q8NCE2	Myotubularin-related protein 14	MTMR14			
P13995	Bifunctional methylenetetrahydrofolate dehydrogenase/cyclohydrolase, mitochondrial	MTHFD2			
O14777	Kinetochore protein NDC80 homolog	NDC80			
Q96M43	Neuroblastoma breakpoint family member 4	NBPF4			
E9PAV3	Nascent polypeptide-associated complex subunit alpha, muscle-specific form	NACA			
Q7Z401	C-myc promoter-binding protein	DENND4A			
Q6P582	Mitotic-spindle organizing protein 2A	MZT2A			
O75113	NEDD4-binding protein 1	N4BP1			
Q14CX7	N-alpha-acetyltransferase 25, NatB auxiliary subunit	NAA25			
Q93015	N-alpha-acetyltransferase 80	NAA80			
A6PVI3	Nuclear cap-binding protein subunit 2-like	NCBP2L			
O76036	Natural cytotoxicity triggering receptor 1	NCR1			
P62166	Neuronal calcium sensor 1	NCS1			
P29371	Neuromedin-K receptor	TACR3			
P26715	NKG2-A/NKG2-B type II integral membrane protein	KLRC1			
Q9C000	NACHT, LRR and PYD domains-containing protein 1	NLRP1			
Q9BVI4	Nucleolar complex protein 4 homolog	NOC4L			
Q9NPH5	NADPH oxidase 4	NOX4			
Q9H2A3	Neurogenin-2	NEUROG2			
P55209	Nucleosome assembly protein 1-like 1	NAP1L1			
P15559	NAD	NQO1			
Q8IUK5	Plexin domain-containing protein 1	PLXDC1			
Q6P4R8	Nuclear factor related to kappa-B-binding protein	NFRKB			
P49116	Nuclear receptor subfamily 2 group C member 2	NR2C2			
Q96RI1	Bile acid receptor	NR1H4			
Q6P988	Palmitoleoyl-protein carboxylesterase NOTUM	NOTUM			
P06748	Nucleophosmin	NPM1			
O94916	Nuclear factor of activated T-cells 5	NFAT5			
O95631	Netrin-1	NTN1			
Q16621	Transcription factor NF-E2 45 kDa subunit	NFE2			
Q9H3P2	Negative elongation factor A	NELFA			
Q8IXM6	Nurim	NRM			
Q6ZVK8	8-oxo-dGDP phosphatase NUDT18	NUDT18			
Q14973	Sodium/bile acid cotransporter	SLC10A1			
Q9NZJ9	Diphosphoinositol polyphosphate phosphohydrolase 2	NUDT4			
Q9BW91	ADP-ribose pyrophosphatase, mitochondrial	NUDT9			
Q96R69	Olfactory receptor 4F4	OR4F4			
P00973	2'-5'-oligoadenylate synthase 1	OAS1			
P58170	Olfactory receptor 1D5	OR1D5			

P37287	Phosphatidylinositol N-acetylglucosaminyltransferase subunit A	PIGA			
P27986	Phosphatidylinositol 3-kinase regulatory subunit alpha	PIK3R1			
P32322	Pyrroline-5-carboxylate reductase 1, mitochondrial	PYCR1			
Q7Z442	Polycystic kidney disease protein 1-like 2	PKD1L2			
P25786	Proteasome subunit alpha type-1	PSMA1			
O00562	Membrane-associated phosphatidylinositol transfer protein 1	PITPNM1			
Q7Z3Z4	Piwi-like protein 4	PIWIL4			
O14684	Prostaglandin E synthase	PTGES			
Q96PZ0	Pseudouridylate synthase 7 homolog	PUS7			
P35241	Radixin	RDX			
Q00765	Receptor expression-enhancing protein 5	REEP5			
O94762	ATP-dependent DNA helicase Q5	RECQL5			
O60895	Receptor activity-modifying protein 2	RAMP2			
Q15311	RalA-binding protein 1	RALBP1			
P35243	Recoverin	RCVRN			
Q15276	Rab GTPase-binding effector protein 1	RABEP1			
P51153	Ras-related protein Rab-13	RAB13			
P57735	Ras-related protein Rab-25	RAB25			
P20338	Ras-related protein Rab-4A	RAB4A			
Q14CB8	Rho GTPase-activating protein 19	ARHGAP19			
Q5T8P6	RNA-binding protein 26	RBM26			
Q6UXK2	Immunoglobulin superfamily containing leucine-rich repeat protein 2	ISLR2			
P61925	cAMP-dependent protein kinase inhibitor alpha	PKIA			
Q969R8	KICSTOR complex protein ITFG2	ITFG2			
Q9BUE6	Iron-sulfur cluster assembly 1 homolog, mitochondrial	ISCA1			
Q9Y6Z4	Putative uncharacterized protein KIF25-AS1	KIF25-AS1			
Q3LI76	Keratin-associated protein 15-1	KRTAP15-1			
Q6L8G4	Keratin-associated protein 5-11	KRTAP5-11			
Q8TDN2	Potassium voltage-gated channel subfamily V member 2	KCNV2			
P17658	Potassium voltage-gated channel subfamily A member 6	KCNA6			
O43896	Kinesin-like protein KIF1C	KIF1C			
Q3LHN1	Keratin-associated protein 21-3	KRTAP21-3			
Q9BYP9	Keratin-associated protein 9-9	KRTAP9-9			
P07942	Laminin subunit beta-1	LAMB1			
A0A0C4DH72	Immunoglobulin kappa variable 1-6	IGKV1-6			
O00292	Left-right determination factor 2	LEFTY2			
A6NM62	Leucine-rich repeat-containing protein 53	LRRC53			
Q8N386	Leucine-rich repeat-containing protein 25	LRRC25			
Q96NW7	Leucine-rich repeat-containing protein 7	LRRC7			
Q9P244	Leucine-rich repeat and fibronectin type III domain-containing protein 1	LRFN1			
Q13449	Limbic system-associated membrane protein	LSAMP			
Q9BX40	Protein LSM14 homolog B	LSM14B			
O75342	Arachidonate 12-lipoxygenase, 12R-type	ALOX12B			

Q9H089	Large subunit GTPase 1 homolog	LSG1			
Q3MHD2	Protein LSM12 homolog	LSM12			
Q6VMQ6	Activating transcription factor 7-interacting protein 1	ATF7IP			
Q86V48	Leucine zipper protein 1	LUZP1			
A0A087WSX0	Immunoglobulin lambda variable 5-45	IGLV5-45			
A0A075B6I7	Probable non-functional immunoglobulin lambda variable 5-48	IGLV5-48			
Q86YW9	Mediator of RNA polymerase II transcription subunit 12-like protein	MED12L			
Q13257	Mitotic spindle assembly checkpoint protein MAD2A	MAD2L1			
O43318	Mitogen-activated protein kinase kinase kinase 7	MAP3K7			
Q9Y4F3	Meiosis regulator and mRNA stability factor 1	MARF1			
Q86UL8	Membrane-associated guanylate kinase, WW and PDZ domain-containing protein 2	MAGI2			
P25205	DNA replication licensing factor MCM3	MCM3			
A6NNE9	E3 ubiquitin-protein ligase MARCHF11	MARCHF11			
Q9UNF1	Melanoma-associated antigen D2	MAGED2			
Q9H7P6	Multivesicular body subunit 12B	MVB12B			
Q9NPA3	Mid1-interacting protein 1	MID1IP1			
O75444	Transcription factor Maf	MAF			
Q15406	Nuclear receptor subfamily 6 group A member 1	NR6A1			
Q9H1L0	Uncharacterized protein MIR1-1HG	MIR1-1HG			
Q9GZQ8	Microtubule-associated proteins 1A/1B light chain 3B	MAP1LC3B			
P21741	Midkine	MDK			
Q8TD08	Mitogen-activated protein kinase 15	MAPK15			
Q08431	Lactadherin	MFGE8			
Q14728	Major facilitator superfamily domain-containing protein 10	MFSD10			
Q9NZW5	MAGUK p55 subfamily member 6	MPP6			
Q96LB2	Mas-related G-protein coupled receptor member X1	MRGPRX1			
Q96KJ4	Mesothelin-like protein	MSLNL			
O14511	Pro-neuregulin-2, membrane-bound isoform	NRG2			
Q8NGL4	Olfactory receptor 5D13	OR5D13			
O43749	Olfactory receptor 1F1	OR1F1			
Q8NGD2	Olfactory receptor 4K2	OR4K2			
A3KFT3	Olfactory receptor 2M5	OR2M5			
Q7RTZ1	Ovochymase-2	OVCH2			
P41586	Pituitary adenylate cyclase-activating polypeptide type I receptor	ADCYAP1R1			
Q9Y5F9	Protocadherin gamma-B6	PCDHGB6			
P42785	Lysosomal Pro-X carboxypeptidase	PRCP			
Q8IWS0	PHD finger protein 6	PHF6			
P0DJD9	Pepsin A-5	PGA5			
Q7Z412	Peroxisome assembly protein 26	PEX26			
P62195	26S proteasome regulatory subunit 8	PSMC5			
Q96PU8	Protein quaking	QKI			
Q13610	Periodic tryptophan protein 1 homolog	PWP1			
Q92878	DNA repair protein RAD50	RAD50			

Q8NHQ8	Ras association domain-containing protein 8	RASSF8			
Q5R372	Rab GTPase-activating protein 1-like	RABGAP1L			
Q9BUV8	Respirasome Complex Assembly Factor 1	RAB5IF			
P43487	Ran-specific GTPase-activating protein	RANBP1			
Q8IYV9	Izumo sperm-egg fusion protein 1	IZUMO1			
Q68EN5	Uncharacterized protein KIAA0895-like	KIAA0895L			
Q9NSK0	Kinesin light chain 4	KLC4			
O43448	Voltage-gated potassium channel subunit beta-3	KCNAB3			
Q12809	Potassium voltage-gated channel subfamily H member 2	KCNH2			
Q92764	Keratin, type I cuticular Ha5	KRT35			
Q3SY46	Keratin-associated protein 13-3	KRTAP13-3			
P46019	Phosphorylase b kinase regulatory subunit alpha, liver isoform	PHKA2			
A0A0C4DH69	Immunoglobulin kappa variable 1-9	IGKV1-9			
Q8NGD4	Olfactory receptor 4K1	OR4K1			
Q9UHY1	Nuclear receptor-binding protein	NRBP1			
Q96R27	Olfactory receptor 2M4	OR2M4			
P47888	Olfactory receptor 3A3	OR3A3			
Q9BXS6	Nucleolar and spindle-associated protein 1	NUSAP1			
Q8NGK9	Olfactory receptor 5D16	OR5D16			
A6NH00	Olfactory receptor 2T8	OR2T8			
P46459	Vesicle-fusing ATPase	NSF			
Q8NGN7	Putative olfactory receptor 10D4	OR10D4P			
Q8NG77	Olfactory receptor 2T12	OR2T12			
B2RN74	Olfactory receptor 11H12	OR11H12			
Q8WWZ8	Oncoprotein-induced transcript 3 protein	OIT3			
Q9UGF7	Olfactory receptor 12D3	OR12D3			
P12694	2-oxoisovalerate dehydrogenase subunit alpha, mitochondrial	BCKDHA			
Q9BXW6	Oxysterol-binding protein-related protein 1	OSBPL1A			
Q96RD6	Pannexin-2	PANX2			
P39877	Phospholipase A2 group V	PLA2G5			
Q9NV79	Protein-L-isoaspartate O-methyltransferase domain-containing protein 2	PCMTD2			
P30101	Protein disulfide-isomerase A3	PDIA3			
O43933	Peroxisome biogenesis factor 1	PEX1			
Q07326	Phosphatidylinositol-glycan biosynthesis class F protein	PIGF			
Q9H300	Presenilins-associated rhomboid-like protein, mitochondrial	PARL			
Q8NEB5	Phospholipid phosphatase 5	PLPP5			
Q8IY17	Patatin-like phospholipase domain-containing protein 6	PNPLA6			
A0A0J9YXQ4	Paraneoplastic antigen Ma6E	PNMA6E			
O75051	Plexin-A2	PLXNA2			
Q5H9R7	Serine/threonine-protein phosphatase 6 regulatory subunit 3	PPP6R3			
Q9NP85	Podocin	NPHS2			
Q9NNZ6	Protamine-3	PRM3			
Q9UKN5	PR domain zinc finger protein 4	PRDM4			
Q9BQI7	PH and SEC7 domain-containing protein 2	PSD2			
P18433	Receptor-type tyrosine-protein phosphatase alpha	PTPRA			

Q9UL46	Proteasome activator complex subunit 2	PSME2			
Q4JDL3	Tyrosine-protein phosphatase non-receptor type 20	PTPN20			
Q8N2H3	Pyridine nucleotide-disulfide oxidoreductase domain-containing protein 2	PYROXD2			
O00391	Sulfhydryl oxidase 1	QSOX1			
Q8N0Z8	tRNA pseudouridine synthase-like 1	PUSL1			
Q9Y3P9	Rab GTPase-activating protein 1	RABGAP1			
P20936	Ras GTPase-activating protein 1	RASA1			
Q8WXF3	Relaxin-3	RLN3			
Q9P0K7	Ankycorbin	RAI14			
P11234	Ras-related protein Ral-B	RALB			
Q9H6H4	Receptor expression-enhancing protein 4	REEP4			
P61026	Ras-related protein Rab-10	RAB10			
Q09028	Histone-binding protein RBBP4	RBBP4			
Q92804	TATA-binding protein-associated factor 2N	TAF15			
Q9NS39	Double-stranded RNA-specific editase B2	ADARB2			
Q14498	RNA-binding protein 39	RBM39			
P20336	Ras-related protein Rab-3A	RAB3A			
O60671	Cell cycle checkpoint protein	RAD1			
Q86U06	Probable RNA-binding protein 23	RBM23			
P57052	Splicing regulator RBM11	RBM11			
O43665	Regulator of G-protein signaling 10	RGS10			
Q9Y3P4	Rhomboid domain-containing protein 3	RHBDD3			
Q8TEB9	Rhomboid-related protein 4	RHBDD1			
Q15669	Rho-related GTP-binding protein RhoH	RHOH			
Q9NPQ8	Synembryn-A	RIC8A			
Q12756	Kinesin-like protein KIF1A	KIF1A			
Q5JUK3	Potassium channel subfamily T member 1	KCNT1			
P05129	Protein kinase C gamma type	PRKCG			
Q6ZWJ8	Kielin/chordin-like protein	KCP			
Q15773	Myeloid leukemia factor 2	MLF2			
O60462	Neuropilin-2	NRP2			
Q9NZB8	Molybdenum cofactor biosynthesis protein 1	MOCS1			
Q96C03	Mitochondrial dynamics protein MID49	MIEF2			
P43246	DNA mismatch repair protein Msh2	MSH2			
O75439	Mitochondrial-processing peptidase subunit beta	PMPCB			
Q9ULH7	Myocardin-related transcription factor B	MRTFB			
Q9HCI7	E3 ubiquitin-protein ligase MSL2	MSL2			
Q13875	Myelin-associated oligodendrocyte basic protein	MOBP			
P49959	Double-strand break repair protein MRE11	MRE11			
Q9Y5S2	Serine/threonine-protein kinase MRCK beta	CDC42BPB			
P36021	Monocarboxylate transporter 8	SLC16A2			
Q8WZ82	Esterase OVCA2	OVCA2			
Q8N6M0	Deubiquitinase OTUD6B	OTUD6B			
Q86VD9	GPI mannosyltransferase 4	PIGZ			
P60510	Serine/threonine-protein phosphatase 4 catalytic subunit	PPP4C			
O14830	Serine/threonine-protein phosphatase with EF-hands 2	PPEF2			
O75688	Protein phosphatase 1B	PPM1B			

Q99575	Ribonucleases P/MRP protein subunit POP1	POP1			
Q9UPG8	Zinc finger protein PLAGL2	PLAGL2			
Q9H4M7	Pleckstrin homology domain-containing family A member 4	PLEKHA4			
P53816	Phospholipase A and acyltransferase 3	PLAAT3			
Q9HAU0	Pleckstrin homology domain-containing family A member 5	PLEKHA5			
Q9Y2H5	Pleckstrin homology domain-containing family A member 6	PLEKHA6			
Q9UL19	Phospholipase A and acyltransferase 4	PLAAT4			
Q96KN8	Phospholipase A and acyltransferase 5	PLAAT5			
O75364	Pituitary homeobox 3	PITX3			
P51178	1-phosphatidylinositol 4,5-bisphosphate phosphodiesterase delta-1	PLCD1			
Q14997	Proteasome activator complex subunit 4	PSME4			
Q02127	Dihydroorotate dehydrogenase	DHODH			
Q7RTV5	Peroxiredoxin-like 2C	PRXL2C			
Q9BZE2	tRNA pseudouridine	PUS3			
P0DJD4	RNA-binding motif protein, Y chromosome, family 1 member C	RBMY1C			
Q9BTL3	RNA guanine-N7 methyltransferase activating subunit	RAMAC			
O95072	Meiotic recombination protein REC8 homolog	REC8			
Q8WXH6	Ras-related protein Rab-40A	RAB40A			
Q8NC74	RBBP8 N-terminal-like protein	RBBP8NL			
Q8TDY2	RB1-inducible coiled-coil protein	RB1CC1			
Q9H902	Receptor expression-enhancing protein 1	REEP1			
Q6NUK4	Receptor expression-enhancing protein 3	REEP3			
Q96AH8	Ras-related protein Rab-7b	RAB7B			
Q6ZSC3	RNA-binding protein 43	RBM43			
Q9BRR9	Rho GTPase-activating protein 9	ARHGAP9			
P51148	Ras-related protein Rab-5C	RAB5C			
Q6WKZ4	Rab11 family-interacting protein 1	RAB11FIP1			
A6NKT7	RanBP2-like and GRIP domain-containing protein 3	RGPD3			
Q7Z3J3	RanBP2-like and GRIP domain-containing protein 4	RGPD4			
Q96MK2	RIPOR family member 3	RIPOR3			
Q14699	Raftlin	RFTN1			
P09455	Retinol-binding protein 1	RBP1			
Q8IUF8	Ribosomal oxygenase 2	RIOX2			
Q53G59	Kelch-like protein 12	KLHL12			
P48730	Casein kinase I isoform delta	CSNK1D			
Q14500	ATP-sensitive inward rectifier potassium channel 12	KCNJ12			
Q3LHN2	Keratin-associated protein 19-2	KRTAP19-2			
P22460	Potassium voltage-gated channel subfamily A member 5	KCNA5			
Q8N5Z5	BTB/POZ domain-containing protein KCTD17	KCTD17			
Q96EK9	Protein KTI12 homolog	KTI12			
A6PVL3	Kinocilin	KNCN			
Q5JUW0	KRAB domain-containing protein 4	KRBOX4			
P01599	Immunoglobulin kappa variable 1-17	IGKV1-17			
P10620	Microsomal glutathione S-transferase 1	MGST1			
O14950	Myosin regulatory light chain 12B	MYL12B			

Q8NBP5	Major facilitator superfamily domain-containing protein 9	MFSD9			
O43347	RNA-binding protein Musashi homolog 1	MSI1			
Q01726	Melanocyte-stimulating hormone receptor	MC1R			
Q6Q8B3	Cell surface glycoprotein CD200 receptor 2	CD200R1L			
Q9NXE4	Sphingomyelin phosphodiesterase 4	SMPD4			
Q99748	Neurturin	NRTN			
O95478	Ribosome biogenesis protein NSA2 homolog	NSA2			
Q5BKU9	Oxidoreductase-like domain-containing protein 1	OXLD1			
Q6IF00	Olfactory receptor 2T2	OR2T2			
Q12769	Nuclear pore complex protein Nup160	NUP160			
Q96RD0	Olfactory receptor 8B2	OR8B2			
P41217	OX-2 membrane glycoprotein	CD200			
Q5TZ20	Olfactory receptor 2G6	OR2G6			
O60431	Olfactory receptor 1I1	OR1I1			
P08100	Rhodopsin	RHO			
Q5TC84	Opioid growth factor receptor-like protein 1	OGFRL1			
Q8NGT7	Olfactory receptor 2A12	OR2A12			
Q8NH54	Olfactory receptor 56A3	OR56A3			
Q9BR26	Osteoclast stimulatory transmembrane protein	OCSTAMP			
Q96RD3	Olfactory receptor 52E6	OR52E6			
Q8NGH8	Olfactory receptor 56A4	OR56A4			
P0C617	Olfactory receptor 5AL1	OR5AL1			
Q5JVF3	PCI domain-containing protein 2	PCID2			
A8CG34	Nuclear envelope pore membrane protein POM 121C	POM121C			
A0A0B4J2A2	Peptidyl-prolyl cis-trans isomerase A-like 4C	PPIAL4C			
Q9H813	Proton-activated chloride channel	PACC1			
Q6L8Q7	2',5'-phosphodiesterase 12	PDE12			
Q16625	Occludin	OCLN			
P40425	Pre-B-cell leukemia transcription factor 2	PBX2			
Q9NXK6	Membrane progestin receptor gamma	PAQR5			
O60829	P antigen family member 4	PAGE4			
Q460N3	Protein mono-ADP-ribosyltransferase PARP15	PARP15			
Q9NP56	cAMP-specific 3',5'-cyclic phosphodiesterase 7B	PDE7B			
Q9BYG5	Partitioning defective 6 homolog beta	PARD6B			
Q9Y5E7	Protocadherin beta-2	PCDHB2			
P05120	Plasminogen activator inhibitor 2	SERPINB2			
P41231	P2Y purinoceptor 2	P2RY2			
Q9Y5I3	Protocadherin alpha-1	PCDHA1			
A5PLL7	Plasmanylethanolamine desaturase	PEDS1			
Q96G91	P2Y purinoceptor 11	P2RY11			
Q96NT5	Proton-coupled folate transporter	SLC46A1			
A6NDG6	Glycerol-3-phosphate phosphatase	PGP			
P53609	Geranylgeranyl transferase type-1 subunit beta	PGGT1B			
O94880	PHD finger protein 14	PHF14			
P07585	Decorin	DCN			
Q96HS1	Serine/threonine-protein phosphatase PGAM5, mitochondrial	PGAM5			
Q9NRX1	RNA-binding protein PNO1	PNO1			

Q99640	Membrane-associated tyrosine- and threonine-specific cdc2-inhibitory kinase	PKMYT1			
P40306	Proteasome subunit beta type-10	PSMB10			
Q9GZP4	PITH domain-containing protein 1	PITHD1			
A5LHX3	Proteasome subunit beta type-11	PSMB11			
Q6P5Z2	Serine/threonine-protein kinase N3	PKN3			
P86479	Proline-rich protein 20C	PRR20C			
Q9BWN1	Proline-rich protein 14	PRR14			
Q9NQX0	Putative histone-lysine N-methyltransferase PRDM6	PRDM6			
A0A0G2JMD5	PRAME family member 33	PRAMEF33			
P02812	Basic salivary proline-rich protein	PRB2			
Q9NQV5	PR domain-containing protein 11	PRDM11			
Q14691	DNA replication complex GINS protein PSF1	GINS1			
Q15238	Pregnancy-specific beta-1-glycoprotein 5	PSG5			
Q96QH2	PML-RARA-regulated adapter molecule 1	PRAM1			
O60809	PRAME family member 10	PRAMEF10			
Q9H0K6	Pseudouridylate synthase 7 homolog-like protein	PUS7L			
Q9HBH5	Retinol dehydrogenase 14	RDH14			
O94761	ATP-dependent DNA helicase Q4	RECQL4			
Q8IUW5	RELT-like protein 1	RELL1			
P11233	Ras-related protein Ral-A	RALA			
O60894	Receptor activity-modifying protein 1	RAMP1			
Q96S21	Ras-related protein Rab-40C	RAB40C			
Q5HYI8	Rab-like protein 3	RABL3			
Q96H35	Probable RNA-binding protein 18	RBM18			
Q5JS13	Ras-specific guanine nucleotide-releasing factor RalGPS1	RALGPS1			
Q9H910	Jupiter microtubule associated homolog 2	JPT2			
O76095	Protein JTB	JTB			
P23458	Tyrosine-protein kinase JAK1	JAK1			
Q8WYK2	Jun dimerization protein 2	JDP2			
P55073	Thyroxine 5-deiodinase	DIO3			
Q15181	Inorganic pyrophosphatase	PPA1			
Q9UK17	Potassium voltage-gated channel subfamily D member 3	KCND3			
P49862	Kallikrein-7	KLK7			
Q8N5I3	Potassium channel regulatory protein	KCNRG			
Q8NEE0	Putative uncharacterized protein KLHL30-AS1	KLHL30-AS1			
Q9UGI6	Small conductance calcium-activated potassium channel protein 3	KCNN3			
Q3LI81	Keratin-associated protein 27-1	KRTAP27-1			
P60410	Keratin-associated protein 10-8	KRTAP10-8			
Q3LI66	Keratin-associated protein 6-2	KRTAP6-2			
Q719H9	BTB/POZ domain-containing protein KCTD1	KCTD1			
Q9UPM6	LIM/homeobox protein Lhx6	LHX6			
P32004	Neural cell adhesion molecule L1	L1CAM			
A0A0C4DH24	Immunoglobulin kappa variable 6-21	IGKV6-21			
Q5TA81	Late cornified envelope protein 2C	LCE2C			
Q96BZ8	Leukocyte receptor cluster member 1	LENG1			
Q96B70	Leukocyte receptor cluster member 9	LENG9			
P50281	Matrix metalloproteinase-14	MMP14			
Q9H306	Matrix metalloproteinase-27	MMP27			

O60291	E3 ubiquitin-protein ligase	MGRN1			
Q9H3V2	Membrane-spanning 4-domains subfamily A member 5	MS4A5			
Q02750	Dual specificity mitogen-activated protein kinase kinase 1	MAP2K1			
Q8NGZ3	Olfactory receptor 13G1	OR13G1			
O96007	Molybdopterin synthase catalytic subunit	MOCS2			
P34130	Neurotrophin-4	NTF4			
Q7Z6K4	Notch-regulated ankyrin repeat-containing protein	NRARP			
Q9NSY0	Nuclear receptor-binding protein 2	NRBP2			
Q9NRN5	Olfactomedin-like protein 3	OLFML3			
Q5VVQ6	Ubiquitin thioesterase OTU1	YOD1			
Q8NGY0	Olfactory receptor 10X1	OR10X1			
Q8NGB8	Olfactory receptor 4F15	OR4F15			
Q56VL3	OCIA domain-containing protein	OCIAD2			
Q86VP3	Phosphofurin acidic cluster sorting protein 2	PACS2			
F5H284	Peptidyl-prolyl cis-trans isomerase A-like 4D	PPIAL4D			
O14813	Paired mesoderm homeobox protein 2A	PHOX2A			
Q99570	Phosphoinositide 3-kinase regulatory subunit 4	PIK3R4			
Q8N2W9	E3 SUMO-protein ligase PIAS4	PIAS4			
Q6PCE3	Glucose 1,6-bisphosphate synthase	PGM2L1			
O15212	Prefoldin subunit 6	PFDN6			
Q96FA3	E3 ubiquitin-protein ligase pellino homolog 1	PELI1			
Q9HCM2	Plexin-A4	PLXNA4			
Q8WVV4	Protein POF1B	POF1B			
P09619	Platelet-derived growth factor receptor beta	PDGFRB			
P49763	Placenta growth factor	PGF			
Q8NFJ6	Prokineticin receptor 2	PROKR2			
Q00722	1-phosphatidylinositol 4,5-bisphosphate phosphodiesterase beta-2	PLCB2			
Q0ZLH3	Pejvakin	PJVK			
A6NEY8	Putative prolyl-tRNA synthetase associated domain-containing protein 1	PRORSD1P			
P49721	Proteasome subunit beta type-2	PSMB2			
Q8WUY3	Protein prune homolog 2	PRUNE2			
P35080	Profilin-2	PFN2			
Q96MT3	Prickle-like protein 1	PRICKLE1			
Q86SH4	Putative testis-specific prion protein	PRNT			
Q9BU68	Proline-rich protein 15-like	PRR15L			
Q86UN3	Reticulon-4 receptor-like 2	RTN4RL2			
Q9UHI5	Large neutral amino acids transporter small subunit 2	SLC7A8			
Q9NQ76	Matrix extracellular phosphoglycoprotein	MEPE			
Q9H2D1	Mitochondrial folate transporter/carrier	SLC25A32			
Q96A46	Mitoferrin-2	SLC25A28			
P40692	DNA mismatch repair protein	MLH1			
Q495T6	Membrane metallo-endopeptidase-like 1	MMEL1			
Q9HD23	Magnesium transporter MRS2 homolog, mitochondrial	MRS2			
Q8TD46	Cell surface glycoprotein CD200 receptor 1	CD200R1			
Q5VT25	Serine/threonine-protein kinase MRCK alpha	CDC42BPA			

Q6UXH9	Inactive serine protease PAMR1	PAMR1			
Q9Y2Z2	Protein MTO1 homolog, mitochondrial	MTO1			
Q8IVN3	Musculoskeletal embryonic nuclear protein 1	MUSTN1			
P42345	Serine/threonine-protein kinase mTOR	MTOR			
P80297	Metallothionein-1X	MT1X			
Q4G0N4	NAD kinase 2, mitochondrial	NADK2			
Q9BZK3	Putative nascent polypeptide-associated complex subunit alpha-like protein	NACA4P			
Q02083	N-acylethanolamine-hydrolyzing acid amidase	NAAA			
P41227	N-alpha-acetyltransferase 10	NAA10			
Q9Y4I1	Unconventional myosin-Va	MYO5A			
A6NI72	Putative neutrophil cytosol factor 1B	NCF1B			
P0C2Y1	Putative neuroblastoma breakpoint family member 7	NBPF7			
Q6PIU2	Neutral cholesterol ester hydrolase 1	NCEH1			
Q5VU43	Myomegalin	PDE4DIP			
P40205	N-cym protein	MYCNOS			
Q8NA29	Sodium-dependent lysophosphatidylcholine symporter 1	MFSD2A			
Q9UKX3	Myosin-13	MYH13			
Q07444	NKG2-E type II integral membrane protein	KLRC3			
Q8NFA2	NADPH oxidase organizer 1	NOXO1			
O75323	Protein NipSnap homolog 2	NIPSNAP2			
Q9BZQ4	Nicotinamide/nicotinic acid mononucleotide adenylyltransferase 2	NMNAT2			
Q15818	Neuronal pentraxin-1	NPTX1			
O15399	Glutamate receptor ionotropic, NMDA 2D	GRIN2D			
P61582	Endogenous retrovirus group K member 7 Np9 protein	ERVK-7			
Q5SYE7	NHS-like protein 1	NHSL1			
O14745	Na	SLC9A3R1			
Q9Y314	Nitric oxide synthase-interacting protein	NOSIP			
O75489	NADH dehydrogenase	NDUFS3			
O75607	Nucleoplasmin-3	NPM3			
A8MQ27	E3 ubiquitin-protein ligase NEURL1B	NEURL1B			
O95182	NADH dehydrogenase	NDUFA7			
O43613	Orexin receptor type 1	HCRTR1			
Q8NH41	Olfactory receptor 4K15	OR4K15			
Q8NHW6	Otospiralin	OTOS			
Q9BW27	Nuclear pore complex protein Nup85	NUP85			
Q8TCD5	5'	NT5C			
Q8NH42	Olfactory receptor 4K13	OR4K13			
Q8NGD0	Olfactory receptor 4M1	OR4M1			
Q96HC4	PDZ and LIM domain protein 5	PDLIM5			
Q7Z3E1	Protein mono-ADP-ribosyltransferase TIPARP	TIPARP			
O60733	85/88 kDa calcium-independent phospholipase A2	PLA2G6			
A0A1B0GUJ8	Paraneoplastic antigen-like protein 8C	PNMA8C			
P23284	Peptidyl-prolyl cis-trans isomerase B	PPIB			
O60260	E3 ubiquitin-protein ligase parkin	PRKN			
O14603	PTPN13-like protein, Y-linked	PRY			
O43474	Krueppel-like factor 4	KLF4			

Q6ZMT4	Lysine-specific demethylase 7A	KDM7A			
Q6ZV70	LanC-like protein 3	LANCL3			
P48729	Casein kinase I isoform alpha	CSNK1A1			
P52954	Transcription factor LBX1	LBX1			
Q9BXB1	Leucine-rich repeat-containing G-protein coupled receptor 4	LGR4			
P10916	Myosin regulatory light chain 2, ventricular/cardiac muscle isoform	MYL2			
Q7RTY0	Monocarboxylate transporter 13	SLC16A13			
Q2QL34	Mpv17-like protein	MPV17L			
Q2TV78	Putative macrophage stimulating 1-like protein	MST1L			
Q9BUK6	Protein misato homolog 1	MSTO1			
P28360	Homeobox protein MSX-1	MSX1			
Q8WXI7	Mucin-16	MUC16			
Q9BTC8	Metastasis-associated protein MTA3	MTA3			
Q86W24	NACHT, LRR and PYD domains-containing protein 14	NLRP14			
Q93070	Ecto-ADP-ribosyltransferase 4	ART4			
P15531	Nucleoside diphosphate kinase A	NME1			
P35579	Myosin-9	MYH9			
Q9H1R3	Myosin light chain kinase 2, skeletal/cardiac muscle	MYLK2			
Q9HCD5	Nuclear receptor coactivator 5	NCOA5			
Q8N8D7	Sodium/potassium-transporting ATPase subunit beta-1-interacting protein 3	NKAIN3			
Q14919	Dr1-associated corepressor	DRAP1			
Q9NPG2	Neuroglobin	NGB			
P43699	Homeobox protein Nkx-2.1	NKX2-1			
O60393	Homeobox protein NOBOX	NOBOX			
P78367	Homeobox protein Nkx-3.2	NKX3-2			
Q96NY8	Nectin-4	NECTIN4			
Q8TCU5	Glutamate receptor ionotropic, NMDA 3A	GRIN3A			
Q96MY1	Nucleolar protein 4-like	NOL4L			
O43676	NADH dehydrogenase	NDUFB3			
Q15599	Na	SLC9A3R2			
P0DQD5	Neuropeptide Y receptor type 4-2	NPY4R2			
Q9NZM5	Ribosome biogenesis protein NOP53	NOP53			
Q99784	Noelin	OLFM1			
O95644	Nuclear factor of activated T-cells, cytoplasmic 1	NFATC1			
Q7RTW8	Otoancorin	OTOA			
O60422	One cut domain family member 3	ONECUT3			
Q96L73	Histone-lysine N-methyltransferase, H3 lysine-36 specific	NSD1			
Q8NGB9	Olfactory receptor 4F6	OR4F6			
Q96MF7	E3 SUMO-protein ligase NSE2	NSMCE2			
Q6DKJ4	Nucleoredoxin	NXN			
A6NL08	Olfactory receptor 6C75	OR6C75			
O15243	Leptin receptor gene-related protein	LEPROT			
Q8NHC4	Olfactory receptor 10J5	OR10J5			
P0C7T3	Olfactory receptor 56A5	OR56A5			
Q8NGF0	Olfactory receptor 52B6	OR52B6			
Q8NH74	Olfactory receptor 10A6	OR10A6			
Q8NGY7	Putative olfactory receptor 10J6	OR10J6P			
Q9UBL9	P2X purinoceptor 2	P2RX2			
Q9H4L5	Oxysterol-binding protein-related protein 3	OSBPL3			
Q96KW2	POM121-like protein 2	POM121L2			
Q9BZF3	Oxysterol-binding protein-related protein 6	OSBPL6			
O76002	Olfactory receptor 2J2	OR2J2			
P0C623	Olfactory receptor 4Q2	OR4Q2			

Q6UW60	Proprotein convertase subtilisin/kexin type 4	PCSK4			
P22079	Lactoperoxidase	LPO			
Q7Z309	PABIR family member 2	PABIR2			
Q8TCW9	Prokineticin receptor 1	PROKR1			
Q8NAT1	Protein O-linked-mannose beta-1,4-N-acetylglucosaminyltransferase 2	POMGNT2			
Q9UMS4	Pre-mRNA-processing factor 19	PRPF19			
Q6ZSY5	Protein phosphatase 1 regulatory subunit 3F	PPP1R3F			
O43490	Prominin-1	PROM1			
Q9UHX1	Poly	PUF60			
Q96D70	R3H domain-containing protein 4	R3HDM4			
P0C870	Bifunctional peptidase and	JMJD7			
P05161	Ubiquitin-like protein ISG15	ISG15			
Q9Y219	Protein jagged-2	JAG2			
Q86VS3	IQ domain-containing protein H	IQCH			
P14616	Insulin receptor-related protein	INSRR			
O15037	Protein KHNYN	KHNYN			
Q8WZA9	Immunity-related GTPase family Q protein	IRGQ			
Q9Y257	Potassium channel subfamily K member 6	KCNK6			
Q9BYR8	Keratin-associated protein 3-1	KRTAP3-1			
Q9Y597	BTB/POZ domain-containing protein KCTD3	KCTD3			
Q6ZP29	Lysosomal amino acid transporter 1 homolog	SLC66A1			
P0DMU2	Putative olfactory receptor 8G3 pseudogene	OR8G3P			
Q8NGM1	Olfactory receptor 4C15	OR4C15			
Q8NG98	Olfactory receptor 7D4	OR7D4			
Q8NGG5	Olfactory receptor 8K1	OR8K1			
Q9GZY0	Nuclear RNA export factor 2	NXF2			
Q8N323	NXPE family member 1	NXPE1			
Q02509	Otoconin-90	OC90			
Q8NGI2	Olfactory receptor 52N4	OR52N4			
Q8NH90	Olfactory receptor 5AK2	OR5AK2			
Q8NH56	Olfactory receptor 52N5	OR52N5			
O76001	Olfactory receptor 2J3	OR2J3			
Q68DD2	Cytosolic phospholipase A2 zeta	PLA2G4F			
Q9BZ23	Pantothenate kinase 2, mitochondrial	PANK2			
P15735	Phosphorylase b kinase gamma catalytic chain, liver/testis isoform	PHKG2			
Q9BZM1	Group XIIA secretory phospholipase A2	PLA2G12A			
Q96EK2	PHD finger protein 21B	PHF21B			
Q76G19	PDZ domain-containing protein 4	PDZD4			
P05186	Alkaline phosphatase, tissue-nonspecific isozyme	ALPL			
P0CG39	POTE ankyrin domain family member J	POTEJ			
Q9HAB8	Phosphopantothenate--cysteine ligase	PPCS			
Q8WVI7	Protein phosphatase 1 regulatory subunit 1C	PPP1R1C			
B2RU33	POTE ankyrin domain family member C	POTEC			
E7EW31	Proline-rich basic protein 1	PROB1			
P07225	Vitamin K-dependent protein S	PROS1			
Q16651	Prostasin	PRSS8			
P49720	Proteasome subunit beta type-3	PSMB3			
Q86YV5	Inactive tyrosine-protein kinase PRAG1	PRAG1			
Q5R3F8	Protein phosphatase 1 regulatory subunit 29	ELFN2			

Q96PX9	Pleckstrin homology domain-containing family G member 4B	PLEKHG4B			
O00231	26S proteasome non-ATPase regulatory subunit 11	PSMD11			
P26045	Tyrosine-protein phosphatase non-receptor type 3	PTPN3			
Q06203	Amidophosphoribosyltransferase	PPAT			
P31930	Cytochrome b-c1 complex subunit 1, mitochondrial	UQCRC1			
P15918	V	RAG1			
Q15434	RNA-binding motif, single-stranded-interacting protein 2	RBMS2			
P26374	Rab proteins geranylgeranyltransferase component A 2	CHML			
P61576	Endogenous retrovirus group K member 104 Rec protein	HERV-K104			
Q9P258	Protein RCC2	RCC2			
Q8IUD2	ELKS/Rab6-interacting/CAST family member 1	ERC1			
Q7Z6I6	Rho GTPase-activating protein 30	ARHGAP30			
Q6ZRI8	Rho GTPase-activating protein 36	ARHGAP36			
Q92785	Zinc finger protein ubi-d4	DPF2			
Q9H4E5	Rho-related GTP-binding protein RhoJ	RHOJ			
O75679	Ret finger protein-like 3	RFPL3			
P23921	Ribonucleoside-diphosphate reductase large subunit	RRM1			
P00797	Renin	REN			
Q8N8K9	Uncharacterized protein KIAA1958	KIAA1958			
O94829	Importin-13	IPO13			
Q9UK53	Inhibitor of growth protein 1	ING1			
P51812	Ribosomal protein S6 kinase alpha-3	RPS6KA3			
Q9BQ31	Potassium voltage-gated channel subfamily S member 3	KCNS3			
P43626	Killer cell immunoglobulin-like receptor 2DL1	KIR2DL1			
O43240	Kallikrein-10	KLK10			
Q9BYU5	Keratin-associated protein 2-1	KRTAP2-1			
A6NGR9	Maestro heat-like repeat-containing protein family member 6	MROH6			
O00180	Potassium channel subfamily K member 1	KCNK1			
Q3LI70	Keratin-associated protein 19-6	KRTAP19-6			
Q16558	Calcium-activated potassium channel subunit beta-1	KCNMB1			
Q96I82	Kazal-type serine protease inhibitor domain-containing protein 1	KAZALD1			
Q96BN8	Ubiquitin thioesterase otulin	OTULIN			
O00110	Putative transcription factor ovo-like protein 3	OVOL3			
Q9UGF6	Olfactory receptor 5V1	OR5V1			
Q8NGE9	Olfactory receptor 9Q2	OR9Q2			
Q7RTU3	Oligodendrocyte transcription factor 3	OLIG3			
Q8NGU1	Putative olfactory receptor 9A1	OR9A1P			
O75147	Obscurin-like protein 1	OBSL1			
Q9Y5P1	Olfactory receptor 51B2	OR51B2			
Q8NGI0	Olfactory receptor 52N2	OR52N2			
Q8NH19	Olfactory receptor 10AG1	OR10AG1			
Q8NGX0	Olfactory receptor 11L1	OR11L1			
Q86UW1	Organic solute transporter subunit alpha	SLC51A			
Q9UKG9	Peroxisomal carnitine O-octanoyltransferase	CROT			

Q8IXM7	Outer dense fiber protein 3-like protein 1	ODF3L1			
Q8NG92	Olfactory receptor 13H1	OR13H1			
Q86WC4	Osteopetrosis-associated transmembrane protein 1	OSTM1			
Q3SYA9	Putative POM121-like protein 1	POM121L1			
Q92882	Osteoclast-stimulating factor 1	OSTF1			
Q504Q3	PAN2-PAN3 deadenylation complex catalytic subunit PAN2	PAN2			
Q13310	Polyadenylate-binding protein 4	PABPC4			
O15496	Group 10 secretory phospholipase A2	PLA2G10			
P13674	Prolyl 4-hydroxylase subunit alpha-1	P4HA1			
P16284	Platelet endothelial cell adhesion molecule	PECAM1			
Q9P215	Pogo transposable element with KRAB domain	POGK			
A0A0A6YYL3	POTE ankyrin domain family member B	POTEB			
H3BUK9	POTE ankyrin domain family member B2	POTEB2			
Q5JR12	Protein phosphatase 1J	PPM1J			
Q8NE79	Blood vessel epicardial substance	BVES			
A5A3E0	POTE ankyrin domain family member F	POTEF			
P08F94	Fibrocystin	PKHD1			
P85299	Proline-rich protein 5	PRR5			
Q8TD55	Pleckstrin homology domain-containing family O member 2	PLEKHO2			
Q2TBC4	Prickle-like protein 4	PRICKLE4			
Q4KWH8	1-phosphatidylinositol 4,5-bisphosphate phosphodiesterase eta-1	PLCH1			
Q13523	Serine/threonine-protein kinase PRP4 homolog	PRPF4B			
Q9NZH4	Putative pituitary tumor-transforming gene 3 protein	PTTG3P			
Q86UN2	Reticulon-4 receptor-like 1	RTN4RL1			
Q8NG50	RAD52 motif-containing protein 1	RDM1			
Q08999	Retinoblastoma-like protein 2	RBL2			
Q9Y6I8	Peroxisomal membrane protein 4	PXMP4			
Q9H4I0	Double-strand-break repair protein rad21-like protein 1	RAD21L1			
P10114	Ras-related protein Rap-2a	RAP2A			
P0DJD3	RNA-binding motif protein, Y chromosome, family 1 member A1	RBMY1A1			
P38159	RNA-binding motif protein, X chromosome	RBMX			
Q14257	Reticulocalbin-2	RCN2			
Q9H5N1	Rab GTPase-binding effector protein 2	RABEP2			
Q14088	Ras-related protein Rab-33A	RAB33A			
Q16576	Histone-binding protein RBBP7	RBBP7			
P61575	Endogenous retrovirus group K member 8 Rec protein	ERVK-8			
Q96E17	Ras-related protein Rab-3C	RAB3C			
Q9P2N2	Rho GTPase-activating protein 28	ARHGAP28			
O15211	Ral guanine nucleotide dissociation stimulator-like 2	RGL2			
Q96T37	RNA-binding protein 15	RBM15			
Q96IZ5	RNA-binding protein 41	RBM41			
Q92546	RAB6A-GEF complex partner protein 2	RGP1			
P20340	Ras-related protein Rab-6A	RAB6A			
O75570	Peptide chain release factor 1, mitochondrial	MTRF1			
Q9H310	Ammonium transporter Rh type B	RHBG			

Q92900	Regulator of nonsense transcripts 1	UPF1			
Q8N443	RIB43A-like with coiled-coils protein 1	RIBC1			
Q6NT55	Cytochrome P450 4F22	CYP4F22			
A0A140G945	Alpha-crystallin A2 chain	CRYAA2			
Q9H336	Cysteine-rich secretory protein LCCL domain-containing 1	CRISPLD1			
Q96I36	Cytochrome c oxidase assembly protein COX14	COX14			
P10589	COUP transcription factor 1	NR2F1			
Q8N9M1	Uncharacterized protein C19orf47	C19orf47			
P0DMV0	Cancer/testis antigen family 45 member A7	CT45A7			
Q9H4R4	Putative nuclear receptor corepressor 1-like protein NCOR1P1	NCOR1P1			
A1L168	Uncharacterized protein C20orf202	C20orf202			
P0C2S0	Cortexin-2	CTXN2			
P0C2W7	Cancer/testis antigen 47B	CT47B1			
Q8TCZ7	Putative uncharacterized protein encoded by LINC00308	LINC00308			
P59091	Putative uncharacterized protein encoded by LINC00315	LINC00315			
Q9NTQ9	Gap junction beta-4 protein	GJB4			
Q13363	C-terminal-binding protein 1	CTBP1			
Q8WWI5	Choline transporter-like protein 1	SLC44A1			
O15255	CAAX box protein 1	RTL8C			
O60888	Protein CutA	CUTA			
Q96P56	Cation channel sperm-associated protein 2	CATSPER2			
Q8IU60	m7GpppN-mRNA hydrolase	DCP2			
Q5SW24	Dapper homolog 2	DACT2			
Q99259	Glutamate decarboxylase 1	GAD1			
Q8NHQ9	ATP-dependent RNA helicase DDX55	DDX55			
A6NKP2	Putative short-chain dehydrogenase/reductase family 42E member 2	SDR42E2			
Q5T1V6	Probable ATP-dependent RNA helicase DDX59	DDX59			
Q8TCX1	Cytoplasmic dynein 2 light intermediate chain 1	DYNC2LI1			
Q9Y4B6	DDB1- and CUL4-associated factor 1	DCAF1			
Q8NFT6	Protein DBF4 homolog B	DBF4B			
Q9UER7	Death domain-associated protein 6	DAXX			
Q9NXF7	DDB1- and CUL4-associated factor 16	DCAF16			
P28067	HLA class II histocompatibility antigen, DM alpha chain	HLA-DMA			
Q92796	Disks large homolog 3	DLG3			
Q6UY11	Protein delta homolog 2	DLK2			
P49619	Diacylglycerol kinase gamma	DGKG			
O75912	Diacylglycerol kinase iota	DGKI			
Q9H1X3	DnaJ homolog subfamily C member 25	DNAJC25			
Q92838	Ectodysplasin-A	EDA			
Q16690	Dual specificity protein phosphatase 5	DUSP5			
O14641	Segment polarity protein dishevelled homolog DVL-2	DVL2			
Q92782	Zinc finger protein neuro-d4	DPF1			
P06028	Glycophorin-B	GYPB			
P63167	Dynein light chain 1, cytoplasmic	DYNLL1			

Q9NVP4	Double zinc ribbon and ankyrin repeat-containing protein 1	DZANK1			
P51654	Glypican-3	GPC3			
Q8N682	DNA damage-regulated autophagy modulator protein 1	DRAM1			
Q5JR98	Dynein light chain Tctex-type 4	DYNLT4			
Q9NQ84	G-protein coupled receptor family C group 5 member C	GPRC5C			
Q9NZD1	G-protein coupled receptor family C group 5 member D	GPRC5D			
P81274	G-protein-signaling modulator 2	GPSM2			
P78333	Glypican-5	GPC5			
Q96T91	Glycoprotein hormone alpha-2	GPHA2			
Q9Y625	Glypican-6	GPC6			
Q5T2R2	All trans-polyprenyl-diphosphate synthase PDSS1	PDSS1			
Q9Y5L3	Ectonucleoside triphosphate diphosphohydrolase 2	ENTPD2			
P78545	ETS-related transcription factor Elf-3	ELF3			
Q9N2J8	HERV-H_2q24.1 provirus ancestral Env polyprotein				
Q16206	Ecto-NOX disulfide-thiol exchanger 2	ENOX2			
P11161	E3 SUMO-protein ligase EGR2	EGR2			
P09104	Gamma-enolase	ENO2			
P00740	Coagulation factor IX	F9			
P62508	Estrogen-related receptor gamma	ESRRG			
Q5FWF5	N-acetyltransferase ESCO1	ESCO1			
A6NIJ5	Putative protein FAM90A20P	FAM90A20P			
A8MXZ1	Putative protein FAM90A23P	FAM90A23P			
Q9BTP7	Fanconi anemia core complex-associated protein 24	FAAP24			
Q5HY64	Putative protein FAM47C	FAM47C			
A6NHR8	Putative protein FAM47D	FAM47DP			
P09467	Fructose-1,6-bisphosphatase 1	FBP1			
Q5TYM5	Protein FAM72A	FAM72A			
P12318	Low affinity immunoglobulin gamma Fc region receptor II-a	FCGR2A			
Q9UBQ6	Exostosin-like 2	EXTL2			
O14526	F-BAR domain only protein 1	FCHO1			
O94887	FERM, ARHGEF and pleckstrin domain-containing protein 2	FARP2			
O15520	Fibroblast growth factor 10	FGF10			
Q14192	Four and a half LIM domains protein 2	FHL2			
Q9P2Q2	FERM domain-containing protein 4A	FRMD4A			
Q16595	Frataxin, mitochondrial	FXN			
O15117	FYN-binding protein 1	FYB1			
Q5VYV0	Forkhead box protein B2	FOXB2			
O60883	G-protein coupled receptor 37-like 1	GPR37L1			
Q68CQ7	Glycosyltransferase 8 domain-containing protein 1	GLT8D1			
Q6IB77	Glycine N-acyltransferase	GLYAT			
P20718	Granzyme H	GZMH			
Q6ZVE7	Vesicle transport protein GOT1A	GOLT1A			
Q8TDV0	G-protein coupled receptor 151	GPR151			
Q9UNW8	Probable G-protein coupled receptor 132	GPR132			
P01275	Pro-glucagon	GCG			
Q9NZD2	Glycolipid transfer protein	GLTP			
P48507	Glutamate--cysteine ligase regulatory subunit	GCLM			
Q4VC39	Putative HIG1 domain family member 2B	HIGD2B			

Q9UPZ3	Hermansky-Pudlak syndrome 5 protein	HPS5			
P34932	Heat shock 70 kDa protein 4	HSPA4			
P17481	Homeobox protein Hox-B8	HOXB8			
P09017	Homeobox protein Hox-C4	HOXC4			
Q9NWW0	Host cell factor C1 regulator 1	HCFC1R1			
O43432	Eukaryotic translation initiation factor 4 gamma 3	EIF4G3			
P84074	Neuron-specific calcium-binding protein hippocalcin	HPCA			
Q2TB90	Hexokinase HKDC1	HKDC1			
P04439	HLA class I histocompatibility antigen, A alpha chain	HLA-A			
Q14623	Indian hedgehog protein	IHH			
Q9H7X7	Intraflagellar transport protein 22 homolog	IFT22			
A9QM74	Importin subunit alpha-8	KPNA7			
P20810	Calpastatin	CAST			
Q6UWB1	Interleukin-27 receptor subunit alpha	IL27RA			
Q13683	Integrin alpha-7	ITGA7			
Q9P055	JNK1/MAPK8-associated membrane protein	JKAMP			
P19827	Inter-alpha-trypsin inhibitor heavy chain H1	ITIH1			
O14990	Protein phosphatase inhibitor 2 family member C	PPP1R2C			
Q9NSA2	Potassium voltage-gated channel subfamily D member 1	KCND1			
Q2TBA0	Kelch-like protein 40	KLHL40			
Q75V66	Anoctamin-5	ANO5			
Q8TER5	Rho guanine nucleotide exchange factor 40	ARHGEF40			
O00203	AP-3 complex subunit beta-1	AP3B1			
Q9NWB6	Arginine and glutamate-rich protein 1	ARGLU1			
Q8IXF9	Aquaporin-12A	AQP12A			
O43307	Rho guanine nucleotide exchange factor 9	ARHGEF9			
Q8NFD5	AT-rich interactive domain-containing protein 1B	ARID1B			
Q9NQ33	Achaete-scute homolog 3	ASCL3			
Q9NT62	Ubiquitin-like-conjugating enzyme ATG3	ATG3			
P17405	Sphingomyelin phosphodiesterase	SMPD1			
Q8NCT1	Arrestin domain-containing protein 4	ARRDC4			
Q9H2C2	Protein ARV1	ARV1			
Q9GZN1	Actin-related protein 6	ACTR6			
O60423	Phospholipid-transporting ATPase IK	ATP8B3			
Q8IUZ5	5-phosphohydroxy-L-lysine phospho-lyase	PHYKPL			
P56750	Claudin-17	CLDN17			
Q156A1	Ataxin-8	ATXN8			
P56747	Claudin-6	CLDN6			
Q9H7T9	Aurora kinase A and ninein-interacting protein	AUNIP			
O75366	Advillin	AVIL			
Q6ZP68	Putative protein ATP11AUN	ATP11AUN			
P30049	ATP synthase subunit delta, mitochondrial	ATP5F1D			
Q8WTQ4	Uncharacterized protein C16orf78	C16orf78			
Q9UHI8	A disintegrin and metalloproteinase with thrombospondin motifs 1	ADAMTS1			
O60477	BMP/retinoic acid-inducible neural-specific protein 1	BRINP1			
Q13515	Phakinin	BFSP2			

A2RUR9	Coiled-coil domain-containing protein 144A	CCDC144A			
P22004	Bone morphogenetic protein 6	BMP6			
Q5H9B9	Putative BMP-2-inducible kinase-like protein	BMP2KL			
Q96CA5	Baculoviral IAP repeat-containing protein 7	BIRC7			
B2RXH4	BTB/POZ domain-containing protein 18	BTBD18			
P11021	Endoplasmic reticulum chaperone BiP	HSPA5			
O00481	Butyrophilin subfamily 3 member A1	BTN3A1			
Q96Q07	BTB/POZ domain-containing protein 9	BTBD9			
O00478	Butyrophilin subfamily 3 member A3	BTN3A3			
Q8TAM1	Bardet-Biedl syndrome 10 protein	BBS10			
Q9NWK9	Box C/D snoRNA protein 1	ZNHIT6			
Q9BXC9	Bardet-Biedl syndrome 2 protein	BBS2			
Q9NZM4	BRD4-interacting chromatin-remodeling complex-associated protein	BICRA			
P55957	BH3-interacting domain death agonist	BID			
Q9Y5Z0	Beta-secretase 2	BACE2			
Q8TBE0	Bromo adjacent homology domain-containing 1 protein	BAHD1			
Q8IYE0	Coiled-coil domain-containing protein 146	CCDC146			
Q9HBI5	Uncharacterized protein C3orf14	C3orf14			
Q9NXV6	CDKN2A-interacting protein	CDKN2AIP			
Q06432	Voltage-dependent calcium channel gamma-1 subunit	CACNG1			
Q6TFL3	Coiled-coil domain-containing protein 171	CCDC171			
Q99731	C-C motif chemokine 19	CCL19			
Q9P2B7	Cilia- and flagella-associated protein 97	CFAP97			
Q8NDM7	Cilia- and flagella-associated protein 43	CFAP43			
Q9HCU4	Cadherin EGF LAG seven-pass G-type receptor 2	CELSR2			
Q9NRB3	Carbohydrate sulfotransferase 12	CHST12			
P47902	Homeobox protein CDX-1	CDX1			
Q9Y6F8	Testis-specific chromodomain protein Y 1	CDY1			
Q569G3	Uncharacterized protein C5orf47	C5orf47			
P0DN87	Choriogonadotropin subunit beta 7	CGB7			
Q5T035	Putative uncharacterized protein C9orf129	C9orf129			
Q5SZB4	Uncharacterized protein C9orf50	C9orf50			
Q69YH5	Cell division cycle-associated protein 2	CDCA2			
Q8N2Z9	Centromere protein S	CENPS			
Q96H22	Centromere protein N	CENPN			
P07358	Complement component C8 beta chain	C8B			
Q86U02	Putative uncharacterized protein encoded by LINC00596	LINC00596			
Q8N1V8	Uncharacterized protein encoded by LINC01561	LINC01561			
P31146	Coronin-1A	CORO1A			
P13073	Cytochrome c oxidase subunit 4 isoform 1, mitochondrial	COX4I1			
Q14894	Ketimine reductase mu-crystallin	CRYM			
Q9Y5P2	Chondrosarcoma-associated gene 2/3 protein	CSAG2			

P02489	Alpha-crystallin A chain	CRYAA			
O43169	Cytochrome b5 type B	CYB5B			
P29033	Gap junction beta-2 protein	GJB2			
O15194	CTD small phosphatase-like protein	CTDSPL			
P99999	Cytochrome c	CYCS			
O15121	Sphingolipid delta	DEGS1			
Q9NUQ9	CYFIP-related Rac1 interactor B	CYRIB			
Q9NUL7	Probable ATP-dependent RNA helicase DDX28	DDX28			
P20711	Aromatic-L-amino-acid decarboxylase	DDC			
A6NH13	Putative uncharacterized protein DNAJC9-AS1	DNAJC9-AS1			
Q9UJU6	Drebrin-like protein	DBNL			
Q5T6F0	DDB1- and CUL4-associated factor 12	DCAF12			
Q86SG3	Deleted in azoospermia protein 4	DAZ4			
Q13268	Dehydrogenase/reductase SDR family member 2, mitochondrial	DHRS2			
P28068	HLA class II histocompatibility antigen, DM beta chain	HLA-DMB			
Q6ZS02	Putative GED domain-containing protein DNM1P46	DNM1P46			
Q9ULA0	Aspartyl aminopeptidase	DNPEP			
Q5TB30	DEP domain-containing protein 1A	DEPDC1			
Q5VUD6	Divergent protein kinase domain 1B	DIPK1B			
Q9NTK1	Protein DEPP1	DEPP1			
Q9P1A6	Disks large-associated protein 2	DLGAP2			
Q9UBM7	7-dehydrocholesterol reductase	DHCR7			
Q9UF33	Ephrin type-A receptor 6	EPHA6			
P21128	Poly	ENDOU			
O15197	Ephrin type-B receptor 6	EPHB6			
P48546	Gastric inhibitory polypeptide receptor	GIPR			
Q3B7J2	Glucose-fructose oxidoreductase domain-containing protein 2	GFOD2			
Q9UGJ1	Gamma-tubulin complex component 4	TUBGCP4			
Q9P2W3	Guanine nucleotide-binding protein G	GNG13			
P16383	Intron Large complex component GCFC2	GCFC2			
Q16445	Gamma-aminobutyric acid receptor subunit alpha-6	GABRA6			
P51124	Granzyme M	GZMM			
Q8TDV5	Glucose-dependent insulinotropic receptor	GPR119			
Q5VW38	Protein GPR107	GPR107			
O95838	Glucagon-like peptide 2 receptor	GLP2R			
A6NDN3	Golgin subfamily A member 6B	GOLGA6B			
I6L899	Golgin subfamily A member 8R	GOLGA8R			
A8MXD5	Glutaredoxin domain-containing cysteine-rich protein 1	GRXCR1			
Q9BY21	G-protein coupled receptor 87	GPR87			
O15499	Homeobox protein goosecoid-2	GSC2			
P58876	Histone H2B type 1-D	H2BC5			
Q96DB2	Histone deacetylase 11	HDAC11			
Q7Z4H3	5'-deoxynucleotidase HDDC2	HDDC2			
P42261	Glutamate receptor 1	GRIA1			
O96004	Heart- and neural crest derivatives-expressed protein 1	HAND1			
P0C0S5	Histone H2A.Z	H2AZ1			
P22492	Histone H1t	H1-6			
Q5TA89	Transcription factor HES-5	HES5			
Q9GZZ0	Homeobox protein Hox-D1	HOXD1			

P0DP02	Immunoglobulin heavy variable 3-30-3	IGHV3-30-3				
Q00839	Heterogeneous nuclear ribonucleoprotein U	HNRNPU				
P61978	Heterogeneous nuclear ribonucleoprotein K	HNRNPK				
Q7LGA3	Heparan sulfate 2-O-sulfotransferase 1	HS2ST1				
P52926	High mobility group protein HMGI-C	HMGA2				
Q8IX15	Homeobox and leucine zipper protein Homez	HOMEZ				
O14561	Acyl carrier protein, mitochondrial	NDUFAB1				
Q15018	BRISC complex subunit Abraxas 2	ABRAXAS2				
P78363	Retinal-specific phospholipid-transporting ATPase ABCA4	ABCA4				
P01023	Alpha-2-macroglobulin	A2M				
Q9P2A4	ABI gene family member 3	ABI3				
Q9P1F3	Costars family protein ABRACL	ABRACL				
Q5XXA6	Anoctamin-1	ANO1				
Q86SG2	Ankyrin repeat domain-containing protein 23	ANKRD23				
P35414	Apelin receptor	APLNR				
Q8N8A2	Serine/threonine-protein phosphatase 6 regulatory ankyrin repeat subunit B	ANKRD44				
O75077	Disintegrin and metalloproteinase domain-containing protein 23	ADAM23				
Q4KMQ2	Anoctamin-6	ANO6				
P40617	ADP-ribosylation factor-like protein 4A	ARL4A				
P78540	Arginase-2, mitochondrial	ARG2				
O15085	Rho guanine nucleotide exchange factor 11	ARHGEF11				
O76027	Annexin A9	ANXA9				
Q8NEN0	Armadillo repeat-containing protein 2	ARMC2				
A6NM10	Aquaporin-12B	AQP12B				
Q92747	Actin-related protein 2/3 complex subunit 1A	ARPC1A				
O15123	Angiopoietin-2	ANGPT2				
Q9NQW6	Anillin	ANLN				
Q96BM1	Ankyrin repeat domain-containing protein 9	ANKRD9				
Q13625	Apoptosis-stimulating of p53 protein 2	TP53BP2				
Q5T2N8	ATPase family AAA domain-containing protein 3C	ATAD3C				
P12645	Bone morphogenetic protein 3	BMP3				
Q99622	Protein C10	C12orf57				
Q075Z2	Binder of sperm protein homolog 1	BSPH1				
Q9UIR0	Butyrophilin-like protein 2	BTNL2				
Q96IK1	Biorientation of chromosomes in cell division protein 1	BOD1				
Q9Y6W3	Calpain-7	CAPN7				
Q9NYF8	Bcl-2-associated transcription factor 1	BCLAF1				
Q5VWW1	Complement C1q-like protein 3	C1QL3				
P11586	C-1-tetrahydrofolate synthase, cytoplasmic	MTHFD1				
P09871	Complement C1s subcomponent	C1S				
Q96PB1	N-acetylneuraminate 9-O-acetyltransferase	CASD1				
Q0VFZ6	Coiled-coil domain-containing protein 173	CCDC173				
Q8N4J0	Carnosine N-methyltransferase	CARNMT1				
Q6ZP82	Coiled-coil domain-containing protein 141	CCDC141				

Q8WTX7	Cytosolic arginine sensor for mTORC1 subunit 1	CASTOR1			
Q53HC0	Coiled-coil domain-containing protein 92	CCDC92			
Q17RM4	Coiled-coil domain-containing protein 142	CCDC142			
O00175	C-C motif chemokine 24	CCL24			
Q9NRJ3	C-C motif chemokine 28	CCL28			
Q8N0U6	Putative uncharacterized protein encoded by LINC00518	LINC00518			
Q9UKJ5	Cysteine-rich hydrophobic domain-containing protein 2	CHIC2			
P51686	C-C chemokine receptor type 9	CCR9			
Q6P1X6	UPF0598 protein C8orf82	C8orf82			
Q9UHD1	Cysteine and histidine-rich domain-containing protein 1	CHORDC1			
Q86X02	Cerebellar degeneration-related protein 2-like	CDR2L			
Q6ZTY9	Uncharacterized protein C7orf65	C7orf65			
C9J3I9	Putative uncharacterized protein C5orf58	C5orf58			
Q9NS84	Carbohydrate sulfotransferase 7	CHST7			
Q9H3R5	Centromere protein H	CENPH			
Q8IZ52	Chondroitin sulfate synthase 2	CHPF			
Q96FF9	Sororin	CDCA5			
O14735	CDP-diacylglycerol--inositol 3-phosphatidyltransferase	CDIPT			
Q52M58	Putative uncharacterized protein C14orf177	C14orf177			
Q8N5U0	Uncharacterized protein C11orf42	C11orf42			
P12111	Collagen alpha-3	COL6A3			
P54108	Cysteine-rich secretory protein 3	CRISP3			
Q16630	Cleavage and polyadenylation specificity factor subunit 6	CPSF6			
Q9NSK7	Protein C19orf12	C19orf12			
Q96D31	Calcium release-activated calcium channel protein 1	ORAI1			
P0DPH9	Uncharacterized protein CXorf51B	CXorf51B			
Q5T3F8	CSC1-like protein 2	TMEM63B			
P26232	Catenin alpha-2	CTNNA2			
Q9UI47	Catenin alpha-3	CTNNA3			
P25024	C-X-C chemokine receptor type 1	CXCR1			
Q6NXN4	Putative C-mannosyltransferase DPY19L2P1	DPY19L2P1			
O14529	Homeobox protein cut-like 2	CUX2			
Q9BQT9	Calsyntenin-3	CLSTN3			
Q96C57	Protein CUSTOS	CUSTOS			
C9J442	Uncharacterized protein C22orf46	C22orf46			
Q9UJW0	Dynactin subunit 4	DCTN4			
Q8NEP3	Dynein assembly factor 1, axonemal	DNAAF1			
Q9H8H2	Probable ATP-dependent RNA helicase DDX31	DDX31			
Q5QP82	DDB1- and CUL4-associated factor 10	DCAF10			
P11926	Ornithine decarboxylase	ODC1			
Q14565	Meiotic recombination protein DMC1/LIM15 homolog	DMC1			
Q30KQ6	Beta-defensin 114	DEFB114			
O95424	Dexamethasone-induced protein	DEXI			
Q8WZ79	Deoxyribonuclease-2-beta	DNASE2B			
Q9NZQ0	DnaJ homolog subfamily C member 27	DNAJC27			
Q6Y2X3	DnaJ homolog subfamily C member 14	DNAJC14			
Q9NYJ7	Delta-like protein 3	DLL3			
P00367	Glutamate dehydrogenase 1, mitochondrial	GLUD1			

O94777	Dolichol phosphate-mannose biosynthesis regulatory protein	DPM2			
P56555	Down syndrome critical region protein 4	DSCR4			
O95147	Dual specificity protein phosphatase 14	DUSP14			
A6NDY0	Embryonic polyadenylate-binding protein 2	PABPN1L			
P28324	ETS domain-containing protein Elk-4	ELK4			
P21709	Ephrin type-A receptor 1	EPHA1			
P52803	Ephrin-A5	EFNA5			
Q8WXT5	Forkhead box protein D4-like 4	FOXD4L4			
Q8NA03	Fibrous sheath-interacting protein 1	FSIP1			
Q5SYB0	FERM and PDZ domain-containing protein 1	FRMPD1			
Q9NS71	Gastrokine-1	GKN1			
Q92847	Growth hormone secretagogue receptor type 1	GHSR			
Q9NY64	Solute carrier family 2, facilitated glucose transporter member 8	SLC2A8			
Q96QA5	Gasdermin-A	GSDMA			
P0C5Y9	Histone H2A-Bbd type 1	H2AB1			
Q9NQ87	Hairy/enhancer-of-split related with YRPW motif-like protein	HEYL			
Q8WVB3	Hexosaminidase D	HEXD			
P05981	Serine protease hepsin	HPN			
O14964	Hepatocyte growth factor-regulated tyrosine kinase substrate	HGS			
P42694	Probable helicase with zinc finger domain	HELZ			
A0A0C4DH34	Immunoglobulin heavy variable 4-28	IGHV4-28			
A0A0B4J2H0	Immunoglobulin heavy variable 1-69D	IGHV1-69D			
P0DP03	Immunoglobulin heavy variable 3-30-5	IGHV3-30-5			
Q86X24	HORMA domain-containing protein 1	HORMAD1			
Q9P0W0	Interferon kappa	IFNK			
Q15653	NF-kappa-B inhibitor beta	NFKBIB			
Q5VVH5	Interleukin-1 receptor-associated kinase 1-binding protein 1	IRAK1BP1			
Q13422	DNA-binding protein Ikaros	IKZF1			
P0DOX3	Immunoglobulin delta heavy chain				
P01589	Interleukin-2 receptor subunit alpha	IL2RA			
Q9NZH6	Interleukin-37	IL37			
P01859	Immunoglobulin heavy constant gamma 2	IGHG2			
Q9C086	INO80 complex subunit B	INO80B			
Q93033	Immunoglobulin superfamily member 2	CD101			
Q8NFV4	Protein ABHD11	ABHD11			
Q8WTS1	1-acylglycerol-3-phosphate O-acyltransferase ABHD5	ABHD5			
P49418	Amphiphysin	AMPH			
O15013	Rho guanine nucleotide exchange factor 10	ARHGEF10			
Q6NSI1	Putative ankyrin repeat domain-containing protein 26-like protein	ANKRD26P1			
P50553	Achaete-scute homolog 1	ASCL1			
Q9BVC5	Ashwin	C2orf49			
Q96CP6	Protein Aster-A	GRAMD1A			
O14613	Cdc42 effector protein 2	CDC42EP2			
P36894	Bone morphogenetic protein receptor type-1A	BMPR1A			

Q9NP55	BPI fold-containing family A member 1	BPIFA1			
P0C671	Protein BNIP5	BNIP5			
Q14032	Bile acid-CoA:amino acid N-acyltransferase	BAAT			
Q13829	BTB/POZ domain-containing adapter for CUL3-mediated RhoA degradation protein 2	TNFAIP1			
Q9BYV9	Transcription regulator protein BACH2	BACH2			
A8MXE2	Beta-1,3-galactosyltransferase 9	B3GALT9			
Q9C0K0	B-cell lymphoma/leukemia 11B	BCL11B			
Q8WY22	BRI3-binding protein	BRI3BP			
Q9C0J9	Class E basic helix-loop-helix protein 41	BHLHE41			
Q16790	Carbonic anhydrase 9	CA9			
P46379	Large proline-rich protein BAG6	BAG6			
Q96L14	Cep170-like protein	CEP170P1			
P09668	Pro-cathepsin H	CTSH			
Q8N4L8	Coiled-coil domain-containing protein 24	CCDC24			
Q96HQ2	CDKN2AIP N-terminal-like protein	CDKN2AIP NL			
A2RUB6	Coiled-coil domain-containing protein 66	CCDC66			
Q8TF44	C2 calcium-dependent domain-containing protein 4C	C2CD4C			
P55210	Caspase-7	CASP7			
Q9H0I3	Coiled-coil domain-containing protein 113	CCDC113			
Q6ZUT6	Coiled-coil domain-containing protein 9B	CCDC9B			
Q5JTY5	COBW domain-containing protein 3	CBWD3			
Q7LBR1	Charged multivesicular body protein 1b	CHMP1B			
Q8TBY9	Cilia- and flagella-associated protein 251	CFAP251			
P01732	T-cell surface glycoprotein CD8 alpha chain	CD8A			
A6NFT4	Cilia- and flagella-associated protein 73	CFAP73			
P0C854	Putative cat eye syndrome critical region protein 9	CECR9			
Q8N4T0	Carboxypeptidase A6	CPA6			
Q8IX05	CD302 antigen	CD302			
Q2M2E5	Uncharacterized protein C5orf64	C5orf64			
Q92772	Cyclin-dependent kinase-like 2	CDKL2			
Q9BPX7	UPF0415 protein C7orf25	C7orf25			
P48509	CD151 antigen	CD151			
Q99967	Cbp/p300-interacting transactivator 2	CITED2			
Q9NQ89	Protein C12orf4	C12orf4			
Q9Y6N3	Calcium-activated chloride channel regulator family member 3	CLCA3P			
Q92600	CCR4-NOT transcription complex subunit 9	CNOT9			
O95476	CTD nuclear envelope phosphatase 1	CTDNEP1			
P49761	Dual specificity protein kinase	CLK3			
A0PJX0	Calcium and integrin-binding family member 4	CIB4			
F2Z3M2	Uncharacterized protein encoded by LINC02876	LINC02876			
Q6UWE3	Colipase-like protein 2	CLPSL2			
P67870	Casein kinase II subunit beta	CSNK2B			
Q6V0L0	Cytochrome P450 26C1	CYP26C1			

Q9H1R2	Dual specificity protein phosphatase 15	DUSP15			
Q9UHN1	DNA polymerase subunit gamma-2, mitochondrial	POLG2			
O95620	tRNA-dihydrouridine	DUS4L			
Q9P2K8	eIF-2-alpha kinase GCN2	EIF2AK4			
Q8IZS6	Dynein light chain Tctex-type protein 2	DYNLT2			
P13639	Elongation factor 2	EEF2			
Q52LR7	Enhancer of polycomb homolog 2	EPC2			
P54756	Ephrin type-A receptor 5	EPHA5			
A1L3X0	Elongation of very long chain fatty acids protein 7	ELOVL7			
P41214	Eukaryotic translation initiation factor 2D	EIF2D			
Q01469	Fatty acid-binding protein 5	FABP5			
P31994	Low affinity immunoglobulin gamma Fc region receptor II-b	FCGR2B			
O75015	Low affinity immunoglobulin gamma Fc region receptor III-B	FCGR3B			
Q9BVV2	Fibronectin type III domain-containing protein 11	FNDC11			
Q9NZU1	Leucine-rich repeat transmembrane protein FLRT1	FLRT1			
Q8N531	F-box/LRR-repeat protein 6	FBXL6			
P02675	Fibrinogen beta chain	FGB			
Q8WUP2	Filamin-binding LIM protein 1	FBLIM1			
P06744	Glucose-6-phosphate isomerase	GPI			
Q96IV6	Fatty acid hydroxylase domain-containing protein 2	FAXDC2			
P51993	4-galactosyl-N-acetylglucosaminide 3-alpha-L-fucosyltransferase FUT6	FUT6			
P21217	3-galactosyl-N-acetylglucosaminide 4-alpha-L-fucosyltransferase FUT3	FUT3			
P23771	Trans-acting T-cell-specific transcription factor GATA-3	GATA3			
P06241	Tyrosine-protein kinase Fyn	FYN			
Q14332	Frizzled-2	FZD2			
Q9NY12	H/ACA ribonucleoprotein complex subunit 1	GAR1			
P32239	Gastrin/cholecystokinin type B receptor	CCKBR			
P15408	Fos-related antigen 2	FOSL2			
O14926	Fascin-2	FSCN2			
O94923	D-glucuronyl C5-epimerase	GLCE			
O14908	PDZ domain-containing protein GIPC1	GIPC1			
Q9UEU5	G antigen 2D	GAGE2D;			
A6NDE8	G antigen 12H	GAGE12H			
P52565	Rho GDP-dissociation inhibitor 1	ARHGDIA			
P24046	Gamma-aminobutyric acid receptor subunit rho-1	GABRR1			
A8MPY1	Gamma-aminobutyric acid receptor subunit rho-3	GABRR3			
Q99679	Probable G-protein coupled receptor 21	GPR21			
Q5T4B2	Inactive glycosyltransferase 25 family member 3	CERCAM			
Q969Y2	tRNA modification GTPase GTPBP3, mitochondrial	GTPBP3			
Q6MZN7	HLA class I histocompatibility antigen protein P5	HCP5			
P0DPK5	Histone H3.X	H3Y2			
P04183	Thymidine kinase, cytosolic	TK1			
Q6DRA6	Putative histone H2B type 2-D	H2BC19P			
P0DOY5	Immunoglobulin heavy diversity 1-1	IGHD1-1			

Q8IV16	Glycosylphosphatidylinositol-anchored high density lipoprotein-binding protein 1	GPIHBP1			
Q9UBQ7	Glyoxylate reductase/hydroxypyruvate reductase	GRHPR			
Q8TE85	Grainyhead-like protein 3 homolog	GRHL3			
Q9BT25	HAUS augmin-like complex subunit 8	HAUS8			
O00165	HCLS1-associated protein X-1	HAX1			
P62805	Histone H4	H4C1			
Q8TF76	Serine/threonine-protein kinase haspin	HASPIN			
Q9UM44	HERV-H LTR-associating protein 2	HHLA2			
Q96JB3	Hypermethylated in cancer 2 protein	HIC2			
P09630	Homeobox protein Hox-C6	HOXC6			
Q8IVG9	Humanin	MT-RNR2			
P01780	Immunoglobulin heavy variable 3-7	IGHV3-7			
Q14568	Heat shock protein HSP 90-alpha A2	HSP90AA2P			
A0A1B0GTS1	Heat shock transcription factor, X-linked member 4	HSFX4			
A6NHT5	Homeobox protein HMX3	HMX3			
P0DMP1	Humanin-like 12	MTRNR2L12			
P40189	Interleukin-6 receptor subunit beta	IL6ST			
P09912	Interferon alpha-inducible protein 6	IFI6			
Q14774	H2.0-like homeobox protein	HLX			
Q01638	Interleukin-1 receptor-like 1	IL1RL1			
P78552	Interleukin-13 receptor subunit alpha-1	IL13RA1			
Q8NI38	NF-kappa-B inhibitor delta	NFKBID			
Q96MG2	Junctional sarcoplasmic reticulum protein 1	JSRP1			
Q96PY0	Putative uncharacterized protein PSMG3-AS1	PSMG3-AS1			
P06213	Insulin receptor	INSR			
Q8WWG9	Potassium voltage-gated channel subfamily E member 4	KCNE4			
Q6TFL4	Kelch-like protein 24	KLHL24			
Q9Y2U2	Potassium channel subfamily K member 7	KCNK7			
Q13886	Krueppel-like factor 9	KLF9			
Q5VTJ3	Kelch domain-containing protein 7A	KLHDC7A			
Q9UKR3	Kallikrein-13	KLK13			
O60516	Eukaryotic translation initiation factor 4E-binding protein 3	EIF4EBP3			
Q9Y312	Protein AAR2 homolog	AAR2			
P17787	Neuronal acetylcholine receptor subunit beta-2	CHRNB2			
P07510	Acetylcholine receptor subunit gamma	CHRNG			
Q2M3G0	ATP-binding cassette sub-family B member 5	ABCB5			
Q9UGI9	5'-AMP-activated protein kinase subunit gamma-3	PRKAG3			
Q6PD74	Alpha- and gamma-adaptin-binding protein p34	AAGAB			
O43741	5'-AMP-activated protein kinase subunit beta-2	PRKAB2			
Q99218	Amelogenin, Y isoform	AMELY			
P31751	RAC-beta serine/threonine-protein kinase	AKT2			

C9JUS6	Putative adrenomedullin-5-like protein	ADM5			
C9J202	Putative glycosyltransferase ALG1L2	ALG1L2			
Q96L96	Alpha-protein kinase 3	ALPK3			
Q9UEY8	Gamma-adducin	ADD3			
P61163	Alpha-centractin	ACTR1A			
A6NHY2	Ankyrin repeat and death domain-containing protein 1B	ANKDD1B			
A8MUL3	Putative uncharacterized protein ADARB2-AS1	ADARB2-AS1			
O95622	Adenylate cyclase type 5	ADCY5			
Q9Y673	Dolichyl-phosphate beta-glucosyltransferase	ALG5			
Q63HQ0	AP-1 complex-associated regulatory protein	AP1AR			
B4E2M5	Ankyrin repeat domain-containing protein 66	ANKRD66			
P39687	Acidic leucine-rich nuclear phosphoprotein 32 family member A	ANP32A			
Q69YU3	Ankyrin repeat domain-containing protein 34A	ANKRD34A			
O94973	AP-2 complex subunit alpha-2	AP2A2			
A6NH57	Putative ADP-ribosylation factor-like protein 5C	ARL5C			
Q8N1W1	Rho guanine nucleotide exchange factor 28	ARHGEF28			
P53367	Arfaptin-1	ARFIP1			
Q8N6H7	ADP-ribosylation factor GTPase-activating protein 2	ARFGAP2			
Q99062	Granulocyte colony-stimulating factor receptor	CSF3R			
Q99829	Copine-1	CPNE1			
Q6UUV9	CREB-regulated transcription coactivator 1	CRTC1			
Q5IJ48	Protein crumbs homolog 2	CRB2			
O95196	Chondroitin sulfate proteoglycan	CSPG5			
Q16527	Cysteine and glycine-rich protein	CSRP2			
Q05D32	CTD small phosphatase-like protein 2	CTDSPL2			
O94985	Calsyntenin-1	CLSTN1			
Q9H114	Cystatin-like 1	CSTL1			
Q7Z4W1	L-xylulose reductase	DCXR			
Q8IV53	DENN domain-containing protein 1C	DENND1C			
Q9P219	Protein Daple	CCDC88C			
Q86TM3	Probable ATP-dependent RNA helicase DDX53	DDX53			
Q9UK59	Lariat debranching enzyme	DBR1			
O75911	Short-chain dehydrogenase/reductase 3	DHRS3			
Q14126	Desmoglein-2	DSG2			
Q86SJ6	Desmoglein-4	DSG4			
Q02487	Desmocollin-2	DSC2			
P0CJ86	Double homeobox protein 4-like protein 3	DUX4L3			
Q14236	Early lymphoid activation gene protein	DIAPH2-AS1			
Q9H223	EH domain-containing protein 4	EHD4			
P54852	Epithelial membrane protein 3	EMP3			
A6NEL3	Putative protein FAM86C2P	FAM86C2P			
B1AK53	Espin	ESPN			
P0C7W8	Putative protein FAM90A13P	FAM90A13P			
Q92636	Protein FAN	NSMAF			
P10768	S-formylglutathione hydrolase	ESD			
Q86YB8	ERO1-like protein beta	ERO1B			
Q9HB96	Fanconi anemia group E protein	FANCE			

Q9BZJ7	G-protein coupled receptor 62	GPR62			
Q8NB91	Fanconi anemia group B protein	FANCB			
Q96RU3	Formin-binding protein 1	FNBP1			
Q9UIM3	FK506-binding protein-like	FKBPL			
O95302	Peptidyl-prolyl cis-trans isomerase FKBP9	FKBP9			
A8MUU1	Putative fatty acid-binding protein 5-like protein 3	FABP5P3			
O75369	Filamin-B	FLNB			
O75955	Flotillin-1	FLOT1			
Q494R0	Putative uncharacterized protein FBXL19-AS1	FBXL19-AS1			
Q5TD97	Four and a half LIM domains protein 5	FHL5			
Q9HCT0	Fibroblast growth factor 22	FGF22			
P35555	Fibrillin-1	FBN1			
O43826	Glucose-6-phosphate exchanger SLC37A4	SLC37A4			
Q9NQR9	Glucose-6-phosphatase 2	G6PC2			
Q96A11	Galactose-3-O-sulfotransferase 3	GAL3ST3			
O95479	GDH/6PGL endoplasmic bifunctional protein	H6PD			
A0A0B4J2D5	Glutamine amidotransferase-like class 1 domain-containing protein 3B, mitochondrial	GATD3B			
P55317	Hepatocyte nuclear factor 3-alpha	FOXA1			
Q7L622	G2/M phase-specific E3 ubiquitin-protein ligase	G2E3			
Q08050	Forkhead box protein M1	FOXM1			
Q86SF2	N-acetylgalactosaminyltransferase 7	GALNT7			
Q9H0R6	Glutamyl-tRNA	QRSL1			
Q68DX3	FERM and PDZ domain-containing protein 2	FRMPD2			
P98177	Forkhead box protein O4	FOXO4			
Q9NXP7	Gypsy retrotransposon integrase-like protein 1	GIN1			
A6NEM1	Golgin subfamily A member 6-like protein 9	GOLGA6L9			
P23434	Glycine cleavage system H protein, mitochondrial	GCSH			
P52566	Rho GDP-dissociation inhibitor 2	ARHGDIB			
Q9NWZ8	Gem-associated protein 8	GEMIN8			
Q9NVN8	Guanine nucleotide-binding protein-like 3-like protein	GNL3L			
O15488	Glycogenin-2	GYG2			
P21695	Glycerol-3-phosphate dehydrogenase	GPD1			
P15421	Glycophorin-E	GYPE			
Q7Z3D6	D-glutamate cyclase, mitochondrial	DGLUCY			
P15104	Glutamine synthetase	GLUL			
P69891	Hemoglobin subunit gamma-1	HBG1			
P23527	Histone H2B type 1-O	H2BC17			
Q14832	Metabotropic glutamate receptor 3	GRM3			
P16104	Histone H2AX	H2AX			
Q9NYQ3	Hydroxyacid oxidase 2	HAO2			
P26927	Hepatocyte growth factor-like protein	MST1			
Q03014	Hematopoietically-expressed homeobox protein HHEX	HHEX			
Q6NVY1	3-hydroxyisobutyryl-CoA hydrolase, mitochondrial	HIBCH			
Q96NU7	Probable imidazolonepropionase	AMDHD1			
A0A0C4DH39	Immunoglobulin heavy variable 1-58	IGHV1-58			
Q96FZ2	Abasic site processing protein HMCES	HMCES			

P0DTE2	Probable non-functional immunoglobulin heavy variable 8-51-1	IGHV8-51-1			
P37235	Hippocalcin-like protein 1	HPCAL1			
Q9Y663	Heparan sulfate glucosamine 3-O-sulfotransferase 3A1	HS3ST3A1			
P35367	Histamine H1 receptor	HRH1			
P30511	HLA class I histocompatibility antigen, alpha chain F	HLA-F			
Q9H6K1	Protein ILRUN	ILRUN			
Q9P2D0	Inhibitor of Bruton tyrosine kinase	IBTK			
P0DOX5	Immunoglobulin gamma-1 heavy chain				
Q86VF2	Immunoglobulin-like and fibronectin type III domain-containing protein 1	IGFN1			
P06730	Eukaryotic translation initiation factor 4E	EIF4E			
Q8NI99	Angiopoietin-related protein 6	ANGPTL6			
Q07973	1,25-dihydroxyvitamin D	CYP24A1			
P23528	Cofilin-1	CFL1			
Q8WXC6	COP9 signalosome complex subunit 9	COPS9			
I3L1E1	Uncharacterized protein C19orf84	C19orf84			
Q9Y534	Cold shock domain-containing protein C2	CSDC2			
Q86T23	Putative ciliary rootlet coiled-coil protein-like 1 protein	CROCCP2			
P0DMU9	Cancer/testis antigen family 45 member A10	CT45A10			
Q53ET0	CREB-regulated transcription coactivator 2	CRTC2			
P16410	Cytotoxic T-lymphocyte protein 4	CTLA4			
Q9NWV4	CXXC motif containing zinc binding protein	CZIB			
Q9NXE8	Pre-mRNA-splicing factor CWC25 homolog	CWC25			
P04080	Cystatin-B	CSTB			
Q9NTU4	Cation channel sperm-associated protein subunit zeta	CATSPERZ			
P08574	Cytochrome c1, heme protein, mitochondrial	CYC1			
Q96LI9	Putative uncharacterized protein CXorf58	CXorf58			
A4D1U4	DENN domain-containing protein 11	DENND11			
Q9HBH1	Peptide deformylase, mitochondrial	PDF			
Q9BQY9	Dysbindin domain-containing protein 2	DBNDD2			
Q9H6R0	ATP-dependent RNA helicase DHX33	DHX33			
Q14183	Double C2-like domain-containing protein alpha	DOC2A			
P59910	DnaJ homolog subfamily B member 13	DNAJB13			
Q53GQ0	Very-long-chain 3-oxoacyl-CoA reductase	HSD17B12			
Q9Y6W6	Dual specificity protein phosphatase 10	DUSP10			
Q9BY84	Dual specificity protein phosphatase 16	DUSP16			
Q9H596	Dual specificity protein phosphatase 21	DUSP21			
O60941	Dystrobrevin beta	DTNB			
Q8N5C7	tRNA-uridine aminocarboxypropyltransferase 1	DTWD1			
P54792	Putative segment polarity protein dishevelled homolog DVL1P1	DVL1P1			

Q05193	Dynamin-1	DNM1			
P0C7U0	Protein ELFN1	ELFN1			
P58107	Epiplakin	EPPK1			
Q13491	Neuronal membrane glycoprotein M6-b	GPM6B			
P48167	Glycine receptor subunit beta	GLRB			
Q96RP9	Elongation factor G, mitochondrial	GFM1			
Q5JVL4	EF-hand domain-containing protein 1	EFHC1			
P60228	Eukaryotic translation initiation factor 3 subunit E	EIF3E			
Q8N3D4	EH domain-binding protein 1-like protein 1	EHBP1L1			
P60509	Endogenous retrovirus group PABLB member 1 Env polyprotein	ERVPABLB-1			
P33947	ER lumen protein-retaining receptor 2	KDELR2			
Q96RT1	Erbin	ERBIN			
O00519	Fatty-acid amide hydrolase 1	FAAH			
Q8TAM6	Ermin	ERMN			
A6NFZ4	Protein FAM24A	FAM24A			
Q9NYY8	FAST kinase domain-containing protein 2, mitochondrial	FASTKD2			
P46093	G-protein coupled receptor 4	GPR4			
Q5JUQ0	Protein FAM78A	FAM78A			
Q5W0V3	Protein FAM160B1	FAM160B1			
O15287	Fanconi anemia group G protein	FANCG			
Q8NAU1	Fibronectin type III domain-containing protein 5	FNDC5			
Q9UKV0	Histone deacetylase 9	HDAC9			
Q9NZ56	Formin-2	FMN2			
Q96SL8	Flt3-interacting zinc finger protein 1	FIZ1			
Q96EF6	F-box only protein 17	FBXO17			
Q9NRD0	F-box only protein 8	FBXO8			
Q5FWF7	F-box only protein 48	FBXO48			
B3EWG5	Protein FAM25C	FAM25C			
Q5JSP0	FYVE, RhoGEF and PH domain-containing protein 3	FGD3			
Q8TBJ5	Fez family zinc finger protein 2	FEZF2			
P49327	Fatty acid synthase	FASN			
Q96A29	GDP-fucose transporter 1	SLC35C1			
Q64ET8	Protein FRG2	FRG2			
Q9H0Q3	FXYD domain-containing ion transport regulator 6	FXYD6			
Q14802	FXYD domain-containing ion transport regulator 3	FXYD3			
Q6ZQY3	Acidic amino acid decarboxylase GADL1	GADL1			
Q6MZW2	Follistatin-related protein 4	FSTL4			
Q4V321	G antigen 13	GAGE13			
Q9ULW2	Frizzled-10	FZD10			
A8MTJ6	Forkhead box protein I3	FOXI3			
O43903	Growth arrest-specific protein 2	GAS2			
Q04609	Glutamate carboxypeptidase 2	FOLH1			
Q9NWU2	Glucose-induced degradation protein 8 homolog	GID8			
Q9UG22	GTPase IMAP family member 2	GIMAP2			
Q5VSY0	G kinase-anchoring protein 1	GKAP1			
Q5H9T9	Fibrous sheath CABYR-binding protein	FSCB			
Q96RT7	Gamma-tubulin complex component 6	TUBGCP6			
Q9BY60	Gamma-aminobutyric acid receptor-associated protein-like 3	GABARAPL3			
Q96PP8	Guanylate-binding protein 5	GBP5			
P61952	Guanine nucleotide-binding protein G	GNG11			

P30679	Guanine nucleotide-binding protein subunit alpha-15	GNA15			
Q8NEA9	Germ cell-less protein-like 2	GMCL2			
O14653	Golgi SNAP receptor complex member 2	GOSR2			
Q8IXQ4	GPALPP motifs-containing protein 1	GPALPP1			
O00155	Probable G-protein coupled receptor 25	GPR25			
Q9HC97	G-protein coupled receptor 35	GPR35			
O95843	Guanylyl cyclase-activating protein 3	GUCA1C			
P51841	Retinal guanylyl cyclase 2	GUCY2F			
A8MUP6	Germ cell-specific gene 1-like protein 2	GSG1L2			
Q8NEC7	Glutathione S-transferase C-terminal domain-containing protein	GSTCD			
P06899	Histone H2B type 1-J	H2BC11			
P07305	Histone H1.0	H1-0			
P30711	Glutathione S-transferase theta-1	GSTT1			
Q99880	Histone H2B type 1-L	H2BC13			
B7U540	Inward rectifier potassium channel 18	KCNJ18			
Q96KK5	Histone H2A type 1-H	H2AC12			
O60269	G protein-regulated inducer of neurite outgrowth 2	GPRIN2			
P0C5Z0	Histone H2A-Bbd type 2/3	H2AB2			
Q96MM7	Heparan-sulfate 6-O-sulfotransferase 2	HS6ST2			
Q9BQA5	Histone H4 transcription factor	HINFP			
P06865	Beta-hexosaminidase subunit alpha	HEXA			
Q9Y5Z7	Host cell factor 2	HCFC2			
Q15011	Homocysteine-responsive endoplasmic reticulum-resident ubiquitin-like domain member 1 protein	HERPUD1			
Q9NRZ9	Lymphoid-specific helicase	HELLS			
P31268	Homeobox protein Hox-A7	HOXA7			
Q9BUP3	Oxidoreductase HTATIP2	HTATIP2			
P01766	Immunoglobulin heavy variable 3-13	IGHV3-13			
A0A0C4DH43	Immunoglobulin heavy variable 2-70D	IGHV2-70D			
A0A075B6Q5	Immunoglobulin heavy variable 3-64	IGHV3-64			
A0A0C4DH41	Immunoglobulin heavy variable 4-61	IGHV4-61			
P06331	Immunoglobulin heavy variable 4-34	IGHV4-34			
Q9H3N8	Histamine H4 receptor	HRH4			
Q6YN16	Hydroxysteroid dehydrogenase-like protein 2	HSDL2			
P04196	Histidine-rich glycoprotein	HRG			
Q9GZV4	Eukaryotic translation initiation factor 5A-2	EIF5A2			
P0CJ76	Humanin-like 9	MTRNR2L9			
P0DKL9	ARL14 effector protein-like	ARL14EPL			
Q676U5	Autophagy-related protein 16-1	ATG16L1			
Q12979	Active breakpoint cluster region-related protein	ABR			
Q8N961	Ankyrin repeat and BTB/POZ domain-containing protein 2	ABTB2			
Q9NRW3	DNA dC->dU-editing enzyme APOBEC-3C	APOBEC3C			
P35318	Pro-adrenomedullin	ADM			
Q5I7T1	Putative Dol-P-Glc:Glc	ALG10B			
O00170	AH receptor-interacting protein	AIP			

O95994	Anterior gradient protein 2 homolog	AGR2			
Q6NUN0	Acyl-coenzyme A synthetase ACSM5, mitochondrial	ACSM5			
P51828	Adenylate cyclase type 7	ADCY7			
P35348	Alpha-1A adrenergic receptor	ADRA1A			
Q9UKJ8	Disintegrin and metalloproteinase domain-containing protein 21	ADAM21			
Q96IZ2	Androgen-dependent TFPI-regulating protein	ADTRP			
Q9HAR2	Adhesion G protein-coupled receptor L3	ADGRL3			
P02654	Apolipoprotein C-I	APOC1			
Q2TAZ0	Autophagy-related protein 2 homolog A	ATG2A			
Q8WVV5	Butyrophilin subfamily 2 member A2	BTN2A2			
Q96K17	Transcription factor BTF3 homolog 4	BTF3L4			
Q6L9W6	Beta-1,4-N-acetylgalactosaminyltransferase 3	B4GALNT3			
Q8NDB2	B-cell scaffold protein with ankyrin repeats	BANK1			
Q07812	Apoptosis regulator BAX	BAX			
Q8N8R5	UPF0565 protein C2orf69	C2orf69			
Q9BV19	Uncharacterized protein C1orf50	C1orf50			
P54284	Voltage-dependent L-type calcium channel subunit beta-3	CACNB3			
Q8WUQ7	Cactin	CACTIN			
Q53S99	Folate transporter-like protein C2orf83	C2orf83			
Q9Y426	C2 domain-containing protein 2	C2CD2			
Q8IWA6	Coiled-coil domain-containing protein 60	CCDC60			
A0A1B0GTZ2	Putative coiled-coil domain-containing protein 196	CCDC196			
Q9Y4X3	C-C motif chemokine 27	CCL27			
P0C7W6	Coiled-coil domain-containing protein 172	CCDC172			
Q13191	E3 ubiquitin-protein ligase CBL-B	CBLB			
Q8N7E2	E3 ubiquitin-protein ligase CBLL2	CBLL2			
Q9UF02	Voltage-dependent calcium channel gamma-5 subunit	CACNG5			
Q01151	CD83 antigen	CD83			
P33681	T-lymphocyte activation antigen CD80	CD80			
Q6ZU80	Centrosomal protein of 128 kDa	CEP128			
Q9BSQ5	Cerebral cavernous malformations 2 protein	CCM2			
Q9Y6A4	Cilia- and flagella-associated protein 20	CFAP20			
Q96M91	Cilia- and flagella-associated protein 53	CFAP53			
Q8N7Q2	Putative uncharacterized protein CELF2-AS1	CELF2-AS1			
Q9C0B2	Cilia- and flagella-associated protein 74	CFAP74			
Q96KX1	Uncharacterized protein C4orf36	C4orf36			
Q8TAP6	Centrosomal protein of 76 kDa	CEP76			
P20273	B-cell receptor CD22	CD22			
P51946	Cyclin-H	CCNH			
Q8N960	Centrosomal protein of 120 kDa	CEP120			
Q7LGC8	Carbohydrate sulfotransferase 3	CHST3			
Q9H5V8	CUB domain-containing protein 1	CDCP1			
Q8TCT0	Ceramide kinase	CERK			
O43916	Carbohydrate sulfotransferase 1	CHST1			
Q15131	Cyclin-dependent kinase 10	CDK10			
Q02224	Centromere-associated protein E	CENPE			
P22674	Cyclin-O	CCNO			

Q6UWU4	Bombesin receptor-activated protein C6orf89	C6orf89			
Q9H9A5	CCR4-NOT transcription complex subunit 10	CNOT10			
Q9P232	Contactin-3	CNTN3			
Q9GZY4	Cytochrome c oxidase assembly factor 1 homolog	COA1			
Q6ZS10	C-type lectin domain family 17, member A	CLEC17A			
A5D8T8	C-type lectin domain family 18 member A	CLEC18A			
Q99439	Calponin-2	CNN2			
P05997	Collagen alpha-2	COL5A2			
O75390	Citrate synthase, mitochondrial	CS			
Q8TAZ6	CKLF-like MARVEL transmembrane domain-containing protein 2	CMTM2			
A8MQB3	Putative uncharacterized protein LINC02693	LINC02693			
Q8WVH0	Complexin-3	CPLX3			
Q96B23	Uncharacterized protein C18orf25	C18orf25			
Q8N118	Cytochrome P450 4X1	CYP4X1			
P78524	DENN domain-containing protein 2B	DENND2B			
Q8IWF6	Protein DENND6A	DENND6A			
Q9GZR7	ATP-dependent RNA helicase DDX24	DDX24			
Q96FC9	ATP-dependent DNA helicase DDX11	DDX11			
Q9P1J3	Putative uncharacterized protein DHRS4-AS1	DHRS4-AS1			
Q9NP97	Dynein light chain roadblock-type 1	DYNLRB1			
Q8N4W6	DnaJ homolog subfamily C member 22	DNAJC22			
Q9NX36	DnaJ homolog subfamily C member 28	DNAJC28			
P56178	Homeobox protein DLX-5	DLX5			
Q96F81	Protein dispatched homolog 1	DISP1			
P0DPQ6	DDIT3 upstream open reading frame protein	DDIT3			
Q96PT4	Putative double homeobox protein 3	DUX3			
Q07864	DNA polymerase epsilon catalytic subunit A	POLE			
Q99607	ETS-related transcription factor Elf-4	ELF4			
P29320	Ephrin type-A receptor 3	EPHA3			
Q6UW88	Epigen	EPGN			
P54760	Ephrin type-B receptor 4	EPHB4			
Q5MY95	Ectonucleoside triphosphate diphosphohydrolase 8	ENTPD8			
Q7Z2Z2	Elongation factor-like GTPase 1	EFL1			
Q6IN97	Putative protein FRMPD2-like	FRMPD2B			
P04406	Glyceraldehyde-3-phosphate dehydrogenase	GAPDH			
Q9ULV1	Frizzled-4	FZD4			
Q75VX8	GRB2-associated and regulator of MAPK protein 2	GAREM2			
Q14435	Polypeptide N-acetylgalactosaminyltransferase 3	GALNT3			
P14136	Glial fibrillary acidic protein	GFAP			
P27539	Embryonic growth/differentiation factor 1	GDF1			
Q14397	Glucokinase regulatory protein	GCKR			
Q8NBF1	Zinc finger protein GLIS1	GLIS1			
Q5U4N7	Protein GDF5-AS1, mitochondrial	GDF5-AS1			
Q9HAV0	Guanine nucleotide-binding protein subunit beta-4	GNB4			

P34897	Serine hydroxymethyltransferase, mitochondrial	SHMT2			
P28676	Grancalcin	GCA			
Q08379	Golgin subfamily A member 2	GOLGA2			
Q9BQQ3	Golgi reassembly-stacking protein 1	GORASP1			
A6NCC3	Golgin subfamily A member 8O	GOLGA8O			
A8MTJ3	Guanine nucleotide-binding protein G	GNAT3			
Q6PRD1	Probable G-protein coupled receptor 179	GPR179			
Q9HCN4	GPN-loop GTPase 1	GPN1			
P84243	Histone H3.3	H3-3A			
Q02747	Guanylin	GUCA2A			
Q6L8H2	Keratin-associated protein 5-3	KRTAP5-3			
Q6XYB7	Transcription factor LBX2	LBX2			
P01772	Immunoglobulin heavy variable 3-33	IGHV3-33			
Q9Y305	Acyl-coenzyme A thioesterase 9, mitochondrial	ACOT9			
P21399	Cytoplasmic aconitate hydratase	ACO1			
Q99798	Aconitate hydratase, mitochondrial	ACO2			
Q04844	Acetylcholine receptor subunit epsilon	CHRNE			
Q8N0Z2	Actin-binding Rho-activating protein	ABRA			
P28335	5-hydroxytryptamine receptor 2C	HTR2C			
U3KPV4	Alpha-1,3-galactosyltransferase 2	A3GALT2			
Q96P47	Arf-GAP with GTPase, ANK repeat and PH domain-containing protein 3	AGAP3			
Q9Y2J4	Angiomotin-like protein 2	AMOTL2			
P59780	AP-3 complex subunit sigma-2	AP3S2			
Q9NPF8	Arf-GAP with dual PH domain-containing protein 2	ADAP2			
Q86SQ3	Putative adhesion G protein-coupled receptor E4P	ADGRE4P			
Q96N21	AP-4 complex accessory subunit Tepsin	TEPSIN			
Q9H6U8	Alpha-1,2-mannosyltransferase ALG9	ALG9			
Q9H1A4	Anaphase-promoting complex subunit 1	ANAPC1			
Q9P0K1	Disintegrin and metalloproteinase domain-containing protein 22	ADAM22			
A6NCL7	Ankyrin repeat domain-containing protein 33B	ANKRD33B			
A2A2Z9	Ankyrin repeat domain-containing protein 18B	ANKRD18B			
O15033	Apoptosis-resistant E3 ubiquitin protein ligase 1	AREL1			
A1IGU5	Rho guanine nucleotide exchange factor 37	ARHGEF37			
P01160	Natriuretic peptides A	NPPA			
Q7Z449	Cytochrome P450 2U1	CYP2U1			
Q9H4W6	Transcription factor COE3	EBF3			
P22792	Carboxypeptidase N subunit 2	CPN2			
Q9BQW3	Transcription factor COE4	EBF4			
Q8TBR5	Putative uncharacterized protein CIRBP-AS1	CIRBP-AS1			
Q9UBG3	Cornulin	CRNN			
A6NEN9	Uncharacterized protein CXorf65	CXorf65			
P48165	Gap junction alpha-8 protein	GJA8			
Q6PEY0	Gap junction beta-7 protein	GJB7			
Q53GD3	Choline transporter-like protein 4	SLC44A4			
A6NGU7	Putative uncharacterized protein encoded by LINC01546	LINC01546			

Q9HCG8	Pre-mRNA-splicing factor CWC22 homolog	CWC22			
Q7L9B9	Endonuclease/exonuclease/phosphatase family domain-containing protein 1	EEPD1			
O60469	Down syndrome cell adhesion molecule	DSCAM			
O94919	Endonuclease domain-containing 1 protein	ENDOD1			
P16452	Protein 4.2	EPB42			
P0DPD7	EEF1A lysine methyltransferase 4	EEF1AKMT			
P52799	Ephrin-B2	EFNB2			
Q96DF8	Splicing factor ESS-2 homolog	ESS2			
Q15768	Ephrin-B3	EFNB3			
Q9NQ30	Endothelial cell-specific molecule	ESM1			
D6RGX4	Putative protein FAM90A26	FAM90A26			
Q9NZY2	Putative uncharacterized protein FAM30A	FAM30A			
O75460	Serine/threonine-protein kinase/endoribonuclease IRE1	ERN1			
Q14289	Protein-tyrosine kinase 2-beta	PTK2B			
Q53R41	FAST kinase domain-containing protein 1, mitochondrial	FASTKD1			
Q6ZT52	Protein FAM43B	FAM43B			
Q6ZN32	ETS translocation variant 3-like protein	ETV3L			
Q9Y5Y3	Probable G-protein coupled receptor 45	GPR45			
Q14CZ7	FAST kinase domain-containing protein 3, mitochondrial	FASTKD3			
Q92817	Envoplakin	EVPL			
Q8IZT9	Protein FAM9C	FAM9C			
Q96KP1	Exocyst complex component 2	EXOC2			
Q9NPI8	Fanconi anemia group F protein	FANCF			
O00471	Exocyst complex component 5	EXOC5			
Q8NFI4	Putative protein FAM10A5	ST13P5			
Q9NRY5	Protein FAM114A2	FAM114A2			
Q9BQ89	Protein FAM110A	FAM110A			
P46095	G-protein coupled receptor 6	GPR6			
Q13868	Exosome complex component RRP4	EXOSC2			
Q5T036	Uncharacterized protein FAM120AOS	FAM120AOS			
A6NHQ4	Elongin BC and Polycomb repressive complex 2-associated protein	EPOP			
Q86V42	Protein FAM124A	FAM124A			
Q96B26	Exosome complex component RRP43	EXOSC8			
Q6UXP7	Protein FAM151B	FAM151B			
Q9NSI2	Protein FAM207A	FAM207A			
Q06265	Exosome complex component RRP45	EXOSC9			
P0C875	Protein FAM228B	FAM228B			
Q6J272	Protein FAM166A	FAM166A			
Q16877	6-phosphofructo-2-kinase/fructose-2,6-bisphosphatase 4	PFKFB4			
A0A1B0GVK7	Protein FAM240A	FAM240A			
Q99502	Eyes absent homolog 1	EYA1			
Q9Y324	rRNA-processing protein FCF1 homolog	FCF1			
Q01740	Dimethylaniline monooxygenase	FMO1			
Q7L513	Fc receptor-like A	FCRLA			
Q6PIJ6	F-box only protein 38	FBXO38			
Q8TB52	F-box only protein 30	FBXO30			
B3EWG6	Protein FAM25G	FAM25G			
Q9BSK4	Protein fem-1 homolog A	FEM1A			
Q9UKA1	F-box/LRR-repeat protein 5	FBXL5			

Q10981	Galactoside alpha-	FUT2			
P19526	Galactoside alpha-	FUT1			
Q6UWH4	Golgi-associated kinase 1B	GASK1B			
Q96DB9	FXYD domain-containing ion transport regulator 5	FXYD5			
O43716	Glutamyl-tRNA	GATC			
Q4V326	G antigen 2E	GAGE2E			
P25090	N-formyl peptide receptor 2	FPR2			
P30793	GTP cyclohydrolase 1	GCH1			
P0DPK2	Histone H3.Y	H3Y1			
O60381	HMG box-containing protein 1	HBP1			
Q06136	3-ketodihydrosphingosine reductase	KDSR			
Q14520	Hyaluronan-binding protein 2	HABP2			
P51858	Hepatoma-derived growth factor	HDGF			
Q96QV6	Histone H2A type 1-A	H2AC1			
Q8TDQ0	Hepatitis A virus cellular receptor	HAVCR2			
Q68CZ6	HAUS augmin-like complex subunit 3	HAUS3			
O60229	Kalirin	KALRN			
Q9H422	Homeodomain-interacting protein kinase 3	HIPK3			
Q6UWX4	HHIP-like protein 2	HHIPL2			
Q96JK4	HHIP-like protein 1	HHIPL1			
Q9Y5R4	MTRF1L release factor glutamine methyltransferase	HEMK1			
Q9UM19	Hippocalcin-like protein 4	HPCAL4			
Q9Y662	Heparan sulfate glucosamine 3-O-sulfotransferase 3B1	HS3ST3B1			
Q86WN2	Interferon epsilon	IFNE			
P16871	Interleukin-7 receptor subunit alpha	IL7R			
O14879	Interferon-induced protein with tetratricopeptide repeats 3	IFIT3			
Q14116	Interleukin-18	IL18			
P80217	Interferon-induced 35 kDa protein	IFI35			
P14778	Interleukin-1 receptor type 1	IL1R1			
Q9NZH7	Interleukin-36 beta	IL36B			
Q17R60	Interphotoreceptor matrix proteoglycan 1	IMPG1			
Q5VWK5	Interleukin-23 receptor	IL23R			
P0CG04	Immunoglobulin lambda constant 1	IGLC1			
Q9UBH0	Interleukin-36 receptor antagonist protein	IL36RN			
P12268	Inosine-5'-monophosphate dehydrogenase 2	IMPDH2			
Q8N6P7	Interleukin-22 receptor subunit alpha-1	IL22RA1			
Q14197	Peptidyl-tRNA hydrolase ICT1, mitochondrial	MRPL58			
Q02535	DNA-binding protein inhibitor ID-3	ID3			
P35475	Alpha-L-iduronidase	IDUA			
P24592	Insulin-like growth factor-binding protein 6	IGFBP6			
P31273	Homeobox protein Hox-C8	HOXC8			
Q9HBG6	Intraflagellar transport protein 122 homolog	IFT122			
P50213	Isocitrate dehydrogenase	IDH3A			
Q92551	Inositol hexakisphosphate kinase 1	IP6K1			
Q96PC2	Inositol hexakisphosphate kinase 3	IP6K3			
Q6ZU52	Uncharacterized protein KIAA0408	KIAA0408			
Q8N9B5	Junction-mediating and -regulatory protein	JMY			
Q15040	Josephin-1	JOSD1			
Q15067	Peroxisomal acyl-coenzyme A oxidase 1	ACOX1			
O96019	Actin-like protein 6A	ACTL6A			

Q96SE0	Protein ABHD1	ABHD1			
P08758	Annexin A5	ANXA5			
O43186	Cone-rod homeobox protein	CRX			
Q10570	Cleavage and polyadenylation specificity factor subunit 1	CPSF1			
Q9BUF7	Protein crumbs homolog 3	CRB3			
P0C5K7	Cancer/testis antigen 62	CT62			
A6NIU2	Putative uncharacterized protein encoded by LINC01549	LINC01549			
P78310	Coxsackievirus and adenovirus receptor	CXADR			
P36382	Gap junction alpha-5 protein	GJA5			
O60931	Cystinosin	CTNS			
Q8WY07	Cationic amino acid transporter 3	SLC7A3			
P52569	Cationic amino acid transporter 2	SLC7A2			
Q5T442	Gap junction gamma-2 protein	GJC2			
Q8N4M1	Choline transporter-like protein 3	SLC44A3			
P21291	Cysteine and glycine-rich protein	CSRP1			
Q96S65	Cysteine/serine-rich nuclear protein 1	CSRNP1			
Q96CG8	Collagen triple helix repeat-containing protein 1	CTHRC1			
P0CZ25	Uncharacterized protein DNAH10OS	DNAH10OS			
P08174	Complement decay-accelerating factor	CD55			
A0PJW8	Death-associated protein-like 1	DAPL1			
Q6P3S1	DENN domain-containing protein 1B	DENND1B			
Q8TCE6	DENN domain-containing protein 10	DENND10			
Q8N8Z6	Discoidin, CUB and LCCL domain-containing protein 1	DCBLD1			
Q08345	Epithelial discoidin domain-containing receptor 1	DDR1			
Q7L2E3	ATP-dependent RNA helicase DHX30	DHX30			
Q96MA1	Doublesex- and mab-3-related transcription factor B1	DMRTB1			
Q8WXX5	DnaJ homolog subfamily C member 9	DNAJC9			
O60610	Protein diaphanous homolog 1	DIAPH1			
Q96BY6	Dedicator of cytokinesis protein	DOCK10			
Q9Y238	Deleted in lung and esophageal cancer protein 1	DLEC1			
Q9UNQ2	Probable dimethyladenosine transferase	DIMT1			
Q6P3W2	DnaJ homolog subfamily C member 24	DNAJC24			
Q9UBU2	Dickkopf-related protein 2	DKK2			
Q96GE9	Distal membrane-arm assembly complex protein 1	DMAC1			
P0CJ87	Double homeobox protein 4-like protein 4	DUX4L4			
A0A1W2PPF3	Double homeobox protein B	DUXB			
P13929	Beta-enolase	ENO3			
Q9Y5Q0	Fatty acid desaturase 3	FADS3			
Q8N9I5	Fatty acid desaturase 6	FADS6			
Q13216	DNA excision repair protein ERCC-8	ERCC8			
Q96KD3	Protein FAM71F1	FAM71F1			
Q96RQ1	Endoplasmic reticulum-Golgi intermediate compartment protein 2	ERGIC2			
Q8N9W8	Protein FAM71D	FAM71D			
P98173	Protein FAM3A	FAM3A			
Q9UK22	F-box only protein 2	FBXO2			

Q14512	Fibroblast growth factor-binding protein 1	FGFBP1			
Q9GZV9	Fibroblast growth factor 23	FGF23			
Q9NSA1	Fibroblast growth factor 21	FGF21			
A0PJY2	Fez family zinc finger protein 1	FEZF1			
P10767	Fibroblast growth factor 6	FGF6			
Q9BXR6	Complement factor H-related protein 5	CFHR5			
Q8TAK5	GA-binding protein subunit beta-2	GABPB2			
P35637	RNA-binding protein FUS	FUS			
O75293	Growth arrest and DNA damage-inducible protein GADD45 beta	GADD45B			
Q9Y2I7	1-phosphatidylinositol 3-phosphate 5-kinase	PIKFYVE			
Q9H461	Frizzled-8	FZD8			
O00409	Forkhead box protein N3	FOXN3			
O94915	Protein furry homolog-like	FRYL			
Q9BZS1	Forkhead box protein P3	FOXP3			
Q16658	Fascin	FSCN1			
P34059	N-acetylgalactosamine-6-sulfatase	GALNS			
O60318	Germinal-center associated nuclear protein	MCM3AP			
Q10471	Polypeptide N-acetylgalactosaminyltransferase 2	GALNT2			
Q9H2C0	Gigaxonin	GAN			
Q9H3K2	Growth hormone-inducible transmembrane protein	GHITM			
H0YKK7	Putative golgin subfamily A member 6-like protein 19	GOLGA6L19			
P55040	GTP-binding protein GEM	GEM			
P63218	Guanine nucleotide-binding protein G	GNG5			
Q9HCC8	Glycerophosphoinositol inositolphosphodiesterase GDPD2	GDPD2			
P39905	Glial cell line-derived neurotrophic factor	GDNF			
Q9H8Y8	Golgi reassembly-stacking protein 2	GORASP2			
Q14344	Guanine nucleotide-binding protein subunit alpha-13	GNA13			
Q9H9Y4	GPN-loop GTPase 2	GPN2			
O75388	Probable G-protein coupled receptor 32	GPR32			
Q5JQS6	Germinal center-associated signaling and motility-like protein	GCSAML			
A4D1E9	GTP-binding protein 10	GTPBP10			
P13807	Glycogen	GYS1			
Q5JVS0	Intracellular hyaluronan-binding protein 4	HABP4			
Q9BX68	Histidine triad nucleotide-binding protein 2, mitochondrial	HINT2			
Q9UBX0	Homeobox expressed in ES cells 1	HESX1			
Q969F9	Hermansky-Pudlak syndrome 3 protein	HPS3			
P31277	Homeobox protein Hox-D11	HOXD11			
P14653	Homeobox protein Hox-B1	HOXB1			
P31276	Homeobox protein Hox-C13	HOXC13			
A0A0J9YX35	Immunoglobulin heavy variable 3-64D	IGHV3-64D			
O14792	Heparan sulfate glucosamine 3-O-sulfotransferase 1	HS3ST1			
Q96JZ2	Hematopoietic SH2 domain-containing protein	HSH2D			
Q9BPY8	Homeodomain-only protein	HOPX			
P04035	3-hydroxy-3-methylglutaryl-coenzyme A reductase	HMGCR			
P01571	Interferon alpha-17	IFNA17			

P01568	Interferon alpha-21	IFNA21			
Q13325	Interferon-induced protein with tetratricopeptide repeats 5	IFIT5			
Q6PI98	INO80 complex subunit C	INO80C			
P14784	Interleukin-2 receptor subunit beta	IL2RB			
Q86VG3	Intraflagellar transport-associated protein	IFTAP			
P01591	Immunoglobulin J chain	JCHAIN			
P05019	Insulin-like growth factor I	IGF1			
P52333	Tyrosine-protein kinase JAK3	JAK3			
P06756	Integrin alpha-V	ITGAV			
P12035	Keratin, type II cytoskeletal 3	KRT3			
Q15825	Neuronal acetylcholine receptor subunit alpha-6	CHRNA6			
P02749	Beta-2-glycoprotein 1	APOH			
Q6VUC0	Transcription factor AP-2-epsilon	TFAP2E			
Q9P241	Phospholipid-transporting ATPase VD	ATP10D			
C9JJ37	BTB/POZ domain-containing protein 19	BTBD19			
Q7KYR7	Butyrophilin subfamily 2 member A1	BTN2A1			
A8MTZ0	BBSome-interacting protein 1	BBIP1			
Q53S33	BolA-like protein 3	BOLA3			
P47710	Alpha-S1-casein	CSN1S1			
Q8IVL8	Carboxypeptidase O	CPO			
O00626	C-C motif chemokine 22	CCL22			
O43633	Charged multivesicular body protein 2a	CHMP2A			
Q9Y258	C-C motif chemokine 26	CCL26			
Q96FZ7	Charged multivesicular body protein 6	CHMP6			
Q8WUX2	Glutathione-specific gamma-glutamylcyclotransferase 2	CHAC2			
Q9BWV3	Cytidine and dCMP deaminase domain-containing protein 1	CDADC1			
Q96MI9	Cytosolic carboxypeptidase 4	AGBL1			
O95170	CMT1A duplicated region transcript 1 protein	CDRT1			
Q13231	Chitotriosidase-1	CHIT1			
P11836	B-lymphocyte antigen CD20	MS4A1			
P60953	Cell division control protein 42 homolog	CDC42			
Q9H7B7	Uncharacterized protein C7orf69	C7orf69			
Q8NI28	Putative uncharacterized protein encoded by LINC01006	LINC01006			
Q49A92	Uncharacterized protein C8orf34	C8orf34			
Q8N8U2	Chromodomain Y-like protein 2	CDYL2			
Q8NCQ2	Uncharacterized protein CSNK1G2-AS1	CSNK1G2-AS1			
Q9UMR7	C-type lectin domain family 4 member A	CLEC4A			
Q8N910	Putative uncharacterized protein C15orf56	C15orf56			
A8TX70	Collagen alpha-5	COL6A5			
Q8IY22	C-Maf-inducing protein	CMIP			
Q8IZM0	Putative CNGA1-overlapping antisense gene protein				
Q99828	Calcium and integrin-binding protein 1	CIB1			
Q00610	Clathrin heavy chain 1	CLTC			
Q6UXG3	CMRF35-like molecule 9	CD300LG			
Q6UWR7	Glycerophosphocholine cholinephosphodiesterase ENPP6	ENPP6			
P15954	Cytochrome c oxidase subunit 7C, mitochondrial	COX7C			
P04798	Cytochrome P450 1A1	CYP1A1			

Q14264	Endogenous retrovirus group 3 member 1 Env polyprotein	ERV3-1			
O43768	Alpha-endosulfine	ENSA			
Q7L2H7	Eukaryotic translation initiation factor 3 subunit M	EIF3M			
P43897	Elongation factor Ts,	TSFM			
B5ME19	Eukaryotic translation initiation factor 3 subunit C-like protein	EIF3CL			
P61550	Endogenous retrovirus group S71 member 1 Env polyprotein	ERVS71-1			
Q9NYP7	Elongation of very long chain fatty acids protein 5	ELOVL5			
Q9H6Z9	Prolyl hydroxylase EGLN3	EGLN3			
B3EWF7	Laforin, isoform 9	EPM2A			
P49961	Ectonucleoside triphosphate diphosphohydrolase 1	ENTPD1			
Q5UCC4	ER membrane protein complex subunit 10	EMC10			
Q96L91	E1A-binding protein p400	EP400			
Q6ZR85	Uncharacterized protein C17orf107	C17orf107			
Q8NE35	Cytoplasmic polyadenylation element-binding protein 3	CPEB3			
A2RUQ5	Uncharacterized protein C17orf102	C17orf102			
P10632	Cytochrome P450 2C8	CYP2C8			
P15509	Granulocyte-macrophage colony-stimulating factor receptor subunit alpha	CSF2RA			
Q14241	Elongin-A	ELOA			
P54764	Ephrin type-A receptor 4	EPHA4			
Q5NDL2	EGF domain-specific O-linked N-acetylglucosamine transferase	EOGT			
Q96DE9	Protein EOLA2	EOLA2			
P52798	Ephrin-A4	EFNA4			
Q96PS1	FANCD2 opposite strand protein	FANCD2OS			
P61579	Endogenous retrovirus group K member 25 Rec protein	ERVK-25			
Q6IPT2	Protein FAM71E1	FAM71E1			
Q6NSI3	Protein FAM53A	FAM53A			
Q5HYJ3	Protein FAM76B	FAM76B			
P08709	Coagulation factor VII	F7			
O94868	F-BAR and double SH3 domains protein 2	FCHSD2			
P68106	Peptidyl-prolyl cis-trans isomerase FKBP1B	FKBP1B			
P02042	Hemoglobin subunit delta	HBD			
P26885	Peptidyl-prolyl cis-trans isomerase FKBP2	FKBP2			
Q8NEZ5	F-box only protein 22	FBXO22			
Q9BRP7	Ferredoxin-fold anticodon-binding domain-containing protein 1	FDXACB1			
O60258	Fibroblast growth factor 17	FGF17			
Q92914	Fibroblast growth factor 11	FGF11			
Q96LL3	Fertilization-influencing membrane protein	FIMP			
Q96RP7	Galactose-3-O-sulfotransferase 4	GAL3ST4			
Q8TAE8	Growth arrest and DNA damage-inducible proteins-interacting protein 1	GADD45GI P1			
Q8WWW8	GRB2-associated-binding protein 3	GAB3			
Q96AE4	Far upstream element-binding protein 1	FUBP1			
P51116	Fragile X mental retardation syndrome-related protein 2	FXR2			
P55318	Hepatocyte nuclear factor 3-gamma	FOXA3			

P43694	Transcription factor GATA-4	GATA4			
Q5JY77	G-protein coupled receptor-associated sorting protein 1	GPRASP1			
Q9C009	Forkhead box protein Q1	FOXQ1			
Q9H1K4	Mitochondrial glutamate carrier 2	SLC25A18			
O14793	Growth/differentiation factor 8	MSTN			
Q9C0B1	Alpha-ketoglutarate-dependent dioxygenase FTO	FTO			
Q9H840	Gem-associated protein 7	GEMIN7			
Q02108	Guanylate cyclase soluble subunit alpha-1	GUCY1A1			
O14893	Gem-associated protein 2	GEMIN2			
O00591	Gamma-aminobutyric acid receptor subunit pi	GABRP			
Q96PP9	Guanylate-binding protein 4	GBP4			
O15544	Protein GR6	LINC01565			
P47871	Glucagon receptor	GCGR			
Q9P1Z3	Potassium/sodium hyperpolarization-activated cyclic nucleotide-gated channel 3	HCN3			
Q6EKJ0	General transcription factor II-I repeat domain-containing protein 2B	GTF2IRD2B			
P49915	GMP synthase	GMPS			
Q16772	Glutathione S-transferase A3	GSTA3			
P08263	Glutathione S-transferase A1	GSTA1			
Q99878	Histone H2A type 1-J	H2AC14			
Q6Y1H2	Very-long-chain	HACD2			
Q13255	Metabotropic glutamate receptor 1	GRM1			
Q13003	Glutamate receptor ionotropic, kainate 3	GRIK3			
P0CG29	Glutathione S-transferase theta-2	GSTT2			
Q16478	Glutamate receptor ionotropic, kainate 5	GRIK5			
Q99075	Proheparin-binding EGF-like growth factor	HBEGF			
O94992	Protein HEXIM1	HEXIM1			
Q02577	Helix-loop-helix protein 2	NHLH2			
P22830	Ferrochelatase, mitochondrial	FECH			
Q8WWQ2	Inactive heparanase-2	HPSE2			
Q9BUJ0	Protein ABHD14A	ABHD14A			
P30939	5-hydroxytryptamine receptor 1F	HTR1F			
P47898	5-hydroxytryptamine receptor 5A	HTR5A			
P28222	5-hydroxytryptamine receptor 1B	HTR1B			
P50406	5-hydroxytryptamine receptor 6	HTR6			
P62258	14-3-3 protein epsilon	YWHAE			
Q9P0M2	A-kinase anchor protein 7 isoform gamma	AKAP7			
P24298	Alanine aminotransferase 1	GPT			
Q6NXT1	Ankyrin repeat domain-containing protein 54	ANKRD54			
Q8WTP8	Apoptosis-enhancing nuclease	AEN			
Q96BT7	Alkylated DNA repair protein alkB homolog 8	ALKBH8			
Q06481	Amyloid-like protein 2	APLP2			
O75843	AP-1 complex subunit gamma-like 2	AP1G2			
Q96BI3	Gamma-secretase subunit APH-1A	APH1A			
Q9NVJ2	ADP-ribosylation factor-like protein 8B	ARL8B			
P08133	Annexin A6	ANXA6			
Q6Q788	Apolipoprotein A-V	APOA5			
P34998	Corticotropin-releasing factor receptor 1	CRHR1			
Q9BU40	Chordin-like protein 1	CHRDL1			
P24462	Cytochrome P450 3A7	CYP3A7			
A0A087X1C5	Putative cytochrome P450 2D7	CYP2D7			

Q8NEV1	Casein kinase II subunit alpha 3	CSNK2A3			
Q9H0B8	Cysteine-rich secretory protein LCCL domain-containing 2	CRISPLD2			
A6NCJ1	Uncharacterized protein C19orf71	C19orf71			
Q8N268	Uncharacterized protein C20orf197	C20orf197			
P14406	Cytochrome c oxidase subunit 7A2, mitochondrial	COX7A2			
Q02221	Cytochrome c oxidase subunit 6A2, mitochondrial	COX6A2			
Q9Y5B0	RNA polymerase II subunit A C-terminal domain phosphatase	CTDP1			
A6NN92	Putative gap junction epsilon-1 protein	GJE1			
Q8IX94	cTAGE family member 4	CTAGE4			
O43927	C-X-C motif chemokine 13	CXCL13			
P32302	C-X-C chemokine receptor type 5	CXCR5			
Q6ZN68	Putative C-mannosyltransferase DPY19L2P2	DPY19L2P2			
Q9NRG7	Epimerase family protein	SDR39U1			
Q8N9T2	Putative uncharacterized protein CXorf42	NKAPP1			
Q16696	Cytochrome P450 2A13	CYP2A13			
Q8WYN3	Cysteine/serine-rich nuclear protein 3	CSRNP3			
Q7L576	Cytoplasmic FMR1-interacting protein 1	CYFIP1			
Q9NUD7	Uncharacterized protein C20orf96	C20orf96			
O76096	Cystatin-F	CST7			
P49238	CX3C chemokine receptor 1	CX3CR1			
Q9H4E7	Differentially expressed in FDCP 6 homolog	DEF6			
Q8WXU2	Dynein assembly factor 4, axonemal	DNAAF4			
Q6PH85	DCN1-like protein 2	DCUN1D2			
Q96D03	DNA damage-inducible transcript 4-like protein	DDIT4L			
Q13206	Probable ATP-dependent RNA helicase DDX10	DDX10			
Q8NES8	Beta-defensin 124	DEFB124			
Q58WW2	DDB1- and CUL4-associated factor 6	DCAF6			
Q96HY7	Probable 2-oxoglutarate dehydrogenase E1 component DHKTD1, mitochondrial	DHTKD1			
Q5VZB9	Doublesex- and mab-3-related transcription factor A1	DMRTA1			
Q8TF46	DIS3-like exonuclease 1	DIS3L			
O60879	Protein diaphanous homolog 2	DIAPH2			
Q8IYB7	DIS3-like exonuclease 2	DIS3L2			
Q92874	Deoxyribonuclease-1-like 2	DNASE1L2			
Q96QD5	DEP domain-containing protein 7	DEPDC7			
Q86XP1	Diacylglycerol kinase eta	DGKH			
Q9H7Y0	Divergent protein kinase domain 2B	DIPK2B			
Q12959	Disks large homolog 1	DLG1			
P05305	Endothelin-1	EDN1			
O14640	Segment polarity protein dishevelled homolog DVL-1	DVL1			
Q9BV47	Dual specificity protein phosphatase 26	DUSP26			
P28340	DNA polymerase delta catalytic subunit	POLD1			
Q05923	Dual specificity protein phosphatase 2	DUSP2			
P01903	HLA class II histocompatibility antigen, DR alpha chain	HLA-DRA			
Q7RTS9	Dymeclin	DYM			

P19525	Interferon-induced, double-stranded RNA-activated protein kinase	EIF2AK2			
P32249	G-protein coupled receptor 183	GPR183			
Q9HC44	Vasculin-like protein 1	GPBP1L1			
Q9UPQ8	Dolichol kinase	DOLK			
P21917	D	DRD4			
Q13474	Dystrophin-related protein 2	DRP2			
P55259	Pancreatic secretory granule membrane major glycoprotein GP2	GP2			
P24534	Elongation factor 1-beta	EEF1B2			
Q99848	Probable rRNA-processing protein EBP2	EBNA1BP2			
Q8N335	Glycerol-3-phosphate dehydrogenase 1-like protein	GPD1L			
Q9BRR8	G patch domain-containing protein 1	GPATCH1			
Q9NW75	G patch domain-containing protein 2	GPATCH2			
Q9UJA9	Ectonucleotide pyrophosphatase/phosphodiesterase family member 5	ENPP5			
Q53HC9	EARP and GARP complex-interacting protein 1	EIPR1			
Q9UBC2	Epidermal growth factor receptor substrate 15-like 1	EPS15L1			
Q9HBU6	Ethanolamine kinase 1	ETNK1			
P98073	Enteropeptidase	TMPRSS15			
Q14507	Epididymal secretory protein E3-alpha	EDDM3A			
Q12926	ELAV-like protein 2	ELAVL2			
P54762	Ephrin type-B receptor 1	EPHB1			
Q15717	ELAV-like protein 1	ELAVL1			
Q8TE02	Elongator complex protein 5	ELP5			
Q9NZN3	EH domain-containing protein 3	EHD3			
Q8N7U6	EF-hand domain-containing family member B	EFHB			
Q9NRD1	F-box only protein 6	FBXO6			
P09769	Tyrosine-protein kinase Fgr	FGR			
P0C7X4	Putative ferritin heavy polypeptide-like 19	FTH1P19			
Q8TDW7	Protocadherin Fat 3	FAT3			
Q14331	Protein FRG1	FRG1			
O95866	Megakaryocyte and platelet inhibitory receptor G6b	MPIG6B			
Q75N90	Fibrillin-3	FBN3			
P24522	Growth arrest and DNA damage-inducible protein GADD45 alpha	GADD45A			
Q96IG2	F-box/LRR-repeat protein 20	FBXL20			
O95633	Follistatin-related protein 3	FSTL3			
P19440	Glutathione hydrolase 1 proenzyme	GGT1			
Q5YKI7	Putative gametogenetin-binding protein 1	GGNBP1			
Q04446	1,4-alpha-glucan-branching enzyme	GBE1			
O75223	Gamma-glutamylcyclotransferase	GGCT			
O00258	Guided entry of tail-anchored proteins factor 1	GET1			
Q8N9F7	Lysophospholipase D GDPD1	GDPD1			
Q9UKD1	Glucocorticoid modulatory element-binding protein 2	GMEB2			
Q9BYB4	Guanine nucleotide-binding protein subunit beta-like protein 1	GNB1L			
Q9Y2T3	Guanine deaminase	GDA			
P11166	Solute carrier family 2, facilitated glucose transporter member 1	SLC2A1			
Q9UJM8	Hydroxyacid oxidase 1	HAO1			

P22303	Acetylcholinesterase	ACHE			
P16219	Short-chain specific acyl-CoA dehydrogenase, mitochondrial	ACADS			
Q9Y614	Actin-like protein 7B	ACTL7B			
Q8WXA8	5-hydroxytryptamine receptor 3C	HTR3C			
Q9NS82	Asc-type amino acid transporter 1	SLC7A10			
Q99217	Amelogenin, X isoform	AMELX			
P49419	Alpha-aminoadipic semialdehyde dehydrogenase	ALDH7A1			
Q96NN9	Apoptosis-inducing factor 3	AIFM3			
Q8NCV1	Adenosine deaminase domain-containing protein 2	ADAD2			
Q8IVF2	Protein AHNAK2	AHNAK2			
Q86TW2	AarF domain-containing protein kinase 1	ADCK1			
P07550	Beta-2 adrenergic receptor	ADRB2			
P10109	Adrenodoxin, mitochondrial	FDX1			
Q32M45	Anoctamin-4	ANO4			
Q6UB99	Ankyrin repeat domain-containing protein 11	ANKRD11			
Q9NVM4	Protein arginine N-methyltransferase 7	PRMT7			
Q02410	Amyloid-beta A4 precursor protein-binding family A member 1	APBA1			
Q6P2P2	Protein arginine N-methyltransferase 9	PRMT9			
Q0VD83	Apolipoprotein B receptor	APOBR			
Q9H6L4	Armadillo repeat-containing protein 7	ARMC7			
Q92974	Rho guanine nucleotide exchange factor 2	ARHGEF2			
O43488	Aflatoxin B1 aldehyde reductase member 2	AKR7A2			
O43315	Aquaporin-9	AQP9			
Q8NFD2	Ankyrin repeat and protein kinase domain-containing protein 1	ANKK1			
P35626	Beta-adrenergic receptor kinase 2	GRK3			
Q01484	Ankyrin-2	ANK2			
Q7Z3E5	LisH domain-containing protein ARMC9	ARMC9			
Q9H993	Damage-control phosphatase ARMT1	ARMT1			
Q99929	Achaete-scute homolog 2	ASCL2			
O95352	Ubiquitin-like modifier-activating enzyme ATG7	ATG7			
Q96SW2	Protein cereblon	CRBN			
Q0VDD7	Break repair meiotic recombinase recruitment factor 1	BRME1			
Q9C0B6	BMP/retinoic acid-inducible neural-specific protein 2	BRINP2			
P13497	Bone morphogenetic protein 1	BMP1			
Q9NW68	BSD domain-containing protein 1	BSDC1			
Q9BYV7	Beta,beta-carotene 9',10'-oxygenase	BCO2			
Q6AI39	BRD4-interacting chromatin-remodeling complex-associated protein-like	BICRAL			
Q8WWF8	Calcyphosin-like protein	CAPSL			
Q8NFC6	Biorientation of chromosomes in cell division protein 1-like 1	BOD1L1			
Q8NFJ8	Class E basic helix-loop-helix protein 22	BHLHE22			
Q96PG8	Bcl-2-binding component 3, isoforms 3/4	BBC3			
Q9NS00	Glycoprotein-N-acetylgalactosamine 3-beta-galactosyltransferase 1	C1GALT1			

Q2M329	Coiled-coil domain-containing protein 96	CCDC96			
Q5T0U0	Coiled-coil domain-containing protein 122	CCDC122			
Q86YS7	C2 domain-containing protein 5	C2CD5			
P33076	MHC class II transactivator	CIITA			
Q6ZRV3	Putative uncharacterized protein encoded by LINC00696	LINC00696			
P10092	Calcitonin gene-related peptide 2	CALCB			
Q05682	Caldesmon	CALD1			
Q86X55	Histone-arginine methyltransferase CARM1	CARM1			
P53701	Holocytochrome c-type synthase	HCCS			
Q15003	Condensin complex subunit 2	NCAPH			
Q96SN8	CDK5 regulatory subunit-associated protein 2	CDK5RAP2			
Q8WWK9	Cytoskeleton-associated protein 2	CKAP2			
Q96KP4	Cytosolic non-specific dipeptidase	CNDP2			
Q9UBY8	Protein CLN8	CLN8			
Q8IYJ1	Copine-9	CPNE9			
Q96FN4	Copine-2	CPNE2			
Q9HB03	Elongation of very long chain fatty acids protein 3	ELOVL3			
A1XBS5	Protein FAM92A	FAM92A			
Q9UI10	Translation initiation factor eIF-2B subunit delta	EIF2B4			
O43731	ER lumen protein-retaining receptor 3	KDELR3			
Q96CS3	FAS-associated factor 2	FAF2			
Q6L9T8	Protein FAM72D	FAM72D			
Q8WWV6	High affinity immunoglobulin alpha and immunoglobulin mu Fc receptor	FCAMR			
Q99518	Dimethylaniline monooxygenase	FMO2			
Q9NVF7	F-box only protein 28	FBXO28			
Q5XUX0	F-box only protein 31	FBXO31			
Q8TF61	F-box only protein 41	FBXO41			
Q9UJT9	F-box/LRR-repeat protein 7	FBXL7			
P0C7T7	Putative uncharacterized protein FRMD6-AS1	FRMD6-AS1			
P39748	Flap endonuclease 1	FEN1			
Q96CD0	F-box/LRR-repeat protein 8	FBXL8			
Q8N441	Fibroblast growth factor receptor-like 1	FGFRL1			
P22087	rRNA 2'-O-methyltransferase fibrillarin	FBL			
O95177	Uncharacterized protein GAS8-AS1	GAS8-AS1			
Q86XJ1	GAS2-like protein 3	GAS2L3			
Q13283	Ras GTPase-activating protein-binding protein 1	G3BP1			
Q7LDI9	Endogenous retrovirus group K member 6 Gag polyprotein	ERVK-6			
O14556	Glyceraldehyde-3-phosphate dehydrogenase, testis-specific	GAPDHS			
O95073	Fibrinogen silencer-binding protein	FSBP			
Q9BTV5	Fibronectin type III and SPRY domain-containing protein 1	FSD1			
P57678	Gem-associated protein 4	GEMIN4			
Q96RT8	Gamma-tubulin complex component 5	TUBGCP5			
P59768	Guanine nucleotide-binding protein G	GNG2			
Q9P109	Beta-1,3-galactosyl-O-glycosyl-glycoprotein beta-1,6-N-acetylglucosaminyltransferase 4	GCNT4			
P04899	Guanine nucleotide-binding protein G	GNAI2			

Q9Y692	Glucocorticoid modulatory element-binding protein 1	GMEB1			
Q9NYA3	Golgin subfamily A member 6A	GOLGA6A			
Q13588	GRB2-related adapter protein	GRAP			
P40197	Platelet glycoprotein V	GP5			
Q99445	Glycosyl-phosphatidylinositol-anchored molecule-like protein	GML			
O94925	Glutaminase kidney isoform, mitochondrial	GLS			
Q9P2T1	GMP reductase 2	GMPR2			
Q92643	GPI-anchor transamidase	PIGK			
P56524	Histone deacetylase 4	HDAC4			
Q8TAX9	Gasdermin-B	GSDMB			
Q9H772	Gremlin-2	GREM2			
Q02539	Histone H1.1	H1-1			
Q9NZI5	Grainyhead-like protein 1 homolog	GRHL1			
P15516	Histatin-3	HTN3			
O95336	6-phosphogluconolactonase	PGLS			
O95342	Bile salt export pump	ABCB11			
Q7L211	Protein ABHD13	ABHD13			
Q9UGJ0	5'-AMP-activated protein kinase subunit gamma-2	PRKAG2			
Q9BXI3	Cytosolic 5'-nucleotidase 1A	NT5C1A			
P63151	Serine/threonine-protein phosphatase 2A 55 kDa regulatory subunit B alpha isoform	PPP2R2A			
Q13685	Angio-associated migratory cell protein	AAMP			
Q96M93	Adenosine deaminase domain-containing protein 1	ADAD1			
Q6NS38	DNA oxidative demethylase ALKBH2	ALKBH2			
Q96SZ5	2-aminoethanethiol dioxygenase	ADO			
P18089	Alpha-2B adrenergic receptor	ADRA2B			
Q9H0C2	ADP/ATP translocase 4	SLC25A31			
A8MVX0	Rho guanine nucleotide exchange factor 33	ARHGEF33			
P28039	Acyloxyacyl hydrolase	AOAH			
Q10567	AP-1 complex subunit beta-1	AP1B1			
Q92572	AP-3 complex subunit sigma-1	AP3S1			
P53365	Arfaptin-2	ARFIP2			
Q9NXL2	Rho guanine nucleotide exchange factor 38	ARHGEF38			
Q8WZ64	Arf-GAP with Rho-GAP domain, ANK repeat and PH domain-containing protein 2	ARAP2			
Q9UII2	ATPase inhibitor, mitochondrial	ATP5IF1			
Q9NSI6	Bromodomain and WD repeat-containing protein 1	BRWD1			
P12643	Bone morphogenetic protein 2	BMP2			
Q9ULD4	Bromodomain and PHD finger-containing protein 3	BRPF3			
Q96A19	Coiled-coil domain-containing protein 102A	CCDC102A			
P22003	Bone morphogenetic protein 5	BMP5			
Q01954	Zinc finger protein basonuclin-1	BNC1			
P16442	Histo-blood group ABO system transferase	ABO			
Q8IZY5	BH3-like motif-containing cell death inducer	BLID			
O75363	Breast carcinoma-amplified sequence 1	BCAS1			
O60885	Bromodomain-containing protein 4	BRD4			
Q6UX72	UDP-GlcNAc:betaGal beta-1,3-N-acetylglucosaminyltransferase 9	B3GNT9			
P61769	Beta-2-microglobulin	B2M			
P53004	Biliverdin reductase A	BLVRA			

Q58F21	Bromodomain testis-specific protein	BRDT			
O76090	Bestrophin-1	BEST1			
P15291	Beta-1,4-galactosyltransferase 1	B4GALT1			
O94812	BAI1-associated protein 3	BAIAP3			
Q14457	Beclin-1	BECN1			
Q9NQY0	Bridging integrator 3	BIN3			
Q01668	Voltage-dependent L-type calcium channel subunit alpha-1D	CACNA1D			
Q5VUE5	Uncharacterized protein C1orf53	C1orf53			
Q13111	Chromatin assembly factor 1 subunit A	CHAF1A			
Q5SR53	Putative uncharacterized protein PIK3CD-AS1	PIK3CD-AS1			
O75493	Carbonic anhydrase-related protein 11	CA11			
O14523	Phospholipid transfer protein C2CD2L	C2CD2L			
Q96M95	Coiled-coil domain-containing protein 42	CCDC42			
A6NI79	Coiled-coil domain-containing protein 69	CCDC69			
Q8IV32	Coiled-coil domain-containing protein 71	CCDC71			
P31415	Calsequestrin-1	CASQ1			
Q9NQ75	Cas scaffolding protein family member 4	CASS4			
Q9Y2G2	Caspase recruitment domain-containing protein 8	CARD8			
Q8ND23	Capping protein, Arp2/3 and myosin-I linker protein 3	CARMIL3			
Q8N163	Cell cycle and apoptosis regulator protein 2	CCAR2			
P60033	CD81 antigen	CD81			
O15078	Centrosomal protein of 290 kDa	CEP290			
P0C7V0	Putative uncharacterized protein encoded by LINC00271	LINC00271			
Q7Z4T9	Cilia- and flagella-associated protein 91	CFAP91			
Q96CF2	Charged multivesicular body protein 4c	CHMP4C			
Q6ZQR2	Cilia- and flagella-associated protein 77	CFAP77			
O15519	CASP8 and FADD-like apoptosis regulator	CFLAR			
P32929	Cystathionine gamma-lyase	CTH			
Q9UKY7	Protein CDV3 homolog	CDV3			
Q5T2Q4	Cyclin-Y-like protein 2	CCNYL2			
Q99674	Cell growth regulator with EF hand domain protein 1	CGREF1			
P49450	Histone H3-like centromeric protein A	CENPA			
Q4VX62	Putative uncharacterized protein C6orf99	C6orf99			
Q86WJ1	Chromodomain-helicase-DNA-binding protein 1-like	CHD1L			
Q3L8U1	Chromodomain-helicase-DNA-binding protein 9	CHD9			
Q8N5I9	Uncharacterized protein C12orf45	C12orf45			
A8MT69	Centromere protein X	CENPX			
Q96NB1	Centrosomal protein 20	CEP20			
Q9NYJ1	Cytochrome c oxidase assembly factor 4 homolog, mitochondrial	COA4			
P51911	Calponin-1	CNN1			
Q8WZ69	Putative uncharacterized protein C11orf40	C11orf40			
Q9BQ75	Protein CMSS1	CMSS1			
Q9H0W9	Ester hydrolase C11orf54	C11orf54			

Q96DZ9	CKLF-like MARVEL transmembrane domain-containing protein 5	CMTM5			
O76031	ATP-dependent Clp protease ATP-binding subunit clpX-like, mitochondrial	CLPX			
Q9NWW5	Ceroid-lipofuscinosis neuronal protein 6	CLN6			
P98187	Cytochrome P450 4F8	CYP4F8			
J3KSC0	Putative uncharacterized protein encoded by LINC01387	LINC01387			
Q16526	Cryptochrome-1	CRY1			
A0A1B0GTH6	Casein kinase II subunit alpha'-interacting protein	CSNKA2IP			
O95377	Gap junction beta-5 protein	GJB5			
Q99708	DNA endonuclease RBBP8	RBBP8			
O14595	Carboxy-terminal domain RNA polymerase II polypeptide A small phosphatase 2	CTDSP2			
Q8WUS8	Short-chain dehydrogenase/reductase family 42E member 1	SDR42E1			
Q5VT33	Putative uncharacterized protein encoded by LINC01545	LINC01545			
P60022	Beta-defensin 1	DEFB1			
Q8NEG7	Protein DENND6B	DENND6B			
Q86XP3	ATP-dependent RNA helicase DDX42	DDX42			
Q9NXZ2	Probable ATP-dependent RNA helicase DDX43	DDX43			
O75553	Disabled homolog 1	DAB1			
O94830	Phospholipase DDHD2	DDHD2			
Q30KQ4	Beta-defensin 116	DEFB116			
P35638	DNA damage-inducible transcript 3 protein	DDIT3			
Q30KP9	Beta-defensin 135	DEFB135			
Q92904	Deleted in azoospermia-like	DAZL			
O14521	Succinate dehydrogenase	SDHD			
Q30KQ1	Beta-defensin 133	DEFB133			
Q96HP0	Dedicator of cytokinesis protein 6	DOCK6			
P52824	Diacylglycerol kinase theta	DGKQ			
P37058	Testosterone 17-beta-dehydrogenase 3	HSD17B3			
P56177	Homeobox protein DLX-1	DLX1			
Q8N9I9	Probable E3 ubiquitin-protein ligase DTX3	DTX3			
P25101	Endothelin-1 receptor	EDNRA			
Q13202	Dual specificity protein phosphatase 8	DUSP8			
P01133	Pro-epidermal growth factor	EGF			
O43281	Embryonal Fyn-associated substrate	EFS			
Q5JPI9	EEF1A lysine methyltransferase 2	EEF1AKMT			
O43921	Ephrin-A2	EFNA2			
A2A2Y4	FERM domain-containing protein 3	FRMD3			
Q9H469	F-box/LRR-repeat protein 15	FBXL15			
P07954	Fumarate hydratase,	FH			
P62683	Endogenous retrovirus group K member 21 Gag polyprotein	ERVK-21			
A0A2Z4LIS9	Forkhead box protein O3B	FOXO3B			
Q16676	Forkhead box protein D1	FOXD1			
Q99853	Forkhead box protein B1	FOXB1			
Q13070	G antigen 6	GAGE6			
P14324	Farnesyl pyrophosphate synthase	FDPS			
Q14393	Growth arrest-specific protein 6	GAS6			
Q9HCQ5	Polypeptide N-acetylgalactosaminyltransferase 9	GALNT9			

Q96D09	G-protein coupled receptor-associated sorting protein 2	GPRASP2			
Q8N9W4	Golgin subfamily A member 6-like protein 2	GOLGA6L2			
P0CL81	G antigen 12G	GAGE12G			
O60262	Guanine nucleotide-binding protein G	GNG7			
Q9BSJ2	Gamma-tubulin complex component 2	TUBGCP2			
Q7L5L3	Lysophospholipase D GDPD3	GDPD3			
O76003	Glutaredoxin-3	GLRX3			
O60741	Potassium/sodium hyperpolarization-activated cyclic nucleotide-gated channel 1	HCN1			
Q8TDF6	RAS guanyl-releasing protein 4	RASGRP4			
Q9HAV7	GrpE protein homolog 1, mitochondrial	GRPEL1			
P49840	Glycogen synthase kinase-3 alpha	GSK3A			
Q8N7I0	Protein GVQW1	GVQW1			
P20671	Histone H2A type 1-D	H2AC7			
Q9BZM3	GS homeobox 2	GSX2			
Q99871	HAUS augmin-like complex subunit 7	HAUS7			
Q6UWZ7	BRCA1-A complex subunit Abraxas 1	ABRAXAS1			
Q969K4	Ankyrin repeat and BTB/POZ domain-containing protein 1	ABTB1			
A8K2U0	Alpha-2-macroglobulin-like protein 1	A2ML1			
Q9BTE6	Alanyl-tRNA editing protein Aarsd1	AARSD1			
Q7Z5M8	Protein ABHD12B	ABHD12B			
P00519	Tyrosine-protein kinase ABL1	ABL1			
P30532	Neuronal acetylcholine receptor subunit alpha-5	CHRNA5			
Q5BKT4	Dol-P-Glc:Glc	ALG10			
Q9Y4X0	AMME syndrome candidate gene 1 protein	AMMECR1			
P35611	Alpha-adducin	ADD1			
Q6IQ32	Activity-dependent neuroprotector homeobox protein 2	ADNP2			
Q96P64	Arf-GAP with GTPase, ANK repeat and PH domain-containing protein 4	AGAP4			
Q9H568	Actin-like protein 8	ACTL8			
Q01718	Adrenocorticotropic hormone receptor	MC2R			
P17516	Aldo-keto reductase family 1 member C4	AKR1C4			
Q99965	Disintegrin and metalloproteinase domain-containing protein 2	ADAM2			
O43306	Adenylate cyclase type 6	ADCY6			
Q6DHV7	Adenosine deaminase-like protein	ADAL			
Q86SQ6	Adhesion G protein-coupled receptor A1	ADGRA1			
Q53FZ2	Acyl-coenzyme A synthetase ACSM3, mitochondrial	ACSM3			
Q9Y6Z5	Putative uncharacterized protein AFDN-DT	AFDN-DT			
Q13790	Apolipoprotein F	APOF			
Q8N9B4	Ankyrin repeat domain-containing protein 42	ANKRD42			
Q6T311	ADP-ribosylation factor-like protein 9	ARL9			
Q9NR80	Rho guanine nucleotide exchange factor 4	ARHGEF4			
Q8N8L6	ADP-ribosylation factor-like protein 10	ARL10			

Q5SZL2	Centrosomal protein of 85 kDa-like	CEP85L			
Q9HC52	Chromobox protein homolog 8	CBX8			
Q9H8W2	Putative uncharacterized protein encoded by LINC00472	LINC00472			
P0DN79	Cystathionine beta-synthase-like protein	CBSL			
O15335	Chondroadherin	CHAD			
Q13042	Cell division cycle protein 16 homolog	CDC16			
Q86SI9	Protein CEI	C5orf38			
Q5SZQ8	CUGBP Elav-like family member 3	CELF3			
Q711Q0	Cardiac-enriched FHL2-interacting protein	CEFIP			
Q9Y3Y2	Chromatin target of PRMT1 protein	CHTOP			
Q8NAJ2	Putative uncharacterized protein C9orf106	C9orf106			
Q5BLP8	Neuropeptide-like protein	C4orf48			
Q13319	Cyclin-dependent kinase 5 activator 2	CDK5R2			
Q2HXU8	C-type lectin domain family 12 member B	CLEC12B			
Q8N815	Cyclin N-terminal domain-containing protein 1	CNTD1			
A0A1B0GVM6	Uncharacterized protein C11orf97	C11orf97			
Q9P2W6	Uncharacterized protein C11orf21	C11orf21			
Q8WTT0	C-type lectin domain family 4 member C	CLEC4C			
P0C0L5	Complement C4-B	C4B			
O43174	Cytochrome P450 26A1	CYP26A1			
P24903	Cytochrome P450 2F1	CYP2F1			
Q9P0S2	Cytochrome c oxidase assembly protein COX16 homolog, mitochondrial	COX16			
K7EIQ3	Uncharacterized protein ZNF561-AS1	ZNF561-AS1			
Q9H112	Cystatin-11	CST11			
Q8N144	Gap junction delta-3 protein	GJD3			
Q86XM0	Cation channel sperm-associated protein subunit delta	CATSPERD			
O60494	Cubilin	CUBN			
Q9GZN8	UPF0687 protein C20orf27	C20orf27			
P51397	Death-associated protein 1	DAP			
Q9H816	5' exonuclease Apollo	DCLRE1B			
Q9H295	Dendritic cell-specific transmembrane protein	DCSTAMP			
Q8N9W5	Dynein assembly factor 3, axonemal	DNAAF3			
Q9NR90	Deleted in azoospermia protein 3	DAZ3			
Q9UHG0	Doublecortin domain-containing protein 2	DCDC2			
Q09019	Dystrophia myotonica WD repeat-containing protein	DMWD			
P60981	Destrin	DSTN			
Q9NNZ3	DnaJ homolog subfamily C member 4	DNAJC4			
P49916	DNA ligase 3	LIG3			
Q16854	Deoxyguanosine kinase, mitochondrial	DGUOK			
Q9UBS4	DnaJ homolog subfamily B member 11	DNAJB11			
Q02413	Desmoglein-1	DSG1			
Q96DT5	Dynein heavy chain 11, axonemal	DNAH11			
Q9Y6K1	DNA	DNMT3A			
Q8TD57	Dynein heavy chain 3, axonemal	DNAH3			
Q15723	ETS-related transcription factor Elf-2	ELF2			

Q9UKW6	ETS-related transcription factor Elf-5	ELF5			
Q9NQZ7	Ectonucleoside triphosphate diphosphohydrolase 7	ENTPD7			
Q8WVX9	Fatty acyl-CoA reductase 1	FAR1			
P0C7X0	Putative protein FAM90A24P	FAM90A24P			
Q9NYF5	Protein FAM13B	FAM13B			
P0C7W9	Putative protein FAM90A14P	FAM90A14P			
Q9NVL1	Putative protein FAM86C1P	FAM86C1P			
Q8N2R8	Protein FAM43A	FAM43A			
P15170	Eukaryotic peptide chain release factor GTP-binding subunit ERF3A	GSPT1			
Q8NA70	Protein FAM47B	FAM47B			
Q8IXL6	Extracellular serine/threonine protein kinase FAM20C	FAM20C			
Q9BZE7	UPF0193 protein EVG1	C22orf23			
Q9NVI1	Fanconi anemia group I protein	FANCI			
Q8IYD8	Fanconi anemia group M protein	FANCM			
P15311	Ezrin	EZR			
O95990	Actin-associated protein FAM107A	FAM107A			
Q8WUF8	Cotranscriptional regulator FAM172A	FAM172A			
Q8TAY7	Protein FAM110D	FAM110D			
A6PVY3	Protein FAM177B	FAM177B			
Q6IPR1	Electron transfer flavoprotein regulatory factor 1	ETFRF1			
Q14677	Clathrin interactor 1	CLINT1			
Q8N0U4	Protein FAM185A	FAM185A			
Q15024	Exosome complex component RRP42	EXOSC7			
Q9NVH0	Exonuclease 3'-5' domain-containing protein 2	EXD2			
A6NFA0	Protein FAM205C	FAM205C			
Q9H201	Epsin-3	EPN3			
Q8WUB2	Protein FAM216A	FAM216A			
P16118	6-phosphofructo-2-kinase/fructose-2,6-bisphosphatase 1	PFKFB1			
Q9NQ60	Equatorin	EQTN			
Q92935	Exostosin-like 1	EXTL1			
Q9Y6R7	IgGFc-binding protein	FCGBP			
Q05932	Folylpolyglutamate synthase, mitochondrial	FPGS			
Q9H9S5	Fukutin-related protein	FKRP			
Q5MNV8	F-box only protein 47	FBXO47			
B3EWG3	Protein FAM25A	FAM25A			
O43427	Acidic fibroblast growth factor intracellular-binding protein	FIBP			
A2VDF0	Fucose mutarotase	FUOM			
Q9UBS5	Gamma-aminobutyric acid type B receptor subunit 1	GABBR1			
Q96I24	Far upstream element-binding protein 3	FUBP3			
Q5VVW2	GTPase-activating Rap/Ran-GAP domain-like protein 3	GARNL3			
O15353	Forkhead box protein N1	FOXN1			
O15409	Forkhead box protein P2	FOXP2			
Q14C86	GTPase-activating protein and VPS9 domain-containing protein 1	GAPVD1			
B5MD39	Putative glutathione hydrolase light chain 3	GGTLC3			
P01225	Follitropin subunit beta	FSHB			
Q96MS3	Glycosyltransferase 1 domain-containing protein 1	GLT1D1			
Q03113	Guanine nucleotide-binding protein subunit alpha-12	GNA12			

Q7Z6J2	Protein TAMALIN	TAMALIN			
Q9Y3E0	Vesicle transport protein GOT1B	GOLT1B			
Q08AF8	Putative golgin subfamily A member 8F/8G	GOLGA8F;			
Q14439	G-protein coupled receptor 176	GPR176			
P38405	Guanine nucleotide-binding protein G	GNAL			
Q14451	Growth factor receptor-bound protein 7	GRB7			
P32189	Glycerol kinase	GK			
O43194	G-protein coupled receptor 39	GPR39			
P46089	G-protein coupled receptor 3	GPR3			
Q9Y2T5	G-protein coupled receptor 52	GPR52			
Q6UWM5	GLIPR1-like protein 1	GLIPR1L1			
Q16695	Histone H3.1t	H3-4			
Q96P50	Arf-GAP with coiled-coil, ANK repeat and PH domain-containing protein 3	ACAP3			
Q9NUN7	Alkaline ceramidase 3	ACER3			
P31937	3-hydroxyisobutyrate dehydrogenase, mitochondrial	HIBADH			
Q9NP78	ATP-binding cassette sub-family B member 9	ABCB9			
Q9UDR5	Alpha-aminoadipic semialdehyde synthase, mitochondrial	AASS			
P20309	Muscarinic acetylcholine receptor M3	CHRM3			
Q05901	Neuronal acetylcholine receptor subunit beta-3	CHRNB3			
P31947	14-3-3 protein sigma	SFN			
P55196	Afadin	AFDN			
Q08AH3	Acyl-coenzyme A synthetase ACSM2A, mitochondrial	ACSM2A			
Q9H013	Disintegrin and metalloproteinase domain-containing protein 19	ADAM19			
Q6QNK2	Adhesion G-protein coupled receptor D1	ADGRD1			
O60241	Adhesion G protein-coupled receptor B2	ADGRB2			
Q9BYT9	Anoctamin-3	ANO3			
Q8N283	Ankyrin repeat domain-containing protein 35	ANKRD35			
Q7Z713	Ankyrin repeat domain-containing protein 37	ANKRD37			
Q12955	Ankyrin-3	ANK3			
Q9NWX5	Ankyrin repeat and SOCS box protein 6	ASB6			
Q9H1I8	Activating signal cointegrator 1 complex subunit 2	ASCC2			
Q6HA08	Astacin-like metalloendopeptidase	ASTL			
Q01814	Plasma membrane calcium-transporting ATPase 2	ATP2B2			
P10523	S-arrestin	SAG			
P0DMR3	Putative protein ATXN8OS	ATXN8OS			
O00192	Armadillo repeat protein deleted in velo-cardio-facial syndrome	ARVCF			
Q8N5I2	Arrestin domain-containing protein 1	ARRDC1			
P56880	Claudin-20	CLDN20			
O14493	Claudin-4	CLDN4			
P54252	Ataxin-3	ATXN3			
Q8NHS1	Claudin domain-containing protein 2	CLDND2			
Q5TC12	ATP synthase mitochondrial F1 complex assembly factor 1	ATPAF1			
Q9H324	A disintegrin and metalloproteinase with thrombospondin motifs 10	ADAMTS10			

Q6UX73	UPF0764 protein C16orf89	C16orf89			
Q9BXS0	Collagen alpha-1	COL25A1			
A8MZG2	Uncharacterized protein C16orf90	C16orf90			
P00395	Cytochrome c oxidase subunit 1	MT-CO1			
Q9H693	Uncharacterized protein C16orf95	C16orf95			
P00403	Cytochrome c oxidase subunit 2	MT-CO2			
P53621	Coatomer subunit alpha	COPA			
P05108	Cholesterol side-chain cleavage enzyme, mitochondrial	CYP11A1			
Q7Z408	CUB and sushi domain-containing protein 2	CSMD2			
Q7Z407	CUB and sushi domain-containing protein 3	CSMD3			
P35606	Coatomer subunit beta'	COPB2			
Q9UPN4	Centrosomal protein of 131 kDa	CEP131			
P53618	Coatomer subunit beta	COPB1			
P20023	Complement receptor type 2	CR2			
Q66GS9	Centrosomal protein of 135 kDa	CEP135			
Q13098	COP9 signalosome complex subunit 1	GPS1			
Q8WTW3	Conserved oligomeric Golgi complex subunit 1	COG1			
Q96BA8	Cyclic AMP-responsive element-binding protein 3-like protein 1	CREB3L1			
Q14746	Conserved oligomeric Golgi complex subunit 2	COG2			
Q70SY1	Cyclic AMP-responsive element-binding protein 3-like protein 2	CREB3L2			
P48444	Coatomer subunit delta	ARCN1			
P61201	COP9 signalosome complex subunit 2	COPS2			
Q96JB2	Conserved oligomeric Golgi complex subunit 3	COG3			
Q68CJ9	Cyclic AMP-responsive element-binding protein 3-like protein 3	CREB3L3			
P05093	Steroid 17-alpha-hydroxylase/17,20 lyase	CYP17A1			
O14579	Coatomer subunit epsilon	COPE			
Q8TEY5	Cyclic AMP-responsive element-binding protein 3-like protein 4	CREB3L4			
Q9H9E3	Conserved oligomeric Golgi complex subunit 4	COG4			
Q9Y678	Coatomer subunit gamma-1	COPG1			
P10606	Cytochrome c oxidase subunit 5B, mitochondrial	COX5B			
Q9UP83	Conserved oligomeric Golgi complex subunit 5	COG5			
Q9UNS2	COP9 signalosome complex subunit 3	COPS3			
Q9UBF2	Coatomer subunit gamma-2	COPG2			
Q5RI15	Cytochrome c oxidase assembly protein COX20, mitochondrial	COX20			
Q9Y2V7	Conserved oligomeric Golgi complex subunit 6	COG6			
Q9NQ79	Cartilage acidic protein 1	CRTAC1			
Q9BT78	COP9 signalosome complex subunit 4	COPS4			
P09669	Cytochrome c oxidase subunit 6C	COX6C			
Q6ZU35	Capping protein inhibiting regulator of actin dynamics	CRACD			
Q9NQ92	Coordinator of PRMT5 and differentiation stimulator	COPRS			
P83436	Conserved oligomeric Golgi complex subunit 7	COG7			
O15431	High affinity copper uptake protein 1	SLC31A1			
P78560	Death domain-containing protein CRADD	CRADD			
P11511	Aromatase	CYP19A1			

O15432	Probable low affinity copper uptake protein 2	SLC31A2			
Q9P218	Collagen alpha-1	COL20A1			
Q9Y6Z7	Collectin-10	COLEC10			
Q9UBW8	COP9 signalosome complex subunit 7a	COPS7A			
P26998	Beta-crystallin B3	CRYBB3			
Q9Y4K1	Beta/gamma crystallin domain-containing protein 1	CRYBG1			
P05177	Cytochrome P450 1A2	CYP1A2			
P00414	Cytochrome c oxidase subunit 3	MT-CO3			
Q7Z7K0	COX assembly mitochondrial protein homolog	CMC1			
Q9BWP8	Collectin-11	COLEC11			
Q9H9Q2	COP9 signalosome complex subunit 7b	COPS7B			
Q8N1P7	Beta/gamma crystallin domain-containing protein 2	CRYBG2			
Q68DQ2	Very large A-kinase anchor protein	CRYBG3			
Q8N668	COMM domain-containing protein 1	COMMD1			
Q9NRP2	COX assembly mitochondrial protein 2 homolog	CMC2			
Q5KU26	Collectin-12	COLEC12			
O60519	cAMP-responsive element-binding protein-like 2	CREBL2			
Q86X83	COMM domain-containing protein 2	COMMD2			
Q9UBI1	COMM domain-containing protein 3	COMMD3			
Q9H0A8	COMM domain-containing protein 4	COMMD4			
P07333	Macrophage colony-stimulating factor 1 receptor	CSF1R			
Q16678	Cytochrome P450 1B1	CYP1B1			
Q9GZQ3	COMM domain-containing protein 5	COMMD5			
Q14019	Coactosin-like protein	COTL1			
Q7Z4G1	COMM domain-containing protein 6	COMMD6			
Q6UW02	Cytochrome P450 20A1	CYP20A1			
Q86VX2	COMM domain-containing protein 7	COMMD7			
Q9NX08	COMM domain-containing protein 8	COMMD8			
P09603	Macrophage colony-stimulating factor 1	CSF1			
Q9P000	COMM domain-containing protein 9	COMMD9			
Q96P44	Collagen alpha-1	COL21A1			
Q5H9J7	Protein BEX5	BEX5			
O95972	Bone morphogenetic protein 15	BMP15			
P30043	Flavin reductase	BLVRB			
Q8N4F0	BPI fold-containing family B member 2	BPIFB2			
P78537	Biogenesis of lysosome-related organelles complex 1 subunit 1	BLOC1S1			
P15538	Cytochrome P450 11B1, mitochondrial	CYP11B1			
Q86WA6	Valacyclovir hydrolase	BPHL			
Q6PJG6	BRCA1-associated ATM activator 1	BRAT1			
P04920	Anion exchange protein 2	SLC4A2			
P51587	Breast cancer type 2 susceptibility protein	BRCA2			
P50895	Basal cell adhesion molecule	BCAM			
P56817	Beta-secretase 1	BACE1			

Q9H3F6	BTB/POZ domain-containing adapter for CUL3-mediated RhoA degradation protein 3	KCTD10			
O75752	UDP-GalNAc:beta-1,3-N-acetylgalactosaminyltransferase 1	B3GALNT1			
O00512	B-cell CLL/lymphoma 9 protein	BCL9			
P43235	Cathepsin K	CTSK			
Q8N1D5	Uncharacterized protein C1orf158	C1orf158			
Q6NSX1	Coiled-coil domain-containing protein 70	CCDC70			
P42574	Caspase-3	CASP3			
Q8N5R6	Coiled-coil domain-containing protein 33	CCDC33			
Q9UBN1	Voltage-dependent calcium channel gamma-4 subunit	CACNG4			
Q9P1Z9	Coiled-coil domain-containing protein 180	CCDC180			
Q92793	CREB-binding protein	CREBBP			
Q6V702	Cilia- and flagella-associated protein 299	CFAP299			
Q7Z460	CLIP-associating protein 1	CLASP1			
Q14031	Collagen alpha-6	COL4A6			
Q96GR2	Long-chain-fatty-acid--CoA ligase ACSBG1	ACSBG1			
Q9NUQ8	ATP-binding cassette sub-family F member 3	ABCF3			
P20594	Atrial natriuretic peptide receptor 2	NPR2			
O95782	AP-2 complex subunit alpha-1	AP2A1			
Q5JPF3	Ankyrin repeat domain-containing protein 36C	ANKRD36C			
P41181	Aquaporin-2	AQP2			
Q8N7S6	Uncharacterized protein ARIH2OS	ARIH2OS			
Q9Y2Y0	ADP-ribosylation factor-like protein 2-binding protein	ARL2BP			
O95841	Angiopoietin-related protein 1	ANGPTL1			
Q9HBZ2	Aryl hydrocarbon receptor nuclear translocator 2	ARNT2			
P20933	N	AGA			
O14744	Protein arginine N-methyltransferase 5	PRMT5			
Q9UBL0	cAMP-regulated phosphoprotein 21	ARPP21			
P50747	Biotin--protein ligase	HLCS			
Q96RK4	Bardet-Biedl syndrome 4 protein	BBS4			
O43521	Bcl-2-like protein 11	BCL2L11			
P56945	Breast cancer anti-estrogen resistance protein 1	BCAR1			
Q9UL15	BAG family molecular chaperone regulator 5	BAG5			
Q9UQB8	Brain-specific angiogenesis inhibitor 1-associated protein 2	BAIAP2			
I3L3R5	Coiled-coil domain-containing glutamate-rich protein 2	CCER2			
P22223	Cadherin-3	CDH3			
Q16568	Cocaine- and amphetamine-regulated transcript protein	CARTPT			
P0CE67	Putative uncharacterized protein encoded by LINC02877	LINC02877			
A6NI56	Coiled-coil domain-containing protein 154	CCDC154			
Q6PK04	Coiled-coil domain-containing protein 137	CCDC137			
Q8IVU9	Ciliary-associated calcium-binding coiled-coil protein 1	CABCOCO1			
Q92851	Caspase-10	CASP10			
P55773	C-C motif chemokine 23	CCL23			
Q8NHU2	Cilia- and flagella-associated protein 61	CFAP61			

P08603	Complement factor H	CFH			
Q9BWS9	Chitinase domain-containing protein 1	CHID1			
O95400	CD2 antigen cytoplasmic tail-binding protein 2	CD2BP2			
Q7L1S5	Carbohydrate sulfotransferase 9	CHST9			
Q8N884	Cyclic GMP-AMP synthase	CGAS			
Q9BZC1	CUGBP Elav-like family member 4	CELF4			
Q9BUW7	UPF0184 protein C9orf16	C9orf16			
P46527	Cyclin-dependent kinase inhibitor 1B	CDKN1B			
P19397	Leukocyte surface antigen CD53	CD53			
Q5VYM1	Uncharacterized protein C9orf131	C9orf131			
P24385	G1/S-specific cyclin-D1	CCND1			
Q5JQF7	Putative uncharacterized protein encoded by LINC01556	LINC01556			
O96020	G1/S-specific cyclin-E2	CCNE2			
Q8NCG5	Carbohydrate sulfotransferase 4	CHST4			
P27544	Ceramide synthase 1	CERS1			
Q9NRU3	Metal transporter CNNM1	CNNM1			
Q9P126	C-type lectin domain family 1 member B	CLEC1B			
Q16740	ATP-dependent Clp protease proteolytic subunit, mitochondrial	CLPP			
Q6ZU45	Putative C-type lectin domain family 20 member A	CLEC20A			
Q13956	Retinal cone rhodopsin-sensitive cGMP 3',5'-cyclic phosphodiesterase subunit gamma	PDE6H			
Q8N2M8	CLK4-associating serine/arginine rich protein	CLASRP			
P08123	Collagen alpha-2	COL1A2			
Q8TEF2	Uncharacterized protein C10orf105	C10orf105			
Q15021	Condensin complex subunit 1	NCAPD2			
P33260	Cytochrome P450 2C18	CYP2C18			
O75462	Cytokine receptor-like factor 1	CRLF1			
Q96RY5	Protein cramped-like	CRAMP1			
Q9UFG5	UPF0449 protein C19orf25	C19orf25			
A0A1B0GTL2	Uncharacterized protein C20orf204	C20orf204			
Q5TEA3	Uncharacterized protein C20orf194	C20orf194			
P0DMU8	Cancer/testis antigen family 45 member A5	CT45A5			
Q9Y2S2	Lambda-crystallin homolog	CRYL1			
P46108	Adapter molecule crk	CRK			
Q8NHU0	Cancer/testis antigen family 45 member A3	CT45A3			
P19876	C-X-C motif chemokine 3	CXCL3			
Q07325	C-X-C motif chemokine 9	CXCL9			
P56545	C-terminal-binding protein 2	CTBP2			
Q6UX04	Spliceosome-associated protein CWC27 homolog	CWC27			
Q9NTM9	Copper homeostasis protein cutC homolog	CUTC			
Q5W186	Cystatin-9	CST9			
Q14999	Cullin-7	CUL7			
Q13619	Cullin-4A	CUL4A			
Q14154	DAP3-binding cell death enhancer 1	DELE1			
Q13561	Dynactin subunit 2	DCTN2			
Q86Y56	Dynein assembly factor 5, axonemal	DNAAF5			
P59103	D-amino acid oxidase activator	DAOA			
Q96B18	Dapper homolog 3	DACT3			
Q9UHL0	ATP-dependent RNA helicase DDX25	DDX25			

O00571	ATP-dependent RNA helicase DDX3X	DDX3X			
Q9H9R9	Dysbindin domain-containing protein 1	DBNDD1			
Q9BVC3	Sister chromatid cohesion protein DCC1	DSCC1			
Q9BY27	Protein DGCR6L	DGCR6L			
Q96PD7	Diacylglycerol O-acyltransferase 2	DGAT2			
Q7Z478	ATP-dependent RNA helicase DHX29	DHX29			
Q8IX18	Probable ATP-dependent RNA helicase DHX40	DHX40			
Q9NVH1	DnaJ homolog subfamily C member 11	DNAJC11			
Q6ZR08	Dynein heavy chain 12, axonemal	DNAH12			
Q9UNI6	Dual specificity protein phosphatase 12	DUSP12			
O43854	EGF-like repeat and discoidin I-like domain-containing protein 3	EDIL3			
Q8TC29	Enkurin	ENKUR			
Q00013	55 kDa erythrocyte membrane protein	MPP1			
Q9NPA0	ER membrane protein complex subunit 7	EMC7			
Q8N6R0	eEF1A lysine and N-terminal methyltransferase	EEF1AKNMT			
Q9Y6B2	EP300-interacting inhibitor of differentiation 1	EID1			
Q4L180	Filamin A-interacting protein 1-like	FILIP1L			
Q5HY92	Fidgetin	FIGN			
Q9Y613	FH1/FH2 domain-containing protein 1	FHOD1			
O95750	Fibroblast growth factor 19	FGF19			
Q13642	Four and a half LIM domains protein 1	FHL1			
Q14517	Protocadherin Fat 1	FAT1			
Q7Z6J6	FERM domain-containing protein 5	FRMD5			
A9Z1Z3	Fer-1-like protein 4	FER1L4			
P63145	Endogenous retrovirus group K member 24 Gag polyprotein	ERVK-24			
Q9Y231	4-galactosyl-N-acetylglucosaminide 3-alpha-L-fucosyltransferase 9	FUT9			
Q4VC44	FLYWCH-type zinc finger-containing protein 1	FLYWCH1			
Q6NT46	G antigen 2A	GAGE2A			
Q9H334	Forkhead box protein P1	FOXP1			
Q9H1C3	Glycosyltransferase 8 domain-containing protein 2	GLT8D2			
P0CL82	G antigen 12I	GAGE12I			
O75420	GRB10-interacting GYF protein 1	GIGYF1			
Q6UWU2	Beta-galactosidase-1-like protein	GLB1L			
Q969I3	Glycine N-acyltransferase-like protein 1	GLYATL1			
A6NDK9	Golgin subfamily A member 6C	GOLGA6C			
Q86SP6	Probable G-protein coupled receptor 149	GPR149			
Q7Z601	Probable G-protein coupled receptor 142	GPR142			
Q8TDT2	Probable G-protein coupled receptor 152	GPR152			
A6NN73	Golgin subfamily A member 8C	GOLGA8CP			
Q86XS8	E3 ubiquitin-protein ligase	RNF130			
Q49SQ1	Probable G-protein coupled receptor 33	GPR33			
Q9NQS5	G-protein coupled receptor 84	GPR84			

P43080	Guanylyl cyclase-activating protein 1	GUCA1A			
P07492	Gastrin-releasing peptide	GRP			
P57764	Gasdermin-D	GSDMD			
P0C1H6	Histone H2B type F-M	H2BW2			
Q9Y3E1	Hepatoma-derived growth factor-related protein 3	HDGFL3			
P04908	Histone H2A type 1-B/E	H2AC4			
P49748	Very long-chain specific acyl-CoA dehydrogenase, mitochondrial	ACADVL			
P36544	Neuronal acetylcholine receptor subunit alpha-7	CHRNA7			
P11229	Muscarinic acetylcholine receptor M1	CHRM1			
Q9NY61	Protein AATF	AATF			
Q0P651	Protein ABHD18	ABHD18			
Q15847	Adipogenesis regulatory factor	ADIRF			
Q9H2P0	Activity-dependent neuroprotector homeobox protein	ADNP			
P84996	Protein ALEX	GNAS			
P30038	Delta-1-pyrroline-5-carboxylate dehydrogenase, mitochondrial	ALDH4A1			
P52594	Arf-GAP domain and FG repeat-containing protein 1	AGFG1			
Q9H0P7	Putative uncharacterized protein encoded by AGPAT4-IT1	AGPAT4-IT1			
P15121	Aldo-keto reductase family 1 member B1	AKR1B1			
Q5TGY3	AT-hook DNA-binding motif-containing protein 1	AHDC1			
Q9BT30	Alpha-ketoglutarate-dependent dioxygenase alkB homolog 7, mitochondrial	ALKBH7			
Q53H80	Akirin-2	AKIRIN2			
Q8IZF7	Adhesion G-protein coupled receptor F2	ADGRF2			
Q6GMV1	Putative glycosyltransferase ALG1-like	ALG1L			
Q8IZ07	Ankyrin repeat domain-containing protein 13A	ANKRD13A			
P01008	Antithrombin-III	SERPINC1			
O95626	Acidic leucine-rich nuclear phosphoprotein 32 family member D	ANP32D			
Q96LR9	Apolipoprotein L domain-containing protein 1	APOLD1			
Q96NW4	Ankyrin repeat domain-containing protein 27	ANKRD27			
Q13795	ADP-ribosylation factor-related protein 1	ARFRP1			
Q9NP61	ADP-ribosylation factor GTPase-activating protein 3	ARFGAP3			
P12429	Annexin A3	ANXA3			
Q9BQE5	Apolipoprotein L2	APOL2			
Q6NXE6	Armadillo repeat-containing protein 6	ARMC6			
Q66PJ3	ADP-ribosylation factor-like protein 6-interacting protein 4	ARL6IP4			
A6NFN9	Protein ANKUB1	ANKUB1			
P04424	Argininosuccinate lyase	ASL			
Q86XS5	Angiopoietin-related protein 5	ANGPTL5			
Q10588	ADP-ribosyl cyclase/cyclic ADP-ribose hydrolase 2	BST1			
P43251	Biotinidase	BTD			
Q96KV6	Putative butyrophilin subfamily 2 member A3	BTN2A3P			
Q3C1V8	Brain-specific homeobox protein homolog	BSX			
Q6UX41	Butyrophilin-like protein 8	BTNL8			

Q12830	Nucleosome-remodeling factor subunit BPTF	BPTF			
Q9BQP9	BPI fold-containing family A member 3	BPIFA3			
P35070	Probetacellulin	BTC			
Q9H165	B-cell lymphoma/leukemia 11A	BCL11A			
Q9UBV7	Beta-1,4-galactosyltransferase 7	B4GALT7			
Q92560	Ubiquitin carboxyl-terminal hydrolase BAP1	BAP1			
Q6ZUV0	Putative cytosolic acyl coenzyme A thioester hydrolase-like	ACOT7L			
Q9H503	Barrier-to-autointegration factor-like protein	BANF2			
Q6PI77	Protein BHLHb9	BHLHB9			
Q86V35	Calcium-binding protein 7	CABP7			
P20851	C4b-binding protein beta chain	C4BPB			
P52907	F-actin-capping protein subunit alpha-1	CAPZA1			
Q9BXY5	Calcyphosin-2	CAPS2			
Q3KRA6	UPF0538 protein C2orf76	C2orf76			
O60911	Cathepsin L2	CTSV			
Q9H159	Cadherin-19	CDH19			
P55283	Cadherin-4	CDH4			
Q96MC4	CEP295 N-terminal-like protein	CEP295NL			
Q8TDN4	CDK5 and ABL1 enzyme substrate 1	CABLES1			
Q96MF4	Coiled-coil domain-containing protein 140	CCDC140			
Q13948	Protein CASP	CUX1			
Q8IYX3	Coiled-coil domain-containing protein 116	CCDC116			
Q15834	Coiled-coil domain-containing protein 85B	CCDC85B			
A0A1B0GUA6	Putative coiled-coil domain-containing protein 195	CCDC195			
Q6UXH8	Collagen and calcium-binding EGF domain-containing protein 1	CCBE1			
Q1T7F1	Putative chemokine-related protein B42				
Q86UT8	Centrosomal AT-AC splicing	CENATAC			
P55774	C-C motif chemokine 18	CCL18			
Q9BUX1	Glutathione-specific gamma-glutamylcyclotransferase 1	CHAC1			
Q96BS2	Calcineurin B homologous protein 3	TESC			
Q7LFX5	Carbohydrate sulfotransferase 15	CHST15			
O14627	Homeobox protein CDX-4	CDX4			
Q8NCH0	Carbohydrate sulfotransferase 14	CHST14			
Q8N6W0	CUGBP Elav-like family member 5	CELF5			
Q8N4C0	Putative uncharacterized protein C9orf62	C9orf62			
Q9H6J7	UPF0705 protein C11orf49	C11orf49			
Q9Y471	Inactive cytidine monophosphate-N-acetylneuraminic acid hydroxylase	CMAHP			
Q86XI2	Condensin-2 complex subunit G2	NCAPG2			
Q9NZA1	Chloride intracellular channel protein 5	CLIC5			
P30622	CAP-Gly domain-containing linker protein 1	CLIP1			
Q6WN34	Chordin-like protein 2	CHRDL2			
P56282	DNA polymerase epsilon subunit	POLE2			
P09884	DNA polymerase alpha catalytic subunit	POLA1			
Q96FJ2	Dynein light chain 2, cytoplasmic	DYNLL2			
Q6P4E1	Protein GOLM2	GOLM2			
Q8TDU6	G-protein coupled bile acid receptor 1	GPBAR1			

P79483	HLA class II histocompatibility antigen, DR beta 3 chain	HLA-DRB3			
Q9Y6X5	Bis	ENPP4			
Q9H9B1	Histone-lysine N-methyltransferase EHMT1	EHMT1			
O95864	Acyl-CoA 6-desaturase	FADS2			
P16930	Fumarylacetoacetase	FAH			
Q8N7N1	Putative protein N-methyltransferase FAM86B1	FAM86B1			
A8MX19	Putative protein FAM90A12P	FAM90A12P			
Q7Z2K6	Endoplasmic reticulum metallopeptidase 1	ERMP1			
A6NEW6	Putative protein FAM90A16P/FAM90A17P	FAM90A16P			
O76062	Delta	TM7SF2			
Q8NFF5	FAD synthase	FLAD1			
Q8TC99	Fibronectin type III domain-containing protein 8	FNDC8			
Q15485	Ficolin-2	FCN2			
Q06787	Synaptic functional regulator	FMR1			
Q96LA5	Fc receptor-like protein 2	FCRL2			
Q5VW36	Focadhesin	FOCAD			
Q6P3S6	F-box only protein 42	FBXO42			
Q8TAL6	Fin bud initiation factor homolog	FIBIN			
Q9Y4F1	FERM, ARHGEF and pleckstrin domain-containing protein 1	FARP1			
Q6V0I7	Protocadherin Fat 4	FAT4			
A6NFH5	Fatty acid-binding protein 12	FABP12			
Q8IVP5	FUN14 domain-containing protein 1	FUNDC1			
P02792	Ferritin light chain	FTL			
Q9P0K9	DOMON domain-containing protein FRRS1L	FRRS1L			
O00451	GDNF family receptor alpha-2	GFRA2			
P55107	Growth/differentiation factor 10	GDF10			
P56159	GDNF family receptor alpha-1	GFRA1			
Q8N5D6	Globoside alpha-1,3-N-acetylgalactosaminyltransferase 1	GBGT1			
Q9HCG7	Non-lysosomal glucosylceramidase	GBA2			
Q96P48	Arf-GAP with Rho-GAP domain, ANK repeat and PH domain-containing protein 1	ARAP1			
P15514	Amphiregulin	AREG			
Q99873	Protein arginine N-methyltransferase 1	PRMT1			
Q9HCJ1	Progressive ankylosis protein homolog	ANKH			
Q7L4S7	Protein ARMCX6	ARMCX6			
Q08708	CMRF35-like molecule 6	CD300C			
Q13733	Sodium/potassium-transporting ATPase subunit alpha-4	ATP1A4			
Q3KR37	Protein Aster-B	GRAMD1B			
Q9NQ11	Polyamine-transporting ATPase 13A2	ATP13A2			
P51689	Arylsulfatase D	ARSD			
P20020	Plasma membrane calcium-transporting ATPase 1	ATP2B1			
P98194	Calcium-transporting ATPase type 2C member 1	ATP2C1			
Q86TL0	Cysteine protease ATG4D	ATG4D			
P49407	Beta-arrestin-1	ARRB1			
P59998	Actin-related protein 2/3 complex subunit 4	ARPC4			
Q96QE3	ATPase family AAA domain-containing protein 5	ATAD5			
P46100	Transcriptional regulator ATRX	ATRX			

P59510	A disintegrin and metalloproteinase with thrombospondin motifs 20	ADAMTS20			
Q9H6S1	5-azacytidine-induced protein 2	AZI2			
P20648	Potassium-transporting ATPase alpha chain 1	ATP4A			
P56378	ATP synthase subunit ATP5MPL, mitochondrial	ATP5MPL			
Q6UY14	ADAMTS-like protein 4	ADAMTSL4			
P78410	Butyrophilin subfamily 3 member A2	BTN3A2			
Q9UBX8	Beta-1,4-galactosyltransferase 6	B4GALT6			
P80723	Brain acid soluble protein 1	BASP1			
Q7L3V2	Protein Bop	RTL10			
Q9H0E9	Bromodomain-containing protein 8	BRD8			
P82251	b	SLC7A9			
Q7RTS1	Class A basic helix-loop-helix protein 15	BHLHA15			
Q9H2M3	S-methylmethionine--homocysteine S-methyltransferase BHMT2	BHMT2			
Q9Y6Q1	Calpain-6	CAPN6			
Q68DN1	Uncharacterized protein C2orf16	C2orf16			
Q16602	Calcitonin gene-related peptide type 1 receptor	CALCRL			
P20160	Azurocidin	AZU1			
P12830	Cadherin-1	CDH1			
P27482	Calmodulin-like protein 3	CALML3			
Q9NZT1	Calmodulin-like protein 5	CALML5			
P49662	Caspase-4	CASP4			
P51878	Caspase-5	CASP5			
Q0P6D6	Coiled-coil domain-containing protein 15	CCDC15			
Q8WXS4	Voltage-dependent calcium channel gamma-like subunit	TMEM37			
Q9UPV0	Centrosomal protein of 164 kDa	CEP164			
Q9NVL8	Uncharacterized protein CCDC198	CCDC198			
Q9BRQ4	Cilia- and flagella-associated protein 300	CFAP300			
P16152	Carbonyl reductase	CBR1			
Q9UJX2	Cell division cycle protein 23 homolog	CDC23			
Q96J87	CUGBP Elav-like family member 6	CELF6			
Q92879	CUGBP Elav-like family member 1	CELF1			
Q5JTW2	Centrosomal protein of 78 kDa	CEP78			
Q8N7R7	Cyclin-Y-like protein 1	CCNYL1			
A0A0K2S4Q6	Protein CD300H	CD300H			
A4QMS7	Uncharacterized protein C5orf49	C5orf49			
P0C7X3	Putative cyclin-Y-like protein 3	CCNYL3			
Q8IVW4	Cyclin-dependent kinase-like 3	CDKL3			
Q99741	Cell division control protein 6 homolog	CDC6			
Q92674	Centromere protein I	CENPI			
Q9HC77	Centromere protein J	CENPJ			
P14635	G2/mitotic-specific cyclin-B1	CCNB1			
Q6ZTQ4	Cadherin-related family member	CDHR3			
P29016	T-cell surface glycoprotein CD1b	CD1B			
P07360	Complement component C8 gamma chain	C8G			
O00622	CCN family member 1	CCN1			
Q9H2U6	Putative uncharacterized protein encoded by LINC00597	LINC00597			
Q86TU6	Putative uncharacterized protein encoded by LINC00523	LINC00523			
P62633	Cellular nucleic acid-binding protein	CNBP			

A4QN01	Putative uncharacterized protein encoded by LINC01553	LINC01553			
O75339	Cartilage intermediate layer protein 1	CILP			
Q86Y22	Collagen alpha-1	COL23A1			
Q5TZA2	Rootletin	CROCC			
P35212	Gap junction alpha-4 protein	GJA4			
O60716	Catenin delta-1	CTNND1			
P19875	C-X-C motif chemokine 2	CXCL2			
Q99895	Chymotrypsin-C	CTRC			
Q6ZMK1	Cysteine and histidine-rich protein 1	CYHR1			
Q8N465	D-2-hydroxyglutarate dehydrogenase, mitochondrial	D2HGDH			
Q9P013	Spliceosome-associated protein CWC15 homolog	CWC15			
Q01523	Defensin-5	DEFA5			
Q16698	2,4-dienoyl-CoA reductase	DECR1			
Q96EX3	Cytoplasmic dynein 2 intermediate chain 2	DYNC2I2			
Q8NFW5	Diencephalon/mesencephalon homeobox protein 1	DMBX1			
Q6UWP2	Dehydrogenase/reductase SDR family member 11	DHRS11			
O00273	DNA fragmentation factor subunit alpha	DFFA			
Q8IY37	Probable ATP-dependent RNA helicase DHX37	DHX37			
Q9BSY9	Deubiquitinase DESI2	DESI2			
Q4LDG9	Dynein light chain 1, axonemal	DNAL1			
Q8NDZ4	Divergent protein kinase domain 2A	DIPK2A			
Q8NHS0	DnaJ homolog subfamily B member 8	DNAJB8			
Q92608	Dedicator of cytokinesis protein 2	DOCK2			
O75140	GATOR complex protein DEPDC5	DEPDC5			
Q96LL9	DnaJ homolog subfamily C member 30, mitochondrial	DNAJC30			
O60479	Homeobox protein DLX-3	DLX3			
A7MBM2	Protein dispatched homolog 2	DISP2			
Q92611	ER degradation-enhancing alpha-mannosidase-like protein 1	EDEM1			
Q9C0G6	Dynein heavy chain 6, axonemal	DNAH6			
Q96FN9	D-aminoacyl-tRNA deacylase 2	DTD2			
Q9UL01	Dermatan-sulfate epimerase	DSE			
Q92997	Segment polarity protein dishevelled homolog DVL-3	DVL3			
P54098	DNA polymerase subunit gamma-1	POLG			
Q16829	Dual specificity protein phosphatase 7	DUSP7			
Q9NZJ5	Eukaryotic translation initiation factor 2-alpha kinase 3	EIF2AK3			
P0DPD6	Endothelin-converting enzyme 2	ECE2			
Q8WW35	Dynein light chain Tctex-type protein 2B	DYNLT2B			
P68104	Elongation factor 1-alpha 1	EEF1A1			
A6NFQ7	Divergent paired-related homeobox	DPRX			
P30084	Enoyl-CoA hydratase, mitochondrial	ECHS1			
Q99795	Cell surface A33 antigen	GPA33			
Q8IUC8	Polypeptide N-acetylgalactosaminyltransferase 13	GALNT13			
P07203	Glutathione peroxidase 1	GPX1			
Q96IJ6	Mannose-1-phosphate guanyltransferase alpha	GMPPA			
Q96FL9	Polypeptide N-acetylgalactosaminyltransferase 14	GALNT14			

Q8N3T1	Polypeptide N-acetylgalactosaminyltransferase 15	GALNT15			
Q8N428	Polypeptide N-acetylgalactosaminyltransferase 16	GALNT16			
Q6IS24	Polypeptide N-acetylgalactosaminyltransferase 17	GALNT17			
P22352	Glutathione peroxidase 3	GPX3			
P14770	Platelet glycoprotein IX	GP9			
A6NH11	Glycolipid transfer protein domain-containing protein 2	GLTPD2			
Q5T6L9	Endoplasmic reticulum membrane-associated RNA degradation protein	ERMARD			
Q8IYW4	ENTH domain-containing protein 1	ENTHD1			
Q09472	Histone acetyltransferase p300	EP300			
Q6PCB8	Embigin	EMB			
Q9NVF9	Ethanolamine kinase 2	ETNK2			
Q4LEZ3	Alanine and arginine-rich domain-containing protein	AARD			
P46098	5-hydroxytryptamine receptor 3A	HTR3A			
P05408	Neuroendocrine protein 7B2	SCG5			
Q96QU6	1-aminocyclopropane-1-carboxylate synthase-like protein 1	ACCS			
Q96GS6	Alpha/beta hydrolase domain-containing protein 17A	ABHD17A			
Q8N5Z0	Kynurenine/alpha-aminoadipate aminotransferase, mitochondrial	AADAT			
Q8IUX4	DNA dC->dU-editing enzyme APOBEC-3F	APOBEC3F			
O95477	Phospholipid-transporting ATPase ABCA1	ABCA1			
Q03154	Aminoacylase-1	ACY1			
Q8IZF5	Adhesion G-protein coupled receptor F3	ADGRF3			
Q86Y34	Adhesion G protein-coupled receptor G3	ADGRG3			
P05141	ADP/ATP translocase 2	SLC25A5			
Q9Y653	Adhesion G-protein coupled receptor G1	ADGRG1			
Q92624	Amyloid protein-binding protein 2	APPBP2			
Q3LIE5	Manganese-dependent ADP-ribose/CDP-alcohol diphosphatase	ADPRM			
Q8TF21	Ankyrin repeat domain-containing protein 24	ANKRD24			
Q9BWW8	Apolipoprotein L6	APOL6			
Q8N4G2	ADP-ribosylation factor-like protein 14	ARL14			
P07306	Asialoglycoprotein receptor 1	ASGR1			
Q96DR5	BPI fold-containing family A member 2	BPIFA2			
Q5BKX8	Caveolae-associated protein 4	CAVIN4			
P43166	Carbonic anhydrase 7	CA7			
O95180	Voltage-dependent T-type calcium channel subunit alpha-1H	CACNA1H			
P41182	B-cell lymphoma 6 protein	BCL6			
P55290	Cadherin-13	CDH13			
P29400	Collagen alpha-5	COL4A5			
Q14CN2	Calcium-activated chloride channel regulator 4	CLCA4			
P20908	Collagen alpha-1	COL5A1			
P23786	Carnitine O-palmitoyltransferase 2, mitochondrial	CPT2			
Q8NEA5	Uncharacterized protein C19orf18	C19orf18			
Q6PB30	Putative chondrosarcoma-associated gene 1 protein	CSAG1			

Q6UUV7	CREB-regulated transcription coactivator 3	CRTC3			
Q94886	CSC1-like protein 1	TMEM63A			
Q5HYN5	Cancer/testis antigen family 45 member A1	CT45A1			
A0A1B0GV90	Cortexin domain containing 2	CTXND2			
P58513	Putative uncharacterized protein encoded by LINC00158	LINC00158			
P0CG41	cTAGE family member 8	CTAGE8			
P49682	C-X-C chemokine receptor type 3	CXCR3			
Q6ZRH7	Cation channel sperm-associated protein subunit gamma	CATSPERG			
Q8WZ74	Cortactin-binding protein 2	CTTNBP2			
Q01524	Defensin-6	DEFA6			
Q9UJV9	Probable ATP-dependent RNA helicase DDX41	DDX41			
P43146	Netrin receptor DCC	DCC			
Q9NV06	DDB1- and CUL4-associated factor 13	DCAF13			
Q5H9S7	DDB1- and CUL4-associated factor 17	DCAF17			
P09417	Dihydropteridine reductase	QDPR			
Q9NPF5	DNA methyltransferase 1-associated protein 1	DMAP1			
A0A087WX78	Putative elongin-A3 member C	ELOA3CP			
O00472	RNA polymerase II elongation factor ELL2	ELL2			
Q9Y6C2	EMILIN-1	EMILIN1			
Q8N9N8	Probable RNA-binding protein EIF1AD	EIF1AD			
B6SEH9	Endogenous retrovirus group V member 2 Env polyprotein	ERVV-2			
Q96HE7	ERO1-like protein alpha	ERO1A			
H0Y354	Protein FAM72C	FAM72C			
Q5VT40	Protein FAM78B	FAM78B			
Q9H790	Exonuclease V	EXO5			
Q5VT28	Protein FAM27A/B/C	FAM27B			
P12104	Fatty acid-binding protein, intestinal	FABP2			
A8MVW0	Protein FAM171A2	FAM171A2			
Q8N9Y4	Protein FAM181A	FAM181A			
A6NEQ2	Protein FAM181B	FAM181B			
Q6ZVS7	Protein FAM183BP	FAM183BP			
Q5T1J6	Protein FAM182A	FAM182A			
Q9ULE4	Protein FAM184B	FAM184B			
Q86XD5	Protein FAM131B	FAM131B			
Q9Y3B2	Exosome complex component CSL4	EXOSC1			
P0CG42	Putative protein FAM157B	FAM157B			
Q96C01	Protein FAM136A	FAM136A			
Q92567	Protein FAM168A	FAM168A			
Q6ZU69	Protein FAM205A	FAM205A			
P0C7A2	Protein FAM153B	FAM153B			
Q8WU58	Protein FAM222B	FAM222B			
B9A014	Protein FAM243A	FAM243A			
A0A494C0N9	Protein FAM246B	FAM246B			
Q8NEV8	Exophilin-5	EXPH5			
Q96PY5	Formin-like protein 2	FMNL2			
Q96RJ6	Fer3-like protein	FERD3L			
Q9HCM7	Fibrosin-1-like protein	FBRSL1			
O75072	Fukutin	FKTN			
Q7Z7B0	Filamin-A-interacting protein 1	FILIP1			
A8MYX2	Protein FAM25E	FAM25E			
Q9UPI3	Feline leukemia virus subgroup C receptor-related protein 2	FLVCR2			
Q6P4F2	Ferredoxin-2, mitochondrial	FDX2			
A6NMB9	Fidgetin-like protein 2	FIGNL2			

Q9NP95	Fibroblast growth factor 20	FGF20			
P23142	Fibulin-1	FBLN1			
Q13480	GRB2-associated-binding protein 1	GAB1			
Q06547	GA-binding protein subunit beta-1	GABPB1			
Q9UN86	Ras GTPase-activating protein-binding protein 2	G3BP2			
Q92837	Proto-oncogene FRAT1	FRAT1			
Q68CZ1	Protein fantom	RPGRIP1L			
Q9NU39	Forkhead box protein D4-like 1	FOXD4L1			
Q96CP2	FLYWCH family member 2	FLYWCH2			
Q9UP38	Frizzled-1	FZD1			
P23945	Follicle-stimulating hormone receptor	FSHR			
O60478	Integral membrane protein GPR137B	GPR137B			
Q8NB37	Glutamine amidotransferase-like class 1 domain-containing protein 1	GATD1			
Q92949	Forkhead box protein J1	FOXJ1			
P01350	Gastrin	GAST			
Q14353	Guanidinoacetate N-methyltransferase	GAMT			
Q8IVS8	Glycerate kinase	GLYCTK			
Q9UK05	Growth/differentiation factor 2	GDF2			
Q86XX4	Extracellular matrix organizing protein FRAS1	FRAS1			
Q99758	ATP-binding cassette sub-family A member 3	ABCA3			
Q8N139	ATP-binding cassette sub-family A member 6	ABCA6			
Q9H172	ATP-binding cassette sub-family G member 4	ABCG4			
Q3KP44	Ankyrin repeat domain-containing protein 55	ANKRD55			
P61966	AP-1 complex subunit sigma-1A	AP1S1			
P30411	B2 bradykinin receptor	BDKRB2			
P35226	Polycomb complex protein BMI-1	BMI1			
Q96B45	BLOC-1-related complex subunit 7	BORCS7			
Q12982	BCL2/adenovirus E1B 19 kDa protein-interacting protein 2	BNIP2			
Q8WXS3	Brain and acute leukemia cytoplasmic protein	BAALC			
Q9Y6D6	Brefeldin A-inhibited guanine nucleotide-exchange protein 1	ARFGEF1			
O60756	Putative protein BCE-1	BCE1			
O14867	Transcription regulator protein BACH1	BACH1			
P35218	Carbonic anhydrase 5A, mitochondrial	CA5A			
O60840	Voltage-dependent L-type calcium channel subunit alpha-1F	CACNA1F			
P55286	Cadherin-8	CDH8			
Q9Y6N8	Cadherin-10	CDH10			
Q9UBX1	Cathepsin F	CTSF			
Q5VVM6	Coiled-coil domain-containing protein 30	CCDC30			
Q96MW1	Coiled-coil domain-containing protein 43	CCDC43			
P56539	Caveolin-3	CAV3			
Q5T0F9	Coiled-coil and C2 domain-containing protein 1B	CC2D1B			
Q4AC94	C2 domain-containing protein 3	C2CD3			
Q16581	C3a anaphylatoxin chemotactic receptor	C3AR1			
Q8IWF9	Coiled-coil domain-containing protein 83	CCDC83			

Q8TD86	Calmodulin-like protein 6	CALML6			
Q8WUD4	Coiled-coil domain-containing protein 12	CCDC12			
Q96MT4	Uncharacterized protein encoded by LINC01600	LINC01600			
P10147	C-C motif chemokine 3	CCL3			
A6NI87	Protein chibby homolog 3	CBY3			
Q99626	Homeobox protein CDX-2	CDX2			
A4D0Y5	Uncharacterized protein C7orf77	C7orf77			
Q15078	Cyclin-dependent kinase 5 activator 1	CDK5R1			
Q6ZMN8	Cyclin-I2	CCNI2			
Q8WWL7	G2/mitotic-specific cyclin-B3	CCNB3			
Q9H305	Cell death-inducing p53-target protein 1	CDIP1			
Q8IY42	Uncharacterized protein C4orf19	C4orf19			
Q5EBM0	UMP-CMP kinase 2, mitochondrial	CMPK2			
Q8IXP5	Uncharacterized protein C11orf53	C11orf53			
Q14008	Cytoskeleton-associated protein 5	CKAP5			
Q9HBJ8	Collectrin	CLTRN			
Q9NSE2	Cytokine-inducible SH2-containing protein	CISH			
Q8IZC6	Collagen alpha-1	COL27A1			
Q8WXD9	Caskin-1	CASKIN1			
Q8WXF5	Gamma-crystallin N	CRYGN			
Q96J86	Cysteine and tyrosine-rich protein 1	CYYR1			
P36383	Gap junction gamma-1 protein	GJC1			
Q5SY80	Cation channel sperm-associated protein subunit epsilon	CATSPERE			
Q96F07	Cytoplasmic FMR1-interacting protein 2	CYFIP2			
Q2M2W7	UPF0450 protein C17orf58	C17orf58			
Q5M775	Cytospin-B	SPECC1			
P0C864	Putative uncharacterized protein DANCR	DANCR			
Q9H773	dCTP pyrophosphatase 1	DCTPP1			
Q9NQI0	Probable ATP-dependent RNA helicase DDX4	DDX4			
O95786	Antiviral innate immune response receptor RIG-I	DDX58			
Q86Y01	E3 ubiquitin-protein ligase DTX1	DTX1			
Q9BZQ6	ER degradation-enhancing alpha-mannosidase-like protein 3	EDEM3			
P61565	Endogenous retrovirus group K member 21 Env polyprotein	ERVK-21			
P29323	Ephrin type-B receptor 2	EPHB2			
Q9BUP0	EF-hand domain-containing protein D1	EFHD1			
Q2NKX8	DNA excision repair protein ERCC-6-like	ERCC6L			
Q8N693	Homeobox protein ESX1	ESX1			
P12259	Coagulation factor V	F5			
Q86X60	Protein FAM72B	FAM72B			
Q7L8L6	FAST kinase domain-containing protein 5, mitochondrial	FASTKD5			
Q96GI7	Protein FAM89A	FAM89A			
P05413	Fatty acid-binding protein, heart	FABP3			
Q9BSJ8	Extended synaptotagmin-1	ESYT1			
Q5VUB5	Protein FAM171A1	FAM171A1			
Q96A65	Exocyst complex component 4	EXOC4			
Q9BVV8	Protein FAM174C	FAM174C			
Q9NQT4	Exosome complex component RRP46	EXOSC5			
Q99714	3-hydroxyacyl-CoA dehydrogenase type-2	HSD17B10			
Q8N8A8	Protein FAM169B	FAM169B			
Q8IW50	Protein FAM219A	FAM219A			
O95925	Eppin	EPPIN			
Q96D05	Protein FAM241B	FAM241B			

A0A1B0GTK5	Protein FAM236D	FAM236D			
Q8TF40	Folliculin-interacting protein 1	FNIP1			
Q8TBE3	Fibronectin type III domain-containing protein 9	FNDC9			
P30273	High affinity immunoglobulin epsilon receptor subunit gamma	FCER1G			
O43155	Leucine-rich repeat transmembrane protein FLRT2	FLRT2			
Q14254	Flotillin-2	FLOT2			
Q8IXS0	Protein FAM217A	FAM217A			
P57775	F-box/WD repeat-containing protein 4	FBXW4			
Q6ZV73	FYVE, RhoGEF and PH domain-containing protein 6	FGD6			
Q9Y3D6	Mitochondrial fission 1 protein	FIS1			
Q8TED4	Glucose-6-phosphate exchanger SLC37A2	SLC37A2			
O95967	EGF-containing fibulin-like extracellular matrix protein 2	EFEMP2			
Q8NHY3	GAS2-like protein 2	GAS2L2			
Q6ZUT3	FERM domain-containing protein 7	FRMD7			
P19883	Follistatin	FST			
O75593	Forkhead box protein H1	FOXH1			
Q9BWX5	Transcription factor GATA-5	GATA5			
Q14CM0	FERM and PDZ domain-containing protein 4	FRMPD4			
Q8WU20	Fibroblast growth factor receptor substrate 2	FRS2			
O60755	Galanin receptor type 3	GALR3			
O14772	Fucose-1-phosphate guanylyltransferase	FPGT			
Q9UKT7	F-box/LRR-repeat protein 3	FBXL3			
Q86UU5	Gametogenetin	GGN			
P21462	fMet-Leu-Phe receptor	FPR1			
P41250	Glycine--tRNA ligase	GARS1			
P0DJR0	GTPase IMAP family member GIMD1	GIMD1			
P52306	Rap1 GTPase-GDP dissociation stimulator 1	RAP1GDS1			
Q9NR23	Growth/differentiation factor 3	GDF3			
Q99988	Growth/differentiation factor 15	GDF15			
P50150	Guanine nucleotide-binding protein G	GNG4			
P50440	Glycine amidinotransferase, mitochondrial	GATM			
P10144	Granzyme B	GZMB			
P18505	Gamma-aminobutyric acid receptor subunit beta-1	GABRB1			
Q8WWP7	GTPase IMAP family member 1	GIMAP1			
Q14410	Glycerol kinase 2	GK2			
Q14789	Golgin subfamily B member 1	GOLGB1			
F8WBI6	Golgin subfamily A member 8N	GOLGA8N			
Q7Z602	Probable G-protein coupled receptor 141	GPR141			
P19086	Guanine nucleotide-binding protein G	GNAZ			
Q9H4A5	Golgi phosphoprotein 3-like	GOLPH3L			
P55087	Aquaporin-4	AQP4			
Q9ULI0	ATPase family AAA domain-containing protein 2B	ATAD2B			
Q8NHP1	Aflatoxin B1 aldehyde reductase member 4	AKR7L			
Q9P2S6	Ankyrin repeat and MYND domain-containing protein 1	ANKMY1			
P26436	Acrosomal protein SP-10	ACRV1			
Q9NR71	Neutral ceramidase	ASAH2			
O75129	Astrotactin-2	ASTN2			

Q96FT7	Acid-sensing ion channel 4	ASIC4			
Q86Y29	B melanoma antigen 3	BAGE3			
P57796	Calcium-binding protein 4	CABP4			
A0A1B0GVN3	Uncharacterized protein C2orf92	C2orf92			
Q0P641	Uncharacterized protein C2orf80	C2orf80			
Q5VVS0	Putative uncharacterized protein C1orf140	C1orf140			
Q6ZSB3	Putative uncharacterized protein encoded by LINC00299	LINC00299			
P55287	Cadherin-11	CDH11			
P0C862	Complement C1q and tumor necrosis factor-related protein 9A	C1QTNF9			
Q8N126	Cell adhesion molecule 3	CADM3			
Q96EU7	C1GALT1-specific chaperone 1	C1GALT1C			
Q8IU53	Protein CASC2, isoforms 1/2	CASC2			
Q3SXR2	Uncharacterized protein C3orf36	C3orf36			
P49069	Calcium signal-modulating cyclophilin ligand	CAMLG			
Q96GE6	Calmodulin-like protein 4	CALML4			
Q8N187	Calcium-responsive transcription factor	CARF			
Q86Z20	Coiled-coil domain-containing protein 125	CCDC125			
Q5BJE1	Coiled-coil domain-containing protein 178	CCDC178			
Q96M89	Coiled-coil domain-containing protein 138	CCDC138			
A6NJW9	T-cell surface glycoprotein CD8 beta-2 chain	CD8B2			
O75828	Carbonyl reductase	CBR3			
Q8WUD6	Cholinephosphotransferase 1	CHPT1			
P51684	C-C chemokine receptor type 6	CCR6			
A6NDU8	UPF0600 protein C5orf51	C5orf51			
Q9NPF2	Carbohydrate sulfotransferase 11	CHST11			
Q9H2X0	Chordin	CHRD			
Q9NYQ7	Cadherin EGF LAG seven-pass G-type receptor 3	CELSR3			
O14647	Chromodomain-helicase-DNA-binding protein 2	CHD2			
Q5T6M2	Putative uncharacterized protein encoded by LINC00242	LINC00242			
Q9H8M5	Metal transporter CNNM2	CNNM2			
Q3C1V1	Uncharacterized protein C11orf91	C11orf91			
P28329	Choline O-acetyltransferase	CHAT			
Q6NUJ2	Uncharacterized protein C11orf87	C11orf87			
Q8IZV2	CKLF-like MARVEL transmembrane domain-containing protein 8	CMTM8			
Q99788	Chemokine-like receptor 1	CMKLR1			
Q5T681	Uncharacterized protein C10orf62	C10orf62			
O15247	Chloride intracellular channel protein 2	CLIC2			
O00299	Chloride intracellular channel protein 1	CLIC1			
Q96CD2	Phosphopantothenoylcysteine decarboxylase	PPCDC			
Q9NZJ6	Ubiquinone biosynthesis O-methyltransferase, mitochondrial	COQ3			
P21964	Catechol O-methyltransferase	COMT			
O75208	Ubiquinone biosynthesis protein COQ9, mitochondrial	COQ9			
Q02318	Sterol 26-hydroxylase, mitochondrial	CYP27A1			
Q8WW18	Uncharacterized protein C17orf50	C17orf50			
Q8N2A0	Putative uncharacterized protein encoded by LINC00269	LINC00269			
P42830	C-X-C motif chemokine 5	CXCL5			
Q9NYP8	Uncharacterized protein C21orf62	C21orf62			

O43310	CBP80/20-dependent translation initiation factor	CTIF			
Q9P0U4	CXXC-type zinc finger protein 1	CXXC1			
Q8IWA5	Choline transporter-like protein 2	SLC44A2			
Q8NET1	Beta-defensin 108B	DEFB108B			
Q8WTQ1	Beta-defensin 104	DEFB104A			
O60759	Cytohesin-interacting protein	CYTIP			
Q86WR6	Uncharacterized protein C17orf64	C17orf64			
Q5TCH4	Cytochrome P450 4A22	CYP4A22			
Q9Y442	Uncharacterized protein C22orf24	C22orf24			
Q8IWT3	Cullin-9	CUL9			
Q00796	Sorbitol dehydrogenase	SORD			
O43598	2'-deoxynucleoside 5'-phosphate N-hydrolase 1	DNPH1			
O00115	Deoxyribonuclease-2-alpha	DNASE2			
Q96T75	Down syndrome critical region protein 8	DSCR8			
P56179	Homeobox protein DLX-6	DLX6			
Q8TDB6	E3 ubiquitin-protein ligase DTX3L	DTX3L			
Q6P1R4	tRNA-dihydrouridine	DUS1L			
Q9UFH2	Dynein heavy chain 17, axonemal	DNAH17			
Q9UBX2	Double homeobox protein 4	DUX4			
P0DN84	Sarcoplasmic/endoplasmic reticulum calcium ATPase regulator DWORF	STRIT1			
Q9NX74	tRNA-dihydrouridine	DUS2			
Q9UGP5	DNA polymerase lambda	POLL			
Q14209	Transcription factor E2F2	E2F2			
O75923	Dysferlin	DYSF			
Q56P03	E2F-associated phosphoprotein	EAPP			
O95672	Endothelin-converting enzyme-like 1	ECEL1			
Q9NPB8	Glycerophosphocholine phosphodiesterase GPCPD1	GPCPD1			
Q8NEU8	DCC-interacting protein 13-beta	APPL2			
Q9Y227	Ectonucleoside triphosphate diphosphohydrolase 4	ENTPD4			
Q15006	ER membrane protein complex subunit 2	EMC2			
Q8IZ81	ELMO domain-containing protein 2	ELMOD2			
O95954	Formimidoyltransferase-cyclodeaminase	FTCD			
P01100	Proto-oncogene c-Fos	FOS			
Q9P0K8	Forkhead box protein J2	FOXJ2			
Q8IVH2	Forkhead box protein P4	FOXP4			
Q9UM11	Fizzy-related protein homolog	FZR1			
O43603	Galanin receptor type 2	GALR2			
P51570	Galactokinase	GALK1			
Q9NY28	Probable polypeptide N-acetylgalactosaminyltransferase 8	GALNT8			
P02728	Erythrocyte membrane glycopeptide				
A6NEF3	Golgin subfamily A member 6-like protein 4	GOLGA6L4			
P48060	Glioma pathogenesis-related protein 1	GLIPR1			
O00144	Frizzled-9	FZD9			
O15228	Dihydroxyacetone phosphate acyltransferase	GNPAT			
Q8N1C3	Gamma-aminobutyric acid receptor subunit gamma-1	GABRG1			
Q92805	Golgin subfamily A member 1	GOLGA1			
P25092	Heat-stable enterotoxin receptor	GUCY2C			
Q9BQ67	Glutamate-rich WD repeat-containing protein 1	GRWD1			
P09211	Glutathione S-transferase P	GSTP1			
Q8N442	Translation factor GUF1, mitochondrial	GUF1			
P69892	Hemoglobin subunit gamma-2	HBG2			

O00222	Metabotropic glutamate receptor 8	GRM8			
Q8IZA3	Histone H1.8	H1-8			
Q92522	Histone H1.10	H1-10			
Q9P298	HIG1 domain family member 1B	HIGD1B			
A8MXZ3	Keratin-associated protein 9-1	KRTAP9-1			
P15515	Histatin-1	HTN1			
P54198	Protein HIRA	HIRA			
O43364	Homeobox protein Hox-A2	HOXA2			
Q92902	Hermansky-Pudlak syndrome 1 protein	HPS1			
Q8TDB4	Protein MGARP	MGARP			
Q9Y278	Heparan sulfate glucosamine 3-O-sulfotransferase 2	HS3ST2			
P25021	Histamine H2 receptor	HRH2			
Q14541	Hepatocyte nuclear factor 4-gamma	HNF4G			
P01570	Interferon alpha-14	IFNA14			
Q5T764	Interferon-induced protein with tetratricopeptide repeats 1B	IFIT1B			
Q96LB3	Intraflagellar transport protein 74 homolog	IFT74			
P0DOX2	Immunoglobulin alpha-2 heavy chain				
P24001	Interleukin-32	IL32			
Q9H9L3	Interferon-stimulated 20 kDa exonuclease-like 2	ISG20L2			
Q13576	Ras GTPase-activating-like protein IQGAP2	IQGAP2			
P35908	Keratin, type II cytoskeletal 2 epidermal	KRT2			
Q9H0B3	IQ domain-containing protein N	IQCN			
P0C7M6	IQ domain-containing protein F3	IQCF3			
O60271	C-Jun-amino-terminal kinase-interacting protein 4	SPAG9			
Q96AA8	Janus kinase and microtubule-interacting protein 2	JAKMIP2			
Q5JSJ4	Integrator complex subunit 6-like	INTS6L			
Q6ZRS4	Protein ITPRID1	ITPRID1			
A4D126	D-ribitol-5-phosphate cytidylyltransferase	CRPPA			
O00400	Acetyl-coenzyme A transporter 1	SLC33A1			
Q8NC06	Acyl-CoA-binding domain-containing protein 4	ACBD4			
Q13085	Acetyl-CoA carboxylase 1	ACACA			
P02708	Acetylcholine receptor subunit alpha	CHRNA1			
Q5SW96	Low density lipoprotein receptor adapter protein 1	LDLRAP1			
Q9GZV1	Ankyrin repeat domain-containing protein 2	ANKRD2			
P56211	cAMP-regulated phosphoprotein 19	ARPP19			
Q8TEP8	Centrosomal protein of 192 kDa	CEP192			
Q8IWY9	Codanin-1	CDAN1			
P29017	T-cell surface glycoprotein CD1c	CD1C			
Q5T655	Cilia- and flagella-associated protein 58	CFAP58			
Q15762	CD226 antigen	CD226			
Q9UFW8	CGG triplet repeat-binding protein 1	CGGBP1			
O43529	Carbohydrate sulfotransferase 10	CHST10			
Q6UWT4	Uncharacterized protein C5orf46	C5orf46			
Q96BP2	Coiled-coil-helix-coiled-coil-helix domain-containing protein 1	CHCHD1			
Q9H2A9	Carbohydrate sulfotransferase 8	CHST8			
P29965	CD40 ligand	CD40LG			
D6RIA3	Uncharacterized protein C4orf54	C4orf54			

Q9UKZ1	CCR4-NOT transcription complex subunit 11	CNOT11			
Q99966	Cbp/p300-interacting transactivator 1	CITED1			
Q8WW14	Uncharacterized protein C10orf82	C10orf82			
Q9UJS0	Calcium-binding mitochondrial carrier protein Aralar2	SLC25A13			
P10645	Chromogranin-A	CHGA			
Q9NSA3	Beta-catenin-interacting protein 1	CTNNBIP1			
Q9UHP7	C-type lectin domain family 2 member D	CLEC2D			
Q8N365	Circadian-associated transcriptional repressor	CIART			
Q9UGN4	CMRF35-like molecule 8	CD300A			
O60543	Cell death activator CIDE-A	CIDEA			
Q96S66	Chloride channel CLIC-like protein 1	CLCC1			
Q01094	Transcription factor E2F1	E2F1			
P50570	Dynamin-2	DNM2			
P51800	Chloride channel protein ClC-Ka	CLCNKA			
P51801	Chloride channel protein ClC-Kb	CLCNKB			
Q8IZS7	C-type lectin-like domain family 1	CLECL1			
P58418	Clarin-1	CLRN1			
O00716	Transcription factor E2F3	E2F3			
Q8N1N2	Dynactin-associated protein	DYNAP			
Q9BZG8	-2	DPH1			
Q30154	HLA class II histocompatibility antigen, DR beta 5 chain	HLA-DRB5			
Q16254	Transcription factor E2F4	E2F4			
Q86YN1	Dolichyldiphosphatase 1	DOLPP1			
Q96GX8	Uncharacterized protein C16orf74	C16orf74			
Q13011	Delta	ECH1			
P42658	Dipeptidyl aminopeptidase-like protein 6	DPP6			
Q6V1X1	Dipeptidyl peptidase 8	DPP8			
P40939	Trifunctional enzyme subunit alpha, mitochondrial	HADHA			
Q86TI2	Dipeptidyl peptidase 9	DPP9			
Q7Z7J5	Developmental pluripotency-associated protein 2	DPPA2			
Q9HCS5	Band 4.1-like protein 4A	EPB41L4A			
Q6W0C5	Developmental pluripotency-associated protein 3	DPPA3			
Q9H329	Band 4.1-like protein 4B	EPB41L4B			
P55084	Trifunctional enzyme subunit beta, mitochondrial	HADHB			
Q7L190	Developmental pluripotency-associated protein 4	DPPA4			
A6NC42	Developmental pluripotency-associated 5 protein	DPPA5			
Q66K89	Transcription factor E4F1	E4F1			
Q9NTX5	Ethylmalonyl-CoA decarboxylase	ECHDC1			
Q8TEK3	Histone-lysine N-methyltransferase, H3 lysine-79 specific	DOT1L			
O43303	Centriolar coiled-coil protein of 110 kDa	CCP110			
Q494V2	Cilia- and flagella-associated protein 100	CFAP100			
O75521	Enoyl-CoA delta isomerase 2	ECI2			
Q1HG43	Dual oxidase maturation factor 1	DUOXA1			
P0CF75	Endogenous Bornavirus-like nucleoprotein 1	EBLN1			
P24311	Cytochrome c oxidase subunit 7B, mitochondrial	COX7B			
Q9UMD9	Collagen alpha-1	COL17A1			
P05813	Beta-crystallin A3	CRYBA1			

Q6UWV6	Ectonucleotide pyrophosphatase/phosphodiesterase family member 7	ENPP7			
Q9NZV1	Cysteine-rich motor neuron 1 protein	CRIM1			
Q03060	cAMP-responsive element modulator	CREM			
Q9H8M1	Coenzyme Q-binding protein COQ10 homolog B, mitochondrial	COQ10B			
Q9Y5Q5	Atrial natriuretic peptide-converting enzyme	CORIN			
O15528	25-hydroxyvitamin D-1 alpha hydroxylase, mitochondrial	CYP27B1			
Q02930	Cyclic AMP-responsive element-binding protein 5	CREB5			
Q9ULV4	Coronin-1C	CORO1C			
Q9NY93	Probable ATP-dependent RNA helicase DDX56	DDX56			
Q8N568	Serine/threonine-protein kinase DCLK2	DCLK2			
P17707	S-adenosylmethionine decarboxylase proenzyme	AMD1			
Q9BTZ2	Dehydrogenase/reductase SDR family member 4	DHRS4			
Q9P2K9	Protein dispatched homolog 3	DISP3			
Q16828	Dual specificity protein phosphatase 6	DUSP6			
P49005	DNA polymerase delta subunit 2	POLD2			
Q3SY89	Elongin-A3 member B	ELOA3BP			
Q5JZY3	Ephrin type-A receptor 10	EPHA10			
P19419	ETS domain-containing protein Elk-1	ELK1			
Q9P0I2	ER membrane protein complex subunit 3	EMC3			
Q9H9T3	Elongator complex protein 3	ELP3			
O75715	Epididymal secretory glutathione peroxidase	GPX5			
Q96D98	EP300-interacting inhibitor of differentiation 2B	EID2B			
P41439	Folate receptor gamma	FOLR3			
Q8N2G8	GH3 domain-containing protein	GHDC			
Q7Z4P5	Growth/differentiation factor 7	GDF7			
Q96MZ0	Ganglioside-induced differentiation-associated protein 1-like 1	GDAP1L1			
Q9BZD3	Putative GRINL1B complex locus protein 2	GCOM2			
Q9UJJ9	N-acetylglucosamine-1-phosphotransferase subunit gamma	GNPTG			
Q9NZ38	Uncharacterized protein IDI2-AS1	IDI2-AS1			
O95249	Golgi SNAP receptor complex member 1	GOSR1			
H3BQL2	Golgin subfamily A member 8T	GOLGA8T			
O95837	Guanine nucleotide-binding protein subunit alpha-14	GNA14			
A7E2F4	Golgin subfamily A member 8A	GOLGA8A			
Q8N0V5	N-acetyllactosaminide beta-1,6-N-acetylglucosaminyl-transferase	GCNT2			
Q6NV75	Probable G-protein coupled receptor 153	GPR153			
Q96P69	G-protein coupled receptor 78	GPR78			
O00178	GTP-binding protein 1	GTPBP1			
Q7LDG7	RAS guanyl-releasing protein 2	RASGRP2			
Q9P0R6	GSK3B-interacting protein	GSKIP			
Q99877	Histone H2B type 1-N	H2BC15			
Q14416	Metabotropic glutamate receptor 2	GRM2			
Q93077	Histone H2A type 1-C	H2AC6			

Q13547	Histone deacetylase 1	HDAC1			
P13716	Delta-aminolevulinic acid dehydratase	ALAD			
Q9P2D3	HEAT repeat-containing protein 5B	HEATR5B			
A0A0B4J1V2	Immunoglobulin heavy variable 2-26	IGHV2-26			
Q96IR7	4-hydroxyphenylpyruvate dioxygenase-like protein	HPDL			
O75506	Heat shock factor-binding protein 1	HSBP1			
Q00613	Heat shock factor protein 1	HSF1			
P78318	Immunoglobulin-binding protein	IGBP1			
O75409	Huntingtin-interacting protein M	H2AP			
P0CF74	Immunoglobulin lambda constant 6	IGLC6			
A0M8Q6	Immunoglobulin lambda constant 7	IGLC7			
Q8IZA0	Dyslexia-associated protein KIAA0319-like protein	KIAA0319L			
Q7Z3Y8	Keratin, type I cytoskeletal 27	KRT27			
Q9NQX7	Integral membrane protein 2C	ITM2C			
Q9BT40	Inositol polyphosphate 5-phosphatase K	INPP5K			
P26006	Integrin alpha-3	ITGA3			
Q8WYH8	Inhibitor of growth protein 5	ING5			
Q99661	Kinesin-like protein KIF2C	KIF2C			
O95069	Potassium channel subfamily K member 2	KCNK2			
Q86VH2	Kinesin-like protein KIF27	KIF27			
Q9NYR9	NF-kappa-B inhibitor-interacting Ras-like protein 2	NKIRAS2			
Q4UJ75	Putative ankyrin repeat domain-containing protein 20A4	ANKRD20A4P			
O00763	Acetyl-CoA carboxylase 2	ACACB			
Q8TB40		ABHD4			
O94805	Actin-like protein 6B	ACTL6B			
Q6P6C2	RNA demethylase ALKBH5	ALKBH5			
Q6H8Q1	Actin-binding LIM protein 2	ABLIM2			
Q5T7M4	Adipolin	C1QTNF12			
Q86TB3	Alpha-protein kinase 2	ALPK2			
Q8TD06	Anterior gradient protein 3	AGR3			
Q9Y6U3	Adseverin	SCIN			
O60242	Adhesion G protein-coupled receptor B3	ADGRB3			
Q9BTT0	Acidic leucine-rich nuclear phosphoprotein 32 family member E	ANP32E			
Q9UKF2	Disintegrin and metalloproteinase domain-containing protein 30	ADAM30			
P36404	ADP-ribosylation factor-like protein 2	ARL2			
A6NC57	Ankyrin repeat domain-containing protein 62	ANKRD62			
P0DUB6	Alpha-amylase 1A	AMY1A			
P19801	Amiloride-sensitive amine oxidase	AOC1			
P25054	Adenomatous polyposis coli protein	APC			
P02649	Apolipoprotein E	APOE			
Q9Y6G5	COMM domain-containing protein 10	COMMD10			
O75629	Protein CREG1	CREG1			
P08686	Steroid 21-hydroxylase	CYP21A2			
Q9P2I0	Cleavage and polyadenylation specificity factor subunit 2	CPSF2			
Q9NYL5	24-hydroxycholesterol 7-alpha-hydroxylase	CYP39A1			
Q9NYF0	Dapper homolog 1	DACT1			
A6NMT0	Homeobox protein DBX1	DBX1			

Q6PK57	Putative GED domain-containing protein DNM1P34	DNM1P34			
Q14562	ATP-dependent RNA helicase DHX8	DHX8			
O75953	DnaJ homolog subfamily B member 5	DNAJB5			
O75190	DnaJ homolog subfamily B member 6	DNAJB6			
Q7Z6W7	DnaJ homolog subfamily B member 7	DNAJB7			
P52429	Diacylglycerol kinase epsilon	DGKE			
Q9H819	DnaJ homolog subfamily C member 18	DNAJC18			
A0A1B0GTR0	Epididymal protein 13	EDDM13			
O94769	Extracellular matrix protein 2	ECM2			
Q9Y4J8	Dystrobrevin alpha	DTNA			
Q08554	Desmocollin-1	DSC1			
Q9HCU8	DNA polymerase delta subunit 4	POLD4			
Q9H4A9	Dipeptidase 2	DPEP2			
Q9UQ16	Dynamin-3	DNM3			
Q9NW81	Distal membrane-arm assembly complex protein 2	DMAC2			
P49448	Glutamate dehydrogenase 2, mitochondrial	GLUD2			
O95057	GTP-binding protein Di-Ras1	DIRAS1			
A2CJ06	Dystrotelin	DYTN			
Q9H4A6	Golgi phosphoprotein 3	GOLPH3			
P21918	D	DRD5			
Q5JWR5	Protein dopey-1	DOP1A			
O75487	Glypican-4	GPC4			
Q86YR5	G-protein-signaling modulator 1	GPSM1			
Q9NZH0	G-protein coupled receptor family C group 5 member B	GPRC5B			
Q08426	Peroxisomal bifunctional enzyme	EHHADH			
Q96DC8	Enoyl-CoA hydratase domain-containing protein 3, mitochondrial	ECHDC3			
Q8IXK2	Polypeptide N-acetylgalactosaminyltransferase 12	GALNT12			
O71037	Endogenous retrovirus group K member 19 Env polyprotein	ERVK-19			
A6NLF2	Elongin-A3 member D	ELOA3DP			
Q9NZC4	ETS homologous factor	EHF			
Q14314	Fibroleukin	FGL2			
P58550	Putative FXYD domain-containing ion transport regulator 8	FXYD6P3			
O14976	Cyclin-G-associated kinase	GAK			
P58549	FXYD domain-containing ion transport regulator 7	FXYD7			
O75084	Frizzled-7	FZD7			
Q12952	Forkhead box protein L1	FOXL1			
P10912	Growth hormone receptor	GHR			
Q92990	Glomulin	GLMN			
Q6W3E5	Glycerophosphodiester phosphodiesterase domain-containing protein 4	GDPD4			
P48728	Aminomethyltransferase, mitochondrial	AMT			
A0A1B0GV03	Golgin subfamily A member 6-like protein 7	GOLGA6L7			
Q7L5D6	Golgi to ER traffic protein 4 homolog	GET4			
P34903	Gamma-aminobutyric acid receptor subunit alpha-3	GABRA3			
O75791	GRB2-related adapter protein 2	GRAP2			
P32019	Type II inositol 1,4,5-trisphosphate 5-phosphatase	INPP5B			

Q14449	Growth factor receptor-bound protein 14	GRB14			
Q49A26	Putative oxidoreductase GLYR1	GLYR1			
O60547	GDP-mannose 4,6 dehydratase	GMDS			
Q53EU6	Glycerol-3-phosphate acyltransferase 3	GPAT3			
Q8N158	Glypican-2	GPC2			
Q86SX6	Glutaredoxin-related protein 5, mitochondrial	GLRX5			
A6NFK2	Glutaredoxin domain-containing cysteine-rich protein 2	GRXCR2			
Q9NYM4	Probable G-protein coupled receptor 83	GPR83			
P68871	Hemoglobin subunit beta	HBB			
Q9BXL5	Hemogen	HEMGN			
Q5GLZ8	Probable E3 ubiquitin-protein ligase HERC4	HERC4			
Q8IYA8	Interactor of HORMAD1 protein 1	IHO1			
Q5DX21	Immunoglobulin superfamily member 11	IGSF11			
Q9H252	Potassium voltage-gated channel subfamily H member 6	KCNH6			
P43627	Killer cell immunoglobulin-like receptor 2DL2	KIR2DL2			
O15554	Intermediate conductance calcium-activated potassium channel protein 4	KCNN4			
P78368	Casein kinase I isoform gamma-2	CSNK1G2			
P59990	Keratin-associated protein 12-1	KRTAP12-1			
Q92845	Kinesin-associated protein 3	KIFAP3			
Q9BYR4	Keratin-associated protein 4-3	KRTAP4-3			
Q8NCM2	Potassium voltage-gated channel subfamily H member 5	KCNH5			
Q16322	Potassium voltage-gated channel subfamily A member 10	KCNA10			
Q9Y4C1	Lysine-specific demethylase 3A	KDM3A			
O15229	Kynurenine 3-monooxygenase	KMO			
A8MUP2	Citrate synthase-lysine N-methyltransferase CSKMT, mitochondrial	CSKMT			
Q9UNU6	7-alpha-hydroxycholest-4-en-3-one 12-alpha-hydroxylase	CYP8B1			
Q1MSJ5	Centrosome and spindle pole-associated protein 1	CSPP1			
Q8IVE0	Putative ciliary rootlet coiled-coil protein-like 2 protein	CROCCP3			
A4D2H0	cTAGE family member 15	CTAGE15			
Q16619	Cardiotrophin-1	CTF1			
P49711	Transcriptional repressor CTCF	CTCF			
P78358	Cancer/testis antigen 1	CTAG1A			
O95715	C-X-C motif chemokine 14	CXCL14			
Q7Z388	Probable C-mannosyltransferase DPY19L4	DPY19L4			
P35663	Cylicin-1	CYLC1			
Q8TB03	Uncharacterized protein CXorf38	CXorf38			
P04839	Cytochrome b-245 heavy chain	CYBB			
Q9NRR1	Cytokine-like protein 1	CYTL1			
Q86UP6	CUB and zona pellucida-like domain-containing protein 1	CUZD1			
P28325	Cystatin-D	CST5			
Q9NQM4	Dynein assembly factor 6, axonemal	DNAAF6			
Q5VZ89	DENN domain-containing protein 4C	DENND4C			
Q16531	DNA damage-binding protein 1	DDB1			
Q9BTE1	Dynactin subunit 5	DCTN5			
O00399	Dynactin subunit 6	DCTN6			
Q10586	D site-binding protein	DBP			

M0R2J8	Doublecortin domain-containing protein 1	DCDC1			
Q8N136	Dynein assembly factor with WDR repeat domains 1	DAW1			
A8MYV0	Doublecortin domain-containing protein 2C	DCDC2C			
Q5J5C9	Beta-defensin 121	DEFB121			
Q9BPW9	Dehydrogenase/reductase SDR family member 9	DHRS9			
Q9NRD8	Dual oxidase 2	DUOX2			
O60762	Dolichol-phosphate mannosyltransferase subunit 1	DPM1			
Q92556	Engulfment and cell motility protein 1	ELMO1			
Q9UKH3	Endogenous retrovirus group K member 9 Env polyprotein	ERVK-9			
Q9HCE0	Ectopic P granules protein 5 homolog	EPG5			
P55884	Eukaryotic translation initiation factor 3 subunit B	EIF3B			
Q96AP7	Endothelial cell-selective adhesion molecule	ESAM			
A6NNJ1	Putative protein FAM90A9P	FAM90A9P			
Q658Y4	Protein FAM91A1	FAM91A1			
A6NDY2	Putative protein FAM90A10P	FAM90A10P			
P0C5J1	Putative protein N-methyltransferase FAM86B2	FAM86B2			
O00321	ETS translocation variant 2	ETV2			
O94988	Protein FAM13A	FAM13A			
P00748	Coagulation factor XII	F12			
Q9Y603	Transcription factor ETV7	ETV7			
Q8NEG0	Protein FAM71C	FAM71C			
Q5T0W9	Protein FAM83B	FAM83B			
P15090	Fatty acid-binding protein, adipocyte	FABP4			
Q0Z7S8	Fatty acid-binding protein 9	FABP9			
Q6P6B1	Glutamate-rich protein 5	ERICH5			
A8MZ36	Envoplakin-like protein	EVPLL			
P0DPP9	Embryonic testis differentiation protein homolog B	ETDB			
Q5T319	Protein FAM182B	FAM182B			
Q96EK7	Constitutive coactivator of peroxisome proliferator-activated receptor gamma	FAM120B			
A6H8Z2	Protein FAM221B	FAM221B			
Q9Y2D4	Exocyst complex component 6B	EXOC6B			
Q9HBA9	Putative N-acetylated-alpha-linked acidic dipeptidase	FOLH1B			
P49354	Protein farnesyltransferase/geranylgeranyltransferase type-1 subunit alpha	FNTA			
P02771	Alpha-fetoprotein	AFP			
Q9UKB1	F-box/WD repeat-containing protein 11	FBXW11			
P36888	Receptor-type tyrosine-protein kinase FLT3	FLT3			
Q8TCJ0	F-box only protein 25	FBXO25			
Q9BVA6	Protein adenylyltransferase FICD	FICD			
A6NGY1	Protein FRG2-like-2	FRG2C			
P35556	Fibrillin-2	FBN2			
O95257	Growth arrest and DNA damage-inducible protein GADD45 gamma	GADD45G			
P87889	Endogenous retrovirus group K member 10 Gag polyprotein	ERVK-10			
P01358	Gastric juice peptide 1				
A6NGK3	G antigen 10	GAGE10			
P59646	FXYD domain-containing ion transport regulator 4	FXYD4			

P55316	Forkhead box protein G1	FOXG1			
Q8NHV1	GTPase IMAP family member 7	GIMAP7			
Q8NEA6	Zinc finger protein GLIS3	GLIS3			
Q8WUU5	GATA zinc finger domain-containing protein 1	GATAD1			
Q9UN88	Gamma-aminobutyric acid receptor subunit theta	GABRQ			
P46976	Glycogenin-1	GYG1			
Q9C091	GREB1-like protein	GREB1L			
Q8IZ08	G-protein coupled receptor 135	GPR135			
Q9H3M0	Potassium voltage-gated channel subfamily F member 1	KCNF1			
Q13304	Uracil nucleotide/cysteinyl leukotriene receptor	GPR17			
O15217	Glutathione S-transferase A4	GSTA4			
Q8WUI4	Histone deacetylase 7	HDAC7			
P69905	Hemoglobin subunit alpha	HBA1;			
P05546	Heparin cofactor 2	SERPIND1			
Q9BTY7	Protein HGH1 homolog	HGH1			
P14651	Homeobox protein Hox-B3	HOXB3			
P22557	5-aminolevulinate synthase, erythroid-specific, mitochondrial	ALAS2			
P86397	Hydroxyacyl-thioester dehydratase type 2, mitochondrial	HTD2			
Q8TCT9	Minor histocompatibility antigen H13	HM13			
O14558	Heat shock protein beta-6	HSPB6			
Q01581	Hydroxymethylglutaryl-CoA synthase, cytoplasmic	HMGCS1			
Q96ED9	Protein Hook homolog 2	HOOK2			
P01567	Interferon alpha-7	IFNA7			
Q13651	Interleukin-10 receptor subunit alpha	IL10RA			
P46199	Translation initiation factor IF-2, mitochondrial	MTIF2			
Q9Y3D8	Adenylate kinase isoenzyme 6	AK6			
Q96AZ6	Interferon-stimulated gene 20 kDa protein	ISG20			
Q92833	Protein Jumonji	JARID2			
Q14571	Inositol 1,4,5-trisphosphate receptor type 2	ITPR2			
Q9BZI1	Iroquois-class homeodomain protein IRX-2	IRX2			
Q9NZN1	Interleukin-1 receptor accessory protein-like 1	IL1RAPL1			
Q9UQ05	Potassium voltage-gated channel subfamily H member 4	KCNH4			
Q13118	Krueppel-like factor 10	KLF10			
P60409	Keratin-associated protein 10-7	KRTAP10-7			
O60259	Kallikrein-8	KLK8			
P17540	Creatine kinase S-type, mitochondrial	CKMT2			
Q9UJQ1	Lysosome-associated membrane glycoprotein 5	LAMP5			
A0A087WSZ0	Immunoglobulin kappa variable 1D-8	IGKV1D-8			
P11047	Laminin subunit gamma-1	LAMC1			
P08195	4F2 cell-surface antigen heavy chain	SLC3A2			
Q3I5F7	Putative acyl-coenzyme A thioesterase 6	ACOT6			
Q5SQ80	Putative ankyrin repeat domain-containing protein 20A2	ANKRD20A2P			
Q9UNA3	Alpha-1,4-N-acetylglucosaminyltransferase	A4GNT			
P02750	Leucine-rich alpha-2-	LRG1			
P28223	5-hydroxytryptamine receptor 2A	HTR2A			
P54619	5'-AMP-activated protein kinase subunit gamma-1	PRKAG1			

P35869	Aryl hydrocarbon receptor	AHR			
Q9BQI0	Allograft inflammatory factor 1-like	AIF1L			
Q9UPQ3	Arf-GAP with GTPase, ANK repeat and PH domain-containing protein 1	AGAP1			
P00352	Retinal dehydrogenase 1	ALDH1A1			
Q8N7X0	Androglobin	ADGB			
Q9H2A2	2-aminomuconic semialdehyde dehydrogenase	ALDH8A1			
Q8IY63	Angiomotin-like protein 1	AMOTL1			
Q9NQ31	A-kinase-interacting protein 1	AKIP1			
Q6UXC1	Apical endosomal glycoprotein	MAMDC4			
P43652	Afamin	AFM			
Q86SQ4	Adhesion G-protein coupled receptor G6	ADGRG6			
P12236	ADP/ATP translocase 3	SLC25A6			
O14672	Disintegrin and metalloproteinase domain-containing protein 10	ADAM10			
P18085	ADP-ribosylation factor 4	ARF4			
Q9NX46	ADP-ribose glycohydrolase ARH3	ADPRS			
Q5CZ79	Ankyrin repeat domain-containing protein 20B	ANKRD20A8P			
P53677	AP-3 complex subunit mu-2	AP3M2			
Q9NVT9	Armadillo repeat-containing protein 1	ARMC1			
Q8WWX0	Ankyrin repeat and SOCS box protein 5	ASB5			
O14525	Astrotactin-1	ASTN1			
Q96CB5	Putative uncharacterized protein C8orf44	C8orf44			
Q9Y5K6	CD2-associated protein	CD2AP			
P24863	Cyclin-C	CCNC			
O95388	CCN family member 4	CCN4			
Q9BU64	Centromere protein O	CENPO			
Q70JA7	Chondroitin sulfate synthase 3	CHSY3			
P16619	C-C motif chemokine 3-like 1	CCL3L1			
Q8IWV2	Contactin-4	CNTN4			
P78357	Contactin-associated protein 1	CNTNAP1			
Q6UXF7	C-type lectin domain family 18 member B	CLEC18B			
Q14055	Collagen alpha-2	COL9A2			
Q3B7I2	Protein canopy homolog 1	CNPY1			
P02458	Collagen alpha-1	COL2A1			
Q9H7T3	Uncharacterized protein C10orf95	C10orf95			
Q86X95	Corepressor interacting with RBPJ 1	CIR1			
Q6IBW4	Condensin-2 complex subunit H2	NCAPH2			
P12107	Collagen alpha-1	COL11A1			
O76071	Probable cytosolic iron-sulfur protein assembly protein CIAO1	CIAO1			
Q8IYS4	Uncharacterized protein C16orf71	C16orf71			
Q14CZ0	UPF0472 protein C16orf72	C16orf72			
A6NNT2	Uncharacterized protein C16orf96	C16orf96			
Q86YB7	Enoyl-CoA hydratase domain-containing protein 2, mitochondrial	ECHDC2			
Q6P2I7	Endogenous Bornavirus-like nucleoprotein 2	EBLN2			
P38432	Coilin	COIL			
A6NIL9	Putative uncharacterized protein CRYM-AS1	CRYM-AS1			
P53673	Beta-crystallin A4	CRYBA4			
Q9P299	Coatomer subunit zeta-2	COPZ2			
P39060	Collagen alpha-1	COL18A1			
O14548	Cytochrome c oxidase subunit 7A-related protein, mitochondrial	COX7A2L			
O60397	Putative cytochrome c oxidase subunit 7A3, mitochondrial	COX7A2P2			

Q7L5N1	COP9 signalosome complex subunit 6	COPS6			
Q7Z4L0	Cytochrome c oxidase subunit 8C, mitochondrial	COX8C			
P43320	Beta-crystallin B2	CRYBB2			
Q9Y2Z9	Ubiquinone biosynthesis monooxygenase COQ6, mitochondrial	COQ6			
Q96BJ8	Engulfment and cell motility protein 3	ELMO3			
Q9NUV9	GTPase IMAP family member 4	GIMAP4			
Q6P9H5	GTPase IMAP family member 6	GIMAP6			
P16520	Guanine nucleotide-binding protein G	GNB3			
P07093	Glia-derived nexin	SERPINE2			
Q9BVP2	Guanine nucleotide-binding protein-like 3	GNL3			
Q8IWJ2	GRIP and coiled-coil domain-containing protein 2	GCC2			
Q99928	Gamma-aminobutyric acid receptor subunit gamma-3	GABRG3			
P02729	Urine glycopeptide				
O95166	Gamma-aminobutyric acid receptor-associated protein	GABARAP			
O43292	Glycosylphosphatidylinositol anchor attachment 1 protein	GPAA1			
Q08378	Golgin subfamily A member 3	GOLGA3			
O14775	Guanine nucleotide-binding protein subunit beta-5	GNB5			
Q96CH1	Probable G-protein coupled receptor 146	GPR146			
Q8TDV2	Probable G-protein coupled receptor 148	GPR148			
Q8NCW6	Polypeptide N-acetylgalactosaminyltransferase 11	GALNT11			
Q9H3H5	UDP-N-acetylglucosamine--dolichyl-phosphate N-acetylglucosaminephosphotransferase	DPAGT1			
Q9Y5P6	Mannose-1-phosphate guanyltransferase beta	GMPPB			
P49019	Hydroxycarboxylic acid receptor 3	HCAR3			
P78417	Glutathione S-transferase omega-1	GSTO1			
Q8WW33	Gametocyte-specific factor 1	GTSF1			
P43250	G protein-coupled receptor kinase 6	GRK6			
Q9H116	GDNF-inducible zinc finger protein 1	GZF1			
Q6PII5	Hydroxyacylglutathione hydrolase-like protein	HAGHL			
O43593	Lysine-specific demethylase hairless	HR			
A0A2R8Y619	Histone H2B type 2-E1	H2BE1			
Q969S8	Polyamine deacetylase HDAC10	HDAC10			
Q16778	Histone H2B type 2-E	H2BC21			
Q96MH2	Protein HEXIM2	HEXIM2			
P17066	Heat shock 70 kDa protein 6	HSPA6			
P01574	Interferon beta	IFNB1			
O00458	Interferon-related developmental regulator 1	IFRD1			
P0DOX4	Immunoglobulin epsilon heavy chain				
Q6UW32	Insulin growth factor-like family member 1	IGFL1			
P20809	Interleukin-11	IL11			
P20839	Inosine-5'-monophosphate dehydrogenase 1	IMPDH1			

Q96N16	Janus kinase and microtubule-interacting protein 1	JAKMIP1			
P08727	Keratin, type I cytoskeletal 19	KRT19			
Q6A162	Keratin, type I cytoskeletal 40	KRT40			
P58062	Serine protease inhibitor Kazal-type 7	SPINK7			
P17301	Integrin alpha-2	ITGA2			
Q6P9B9	Integrator complex subunit 5	INTS5			
Q14653	Interferon regulatory factor 3	IRF3			
P48551	Interferon alpha/beta receptor 2	IFNAR2			
Q02556	Interferon regulatory factor 8	IRF8			
Q27J81	Inverted formin-2	INF2			
Q15051	IQ calmodulin-binding motif-containing protein 1	IQCB1			
P48048	ATP-sensitive inward rectifier potassium channel 1	KCNJ1			
Q99612	Krueppel-like factor 6	KLF6			
O00139	Kinesin-like protein KIF2A	KIF2A			
P49674	Casein kinase I isoform epsilon	CSNK1E			
Q17RG1	BTB/POZ domain-containing protein KCTD19	KCTD19			
Q13351	Krueppel-like factor 1	KLF1			
Q8NAX2	Keratinocyte differentiation factor 1	KDF1			
Q6ZWB6	BTB/POZ domain-containing protein KCTD8	KCTD8			
Q14678	KN motif and ankyrin repeat domain-containing protein 1	KANK1			
Q969E1	Liver-expressed antimicrobial peptide 2	LEAP2			
Q14558	Phosphoribosyl pyrophosphate synthase-associated protein 1	PRPSAP1			
Q02928	Cytochrome P450 4A11	CYP4A11			
Q5TAT6	Collagen alpha-1	COL13A1			
P06850	Corticoliberin	CRH			
P22680	Cytochrome P450 7A1	CYP7A1			
Q9Y2W7	Calsenilin	KCNIP3			
P0DMV2	Cancer/testis antigen family 45 member A9	CT45A9			
Q6XXX2	Putative uncharacterized protein encoded by LINC00114	LINC00114			
P59036	Putative uncharacterized protein encoded by LINC00310	LINC00310			
Q8NCS7	Choline transporter-like protein 5	SLC44A5			
P58505	Uncharacterized protein C21orf58	C21orf58			
P80162	C-X-C motif chemokine 6	CXCL6			
Q8IX95	Putative cTAGE family member 3	CTAGE3P			
Q8TB33	Putative uncharacterized protein encoded by LINC01560	LINC01560			
Q9H4D0	Calsyntenin-2	CLSTN2			
Q9H1C7	Cysteine-rich and transmembrane domain-containing protein 1	CYSTM1			
Q6ZUT9	DENN domain-containing protein 5B	DENND5B			
Q5VWQ8	Disabled homolog 2-interacting protein	DAB2IP			
O75935	Dynactin subunit 3	DCTN3			
Q9Y4D1	Disheveled-associated activator of morphogenesis 1	DAAM1			
Q3SXM0	DDB1- and CUL4-associated factor 4-like protein 1	DCAF4L1			
Q13409	Cytoplasmic dynein 1 intermediate chain 2	DYNC1I2			
Q9C098	Serine/threonine-protein kinase DCLK3	DCLK3			
Q8WVC6	Dephospho-CoA kinase domain-containing protein	DCAKD			
Q96PH6	Beta-defensin 118	DEFB118			

A6NHG4	D-dopachrome decarboxylase-like protein	DDTL			
Q8N687	Beta-defensin 125	DEFB125			
Q9H2U1	ATP-dependent DNA/RNA helicase DHX36	DHX36			
Q96SC8	Doublesex- and mab-3-related transcription factor A2	DMRTA2			
Q8NFT8	Delta and Notch-like epidermal growth factor-related receptor	DNER			
Q8N7S2	DnaJ homolog subfamily C member 5G	DNAJC5G			
Q6XZF7	Dynamin-binding protein	DNMBP			
Q99543	DnaJ homolog subfamily C member 2	DNAJC2			
Q14185	Dedicator of cytokinesis protein 1	DOCK1			
Q14129	Protein DGCR6	DGCR6			
Q8N2C3	DEP domain-containing protein 4	DEPDC4			
Q8IZD9	Dedicator of cytokinesis protein 3	DOCK3			
Q8IXB1	DnaJ homolog subfamily C member 10	DNAJC10			
Q9P2X0	Dolichol-phosphate mannosyltransferase subunit 3	DPM3			
P14138	Endothelin-3	EDN3			
P24530	Endothelin receptor type B	EDNRB			
Q9NYC9	Dynein heavy chain 9, axonemal	DNAH9			
P12724	Eosinophil cationic protein	RNASE3			
Q8TE73	Dynein heavy chain 5, axonemal	DNAH5			
Q6RFH8	Double homeobox protein 4C	DUX4L9			
Q6ZN92	Putative inactive deoxyuridine 5'-triphosphate nucleotidohydrolase-like protein FLJ16323				
Q6B8I1	Dual specificity protein phosphatase 13 isoform A	DUSP13			
Q8N7M0	Dynein light chain Tctex-type 5	DYNLT5			
P06746	DNA polymerase beta	POLB			
O00418	Eukaryotic elongation factor 2 kinase	EEF2K			
O75417	DNA polymerase theta	POLQ			
P61567	Endogenous retrovirus group K member 7 Env polyprotein	ERVK-7			
P41970	ETS domain-containing protein Elk-3	ELK3			
Q99613	Eukaryotic translation initiation factor 3 subunit C	EIF3C			
P54753	Ephrin type-B receptor 3	EPHB3			
P18146	Early growth response protein 1	EGR1			
Q9Y3B6	ER membrane protein complex subunit 9	EMC9			
Q9Y262	Eukaryotic translation initiation factor 3 subunit L	EIF3L			
Q9H6T0	Epithelial splicing regulatory protein 2	ESRP2			
Q6ZMW3	Echinoderm microtubule-associated protein-like 6	EML6			
Q96C19	EF-hand domain-containing protein D2	EFHD2			
Q9UJM3	ERBB receptor feedback inhibitor	ERRFI1			
Q9H501	ESF1 homolog	ESF1			
P0C7V4	Putative protein FAM90A15P	FAM90A15P			
O43414	ERI1 exoribonuclease 3	ERI3			
Q5W0A0	Glutamate-rich protein 6B	ERICH6B			
Q9NV70	Exocyst complex component 1	EXOC1			
Q9BX63	Fanconi anemia group J protein	BRIP1			
O60645	Exocyst complex component 3	EXOC3			
Q86X53	Glutamate-rich protein 1	ERICH1			
A6NC97	Putative protein FAM172B	FAM172BP			
Q70Z53	Protein FRA10AC1	FRA10AC1			
Q5T8I3	Protein FAM102B	FAM102B			

Q6P0A1	Protein FAM180B	FAM180B			
Q8WW52	Protein FAM151A	FAM151A			
Q96PV7	Protein FAM193B	FAM193B			
P0C2L3	Protein FAM163B	FAM163B			
A0A1B0GUQ0	Protein FAM236A	FAM236A			
Q4G148	Glucoside xylosyltransferase 1	GXYLT1			
P06734	Low affinity immunoglobulin epsilon Fc receptor	FCER2			
O00602	Ficolin-1	FCN1			
P31513	Dimethylaniline monooxygenase	FMO3			
Q5T1H1	Protein eyes shut homolog	EYS			
Q9UGQ2	Calcium channel flower homolog	CACFD1			
P02765	Alpha-2-HS-glycoprotein	AHSG			
O14843	Free fatty acid receptor 3	FFAR3			
O14842	Free fatty acid receptor 1	FFAR1			
Q53RD9	Fibulin-7	FBLN7			
Q6PIW4	Fidgetin-like protein 1	FIGNL1			
O75879	Glutamyl-tRNA	GATB			
Q99999	Galactosylceramide sulfotransferase	GAL3ST1			
Q5VV16	Forkhead box protein D4-like 5	FOXD4L5			
A8MYZ6	Forkhead box protein O6	FOXO6			
Q8NGA4	Putative G-protein coupled receptor GPR32P1	GPR32P1			
Q10472	Polypeptide N-acetylgalactosaminyltransferase 1	GALNT1			
Q96JP0	Protein fem-1 homolog C	FEM1C			
Q8TET4	Neutral alpha-glucosidase C	GANC			
P53539	Protein fosB	FOSB			
P30047	GTP cyclohydrolase 1 feedback regulatory protein	GCHFR			
P62873	Guanine nucleotide-binding protein G	GNB1			
Q92616	eIF-2-alpha kinase activator	GCN1			
Q02153	Guanylate cyclase soluble subunit beta-1	GUCY1B1			
O14610	Guanine nucleotide-binding protein G	GNGT2			
P19087	Guanine nucleotide-binding protein G	GNAT2			
A6NC78	Putative golgin subfamily A member 8I	GOLGA8IP			
P56915	Homeobox protein goosecoid	GSC			
Q9H1H1	Gametocyte-specific factor 1-like	GTSF1L			
Q86UQ5	Gilles de la Tourette syndrome chromosomal region candidate gene 1 protein	GTSCR1			
P16402	Histone H1.3	H1-3			
Q9H0R4	Haloacid dehalogenase-like hydrolase domain-containing protein 2	HDHD2			
Q9UJ83	2-hydroxyacyl-CoA lyase 1	HACL1			
P09105	Hemoglobin subunit theta-1	HBQ1			
Q9C0E4	Glutamate receptor-interacting protein 2	GRIP2			
Q15486	Putative inactive beta-glucuronidase-like protein SMA3	GUSBP1			
P61296	Heart- and neural crest derivatives-expressed protein 2	HAND2			
Q8WYB5	Histone acetyltransferase KAT6B	KAT6B			
Q7Z4H7	HAUS augmin-like complex subunit 6	HAUS6			
Q30201	Hereditary hemochromatosis protein	HFE			
P49773	Histidine triad nucleotide-binding protein 1	HINT1			
O94927	HAUS augmin-like complex subunit 5	HAUS5			

Q86Z02	Homeodomain-interacting protein kinase 1	HIPK1			
P14652	Homeobox protein Hox-B2	HOXB2			
Q0VDF9	Heat shock 70 kDa protein 14	HSPA14			
Q8IVU1	Immunoglobulin superfamily DCC subclass member 3	IGDCC3			
Q9UIJ7	GTP:AMP phosphotransferase AK3, mitochondrial	AK3			
Q96CB8	Integrator complex subunit 12	INTS12			
Q5VZ72	Izumo sperm-egg fusion protein 3	IZUMO3			
P26010	Integrin beta-7	ITGB7			
Q9UJP4	Kelch-like protein 21	KLHL21			
Q9NP60	X-linked interleukin-1 receptor accessory protein-like 2	IL1RAPL2			
Q2M1P5	Kinesin-like protein KIF7	KIF7			
O76015	Keratin, type I cuticular Ha8	KRT38			
Q9NSB2	Keratin, type II cuticular Hb4	KRT84			
Q9NR82	Potassium voltage-gated channel subfamily KQT member 5	KCNQ5			
P60331	Keratin-associated protein 10-1	KRTAP10-1			
Q3LI60	Keratin-associated protein 20-3	KRTAP20-3			
O76014	Keratin, type I cuticular Ha7	KRT37			
Q96G42	Kelch domain-containing protein 7B	KLHDC7B			
A0A075B6H8	Probable non-functional immunoglobulin kappa variable 1D-42	IGKV1D-42			
A0A087WW87	Immunoglobulin kappa variable 2-40	IGKV2-40			
A0A0C4DH55	Immunoglobulin kappa variable 3D-7	IGKV3D-7			
Q8IY47	Kelch repeat and BTB domain-containing protein 2	KBTBD2			
Q96PE2	Rho guanine nucleotide exchange factor 17	ARHGEF17			
Q9NZ32	Actin-related protein 10	ACTR10			
P18848	Cyclic AMP-dependent transcription factor ATF-4	ATF4			
P59826	BPI fold-containing family B member 3	BPIFB3			
Q8N3I7	Bardet-Biedl syndrome 5 protein	BBS5			
Q9H6U6	Breast carcinoma-amplified sequence 3	BCAS3			
Q9NXR7	BRISC and BRCA1-A complex member 2	BABAM2			
Q5VTR2	E3 ubiquitin-protein ligase BRE1A	RNF20			
Q6PGQ7	Protein aurora borealis	BORA			
P54687	Branched-chain-amino-acid aminotransferase, cytosolic	BCAT1			
Q9HA72	Calcium homeostasis modulator protein 2	CALHM2			
A8MX76	Calpain-14	CAPN14			
Q9NRR3	CDC42 small effector protein 2	CDC42SE2			
Q86VP6	Cullin-associated NEDD8-dissociated protein 1	CAND1			
Q6ZMU1	Putative protein C3P1	C3P1			
Q8NAE3	Putative uncharacterized protein encoded by LINC01555	LINC01555			
Q9BXJ0	Complement C1q tumor necrosis factor-related protein 5	C1QTNF5			
Q8IZS8	Voltage-dependent calcium channel subunit alpha-2/delta-3	CACNA2D3			
Q9HBT6	Cadherin-20	CDH20			
Q14444	Caprin-1	CAPRIN1			
Q07021	Complement component 1 Q subcomponent-binding protein, mitochondrial	C1QBP			
Q4VC31	Coiled-coil domain-containing protein 58	CCDC58			

Q9NS85	Carbonic anhydrase-related protein 10	CA10			
O75976	Carboxypeptidase D	CPD			
D6REC4	Cilia- and flagella-associated protein 99	CFAP99			
O00257	E3 SUMO-protein ligase CBX4	CBX4			
P05156	Complement factor I	CFI			
Q5ZPR3	CD276 antigen	CD276			
B1AMM8	Putative uncharacterized protein encoded by LINC00587	LINC00587			
Q96ST8	Centrosomal protein of 89 kDa	CEP89			
P09564	T-cell antigen CD7	CD7			
Q96NF6	Putative uncharacterized protein C8orf49	C8orf49			
Q8IV13	Cyclin-J-like protein	CCNJL			
Q8N6Q3	CD177 antigen	CD177			
Q96BR5	Cytochrome c oxidase assembly factor 7	COA7			
Q8N137	Centrobin	CNTROB			
Q9BZ76	Contactin-associated protein-like 3	CNTNAP3			
Q9UIV1	CCR4-NOT transcription complex subunit 7	CNOT7			
Q96A22	Uncharacterized protein C11orf52	C11orf52			
Q86VU5	Catechol O-methyltransferase domain-containing protein 1	COMTD1			
Q15846	Clusterin-like protein 1	CLUL1			
P21554	Cannabinoid receptor 1	CNR1			
Q5K130	Putative uncharacterized protein CLLU1-AS1	CLLU1-AS1			
Q0P5P2	Uncharacterized protein C17orf67	C17orf67			
A8MU93	Uncharacterized protein C17orf100	C17orf100			
P07315	Gamma-crystallin C	CRYGC			
Q96D53	Atypical kinase COQ8B, mitochondrial	COQ8B			
O43812	Double homeobox protein 1	DUX1			
Q9H4M9	EH domain-containing protein 1	EHD1			
P20827	Ephrin-A1	EFNA1			
Q1W209	Embryonic stem cell-related gene protein	ESRG			
Q6NT32	Carboxylesterase 5A	CES5A			
Q8NCA5	Protein FAM98A	FAM98A			
O00748	Cocaine esterase	CES2			
A8MWA6	Putative protein FAM90A22P	FAM90A22P			
Q6NZ36	Fanconi anemia core complex-associated protein 20	FAAP20			
A6NL05	Protein FAM74A7	FAM74A7			
P41161	ETS translocation variant 5	ETV5			
Q9Y282	Endoplasmic reticulum-Golgi intermediate compartment protein 3	ERGIC3			
Q96LP2	Protein FAM81B	FAM81B			
Q68DA7	Formin-1	FMN1			
Q8TES7	Fas-binding factor 1	FBF1			
Q9UK73	Protein fem-1 homolog B	FEM1B			
Q9UKT4	F-box only protein 5	FBXO5			
P11413	Glucose-6-phosphate 1-dehydrogenase	G6PD			
Q2WGJ9	Fer-1-like protein 6	FER1L6			
Q8N3F9	Integral membrane protein GPR137C	GPR137C			
Q6PJQ5	Forkhead box protein R2	FOXR2			
Q6Y7W6	GRB10-interacting GYF protein 2	GIGYF2			
O60609	GDNF family receptor alpha-3	GFRA3			
O95390	Growth/differentiation factor 11	GDF11			
Q99684	Zinc finger protein Gfi-1	GFI1			
P63096	Guanine nucleotide-binding protein G	GNAI1			

Q9HD26	Golgi-associated PDZ and coiled-coil motif-containing protein	GOPC			
P46926	Glucosamine-6-phosphate isomerase 1	GNPDA1			
A0A0U1RQE8	Putative glycine N-acyltransferase-like protein 1B	GLYATL1B			
Q5T848	Probable G-protein coupled receptor 158	GPR158			
O60234	Glia maturation factor gamma	GMFG			
Q13227	G protein pathway suppressor 2	GPS2			
P13224	Platelet glycoprotein Ib beta chain	GP1BB			
Q13439	Golgin subfamily A member 4	GOLGA4			
A4D2P6	Delphilin	GRID2IP			
Q9GZN0	Probable G-protein coupled receptor 88	GPR88			
Q86UP8	General transcription factor II-I repeat domain-containing protein 2A	GTF2IRD2			
Q15835	Rhodopsin kinase GRK1	GRK1			
Q8IUE6	Histone H2A type 2-B	H2AC21			
Q75WM6	Testis-specific H1 histone	H1-7			
Q5VWC8	Very-long-chain	HACD4			
P31267	Homeobox protein Hox-A6	HOXA6			
P01767	Immunoglobulin heavy variable 3-53	IGHV3-53			
Q96D96	Voltage-gated hydrogen channel 1	HVCN1			
P01743	Immunoglobulin heavy variable 1-46	IGHV1-46			
P01764	Immunoglobulin heavy variable 3-23	IGHV3-23			
Q96QI5	Heparan sulfate glucosamine 3-O-sulfotransferase 6	HS3ST6			
Q58FG0	Putative heat shock protein HSP 90-alpha A5	HSP90AA5P			
P01824	Immunoglobulin heavy variable 4-39	IGHV4-39			
Q9BYM8	RanBP-type and C3HC4-type zinc finger-containing protein 1	RBCK1			
P63241	Eukaryotic translation initiation factor 5A-1	EIF5A			
P08238	Heat shock protein HSP 90-beta	HSP90AB1			
A6NMD0	Interferon-induced transmembrane protein 10	IFITM10			
P52597	Heterogeneous nuclear ribonucleoprotein F	HNRNPF			
O14979	Heterogeneous nuclear ribonucleoprotein D-like	HNRNPDL			
P05013	Interferon alpha-6	IFNA6			
Q9H2S9	Zinc finger protein Eos	IKZF4			
O14920	Inhibitor of nuclear factor kappa-B kinase subunit beta	IKBKB			
Q13261	Interleukin-15 receptor subunit alpha	IL15RA			
Q6EBC2	Interleukin-31	IL31			
P0DOY2	Immunoglobulin lambda constant 2	IGLC2			
A0A1B0GVH7	IQ domain-containing protein M	IQCM			
Q7Z3Y7	Keratin, type I cytoskeletal 28	KRT28			
P78413	Iroquois-class homeodomain protein IRX-4	IRX4			
P19013	Keratin, type II cytoskeletal 4	KRT4			
O95235	Kinesin-like protein KIF20A	KIF20A			
Q9NZS2	Killer cell lectin-like receptor subfamily F member 1	KLRF1			
P01130	Low-density lipoprotein receptor	LDLR			
Q8IZD2	Inactive histone-lysine N-methyltransferase 2E	KMT2E			

Q93100	Phosphorylase b kinase regulatory subunit beta	PHKB			
P06315	Immunoglobulin kappa variable 5-2	IGKV5-2			
Q96JJ3	Engulfment and cell motility protein 2	ELMO2			
Q9UBQ5	Eukaryotic translation initiation factor 3 subunit K	EIF3K			
Q05215	Early growth response protein 4	EGR4			
P41567	Eukaryotic translation initiation factor 1	EIF1			
O76042	Putative uncharacterized protein encoded by ERC2-IT1	ERC2-IT1			
O94905	Erlin-2	ERLIN2			
Q8NE31	Protein FAM13C	FAM13C			
Q8TC56	Protein FAM71B	FAM71B			
P0C7U9	Protein FAM87A	FAM87A			
Q5TZK3	Protein FAM74A4/A6	FAM74A4			
Q00597	Fanconi anemia group C protein	FANCC			
Q6ZRV2	Protein FAM83H	FAM83H			
O15360	Fanconi anemia group A protein	FANCA			
Q8TBP5	Membrane protein FAM174A	FAM174A			
Q9UPT5	Exocyst complex component 7	EXOC7			
Q8N2X6	Uncharacterized protein EXOC3-AS1	EXOC3-AS1			
Q17R55	Protein FAM187B	FAM187B			
P78312	Protein FAM193A	FAM193A			
Q5RKV6	Exosome complex component MTR3	EXOSC6			
Q6PEV8	Protein FAM199X	FAM199X			
F5H4B4	Protein FAM227A	FAM227A			
Q96M60	Protein FAM227B	FAM227B			
A0A1B0GV22	Protein FAM236B	FAM236B			
P0DP71	Protein FAM236C	FAM236C			
Q53EP0	Fibronectin type III domain-containing protein 3B	FNDC3B			
Q96PJ5	Fc receptor-like protein 4	FCRL4			
P28799	Progranulin	GRN			
A5D6W6	Fat storage-inducing transmembrane protein 1	FITM1			
Q15007	Pre-mRNA-splicing regulator WTAP	WTAP			
Q9UKT5	F-box only protein 4	FBXO4			
Q9NVK5	FGFR1 oncogene partner 2	FGFR1OP2			
Q9BXU8	Ferritin heavy polypeptide-like 17	FTHL17			
P12034	Fibroblast growth factor 5	FGF5			
P0C091	FRAS1-related extracellular matrix protein 3	FREM3			
P35575	Glucose-6-phosphatase	G6PC			
Q9NXK8	F-box/LRR-repeat protein 12	FBXL12			
Q9BQS8	FYVE and coiled-coil domain-containing protein 1	FYCO1			
Q5TBA9	Protein furry homolog	FRY			
O43638	Forkhead box protein S1	FOXS1			
Q8NCL4	Polypeptide N-acetylgalactosaminyltransferase 6	GALNT6			
Q9HBQ8	Putative golgin subfamily A member 2B	GOLGA2P5			
Q9H3P7	Golgi resident protein GCP60	ACBD3			
P60520	Gamma-aminobutyric acid receptor-associated protein-like 2	GABARAPL2			
Q4V328	GRIP1-associated protein 1	GRIPAP1			
P0CJ92	Golgin subfamily A member 8H	GOLGA8H			
Q9NPR9	Protein GPR108	GPR108			
P11488	Guanine nucleotide-binding protein G	GNAT1			
Q16538	Probable G-protein coupled receptor 162	GPR162			

Q6NUI2	Glycerol-3-phosphate acyltransferase 2, mitochondrial	GPAT2			
Q9UI32	Glutaminase liver isoform, mitochondrial	GLS2			
Q9P107	GEM-interacting protein	GMIP			
P23415	Glycine receptor subunit alpha-1	GLRA1			
P56199	Integrin alpha-1	ITGA1			
Q9UPP5	AP2-interacting clathrin-endocytosis protein	KIAA1107			
P51674	Neuronal membrane glycoprotein M6-a	GPM6A			
Q13387	C-Jun-amino-terminal kinase-interacting protein 2	MAPK8IP2			
Q9Y283	Inversin	INVS			
P46091	G-protein coupled receptor 1	GPR1			
O15354	Prosaposin receptor GPR37	GPR37			
Q96PQ7	Kelch-like protein 5	KLHL5			
Q16836	Hydroxyacyl-coenzyme A dehydrogenase, mitochondrial	HADH			
Q71DI3	Histone H3.2	H3C15			
P28161	Glutathione S-transferase Mu 2	GSTM2			
Q7RTV2	Glutathione S-transferase A5	GSTA5			
Q99879	Histone H2B type 1-M	H2BC14			
O60443	Gasdermin-E	GSDME			
Q9UBN7	Histone deacetylase 6	HDAC6			
Q6ISB3	Grainyhead-like protein 2 homolog	GRHL2			
P42263	Glutamate receptor 3	GRIA3			
Q6ZVF9	G protein-regulated inducer of neurite outgrowth 3	GPRIN3			
P48058	Glutamate receptor 4	GRIA4			
Q9P035	Very-long-chain	HACD3			
Q8IV36	Protein HID1	HID1			
A4D0S4	Laminin subunit beta-4	LAMB4			
P08397	Porphobilinogen deaminase	HMBS			
Q5T8I9	Small RNA 2'-O-	HENMT1			
Q15034	Probable E3 ubiquitin-protein ligase HERC3	HERC3			
P01854	Immunoglobulin heavy constant epsilon	IGHE			
P27930	Interleukin-1 receptor type 2	IL1R2			
Q9UHF5	Interleukin-17B	IL17B			
P01584	Interleukin-1 beta	IL1B			
P01876	Immunoglobulin heavy constant alpha 1	IGHA1			
P29460	Interleukin-12 subunit beta	IL12B			
Q6UWQ7	Insulin growth factor-like family member 2	IGFL2			
P01860	Immunoglobulin heavy constant gamma 3	IGHG3			
Q14626	Interleukin-11 receptor subunit alpha	IL11RA			
B1ANH7	Putative uncharacterized protein IBA57-DT	IBA57-DT			
P18564	Integrin beta-6	ITGB6			
Q9HCI6	E3 SUMO-protein ligase	KIAA1586			
Q04695	Keratin, type I cytoskeletal 17	KRT17			
P05412	Transcription factor AP-1	JUN			
P78385	Keratin, type II cuticular Hb3	KRT83			
O75164	Lysine-specific demethylase 4A	KDM4A			
Q9H008	Phospholysine phosphohistidine inorganic pyrophosphate phosphatase	LHPP			
Q6P5S2	Protein LEG1 homolog	LEG1			
Q5T7P2	Late cornified envelope protein 1A	LCE1A			
Q5TA79	Late cornified envelope protein 2A	LCE2A			
Q8IWV1	Lymphocyte transmembrane adapter 1	LAX1			

Q5T700	Low-density lipoprotein receptor class A domain-containing protein 1	LDLRAD1			
A6NHZ5	Leucine-rich repeat-containing protein 14B	LRRC14B			
Q8N456	Leucine-rich repeat-containing protein 18	LRRC18			
Q8IWT6	Volume-regulated anion channel subunit LRRC8A	LRRC8A			
Q9Y2L9	Leucine-rich repeat and calponin homology domain-containing protein 1	LRCH1			
P48449	Lanosterol synthase	LSS			
Q8IV03	Leucine rich adaptor protein 1-	LURAP1L			
O75019	Leukocyte immunoglobulin-like receptor subfamily A member 1	LILRA1			
Q717R9	Cystin-1	CYS1			
Q7L014	Probable ATP-dependent RNA helicase DDX46	DDX46			
Q9NUI1	Peroxisomal 2,4-dienoyl-CoA reductase	DECR2			
Q30KR1	Putative beta-defensin 109B	DEFB109B			
Q5HYR2	Doublesex- and mab-3-related transcription factor C1	DMRTC1;			
Q9Y2H0	Disks large-associated protein 4	DLGAP4			
Q86SQ9	Dehydrodolichyl diphosphate synthase complex subunit DHDDS	DHDDS			
Q96HU8	GTP-binding protein Di-Ras2	DIRAS2			
Q9UBP4	Dickkopf-related protein 3	DKK3			
Q9UBT3	Dickkopf-related protein 4	DKK4			
Q9BTV6	Diphthine methyltransferase	DPH7			
O94907	Dickkopf-related protein 1	DKK1			
Q4G0W2	Dual specificity phosphatase 28	DUSP28			
P20800	Endothelin-2	EDN2			
P0CJ90	Double homeobox protein 4-like protein 7	DUX4L7			
O75319	RNA/RNP complex-1-interacting phosphatase	DUSP11			
A8MWY0	Endosome/lysosome-associated apoptosis and autophagy regulator family member 2	ELAPOR2			
Q6UXG2	Endosome/lysosome-associated apoptosis and autophagy regulator 1	ELAPOR1			
Q99814	Endothelial PAS domain-containing protein 1	EPAS1			
Q9H2F5	Enhancer of polycomb homolog 1	EPC1			
Q9HB65	RNA polymerase II elongation factor ELL3	ELL3			
O00303	Eukaryotic translation initiation factor 3 subunit F	EIF3F			
Q17RN3	Protein FAM98C	FAM98C			
Q56NI9	N-acetyltransferase ESCO2	ESCO2			
P07992	DNA excision repair protein ERCC-1	ERCC1			
Q9BS26	Endoplasmic reticulum resident protein 44	ERP44			
Q86YD7	Protein FAM90A1	FAM90A1			
Q92520	Protein FAM3C	FAM3C			
Q96CN4	EVI5-like protein	EVI5L			
Q9Y2M0	Fanconi-associated nuclease 1	FAN1			
Q5T9C2	Protein FAM102A	FAM102A			
Q96KR6	Protein FAM210B, mitochondrial	FAM210B			
Q7L5A3	Protein FAM214B	FAM214B			
Q4G0N7	Protein FAM229B	FAM229B			
O60825	6-phosphofructo-2-kinase/fructose-2,6-bisphosphatase 2	PFKFB2			
Q99645	Epiphycan	EPYC			

Q6P179	Endoplasmic reticulum aminopeptidase 2	ERAP2			
Q8N8J7	Uncharacterized protein FAM241A	FAM241A			
Q0JRZ9	F-BAR domain only protein 2	FCHO2			
Q9UGM5	Fetuin-B	FETUB			
Q9UBX5	Fibulin-5	FBLN5			
P49789	Bis	FHIT			
P04066	Tissue alpha-L-fucosidase	FUCA1			
Q5T0N5	Formin-binding protein 1-like	FNBP1L			
P62685	Endogenous retrovirus group K member 8 Gag polyprotein	ERVK-8			
Q9H3C7	Gametogenetin-binding protein 2	GGNBP2			
I3L273	Golgi-associated olfactory signaling regulator	GFY			
Q14749	Glycine N-methyltransferase	GNMT			
P01148	Progonadoliberin-1	GNRH1			
P47870	Gamma-aminobutyric acid receptor subunit beta-2	GABRB2			
P60983	Glia maturation factor beta	GMFB			
Q5SZD4	Glycine N-acyltransferase-like protein 3	GLYATL3			
Q2TAP0	Golgin subfamily A member 7B	GOLGA7B			
Q99680	G-protein coupled receptor 22	GPR22			
Q9NRJ2	Putative uncharacterized protein GSN-AS1	GSN-AS1			
O43824	Putative GTP-binding protein 6	GTPBP6			
Q13442	28 kDa heat- and acid-stable phosphoprotein	PDAP1			
Q58FF8	Putative heat shock protein HSP 90-beta 2	HSP90AB2P			
P49639	Homeobox protein Hox-A1	HOXA1			
P01817	Immunoglobulin heavy variable 2-5	IGHV2-5			
P0DP04	Immunoglobulin heavy variable 3-43D	IGHV3-43D			
P0DP06	Immunoglobulin heavy variable 4-30-4	IGHV4-30-4			
A0A0B4J1X5	Immunoglobulin heavy variable 3-74	IGHV3-74			
Q8WVV9	Heterogeneous nuclear ribonucleoprotein L-like	HNRNPLL			
Q9NZL4	Hsp70-binding protein 1	HSPBP1			
Q8IWL3	Iron-sulfur cluster co-chaperone protein HscB	HSCB			
P0CJ73	Humanin-like 6	MTRNR2L6			
O97980	Minor histocompatibility protein HB-1	HMHB1			
Q71H61	Immunoglobulin-like domain-containing receptor 2	ILDR2			
Q6B9Z1	Insulin growth factor-like family member 4	IGFL4			
P38919	Eukaryotic initiation factor 4A-III	EIF4A3			
Q9NX55	Huntingtin-interacting protein K	HYPK			
O00505	Importin subunit alpha-4	KPNA3			
Q5DT21	Serine protease inhibitor Kazal-type 9	SPINK9			
P15884	Transcription factor 4	TCF4			
A6NMK8	Protein INSYN2B	INSYN2B			
Q9P2G3	Kelch-like protein 14	KLHL14			
P55103	Inhibin beta C chain	INHBC			
Q96J84	Kin of IRRE-like protein 1	KIRREL1			
P78415	Iroquois-class homeodomain protein IRX-3	IRX3			
Q8NB78	Lysine-specific histone demethylase 1B	KDM1B			
Q8NEP7	Kelch domain-containing protein	KLHDC9			
P78508	ATP-sensitive inward rectifier potassium channel 10	KCNJ10			

Q07666	KH domain-containing, RNA-binding, signal transduction-associated protein 1	KHDRBS1			
Q9BYQ3	Keratin-associated protein 9-3	KRTAP9-3			
Q9NS86	LanC-like protein 2	LANCL2			
Q9BZL6	Serine/threonine-protein kinase D2	PRKD2			
Q05315	Galectin-10	CLC			
Q8IYG6	Leucine-rich repeat-containing protein 56	LRRC56			
Q8IUZ0	Leucine-rich repeat-containing protein 49	LRRC49			
Q86VH4	Leucine-rich repeat transmembrane neuronal protein 4	LRRTM4			
O14520	Aquaporin-7	AQP7			
Q92482	Aquaporin-3	AQP3			
Q86WG3	Caytaxin	ATCAY			
Q9P1U1	Actin-related protein 3B	ACTR3B			
Q9H765	Ankyrin repeat and SOCS box protein 8	ASB8			
Q9NX76	CKLF-like MARVEL transmembrane domain-containing protein 6	CMTM6			
Q14028	Cyclic nucleotide-gated cation channel beta-1	CNGB1			
Q5SYC1	Clavesin-2	CLVS2			
O43734	E3 ubiquitin ligase TRAF3IP2	TRAF3IP2			
Q7Z7G1	Cytokine-dependent hematopoietic cell linker	CLNK			
Q92905	COP9 signalosome complex subunit 5	COPS5			
Q14993	Collagen alpha-1	COL19A1			
Q96A23	Copine-4	CPNE4			
P04118	Colipase	CLPS			
Q96N68	Putative uncharacterized protein C18orf15	C18orf15			
Q8N1L4	Putative inactive cytochrome P450 family member 4Z2	CYP4Z2P			
Q8WTU0	Protein DDI1 homolog 1	DDI1			
O95822	Malonyl-CoA decarboxylase, mitochondrial	MLYCD			
O43143	Pre-mRNA-splicing factor ATP-dependent RNA helicase DHX15	DHX15			
P15924	Desmoplakin	DSP			
Q8IYX4	Dead end protein homolog 1	DND1			
Q9H3Z4	DnaJ homolog subfamily C member 5	DNAJC5			
P49917	DNA ligase 4	LIG4			
Q9UPY3	Endoribonuclease Dicer	DICER1			
Q8NCG7	Diacylglycerol lipase-beta	DAGLB			
Q9Y5T4	DnaJ homolog subfamily C member 15	DNAJC15			
Q9NVM6	DnaJ homolog subfamily C member 17	DNAJC17			
Q9UK85	Dickkopf-like protein 1	DKKL1			
Q99956	Dual specificity protein phosphatase 9	DUSP9			
Q6UX65	DNA damage-regulated autophagy modulator protein 2	DRAM2			
Q96G04	Protein-lysine N-methyltransferase EEF2KMT	EEF2KMT			
Q12967	Ral guanine nucleotide dissociation stimulator	RALGDS			
P42892	Endothelin-converting enzyme 1	ECE1			
Q03001	Dystonin	DST			
Q5VTE0	Putative elongation factor 1-alpha-like 3	EEF1A1P5			

Q99527	G-protein coupled estrogen receptor 1	GPER1			
Q9Y3R5	Protein dopey-2	DOP1B			
P56851	Epididymal secretory protein E3-beta	EDDM3B			
Q9BXX0	EMILIN-2	EMILIN2			
Q8N336	ELMO domain-containing protein 1	ELMOD1			
Q9UM22	Mammalian ependymin-related protein 1	EPDR1			
Q96C92	Endosome-associated-trafficking regulator 1	ENTR1			
Q8NCI6	Beta-galactosidase-1-like protein 3	GLB1L3			
Q8TB36	Ganglioside-induced differentiation-associated protein 1	GDAP1			
Q14549	Homeobox protein GBX-1	GBX1			
Q8WZA8	Putative gastric cancer-related gene 224 protein	GCRG224			
O95528	Solute carrier family 2, facilitated glucose transporter member 10	SLC2A10			
Q8IXQ5	Kelch-like protein 7	KLHL7			
Q9Y450	HBS1-like protein	HBS1L			
Q6FI13	Histone H2A type 2-A	H2AC18			
Q3ZCU0	Protein GVQW3	GVQW3			
Q92993	Histone acetyltransferase KAT5	KAT5			
Q8WTQ7	Rhodopsin kinase GRK7	GRK7			
P16401	Histone H1.5	H1-5			
A8MVS5	Protein HIDE1	HIDE1			
Q96MB7	Putative nuclease HARBI1	HARBI1			
P12081	Histidine--tRNA ligase, cytoplasmic	HARS1			
C9JL84	HERV-H LTR-associating protein 1	HHLA1			
Q5U5R9	Probable E3 ubiquitin-protein ligase HECTD2	HECTD2			
O43365	Homeobox protein Hox-A3	HOXA3			
Q00056	Homeobox protein Hox-A4	HOXA4			
A6NFD8	Hairy and enhancer of split-related protein HELT	HELT			
A0A0J9YXX1	Immunoglobulin heavy variable 5-10-1	IGHV5-10-1			
P01742	Immunoglobulin heavy variable 1-69	IGHV1-69			
A0A0B4J1X8	Immunoglobulin heavy variable 3-43	IGHV3-43			
P0DMV9	Heat shock 70 kDa protein 1B	HSPA1B			
P54868	Hydroxymethylglutaryl-CoA synthase, mitochondrial	HMGCS2			
Q86SU0	Immunoglobulin-like domain-containing receptor 1	ILDR1			
P05114	Non-histone chromosomal protein HMG-14	HMGN1			
P13747	HLA class I histocompatibility antigen, alpha chain E	HLA-E			
P55010	Eukaryotic translation initiation factor 5	EIF5			
Q9NZI8	Insulin-like growth factor 2 mRNA-binding protein 1	IGF2BP1			
Q9P0M4	Interleukin-17C	IL17C			
O00629	Importin subunit alpha-3	KPNA4			
Q6NXR0	Interferon-inducible GTPase 5	IRGC			
P60568	Interleukin-2	IL2			
P0DOY3	Immunoglobulin lambda constant 3	IGLC3			
P05155	Plasma protease C1 inhibitor	SERPING1			
Q9NRM6	Interleukin-17 receptor B	IL17RB			
Q6ZMJ4	Interleukin-34	IL34			

P24394	Interleukin-4 receptor subunit alpha	IL4R			
Q8WUF5	RelA-associated inhibitor	PPP1R13L			
Q96CN7	Isochorismatase domain-containing protein 1	ISOC1			
Q96MA6	Adenylate kinase 8	AK8			
O15397	Importin-8	IPO8			
P08779	Keratin, type I cytoskeletal 16	KRT16			
Q9H160	Inhibitor of growth protein 2	ING2			
P26440	Isovaleryl-CoA dehydrogenase, mitochondrial	IVD			
Q9UM21	Alpha-1,3-mannosyl-glycoprotein 4-beta-N-acetylglucosaminyltransferase A	MGAT4A			
Q96M94	Kelch-like protein 15	KLHL15			
D3W0D1	Killer cell lectin-like receptor subfamily F member 2	KLRF2			
Q53RY4	Keratinocyte-associated protein 3	KRTCAP3			
Q6PI47	BTB/POZ domain-containing protein KCTD18	KCTD18			
O76011	Keratin, type I cuticular Ha4	KRT34			
Q7Z5Y7	BTB/POZ domain-containing protein KCTD20	KCTD20			
Q14721	Potassium voltage-gated channel subfamily B member 1	KCNB1			
Q63HM1	Kynurenine formamidase	AFMID			
Q8TBQ9	Protein kish-A	TMEM167A			
Q9NZV8	Potassium voltage-gated channel subfamily D member 2	KCND2			
Q9NXV2	BTB/POZ domain-containing protein KCTD5	KCTD5			
Q53GT1	Kelch-like protein 22	KLHL22			
P41743	Protein kinase C iota type	PRKCI			
Q8NCW0	Kremen protein 2	KREMEN2			
A0A0C4DH67	Immunoglobulin kappa variable 1-8	IGKV1-8			
P13473	Lysosome-associated membrane glycoprotein 2	LAMP2			
Q9NUJ1	Palmitoyl-protein thioesterase ABHD10, mitochondrial	ABHD10			
P08173	Muscarinic acetylcholine receptor M4	CHRM4			
P46952	3-hydroxyanthranilate 3,4-dioxygenase	HAAO			
P42684	Tyrosine-protein kinase ABL2	ABL2			
Q9NPC4	Lactosylceramide 4-alpha-galactosyltransferase	A4GALT			
Q9UJX6	Anaphase-promoting complex subunit 2	ANAPC2			
Q9UPM8	AP-4 complex subunit epsilon-1	AP4E1			
Q8N4T4	Rho guanine nucleotide exchange factor 39	ARHGEF39			
Q0P5N6	ADP-ribosylation factor-like protein 16	ARL16			
Q8N6S5	ADP-ribosylation factor-like protein 6-interacting protein 6	ARL6IP6			
Q9Y2G4	Ankyrin repeat domain-containing protein 6	ANKRD6			
P10398	Serine/threonine-protein kinase A-Raf	ARAF			
Q5SQI0	Alpha-tubulin N-acetyltransferase 1	ATAT1			
Q8TDY4	Arf-GAP with SH3 domain, ANK repeat and PH domain-containing protein 3	ASAP3			
Q6ZVZ8	Ankyrin repeat and SOCS box protein 18	ASB18			
Q3MJ40	Coiled-coil domain-containing protein 144B	CCDC144B			

Q9NUP1	Biogenesis of lysosome-related organelles complex 1 subunit 4	BLOC1S4			
A6QL63	Ankyrin repeat and BTB/POZ domain-containing protein BTBD11	BTBD11			
Q5TBC7	Bcl-2-like protein 15	BCL2L15			
P55061	Bax inhibitor 1	TMBIM6			
Q969J3	BLOC-1-related complex subunit 5	BORCS5			
Q9Y6D5	Brefeldin A-inhibited guanine nucleotide-exchange protein 2	ARFGEF2			
Q3B7T3	Protein BEAN1	BEAN1			
Q9NPZ5	Galactosylgalactosylxylosylprotein 3-beta-glucuronosyltransferase 2	B3GAT2			
O95429	BAG family molecular chaperone regulator 4	BAG4			
Q16520	Basic leucine zipper transcriptional factor ATF-like	BATF			
P06881	Calcitonin gene-related peptide 1	CALCA			
Q9P2K1	Coiled-coil and C2 domain-containing protein 2A	CC2D2A			
Q9ULV8	E3 ubiquitin-protein ligase CBL-C	CBLC			
Q9UK00	Uncharacterized protein C3orf18	C3orf18			
P13994	Coiled-coil domain-containing protein 130	CCDC130			
Q8NCU1	Uncharacterized protein CCDC197	CCDC197			
Q7Z3E2	Coiled-coil domain-containing protein 186	CCDC186			
Q8NDL9	Cytosolic carboxypeptidase-like protein 5	AGBL5			
P00918	Carbonic anhydrase 2	CA2			
P13501	C-C motif chemokine 5	CCL5			
Q5SZD1	Uncharacterized protein C6orf141	C6orf141			
Q8IWX8	Calcium homeostasis endoplasmic reticulum protein	CHERP			
Q03701	CCAAT/enhancer-binding protein zeta	CEBPZ			
P16070	CD44 antigen	CD44			
P61604	10 kDa heat shock protein, mitochondrial	HSPE1			
Q8N9P6	Uncharacterized protein C9orf163	C9orf163			
Q9P2D1	Chromodomain-helicase-DNA-binding protein 7	CHD7			
Q8TDX6	Chondroitin sulfate N-acetylgalactosaminyltransferase 1	CSGALNACT1			
Q96MD7	Uncharacterized protein C9orf85	C9orf85			
Q5T5M9	Cyclin-J	CCNJ			
Q5T5N4	Uncharacterized protein C6orf118	C6orf118			
Q8IU89	Ceramide synthase 3	CERS3			
C9JXX5	Uncharacterized protein C11orf94	C11orf94			
B7Z368	Uncharacterized protein encoded by LINC02881	LINC02881			
Q03692	Collagen alpha-1	COL10A1			
Q6PJW8	Consortin	CNST			
Q9UQC9	Calcium-activated chloride channel regulator 2	CLCA2			
O14967	Calmegin	CLGN			
P54107	Cysteine-rich secretory protein 1	CRISP1			
Q9BZB8	Cytoplasmic polyadenylation element-binding protein 1	CPEB1			
P13584	Cytochrome P450 4B1	CYP4B1			
O14810	Complexin-1	CPLX1			
Q9H6X5	Uncharacterized protein C19orf44	C19orf44			
C9J6K1	Putative uncharacterized protein C19orf81	C19orf81			
Q6ZPD9	Probable C-mannosyltransferase DPY19L3	DPY19L3			
P50461	Cysteine and glycine-rich protein	CSRP3			
Q8N104	Beta-defensin 106	DEFB106A			

A0A1B0GTR3	Uncharacterized protein CXorf51A	CXorf51A			
P0DP73	Beta-defensin 130B	DEFB130B			
Q9H467	CUE domain-containing protein 2	CUEDC2			
Q8WYQ4	Uncharacterized protein C22orf15	C22orf15			
Q9H0Q0	CYFIP-related Rac1 interactor A	CYRIA			
Q96SD1	Protein artemis	DCLRE1C			
A2RUS2	DENN domain-containing protein 3	DENND3			
Q9Y6V7	Probable ATP-dependent RNA helicase DDX49	DDX49			
Q8IZD4	mRNA-decapping enzyme 1B	DCP1B			
P80365	Corticosteroid 11-beta-dehydrogenase isozyme 2	HSD11B2			
P37059	17-beta-hydroxysteroid dehydrogenase type 2	HSD17B2			
P51659	Peroxisomal multifunctional enzyme type 2	HSD17B4			
Q9H5J4	Elongation of very long chain fatty acids protein 6	ELOVL6			
Q5J8M3	ER membrane protein complex subunit 4	EMC4			
P06733	Alpha-enolase	ENO1			
Q9UHY7	Enolase-phosphatase E1	ENOPH1			
Q96J88	Epithelial-stromal interaction protein 1	EPSTI1			
Q5XG92	Carboxylesterase 4A	CES4A			
Q96K12	Fatty acyl-CoA reductase 2	FAR2			
O15083	ERC protein 2	ERC2			
P62495	Eukaryotic peptide chain release factor subunit 1	ETF1			
Q05397	Focal adhesion kinase 1	PTK2			
P50548	ETS domain-containing transcription factor ERF	ERF			
Q6P587	Acylpyruvase FAHD1, mitochondrial	FAHD1			
Q86UY5	Protein FAM83A	FAM83A			
P22794	Protein EVI2A	EVI2A			
Q3ZCQ3	Membrane protein FAM174B	FAM174B			
Q8N128	Protein FAM177A1	FAM177A1			
O95571	Persulfide dioxygenase ETHE1, mitochondrial	ETHE1			
Q49AJ0	Protein FAM135B	FAM135B			
C9JC47	Putative protein FAM157A	FAM157A			
Q8N7L0	Protein FAM216B	FAM216B			
A0A1B0GVR7	Protein FAM240C	FAM240C			
O60774	Putative dimethylaniline monooxygenase	FMO6P			
Q96DP5	Methionyl-tRNA formyltransferase, mitochondrial	MTFMT			
Q6BAA4	Fc receptor-like B	FCRLB			
Q96AY3	Peptidyl-prolyl cis-trans isomerase FKBP10	FKBP10			
Q5T1M5	FK506-binding protein 15	FKBP15			
Q8N0W3	L-fucose kinase	FCSK			
Q7Z6M2	F-box only protein 33	FBXO33			
Q96C11	FGGY carbohydrate kinase domain-containing protein	FGGY			
Q96QU4	Protein FRG2-like-1	FRG2B			
Q5NUL3	Free fatty acid receptor 4	FFAR4			
Q9UQC2	GRB2-associated-binding protein 2	GAB2			
Q9BTY2	Plasma alpha-L-fucosidase	FUCA2			
Q9Y2L6	FERM domain-containing protein 4B	FRMD4B			
Q8N292	Protein GAPT	GAPT			
Q12841	Follistatin-related protein 1	FSTL1			

Q0VG06	Fanconi anemia core complex-associated protein 100	FAAP100			
P85037	Forkhead box protein K1	FOXK1			
A8MWK0	Putative fatty acid desaturase 2-like protein FADS2B	FADS2B			
Q9H4G4	Golgi-associated plant pathogenesis-related protein 1	GLIPR2			
Q9H936	Mitochondrial glutamate carrier 1	SLC25A22			
Q8TF65	PDZ domain-containing protein GIPC2	GIPC2			
Q8WU67	Phospholipase ABHD3	ABHD3			
P11230	Acetylcholine receptor subunit beta	CHRNB1			
Q6ZSZ5	Rho guanine nucleotide exchange factor 18	ARHGEF18			
O14497	AT-rich interactive domain-containing protein 1A	ARID1A			
P28838	Cytosol aminopeptidase	LAP3			
Q8NB49	Phospholipid-transporting ATPase IG	ATP11C			
Q9Y2G3	Probable phospholipid-transporting ATPase IF	ATP11B			
P11245	Arylamine N-acetyltransferase 2	NAT2			
Q9H1Y0	Autophagy protein 5	ATG5			
Q8WUW1	Protein BRICK1	BRK1			
O95393	Bone morphogenetic protein 10	BMP10			
Q9HCU9	Breast cancer metastasis-suppressor 1	BRMS1			
Q9BRD0	BUD13 homolog	BUD13			
Q9H3Q1	Cdc42 effector protein 4	CDC42EP4			
Q9UKI2	Cdc42 effector protein 3	CDC42EP3			
O60909	Beta-1,4-galactosyltransferase 2	B4GALT2			
P62952	Bladder cancer-associated protein	BLCAP			
Q7Z5W3	RNA 5'-monophosphate methyltransferase	BCDIN3D			
Q9BZR8	Apoptosis facilitator Bcl-2-like protein 14	BCL2L14			
Q96GS4	BLOC-1-related complex subunit 6	BORCS6			
Q9BYG0	Lactosylceramide 1,3-N-acetyl-beta-D-glucosaminyltransferase	B3GNT5			
Q9H8M2	Bromodomain-containing protein 9	BRD9			
Q13072	B melanoma antigen 1	BAGE			
Q8NHY0	Beta-1,4 N-acetylgalactosaminyltransferase 2	B4GALNT2			
Q7Z5L3	Complement C1q-like protein 2	C1QL2			
P05814	Beta-casein	CSN2			
Q8WV48	Coiled-coil domain-containing protein 107	CCDC107			
Q6ZN84	Coiled-coil domain-containing protein 81	CCDC81			
Q8N350	Voltage-dependent calcium channel beta subunit-associated regulatory protein	CBARP			
P45973	Chromobox protein homolog 5	CBX5			
P22362	C-C motif chemokine 1	CCL1			
Q6UY09	Carcinoembryonic antigen-related cell adhesion molecule 20	CEACAM20			
Q16663	C-C motif chemokine 15	CCL15			
Q5VT06	Centrosome-associated protein 350	CEP350			
Q9UQN3	Charged multivesicular body protein 2b	CHMP2B			
P0DO97	Coiled-coil domain-containing protein 192	CCDC192			
P52757	Beta-chimaerin	CHN2			
P51685	C-C chemokine receptor type 8	CCR8			
Q9P209	Centrosomal protein of 72 kDa	CEP72			

P11912	B-cell antigen receptor complex-associated protein alpha chain	CD79A			
Q6ZMG9	Ceramide synthase 6	CERS6			
Q9GZU0	Uncharacterized protein C6orf62	C6orf62			
Q99618	Cell division cycle-associated protein 3	CDCA3			
Q00537	Cyclin-dependent kinase 17	CDK17			
Q6P9H4	Connector enhancer of kinase suppressor of ras 3	CNKSR3			
Q14050	Collagen alpha-3	COL9A3			
Q7Z7K6	Centromere protein V	CENPV			
P20849	Collagen alpha-1	COL9A1			
Q5JTJ3	Cytochrome c oxidase assembly factor 6 homolog	COA6			
Q9NZ45	CDGSH iron-sulfur domain-containing protein 1	CISD1			
Q8N769	Uncharacterized protein C14orf178	C14orf178			
Q9NWQ9	Uncharacterized protein C14orf119	C14orf119			
Q8N1G2	Cap-specific mRNA	CMTR1			
Q9H972	Uncharacterized protein C14orf93	C14orf93			
Q8IYJ2	Uncharacterized protein C10orf67, mitochondrial	C10orf67			
Q96AJ1	Clusterin-associated protein 1	CLUAP1			
Q8IYA6	Cytoskeleton-associated protein 2-like	CKAP2L			
P53675	Clathrin heavy chain 2	CLTCL1			
Q9BW66	Cyclin-dependent kinase 2-interacting protein	CINP			
P82279	Protein crumbs homolog 1	CRB1			
Q9UQ03	Coronin-2B	CORO2B			
P31327	Carbamoyl-phosphate synthase	CPS1			
Q96SQ9	Cytochrome P450 2S1	CYP2S1			
P07316	Gamma-crystallin B	CRYGB			
P02511	Alpha-crystallin B chain	CRYAB			
P46109	Crk-like protein	CRKL			
P0CG13	Chromosome transmission fidelity protein 8 homolog	CHTF8			
Q9GZU7	Carboxy-terminal domain RNA polymerase II polypeptide A small phosphatase 1	CTDSP1			
Q96RT6	cTAGE family member 2	CTAGE1			
Q8NFK1	Gap junction gamma-3 protein	GJC3			
Q9H8E8	Cysteine-rich protein 2-binding protein	KAT14			
Q9Y6G9	Cytoplasmic dynein 1 light intermediate chain 1	DYNC1LI1			
Q9BTE7	DCN1-like protein 5	DCUN1D5			
Q8IY21	Probable ATP-dependent RNA helicase DDX60	DDX60			
Q8WXF8	DNA-binding death effector domain-containing protein 2	DEDD2			
Q9NPI6	mRNA-decapping enzyme 1A	DCP1A			
Q8N690	Beta-defensin 119	DEFB119			
Q92771	Putative ATP-dependent RNA helicase DDX12	DDX12P			
Q9UI46	Dynein intermediate chain 1, axonemal	DNAI1			
P06340	HLA class II histocompatibility antigen, DO alpha chain	HLA-DOA			
Q9NSV4	Protein diaphanous homolog 3	DIAPH3			
Q05329	Glutamate decarboxylase 2	GAD2			
Q5T7M9	Divergent protein kinase domain 1A	DIPK1A			
Q8N1I0	Dedicator of cytokinesis protein 4	DOCK4			
O95886	Disks large-associated protein 3	DLGAP3			
P59022	Down syndrome critical region protein 10	DSCR10			

Q0VDD8	Dynein heavy chain 14, axonemal	DNAH14			
Q15054	DNA polymerase delta subunit 3	POLD3			
Q8N8Q3	Endonuclease V	ENDOV			
Q15370	Elongin-B	ELOB			
Q8N140	EP300-interacting inhibitor of differentiation 3	EID3			
Q96AZ1	EEF1A lysine methyltransferase 3	EEF1AKMT			
Q8WVE0	EEF1A lysine methyltransferase 1	EEF1AKMT			
P11308	Transcriptional regulator ERG	ERG			
O60667	Fas apoptotic inhibitory molecule 3	FCMR			
P84090	Enhancer of rudimentary homolog	ERH			
P34910	Protein EVI2B	EVI2B			
Q6P995	Protein FAM171B	FAM171B			
Q5XKR9	Protein FAM104B	FAM104B			
Q9BPY3	Protein FAM118B	FAM118B			
Q9H098	Protein FAM107B	FAM107B			
Q7L775	EPM2A-interacting protein 1	EPM2AIP1			
Q8IYM0	Protein FAM186B	FAM186B			
Q32MH5	Protein FAM214A	FAM214A			
A0A1B0GW35	Exocyst complex component 1-like	EXOC1L			
P14921	Protein C-ets-1	ETS1			
B1AL88	Transmembrane protein	FAM155A			
Q96ND0	Protein FAM210A	FAM210A			
Q9Y5M1	Uncharacterized protein FAM215A	FAM215A			
Q9UHL3	Protein FAM153A	FAM153A			
Q12929	Epidermal growth factor receptor kinase substrate 8	EPS8			
Q9Y6X4	Soluble lamin-associated protein of 75 kDa	FAM169A			
A0A1B0GVD1	Protein FAM237B	FAM237B			
O00167	Eyes absent homolog 2	EYA2			
Q9NYL4	Peptidyl-prolyl cis-trans isomerase FKBP11	FKBP11			
Q969P5	F-box only protein 32	FBXO32			
Q8NCQ5	F-box only protein 15	FBXO15			
Q9UK97	F-box only protein 9	FBXO9			
Q8NA97	Putative uncharacterized protein FER1L6-AS1	FER1L6-AS1			
Q969F0	Fetal and adult testis-expressed transcript protein	FATE1			
Q6QHK4	Factor in the germline alpha	FIGLA			
P36980	Complement factor H-related protein 2	CFHR2			
Q9NRK6	ATP-binding cassette sub-family B member 10, mitochondrial	ABCB10			
P08910	Monoacylglycerol lipase ABHD2	ABHD2			
P49902	Cytosolic purine 5'-nucleotidase	NT5C2			
P34969	5-hydroxytryptamine receptor 7	HTR7			
Q9NYB9	Abl interactor 2	ABI2			
P55263	Adenosine kinase	ADK			
Q60I27	ALS2 C-terminal-like protein	ALS2CL			
Q02040	A-kinase anchor protein 17A	AKAP17A			
P55789	FAD-linked sulfhydryl oxidase ALR	GFER			
Q9H6R3	Acyl-CoA synthetase short-chain family member 3, mitochondrial	ACSS3			
Q8WV93	AFG1-like ATPase	AFG1L			
A1A5B4	Anoctamin-9	ANO9			
Q9BS18	Anaphase-promoting complex subunit 13	ANAPC13			
Q96LA8	Protein arginine N-methyltransferase 6	PRMT6			
Q6UX39	Amelotin	AMTN			
A6NJG6	Arginine-fifty homeobox	ARGFX			
Q9UKU9	Angiopoietin-related protein 2	ANGPTL2			
Q9NZN5	Rho guanine nucleotide exchange factor 12	ARHGEF12			
O60306	RNA helicase aquarius	AQR			

Q15052	Rho guanine nucleotide exchange factor 6	ARHGEF6			
Q8IWZ3	Ankyrin repeat and KH domain-containing protein 1	ANKHD1			
Q5U4P2	Aspartate beta-hydroxylase domain-containing protein 1	ASPHD1			
Q12797	Aspartyl/asparaginyl beta-hydroxylase	ASPH			
P61158	Actin-related protein 3	ACTR3			
Q9BSF8	BTB/POZ domain-containing protein 10	BTBD10			
P55201	Peregrin	BRPF1			
Q68D86	Coiled-coil domain-containing protein 102B	CCDC102B			
Q7Z465	Bcl-2/adenovirus E1B 19 kDa-interacting protein 2-like protein	BNIPL			
Q96G97	Seipin	BSCL2			
Q96Q91	Anion exchange protein 4	SLC4A9			
A0A1B0GUI7	Putative uncharacterized protein BRD3OS	BRD3OS			
A1A5D9	BICD family-like cargo adapter 2	BICDL2			
Q8NA47	Coiled-coil domain-containing protein 63	CCDC63			
Q14790	Caspase-8	CASP8			
Q96AQ1	Coiled-coil domain-containing protein 74A	CCDC74A			
Q8IYE1	Coiled-coil domain-containing protein 13	CCDC13			
Q5I0X4	Uncharacterized protein C6orf226	C6orf226			
Q8WXQ8	Carboxypeptidase A5	CPA5			
Q5SW79	Centrosomal protein of 170 kDa	CEP170			
Q01850	Cerebellar degeneration-related protein 2	CDR2			
P04234	T-cell surface glycoprotein CD3 delta chain	CD3D			
P11049	Leukocyte antigen CD37	CD37			
Q5BN46	UPF0691 protein C9orf116	C9orf116			
Q6NVV7	Cysteine-rich DPF motif domain-containing protein 1	CDPF1			
P86790	Vacuolar fusion protein CCZ1 homolog B	CCZ1B			
O00311	Cell division cycle 7-related protein kinase	CDC7			
Q6UXA7	Uncharacterized protein C6orf15	C6orf15			
P34810	Macrosialin	CD68			
P21127	Cyclin-dependent kinase 11B	CDK11B			
Q2KHT3	Protein CLEC16A	CLEC16A			
O95389	Cellular communication network factor 6	CCN6			
Q8N5B7	Ceramide synthase 5	CERS5			
G3V211	Uncharacterized protein encoded by LINC01619	LINC01619			
H3BRN8	Uncharacterized protein C15orf65	C15orf65			
O75596	C-type lectin domain family 3 member A	CLEC3A			
P04632	Calpain small subunit 1	CAPNS1			
A0A1W2PPM1	Cytoplasmic polyadenylated homeobox-like	CPHXL			
O43405	Cochlin	COCH			
Q9BZP3	Putative uncharacterized protein encoded by LINC00470	LINC00470			
Q9HCS2	Cytochrome P450 4F12	CYP4F12			
Q6QEF8	Coronin-6	CORO6			
Q9UJA2	Cardiolipin synthase	CRLS1			
P24310	Cytochrome c oxidase subunit 7A1, mitochondrial	COX7A1			
P60606	Cortexin-1	CTXN1			
Q8NI51	Transcriptional repressor CTCFL	CTCFL			
Q9HB71	Calcyclin-binding protein	CACYBP			

O75712	Gap junction beta-3 protein	GJB3			
Q8NBI2	Cytochrome b ascorbate-dependent protein 3	CYB561A3			
Q5W188	Putative cystatin-9-like protein CST9LP1	CST9LP1			
O95567	Uncharacterized protein C22orf31	C22orf31			
Q92466	DNA damage-binding protein 2	DDB2			
O15075	Serine/threonine-protein kinase DCLK1	DCLK1			
Q66K64	DDB1- and CUL4-associated factor 15	DCAF15			
Q7Z7B8	Beta-defensin 128	DEFB128			
O15263	Beta-defensin 4A	DEFB4A			
Q08211	ATP-dependent RNA helicase A	DHX9			
P31689	DnaJ homolog subfamily A member 1	DNAJA1			
Q68CQ4	Digestive organ expansion factor homolog	DIEXF			
Q13609	Deoxyribonuclease gamma	DNASE1L3			
Q9NR61	Delta-like protein 4	DLL4			
Q14689	Disco-interacting protein 2 homolog A	DIP2A			
Q96SL1	Solute carrier family 49 member 4	SLC49A4			
Q9Y2E6	E3 ubiquitin-protein ligase DTX4	DTX4			
Q8WWZ3	Ectodysplasin-A receptor-associated adapter protein	EDARADD			
Q96G46	tRNA-dihydrouridine	DUS3L			
Q13347	Eukaryotic translation initiation factor 3 subunit I	EIF3I			
O42043	Endogenous retrovirus group K member 18 Env polyprotein	ERVK-18			
Q06889	Early growth response protein 3	EGR3			
Q8TE69	Protein EOLA1	EOLA1			
Q01844	RNA-binding protein EWS	EWSR1			
Q6NXG1	Epithelial splicing regulatory protein 1	ESRP1			
Q86US8	Telomerase-binding protein EST1A	SMG6			
O60427	Acyl-CoA	FADS1			
A6NE21	Putative protein FAM90A18P/FAM90A19P	FAM90A18P			
A6NJQ4	Putative protein FAM90A8P	FAM90A8P			
Q9Y256	CAAX prenyl protease 2	RCE1			
Q7L5A8	Fatty acid 2-hydroxylase	FA2H			
Q14320	Protein FAM50A	FAM50A			
Q05DH4	Protein FAM160A1	FAM160A1			
Q5JRC9	Protein FAM47A	FAM47A			
Q16875	6-phosphofructo-2-kinase/fructose-2,6-bisphosphatase 3	PFKFB3			
Q5T7N8	Protein FAM27D1	FAM27D1			
Q08E93	Protein FAM27E3	FAM27E3			
Q9BQL6	Fermitin family homolog 1	FERMT1			
Q6X9E4	F-box/WD repeat-containing protein 12	FBXW12			
Q9H4M3	F-box only protein 44	FBXO44			
Q5VTH2	Protein Flattop	CFAP126			
Q8N4B4	F-box only protein 39	FBXO39			
Q14296	Fas-activated serine/threonine kinase	FASTK			
P20930	Filaggrin	FLG			
Q969U6	F-box/WD repeat-containing protein 5	FBXW5			
Q9UHY8	Fasciculation and elongation protein zeta-2	FEZ2			
P61328	Fibroblast growth factor 12	FGF12			
Q9H3Q3	Galactose-3-O-sulfotransferase 2	GAL3ST2			
Q99501	GAS2-like protein 1	GAS2L1			
P02794	Ferritin heavy chain	FTH1			
Q8N4E7	Ferritin, mitochondrial	FTMT			

Q8N1E6	F-box/LRR-repeat protein 14	FBXL14			
Q6PCT2	F-box/LRR-repeat protein 19	FBXL19			
Q5VWT5	FYN-binding protein 2	FYB2			
Q8WYK0	Acetyl-coenzyme A thioesterase	ACOT12			
P02763	Alpha-1-acid glycoprotein 1	ORM1			
Q5VUR7	Putative ankyrin repeat domain-containing protein 20A3	ANKRD20A3P			
Q15027	Arf-GAP with coiled-coil, ANK repeat and PH domain-containing protein 1	ACAP1			
Q9BZC7	ATP-binding cassette sub-family A member 2	ABCA2			
Q8WXI4	Acyl-coenzyme A thioesterase 11	ACOT11			
Q9H222	ATP-binding cassette sub-family G member 5	ABCG5			
Q99424	Peroxisomal acyl-coenzyme A oxidase 2	ACOX2			
Q9UM73	ALK tyrosine kinase receptor	ALK			
Q8NB90	ATPase family protein 2 homolog	SPATA5			
Q16186	Proteasomal ubiquitin receptor ADRM1	ADRM1			
Q07075	Glutamyl aminopeptidase	ENPEP			
Q5VV41	Rho guanine nucleotide exchange factor 16	ARHGEF16			
E9PGG2	Anomalous homeobox protein	ANHX			
P05090	Apolipoprotein D	APOD			
A6NKF2	AT-rich interactive domain-containing protein 3C	ARID3C			
Q8N957	Ankyrin repeat and fibronectin type-III domain-containing protein 1	ANKFN1			
Q03989	AT-rich interactive domain-containing protein 5A	ARID5A			
Q8WXI3	Ankyrin repeat and SOCS box protein 10	ASB10			
Q96LR7	Uncharacterized protein C2orf50	C2orf50			
A6H8Y1	Transcription factor TFIIIB component B'' homolog	BDP1			
Q9BXL7	Caspase recruitment domain-containing protein 11	CARD11			
O75155	Cullin-associated NEDD8-dissociated protein 2	CAND2			
Q2NKX9	UPF0561 protein C2orf68	C2orf68			
Q13137	Calcium-binding and coiled-coil domain-containing protein 2	CALCOCO2			
P33151	Cadherin-5	CDH5			
Q9Y4F5	Centrosomal protein of 170 kDa protein B	CEP170B			
Q9H9S4	Calcium-binding protein 39-like	CAB39L			
P00736	Complement C1r subcomponent	C1R			
Q9BXI9	Complement C1q tumor necrosis factor-related protein 6	C1QTNF6			
Q6F5E8	Capping protein, Arp2/3 and myosin-I linker protein 2	CARMIL2			
P22748	Carbonic anhydrase 4	CA4			
P06307	Cholecystokinin	CCK			
H3BU77	Coiled-coil domain-containing protein 179	CCDC179			
Q6P656	Cilia- and flagella-associated protein 161	CFAP161			
Q8NCU4	Coiled-coil domain-containing protein 191	CCDC191			
A8MTT3	Protein CEBPZOS	CEBPZOS			
Q9UL16	Cilia- and flagella-associated protein 45	CFAP45			
Q9BZP6	Acidic mammalian chitinase	CHIA			
P01730	T-cell surface glycoprotein CD4	CD4			
P07766	T-cell surface glycoprotein CD3 epsilon chain	CD3E			

Q96NL8	Protein C8orf37	C8orf37			
O75794	Cell division cycle protein 123 homolog	CDC123			
P07199	Major centromere autoantigen B	CENPB			
O60563	Cyclin-T1	CCNT1			
O75909	Cyclin-K	CCNK			
Q9Y2V2	Calcium-regulated heat-stable protein 1	CARHSP1			
P13942	Collagen alpha-2	COL11A2			
Q8WXQ3	Putative uncharacterized protein encoded by LINC01599	LINC01599			
P02461	Collagen alpha-1	COL3A1			
E9PRG8	Uncharacterized protein C11orf98	C11orf98			
P61024	Cyclin-dependent kinases regulatory subunit 1	CKS1B			
O96005	Cleft lip and palate transmembrane protein 1	CLPTM1			
Q96KH6	Uncharacterized protein C18orf12	C18orf12			
Q92523	Carnitine O-palmitoyltransferase 1, muscle isoform	CPT1B			
A8K830	Colorectal cancer-associated protein 2	COLCA2			
P24468	COUP transcription factor 2	NR2F2			
Q9HC73	Cytokine receptor-like factor 2	CRLF2			
Q5BKX5	UPF0692 protein C19orf54	C19orf54			
Q8TCD1	UPF0729 protein C18orf32	C18orf32			
P22914	Gamma-crystallin S	CRYGS			
Q6UVK1	Chondroitin sulfate proteoglycan	CSPG4			
Q7Z7A3	Cytoplasmic tRNA 2-thiolation protein 1	CTU1			
Q2NKJ3	CST complex subunit CTC1	CTC1			
Q9UQB3	Catenin delta-2	CTNND2			
P02775	Platelet basic protein	PPBP			
P81534	Beta-defensin 103	DEFB103A			
O60676	Cystatin-8	CST8			
O14578	Citron Rho-interacting kinase	CIT			
Q8N8I6	Putative uncharacterized protein encoded by LINC00482	LINC00482			
O43583	Density-regulated protein	DENR			
Q92499	ATP-dependent RNA helicase DDX1	DDX1			
Q9NR28	Diablo homolog, mitochondrial	DIABLO			
P27707	Deoxycytidine kinase	DCK			
Q8NA75	DDB1- and CUL4-associated factor 4-like protein 2	DCAF4L2			
Q96JK2	DDB1- and CUL4-associated factor 5	DCAF5			
Q14147	Probable ATP-dependent RNA helicase DHX34	DHX34			
Q6IED9	Putative diacylglycerol O-acyltransferase 2-like protein DGAT2L7P	DGAT2L7P			
Q9H5Z1	Probable ATP-dependent RNA helicase DHX35	DHX35			
Q9NQL9	Doublesex- and mab-3-related transcription factor 3	DMRT3			
Q96KC8	DnaJ homolog subfamily C member 1	DNAJC1			
Q5T8R8	Uncharacterized protein DOCK8-AS1	DOCK8-AS1			
Q9NRI5	Disrupted in schizophrenia 1 protein	DISC1			
Q14204	Cytoplasmic dynein 1 heavy chain 1	DYNC1H1			
Q9BQ95	Evolutionarily conserved signaling intermediate in Toll pathway, mitochondrial	ECSIT			
Q68J44	Dual specificity phosphatase 29	DUSP29			

Q96FG2	ELMO domain-containing protein 3	ELMOD3			
Q9NVQ4	Fas apoptotic inhibitory molecule 1	FAIM			
Q96GK7	Fumarylacetoacetate hydrolase domain-containing protein 2A	FAHD2A			
Q969Z0	FAST kinase domain-containing protein 4	TBRG4			
P51161	Gastrotropin	FABP6			
O15540	Fatty acid-binding protein, brain	FABP7			
P0CH98	Protein FAM106C	FAM106C			
Q8TAG9	Exocyst complex component 6	EXOC6			
P07148	Fatty acid-binding protein, liver	FABP1			
Q9BZJ6	Probable G-protein coupled receptor 63	GPR63			
A1A519	Protein FAM170A	FAM170A			
Q8IVS2	Malonyl-CoA-acyl carrier protein transacylase, mitochondrial	MCAT			
Q8IXQ9	Electron transfer flavoprotein beta subunit lysine methyltransferase	ETFBKMT			
Q01780	Exosome component 10	EXOSC10			
O60320	Protein FAM189A1	FAM189A1			
A6NFU0	Ig-like V-type domain-containing protein FAM187A	FAM187A			
Q8IWN6	Protein FAM223A	FAM223A			
P0CG43	Putative protein FAM157C	FAM157C			
B1ANY3	Putative protein FAM220BP	FAM220BP			
A1KXE4	Myelin-associated neurite-outgrowth inhibitor	FAM168B			
A0A1B0GTK4	Protein FAM237A	FAM237A			
Q9C0D9	Ethanolaminephosphotransferase 1	SELENOI			
P12319	High affinity immunoglobulin epsilon receptor subunit alpha	FCER1A			
P22607	Fibroblast growth factor receptor 3	FGFR3			
A6NHQ2	rRNA/tRNA 2'-O-methyltransferase fibrillarin-like protein 1	FBLL1			
P22455	Fibroblast growth factor receptor 4	FGFR4			
Q02985	Complement factor H-related protein 3	CFHR3			
O76093	Fibroblast growth factor 18	FGF18			
Q9NYQ8	Protocadherin Fat 2	FAT2			
Q12805	EGF-containing fibulin-like extracellular matrix protein 1	EFEMP1			
O95876	WD repeat-containing and planar cell polarity effector protein fritz homolog	WDPCP			
Q2WGN9	GRB2-associated-binding protein 4	GAB4			
Q9BZ67	FERM domain-containing protein 8	FRMD8			
Q9YNA8	Endogenous retrovirus group K member 19 Gag polyprotein	ERVK-19			
Q92908	Transcription factor GATA-6	GATA6			
P11310	Medium-chain specific acyl-CoA dehydrogenase, mitochondrial	ACADM			
Q6P4H8	ATP synthase subunit C lysine N-methyltransferase	ATPSCKMT			
P53396	ATP-citrate synthase	ACLY			
Q9NPJ3	Acyl-coenzyme A thioesterase 13	ACOT13			
Q96GX2	Ataxin-7-like protein 3B	ATXN7L3B			
Q9ULC5	Long-chain-fatty-acid--CoA ligase 5	ACSL5			
O95996	Adenomatous polyposis coli protein 2	APC2			

P40616	ADP-ribosylation factor-like protein 1	ARL1			
Q15389	Angiopoietin-1	ANGPT1			
Q8IV38	Ankyrin repeat and MYND domain-containing protein 2	ANKMY2			
P27540	Aryl hydrocarbon receptor nuclear translocator	ARNT			
Q13510	Acid ceramidase	ASAH1			
P54709	Sodium/potassium-transporting ATPase subunit beta-3	ATP1B3			
P42127	Agouti-signaling protein	ASIP			
P07307	Asialoglycoprotein receptor 2	ASGR2			
Q8IWQ3	Serine/threonine-protein kinase BRSK2	BRSK2			
Q12983	BCL2/adenovirus E1B 19 kDa protein-interacting protein 3	BNIP3			
O60513	Beta-1,4-galactosyltransferase 4	B4GALT4			
Q9NY97	N-acetyllactosaminide beta-1,3-N-acetylglucosaminyltransferase 2	B3GNT2			
Q9HAW0	Transcription factor IIIB 50 kDa subunit	BRF2			
Q8WZ19	BTB/POZ domain-containing adapter for CUL3-mediated RhoA degradation protein 1	KCTD13			
Q9P281	BAH and coiled-coil domain-containing protein 1	BAHCC1			
Q9Y276	Mitochondrial chaperone BCS1	BCS1L			
O75808	Calpain-15	CAPN15			
Q96LW7	Caspase recruitment domain-containing protein 19	CARD19			
Q9BWT7	Caspase recruitment domain-containing protein 10	CARD10			
P0C6P0	Putative protein BCL8	NBEAP1			
Q9BXJ1	Complement C1q tumor necrosis factor-related protein 1	C1QTNF1			
Q7Z7H3	Ciliogenesis-associated TTC17-interacting protein	CATIP			
Q9UBR2	Cathepsin Z	CTSZ			
Q5VU69	Uncharacterized protein C1orf189	C1orf189			
Q8IVY1	Type III endosome membrane protein TEMP	C1orf210			
Q8NCU7	C2 calcium-dependent domain-containing protein 4A	C2CD4A			
Q53TS8	C2 calcium-dependent domain-containing protein 6	C2CD6			
H3BNL1	Uncharacterized protein C3orf84	C3orf84			
P83916	Chromobox protein homolog 1	CBX1			
Q5VU57	Cytosolic carboxypeptidase 6	AGBL4			
P48052	Carboxypeptidase A2	CPA2			
Q96IY4	Carboxypeptidase B2	CPB2			
Q96MR6	Cilia- and flagella-associated protein 57	CFAP57			
Q96JM3	Chromosome alignment-maintaining phosphoprotein 1	CHAMP1			
Q86Y33	Cell division cycle protein 20 homolog B	CDC20B			
P51679	C-C chemokine receptor type 4	CCR4			
O95319	CUGBP Elav-like family member 2	CELF2			
P20138	Myeloid cell surface antigen CD33	CD33			
Q9BRJ6	Uncharacterized protein C7orf50	C7orf50			
Q8NAV2	Uncharacterized protein C8orf58	C8orf58			
P19835	Bile salt-activated lipase	CEL			
Q9Y375	Complex I intermediate-associated protein 30, mitochondrial	NDUFAF1			
P20963	T-cell surface glycoprotein CD3 zeta chain	CD247			
Q8IW35	Centrosomal protein of 97 kDa	CEP97			

Q5M9N0	Coiled-coil domain-containing protein 158	CCDC158			
Q9BWT1	Cell division cycle-associated protein 7	CDCA7			
Q9Y3D0	Cytosolic iron-sulfur assembly component 2B	CIAO2B			
Q5JTZ5	Uncharacterized protein C9orf152	C9orf152			
Q96S94	Cyclin-L2	CCNL2			
Q9NSP4	Centromere protein M	CENPM			
Q9HD87	Putative uncharacterized protein C6orf50	C6orf50			
Q9HBB8	Cadherin-related family member	CDHR5			
O94779	Contactin-5	CNTN5			
P51677	C-C chemokine receptor type 3	CCR3			
P02452	Collagen alpha-1	COL1A1			
P20248	Cyclin-A2	CCNA2			
Q7Z7A1	Centriolin	CNTRL			
Q8NCF0	C-type lectin domain family 18 member C	CLEC18C			
Q9Y6Y1	Calmodulin-binding transcription activator 1	CAMTA1			
Q96S95	Calcium/calmodulin-dependent protein kinase II inhibitor 2	CAMK2N2			
A5YKK6	CCR4-NOT transcription complex subunit 1	CNOT1			
Q96KN2	Beta-Ala-His dipeptidase	CNDP1			
Q9Y271	Cysteinyl leukotriene receptor 1	CYSLTR1			
Q7Z7G2	Complexin-4	CPLX4			
P02741	C-reactive protein	CRP			
P19784	Casein kinase II subunit alpha'	CSNK2A2			
Q9H799	Ciliogenesis and planar polarity effector 1	CPLANE1			
Q49AN0	Cryptochrome-2	CRY2			
P0DMV1	Cancer/testis antigen family 45 member A8	CT45A8			
Q8TF08	Cytochrome c oxidase subunit 7B2, mitochondrial	COX7B2			
Q6PD62	RNA polymerase-associated protein CTR9 homolog	CTR9			
Q8IZN7	Beta-defensin 107	DEFB107A			
Q8N7U9	Putative uncharacterized protein encoded by LINC00469	LINC00469			
P33240	Cleavage stimulation factor subunit 2	CSTF2			
P01040	Cystatin-A	CSTA			
Q9NWM3	CUE domain-containing protein 1	CUEDC1			
Q13616	Cullin-1	CUL1			
Q8TEH3	DENN domain-containing protein 1A	DENND1A			
Q14203	Dynactin subunit 1	DCTN1			
Q5VW00	DDB1- and CUL4-associated factor 12-like protein 2	DCAF12L2			
O43237	Cytoplasmic dynein 1 light intermediate chain 2	DYNC1LI2			
O75618	Death effector domain-containing protein	DEDD			
A0PJE2	Dehydrogenase/reductase SDR family member 12	DHRS12			
Q30KQ8	Beta-defensin 112	DEFB112			
Q7Z7B7	Beta-defensin 132	DEFB132			
P11532	Dystrophin	DMD			
Q9NVP1	ATP-dependent RNA helicase DDX18	DDX18			
O75937	DnaJ homolog subfamily C member 8	DNAJC8			
Q9UF47	DnaJ homolog subfamily C member 5B	DNAJC5B			
Q15700	Disks large homolog 2	DLG2			
O43323	Desert hedgehog protein	DHH			

Q7Z5J1	Hydroxysteroid 11-beta-dehydrogenase 1-like protein	HSD11B1L			
Q8NBQ5	Estradiol 17-beta-dehydrogenase 11	HSD17B11			
Q96JB1	Dynein heavy chain 8, axonemal	DNAH8			
Q15375	Ephrin type-A receptor 7	EPHA7			
O75356	Ectonucleoside triphosphate diphosphohydrolase 5	ENTPD5			
O75821	Eukaryotic translation initiation factor 3 subunit G	EIF3G			
Q969X5	Endoplasmic reticulum-Golgi intermediate compartment protein 1	ERGIC1			
Q01362	High affinity immunoglobulin epsilon receptor subunit beta	MS4A2			
O95466	Formin-like protein 1	FMNL1			
Q8N0W7	Fragile X mental retardation 1 neighbor protein	FMR1NB			
Q96P31	Fc receptor-like protein 3	FCRL3			
Q96LA6	Fc receptor-like protein 1	FCRL1			
Q75LS8	Putative FK506-binding protein 9-like protein	FKBP9P1			
Q14318	Peptidyl-prolyl cis-trans isomerase FKBP8	FKBP8			
P55075	Fibroblast growth factor 8	FGF8			
Q14315	Filamin-C	FLNC			
Q9UKC9	F-box/LRR-repeat protein 2	FBXL2			
P05230	Fibroblast growth factor 1	FGF1			
O43320	Fibroblast growth factor 16	FGF16			
P80404	4-aminobutyrate aminotransferase, mitochondrial	ABAT			
Q8NAA4	Protein Atg16l2	ATG16L2			
Q8IUA7	ATP-binding cassette sub-family A member 9	ABCA9			
Q8WWZ4	ATP-binding cassette sub-family A member 10	ABCA10			
Q8TDX5	2-amino-3-carboxymuconate-6-semialdehyde decarboxylase	ACMSD			
Q9H0P0	Cytosolic 5'-nucleotidase 3A	NT5C3A			
P47895	Aldehyde dehydrogenase family 1 member A3	ALDH1A3			
P37023	Serine/threonine-protein kinase receptor R3	ACVRL1			
P61204	ADP-ribosylation factor 3	ARF3			
Q9UKA4	A-kinase anchor protein 11	AKAP11			
Q9HCK5	Protein argonaute-4	AGO4			
Q7Z6V5	tRNA-specific adenosine deaminase 2	ADAT2			
Q8TED9	Actin filament-associated protein 1-like 1	AFAP1L1			
Q5VUY2	Arylacetamide deacetylase-like 4	AADACL4			
O60266	Adenylate cyclase type 3	ADCY3			
Q9UKQ2	Disintegrin and metalloproteinase domain-containing protein 28	ADAM28			
Q9Y4W6	AFG3-like protein 2	AFG3L2			
P33121	Long-chain-fatty-acid--CoA ligase 1	ACSL1			
A6QL64	Ankyrin repeat domain-containing protein 36A	ANKRD36			
Q9BZ19	Ankyrin repeat domain-containing protein 60	ANKRD60			
Q8N8V4	Ankyrin repeat and SAM domain-containing protein 4B	ANKS4B			
P54802	Alpha-N-acetylglucosaminidase	NAGLU			
Q400G9	Archaemetzincin-1	AMZ1			
Q9BPW4	Apolipoprotein L4	APOL4			
P51693	Amyloid-like protein 1	APLP1			
P10275	Androgen receptor	AR			
Q8NDY3		ADPRHL1			

Q6ZP65	BICD family-like cargo adapter 1	BICDL1			
Q8NDY6	Class E basic helix-loop-helix protein 23	BHLHE23			
A2IDD5	Coiled-coil domain-containing protein 78	CCDC78			
A1L170	Uncharacterized protein C1orf226	C1orf226			
Q6NZI2	Caveolae-associated protein 1	CAVIN1			
Q8IYT3	Coiled-coil domain-containing protein 170	CCDC170			
Q8IVM0	Coiled-coil domain-containing protein 50	CCDC50			
Q8TBZ0	Coiled-coil domain-containing protein 110	CCDC110			
O95931	Chromobox protein homolog 7	CBX7			
O75871	Carcinoembryonic antigen-related cell adhesion molecule 4	CEACAM4			
Q16739	Ceramide glucosyltransferase	UGCG			
Q8NAS9	Putative uncharacterized protein C5orf17	C5orf17			
O14757	Serine/threonine-protein kinase Chk1	CHEK1			
Q7L3B6	Hsp90 co-chaperone Cdc37-like	CDC37L1			
Q8TD26	Chromodomain-helicase-DNA-binding protein 6	CHD6			
Q86XR8	Centrosomal protein of 57 kDa	CEP57			
A2RU37	Uncharacterized protein encoded by LINC02872	LINC02872			
Q96MH7	Uncharacterized protein C5orf34	C5orf34			
Q9Y4M8	Putative uncharacterized protein encoded by LINC00588	LINC00588			
O95684	Centrosomal protein 43	CEP43			
P21854	B-cell differentiation antigen	CD72			
Q8N1B3	Cyclin-Q	CCNQ			
Q96BT3	Centromere protein T	CENPT			
Q4KMG0	Cell adhesion molecule-related/down-regulated by oncogenes	CDON			
O14519	Cyclin-dependent kinase 2-associated protein 1	CDK2AP1			
O75956	Cyclin-dependent kinase 2-associated protein 2	CDK2AP2			
Q96F05	Uncharacterized protein C11orf24	C11orf24			
Q8NC01	C-type lectin domain family 1 member A	CLEC1A			
Q96SZ6	Mitochondrial tRNA methylthiotransferase CDK5RAP1	CDK5RAP1			
P26992	Ciliary neurotrophic factor receptor subunit alpha	CNTFR			
P08684	Cytochrome P450 3A4	CYP3A4			
A4D0V7	Cadherin-like and PC-esterase domain-containing protein 1	CPED1			
Q8NFW1	Collagen alpha-1	COL22A1			
Q9BSJ5	Uncharacterized protein C17orf80	C17orf80			
Q9BR76	Coronin-1B	CORO1B			
Q9HAK2	Transcription factor COE2	EBF2			
Q53TN4	Cytochrome b reductase 1	CYBRD1			
P08034	Gap junction beta-1 protein	GJB1			
P25025	C-X-C chemokine receptor type 2	CXCR2			
O14625	C-X-C motif chemokine 11	CXCL11			
Q9H4G1	Cystatin-9-like	CST9L			
Q13620	Cullin-4B	CUL4B			
Q9H6A0	DENN domain-containing protein 2D	DENND2D			
Q9H1M4	Beta-defensin 127	DEFB127			
Q30KP8	Beta-defensin 136	DEFB136			
Q6ICB0	Desumoylating isopeptidase 1	DESI1			
Q9UJW3	DNA	DNMT3L			
Q9BUN8	Derlin-1	DERL1			
Q155Q3	Dixin	DIXDC1			

Q9P225	Dynein heavy chain 2, axonemal	DNAH2			
Q9H410	Kinetochore-associated protein DSN1 homolog	DSN1			
O14681	Etoposide-induced protein 2.4 homolog	EI24			
Q8TBF8	Protein FAM81A	FAM81A			
Q6P1L5	Protein FAM117B	FAM117B			
Q8TC76	Protein FAM110B	FAM110B			
Q9NWS6	Protein FAM118A	FAM118A			
Q6UWF9	Protein FAM180A	FAM180A			
Q5BKY9	Protein FAM133B	FAM133B			
P05160	Coagulation factor XIII B chain	F13B			
Q9P2D6	Protein FAM135A	FAM135A			
Q96A26	Protein FAM162A	FAM162A			
A4D161	Protein FAM221A	FAM221A			
Q8TCP9	Protein FAM200A	FAM200A			
Q5U5X8	Protein FAM222A	FAM222A			
Q7Z4H9	Protein FAM220A	FAM220A			
Q5T6X4	Protein FAM162B	FAM162B			
A8MTA8	Protein FAM166B	FAM166B			
A2RU67	Protein FAM234B	FAM234B			
A0PJZ3	Glucoside xylosyltransferase 2	GXYLT2			
P55899	IgG receptor FcRn large subunit p51	FCGRT			
Q86WN1	F-BAR and double SH3 domains protein 1	FCHSD1			
Q8WW38	Zinc finger protein ZFPM2	ZFPM2			
Q00688	Peptidyl-prolyl cis-trans isomerase FKBP3	FKBP3			
Q9Y680	Peptidyl-prolyl cis-trans isomerase FKBP7	FKBP7			
Q86XK2	F-box only protein 11	FBXO11			
Q6ZVX7	F-box only protein 50	NCCRP1			
Q15822	Neuronal acetylcholine receptor subunit alpha-2	CHRNA2			
Q8N6T3	ADP-ribosylation factor GTPase-activating protein 1	ARFGAP1			
P27216	Annexin A13	ANXA13			
Q14155	Rho guanine nucleotide exchange factor 7	ARHGEF7			
O95376	E3 ubiquitin-protein ligase ARIH2	ARIH2			
Q8IWT0	Protein archease	ZBTB8OS			
Q15041	ADP-ribosylation factor-like protein 6-interacting protein 1	ARL6IP1			
Q9BZE9	Tether containing UBX domain for GLUT4	ASPSCR1			
Q9ULH1	Arf-GAP with SH3 domain, ANK repeat and PH domain-containing protein 1	ASAP1			
Q9UBD9	Cardiotrophin-like cytokine factor 1	CLCF1			
Q9NTI2	Phospholipid-transporting ATPase IB	ATP8A2			
Q5T4W7	Artemin	ARTN			
P58397	A disintegrin and metalloproteinase with thrombospondin motifs 12	ADAMTS12			
Q9HBH7	Protein BEX1	BEX1			
Q13490	Baculoviral IAP repeat-containing protein 2	BIRC2			
Q6QNY0	Biogenesis of lysosome-related organelles complex 1 subunit 3	BLOC1S3			
P34820	Bone morphogenetic protein 8B	BMP8B			
Q86UB2	Basic immunoglobulin-like variable motif-containing protein	BIVM			
P19099	Cytochrome P450 11B2, mitochondrial	CYP11B2			
Q5T3Y7	Putative uncharacterized protein BVES-AS1	BVES-AS1			

A4D250	B-cell acute lymphoblastic leukemia-expressed protein	BLACE			
Q6ZW61	Bardet-Biedl syndrome 12 protein	BBS12			
Q9H694	Protein bicaudal C homolog 1	BICC1			
Q7Z7M8	UDP-GlcNAc:betaGal beta-1,3-N-acetylglucosaminyltransferase 8	B3GNT8			
O96024	Beta-1,3-galactosyltransferase 4	B3GALT4			
Q9UPM9	B9 domain-containing protein 1	B9D1			
O95816	BAG family molecular chaperone regulator 2	BAG2			
Q07817	Bcl-2-like protein 1	BCL2L1			
O95817	BAG family molecular chaperone regulator 3	BAG3			
Q02641	Voltage-dependent L-type calcium channel subunit beta-1	CACNB1			
A0A1B0GU71	Uncharacterized protein CFAP97D2	CFAP97D2			
P0DN25	C1GALT1-specific chaperone 1-like protein	C1GALT1C1L			
Q9NX04	Uncharacterized protein C1orf109	C1orf109			
Q5T0J3	Putative uncharacterized protein C1orf220	C1orf220			
Q8TC20	Cancer-associated gene 1 protein	CAGE1			
Q96BQ5	Coiled-coil domain-containing protein 127	CCDC127			
Q5JPI3	Uncharacterized protein C3orf38	C3orf38			
Q567U6	Coiled-coil domain-containing protein 93	CCDC93			
Q8NFR7	Coiled-coil domain-containing protein 148	CCDC148			
Q92583	C-C motif chemokine 17	CCL17			
P35520	Cystathionine beta-synthase	CBS			
P13688	Carcinoembryonic antigen-related cell adhesion molecule 1	CEACAM1			
Q96MT7	Cilia- and flagella-associated protein 44	CFAP44			
P40198	Carcinoembryonic antigen-related cell adhesion molecule 3	CEACAM3			
Q13185	Chromobox protein homolog 3	CBX3			
P0CG36	Cryptic family protein 1B	CFC1B			
Q9NNX6	CD209 antigen	CD209			
Q8WU49	Uncharacterized protein C7orf33	C7orf33			
P25063	Signal transducer CD24	CD24			
Q9H5X1	Cytosolic iron-sulfur assembly component 2A	CIAO2A			
Q6ZUL3	Uncharacterized protein C8orf86	C8orf86			
P0C843	Putative uncharacterized protein encoded by LINC00032	LINC00032			
P41597	C-C chemokine receptor type 2	CCR2			
Q71F23	Centromere protein U	CENPU			
Q96LK0	Centrosomal protein of 19 kDa	CEP19			
Q9P003	Protein cornichon homolog 4	CNIH4			
Q8IXR9	Uncharacterized protein C12orf56	C12orf56			
Q01955	Collagen alpha-3	COL4A3			
Q9BPX3	Condensin complex subunit 3	NCAPG			
Q16281	Cyclic nucleotide-gated cation channel alpha-3	CNGA3			
Q8WXI8	C-type lectin domain family 4 member D	CLEC4D			
Q7Z624	Calmodulin-lysine N-methyltransferase	CAMKMT			
Q96DZ5	CAP-Gly domain-containing linker protein 3	CLIP3			
Q7Z5Q1	Cytoplasmic polyadenylation element-binding protein 2	CPEB2			
Q8IUR6	CREB3 regulatory factor	CREBRF			
Q14061	Cytochrome c oxidase copper chaperone	COX17			
Q9P021	Cysteine-rich PDZ-binding	CRIPT			

Q86W10	Cytochrome P450 4Z1	CYP4Z1			
O75398	Deformed epidermal autoregulatory factor 1 homolog	DEAF1			
Q13117	Deleted in azoospermia protein 2	DAZ2			
O14576	Cytoplasmic dynein 1 intermediate chain 1	DYNC1I1			
Q6ZPD8	Diacylglycerol O-acyltransferase 2-like protein 6	DGAT2L6			
A8MXU0	Putative beta-defensin 108A	DEFB108A			
Q13217	DnaJ homolog subfamily C member 3	DNAJC3			
Q01459	Di-N-acetylchitobiase	CTBS			
P09622	Dihydrolipoyl dehydrogenase, mitochondrial	DLD			
Q9BU89	Deoxyhypusine hydroxylase	DOHH			
Q96Q80	Derlin-3	DERL3			
Q5KSL6	Diacylglycerol kinase kappa	DGKK			
Q96N67	Dedicator of cytokinesis protein 7	DOCK7			
Q7Z5P4	17-beta-hydroxysteroid dehydrogenase 13	HSD17B13			
P59020	Down syndrome critical region protein 9	DSCR9			
Q15075	Early endosome antigen 1	EEA1			
Q14156	Protein EFR3 homolog A	EFR3A			
P08246	Neutrophil elastase	ELANE			
P00533	Epidermal growth factor receptor	EGFR			
Q03111	Protein ENL	MLLT1			
Q8N6I1	EP300-interacting inhibitor of differentiation 2	EID2			
Q52LJ0	Protein FAM98B	FAM98B			
P19447	General transcription and DNA repair factor IIH helicase subunit XPB	ERCC3			
A1L162	Glutamate-rich protein 2	ERICH2			
Q8N5W8	Protein FAM24B	FAM24B			
Q14153	Protein FAM53B	FAM53B			
Q9UBU6	Protein FAM8A1	FAM8A1			
P57679	Ellis-van Creveld syndrome	EVC			
Q9H4H8	Protein FAM83D	FAM83D			
Q8IYI6	Exocyst complex component 8	EXOC8			
A6NMN3	Protein FAM170B	FAM170B			
P50502	Hsc70-interacting protein	ST13			
Q16134	Electron transfer flavoprotein-ubiquinone oxidoreductase, mitochondrial	ETFDH			
Q8NB25	Protein FAM184A	FAM184A			
Q6SJ93	Serine protease FAM111B	FAM111B			
Q9NZB2	Constitutive coactivator of PPAR-gamma-like protein 1	FAM120A			
Q9H5Z6	Protein FAM124B	FAM124B			
Q9Y6I3	Epsin-1	EPN1			
Q8N9E0	Protein FAM133A	FAM133A			
Q9NPD3	Exosome complex component RRP41	EXOSC4			
Q5JX71	Protein FAM209A	FAM209A			
Q63HN1	Putative protein FAM205B	FAM205BP			
Q96BN6	Primary cilium assembly protein FAM149B1	FAM149B1			
Q3B820	Protein FAM161A	FAM161A			
A6NJV1	Protein FAM166C	FAM166C			
P0DMU3	FAM231A/C-like protein LOC102723383				
P42566	Epidermal growth factor receptor substrate 15	EPS15			
H3BQW9	Protein FAM229A	FAM229A			
Q16394	Exostosin-1	EXT1			
P0DPQ4	Protein FAM243B	FAM243B			
A0A494C0Y3	Protein FAM246A	FAM246A			
Q93063	Exostosin-2	EXT2			

Q9NZ08	Endoplasmic reticulum aminopeptidase 1	ERAP1			
Q9P278	Folliculin-interacting protein 2	FNIP2			
Q6JQN1	Acyl-CoA dehydrogenase family member 10	ACAD10			
Q13639	5-hydroxytryptamine receptor 4	HTR4			
O60706	ATP-binding cassette sub-family C member 9	ABCC9			
Q8N2K0	Lysophosphatidylserine lipase ABHD12	ABHD12			
P26439	3 beta-hydroxysteroid dehydrogenase/Delta 5-->4-isomerase type 2	HSD3B2			
O94788	Retinal dehydrogenase 2	ALDH1A2			
P00325	All-trans-retinol dehydrogenase	ADH1B			
P12814	Alpha-actinin-1	ACTN1			
C9JRZ8	Aldo-keto reductase family 1 member B15	AKR1B15			
Q13155	Aminoacyl tRNA synthase complex-interacting multifunctional protein 2	AIMP2			
Q92667	A-kinase anchor protein 1, mitochondrial	AKAP1			
P55197	Protein AF-10	MLLT10			
P06727	Apolipoprotein A-IV	APOA4			
P51825	AF4/FMR2 family member 1	AFF1			
Q8IZP9	Adhesion G-protein coupled receptor G2	ADGRG2			
Q9UJX4	Anaphase-promoting complex subunit 5	ANAPC5			
Q9UJX5	Anaphase-promoting complex subunit 4	ANAPC4			
Q562R1	Beta-actin-like protein 2	ACTBL2			
Q9H0R1	AP-5 complex subunit mu-1	AP5M1			
P0C6C1	Ankyrin repeat domain-containing protein 34C	ANKRD34C			
Q9HCE6	Rho guanine nucleotide exchange factor 10-like protein	ARHGEF10L			
Q6Q4G3	Aminopeptidase Q	LVRN			
Q5TZF3	Ankyrin repeat domain-containing protein 45	ANKRD45			
Q6AI12	Ankyrin repeat domain-containing protein 40	ANKRD40			
O75106	Retina-specific copper amine oxidase	AOC2			
Q9Y2T2	AP-3 complex subunit mu-1	AP3M1			
Q4LE39	AT-rich interactive domain-containing protein 4B	ARID4B			
Q5W041	Armadillo repeat-containing protein 3	ARMC3			
Q5T2E6	Armadillo-like helical domain-containing protein 3	ARMH3			
Q86TY3	Armadillo-like helical domain-containing protein 4	ARMH4			
O60678	Protein arginine N-methyltransferase 3	PRMT3			
Q9UH62	Armadillo repeat-containing X-linked protein 3	ARMCX3			
Q6P1M9	Armadillo repeat-containing X-linked protein 5	ARMCX5			
Q8WXH4	Ankyrin repeat and SOCS box protein 11	ASB11			
Q6XD76	Achaete-scute homolog 4	ASCL4			
P13637	Sodium/potassium-transporting ATPase subunit alpha-3	ATP1A3			
P05026	Sodium/potassium-transporting ATPase subunit beta-1	ATP1B1			
Q4VNC0	Probable cation-transporting ATPase 13A5	ATP13A5			

O15511	Actin-related protein 2/3 complex subunit 5	ARPC5			
Q8N6G6	ADAMTS-like protein 1	ADAMTSL1			
Q8TBG4	Ethanolamine-phosphate phospho-lyase	ETNPPL			
O14965	Aurora kinase A	AURKA			
P27037	Activin receptor type-2A	ACVR2A			
Q6RW13	Type-1 angiotensin II receptor-associated protein	AGTRAP			
Q8WV28	B-cell linker protein	BLNK			
O60566	Mitotic checkpoint serine/threonine-protein kinase BUB1 beta	BUB1B			
Q6NUI1	Putative coiled-coil domain-containing protein 144 N-terminal-like	CCDC144NL			
Q96FH0	BLOC-1-related complex subunit 8	BORCS8			
P78543	Protein BTG2	BTG2			
Q10589	Bone marrow stromal antigen 2	BST2			
Q9H0C5	BTB/POZ domain-containing protein 1	BTBD1			
P59051	Putative uncharacterized protein encoded by BRWD1-AS2	BRWD1-AS2			
Q9NWV8	BRISC and BRCA1-A complex member 1	BABAM1			
Q9HAY6	Beta,beta-carotene 15,15'-dioxygenase	BCO1			
Q96G01	Protein bicaudal D homolog 1	BICD1			
O95415	Brain protein I3	BRI3			
Q9UIF8	Bromodomain adjacent to zinc finger domain protein 2B	BAZ2B			
Q02338	D-beta-hydroxybutyrate dehydrogenase, mitochondrial	BDH1			
Q15878	Voltage-dependent R-type calcium channel subunit alpha-1E	CACNA1E			
Q8WWF1	Uncharacterized protein C1orf54	C1orf54			
Q01518	Adenylyl cyclase-associated protein 1	CAP1			
Q6ZS94	Putative uncharacterized protein C1orf229	C1orf229			
P00915	Carbonic anhydrase 1	CA1			
Q5TG92	Putative uncharacterized protein C1orf195	C1orf195			
O43852	Calumenin	CALU			
Q6P9F0	Coiled-coil domain-containing protein 62	CCDC62			
Q9BV29	Coiled-coil domain-containing protein 32	CCDC32			
Q9BWC9	Coiled-coil domain-containing protein 106	CCDC106			
Q9Y3X0	Coiled-coil domain-containing protein 9	CCDC9			
Q96EE4	Coiled-coil domain-containing protein 126	CCDC126			
P23435	Cerebellin-1	CBLN1			
O15444	C-C motif chemokine 25	CCL25			
Q9P2H0	Centrosomal protein of 126 kDa	CEP126			
Q5SQH8	Uncharacterized protein C6orf136	C6orf136			
Q5TEZ5	Uncharacterized protein C6orf163	C6orf163			
Q9H8X3	Putative uncharacterized protein LINC00574	LINC00574			
Q8NEM8	Cytosolic carboxypeptidase 3	AGBL3			
Q53EZ4	Centrosomal protein of 55 kDa	CEP55			
Q07108	Early activation antigen CD69	CD69			
Q99467	CD180 antigen	CD180			
Q96MT8	Centrosomal protein of 63 kDa	CEP63			
A0A0U1RR11	Centromere protein V-like protein 1	CENPVL1			

Q16667	Cyclin-dependent kinase inhibitor 3	CDKN3			
Q8NHQ1	Centrosomal protein of 70 kDa	CEP70			
O60583	Cyclin-T2	CCNT2			
Q96GE4	Centrosomal protein of 95 kDa	CEP95			
Q9Y2V0	CDAN1-interacting nuclease 1	CDIN1			
Q495D7	Putative uncharacterized protein encoded by LINC01559	LINC01559			
Q32M92	Uncharacterized protein C15orf32	C15orf32			
Q6EIG7	C-type lectin domain family 6 member A	CLEC6A			
Q9Y2B0	Protein canopy homolog 2	CNPY2			
Q8N129	Protein canopy homolog 4	CNPY4			
Q96RK1	Cbp/p300-interacting transactivator 4	CITED4			
Q9H3G5	Probable serine carboxypeptidase CPVL	CPVL			
Q6ZST2	Putative uncharacterized protein COL25A1-DT	COL25A1-DT			
Q9NZW4	Dentin sialophosphoprotein	DSPP			
Q6IAN0	Dehydrogenase/reductase SDR family member 7B	DHRS7B			
Q6UWT2	Adropin	ENHO			
P29317	Ephrin type-A receptor 2	EPHA2			
Q6IA86	Elongator complex protein 2	ELP2			
Q9HC35	Echinoderm microtubule-associated protein-like 4	EML4			
P21860	Receptor tyrosine-protein kinase erbB-3	ERBB3			
A6ND36	Protein FAM83G	FAM83G			
Q5RHP9	Glutamate-rich protein 3	ERICH3			
A6NGS2	Glutamate-rich protein 4	ERICH4			
Q8IV48	3'-5' exoribonuclease 1	ERI1			
Q9UI08	Ena/VASP-like protein	EVL			
Q4KMX7	Protein FAM106A	FAM106A			
Q96PZ2	Serine protease FAM111A	FAM111A			
Q9NX05	Constitutive coactivator of PPAR-gamma-like protein 2	FAM120C			
Q15884	Protein FAM189A2	FAM189A2			
Q5JX69	Protein FAM209B	FAM209B			
P19235	Erythropoietin receptor	EPOR			
Q96AQ9	Protein FAM131C	FAM131C			
Q4W5N1	Putative ATP-binding cassette sub-family A member 11	ABCA11P			
Q8WWZ7	ATP-binding cassette sub-family A member 5	ABCA5			
Q86UQ4	ATP-binding cassette sub-family A member 13	ABCA13			
P28288	ATP-binding cassette sub-family D member 3	ABCD3			
P30154	Serine/threonine-protein phosphatase 2A 65 kDa regulatory subunit A beta isoform	PPP2R1B			
Q16671	Anti-Muellerian hormone type-2 receptor	AMHR2			
Q6NTF7	DNA dC->dU-editing enzyme APOBEC-3H	APOBEC3H			
Q99490	Arf-GAP with GTPase, ANK repeat and PH domain-containing protein 2	AGAP2			
Q5VTM2	Arf-GAP with GTPase, ANK repeat and PH domain-containing protein 9	AGAP9			
A6NIR3	Arf-GAP with GTPase, ANK repeat and PH domain-containing protein 5	AGAP5			
Q9BXJ7	Protein amnionless	AMN			
Q9BRR6	ADP-dependent glucokinase	ADPGK			
Q86UN6	A-kinase anchor protein 14	AKAP14			

Q13023	A-kinase anchor protein 6	AKAP6			
Q96QF7	Acidic repeat-containing protein	GCNA			
O75078	Disintegrin and metalloproteinase domain-containing protein 11	ADAM11			
O43423	Acidic leucine-rich nuclear phosphoprotein 32 family member C	ANP32C			
E5RJM6	Ankyrin repeat domain-containing protein 65	ANKRD65			
Q9BYV1	Alanine--glyoxylate aminotransferase 2, mitochondrial	AGXT2			
O95704	Amyloid-beta A4 precursor protein-binding family B member 3	APBB3			
Q9Y6Q5	AP-1 complex subunit mu-2	AP1M2			
P02647	Apolipoprotein A-I	APOA1			
Q9NXU5	ADP-ribosylation factor-like protein 15	ARL15			
O94989	Rho guanine nucleotide exchange factor 15	ARHGEF15			
Q68CP9	AT-rich interactive domain-containing protein 2	ARID2			
Q92527	Ankyrin repeat domain-containing protein 7	ANKRD7			
Q6ZU67	BEN domain-containing protein 4	BEND4			
P35613	Basigin	BSG			
Q9P0X4	Voltage-dependent T-type calcium channel subunit alpha-1I	CACNA1I			
Q9BQE9	B-cell CLL/lymphoma 7 protein family member B	BCL7B			
O75309	Cadherin-16	CDH16			
P27797	Calreticulin	CALR			
Q8NFZ8	Cell adhesion molecule 4	CADM4			
Q9BXJ2	Complement C1q tumor necrosis factor-related protein 7	C1QTNF7			
P56202	Cathepsin W	CTSW			
Q6P9G0	Cytochrome b5 domain-containing protein 1	CYB5D1			
Q86UP0	Cadherin-24	CDH24			
Q5T440	Putative transferase CAF17, mitochondrial	IBA57			
P07498	Kappa-casein	CSN3			
Q9H6F5	Coiled-coil domain-containing protein 86	CCDC86			
Q8IW40	Coiled-coil domain-containing protein 103	CCDC103			
Q96CT7	Coiled-coil domain-containing protein 124	CCDC124			
Q9BUN5	Coiled-coil domain-containing protein 28B	CCDC28B			
Q8TC90	Coiled-coil domain-containing glutamate-rich protein 1	CCER1			
Q6ZUS6	Coiled-coil domain-containing protein 149	CCDC149			
Q7Z4U5	Uncharacterized protein C6orf201	C6orf201			
Q9Y3E7	Charged multivesicular body protein 3	CHMP3			
Q9ULG3	Uncharacterized protein CFAP92	CFAP92			
Q12834	Cell division cycle protein 20 homolog	CDC20			
P06276	Cholinesterase	BCHE			
Q9H5L9	Putative uncharacterized protein C5orf66	C5orf66			
Q96K31	Uncharacterized protein C8orf76	C8orf76			
P55273	Cyclin-dependent kinase 4 inhibitor D	CDKN2D			
Q7Z7J9	Calcium/calmodulin-dependent protein kinase II inhibitor 1	CAMK2N1			

A0A1B0GVH6	Uncharacterized protein C13orf42	C13orf42			
P0C842	Putative uncharacterized protein encoded by LINC00614	LINC00614			
Q9Y696	Chloride intracellular channel protein 4	CLIC4			
Q92989	Polyribonucleotide 5'-hydroxyl-kinase Clp1	CLP1			
Q96Q77	Calcium and integrin-binding family member 3	CIB3			
O95833	Chloride intracellular channel protein 3	CLIC3			
Q8N3C7	CAP-Gly domain-containing linker protein 4	CLIP4			
Q5HYK3	2-methoxy-6-polyprenyl-1,4-benzoquinol methylase, mitochondrial	COQ5			
Q96MF6	Coenzyme Q-binding protein COQ10 homolog A, mitochondrial	COQ10A			
P20853	Cytochrome P450 2A7	CYP2A7			
Q99627	COP9 signalosome complex subunit 8	COPS8			
Q8IUH2	Protein CREG2	CREG2			
P23508	Colorectal mutant cancer protein	MCC			
O43809	Cleavage and polyadenylation specificity factor subunit 5	NUDT21			
Q9UGL9	Cysteine-rich C-terminal protein 1	CRCT1			
Q6XLA1	Protein CASC2, isoform 3	CASC2			
O95727	Cytotoxic and regulatory T-cell molecule	CRTAM			
A6NJJ6	UPF0575 protein C19orf67	C19orf67			
A0A1B0GUS0	Uncharacterized protein C19orf85	C19orf85			
Q9BZJ0	Crooked neck-like protein 1	CRNKL1			
Q5JRM2	Uncharacterized protein CXorf66	CXorf66			
Q9P1W3	Calcium permeable stress-gated cation channel 1	TMEM63C			
Q96LM9	Uncharacterized protein C20orf173	C20orf173			
P35222	Catenin beta-1	CTNNB1			
A4FU28	cTAGE family member 9	CTAGE9			
Q9UIA0	Cytohesin-4	CYTH4			
Q7LFL8	CXXC-type zinc finger protein 5	CXXC5			
O43739	Cytohesin-3	CYTH3			
Q6ZN03	Putative uncharacterized protein encoded by LINC00322	LINC00322			
Q9H1P6	Uncharacterized protein C20orf85	C20orf85			
P01037	Cystatin-SN	CST1			
Q9NUB4	Uncharacterized protein C20orf141	C20orf141			
Q9BQM9	Uncharacterized protein C20orf144	C20orf144			
Q6P5X5	UPF0545 protein C22orf39	C22orf39			
Q13618	Cullin-3	CUL3			
Q9NUU7	ATP-dependent RNA helicase DDX19A	DDX19A			
Q9UN19	Dual adapter for phosphotyrosine and 3-phosphotyrosine and 3-phosphoinositide	DAPP1			
Q9NX09	DNA damage-inducible transcript 4 protein	DDIT4			
P51530	DNA replication ATP-dependent helicase/nuclease DNA2	DNA2			
Q13316	Dentin matrix acidic phosphoprotein 1	DMP1			
P26358	DNA	DNMT1			
P28845	Corticosteroid 11-beta-dehydrogenase isozyme 1	HSD11B1			

P55265	Double-stranded RNA-specific adenosine deaminase	ADAR			
Q7Z589	BRCA2-interacting transcriptional repressor EMSY	EMSY			
Q86VI1	Exocyst complex component 3-like protein	EXOC3L1			
P52797	Ephrin-A3	EFNA3			
Q6ZVH7	Espin-like protein	ESPNL			
A8MXJ8	Putative protein FAM90A5P	FAM90A5P			
Q9UQ84	Exonuclease 1	EXO1			
P19652	Alpha-1-acid glycoprotein 2	ORM2			
Q96AP0	Adrenocortical dysplasia protein homolog	ACD			
Q13541	Eukaryotic translation initiation factor 4E-binding protein 1	EIF4EBP1			
O95264	5-hydroxytryptamine receptor 3B	HTR3B			
P40394	All-trans-retinol dehydrogenase	ADH7			
Q9NP70	Ameloblastin	AMBN			
O43572	A-kinase anchor protein 10, mitochondrial	AKAP10			
O95076	Homeobox protein aristaless-like 3	ALX3			
P02768	Albumin	ALB			
P18825	Alpha-2C adrenergic receptor	ADRA2C			
Q9NQ90	Anoctamin-2	ANO2			
P50583	Bis	NUDT2			
Q96PC3	AP-1 complex subunit sigma-3	AP1S3			
P30533	Alpha-2-macroglobulin receptor-associated protein	LRPAP1			
Q7Z628	Neuroepithelial cell-transforming gene 1 protein	NET1			
Q96C12	Armadillo repeat-containing protein 5	ARMC5			
Q9BY76	Angiopoietin-related protein 4	ANGPTL4			
Q8IUR7	Armadillo repeat-containing protein 8	ARMC8			
Q9UMQ3	Homeobox protein BarH-like 2	BARX2			
Q8N535	Putative uncharacterized protein encoded by LINC00471	LINC00471			
Q5JW98	Calcium homeostasis modulator protein 4	CALHM4			
Q5EG05	Caspase recruitment domain-containing protein 16	CARD16			
Q96MC9	Uncharacterized protein C1orf147	C1orf147			
Q9P1Z2	Calcium-binding and coiled-coil domain-containing protein 1	CALCOCO1			
Q9NR16	Scavenger receptor cysteine-rich type 1 protein M160	CD163L1			
Q5JVX7	Uncharacterized protein C1orf141	C1orf141			
Q9H251	Cadherin-23	CDH23			
O75973	C1q-related factor	C1QL1			
B7Z1M9	C2 calcium-dependent domain-containing protein 4D	C2CD4D			
P05937	Calbindin	CALB1			
Q9BX69	Caspase recruitment domain-containing protein 6	CARD6			
Q9UNH5	Dual specificity protein phosphatase CDC14A	CDC14A			
Q4V339	COBW domain-containing protein 6	CBWD6			
Q5T9S5	Coiled-coil domain-containing protein 18	CCDC18			
Q96GN5	Cell division cycle-associated 7-like protein	CDCA7L			
Q49A88	Coiled-coil domain-containing protein 14	CCDC14			
Q8N1V2	Cilia- and flagella-associated protein 52	CFAP52			
P0CG37	Cryptic protein	CFC1			

Q9H9P2	Chondrolectin	CHODL			
P16671	Platelet glycoprotein 4	CD36			
Q08722	Leukocyte surface antigen CD47	CD47			
Q8NET6	Carbohydrate sulfotransferase 13	CHST13			
Q9H2J1	Uncharacterized protein ARRDC1-AS1	ARRDC1-AS1			
P38936	Cyclin-dependent kinase inhibitor 1	CDKN1A			
Q6ZRZ4	Uncharacterized protein C9orf47	C9orf47			
Q5VTT2	Protein C9orf135	C9orf135			
Q9BXL8	Cell division cycle-associated protein 4	CDCA4			
Q9UK58	Cyclin-L1	CCNL1			
P06126	T-cell surface glycoprotein CD1a	CD1A			
Q9Y5P4	Ceramide transfer protein	CERT1			
O76076	CCN family member 5	CCN5			
P78396	Cyclin-A1	CCNA1			
C9JDV5	Putative uncharacterized protein C12orf77	C12orf77			
Q9Y2R0	Cytochrome c oxidase assembly factor 3 homolog, mitochondrial	COA3			
O95628	CCR4-NOT transcription complex subunit 4	CNOT4			
Q9UFF9	CCR4-NOT transcription complex subunit 8	CNOT8			
Q9NS75	Cysteinyl leukotriene receptor 2	CYSLTR2			
O94983	Calmodulin-binding transcription activator 2	CAMTA2			
Q14406	Chorionic somatomammotropin hormone-like 1	CSHL1			
P0DMU7	Cancer/testis antigen family 45 member A6	CT45A6			
Q8WUE5	Cancer/testis antigen 55	CT55			
P12074	Cytochrome c oxidase subunit 6A1, mitochondrial	COX6A1			
Q9BQA9	Cytochrome b-245 chaperone 1	CYBC1			
Q8WVB6	Chromosome transmission fidelity protein 18 homolog	CHTF18			
O43246	Cationic amino acid transporter 4	SLC7A4			
Q9BWK5	Cell cycle regulator of non-homologous end joining	CYREN			
Q8N907	DAN domain family member 5	DAND5			
P0C7V8	DDB1- and CUL4-associated factor 8-like protein 2	DCAF8L2			
Q9NQZ3	Deleted in azoospermia protein 1	DAZ1			
Q6E0U4	Dermokine	DMKN			
O76075	DNA fragmentation factor subunit beta	DFFB			
Q9H1M3	Beta-defensin 129	DEFB129			
Q96C10	Probable ATP-dependent RNA helicase DHX58	DHX58			
Q99615	DnaJ homolog subfamily C member 7	DNAJC7			
Q9BPX1	17-beta-hydroxysteroid dehydrogenase 14	HSD17B14			
Q15398	Disks large-associated protein 5	DLGAP5			
Q5F1R6	DnaJ homolog subfamily C member 21	DNAJC21			
P14061	17-beta-hydroxysteroid dehydrogenase type 1	HSD17B1			
Q9Y2G8	DnaJ homolog subfamily C member 16	DNAJC16			
P51452	Dual specificity protein phosphatase 3	DUSP3			
Q19T08	Endothelial cell-specific chemotaxis regulator	ECSCR			
Q9UII6	Dual specificity protein phosphatase 13 isoform B	DUSP13			
Q9NZN4	EH domain-containing protein 2	EHD2			

Q9Y2G0	Protein EFR3 homolog B	EFR3B			
P0DP91	Chimeric ERCC6-PGBD3 protein	ERCC6			
P28715	DNA repair protein complementing XP-G cells	ERCC5			
P03372	Estrogen receptor	ESR1			
P36639	7,8-dihydro-8-oxoguanine triphosphatase	NUDT1			
Q9UBZ4	DNA-	APEX2			
A6NGH8	Ankyrin repeat domain-containing protein 61	ANKRD61			
O00213	Amyloid-beta A4 precursor protein-binding family B member 1	APBB1			
Q96BM9	ADP-ribosylation factor-like protein 8A	ARL8A			
Q7Z2E3	Aprataxin	APTX			
P04114	Apolipoprotein B-100	APOB			
P08519	Apolipoprotein	LPA			
Q68DC2	Ankyrin repeat and SAM domain-containing protein 6	ANKS6			
Q9H8Y5	Ankyrin repeat and zinc finger domain-containing protein 1	ANKZF1			
Q9C0F0	Putative Polycomb group protein ASXL3	ASXL3			
Q9HD20	Endoplasmic reticulum transmembrane helix translocase	ATP13A1			
Q9GZN4	Brain-specific serine protease 4	PRSS22			
O95861	3'	BPNT1			
P62324	Protein BTG1	BTG1			
P48751	Anion exchange protein 3	SLC4A3			
Q9UHQ4	B-cell receptor-associated protein 29	BCAP29			
Q9NRL2	Bromodomain adjacent to zinc finger domain protein 1A	BAZ1A			
Q8NDZ0	BEN domain-containing protein 2	BEND2			
Q8N3J6	Cell adhesion molecule 2	CADM2			
Q5T5A4	Protein C1orf194	C1orf194			
Q3MIR4	Cell cycle control protein 50B	TMEM30B			
Q8N715	Coiled-coil domain-containing protein 185	CCDC185			
Q9Y698	Voltage-dependent calcium channel gamma-2 subunit	CACNG2			
P51671	Eotaxin	CCL11			
Q9C0D2	Centrosomal protein of 295 kDa	CEP295			
P80098	C-C motif chemokine 7	CCL7			
Q9H444	Charged multivesicular body protein 4b	CHMP4B			
Q99653	Calcineurin B homologous protein 1	CHP1			
Q9UEE9	Craniofacial development protein 1	CFDP1			
Q96N11	Uncharacterized protein C7orf26	C7orf26			
Q9C0I3	Serine-rich coiled-coil domain-containing protein 1	CCSER1			
P28906	Hematopoietic progenitor cell antigen CD34	CD34			
P09326	CD48 antigen	CD48			
P30279	G1/S-specific cyclin-D2	CCND2			
Q96LM1	Putative uncharacterized protein encoded by LINC00615	LINC00615			
P07357	Complement component C8 alpha chain	C8A			
P15391	B-lymphocyte antigen CD19	CD19			
O75829	Leukocyte cell-derived chemotaxin 1	CNMD			
Q8N812	Uncharacterized protein C12orf76	C12orf76			
Q96JP9	Cadherin-related family member	CDHR1			
P27658	Collagen alpha-1	COL8A1			
Q8NA57	Uncharacterized protein C12orf50	C12orf50			

Q8WYK1	Contactin-associated protein-like 5	CNTNAP5			
Q9C0C6	CLOCK-interacting pacemaker	CIPC			
Q86T13	C-type lectin domain family 14 member A	CLEC14A			
O75122	CLIP-associating protein 2	CLASP2			
Q8NE01	Metal transporter CNNM3	CNNM3			
Q9NXG0	Centlein	CNTLN			
Q8N6V4	UPF0728 protein C10orf53	C10orf53			
Q96F83	Uncharacterized protein CLBA1	CLBA1			
P56277	Cx9C motif-containing protein 4	CMC4			
O75838	Calcium and integrin-binding family member 2	CIB2			
P01911	HLA class II histocompatibility antigen, DRB1 beta chain	HLA-DRB1			
P13762	HLA class II histocompatibility antigen, DR beta 4 chain	HLA-DRB4			
A0PK11	Clarin-2	CLRN2			
P35523	Chloride channel protein 1	CLCN1			
Q96DY2	Dynein regulatory complex protein 10	IQCD			
Q15329	Transcription factor E2F5	E2F5			
Q86XH1	Dynein regulatory complex protein 11	IQCA1			
Q13627	Dual specificity tyrosine-phosphorylation-regulated kinase 1A	DYRK1A			
O75461	Transcription factor E2F6	E2F6			
Q96MC2	Dynein regulatory complex protein 1	DRC1			
Q9Y463	Dual specificity tyrosine-phosphorylation-regulated kinase 1B	DYRK1B			
Q16643	Drebrin	DBN1			
Q96AV8	Transcription factor E2F7	E2F7			
Q9BQC3	-2	DPH2			
P33316	Deoxyuridine 5'-triphosphate nucleotidohydrolase, mitochondrial	DUT			
Q8IXS2	Dynein regulatory complex subunit 2	CCDC65			
Q86XF0	Dihydrofolate reductase 2, mitochondrial	DHFR2			
Q9Y295	Developmentally-regulated GTP-binding protein 1	DRG1			
Q9H069	Dynein regulatory complex subunit 3	DRC3			
A6NFE3	EF-hand calcium-binding domain-containing protein 10	EFCAB10			
Q9H4G8	Putative DPH3 homolog B	DPH3P1			
A0AVK6	Transcription factor E2F8	E2F8			
Q9BUY7	EF-hand calcium-binding domain-containing protein 11	EFCAB11			
Q6NXP0	EF-hand calcium-binding domain-containing protein 12	EFCAB12			
Q8IY85	EF-hand calcium-binding domain-containing protein 13	EFCAB13			
P55039	Developmentally-regulated GTP-binding protein 2	DRG2			
O75071	EF-hand calcium-binding domain-containing protein 14	EFCAB14			
O95995	Dynein regulatory complex subunit 4	GAS8			
Q96FX2	DPH3 homolog	DPH3			
P60507	Endogenous retrovirus group FC1 Env polyprotein	ERVFC1			
Q92630	Dual specificity tyrosine-phosphorylation-regulated kinase 2	DYRK2			

A6NNA5	Dorsal root ganglia homeobox protein	DRGX			
P60608	Endogenous retrovirus group FC1 member 1 Env polyprotein	ERVFC1-1			
Q6PGQ1	Aspartate-rich protein 1	DRICH1			
Q5JU00	Dynein regulatory complex subunit 5	TCTE1			
Q86YF9	Zinc finger protein DZIP1	DZIP1			
P09172	Dopamine beta-hydroxylase	DBH			
Q86Y13	E3 ubiquitin-protein ligase DZIP3	DZIP3			
Q8N4Y2	EF-hand calcium-binding domain-containing protein 4A	CRACR2B			
O43781	Dual specificity tyrosine-phosphorylation-regulated kinase 3	DYRK3			
Q8NEE6	Dynein regulatory complex subunit 6	FBXL13			
Q9BSW2	EF-hand calcium-binding domain-containing protein 4B	CRACR2A			
Q9NR20	Dual specificity tyrosine-phosphorylation-regulated kinase 4	DYRK4			
Q9HAE3	EF-hand calcium-binding domain-containing protein 1	EFCAB1			
Q8IY82	Dynein regulatory complex subunit 7	DRC7			
Q8N7B9	EF-hand calcium-binding domain-containing protein 3	EFCAB3			
Q5VUJ9	Dynein regulatory complex protein 8	EFCAB2			
A4FU69	EF-hand calcium-binding domain-containing protein 5	EFCAB5			
Q5THR3	EF-hand calcium-binding domain-containing protein 6	EFCAB6			
Q9H095	Dynein regulatory complex protein 9	IQCG			
Q9H2P9	Diphthine methyl ester synthase	DPH5			
A8K855	EF-hand calcium-binding domain-containing protein 7	EFCAB7			
A8MWE9	EF-hand calcium-binding domain-containing protein 8	EFCAB8			
A8MZ26	EF-hand calcium-binding domain-containing protein 9	EFCAB9			
P21728	D	DRD1			
P00374	Dihydrofolate reductase	DHFR			
Q9HA90	EF-hand and coiled-coil domain-containing protein 1	EFCC1			
Q99704	Docking protein 1	DOK1			
P0DPD8	EEF1AKMT4-ECE2 readthrough transcript protein	EEF1AKMT 4-ECE2			
Q9HAF1	Chromatin modification-related protein MEAF6	MEAF6			
Q6ZTU2	Putative EP400-like protein	EP400P1			
O60496	Docking protein 2	DOK2			
Q9H4G0	Band 4.1-like protein 1	EPB41L1			
Q7L591	Docking protein 3	DOK3			
Q8TEW6	Docking protein 4	DOK4			
P14416	D	DRD2			
O43491	Band 4.1-like protein 2	EPB41L2			
Q9P104	Docking protein 5	DOK5			
Q6PKX4	Docking protein 6	DOK6			
Q9Y2J2	Band 4.1-like protein 3	EPB41L3			
Q18PE1	Protein Dok-7	DOK7			
P35462	D	DRD3			
O95905	Protein ecdysoneless homolog	ECD			
Q9HCM4	Band 4.1-like protein 5	EPB41L5			
Q8IYY4	Zinc finger protein DZIP1L	DZIP1L			
P39059	Collagen alpha-1	COL15A1			
Q17RW2	Collagen alpha-1	COL24A1			

Q5T8D3	Acyl-CoA-binding domain-containing protein 5	ACBD5			
P20848	Putative alpha-1-antitrypsin-related protein	SERPINA2			
P27348	14-3-3 protein theta	YWHAQ			
Q96P26	Cytosolic 5'-nucleotidase 1B	NT5C1B			
P28332	Alcohol dehydrogenase 6	ADH6			
O75891	Cytosolic 10-formyltetrahydrofolate dehydrogenase	ALDH1L1			
Q8N6M5	Probable allantoicase	ALLC			
P05062	Fructose-bisphosphate aldolase B	ALDOB			
C9J069	Apical junction component 1 homolog	AJM1			
Q96IF1	LIM domain-containing protein ajuba	AJUBA			
O43918	Autoimmune regulator	AIRE			
Q9Y2D5	A-kinase anchor protein 2	AKAP2			
Q01433	AMP deaminase 2	AMPD2			
Q01432	AMP deaminase 3	AMPD3			
Q3KRA9	Alpha-ketoglutarate-dependent dioxygenase alkB homolog 6	ALKBH6			
Q9ULX6	A-kinase anchor protein 8-like	AKAP8L			
Q9NXW9	Alpha-ketoglutarate-dependent dioxygenase alkB homolog 4	ALKBH4			
Q9Y3Q7	Disintegrin and metalloproteinase domain-containing protein 18	ADAM18			
O95490	Adhesion G protein-coupled receptor L2	ADGRL2			
P68032	Actin, alpha cardiac muscle 1	ACTC1			
P54922		ADPRH			
Q86YJ7	Ankyrin repeat domain-containing protein 13B	ANKRD13B			
P50995	Annexin A11	ANXA11			
P60006	Anaphase-promoting complex subunit 15	ANAPC15			
Q92481	Transcription factor AP-2-beta	TFAP2B			
Q9C0C7	Activating molecule in BECN1-regulated autophagy protein 1	AMBRA1			
P0DTE8	Alpha-amylase 1C	AMY1C			
P03950	Angiogenin	ANG			
O43827	Angiopoietin-related protein 7	ANGPTL7			
Q9Y264	Angiopoietin-4	ANGPT4			
Q6PIY5	Armadillo-like helical domain containing protein 1	ARMH1			
Q6ICH7	Aspartate beta-hydroxylase domain-containing protein 2	ASPHD2			
Q8IZT6	Abnormal spindle-like microcephaly-associated protein	ASPM			
Q96KQ4	Apoptosis-stimulating of p53 protein 1	PPP1R13B			
P0C7U1	Putative inactive neutral ceramidase B	ASAH2B			
Q7L266	Isoaspartyl peptidase/L-asparaginase	ASRGL1			
Q8N9N2	Activating signal cointegrator 1 complex subunit 1	ASCC1			
Q9BSB4	Autophagy-related protein 101	ATG101			
O75185	Calcium-transporting ATPase type 2C member 2	ATP2C2			
P23634	Plasma membrane calcium-transporting ATPase 4	ATP2B4			
O15145	Actin-related protein 2/3 complex subunit 3	ARPC3			
P51795	H	CLCN5			
O95832	Claudin-1	CLDN1			
O95484	Claudin-9	CLDN9			
P03928	ATP synthase protein 8	MT-ATP8			

O43861	Probable phospholipid-transporting ATPase IIB	ATP9B			
Q58HT5	Acyl-CoA wax alcohol acyltransferase 1	AWAT1			
Q5VV63	Attractin-like protein 1	ATRNL1			
Q6ZW13	Uncharacterized protein C16orf86	C16orf86			
Q9UKP5	A disintegrin and metalloproteinase with thrombospondin motifs 6	ADAMTS6			
O14981	TATA-binding protein-associated factor 172	BTAF1			
Q7Z5Y6	Bone morphogenetic protein 8A	BMP8A			
O00238	Bone morphogenetic protein receptor type-1B	BMPR1B			
Q06187	Tyrosine-protein kinase BTK	BTK			
Q9H3K6	BolA-like protein 2	BOLA2			
Q69YU5	Protein BRAWNIN	BRAWNIN			
Q8N9W6	Protein boule-like	BOLL			
Q7Z569	BRCA1-associated protein	BRAP			
Q00973	Beta-1,4 N-acetylgalactosaminyltransferase 1	B4GALNT1			
P51572	B-cell receptor-associated protein 31	BCAP31			
Q5SZJ8	BEN domain-containing protein 6	BEND6			
O14874		BCKDK			
P47755	F-actin-capping protein subunit alpha-2	CAPZA2			
Q86XJ0	Calcium homeostasis modulator protein 3	CALHM3			
Q6UXQ4	Uncharacterized protein C2orf66	C2orf66			
A6NN90	Uncharacterized protein C2orf81	C2orf81			
P55285	Cadherin-6	CDH6			
Q8NEQ5	Transmembrane protein C1orf162	C1orf162			
P54289	Voltage-dependent calcium channel subunit alpha-2/delta-1	CACNA2D1			
P02746	Complement C1q subcomponent subunit B	C1QB			
Q7Z692	Carcinoembryonic antigen-related cell adhesion molecule 19	CEACAM19			
Q14002	Carcinoembryonic antigen-related cell adhesion molecule 7	CEACAM7			
Q5T1J5	Putative coiled-coil-helix-coiled-coil-helix domain-containing protein CHCHD2P9, mitochondrial	CHCHD2P9			
P09693	T-cell surface glycoprotein CD3 gamma chain	CD3G			
Q6ZRC1	Uncharacterized protein C4orf50	C4orf50			
Q4W4Y0	Uncharacterized protein C14orf28	C14orf28			
Q96M20	Cyclic nucleotide-binding domain-containing protein 2	CNBD2			
Q96DG6	Carboxymethylenebutenolidase homolog	CMBL			
P08572	Collagen alpha-2	COL4A2			
A8MV24	Uncharacterized protein C17orf98	C17orf98			
Q9Y3A0	Ubiquinone biosynthesis protein COQ4 homolog, mitochondrial	COQ4			
Q68DL7	Uncharacterized protein C18orf63	C18orf63			
Q6ZWL3	Cytochrome P450 4V2	CYP4V2			
P04141	Granulocyte-macrophage colony-stimulating factor	CSF2			
P00156	Cytochrome b	MT-CYB			
Q6QHC5	Sphingolipid delta	DEGS2			
Q9UDY4	DnaJ homolog subfamily B member 4	DNAJB4			
Q9GZP9	Derlin-2	DERL2			
P0CG12	Decreased expression in renal and prostate cancer protein	DERPC			
Q8TDM6	Disks large homolog 5	DLG5			

Q8TBM8	DnaJ homolog subfamily B member 14	DNAJB14			
O75165	DnaJ homolog subfamily C member 13	DNAJC13			
O95661	GTP-binding protein Di-Ras3	DIRAS3			
Q969H9	Disrupted in renal carcinoma protein 1	DIRC1			
P57055	Protein ripply3	RIPPLY3			
Q9NRF9	DNA polymerase epsilon subunit	POLE3			
Q008S8	Epithelial cell-transforming sequence 2 oncogene-like	ECT2L			
Q9H4B8	Dipeptidase 3	DPEP3			
P51808	Dynein light chain Tctex-type 3	DYNLT3			
Q9UJ72	Annexin A10	ANXA10			
Q5T9G4	Armadillo repeat-containing protein 12	ARMC12			
Q99856	AT-rich interactive domain-containing protein 3A	ARID3A			
H3BNL8	Armadillo-like helical domain-containing protein 2	ARMH2			
Q9H0Y0	Ubiquitin-like-conjugating enzyme ATG10	ATG10			
P61160	Actin-related protein 2	ACTR2			
Q9UHC3	Acid-sensing ion channel 3	ASIC3			
Q5FYA8	Arylsulfatase H	ARSH			
Q5FYB1	Arylsulfatase I	ARSI			
Q92485	Acid sphingomyelinase-like phosphodiesterase 3b	SMPDL3B			
Q9BPX5	Actin-related protein 2/3 complex subunit 5-like protein	ARPC5L			
P51797	Chloride transport protein 6	CLCN6			
Q8N6F1	Claudin-19	CLDN19			
Q9NQS1	Cell death regulator Aven	AVEN			
Q9H1Z8	Augurin	ECRG4			
P35670	Copper-transporting ATPase 2	ATP7B			
Q9Y281	Cofilin-2	CFL2			
A6NMY6	Putative annexin A2-like protein	ANXA2P2			
P56381	ATP synthase subunit epsilon, mitochondrial	ATP5F1E			
Q92858	Protein atonal homolog 1	ATOH1			
P54132	Bloom syndrome protein	BLM			
Q8N8U9	BMP-binding endothelial regulator protein	BMPER			
O60238	BCL2/adenovirus E1B 19 kDa protein-interacting protein 3-like	BNIP3L			
Q13075	Baculoviral IAP repeat-containing protein 1	NAIP			
P25440	Bromodomain-containing protein 2	BRD2			
Q92934	Bcl2-associated agonist of cell death	BAD			
Q9Y2C3	Beta-1,3-galactosyltransferase 5	B3GALT5			
Q9Y2A9	N-acetyllactosaminide beta-1,3-N-acetylglucosaminyltransferase 3	B3GNT3			
P63098	Calcineurin subunit B type 1	PPP3R1			
Q93088	Betaine--homocysteine S-methyltransferase 1	BHMT			
Q9UMQ6	Calpain-11	CAPN11			
Q9NRR8	CDC42 small effector protein 1	CDC42SE1			
A2AJT9	BCLAF1 and THRAP3 family member 3	BCLAF3			
Q8NDD1	Uncharacterized protein C1orf131	C1orf131			
Q9NSG2	Uncharacterized protein C1orf112	C1orf112			
Q6ZSJ8	Uncharacterized protein C1orf122	C1orf122			
P02747	Complement C1q subcomponent subunit C	C1QC			
Q9P1Y5	Calmodulin-regulated spectrin-associated protein 3	CAMSAP3			

B2RNN3	Complement C1q and tumor necrosis factor-related protein 9B	C1QTNF9B			
Q03135	Caveolin-1	CAV1			
Q76M96	Coiled-coil domain-containing protein 80	CCDC80			
Q8N1I8	Putative uncharacterized protein encoded by CACTIN-AS1	CACTIN-AS1			
Q9ULU8	Calcium-dependent secretion activator 1	CADPS			
P30988	Calcitonin receptor	CALCR			
F5H4A9	Uncharacterized membrane protein C3orf80	C3orf80			
Q6UXS9	Inactive caspase-12	CASP12			
A0A0D9SF12	Transmembrane protein CCDC163	CCDC163			
P13500	C-C motif chemokine 2	CCL2			
P0C7I6	Coiled-coil domain-containing protein 159	CCDC159			
Q16627	C-C motif chemokine 14	CCL14			
Q8IYX8	Centrosomal protein CEP57L1	CEP57L1			
Q9NTU7	Cerebellin-4	CBLN4			
P15086	Carboxypeptidase B	CPB1			
Q9BRT8	COBW domain-containing protein 1	CBWD1			
Q9NRG0	Chromatin accessibility complex protein 1	CHRAC1			
P15882	N-chimaerin	CHN1			
P49716	CCAAT/enhancer-binding protein delta	CEBPD			
Q7Z6I8	UPF0461 protein C5orf24	C5orf24			
P0DMQ9	Putative uncharacterized protein C8orf89	C8orf89			
A4D0T2	Uncharacterized protein C7orf66	C7orf66			
O14646	Chromodomain-helicase-DNA-binding protein 1	CHD1			
Q9HCK8	Chromodomain-helicase-DNA-binding protein 8	CHD8			
Q8N1A6	UPF0462 protein C4orf33	C4orf33			
Q14094	Cyclin-I	CCNI			
A0A0U1RRI6	Centromere protein V-like protein 3	CENPVL3			
Q07002	Cyclin-dependent kinase 18	CDK18			
P11802	Cyclin-dependent kinase 4	CDK4			
Q9GZX3	Carbohydrate sulfotransferase 6	CHST6			
Q9NZN8	CCR4-NOT transcription complex subunit 2	CNOT2			
Q96F85	CB1 cannabinoid receptor-interacting protein 1	CNRIP1			
C9JLR9	Uncharacterized protein C11orf95	C11orf95			
A8K7I4	Calcium-activated chloride channel regulator 1	CLCA1			
P02462	Collagen alpha-1	COL4A1			
P29973	cGMP-gated cation channel alpha-1	CNGA1			
Q9BT09	Protein canopy homolog 3	CNPY3			
O00230	Cortistatin	CORT			
Q6Q6R5	Cysteine-rich protein 3	CRIP3			
Q6RUI8	Uncharacterized protein C19orf48	C19orf48			
Q9NVV2	Putative uncharacterized protein C19orf73	C19orf73			
Q8IUI8	Cytokine receptor-like factor 3	CRLF3			
Q13536	Protein CROC-4	MIR9-1HG			
Q6YFQ2	Cytochrome c oxidase subunit 6B2	COX6B2			
P14854	Cytochrome c oxidase subunit 6B1	COX6B1			
Q8WWM9	Cytoglobin	CYGB			
Q8NEC5	Cation channel sperm-associated protein 1	CATSPER1			
Q6PJP8	DNA cross-link repair 1A protein	DCLRE1A			
P12838	Neutrophil defensin 4	DEFA4			

Q9BUQ8	Probable ATP-dependent RNA helicase DDX23	DDX23			
Q96EP5	DAZ-associated protein 1	DAZAP1			
P24855	Deoxyribonuclease-1	DNASE1			
P25686	DnaJ homolog subfamily B member 2	DNAJB2			
Q9UBX3	Mitochondrial dicarboxylate	SLC25A10			
P25685	DnaJ homolog subfamily B member 1	DNAJB1			
P49184	Deoxyribonuclease-1-like 1	DNASE1L1			
Q8WUY9	DEP domain-containing protein	DEPDC1B			
O14490	Disks large-associated protein 1	DLGAP1			
Q9Y4D2	Diacylglycerol lipase-alpha	DAGLA			
Q92506	Estradiol 17-beta-dehydrogenase 8	HSD17B8			
Q9UQ10	Trans-1,2-dihydrobenzene-1,2-diol dehydrogenase	DHDH			
O60869	Endothelial differentiation-related factor 1	EDF1			
P32926	Desmoglein-3	DSG3			
Q86UW9	Probable E3 ubiquitin-protein ligase DTX2	DTX2			
Q8NEJ0	Dual specificity protein phosphatase 18	DUSP18			
Q8TD84	Down syndrome cell adhesion molecule-like protein 1	DSCAML1			
Q8NCM8	Cytoplasmic dynein 2 heavy chain 1	DYNC2H1			
Q9BVJ7	Dual specificity protein phosphatase 23	DUSP23			
P63172	Dynein light chain Tctex-type 1	DYNLT1			
Q7Z5Q5	DNA polymerase nu	POLN			
P55199	RNA polymerase II elongation factor ELL	ELL			
Q9UKU7	Isobutyryl-CoA dehydrogenase, mitochondrial	ACAD8			
Q16570	Atypical chemokine receptor 1	ACKR1			
A0PJZ0	Putative ankyrin repeat domain-containing protein 20A5	ANKRD20A5P			
P30926	Neuronal acetylcholine receptor subunit beta-4	CHRNB4			
Q96IX9	Putative ankyrin repeat domain-containing protein 26-like 1	ANKRD36BP1			
Q8NHS2	Putative aspartate aminotransferase, cytoplasmic 2	GOT1L1			
P33897	ATP-binding cassette sub-family D member 1	ABCD1			
P08908	5-hydroxytryptamine receptor 1A	HTR1A			
Q9UG63	ATP-binding cassette sub-family F member 2	ABCF2			
Q5JWF8	Actin-like protein 10	ACTL10			
Q6ZNF0	Acid phosphatase type 7	ACP7			
Q13362	Serine/threonine-protein phosphatase 2A 56 kDa regulatory subunit gamma isoform	PPP2R5C			
O60503	Adenylate cyclase type 9	ADCY9			
Q14246	Adhesion G protein-coupled receptor E1	ADGRE1			
P30556	Type-1 angiotensin II receptor	AGTR1			
Q8IVF6	Ankyrin repeat domain-containing protein 18A	ANKRD18A			
Q8N2N9	Ankyrin repeat domain-containing protein 36B	ANKRD36B			
O14727	Apoptotic protease-activating factor 1	APAF1			
Q53RE8	Ankyrin repeat domain-containing protein 39	ANKRD39			

Q8NB46	Serine/threonine-protein phosphatase 6 regulatory ankyrin repeat subunit C	ANKRD52			
Q9Y689	ADP-ribosylation factor-like protein 5A	ARL5A			
P04083	Annexin A1	ANXA1			
Q13520	Aquaporin-6	AQP6			
Q96PS8	Aquaporin-10	AQP10			
Q9P291	Armadillo repeat-containing X-linked protein 1	ARMCX1			
P18850	Cyclic AMP-dependent transcription factor ATF-6 alpha	ATF6			
P18847	Cyclic AMP-dependent transcription factor ATF-3	ATF3			
P00966	Argininosuccinate synthase	ASS1			
A6NEK1	Arrestin domain-containing protein 5	ARRDC5			
P54253	Ataxin-1	ATXN1			
P25705	ATP synthase subunit alpha, mitochondrial	ATP5F1A			
P56748	Claudin-8	CLDN8			
Q04656	Copper-transporting ATPase 1	ATP7A			
O15169	Axin-1	AXIN1			
P82987	ADAMTS-like protein 3	ADAMTSL3			
A8MY62	Putative beta-lactamase-like 1	LACTBL1			
Q96KE9	BTB/POZ domain-containing protein 6	BTBD6			
A8MVZ5	Butyrophilin-like protein 10	BTNL10			
Q6ZT62	Bargin	BARGIN			
Q9Y3E2	BolA-like protein 1	BOLA1			
Q99933	BAG family molecular chaperone regulator 1	BAG1			
Q8N1M1	Bestrophin-3	BEST3			
Q9UIF9	Bromodomain adjacent to zinc finger domain protein 2A	BAZ2A			
Q9NP86	Calcium-binding protein 5	CABP5			
Q96LS8	Putative uncharacterized protein C2orf48	C2orf48			
Q9BXJ3	Complement C1q tumor necrosis factor-related protein 4	C1QTNF4			
Q5T5S1	Coiled-coil domain-containing protein 183	CCDC183			
Q5RIA9	COBW domain-containing protein 5	CBWD5			
Q8N319	Uncharacterized protein C6orf223	C6orf223			
Q99459	Cell division cycle 5-like protein	CDC5L			
O75419	Cell division control protein 45 homolog	CDC45			
Q9Y259	Choline/ethanolamine kinase	CHKB			
P36222	Chitinase-3-like protein 1	CHI3L1			
Q9C0F1	Centrosomal protein of 44 kDa	CEP44			
Q8IZ16	Uncharacterized protein C7orf61	C7orf61			
Q99675	Cell growth regulator with RING finger domain protein 1	CGRRF1			
Q8WZB0	Putative uncharacterized protein encoded by LINC00476	LINC00476			
Q13352	Centromere protein R	ITGB3BP			
Q00526	Cyclin-dependent kinase 3	CDK3			
Q15642	Cdc42-interacting protein 4	TRIP10			
Q5T4I8	Putative uncharacterized protein C6orf52	C6orf52			
A6NJI1	Uncharacterized protein C11orf86	C11orf86			
Q9H3H3	UPF0696 protein C11orf68	C11orf68			
Q96LI5	CCR4-NOT transcription complex subunit 6-like	CNOT6L			
Q8IZR5	CKLF-like MARVEL transmembrane domain-containing protein 4	CMTM4			
Q8TCG1	Protein CIP2A	CIP2A			

Q9H6Q4	Cytosolic iron-sulfur assembly component 3	CIAO3			
O75503	Ceroid-lipofuscinosis neuronal protein 5	CLN5			
Q9H6B4	CXADR-like membrane protein	CLMP			
Q96MW5	Conserved oligomeric Golgi complex subunit 8	COG8			
P53674	Beta-crystallin B1	CRYBB1			
Q07092	Collagen alpha-1	COL16A1			
Q9HB55	Cytochrome P450 3A43	CYP3A43			
P01189	Pro-opiomelanocortin	POMC			
O95639	Cleavage and polyadenylation specificity factor subunit 4	CPSF4			
O75131	Copine-3	CPNE3			
Q2UY09	Collagen alpha-1	COL28A1			
Q8IZJ3	C3 and PZP-like alpha-2-macroglobulin domain-containing protein 8	CPAMD8			
P59665	Neutrophil defensin 1	DEFA1			
Q9BW61	DET1- and DDB1-associated protein 1	DDA1			
Q6UX07	Dehydrogenase/reductase SDR family member 13	DHRS13			
Q9UHL4	Dipeptidyl peptidase 2	DPP7			
Q9P2D7	Dynein heavy chain 1, axonemal	DNAH1			
Q9NY33	Dipeptidyl peptidase 3	DPP3			
P0CJ88	Double homeobox protein 4-like protein 5	DUX4L5			
Q96PT3	Double homeobox protein 5	DUX5			
O77932	Decapping and exoribonuclease protein	DXO			
Q13838	Spliceosome RNA helicase DDX39B	DDX39B			
C9JQL5	Putative dispanin subfamily A member 2d				
Q8NBJ4	Golgi membrane protein 1	GOLM1			
Q8N766	ER membrane protein complex subunit 1	EMC1			
Q9H221	ATP-binding cassette sub-family G member 8	ABCG8			
P08172	Muscarinic acetylcholine receptor M2	CHRM2			
Q9NPB9	Atypical chemokine receptor 4	ACKR4			
Q86V21	Acetoacetyl-CoA synthetase	AACS			
Q04917	14-3-3 protein eta	YWHAH			
P29274	Adenosine receptor A2a	ADORA2A			
Q86WK6	Amphoterin-induced protein 1	AMIGO1			
O15204	ADAM DEC1	ADAMDEC			
Q7Z4H4	Protein ADM2	ADM2			
Q0VDE8	Adipogenin	ADIG			
P00326	Alcohol dehydrogenase 1C	ADH1C			
P35612	Beta-adducin	ADD2			
P35609	Alpha-actinin-2	ACTN2			
Q9NZN9	Aryl-hydrocarbon-interacting protein-like 1	AIPL1			
Q9UHK6	Alpha-methylacyl-CoA racemase	AMACR			
O75969	A-kinase anchor protein 3	AKAP3			
Q495B1	Ankyrin repeat and death domain-containing protein 1A	ANKDD1A			
Q96JD6	1,5-anhydro-D-fructose	AKR1E2			
Q2TAA5	GDP-Man:Man	ALG11			
Q4L235	Beta-alanine-activating enzyme	AASDH			
Q4G176	Malonate--CoA ligase ACSF3, mitochondrial	ACSF3			
O00116	Alkyldihydroxyacetonephosphate synthase, peroxisomal	AGPS			
Q8N4X5	Actin filament-associated protein 1-like 2	AFAP1L2			
Q9H9L7	Akirin-1	AKIRIN1			

Q9UHB7	AF4/FMR2 family member 4	AFF4			
Q08AH1	Acyl-coenzyme A synthetase ACSM1, mitochondrial	ACSM1			
O43184	Disintegrin and metalloproteinase domain-containing protein 12	ADAM12			
P13945	Beta-3 adrenergic receptor	ADRB3			
Q96K78	Adhesion G-protein coupled receptor G7	ADGRG7			
P60709	Actin, cytoplasmic 1	ACTB			
Q9BYX7	Putative beta-actin-like protein 3	POTEKP			
P35368	Alpha-1B adrenergic receptor	ADRA1B			
Q96DE5	Anaphase-promoting complex subunit 16	ANAPC16			
Q8J025	Protein APCDD1	APCDD1			
Q6ZTN6	Ankyrin repeat domain-containing protein 13D	ANKRD13D			
P84077	ADP-ribosylation factor 1	ARF1			
P02655	Apolipoprotein C-II	APOC2			
Q13367	AP-3 complex subunit beta-2	AP3B2			
Q5T9A4	ATPase family AAA domain-containing protein 3B	ATAD3B			
Q86TH1	ADAMTS-like protein 2	ADAMTSL2			
Q6UWY0	Arylsulfatase K	ARSK			
Q14CW9	Ataxin-7-like protein 3	ATXN7L3			
Q06055	ATP synthase F	ATP5MC2			
Q8NBU5	Outer mitochondrial transmembrane helix translocase	ATAD1			
P98198	Phospholipid-transporting ATPase ID	ATP8B2			
P51790	H	CLCN3			
P06576	ATP synthase subunit beta, mitochondrial	ATP5F1B			
Q9UQB9	Aurora kinase C	AURKC			
Q8WXE1	ATR-interacting protein	ATRIP			
Q6PH81	UPF0547 protein C16orf87	C16orf87			
O95971	CD160 antigen	CD160			
Q96LC9	Bcl-2-modifying factor	BMF			
Q00587	Cdc42 effector protein 1	CDC42EP1			
Q5XKL5	BTB/POZ domain-containing protein 8	BTBD8			
P16278	Beta-galactosidase	GLB1			
Q8NFQ6	BPI fold-containing family C protein	BPIFC			
Q14201	Protein BTG3	BTG3			
P0C853	Putative uncharacterized protein BAALC-AS2	BAALC-AS2			
P15056	Serine/threonine-protein kinase B-raf	BRAF			
Q67FW5	UDP-GlcNAc:betaGal beta-1,3-N-acetylglucosaminyltransferase-like protein 1	B3GNTL1			
Q5TH69	Brefeldin A-inhibited guanine nucleotide-exchange protein 3	ARFGEF3			
Q86Y28	B melanoma antigen 4	BAGE4			
Q9BTV7	CDK5 and ABL1 enzyme substrate 2	CABLES2			
O75952	Calcium-binding tyrosine phosphorylation-regulated protein	CABYR			
P07384	Calpain-1 catalytic subunit	CAPN1			
A0A0U1RR37	Uncharacterized protein C1orf232	C1orf232			
Q9ULB5	Cadherin-7	CDH7			
Q5T5Y3	Calmodulin-regulated spectrin-associated protein 1	CAMSAP1			
Q2TBE0	CWF19-like protein 2	CWF19L2			
Q9ULB4	Cadherin-9	CDH9			
Q8IWD4	Coiled-coil domain-containing protein 117	CCDC117			

A6NLC5	UPF0524 protein C3orf70	C3orf70			
A0A1B0GTI1	Coiled-coil domain-containing protein 201	CCDC201			
Q86V15	Zinc finger protein castor homolog 1	CASZ1			
P32238	Cholecystokinin receptor type A	CCKAR			
P0C221	Coiled-coil domain-containing protein 175	CCDC175			
Q6ZUS5	Coiled-coil domain-containing protein 121	CCDC121			
P42081	T-lymphocyte activation antigen CD86	CD86			
Q6ZRI6	Uncharacterized protein C15orf39	C15orf39			
Q9HAW4	Claspin	CLSPN			
Q9BYD5	Cornifelin	CNFN			
P33552	Cyclin-dependent kinases regulatory subunit 2	CKS2			
Q6PI25	Protein cornichon homolog 2	CNIH2			
Q12887	Protoheme IX farnesyltransferase, mitochondrial	COX10			
P78329	Cytochrome P450 4F2	CYP4F2			
O43889	Cyclic AMP-responsive element-binding protein 3	CREB3			
Q8TAV3	Cytochrome P450 2W1	CYP2W1			
Q9BQ39	ATP-dependent RNA helicase DDX50	DDX50			
Q8TF63	Dendritic cell nuclear protein 1	DCANP1			
Q8N8A6	ATP-dependent RNA helicase DDX51	DDX51			
Q8IWE4	DCN1-like protein 3	DCUN1D3			
P61962	DDB1- and CUL4-associated factor 7	DCAF7			
A2VCK2	Doublecortin domain-containing protein 2B	DCDC2B			
Q30KQ7	Beta-defensin 113	DEFB113			
Q9Y5R5	Doublesex- and mab-3-related transcription factor 2	DMRT2			
P17661	Desmin	DES			
Q9GZS0	Dynein intermediate chain 2, axonemal	DNAI2			
Q9UBS3	DnaJ homolog subfamily B member 9	DNAJB9			
Q86YH6	All trans-polyprenyl-diphosphate synthase PDSS2	PDSS2			
Q8NBA8	tRNA-uridine aminocarboxypropyltransferase 2	DTWD2			
Q9UKB3	DnaJ homolog subfamily C member 12	DNAJC12			
Q8IVF4	Dynein heavy chain 10, axonemal	DNAH10			
P08697	Alpha-2-antiplasmin	SERPINF2			
Q5QJU3	Alkaline ceramidase 2	ACER2			
Q9NX38	Protein Abitram	ABITRAM			
Q86UK0	ATP-binding cassette sub-family A member 12	ABCA12			
Q9NRA8	Eukaryotic translation initiation factor 4E transporter	EIF4ENIF1			
O60239	SH3 domain-binding protein 5	SH3BP5			
P19021	Peptidyl-glycine alpha-amidating monooxygenase	PAM			
P45381	Aspartoacylase	ASPA			
Q8N7J2	APC membrane recruitment protein 2	AMER2			
Q8IY45	Protein AMN1 homolog	AMN1			
O43707	Alpha-actinin-4	ACTN4			
O43687	A-kinase anchor protein 7 isoforms alpha and beta	AKAP7			
Q9BYD9	Actin-related protein T3	ACTRT3			
Q9UKV8	Protein argonaute-2	AGO2			
Q6ZNB7	Alkylglycerol monooxygenase	AGMO			

P51816	AF4/FMR2 family member 2	AFF2			
Q9Y6B7	AP-4 complex subunit beta-1	AP4B1			
Q9NYG5	Anaphase-promoting complex subunit 11	ANAPC11			
Q9Y587	AP-4 complex subunit sigma-1	AP4S1			
Q2VPB7	AP-5 complex subunit beta-1	AP5B1			
Q8IVW1	ADP-ribosylation factor-like protein 17	ARL17A			
P13928	Annexin A8	ANXA8			
P27695	DNA-	APEX1			
Q8N6D5	Ankyrin repeat domain-containing protein 29	ANKRD29			
Q8N726	Tumor suppressor ARF	CDKN2A			
Q8N2F6	Armadillo repeat-containing protein 10	ARMC10			
P16157	Ankyrin-1	ANK1			
Q5VTE6	Protein angel homolog 2	ANGEL2			
Q9UNK9	Protein angel homolog 1	ANGEL1			
Q8NAG6	Ankyrin repeat and LEM domain-containing protein 1	ANKLE1			
Q5H9R4	Armadillo repeat-containing X-linked protein 4	ARMCX4			
Q7L311	Armadillo repeat-containing X-linked protein 2	ARMCX2			
P17544	Cyclic AMP-dependent transcription factor ATF-7	ATF7			
Q2TB18	Protein asteroid homolog 1	ASTE1			
P14415	Sodium/potassium-transporting ATPase subunit beta-2	ATP1B2			
Q8IYS0	Protein Aster-C	GRAMD1C			
Q8IYA2	Putative coiled-coil domain-containing protein 144C	CCDC144CP			
P46663	B1 bradykinin receptor	BDKRB1			
Q9Y6E2	Basic leucine zipper and W2 domain-containing protein 2	BZW2			
P20290	Transcription factor BTF3	BTF3			
Q9GZL8	Putative BPES syndrome breakpoint region protein	BPESC1			
Q6Y288	Beta-1,3-glucosyltransferase	B3GLCT			
P59052	Putative uncharacterized protein B3GALT5-AS1	B3GALT5-AS1			
Q96L58	Beta-1,3-galactosyltransferase 6	B3GALT6			
Q9P287	BRCA2 and CDKN1A-interacting protein	BCCIP			
Q9BUH8	Brain-enriched guanylate kinase-associated protein	BEGAIN			
P35219	Carbonic anhydrase-related protein	CA8			
P01036	Cystatin-S	CST4			
Q8IU99	Calcium homeostasis modulator protein 1	CALHM1			
Q9NZU7	Calcium-binding protein 1	CABP1			
Q969G5	Caveolae-associated protein 3	CAVIN3			
Q9H246	Uncharacterized protein C1orf21	C1orf21			
O95561	Uncharacterized protein C1orf105	C1orf105			
O14815	Calpain-9	CAPN9			
Q8NC38	Putative uncharacterized protein ZNF436-AS1	ZNF436-AS1			
P08311	Cathepsin G	CTSG			
Q6ZUJ4	Uncharacterized protein C3orf62	C3orf62			
P0DN24	Uncharacterized protein C3orf86	C3orf86			
P31944	Caspase-14	CASP14			
Q5VZK9	F-actin-uncapping protein LRRC16A	CARMIL1			
P04040	Catalase	CAT			
Q9NQR7	Coiled-coil domain-containing protein 177	CCDC177			
Q9UNI1	Chymotrypsin-like elastase family member 1	CELA1			

Q9NYQ6	Cadherin EGF LAG seven-pass G-type receptor 1	CELSR1			
P0DN86	Choriogonadotropin subunit beta 3	CGB3			
Q5TBE3	Uncharacterized protein C9orf153	C9orf153			
Q00532	Cyclin-dependent kinase-like 1	CDKL1			
Q9Y6H1	Coiled-coil-helix-coiled-coil-helix domain-containing protein 2	CHCHD2			
Q5VXM1	CUB domain-containing protein 2	CDCP2			
Q9Y240	C-type lectin domain family 11 member A	CLEC11A			
Q5T0Z8	Uncharacterized protein C6orf132	C6orf132			
Q969H4	Connector enhancer of kinase suppressor of ras 1	CNKSR1			
Q8WXI2	Connector enhancer of kinase suppressor of ras 2	CNKSR2			
Q8TAV5	Putative uncharacterized protein C11orf45	C11orf45			
A0A1B0GUT2	Uncharacterized protein C10orf143	C10orf143			
Q9H2X3	C-type lectin domain family 4 member M	CLEC4M			
P10909	Clusterin	CLU			
Q9P2M7	Cingulin	CGN			
A0A1B0GUA9	Uncharacterized protein C13orf46	C13orf46			
Q96JQ2	Calmin	CLMN			
Q96AQ7	Cell death activator CIDE-3	CIDEC			
Q96NY7	Chloride intracellular channel protein 6	CLIC6			
Q9NR63	Cytochrome P450 26B1	CYP26B1			
Q7KZN9	Cytochrome c oxidase assembly protein COX15 homolog	COX15			
A0A1B0GUU1	Transmembrane protein C17orf113	C17orf113			
Q0VDD5	Putative uncharacterized protein encoded by MIR22HG	MIR22HG			
Q6ZS62	Colorectal cancer-associated protein 1	COLCA1			
Q9H3Y0	Peptidase inhibitor R3HDML	R3HDML			
Q96KJ9	Cytochrome c oxidase subunit 4 isoform 2, mitochondrial	COX4I2			
O75718	Cartilage-associated protein	CRTAP			
Q32NC0	UPF0711 protein C18orf21	C18orf21			
A2RUU4	Colipase-like protein 1	CLPSL1			
Q5JQC4	Cancer/testis antigen 47A	CT47A1			
A6NM66	Uncharacterized protein encoded by LINC01548	LINC01548			
P17302	Gap junction alpha-1 protein	GJA1			
O75638	Cancer/testis antigen 2	CTAG2			
P57773	Gap junction alpha-9 protein	GJA9			
P49447	Cytochrome b561	CYB561			
Q96KN9	Gap junction delta-4 protein	GJD4			
Q9UKL4	Gap junction delta-2 protein	GJD2			
Q9H2A7	C-X-C motif chemokine 16	CXCL16			
Q9H7T0	Cation channel sperm-associated protein subunit beta	CATSPERB			
Q5T1A1	DC-STAMP domain-containing protein 2	DCST2			
Q08495	Dematin	DMTN			
Q6ZN54	Differentially expressed in FDCP 8 homolog	DEF8			
P98082	Disabled homolog 2	DAB2			
Q9H0S4	Probable ATP-dependent RNA helicase DDX47	DDX47			
Q8N5I4	Dehydrogenase/reductase SDR family member on chromosome X	DHRSX			
Q09013	Myotonin-protein kinase	DMPK			

Q8IWG1	Dynein intermediate chain 3, axonemal	DNAI3			
Q9Y485	DmX-like protein 1	DMXL1			
Q0P6D2	Divergent protein kinase domain 1C	DIPK1C			
Q9BTC0	Death-inducer obliterator 1	DIDO1			
Q13574	Diacylglycerol kinase zeta	DGKZ			
Q709F0	Acyl-CoA dehydrogenase family member 11	ACAD11			
P01009	Alpha-1-antitrypsin	SERPINA1			
Q07912	Activated CDC42 kinase 1	TNK2			
O15254	Peroxisomal acyl-coenzyme A oxidase 3	ACOX3			
Q96I13	Protein ABHD8	ABHD8			
Q9ULZ1	Apelin	APLN			
Q8IW19	Aprataxin and PNK-like factor	APLF			
Q9H9E1	Ankyrin repeat family A protein 2	ANKRA2			
Q5VYY1	Ankyrin repeat domain-containing protein 22	ANKRD22			
A6NF34	Anthrax toxin receptor-like	ANTXRL			
Q86W74	Ankyrin repeat domain-containing protein 46	ANKRD46			
P04746	Pancreatic alpha-amylase	AMY2A			
P16860	Natriuretic peptides B	NPPB			
Q7Z5J8	Ankyrin and armadillo repeat-containing protein	ANKAR			
Q8WXK4	Ankyrin repeat and SOCS box protein 12	ASB12			
L0R819	ASNSD1 upstream open reading frame protein	ASDURF			
Q4VNC1	Probable cation-transporting ATPase 13A4	ATP13A4			
P16615	Sarcoplasmic/endoplasmic reticulum calcium ATPase 2	ATP2A2			
P50993	Sodium/potassium-transporting ATPase subunit alpha-2	ATP1A2			
Q96SQ7	Protein atonal homolog 8	ATOH8			
Q7L1Q6	Basic leucine zipper and W2 domain-containing protein 1	BZW1			
Q13410	Butyrophilin subfamily 1 member A1	BTN1A1			
O15392	Baculoviral IAP repeat-containing protein 5	BIRC5			
O43684	Mitotic checkpoint protein BUB3	BUB3			
P51813	Cytoplasmic tyrosine-protein kinase BMX	BMX			
Q9BWV1	Brother of CDO	BOC			
Q9HB09	Bcl-2-like protein 12	BCL2L12			
O95696	Bromodomain-containing protein 1	BRD1			
O00154	Cytosolic acyl coenzyme A thioester hydrolase	ACOT7			
Q8WY36	HMG box transcription factor BBX	BBX			
Q86Y27	B melanoma antigen 5	BAGE5			
Q8TD16	Protein bicaudal D homolog 2	BICD2			
Q9UBW5	Bridging integrator 2	BIN2			
Q8WU43	Uncharacterized protein C2orf15	C2orf15			
P20807	Calpain-3	CAPN3			
Q5R3K3	Calcium homeostasis modulator protein 6	CALHM6			
P20749	B-cell lymphoma 3 protein	BCL3			
O43497	Voltage-dependent T-type calcium channel subunit alpha-1G	CACNA1G			
P57730	Caspase recruitment domain-containing protein 18	CARD18			
Q5NE16	Putative inactive cathepsin L-like protein CTSL3P	CTSL3P			
Q4G0S4	Cytochrome P450 27C1	CYP27C1			
P55212	Caspase-6	CASP6			

Q6ZTR7	CBY1-interacting BAR domain-containing protein 2	CIBAR2			
Q9BR77	Coiled-coil domain-containing protein 77	CCDC77			
P55211	Caspase-9	CASP9			
A6NGH7	Coiled-coil domain-containing protein 160	CCDC160			
Q9H6E4	Coiled-coil domain-containing protein 134	CCDC134			
A6NF36	Coiled-coil domain-containing protein 182	CCDC182			
Q8NEF3	Coiled-coil domain-containing protein 112	CCDC112			
Q8NDH2	Coiled-coil domain-containing protein 168	CCDC168			
Q8NHW4	C-C motif chemokine 4-like	CCL4L1			
P08185	Corticosteroid-binding globulin	SERPINA6			
Q2WEN9	Carcinoembryonic antigen-related cell adhesion molecule 16	CEACAM16			
Q8IUF1	COBW domain-containing protein 2	CBWD2			
Q15744	CCAAT/enhancer-binding protein epsilon	CEBPE			
P15813	Antigen-presenting glycoprotein CD1d	CD1D			
Q5VXU3	Cysteine-rich hydrophobic domain-containing protein 1	CHIC1			
O95674	Phosphatidate cytidylyltransferase 2	CDS2			
Q9H211	DNA replication factor Cdt1	CDT1			
Q15517	Corneodesmosin	CDSN			
O14618	Copper chaperone for superoxide dismutase	CCS			
Q504U0	Renal cancer differentiation gene 1 protein	C4orf46			
Q9H7E9	UPF0488 protein C8orf33	C8orf33			
Q9H5F2	UPF0686 protein C11orf1	C11orf1			
Q6P4Q7	Metal transporter CNNM4	CNNM4			
Q8N3K9	Cardiomyopathy-associated protein 5	CMYA5			
Q9NQ32	Uncharacterized protein C11orf16	C11orf16			
Q6UXN8	C-type lectin domain family 9 member A	CLEC9A			
P53420	Collagen alpha-4	COL4A4			
Q8IZ96	CKLF-like MARVEL transmembrane domain-containing protein 1	CMTM1			
Q8WXE0	Caskin-2	CASKIN2			
O95741	Copine-6	CPNE6			
Q9BV73	Centrosome-associated protein CEP250	CEP250			
Q8N4C9	Uncharacterized protein C17orf78	C17orf78			
Q8N436	Inactive carboxypeptidase-like protein X2	CPXM2			
O14936	Peripheral plasma membrane protein CASK	CASK			
Q2VPA4	Complement component receptor 1-like protein	CR1L			
O75881	Cytochrome P450 7B1	CYP7B1			
Q8NBC4	Uncharacterized protein C20orf203	C20orf203			
Q9P2B4	CTTNBP2 N-terminal-like protein	CTTNBP2N			
Q5T4H9	Putative uncharacterized protein encoded by MIR1915-HG	MIR1915HG			
Q969M2	Gap junction alpha-10 protein	GJA10			
Q9UJ94	Putative uncharacterized protein encoded by LINC00527	LINC00527			
Q9UBT7	Alpha-catulin	CTNNAL1			

Q2PZI1	Probable C-mannosyltransferase DPY19L1	DPY19L1			
A8MYA2	Uncharacterized protein CXorf49	CXorf49;			
P13498	Cytochrome b-245 light chain	CYBA			
Q05048	Cleavage stimulation factor subunit 1	CSTF1			
Q12996	Cleavage stimulation factor subunit 3	CSTF3			
Q6ZV56	Putative uncharacterized protein C22orf34	C22orf34			
Q13617	Cullin-2	CUL2			
O94850	Dendrin	DDN			
Q96PD2	Discoidin, CUB and LCCL domain-containing protein 2	DCBLD2			
Q30KQ9	Beta-defensin 110	DEFB110			
Q30KQ5	Beta-defensin 115	DEFB115			
Q9Y5R6	Doublesex- and mab-3-related transcription factor 1	DMRT1			
Q6P158	Putative ATP-dependent RNA helicase DHX57	DHX57			
O96015	Dynein light chain 4, axonemal	DNAL4			
Q8IXT2	Doublesex- and mab-3-related transcription factor C2	DMRTC2			
Q96EY1	DnaJ homolog subfamily A member 3, mitochondrial	DNAJA3			
P18858	DNA ligase 1	LIG1			
Q8WYQ5	Microprocessor complex subunit DGCR8	DGCR8			
P80370	Protein delta homolog 1	DLK1			
Q14184	Double C2-like domain-containing protein beta	DOC2B			
Q07507	Dermatopontin	DPT			
Q16760	Diacylglycerol kinase delta	DGKD			
P23743	Diacylglycerol kinase alpha	DGKA			
Q9Y6T7	Diacylglycerol kinase beta	DGKB			
Q8TF09	Dynein light chain roadblock-type 2	DYNLRB2			
Q9NXW2	DnaJ homolog subfamily B member 12	DNAJB12			
Q5SXM8	DNL-type zinc finger protein	DNLZ			
O00429	Dynamin-1-like protein	DNM1L			
Q9P265	Disco-interacting protein 2 homolog B	DIP2B			
Q96IM9	DPY30 domain-containing protein 2	DYDC2			
Q8TEA8	D-aminoacyl-tRNA deacylase 1	DTD1			
P0CJ89	Double homeobox protein 4-like protein 6	DUX4L6			
Q9NRW4	Dual specificity protein phosphatase 22	DUSP22			
Q9H4I9	Essential MCU regulator, mitochondrial	SMDT1			
P50402	Emerin	EMD			
Q8WYP5	Protein ELYS	AHCTF1			
Q9BY44	Eukaryotic translation initiation factor 2A	EIF2A			
Q2M3D2	Exocyst complex component 3-like protein 2	EXOC3L2			
Q86TX2	Acyl-coenzyme A thioesterase 1	ACOT1			
Q13542	Eukaryotic translation initiation factor 4E-binding protein 2	EIF4EBP2			
Q9NP58	ATP-binding cassette sub-family B member 6, mitochondrial	ABCB6			
Q16537	Serine/threonine-protein phosphatase 2A 56 kDa regulatory subunit epsilon isoform	PPP2R5E			
Q70Z44	5-hydroxytryptamine receptor 3D	HTR3D			
Q8WW27	Putative C->U-editing enzyme APOBEC-4	APOBEC4			

Q96BJ3	Axin interactor, dorsalization-associated protein	AIDA			
Q9H8T0	AKT-interacting protein	AKTIP			
Q9Y2D8	Afadin- and alpha-actinin-binding protein	SSX2IP			
Q6DCA0	AMMECR1-like protein	AMMECR1L			
P06280	Alpha-galactosidase A	GLA			
Q92688	Acidic leucine-rich nuclear phosphoprotein 32 family member B	ANP32B			
Q92870	Amyloid-beta A4 precursor protein-binding family B member 2	APBB2			
P16066	Atrial natriuretic peptide receptor 1	NPR1			
O43747	AP-1 complex subunit gamma-1	AP1G1			
Q9NR22	Protein arginine N-methyltransferase 8	PRMT8			
Q9ULJ7	Ankyrin repeat domain-containing protein 50	ANKRD50			
P0DTE7	Alpha-amylase 1B	AMY1B			
P09525	Annexin A4	ANXA4			
Q9NU02	Ankyrin repeat and EF-hand domain-containing protein 1	ANKEF1			
Q9NR81	Rho guanine nucleotide exchange factor 3	ARHGEF3			
Q8WWN8	Arf-GAP with Rho-GAP domain, ANK repeat and PH domain-containing protein 3	ARAP3			
O43150	Arf-GAP with SH3 domain, ANK repeat and PH domain-containing protein 2	ASAP2			
Q9ULZ3	Apoptosis-associated speck-like protein containing a CARD	PYCARD			
Q8N3C0	Activating signal cointegrator 1 complex subunit 3	ASCC3			
P54793	Arylsulfatase F	ARSF			
P51690	Arylsulfatase L	ARSL			
P46597	Acetylserotonin O-methyltransferase	ASMT			
P18440	Arylamine N-acetyltransferase 1	NAT1			
Q9ULK2	Ataxin-7-like protein 1	ATXN7L1			
Q9Y2Q0	Phospholipid-transporting ATPase IA	ATP8A1			
O43520	Phospholipid-transporting ATPase IC	ATP8B1			
P51793	H	CLCN4			
P78369	Claudin-10	CLDN10			
O15265	Ataxin-7	ATXN7			
Q8TE58	A disintegrin and metalloproteinase with thrombospondin motifs 15	ADAMTS15			
Q9Y6H3	Mitochondrial inner membrane protease ATP23 homolog	ATP23			
Q8WXF7	Atlastin-1	ATL1			
Q9NWD9	Protein BEX4	BEX4			
P12644	Bone morphogenetic protein 4	BMP4			
O43683	Mitotic checkpoint serine/threonine-protein kinase BUB1	BUB1			
P02730	Band 3 anion transport protein	SLC4A1			
Q16548	Bcl-2-related protein A1	BCL2A1			
Q9C0J1	N-acetyllactosaminide beta-1,3-N-acetylglucosaminyltransferase 4	B3GNT4			
Q8NCR0	UDP-GalNAc:beta-1,3-N-acetylgalactosaminyltransferase 2	B3GALNT2			
Q9BXU9	Calcium-binding protein 8	CALN1			
A6NCI8	Uncharacterized protein C2orf78	C2orf78			

P21730	C5a anaphylatoxin chemotactic receptor 1	C5AR1			
A6NCS6	Uncharacterized protein C2orf72	C2orf72			
P0DPF6	Putative uncharacterized protein CDRT15P3	CDRT15P3			
Q5SNV9	Uncharacterized protein C1orf167	C1orf167			
Q12864	Cadherin-17	CDH17			
Q9BXJ5	Complement C1q tumor necrosis factor-related protein 2	C1QTNF2			
Q9Y376	Calcium-binding protein 39	CAB39			
Q96NT0	Coiled-coil domain-containing protein 115	CCDC115			
Q7Z6B0	Coiled-coil domain-containing protein 91	CCDC91			
Q9BRK5	45 kDa calcium-binding protein	SDF4			
Q9H257	Caspase recruitment domain-containing protein 9	CARD9			
A4D256	Dual specificity protein phosphatase CDC14C	CDC14C			
Q9NV96	Cell cycle control protein 50A	TMEM30A			
Q96PX6	Coiled-coil domain-containing protein 85A	CCDC85A			
Q96LX7	Coiled-coil domain-containing protein 17	CCDC17			
Q9UI42	Carboxypeptidase A4	CPA4			
P14209	CD99 antigen	CD99			
Q9UPW5	Cytosolic carboxypeptidase 1	AGTPBP1			
P24864	G1/S-specific cyclin-E1	CCNE1			
Q8IXQ3	Uncharacterized protein C9orf40	C9orf40			
Q9BUK0	Coiled-coil-helix-coiled-coil-helix domain-containing protein 7	CHCHD7			
Q96Q40	Cyclin-dependent kinase 15	CDK15			
P46092	C-C chemokine receptor type 10	CCR10			
O95067	G2/mitotic-specific cyclin-B2	CCNB2			
Q9BYE9	Cadherin-related family member	CDHR2			
P51959	Cyclin-G1	CCNG1			
Q03188	Centromere protein C	CENPC			
Q7Z4R8	UPF0669 protein C6orf120	C6orf120			
Q02246	Contactin-2	CNTN2			
P50613	Cyclin-dependent kinase 7	CDK7			
P34972	Cannabinoid receptor 2	CNR2			
Q8WUR7	UPF0235 protein C15orf40	C15orf40			
P06681	Complement C2	C2			
Q8N8G6	Putative uncharacterized protein C15orf54	C15orf54			
P42695	Condensin-2 complex subunit D3	NCAPD3			
P13671	Complement component C6	C6			
Q5SY13	Putative uncharacterized protein encoded by COL5A1-AS1	COL5A1-AS1			
O75175	CCR4-NOT transcription complex subunit 3	CNOT3			
Q8IUQ0	Clavesin-1	CLVS1			
O95406	Protein cornichon homolog 1	CNIH1			
Q9HAZ1	Dual specificity protein kinase	CLK4			
Q9H078	Caseinolytic peptidase B protein homolog	CLPB			
Q9UHD4	Cell death activator CIDE-B	CIDEB			
P50416	Carnitine O-palmitoyltransferase 1, liver isoform	CPT1A			
P61803	Dolichyl-diphosphooligosaccharide--protein glycosyltransferase subunit DAD1	DAD1			
P26196	Probable ATP-dependent RNA helicase DDX6	DDX6			
Q9Y2R4	Probable ATP-dependent RNA helicase DDX52	DDX52			
Q96GG9	DCN1-like protein 1	DCUN1D1			

Q8TEB1	DDB1- and CUL4-associated factor 11	DCAF11			
Q4QY38	Beta-defensin 134	DEFB134			
Q8N688	Beta-defensin 123	DEFB123			
Q16832	Discoidin domain-containing receptor 2	DDR2			
P28221	5-hydroxytryptamine receptor 1D	HTR1D			
Q7Z5R6	Amyloid beta A4 precursor protein-binding family B member 1-interacting protein	APBB1IP			
Q8N8R7	ARL14 effector protein	ARL14EP			
Q8NER5	Activin receptor type-1C	ACVR1C			
Q53H12	Acylglycerol kinase, mitochondrial	AGK			
P42025	Beta-centractin	ACTR1B			
O60218	Aldo-keto reductase family 1 member B10	AKR1B10			
Q13443	Disintegrin and metalloproteinase domain-containing protein 9	ADAM9			
Q9H4A4	Aminopeptidase B	RNPEP			
Q9NRN7	L-aminoadipate-semialdehyde dehydrogenase-phosphopantetheinyl transferase	AASDHPPT			
Q9NZK5	Adenosine deaminase 2	ADA2			
Q9NUS5	AP-5 complex subunit sigma-1	AP5S1			
Q9NW15	Anoctamin-10	ANO10			
O15084	Serine/threonine-protein phosphatase 6 regulatory ankyrin repeat subunit A	ANKRD28			
P07355	Annexin A2	ANXA2			
Q9P2R3	Rabankyrin-5	ANKFY1			
P02652	Apolipoprotein A-II	APOA2			
Q96Q27	Ankyrin repeat and SOCS box protein 2	ASB2			
Q96DX5	Ankyrin repeat and SOCS box protein 9	ASB9			
Q9NR48	Histone-lysine N-methyltransferase ASH1L	ASH1L			
Q96EG1	Arylsulfatase G	ARSG			
Q9Y4P1	Cysteine protease ATG4B	ATG4B			
O95671	Probable bifunctional dTTP/UTP pyrophosphatase/methyltransferase protein	ASMTL			
Q5VTU8	ATP synthase subunit epsilon-like protein, mitochondrial	ATP5F1EP2			
Q8NCR9	Clarin-3	CLRN3			
P48047	ATP synthase subunit O, mitochondrial	ATP5PO			
Q8N5M1	ATP synthase mitochondrial F1 complex assembly factor 2	ATPAF2			
Q9NR09	Baculoviral IAP repeat-containing protein 6	BIRC6			
Q7Z6A9	B- and T-lymphocyte attenuator	BTLA			
O43709	Probable 18S rRNA	BUD23			
Q8N1D0	Beckwith-Wiedemann syndrome chromosomal region 1 candidate gene B protein	SLC22A18AS			
Q9UL45	Biogenesis of lysosome-related organelles complex 1 subunit 6	BLOC1S6			
O15382	Branched-chain-amino-acid aminotransferase, mitochondrial	BCAT2			
P23560	Brain-derived neurotrophic factor	BDNF			
P11274	Breakpoint cluster region protein	BCR			
Q8N5N4	Uncharacterized protein C3orf22	C3orf22			
Q96HB5	Coiled-coil domain-containing protein 120	CCDC120			
Q6PII3	Coiled-coil domain-containing protein 174	CCDC174			
Q9BQI4	Coiled-coil domain-containing protein 3	CCDC3			

Q8IYR0	Cilia- and flagella-associated protein 206	CFAP206			
Q86UF4	Coiled-coil domain-containing protein 190	CCDC190			
Q5TID7	Coiled-coil domain-containing protein 181	CCDC181			
Q9GZT6	Coiled-coil domain-containing protein 90B, mitochondrial	CCDC90B			
Q9Y3M2	Protein chibby homolog 1	CBY1			
Q8IYW2	Cilia- and flagella-associated protein 46	CFAP46			
Q8WUX9	Charged multivesicular body protein 7	CHMP7			
Q96G28	Cilia- and flagella-associated protein 36	CFAP36			
Q8NHZ8	Anaphase-promoting complex subunit CDC26	CDC26			
P00751	Complement factor B	CFB			
P10747	T-cell-specific surface glycoprotein CD28	CD28			
Q8N9R6	CMT1A duplicated region transcript 4 protein	CDRT4			
P13569	Cystic fibrosis transmembrane conductance regulator	CFTR			
Q0VF96	Cingulin-like protein 1	CGNL1			
O43866	CD5 antigen-like	CD5L			
Q8WYQ3	Coiled-coil-helix-coiled-coil-helix domain-containing protein 10, mitochondrial	CHCHD10			
Q96KT6	Putative uncharacterized protein encoded by LINC00208	LINC00208			
Q16878	Cysteine dioxygenase type 1	CDO1			
Q5MAI5	Cyclin-dependent kinase-like 4	CDKL4			
Q9UQ88	Cyclin-dependent kinase 11A	CDK11A			
Q9Y6K0	Choline/ethanolaminephosphotransferase 1	CEPT1			
E5RJ46	Uncharacterized protein C8orf87	C8orf87			
P0DPI3	Centromere protein V-like protein 2	CENPVL2			
Q9BS16	Centromere protein K	CENPK			
Q6P2H3	Centrosomal protein of 85 kDa	CEP85			
Q9H8S5	Cyclin-P	CCNP			
Q14004	Cyclin-dependent kinase 13	CDK13			
Q00535	Cyclin-dependent-like kinase 5	CDK5			
Q7L2Z9	Centromere protein Q	CENPQ			
Q9UQ52	Contactin-6	CNTN6			
Q9UHC6	Contactin-associated protein-like 2	CNTNAP2			
Q6UXS0	C-type lectin domain family 19 member A	CLEC19A			
P24941	Cyclin-dependent kinase 2	CDK2			
P25940	Collagen alpha-3	COL5A3			
O75128	Protein cordon-bleu	COBL			
P11509	Cytochrome P450 2A6	CYP2A6			
Q96MU5	Uncharacterized protein C17orf77	C17orf77			
Q9HCH3	Copine-5	CPNE5			
Q6FI81	Anamorsin	CIAPIN1			
Q14093	Cylicin-2	CYLC2			
Q99418	Cytohesin-2	CYTH2			
Q86XQ3	Cation channel sperm-associated protein 3	CATSPER3			
Q69YQ0	Cytospin-A	SPECC1L			
Q9UI36	Dachshund homolog 1	DACH1			
O15523	ATP-dependent RNA helicase DDX3Y	DDX3Y			
Q6ZNG2	Homeobox protein DBX2	DBX2			
Q8WVS4	Cytoplasmic dynein 2 intermediate chain 1	DYNC2I1			
Q15038	DAZ-associated protein 2	DAZAP2			

Q9BYW3	Beta-defensin 126	DEFB126			
O75907	Diacylglycerol O-acyltransferase 1	DGAT1			
O60231	Pre-mRNA-splicing factor ATP-dependent RNA helicase DHX16	DHX16			
Q7L7V1	Putative pre-mRNA-splicing factor ATP-dependent RNA helicase DHX32	DHX32			
Q7L5Y6	DET1 homolog	DET1			
Q8WWF6	DnaJ homolog subfamily B member 3	DNAJB3			
Q5JSL3	Dedicator of cytokinesis protein	DOCK11			
Q15057	Arf-GAP with coiled-coil, ANK repeat and PH domain-containing protein 2	ACAP2			
Q9ULW3	Activator of basal transcription 1	ABT1			
P04217	Alpha-1B-glycoprotein	A1BG			
Q9UKV3	Apoptotic chromatin condensation inducer in the nucleus	ACIN1			
P49189	4-trimethylaminobutyraldehyde dehydrogenase	ALDH9A1			
Q9NQ94	APOBEC1 complementation factor	A1CF			
P00505	Aspartate aminotransferase, mitochondrial	GOT2			
P61981	14-3-3 protein gamma	YWHAG			
P0DMS8	Adenosine receptor A3	ADORA3			
P22760	Arylacetamide deacetylase	AADAC			
O95433	Activator of 90 kDa heat shock protein ATPase homolog 1	AHSA1			
P20292	Arachidonate 5-lipoxygenase-activating protein	ALOX5AP			
Q5VUJ5	Putative Arf-GAP with GTPase, ANK repeat and PH domain-containing protein 7	AGAP7P			
Q9H161	Homeobox protein aristaless-like 4	ALX4			
Q5JQC9	A-kinase anchor protein 4	AKAP4			
Q9H2U9	Disintegrin and metalloproteinase domain-containing protein 7	ADAM7			
Q96CM8	Medium-chain acyl-CoA ligase ACSF2, mitochondrial	ACSF2			
P40145	Adenylate cyclase type 8	ADCY8			
Q5T601	Adhesion G-protein coupled receptor F1	ADGRF1			
P22570	NADPH:adrenodoxin oxidoreductase, mitochondrial	FDXR			
O00468	Agrin	AGRN			
P12235	ADP/ATP translocase 1	SLC25A4			
Q9UM13	Anaphase-promoting complex subunit 10	ANAPC10			
Q9H560	Putative ankyrin repeat domain-containing protein 19	ANKRD19P			
O95236	Apolipoprotein L3	APOL3			
Q8WW43	Gamma-secretase subunit APH-1B	APH1B			
O96018	Amyloid-beta A4 precursor protein-binding family A member 3	APBA3			
Q9HDC9	Adipocyte plasma membrane-associated protein	APMAP			
Q92888	Rho guanine nucleotide exchange factor 1	ARHGEF1			
Q8N6M6	Aminopeptidase O	AOPEP			
Q96NS5	Ankyrin repeat and SOCS box protein 16	ASB16			
P98196	Probable phospholipid-transporting ATPase IH	ATP11A			
Q96B67	Arrestin domain-containing protein 3	ARRDC3			

P15289	Arylsulfatase A	ARSA			
Q9HBK9	Arsenite methyltransferase	AS3MT			
Q13705	Activin receptor type-2B	ACVR2B			
O75061	Putative tyrosine-protein phosphatase auxilin	DNAJC6			
O95450	A disintegrin and metalloproteinase with thrombospondin motifs 2	ADAMTS2			
P18859	ATP synthase-coupling factor 6, mitochondrial	ATP5PF			
Q6DD88	Atlastin-3	ATL3			
Q8TDN6	Ribosome biogenesis protein BRX1 homolog	BRIX1			
Q6UXE8	Butyrophilin-like protein 3	BTNL3			
Q9Y2F9	BTB/POZ domain-containing protein 3	BTBD3			
Q5W0U4	B box and SPRY domain-containing protein	BSPRY			
Q9BPU9	B9 domain-containing protein 2	B9D2			
Q76KP1	N-acetyl-beta-glucosaminyl-glycoprotein 4-beta-N-acetylgalactosaminyltransferase 1	B4GALNT4			
Q6ZMB0	Acetylgalactosaminyl-O-glycosyl-glycoprotein beta-1,3-N-acetylglucosaminyltransferase	B3GNT6			
Q5T5X7	BEN domain-containing protein 3	BEND3			
Q92843	Bcl-2-like protein 2	BCL2L2			
Q9NY43	BarH-like 2 homeobox protein	BARHL2			
Q99728	BRCA1-associated RING domain protein 1	BARD1			
Q5H9F3	BCL-6 corepressor-like protein 1	BCORL1			
P23280	Carbonic anhydrase 6	CA6			
Q7L4P6	BEN domain-containing protein 5	BEND5			
Q8N9H9	Uncharacterized protein C1orf127	C1orf127			
Q9BXH1	Bcl-2-binding component 3, isoforms 1/2	BBC3			
Q5T7R7	Uncharacterized protein C1orf185	C1orf185			
Q9BXJ4	Complement C1q tumor necrosis factor-related protein 3	C1QTNF3			
Q69YN2	CWF19-like protein 1	CWF19L1			
Q9NYX4	Neuron-specific vesicular protein calcyon	CALY			
Q8N4S0	Coiled-coil domain-containing protein 82	CCDC82			
Q8IX12	Cell division cycle and apoptosis regulator protein 1	CCAR1			
Q9Y646	Carboxypeptidase Q	CPQ			
Q99440	Uncharacterized protein encoded by LINC01587	LINC01587			
Q8TDI0	Chromodomain-helicase-DNA-binding protein 5	CHD5			
P42771	Cyclin-dependent kinase inhibitor 2A	CDKN2A			
O94921	Cyclin-dependent kinase 14	CDK14			
Q8NE62	Choline dehydrogenase, mitochondrial	CHDH			
O15182	Centrin-3	CETN3			
A6NGG3	Putative uncharacterized protein C9orf92	C9orf92			
Q6IPU0	Centromere protein P	CENPP			
Q8TBE1	Protein cornichon homolog 3	CNIH3			
Q9C0A0	Contactin-associated protein-like 4	CNTNAP4			
Q96M02		C10orf90			
Q86WW8	Cytochrome c oxidase assembly factor 5	COA5			
Q96KA5	Cleft lip and palate transmembrane protein 1-like protein	CLPTM1L			

Q96RK0	Protein capicua homolog	CIC			
Q8NHS4	Clathrin heavy chain linker domain-containing protein 1	CLHC1			
P24387	Corticotropin-releasing factor-binding protein	CRHBP			
P0DML3	Chorionic somatomammotropin hormone 2	CSH2			
Q6NV74	CRACD-like protein	CRACDL			
A6NMK7	Putative cleavage and polyadenylation specificity factor subunit 4-like protein	CPSF4L			
Q6UXH1	Protein disulfide isomerase CRELD2	CRELD2			
Q7Z4B0	Putative uncharacterized protein encoded by LINC00305	LINC00305			
Q99807	5-demethoxyubiquinone hydroxylase, mitochondrial	COQ7			
Q93034	Cullin-5	CUL5			
Q9ULE3	DENN domain-containing protein 2A	DENND2A			
Q5D0E6	DALR anticodon-binding domain-containing protein 3	DALRD3			
P06132	Uroporphyrinogen decarboxylase	UROD			
Q9NR30	Nucleolar RNA helicase 2	DDX21			
Q9NQC7	Ubiquitin carboxyl-terminal hydrolase CYLD	CYLD			
Q5TAQ9	DDB1- and CUL4-associated factor 8	DCAF8			
Q96IU4	Protein ABHD14B	ABHD14B			
Q96HD1	Protein disulfide isomerase CRELD1	CRELD1			
Q6PUV4	Complexin-2	CPLX2			
A8MQ03	Cysteine-rich tail protein 1	CYSRT1			
Q5DJT8	Cancer/testis antigen family 45 member A2	CT45A2			
P00167	Cytochrome b5	CYB5A			
A0A1B0GTU2	Cortexin domain-containing 1	CTXND1			
Q9HC47	Cutaneous T-cell lymphoma-associated antigen 1	CTAGE1			
O95452	Gap junction beta-6 protein	GJB6			
O00574	C-X-C chemokine receptor type 6	CXCR6			
A0A096LNP1	Beta-defensin 131B	DEFB131B			
P0DP74	Beta-defensin 130A	DEFB130A			
O96002	Putative transmembrane protein CXorf1	CXorf1			
Q6UXB2	C-X-C motif chemokine 17	CXCL17			
Q8N1L1	Putative uncharacterized protein encoded by LINC00528	LINC00528			
P01034	Cystatin-C	CST3			
Q6IC83	Uncharacterized protein C22orf42	C22orf42			
Q86T65	Disheveled-associated activator of morphogenesis 2	DAAM2			
Q68D51	DENN domain-containing protein 2C	DENND2C			
P32321	Deoxycytidylate deaminase	DCTD			
Q96GQ7	Probable ATP-dependent RNA helicase DDX27	DDX27			
Q5TDH0	Protein DDI1 homolog 2	DDI2			
Q9Y315	Deoxyribose-phosphate aldolase	DERA			
Q9UBU7	Protein DBF4 homolog A	DBF4			
Q05D60	Deuterosome assembly protein 1	DEUP1			
Q8TDJ6	DmX-like protein 2	DMXL2			
Q14574	Desmocollin-3	DSC3			
Q8WWB3	DPY30 domain-containing protein 1	DYDC1			
Q9UNE0	Tumor necrosis factor receptor superfamily member EDAR	EDAR			

Q9BV94	ER degradation-enhancing alpha-mannosidase-like protein 2	EDEM2			
Q3B7T1	Erythroid differentiation-related factor 1	EDRF1			
A6NNS2	Dehydrogenase/reductase SDR family member 7C	DHRS7C			
Q9BQI3	Eukaryotic translation initiation factor 2-alpha kinase 1	EIF2AK1			
Q86V85	Integral membrane protein GPR180	GPR180			
P26641	Elongation factor 1-gamma	EEF1G			
O43402	ER membrane protein complex subunit 8	EMC8			
P26378	ELAV-like protein 4	ELAVL4			
Q9BVM4	Gamma-glutamylaminecyclotransferase	GGACT			
Q6UXU4	Germ cell-specific gene 1-like protein	GSG1L			
O95267	RAS guanyl-releasing protein 1	RASGRP1			
Q5TEC6	Histone HIST2H3PS2	H3-2			
Q5TGJ6	Hepatoma-derived growth factor-like protein 1	HDGFL1			
P02008	Hemoglobin subunit zeta	HBZ			
Q9BYQ4	Keratin-associated protein 9-2	KRTAP9-2			
A2PYH4	Probable ATP-dependent DNA helicase HFM1	HFM1			
Q5VTY9	Protein-cysteine N-palmitoyltransferase HHAT	HHAT			
P31260	Homeobox protein Hox-A10	HOXA10			
Q9BVR0	Putative HERC2-like protein 3	HERC2P3			
P11142	Heat shock cognate 71 kDa protein	HSPA8			
A0A0C4DH38	Immunoglobulin heavy variable 5-51	IGHV5-51			
A0A0B4J1U7	Immunoglobulin heavy variable 6-1	IGHV6-1			
A0A0J9YVY3	Immunoglobulin heavy variable 7-4-1	IGHV7-4-1			
A0A0B4J1V7	Probable non-functional immunoglobulin heavy variable 7-81	IGHV7-81			
Q6MZM0	Ferroxidase HEPHL1	HEPHL1			
P32754	4-hydroxyphenylpyruvate dioxygenase	HPD			
P17693	HLA class I histocompatibility antigen, alpha chain G	HLA-G			
P01893	Putative HLA class I histocompatibility antigen, alpha chain H	HLA-H			
P31942	Heterogeneous nuclear ribonucleoprotein H3	HNRNPH3			
O14732	Inositol monophosphatase 2	IMPA2			
P01583	Interleukin-1 alpha	IL1A			
Q9UF12	Hydroxyproline dehydrogenase	PRODH2			
O60684	Importin subunit alpha-7	KPNA6			
P01834	Immunoglobulin kappa constant	IGKC			
Q6UXB1	Insulin growth factor-like family member 3	IGFL3			
Q6GPH6	Inositol 1,4,5-trisphosphate receptor-interacting protein-like 1	ITPRIPL1			
Q86U28	Iron-sulfur cluster assembly 2 homolog, mitochondrial	ISCA2			
Q6UXX5	Inter-alpha-trypsin inhibitor heavy chain H6	ITIH6			
Q3SY84	Keratin, type II cytoskeletal 71	KRT71			
Q9C010	cAMP-dependent protein kinase inhibitor beta	PKIB			
Q9ULG1	Chromatin-remodeling ATPase INO80	INO80			

Q6UWL6	Kin of IRRE-like protein 2	KIRREL2			
P78412	Iroquois-class homeodomain protein IRX-6	IRX6			
Q3LI77	Keratin-associated protein 13-4	KRTAP13-4			
P16389	Potassium voltage-gated channel subfamily A member 2	KCNA2			
A0A0B4J2D9	Immunoglobulin kappa variable 1D-13	IGKV1D-13			
Q8TAP9	M-phase-specific PLK1-interacting protein	MPLKIP			
Q9BU76	Multiple myeloma tumor-associated protein 2	MMTAG2			
Q96HT8	MORF4 family-associated protein 1-like 1	MRFAP1L1			
Q9H845	Complex I assembly factor ACAD9, mitochondrial	ACAD9			
Q9Y243	RAC-gamma serine/threonine-protein kinase	AKT3			
Q9BR61	Acyl-CoA-binding domain-containing protein 6	ACBD6			
P45844	ATP-binding cassette sub-family G member 1	ABCG1			
Q8IZP0	Abl interactor 1	ABI1			
P01031	Complement C5	C5			
Q496F6	CMRF35-like molecule 2	CD300E			
Q6UXZ3	CMRF35-like molecule 5	CD300LD			
P49760	Dual specificity protein kinase	CLK2			
Q05639	Elongation factor 1-alpha 2	EEF1A2			
Q4G0I0	Protein CCSMST1	CCSMST1			
Q16610	Extracellular matrix protein 1	ECM1			
Q15125	3-beta-hydroxysteroid-Delta	EBP			
P07320	Gamma-crystallin D	CRYGD			
Q9BU20	Ciliogenesis and planar polarity effector 2	CPLANE2			
P33261	Cytochrome P450 2C19	CYP2C19			
Q9H7D0	Dedicator of cytokinesis protein 5	DOCK5			
O00548	Delta-like protein 1	DLL1			
P78352	Disks large homolog 4	DLG4			
Q92988	Homeobox protein DLX-4	DLX4			
Q7L8W6	Diphthine--ammonia ligase	DPH6			
P27487	Dipeptidyl peptidase 4	DPP4			
Q9NXC2	Glucose-fructose oxidoreductase domain-containing protein 1	GFOD1			
P16260	Graves disease carrier protein	SLC25A16			
O94808	Glutamine--fructose-6-phosphate aminotransferase	GFPT2			
P00390	Glutathione reductase, mitochondrial	GSR			
Q6B0K9	Hemoglobin subunit mu	HBM			
Q8IV61	Ras guanyl-releasing protein 3	RASGRP3			
Q9BYG8	Gasdermin-C	GSDMC			
Q5QNW6	Histone H2B type 2-F	H2BC18			
Q9H4S2	GS homeobox 1	GSX1			
A0A096LPI5	Putative uncharacterized protein CCDC28A-AS1	CCDC28A-AS1			
Q9ULK0	Glutamate receptor ionotropic, delta-1	GRID1			
Q96GX5	Serine/threonine-protein kinase greatwall	MASTL			
Q9BYE0	Transcription factor HES-7	HES7			
Q96N76	Urocanate hydratase	UROC1			
P00492	Hypoxanthine-guanine phosphoribosyltransferase	HPRT1			
Q8N3J3	Homologous recombination OB-fold protein	HROB			
P17096	High mobility group protein HMG-I/HMG-Y	HMGA1			
P05204	Non-histone chromosomal protein HMG-17	HMGN2			

P55795	Heterogeneous nuclear ribonucleoprotein H2	HNRNPH2			
P15248	Interleukin-9	IL9			
P05014	Interferon alpha-4	IFNA4			
Q9HBE5	Interleukin-21 receptor	IL21R			
P41091	Eukaryotic translation initiation factor 2 subunit 3	EIF2S3			
P78344	Eukaryotic translation initiation factor 4 gamma 2	EIF4G2			
Q96A47	Insulin gene enhancer protein ISL-2	ISL2			
P08729	Keratin, type II cytoskeletal 7	KRT7			
Q9BX67	Junctional adhesion molecule C	JAM3			
Q15811	Intersectin-1	ITSN1			
Q0D2K2	Kelch-like protein 30	KLHL30			
Q9NPC2	Potassium channel subfamily K member 9	KCNK9			
Q96QS6	Serine/threonine-protein kinase H2	PSKH2			
Q6YP21	Kynurenine--oxoglutarate transaminase 3	KYAT3			
Q13554	Calcium/calmodulin-dependent protein kinase type II subunit beta	CAMK2B			
O43766	Lipoyl synthase, mitochondrial	LIAS			
Q6UWM7	Lactase-like protein	LCTL			
P47929	Galectin-7	LGALS7			
O15230	Laminin subunit alpha-5	LAMA5			
Q6PKG0	La-related protein 1	LARP1			
Q6JVE9	Epididymal-specific lipocalin-8	LCN8			
Q96CN5	Leucine-rich repeat-containing protein 45	LRRC45			
Q86YD5	Low-density lipoprotein receptor class A domain-containing protein 3	LDLRAD3			
Q9H756	Leucine-rich repeat-containing protein 19	LRRC19			
Q9UN81	LINE-1 retrotransposable element ORF1 protein	L1RE1			
Q53EV4	Leucine-rich repeat-containing protein 23	LRRC23			
Q8TDW0	Volume-regulated anion channel subunit LRRC8C	LRRC8C			
Q96GA3	Protein LTV1 homolog	LTV1			
Q05469	Hormone-sensitive lipase	LIPE			
A0A075B6I4	Immunoglobulin lambda variable 10-54	IGLV10-54			
P42704	Leucine-rich PPR motif-containing protein, mitochondrial	LRPPRC			
Q8N448	Ligand of Numb protein X 2	LNX2			
Q5SRR4	Lymphocyte antigen 6 complex locus protein G5c	LY6G5C			
Q0VAK6	Leiomodin-3	LMOD3			
P01703	Immunoglobulin lambda variable 1-40	IGLV1-40			
A0A075B6I6	Probable non-functional immunoglobulin lambda variable 1-50	IGLV1-50			
Q9NX58	Cell growth-regulating nucleolar protein	LYAR			
A8MSI8	LYR motif-containing protein 9	LYRM9			
P01704	Immunoglobulin lambda variable 2-14	IGLV2-14			
Q5VYS4	Mesenteric estrogen-dependent adipogenesis protein	MEDAG			
A0A075B6K5	Immunoglobulin lambda variable 3-9	IGLV3-9			
Q9ULC4	Malignant T-cell-amplified sequence 1	MCTS1			
P43243	Matrin-3	MATR3			

O15553	Pyrin	MEFV			
Q8NEM0	Microcephalin	MCPH1			
O43283	Mitogen-activated protein kinase kinase kinase 13	MAP3K13			
O75448	Mediator of RNA polymerase II transcription subunit 24	MED24			
Q8N4V1	ER membrane protein complex subunit 5	MMGT1			
P32519	ETS-related transcription factor Elf-1	ELF1			
Q6UWW8	Carboxylesterase 3	CES3			
Q9H6S3	Epidermal growth factor receptor kinase substrate 8-like protein 2	EPS8L2			
P0C866	Putative uncharacterized protein encoded by LINC00869	LINC00869			
P00742	Coagulation factor X	F10			
P03951	Coagulation factor XI	F11			
P58658	Protein eva-1 homolog C	EVA1C			
Q76MJ5	Serine/threonine-protein kinase/endoribonuclease IRE2	ERN2			
O60447	Ecotropic viral integration site 5 protein homolog	EVI5			
Q8N612	FTS and Hook-interacting protein	FAM160A2			
Q13630	GDP-L-fucose synthase	GFUS			
P12314	High affinity immunoglobulin gamma Fc receptor I	FCGR1A			
P31995	Low affinity immunoglobulin gamma Fc region receptor II-c	FCGR2C			
Q96RD9	Fc receptor-like protein 5	FCRL5			
Q5VTL7	Fibronectin type III domain-containing protein 7	FNDC7			
Q13451	Peptidyl-prolyl cis-trans isomerase FKBP5	FKBP5			
Q4G163	F-box only protein 43	FBXO43			
Q9BYJ0	Fibroblast growth factor-binding protein 2	FGFBP2			
Q8N3Y1	F-box/WD repeat-containing protein 8	FBXW8			
Q8TAT2	Fibroblast growth factor-binding protein 3	FGFBP3			
P98174	FYVE, RhoGEF and PH domain-containing protein 1	FGD1			
Q13643	Four and a half LIM domains protein 3	FHL3			
Q495W5	Alpha-	FUT11			
A0AVI2	Fer-1-like protein 5	FER1L5			
Q9HDB9	Endogenous retrovirus group K member 5 Gag polyprotein	ERVK-5			
Q96ME1	F-box/LRR-repeat protein 18	FBXL18			
Q9BT04	Protein fuzzy homolog	FUZ			
Q6ZQN5	Forkhead box protein I2	FOXI2			
P58012	Forkhead box protein L2	FOXL2			
Q13069	G antigen 5	GAGE5			
Q9BXM9	FSD1-like protein	FSD1L			
A6NEY3	Putative golgin subfamily A member 6-like protein 3	GOLGA6L3			
Q8WTR4	Glycerophosphodiester phosphodiesterase domain-containing protein 5	GDPD5			
Q8IW92	Beta-galactosidase-1-like protein 2	GLB1L2			
P01215	Glycoprotein hormones alpha chain	CGA			
Q9UK08	Guanine nucleotide-binding protein G	GNG8			
P29992	Guanine nucleotide-binding protein subunit alpha-11	GNA11			
P43220	Glucagon-like peptide 1 receptor	GLP1R			
Q9NS18	Glutaredoxin-2, mitochondrial	GLRX2			

Q4ZG55	Protein GREB1	GREB1			
P14314	Glucosidase 2 subunit beta	PRKCSH			
Q15760	Probable G-protein coupled receptor 19	GPR19			
A4D1B5	Gamma-secretase-activating protein	GSAP			
Q03013	Glutathione S-transferase Mu 4	GSTM4			
P38646	Stress-70 protein, mitochondrial	HSPA9			
Q9P2G9	Kelch-like protein 8	KLHL8			
Q5TC63	Growth hormone-regulated TBC protein 1	GRTP1			
Q9UGQ3	Solute carrier family 2, facilitated glucose transporter member 6	SLC2A6			
Q9BYR0	Keratin-associated protein 4-7	KRTAP4-7			
Q9BYQ8	Keratin-associated protein 4-9	KRTAP4-9			
Q86T24	Transcriptional regulator Kaiso	ZBTB33			
Q6L8H4	Keratin-associated protein 5-1	KRTAP5-1			
O75367	Core histone macro-H2A.1	MACROH2A1			
Q16777	Histone H2A type 2-C	H2AC20			
O43424	Glutamate receptor ionotropic, delta-2	GRID2			
P57053	Histone H2B type F-S	H2BS1			
A1L0T0	2-hydroxyacyl-CoA lyase 2	ILVBL			
Q68CP4	Heparan-alpha-glucosaminide N-acetyltransferase	HGSNAT			
Q9ULI3	Protein HEG homolog 1	HEG1			
P54840	Glycogen	GYS2			
A0A0C4DH35	Probable non-functional immunoglobulin heavy variable 3-35	IGHV3-35			
O43390	Heterogeneous nuclear ribonucleoprotein R	HNRNPR			
B7ZW38	Heterogeneous nuclear ribonucleoprotein C-like 3	HNRNPCL3			
Q9UBC0	Hepatocyte nuclear factor 6	ONECUT1			
O15347	High mobility group protein B3	HMGB3			
Q9HBE4	Interleukin-21	IL21			
Q9UKT9	Zinc finger protein Aiolos	IKZF3			
Q8WWZ1	Interleukin-1 family member 10	IL1F10			
Q6ZVL6	UPF0606 protein KIAA1549L	KIAA1549L			
Q9UK76	Jupiter microtubule associated homolog 1	JPT1			
P51617	Interleukin-1 receptor-associated kinase 1	IRAK1			
O60575	Serine protease inhibitor Kazal-type 4	SPINK4			
Q6ZMV9	Kinesin-like protein KIF6	KIF6			
P43631	Killer cell immunoglobulin-like receptor 2DS2	KIR2DS2			
Q6UVM3	Potassium channel subfamily T member 2	KCNT2			
Q92876	Kallikrein-6	KLK6			
Q6PIL6	Kv channel-interacting protein 4	KCNIP4			
P10644	cAMP-dependent protein kinase type I-alpha regulatory subunit	PRKAR1A			
Q3LI54	Keratin-associated protein 19-8	KRTAP19-8			
Q92794	Histone acetyltransferase KAT6A	KAT6A			
Q701N2	Keratin-associated protein 5-5	KRTAP5-5			
Q9H9L4	KAT8 regulatory NSL complex subunit 2	KANSL2			
Q63ZY3	KN motif and ankyrin repeat domain-containing protein 2	KANK2			
Q8IUC3	Keratin-associated protein 7-1	KRTAP7-1			
P83111	Serine beta-lactamase-like protein LACTB, mitochondrial	LACTB			
A0A0C4DH73	Immunoglobulin kappa variable 1-12	IGKV1-12			
P61326	Protein mago nashi homolog	MAGOH			

Q8N468	Major facilitator superfamily domain-containing protein 4A	MFSD4A			
Q6WCQ1	Myosin phosphatase Rho-interacting protein	MPRIP			
P11712	Cytochrome P450 2C9	CYP2C9			
P20813	Cytochrome P450 2B6	CYP2B6			
P13611	Versican core protein	VCAN			
P17927	Complement receptor type 1	CR1			
P40313	Chymotrypsin-like protease CTRL-1	CTRL			
P61073	C-X-C chemokine receptor type 4	CXCR4			
P02778	C-X-C motif chemokine 10	CXCL10			
Q6NUT2	Probable C-mannosyltransferase DPY19L2	DPY19L2			
P35659	Protein DEK	DEK			
O94760	N	DDAH1			
Q8NEL9	Phospholipase DDHD1	DDHD1			
Q5VU92	DDB1- and CUL4-associated factor 12-like protein 1	DCAF12L1			
Q6NSW5	Putative DENN domain-containing protein 10 B	DENND10P1			
P17844	Probable ATP-dependent RNA helicase DDX5	DDX5			
Q9P2X7	Deleted in esophageal cancer 1	DELEC1			
P81605	Dermcidin	DCD			
Q96LJ7	Dehydrogenase/reductase SDR family member 1	DHRS1			
Q9UGM3	Deleted in malignant brain tumors 1 protein	DMBT1			
Q8WXX0	Dynein heavy chain 7, axonemal	DNAH7			
Q6ZMT9	Death domain-containing protein 1	DTHD1			
Q6P2E9	Enhancer of mRNA-decapping protein 4	EDC4			
O75530	Polycomb protein EED	EED			
P11171	Protein 4.1	EPB41			
Q9N2K0	HERV-H_2q24.3 provirus ancestral Env polyprotein				
Q902F8	Endogenous retrovirus group K member 8 Env polyprotein	ERVK-8			
Q9NX77	Endogenous retrovirus group K member 13-1 Env polyprotein	ERVK13-1			
Q13144	Translation initiation factor eIF-2B subunit epsilon	EIF2B5			
O95936	Eomesodermin homolog	EOMES			
Q8TE68	Epidermal growth factor receptor kinase substrate 8-like protein 1	EPS8L1			
Q9BXW9	Fanconi anemia group D2 protein	FANCD2			
Q92731	Estrogen receptor beta	ESR2			
O75844	CAAX prenyl protease 1 homolog	ZMPSTE24			
Q9Y421	Protein FAM32A	FAM32A			
Q8TAV0	Protein FAM76A	FAM76A			
Q2M2I3	Protein FAM83E	FAM83E			
F2Z333	Fibronectin type III domain-containing protein 10	FNDC10			
O75636	Ficolin-3	FCN3			
Q4ZHG4	Fibronectin type III domain-containing protein 1	FNDC1			
Q02790	Peptidyl-prolyl cis-trans isomerase FKBP4	FKBP4			
Q9Y5Y0	Feline leukemia virus subgroup C receptor-related protein 1	FLVCR1			
P31371	Fibroblast growth factor 9	FGF9			
P21781	Fibroblast growth factor 7	FGF7			
Q5D862	Filaggrin-2	FLG2			
P08620	Fibroblast growth factor 4	FGF4			
Q92496	Complement factor H-related protein 4	CFHR4			
Q5TGI0	Failed axon connections homolog	FAXC			

P62684	Endogenous retrovirus group K member 113 Gag polyprotein	HERVK_113			
Q96NE9	FERM domain-containing protein 6	FRMD6			
Q92945	Far upstream element-binding protein 2	KHSRP			
Q14697	Neutral alpha-glucosidase AB	GANAB			
Q9UJY5	ADP-ribosylation factor-binding protein GGA1	GGA1			
A6NI86	Golgin subfamily A member 6-like protein 10	GOLGA6L10			
O75603	Chorion-specific transcription factor GCMb	GCM2			
A6NCL1	Geminin coiled-coil domain-containing protein 1	GMNC			
Q8TEQ6	Gem-associated protein 5	GEMIN5			
P50151	Guanine nucleotide-binding protein G	GNG10			
Q96CW5	Gamma-tubulin complex component 3	TUBGCP3			
Q9H227	Cytosolic beta-glucosidase	GBA3			
Q9NS67	Probable G-protein coupled receptor 27	GPR27			
Q14330	N-arachidonyl glycine receptor	GPR18			
O00270	-12	GPR31			
O95259	Potassium voltage-gated channel subfamily H member 1	KCNH1			
Q8IYK4	Procollagen galactosyltransferase 2	COLGALT2			
P60893	Probable G-protein coupled receptor 85	GPR85			
Q96P67	Probable G-protein coupled receptor 82	GPR82			
Q9UL51	Potassium/sodium hyperpolarization-activated cyclic nucleotide-gated channel 2	HCN2			
P48506	Glutamate--cysteine ligase catalytic subunit	GCLC			
B0YJ81	Very-long-chain	HACD1			
Q96EW2	HSPB1-associated protein 1	HSPBAP1			
Q08623	Pseudouridine-5'-phosphatase	PUDP			
Q99525	Histone H4-like protein type G	H4C7			
Q53FT3	Protein Hikeshi	HIKESHI			
Q76N89	E3 ubiquitin-protein ligase	HECW1			
Q9NQG7	Hermansky-Pudlak syndrome 4 protein	HPS4			
Q9ULT8	E3 ubiquitin-protein ligase HECTD1	HECTD1			
P20719	Homeobox protein Hox-A5	HOXA5			
P00739	Haptoglobin-related protein	HPR			
A8MYZ5	IQ domain-containing protein F6	IQCF6			
Q9H0H0	Integrator complex subunit 2	INTS2			
Q16674	Melanoma-derived growth regulatory protein	MIA			
P52732	Kinesin-like protein KIF11	KIF11			
Q6P597	Kinesin light chain 3	KLC3			
Q5H943	Kita-kyushu lung cancer antigen 1	CT83			
Q3LI63	Keratin-associated protein 20-1	KRTAP20-1			
Q9NVR5	Protein kintoun	DNAAF2			
Q9NS40	Potassium voltage-gated channel subfamily H member 7	KCNH7			
O75840	Krueppel-like factor 7	KLF7			
Q9UMN6	Histone-lysine N-methyltransferase 2B	KMT2B			
P48742	LIM/homeobox protein Lhx1	LHX1			
P01615	Immunoglobulin kappa variable 2D-28	IGKV2D-28			
Q8NG48	Protein Lines homolog 1	LINS1			
Q14847	LIM and SH3 domain protein 1	LASP1			

Q9Y383	Putative RNA-binding protein Luc7-like 2	LUC7L2			
P05455	Lupus La protein	SSB			
Q9GIP4	Putative L-type amino acid transporter 1-like protein IMAA	SLC7A5P2			
Q86WA8	Lon protease homolog 2, peroxisomal	LONP2			
Q86VZ4	Low-density lipoprotein receptor-related protein 11	LRP11			
Q8WV07	Protein LTO1 homolog	LTO1			
Q96L50	Leucine-rich repeat protein 1	LRR1			
A6NK58	Putative lipoyltransferase 2, mitochondrial	LIPT2			
Q8N149	Leukocyte immunoglobulin-like receptor subfamily A member 2	LILRA2			
Q16798	NADP-dependent malic enzyme, mitochondrial	ME3			
A6BM72	Multiple epidermal growth factor-like domains protein 11	MEGF11			
A0A0B4J1Y8	Immunoglobulin lambda variable 9-49	IGLV9-49			
O15068	Guanine nucleotide exchange factor DBS	MCF2L			
P10911	Proto-oncogene DBL	MCF2			
P49137	MAP kinase-activated protein kinase 2	MAPKAPK2			
Q15555	Microtubule-associated protein RP/EB family member 2	MAPRE2			
Q969V1	Melanin-concentrating hormone receptor 2	MCHR2			
O00462	Beta-mannosidase	MANBA			
Q9UJA3	DNA helicase MCM8	MCM8			
Q9HDB8	Endogenous retrovirus group K member 5 Env polyprotein	ERVK-5			
P51610	Host cell factor 1	HCFC1			
Q6PXP3	Solute carrier family 2, facilitated glucose transporter member 7	SLC2A7			
P0CAP2	DNA-directed RNA polymerase II subunit GRINL1A	POLR2M			
Q5TGS1	Transcription factor HES-3	HES3			
Q8NE63	Homeodomain-interacting protein kinase 4	HIPK4			
Q7Z353	Highly divergent homeobox	HDX			
O43679	LIM domain-binding protein 2	LDB2			
Q86XA9	HEAT repeat-containing protein 5A	HEATR5A			
P35453	Homeobox protein Hox-D13	HOXD13			
P57058	Hormonally up-regulated neu tumor-associated kinase	HUNK			
A0A0B4J1V0	Immunoglobulin heavy variable 3-15	IGHV3-15			
P0DTE1	Probable non-functional immunoglobulin heavy variable 3-38-3	IGHV3-38-3			
Q9ULV5	Heat shock factor protein 4	HSF4			
O95757	Heat shock 70 kDa protein 4L	HSPA4L			
Q86YM7	Homer protein homolog 1	HOMER1			
Q16534	Hepatic leukemia factor	HLF			
Q6P1K1	Heme transporter HRG1	SLC48A1			
Q5SSJ5	Heterochromatin protein 1-binding protein 3	HP1BP3			
A0A0C4DH62	Immunoglobulin heavy joining 1	IGHJ1			
Q01344	Interleukin-5 receptor subunit alpha	IL5RA			
Q92598	Heat shock protein 105 kDa	HSPH1			
P56537	Eukaryotic translation initiation factor 6	EIF6			
Q13478	Interleukin-18 receptor 1	IL18R1			

P05198	Eukaryotic translation initiation factor 2 subunit 1	EIF2S1			
P09565	Putative insulin-like growth factor 2-associated protein	GIG44			
Q8NBZ0	INO80 complex subunit E	INO80E			
Q8WWN9	Interactor protein for cytohesin exchange factors 1	IPCEF1			
Q9UMF0	Intercellular adhesion molecule 5	ICAM5			
A6NGN9	IgLON family member 5	IGLON5			
P52294	Importin subunit alpha-5	KPNA1			
Q7Z5L9	Interferon regulatory factor 2-binding protein 2	IRF2BP2			
Q9BTL4	Immediate early response gene 2 protein	IER2			
P07099	Epoxide hydrolase 1	EPHX1			
Q8IVH4	Methylmalonic aciduria type A protein, mitochondrial	MMAA			
Q9BQA1	Methylosome protein 50	WDR77			
Q96EY8	Corrinoid adenosyltransferase	MMAB			
Q96FF7	Uncharacterized protein MISP3	MISP3			
Q8IVT2	Mitotic interactor and substrate of PLK1	MISP			
Q8NDC0	MAPK-interacting and spindle-stabilizing protein-like	MAPK1IP1L			
P23677	Inositol-trisphosphate 3-kinase A	ITPKA			
Q8NCT3	Uncharacterized protein KIAA0895	KIAA0895			
Q9UKX5	Integrin alpha-11	ITGA11			
O94854	Uncharacterized protein KIAA0754	KIAA0754			
Q6ICG6	Uncharacterized protein KIAA0930	KIAA0930			
Q9Y287	Integral membrane protein 2B	ITM2B			
P13646	Keratin, type I cytoskeletal 13	KRT13			
Q14573	Inositol 1,4,5-trisphosphate receptor type 3	ITPR3			
Q2M2I5	Keratin, type I cytoskeletal 24	KRT24			
O95050	Indolethylamine N-methyltransferase	INMT			
Q99550	M-phase phosphoprotein 9	MPHOSPH			
Q9NR64	Kelch-like protein 1	KLHL1			
Q8NCY6	Myb/SANT-like DNA-binding domain-containing protein 4	MSANTD4			
Q8TDN1	Potassium voltage-gated channel subfamily G member 4	KCNG4			
Q6IPM2	IQ domain-containing protein E	IQCE			
P10914	Interferon regulatory factor 1	IRF1			
Q9UK32	Ribosomal protein S6 kinase alpha-6	RPS6KA6			
Q8N5S9	Calcium/calmodulin-dependent protein kinase kinase 1	CAMKK1			
Q13976	cGMP-dependent protein kinase 1	PRKG1			
Q8NAB2	Kelch repeat and BTB domain-containing protein 3	KBTBD3			
Q5TKA1	Protein lin-9 homolog	LIN9			
O43813	Glutathione S-transferase LANCL1	LANCL1			
Q9HBX8	Leucine-rich repeat-containing G-protein coupled receptor 6	LGR6			
O95835	Serine/threonine-protein kinase LATS1	LATS1			
Q9Y561	Low-density lipoprotein receptor-related protein 12	LRP12			
Q96KN4	Protein LRATD1	LRATD1			
Q9NZR2	Low-density lipoprotein receptor-related protein 1B	LRP1B			
Q96M69	Leucine-rich repeat and guanylate kinase domain-containing protein	LRGUK			
Q12907	Vesicular integral-membrane protein VIP36	LMAN2			
P49257	Protein ERGIC-53	LMAN1			
O95274	Ly6/PLAUR domain-containing protein 3	LYPD3			

Q8N9Z9	Lamin tail domain-containing protein 1	LMNTD1			
P01701	Immunoglobulin lambda variable 1-51	IGLV1-51			
Q9UKM7	Endoplasmic reticulum mannosyl-oligosaccharide 1,2-alpha-mannosidase	MAN1B1			
A0A087WXM9	Meiosis-specific kinetochore protein	MEIKIN			
Q9H944	Mediator of RNA polymerase II transcription subunit 20	MED20			
Q7Z7M0	Multiple epidermal growth factor-like domains protein 8	MEGF8			
Q99558	Mitogen-activated protein kinase kinase kinase 14	MAP3K14			
Q7L590	Protein MCM10 homolog	MCM10			
P25106	Atypical chemokine receptor 3	ACKR3			
P08912	Muscarinic acetylcholine receptor M5	CHRM5			
P45954	Short/branched chain specific acyl-CoA dehydrogenase, mitochondrial	ACADSB			
Q8TDN7	Alkaline ceramidase 1	ACER1			
P86434	Putative uncharacterized protein ADORA2A-AS1	ADORA2A-AS1			
Q8IW93	Rho guanine nucleotide exchange factor 19	ARHGEF19			
Q9Y4X5	E3 ubiquitin-protein ligase ARIH1	ARIH1			
Q9NWL6	Asparagine synthetase domain-containing protein 1	ASNSD1			
P42575	Caspase-2	CASP2			
Q96JN2	Coiled-coil domain-containing protein 136	CCDC136			
Q8N998	Coiled-coil domain-containing protein 89	CCDC89			
Q9NVE4	Coiled-coil domain-containing protein 87	CCDC87			
Q8NAP1	Putative protein CASTOR 3	CASTOR3			
P21926	CD9 antigen	CD9			
Q9HD42	Charged multivesicular body protein 1a	CHMP1A			
A8MTB9	Carcinoembryonic antigen-related cell adhesion molecule 18	CEACAM18			
Q96N23	Cilia- and flagella-associated protein 54	CFAP54			
Q15782	Chitinase-3-like protein 2	CHI3L2			
P06731	Carcinoembryonic antigen-related cell adhesion molecule 5	CEACAM5			
Q16543	Hsp90 co-chaperone Cdc37	CDC37			
Q6ZU64	Cilia- and flagella-associated protein 65	CFAP65			
Q6IQ19	Centriole, cilia and spindle-associated protein	CCSAP			
F2Z3F1	Uncharacterized protein C5orf67	C5orf67			
P86791	Vacuolar fusion protein CCZ1 homolog	CCZ1			
P28907	ADP-ribosyl cyclase/cyclic ADP-ribose hydrolase 1	CD38			
P49454	Centromere protein F	CENPF			
Q96G23	Ceramide synthase 2	CERS2			
B2RD01	CENPB DNA-binding domain-containing protein 1	CENPBD1			
Q5EE01	Centromere protein W	CENPW			
P11597	Cholesteryl ester transfer protein	CETP			
Q86WS4	Uncharacterized protein C12orf40	C12orf40			
Q8NBR9	Uncharacterized protein C11orf72	C11orf72			
P26441	Ciliary neurotrophic factor	CNTF			
Q07065	Cytoskeleton-associated protein 4	CKAP4			

O75746	Calcium-binding mitochondrial carrier protein Aralar1	SLC25A12			
P23946	Chymase	CMA1			
P09919	Granulocyte colony-stimulating factor	CSF3			
P68400	Casein kinase II subunit alpha	CSNK2A1			
Q96SM3	Probable carboxypeptidase X1	CPXM1			
Q96EV8	Dysbindin	DTNBP1			
Q13115	Dual specificity protein phosphatase 4	DUSP4			
P29692	Elongation factor 1-delta	EEF1D			
Q92784	Zinc finger protein DPF3	DPF3			
Q9H8V3	Protein ECT2	ECT2			
Q6XUX3	Dual serine/threonine and tyrosine protein kinase	DSTYK			
Q8NEX9	Short-chain dehydrogenase/reductase family 9C member 7	SDR9C7			
O15218	G-protein coupled receptor 182	GPR182			
Q658K8	Putative elongation factor 1-delta-like protein	EEF1DP3			
P16444	Dipeptidase 1	DPEP1			
P30046	D-dopachrome decarboxylase	DDT			
Q9NYP3	Protein downstream neighbor of Son	DONSON			
Q9NWQ4	G patch domain-containing protein 2-like	GPATCH2L			
P16422	Epithelial cell adhesion molecule	EPCAM			
Q8NDI1	EH domain-binding protein 1	EHBP1			
O75355	Ectonucleoside triphosphate diphosphohydrolase 3	ENTPD3			
O15372	Eukaryotic translation initiation factor 3 subunit H	EIF3H			
Q6ZNA5	Ferric-chelate reductase 1	FRRS1			
Q9UPW0	Forkhead box protein J3	FOXJ3			
P0DPI2	Glutamine amidotransferase-like class 1 domain-containing protein 3A, mitochondrial	GATD3A			
Q86VQ1	Glucocorticoid-induced transcript 1 protein	GLCCI1			
Q8TF64	PDZ domain-containing protein GIPC3	GIPC3			
A1L429	G antigen 12B/C/D/E	GAGE12B;			
Q9NZ52	ADP-ribosylation factor-binding protein GGA3	GGA3			
P0CL80	G antigen 12F	GAGE12F			
P10075	Zinc finger protein GLI4	GLI4			
P10071	Transcriptional activator GLI3	GLI3			
O75496	Geminin	GMNN			
Q02742	Beta-1,3-galactosyl-O-glycosyl-glycoprotein beta-1,6-N-acetylglucosaminyltransferase	GCNT1			
Q17RS7	Flap endonuclease GEN homolog 1	GEN1			
O43555	Progonadoliberin-2	GNRH2			
P63215	Guanine nucleotide-binding protein G	GNG3			
P78334	Gamma-aminobutyric acid receptor subunit epsilon	GABRE			
P18507	Gamma-aminobutyric acid receptor subunit gamma-2	GABRG2			
P35754	Glutaredoxin-1	GLRX			
Q8N3Z3	GTP-binding protein 8	GTPBP8			
Q16661	Guanylate cyclase activator 2B	GUCA2B			
O60814	Histone H2B type 1-K	H2BC12			
P0CG30	Glutathione S-transferase theta-2B	GSTT2B			
Q4G1C9	GLIPR1-like protein 2	GLIPR1L2			

P32298	G protein-coupled receptor kinase 4	GRK4			
Q16099	Glutamate receptor ionotropic, kainate 4	GRIK4			
Q16665	Hypoxia-inducible factor 1-alpha	HIF1A			
Q6WQI6	Putative cancer susceptibility gene HEPN1 protein	HEPN1			
Q9XRX5	HERV-H LTR-associating protein 3	HHLA3			
Q9Y5Z4	Heme-binding protein 2	HEBP2			
P17483	Homeobox protein Hox-B4	HOXB4			
Q9NVR2	Integrator complex subunit 10	INTS10			
Q15306	Interferon regulatory factor 4	IRF4			
A6NK06	Cis-aconitate decarboxylase	ACOD1			
Q3LI68	Keratin-associated protein 22-2	KRTAP22-2			
Q15349	Ribosomal protein S6 kinase alpha-2	RPS6KA2			
O76009	Keratin, type I cuticular Ha3-I	KRT33A			
Q9BYR6	Keratin-associated protein 3-3	KRTAP3-3			
Q9NVR0	Kelch-like protein 11	KLHL11			
Q9BYR7	Keratin-associated protein 3-2	KRTAP3-2			
C9JR72	Kelch repeat and BTB domain-containing protein 13	KBTBD13			
Q9GZZ8	Extracellular glycoprotein lacritin	LACRT			
Q16659	Mitogen-activated protein kinase 6	MAPK6			
Q86YT6	E3 ubiquitin-protein ligase MIB1	MIB1			
O60882	Matrix metalloproteinase-20	MMP20			
Q9BRJ9	Mesoderm posterior protein 1	MESP1			
Q9GZT9	Egl nine homolog 1	EGLN1			
Q8IUS5	Epoxide hydrolase 4	EPHX4			
Q9UNN8	Endothelial protein C receptor	PROCR			
Q14232	Translation initiation factor eIF-2B subunit alpha	EIF2B1			
Q5JST6	EF-hand domain-containing family member C2	EFHC2			
Q92913	Fibroblast growth factor 13	FGF13			
Q8N475	Follistatin-related protein 5	FSTL5			
O76087	G antigen 7	GAGE7			
O60548	Forkhead box protein D2	FOXD2			
Q96NZ1	Forkhead box protein N4	FOXN4			
Q96C23	Galactose mutarotase	GALM			
Q12946	Forkhead box protein F1	FOXF1			
A1L4K1	Fibronectin type III and SPRY domain-containing protein 2	FSD2			
Q9UBC7	Galanin-like peptide	GALP			
P09681	Gastric inhibitory polypeptide	GIP			
P22749	Granulysin	GNLY			
Q96EK6	Glucosamine 6-phosphate N-acetyltransferase	GNPNAT1			
Q6ZN66	Guanylate-binding protein 6	GBP6			
P12544	Granzyme A	GZMA			
D6RF30	Golgin subfamily A member 8K	GOLGA8K			
Q3T906	N-acetylglucosamine-1-phosphotransferase subunits alpha/beta	GNPTAB			
O14626	Probable G-protein coupled receptor 171	GPR171			
Q86WP2	Vasculin	GPBP1			
P43304	Glycerol-3-phosphate dehydrogenase, mitochondrial	GPD2			
Q02643	Growth hormone-releasing hormone receptor	GHRHR			
Q9UJ14	Glutathione hydrolase 7	GGT7			
P04150	Glucocorticoid receptor	NR3C1			
Q9H2K0	Translation initiation factor IF-3, mitochondrial	MTIF3			
O43837	Isocitrate dehydrogenase	IDH3B			
P19367	Hexokinase-1	HK1			
P52789	Hexokinase-2	HK2			
O75874	Isocitrate dehydrogenase	IDH1			

Q16666	Gamma-interferon-inducible protein 16	IFI16			
P34913	Bifunctional epoxide hydrolase 2	EPHX2			
Q9UG01	Intraflagellar transport protein 172 homolog	IFT172			
P52790	Hexokinase-3	HK3			
P47813	Eukaryotic translation initiation factor 1A, X-chromosomal	EIF1AX			
O14602	Eukaryotic translation initiation factor 1A, Y-chromosomal	EIF1AY			
Q9HCN6	Platelet glycoprotein VI	GP6			
O75311	Glycine receptor subunit alpha-3	GLRA3			
Q7Z4T8	Inactive polypeptide N-acetylgalactosaminyltransferase-like protein 5	GALNTL5			
Q49A17	Polypeptide N-acetylgalactosaminyltransferase-like 6	GALNTL6			
Q16775	Hydroxyacylglutathione hydrolase, mitochondrial	HAGH			
P49685	G-protein coupled receptor 15	GPR15			
O15063	Granule associated Rac and RHOG effector protein 1	GARRE1			
P30550	Gastrin-releasing peptide receptor	GRPR			
Q6NXT2	Histone H3.3C	H3-5			
Q92896	Golgi apparatus protein 1	GLG1			
Q14833	Metabotropic glutamate receptor 4	GRM4			
Q9BXW7	Haloacid dehalogenase-like hydrolase domain-containing 5	HDHD5			
P54257	Huntingtin-associated protein 1	HAP1			
Q7Z2Y8	Interferon-induced very large GTPase 1	GVINP1			
O60243	Heparan-sulfate 6-O-sulfotransferase 1	HS6ST1			
Q9BYK8	Helicase with zinc finger domain 2	HELZ2			
Q9Y543	Transcription factor HES-2	HES2			
P01825	Immunoglobulin heavy variable 4-59	IGHV4-59			
Q9UBD0	Heat shock transcription factor, X-linked	HSFX1			
P09913	Interferon-induced protein with tetratricopeptide repeats 2	IFIT2			
P20823	Hepatocyte nuclear factor 1-alpha	HNF1A			
Q9HB29	Interleukin-1 receptor-like 2	IL1RL2			
Q6U949	Putative insulin-like growth factor 2 antisense gene protein	IGF2-AS			
P15814	Immunoglobulin lambda-like polypeptide 1	IGLL1			
P01861	Immunoglobulin heavy constant gamma 4	IGHG4			
O00221	NF-kappa-B inhibitor epsilon	NFKBIE			
Q8NEV9	Interleukin-27 subunit alpha	IL27			
P13598	Intercellular adhesion molecule 2	ICAM2			
Q2VIR3	Eukaryotic translation initiation factor 2 subunit 3B	EIF2S3B			
Q86T90	Protein hinderin	KIAA1328			
Q9UKP3	Integrin beta-1-binding protein 2	ITGB1BP2			
Q6ZSG2	Inhibitory synaptic factor 2A	INSYN2A			
O94889	Kelch-like protein 18	KLHL18			
Q9HAQ2	Kinesin-like protein KIF9	KIF9			
Q6PIU1	Potassium voltage-gated channel subfamily V member 1	KCNV1			
Q15418	Ribosomal protein S6 kinase alpha-1	RPS6KA1			
Q8N1A0	Keratin-like protein KRT222	KRT222			
P0C7H8	Keratin-associated protein 2-3	KRTAP2-3			
Q8NBH2	Kyphoscoliosis peptidase	KY			

Q8WVZ9	Kelch repeat and BTB domain-containing protein 7	KBTBD7			
Q9UPQ0	LIM and calponin homology domains-containing protein 1	LIMCH1			
Q9H239	Matrix metalloproteinase-28	MMP28			
Q5HYA8	Meckelin	TMEM67			
Q8IY33	MICAL-like protein 2	MICALL2			
Q01449	Myosin regulatory light chain 2, atrial isoform	MYL7			
O14880	Microsomal glutathione S-transferase 3	MGST3			
Q99735	Microsomal glutathione S-transferase 2	MGST2			
O00587	Beta-1,3-N-acetylglucosaminyltransferase manic fringe	MFNG			
Q5VWP3	Muscular LMNA-interacting protein	MLIP			
P0DMC3	Apelin receptor early endogenous ligand	APELA			
Q8IYF1	Elongin-A2	ELOA2			
Q96KQ7	Histone-lysine N-methyltransferase EHMT2	EHMT2			
Q8TE67	Epidermal growth factor receptor kinase substrate 8-like protein 3	EPS8L3			
Q14674	Separin	ESPL1			
Q8IYD1	Eukaryotic peptide chain release factor GTP-binding subunit ERF3B	GSPT2			
Q9Y247	Protein FAM50B	FAM50B			
Q8NDB6	Protein FAM156A/FAM156B	FAM156A			
P24071	Immunoglobulin alpha Fc receptor	FCAR			
Q9Y2H6	Fibronectin type-III domain-containing protein 3A	FNDC3A			
P31512	Dimethylaniline monooxygenase	FMO4			
A0FGR8	Extended synaptotagmin-2	ESYT2			
P10412	Histone H1.4	H1-4			
P23610	40-kDa huntingtin-associated protein	F8A1;			
Q8N6M3	Acyl-coenzyme A diphosphatase FITM2	FITM2			
P0C2W1	F-box/SPRY domain-containing protein 1	FBXO45			
O75426	F-box only protein 24	FBXO24			
P21333	Filamin-A	FLNA			
Q8IX29	F-box only protein 16	FBXO16			
Q9UKT8	F-box/WD repeat-containing protein 2	FBXW2			
P02751	Fibronectin	FN1			
B1AJZ9	Forkhead-associated domain-containing protein 1	FHAD1			
Q03591	Complement factor H-related protein 1	CFHR1			
P98095	Fibulin-2	FBLN2			
O15552	Free fatty acid receptor 2	FFAR2			
Q5H8C1	FRAS1-related extracellular matrix protein 1	FREM1			
P57057	Glucose-6-phosphate exchanger SLC37A1	SLC37A1			
Q06546	GA-binding protein alpha chain	GABPA			
Q9P2B2	Prostaglandin F2 receptor negative regulator	PTGFRN			
Q8IWF2	FAD-dependent oxidoreductase domain-containing protein 2	FOXRED2			
P22466	Galanin peptides	GAL			
Q14376	UDP-glucose 4-epimerase	GALE			
Q92820	Gamma-glutamyl hydrolase	GGH			
P36269	Glutathione hydrolase 5 proenzyme	GGT5			

Q9UMX6	Guanylyl cyclase-activating protein 2	GUCA1B			
P62807	Histone H2B type 1-C/E/F/G/I	H2BC4			
Q75N03	E3 ubiquitin-protein ligase Hakai	CBLL1			
Q8N257	Histone H2B type 3-B	H2BU1			
P42262	Glutamate receptor 2	GRIA2			
A8MV81	HIG1 domain family member 1C	HIGD1C			
Q8IZP7	Heparan-sulfate 6-O-sulfotransferase 3	HS6ST3			
Q04756	Hepatocyte growth factor activator	HGFAC			
Q13571	Lysosomal-associated transmembrane protein 5	LAPTM5			
Q86WZ0	HEAT repeat-containing protein 4	HEATR4			
P42858	Huntingtin	HTT			
P54652	Heat shock-related 70 kDa protein 2	HSPA2			
P09629	Homeobox protein Hox-B7	HOXB7			
P0DP01	Immunoglobulin heavy variable 1-8	IGHV1-8			
Q14103	Heterogeneous nuclear ribonucleoprotein D0	HNRNPD			
Q8IWW8	Hydroxyacid-oxoacid transhydrogenase, mitochondrial	ADHFE1			
Q9Y5L2	Hypoxia-inducible lipid droplet-associated protein	HILPDA			
P0DMR1	Heterogeneous nuclear ribonucleoprotein C-like 4	HNRNPCL4			
Q1KMD3	Heterogeneous nuclear ribonucleoprotein U-like protein 2	HNRNPUL2			
P13232	Interleukin-7	IL7			
Q13418	Integrin-linked protein kinase	ILK			
P60842	Eukaryotic initiation factor 4A-I	EIF4A1			
P0DOX7	Immunoglobulin kappa light chain				
Q9H583	HEAT repeat-containing protein 1	HEATR1			
Q9BYH8	NF-kappa-B inhibitor zeta	NFKBIZ			
O75144	ICOS ligand	ICOSLG			
Q14773	Intercellular adhesion molecule 4	ICAM4			
Q8N6C5	Immunoglobulin superfamily member 1	IGSF1			
O95998	Interleukin-18-binding protein	IL18BP			
Q99456	Keratin, type I cytoskeletal 12	KRT12			
Q86VI3	Ras GTPase-activating-like protein IQGAP3	IQGAP3			
Q96AB3	Isochorismatase domain-containing protein 2	ISOC2			
Q9NRR6	Phosphatidylinositol polyphosphate 5-phosphatase type IV	INPP5E			
Q5VWX1	KH domain-containing, RNA-binding, signal transduction-associated protein 2	KHDRBS2			
Q9UBX7	Kallikrein-11	KLK11			
Q9Y337	Kallikrein-5	KLK5			
Q3LI83	Keratin-associated protein 24-1	KRTAP24-1			
Q9HB15	Potassium channel subfamily K member 12	KCNK12			
Q9BVA0	Katanin p80 WD40 repeat-containing subunit B1	KATNB1			
P60413	Keratin-associated protein 10-12	KRTAP10-12			
Q96NX5	Calcium/calmodulin-dependent protein kinase type 1G	CAMK1G			
A0A0A0MT36	Immunoglobulin kappa variable 6D-21	IGKV6D-21			
Q9UBR4	LIM/homeobox protein Lhx3	LHX3			
P01593	Immunoglobulin kappa variable 1D-33	IGKV1D-33			
Q6ZQX7	Protein LIAT1	LIAT1			

Q95447	Lebercilin-like protein	LCA5L			
Q92615	La-related protein 4B	LARP4B			
Q7L985	Leucine-rich repeat and immunoglobulin-like domain-containing nogo receptor-interacting protein 2	LINGO2			
Q5BKY1	Leucine-rich repeat-containing protein 10	LRRC10			
Q96E66	Leucine-rich repeat-containing protein 51	LRTOMT			
Q9HCP0	Casein kinase I isoform gamma-1	CSNK1G1			
Q7L1W4	Volume-regulated anion channel subunit LRRC8D	LRRC8D			
Q16609	Putative apolipoprotein	LPAL2			
Q9HCC9	Lateral signaling target protein 2 homolog	ZFYVE28			
Q8NI32	Ly6/PLAUR domain-containing protein 6B	LYPD6B			
Q96JM4	Leucine-rich repeat and IQ domain-containing protein 1	LRRIQ1			
Q9P2V4	Leucine-rich repeat, immunoglobulin-like domain and transmembrane domain-containing protein 1	LRIT1			
O95711	Lymphocyte antigen 86	LY86			
P07098	Gastric triacylglycerol lipase	LIPF			
Q8WWY8	Lipase member H	LIPH			
Q5VXI9	Lipase member N	LIPN			
P22888	Lutropin-choriogonadotropic hormone receptor	LHCGR			
A6NCF6	Putative MAGE domain-containing protein MAGEA13P	MAGEA13P			
O43772	Mitochondrial carnitine/acylcarnitine carrier protein	SLC25A20			
A0A075B6I9	Immunoglobulin lambda variable 7-46	IGLV7-46			
A8MW99	Meiosis-specific protein MEI4	MEI4			
Q4G0Z9	Minichromosome maintenance domain-containing protein 2	MCMDC2			
Q16584	Mitogen-activated protein kinase kinase kinase 11	MAP3K11			
P04201	Proto-oncogene Mas	MAS1			
Q9NS73	MAP3K12-binding inhibitory protein 1	MBIP			
Q8IVH8	Mitogen-activated protein kinase kinase kinase kinase 3	MAP4K3			
Q92918	Mitogen-activated protein kinase kinase kinase kinase 1	MAP4K1			
Q5TCQ9	Membrane-associated guanylate kinase, WW and PDZ domain-containing protein 3	MAGI3			
Q9Y5Q3	Transcription factor MafB	MAFB			
P43359	Melanoma-associated antigen 5	MAGEA5			
O15116	U6 snRNA-associated Sm-like protein LSm1	LSM1			
Q9BZ81	Melanoma-associated antigen B5	MAGEB5			
Q7L2J0	7SK snRNA methylphosphate capping enzyme	MEPCE			
Q0PNE2	Elongator complex protein 6	ELP6			
Q15369	Elongin-C	ELOC			
Q05BV3	Echinoderm microtubule-associated protein-like 5	EML5			
P98172	Ephrin-B1	EFNB1			
Q658T7	Putative protein FAM90A2P	FAM90A2P			
Q03468	DNA excision repair protein ERCC-6	ERCC6			
P50549	ETS translocation variant 1	ETV1			
P43268	ETS translocation variant 4	ETV4			

A0A1W2PQ73	ETS domain-containing transcription factor ERF-like	ERFL			
Q96DN0	Endoplasmic reticulum resident protein 27	ERP27			
Q9NYK6	Protein EURL homolog	EURL			
Q9UNN5	FAS-associated factor 1	FAF1			
P49640	Homeobox even-skipped homolog protein 1	EVX1			
Q6P2I3	Fumarylacetoacetate hydrolase domain-containing protein 2B	FAHD2B			
A8K979	ERI1 exoribonuclease 2	ERI2			
Q8TC84	Fibronectin type 3 and ankyrin repeat domains protein 1	FANK1			
Q9Y2C4	Nuclease EXOG, mitochondrial	EXOG			
Q8IXR5	Protein FAM178B	FAM178B			
P13804	Electron transfer flavoprotein subunit alpha, mitochondrial	ETFA			
Q9NW38	E3 ubiquitin-protein ligase FANCL	FANCL			
Q5T890	DNA excision repair protein ERCC-6-like 2	ERCC6L2			
P08637	Low affinity immunoglobulin gamma Fc region receptor III-A	FCGR3A			
Q8NI29	F-box only protein 27	FBXO27			
Q9UK99	F-box only protein 3	FBXO3			
Q6PJ61	F-box only protein 46	FBXO46			
Q08830	Fibrinogen-like protein 1	FGL1			
Q9C0D6	FH2 domain-containing protein 1	FHDC1			
P42685	Tyrosine-protein kinase FRK	FRK			
Q11130	Alpha-	FUT7			
P22083	Alpha-	FUT4			
Q3SYB3	Forkhead box protein D4-like 6	FOXD4L6			
Q9BRQ8	Ferroptosis suppressor protein 1	AIFM2			
Q9Y261	Hepatocyte nuclear factor 3-beta	FOXA2			
Q12948	Forkhead box protein C1	FOXC1			
Q96F15	GTPase IMAP family member 5	GIMAP5			
P0CG01	Gastrokine-3	GKN3P			
Q9NZC3	Glycerophosphodiester phosphodiesterase 1	GDE1			
Q5JRK9	Putative G antigen family E member 3	PAGE2B			
Q6UXV0	GDNF family receptor alpha-like	GFRAL			
O60383	Growth/differentiation factor 9	GDF9			
O43681	ATPase GET3	GET3			
Q5UAW9	G-protein coupled receptor 157	GPR157			
O95467	Neuroendocrine secretory protein 55	GNAS			
P59796	Glutathione peroxidase 6	GPX6			
Q14956	Transmembrane glycoprotein NMB	GPNMB			
Q8NBJ5	Procollagen galactosyltransferase 1	COLGALT1			
Q14687	Genetic suppressor element 1	GSE1			
A0A1W2PR19	Glutathione S-transferase theta-4	GSTT4			
P21266	Glutathione S-transferase Mu 3	GSTM3			
A4D1Z8	Grifin	GRIFIN			
P60008	Putative spermatid-specific linker histone H1-like protein	H1-9P			
Q9NWT6	Hypoxia-inducible factor 1-alpha inhibitor	HIF1AN			
Q9UBP5	Hairy/enhancer-of-split related with YRPW motif protein 2	HEY2			
P13196	5-aminolevulinate synthase, nonspecific, mitochondrial	ALAS1			
Q8IVU3	Probable E3 ubiquitin-protein ligase HERC6	HERC6			
Q8NG08	DNA helicase B	HELB			
P52272	Heterogeneous nuclear ribonucleoprotein M	HNRNPM			

A0A0C4DH29	Immunoglobulin heavy variable 1-3	IGHV1-3			
A0A0G2JMI3	Immunoglobulin heavy variable 1-69-2	IGHV1-69-2			
A2RU54	Homeobox protein HMX2	HMX2			
Q92619	Rho GTPase-activating protein 45	ARHGAP45			
O60812	Heterogeneous nuclear ribonucleoprotein C-like 1	HNRNPCL1			
P0CJ71	Humanin-like 4	MTRNR2L4			
P0CJ74	Humanin-like 7	MTRNR2L7			
P10145	Interleukin-8	CXCL8			
Q53G44	Interferon-induced protein 44-like	IFI44L			
Q13007	Interleukin-24	IL24			
Q6WRI0	Immunoglobulin superfamily member 10	IGSF10			
Q14974	Importin subunit beta-1	KPNB1			
P00995	Serine protease inhibitor Kazal-type 1	SPINK1			
Q9BY89	Uncharacterized protein KIAA1671	KIAA1671			
Q96S16	JmjC domain-containing protein 8	JMJD8			
Q9HDC5	Junctophilin-1	JPH1			
Q8IVT5	Kinase suppressor of Ras 1	KSR1			
Q9UGL1	Lysine-specific demethylase 5B	KDM5B			
Q14525	Keratin, type I cuticular Ha3-II	KRT33B			
P60328	Keratin-associated protein 12-3	KRTAP12-3			
Q86Y97	Histone-lysine N-methyltransferase KMT5C	KMT5C			
Q13237	cGMP-dependent protein kinase 2	PRKG2			
Q8WVF5	BTB/POZ domain-containing protein KCTD4	KCTD4			
Q3ZCT8	Kelch repeat and BTB domain-containing protein 12	KBTBD12			
Q5VSP4	Putative lipocalin 1-like protein 1	LCN1P1			
Q5TCM9	Late cornified envelope protein 5A	LCE5A			
Q8N0V4	Leucine-rich repeat LGI family member 2	LGI2			
Q96L11	Sperm-egg fusion protein LLCFC1	LLCFC1			
Q92604	Acyl-CoA:lysophosphatidylglycerol acyltransferase 1	LPGAT1			
Q13164	Mitogen-activated protein kinase 7	MAPK7			
Q9H492	Microtubule-associated proteins 1A/1B light chain 3A	MAP1LC3A			
A6NFX1	Major facilitator superfamily domain-containing protein 2B	MFSD2B			
Q10571	Transcriptional activator MN1	MN1			
O95163	Elongator complex protein 1	ELP1			
Q8NG57	Elongin-A3	ELOA3P			
P61568	Putative endogenous retrovirus group K member 11-1 Env polyprotein	ERVK11-1			
Q96AY2	Crossover junction endonuclease EME1	EME1			
Q9NR50	Translation initiation factor eIF-2B subunit gamma	EIF2B3			
P18074	General transcription and DNA repair factor IIH helicase subunit XPD	ERCC2			
A6NKC0	Putative protein FAM90A7P	FAM90A7P			
Q6NXP2	Protein FAM71F2	FAM71F2			
Q96PL5	Erythroid membrane-associated protein	ERMAP			
Q14534	Squalene monooxygenase	SQLE			
P0C841	Putative protein FAM66E	FAM66E			
Q86V87	Protein FAM160B2	FAM160B2			
P58499	Protein FAM3B	FAM3B			
Q3ZM63	Embryonic testis differentiation protein homolog A	ETDA			

P38117	Electron transfer flavoprotein subunit beta	ETFB			
Q9C073	Protein FAM117A	FAM117A			
Q9NQT5	Exosome complex component RRP40	EXOSC3			
P0CF97	Protein FAM200B	FAM200B			
Q6UXB0	Protein FAM131A	FAM131A			
P15036	Protein C-ets-2	ETS2			
Q5XKK7	Protein FAM219B	FAM219B			
Q86W67	Protein FAM228A	FAM228A			
A5PLN7	Protein FAM149A	FAM149A			
Q86X51	EZH inhibitory protein	EZHIP			
O43909	Exostosin-like 3	EXTL3			
P16591	Tyrosine-protein kinase Fer	FER			
Q01543	Friend leukemia integration 1 transcription factor	FLI1			
P62942	Peptidyl-prolyl cis-trans isomerase FKBP1A	FKBP1A			
Q8NFG4	Folliculin	FLCN			
Q8NFZ0	F-box DNA helicase 1	FBH1			
Q86VR8	Four-jointed box protein 1	FJX1			
Q13045	Protein flightless-1 homolog	FLII			
P02679	Fibrinogen gamma chain	FGG			
P02671	Fibrinogen alpha chain	FGA			
Q7Z6J4	FYVE, RhoGEF and PH domain-containing protein 2	FGD2			
Q5SZK8	FRAS1-related extracellular matrix protein 2	FREM2			
P09038	Fibroblast growth factor 2	FGF2			
P11487	Fibroblast growth factor 3	FGF3			
Q6VB84	Forkhead box protein D4-like 3	FOXD4L3			
O75899	Gamma-aminobutyric acid type B receptor subunit 2	GABBR2			
Q11128	4-galactosyl-N-acetylglucosaminide 3-alpha-L-fucosyltransferase FUT5	FUT5			
A0A1W2PRP0	Forkhead box protein L3	FOXL3			
Q6PIV2	Forkhead box protein R1	FOXR1			
P14207	Folate receptor beta	FOLR2			
Q8IVK1	Putative glycosylation-dependent cell adhesion molecule 1	GLYCAM1			
A6NGU5	Putative glutathione hydrolase 3 proenzyme	GGT3P			
Q6PIY7	Poly	TENT2			
A8MZA4	Golgin subfamily A member 6-like protein 6	GOLGA6L6			
Q9UJY4	ADP-ribosylation factor-binding protein GGA2	GGA2			
Q9Y2X7	ARF GTPase-activating protein GIT1	GIT1			
Q9GZZ7	GDNF family receptor alpha-4	GFRA4			
O75343	Guanylate cyclase soluble subunit beta-2	GUCY1B2			
Q92538	Golgi-specific brefeldin A-resistance guanine nucleotide exchange factor 1	GBF1			
P14867	Gamma-aminobutyric acid receptor subunit alpha-1	GABRA1			
P08754	Guanine nucleotide-binding protein G	GNAI3			
P47869	Gamma-aminobutyric acid receptor subunit alpha-2	GABRA2			
Q8WU03	Glycine N-acyltransferase-like protein 2	GLYATL2			
H3BV12	Golgin subfamily A member 8Q	GOLGA8Q			
Q0D2H9	Putative golgin subfamily A member 8D	GOLGA8DP			
H3BPF8	Golgin subfamily A member 8S	GOLGA8S			

Q9HCL2	Glycerol-3-phosphate acyltransferase 1, mitochondrial	GPAM			
Q7Z3F1	Integral membrane protein GPR155	GPR155			
Q8NFN8	Probable G-protein coupled receptor 156	GPR156			
P04921	Glycophorin-C	GYPC			
Q96I76	G patch domain-containing protein 3	GPATCH3			
Q9UKJ3	G patch domain-containing protein 8	GPATCH8			
P36959	GMP reductase 1	GMPR			
P36969	Phospholipid hydroperoxide glutathione peroxidase	GPX4			
P47775	G-protein coupled receptor 12	GPR12			
Q8NDV2	G-protein coupled receptor 26	GPR26			
Q8WZ60	Kelch-like protein 6	KLHL6			
Q9BXC0	Hydroxycarboxylic acid receptor 1	HCAR1			
Q9UHL9	General transcription factor II-I repeat domain-containing protein 1	GTF2IRD1			
Q13002	Glutamate receptor ionotropic, kainate 2	GRIK2			
O14929	Histone acetyltransferase type B catalytic subunit	HAT1			
Q9Y5J3	Hairy/enhancer-of-split related with YRPW motif protein 1	HEY1			
A0A0C4DH31	Immunoglobulin heavy variable 1-18	IGHV1-18			
P10321	HLA class I histocompatibility antigen, C alpha chain	HLA-C			
Q8IZI9	Interferon lambda-3	IFNL3			
Q16552	Interleukin-17A	IL17A			
B9A064	Immunoglobulin lambda-like polypeptide 5	IGLL5			
Q969J5	Interleukin-22 receptor subunit alpha-2	IL22RA2			
A6ND01	Sperm-egg fusion protein Juno	IZUMO1R			
Q9HCM3	UPF0606 protein KIAA1549	KIAA1549			
P17181	Interferon alpha/beta receptor 1	IFNAR1			
P09529	Inhibin beta B chain	INHBB			
Q8IZU9	Kin of IRRE-like protein 3	KIRREL3			
Q6PEX3	Keratin-associated protein 26-1	KRTAP26-1			
Q6NY19	KN motif and ankyrin repeat domain-containing protein 3	KANK3			
Q09470	Potassium voltage-gated channel subfamily A member 1	KCNA1			
Q96JN0	Ligand-dependent corepressor	LCOR			
Q71F78	Putative lung carcinoma-associated protein 10	LCA10			
P0DO92	Putative protein T-ENOL	CDIPTOSP			
Q96KS0	Prolyl hydroxylase EGLN2	EGLN2			
A4GXA9	Probable crossover junction endonuclease EME2	EME2			
P49411	Elongation factor Tu, mitochondrial	TUFM			
Q14152	Eukaryotic translation initiation factor 3 subunit A	EIF3A			
Q96BH3	Epididymal sperm-binding protein 1	ELSPBP1			
B6SEH8	Endogenous retrovirus group V member 1 Env polyprotein	ERVV-1			
P04626	Receptor tyrosine-protein kinase erbB-2	ERBB2			
Q96DZ1	Endoplasmic reticulum lectin 1	ERLEC1			
O75477	Erlin-1	ERLIN1			
Q9H8M9	Protein eva-1 homolog A	EVA1A			
P30040	Endoplasmic reticulum resident protein 29	ERP29			

O15529	G-protein coupled receptor 42	GPR42			
Q9H479	Fructosamine-3-kinase	FN3K			
Q8IX07	Zinc finger protein ZFPM1	ZFPM1			
P07332	Tyrosine-protein kinase Fes/Fps	FES			
Q9UK96	F-box only protein 10	FBXO10			
Q99581	Protein FEV	FEV			
Q96M78	Putative uncharacterized protein encoded by FER1L6-AS2	FER1L6-AS2			
P21802	Fibroblast growth factor receptor 2	FGFR2			
Q2V2M9	FH1/FH2 domain-containing protein 3	FHOD3			
P25089	N-formyl peptide receptor 3	FPR3			
O75474	GSK-3-binding protein FRAT2	FRAT2			
Q01167	Forkhead box protein K2	FOXK2			
Q12778	Forkhead box protein O1	FOXO1			
P54826	Growth arrest-specific protein 1	GAS1			
O60861	Growth arrest-specific protein 7	GAS7			
Q14161	ARF GTPase-activating protein GIT2	GIT2			
Q6KF10	Growth/differentiation factor 6	GDF6			
Q6ZMI3	Gliomedin	GLDN			
Q8TC17	GRB2-related adapter protein-like	GRAPL			
P50148	Guanine nucleotide-binding protein G	GNAQ			
Q8TDQ7	Glucosamine-6-phosphate isomerase 2	GNPDA2			
Q6UWK7	Protein GPR15L	GPR15L			
Q5T6J7	Probable gluconokinase	IDNK			
P62993	Growth factor receptor-bound protein 2	GRB2			
Q86UL3	Glycerol-3-phosphate acyltransferase 4	GPAT4			
P14735	Insulin-degrading enzyme	IDE			
Q13568	Interferon regulatory factor 5	IRF5			
Q02846	Retinal guanylyl cyclase 1	GUCY2D			
Q9Y2N7	Hypoxia-inducible factor 3-alpha	HIF3A			
Q14CZ8	Hepatocyte cell adhesion	HEPACAM			
Q5T447	E3 ubiquitin-protein ligase HECTD3	HECTD3			
P0DP08	Immunoglobulin heavy variable 4-38-2	IGHV4-38-2			
Q96MM6	Heat shock 70 kDa protein 12B	HSPA12B			
P13985	Putative HTLV-1-related endogenous sequence	HRES1			
Q9NSB8	Homer protein homolog 2	HOMER2			
O60760	Hematopoietic prostaglandin D synthase	HPGDS			
Q58FG1	Putative heat shock protein HSP 90-alpha A4	HSP90AA4P			
Q8TB92	3-hydroxy-3-methylglutaryl-CoA lyase, cytoplasmic	HMGCLL1			
P30519	Heme oxygenase 2	HMOX2			
P05231	Interleukin-6	IL6			
Q01629	Interferon-induced transmembrane protein 2	IFITM2			
P05000	Interferon omega-1	IFNW1			
Q9GZP8	Immortalization up-regulated protein	IMUP			
Q70UQ0	Inhibitor of nuclear factor kappa-B kinase-interacting protein	IKBIP			
Q8TAD2	Interleukin-17D	IL17D			
P23588	Eukaryotic translation initiation factor 4B	EIF4B			
P20042	Eukaryotic translation initiation factor 2 subunit 2	EIF2S2			
Q05084	Islet cell autoantigen 1	ICA1			
P26012	Integrin beta-8	ITGB8			

P61371	Insulin gene enhancer protein ISL-1	ISL1			
Q9P266	Junctional protein associated with coronary artery disease	JCAD			
P08514	Integrin alpha-IIb	ITGA2B			
A8MTL0	IQ domain-containing protein F5	IQCF5			
Q9Y5U4	Insulin-induced gene 2 protein	INSIG2			
A6NCM1	IQ and AAA domain-containing protein 1-like	IQCA1L			
Q03721	Potassium voltage-gated channel subfamily C member 4	KCNC4			
Q9ULD8	Potassium voltage-gated channel subfamily H member 3	KCNH3			
Q92830	Histone acetyltransferase KAT2A	KAT2A			
Q96KK3	Potassium voltage-gated channel subfamily S member 1	KCNS1			
O75600	2-amino-3-ketobutyrate coenzyme A ligase, mitochondrial	GCAT			
Q9UNX9	ATP-sensitive inward rectifier potassium channel 14	KCNJ14			
P31321	cAMP-dependent protein kinase type I-beta regulatory subunit	PRKAR1B			
P13861	cAMP-dependent protein kinase type II-alpha regulatory subunit	PRKAR2A			
Q9BYQ5	Keratin-associated protein 4-6	KRTAP4-6			
Q02156	Protein kinase C epsilon type	PRKCE			
Q9NQ69	LIM/homeobox protein Lhx9	LHX9			
Q5T5A8	Late cornified envelope protein 3C	LCE3C			
Q5T871	Late cornified envelope-like proline-rich protein 1	LELP1			
Q16787	Laminin subunit alpha-3	LAMA3			
Q5TA78	Late cornified envelope protein 4A	LCE4A			
Q7Z429	Protein lifeguard 1	GRINA			
Q68G75	LEM domain-containing protein 1	LEMD1			
Q9HBL6	Leucine-rich repeat and transmembrane domain-containing protein 1	LRTM1			
Q8N423	Leukocyte immunoglobulin-like receptor subfamily B member 2	LILRB2			
P01700	Immunoglobulin lambda variable 1-47	IGLV1-47			
P01706	Immunoglobulin lambda variable 2-11	IGLV2-11			
P01714	Immunoglobulin lambda variable 3-19	IGLV3-19			
Q6DN12	Multiple C2 and transmembrane domain-containing protein 2	MCTP2			
Q02779	Mitogen-activated protein kinase kinase kinase 10	MAP3K10			
P51608	Methyl-CpG-binding protein 2	MECP2			
P80192	Mitogen-activated protein kinase kinase kinase 9	MAP3K9			
Q99750	MyoD family inhibitor	MDFI			
Q6ZWT7	Lysophospholipid acyltransferase	MBOAT2			
Q9Y2X3	Nucleolar protein 58	NOP58			
Q08AI8	Protein mab-21-like 4	MAB21L4			
P11226	Mannose-binding protein C	MBL2			
O75354	Ectonucleoside triphosphate diphosphohydrolase 6	ENTPD6			
O15371	Eukaryotic translation initiation factor 3 subunit D	EIF3D			
A6NNW6	Enolase 4	ENO4			
Q9NPA8	Transcription and mRNA export factor ENY2	ENY2			
Q15303	Receptor tyrosine-protein kinase erbB-4	ERBB4			
Q96KT0	Uncharacterized protein FAM167A-AS1	FAM167A-AS1			
O95718	Steroid hormone receptor ERR2	ESRRB			

Q96Q35	Flagellum-associated coiled-coil domain-containing protein 1	FLACC1			
A6NNH2	Protein FAM90A27P	FAM90A27P			
Q8N5Q1	Protein FAM71E2	FAM71E2			
Q96MK3	Pseudokinase FAM20A	FAM20A			
Q6GMR7	Fatty-acid amide hydrolase 2	FAAH2			
Q9UKR5	Ergosterol biosynthetic protein 28 homolog	ERG28			
Q8IZU0	Protein FAM9B	FAM9B			
Q7L0X2	Glutamate-rich protein 6	ERICH6			
Q8IZU1	Protein FAM9A	FAM9A			
Q969W3	Protein FAM104A	FAM104A			
Q9NY74	Ewing's tumor-associated antigen 1	ETAA1			
O00423	Echinoderm microtubule-associated protein-like 1	EML1			
A6NL82	Protein FAM183A	FAM183A			
Q5SY85	Protein FAM201A	FAM201A			
Q8IXS8	Protein FAM126B	FAM126B			
O75616	GTPase Era, mitochondrial	ERAL1			
A0A1B0GVZ2	Protein FAM240B	FAM240B			
P49326	Flavin-containing monooxygenase 5	FMO5			
Q06828	Fibromodulin	FMOD			
O95677	Eyes absent homolog 4	EYA4			
Q8N3X1	Formin-binding protein 4	FNBP4			
P37268	Squalene synthase	FDFT1			
Q5XX13	F-box/WD repeat-containing protein 10	FBXW10			
Q9NWN3	F-box only protein 34	FBXO34			
P49771	Fms-related tyrosine kinase 3 ligand	FLT3LG			
Q8NFU4	Follicular dendritic cell secreted peptide	FDCSP			
O75949	Transmembrane protein FAM155B	FAM155B			
Q6P4F1	Alpha-	FUT10			
Q9BUM1	Glucose-6-phosphatase 3	G6PC3			
P54803	Galactocerebrosidase	GALC			
Q9UJU5	Forkhead box protein D3	FOXD3			
Q9NQT6	Fascin-3	FSCN3			
Q9Y3Q4	Potassium/sodium hyperpolarization-activated cyclic nucleotide-gated channel 4	HCN4			
P46439	Glutathione S-transferase Mu 5	GSTM5			
Q2KHT4	Germ cell-specific gene 1 protein	GSG1			
Q96A08	Histone H2B type 1-A	H2BC1			
P16403	Histone H1.2	H1-2			
Q9BYQ9	Keratin-associated protein 4-8	KRTAP4-8			
P0C0S8	Histone H2A type 1	H2AC11			
Q7L7L0	Histone H2A type 3	H2AW			
Q7Z2K8	G protein-regulated inducer of neurite outgrowth 1	GPRIN1			
Q96D42	Hepatitis A virus cellular receptor	HAVCR1			
Q96CS2	HAUS augmin-like complex subunit 1	HAUS1			
Q9H2X6	Homeodomain-interacting protein kinase 2	HIPK2			
Q9UII4	E3 ISG15--protein ligase HERC5	HERC5			
Q9NRV9	Heme-binding protein 1	HEBP1			
P83110	Serine protease HTRA3	HTRA3			
Q6NT76	Homeobox-containing protein 1	HMBOX1			
Q96RW7	Hemicentin-1	HMCN1			
O00198	Activator of apoptosis harakiri	HRK			
P0CJ70	Humanin-like 3	MTRNR2L3			
Q15056	Eukaryotic translation initiation factor 4H	EIF4H			
Q96T52	Mitochondrial inner membrane protease subunit 2	IMMP2L			

A6NNB3	Interferon-induced transmembrane protein 5	IFITM5			
P01344	Insulin-like growth factor II	IGF2			
Q8NI17	Interleukin-31 receptor subunit alpha	IL31RA			
Q9UHA7	Interleukin-36 alpha	IL36A			
P29459	Interleukin-12 subunit alpha	IL12A			
P14902	Indoleamine 2,3-dioxygenase 1	IDO1			
P17936	Insulin-like growth factor-binding protein 3	IGFBP3			
Q5VZ66	Janus kinase and microtubule-interacting protein 3	JAKMIP3			
P05107	Integrin beta-2	ITGB2			
Q8IXL9	IQ domain-containing protein F2	IQCF2			
Q6A163	Keratin, type I cytoskeletal 39	KRT39			
O14713	Integrin beta-1-binding protein 1	ITGB1BP1			
Q1W4C9	Serine protease inhibitor Kazal-type 13	SPINK13			
Q86YT9	Junctional adhesion molecule-like	JAML			
Q96P70	Importin-9	IPO9			
P48544	G protein-activated inward rectifier potassium channel 4	KCNJ5			
Q9UKR0	Kallikrein-12	KLK12			
P60329	Keratin-associated protein 12-4	KRTAP12-4			
Q8N6Q8	Methyltransferase-like protein 25	METTL25			
Q8N344	Mesoderm induction early response protein 2	MIER2			
Q9GZY8	Mitochondrial fission factor	MFF			
Q7Z3U7	Protein MON2 homolog	MON2			
Q9H867	Protein-lysine methyltransferase METTL21D	VCPKMT			
Q7L5Y1	Mitochondrial enolase superfamily member 1	ENOSF1			
P19957	Elafin	PI3			
P29322	Ephrin type-A receptor 8	EPHA8			
Q9NU53	Glycoprotein integral membrane protein 1	GINM1			
Q5VTD9	Zinc finger protein Gfi-1b	GFI1B			
Q9UBU3	Appetite-regulating hormone	GHRL			
Q9NP62	Chorion-specific transcription factor GCMa	GCM1			
Q8WXD5	Gem-associated protein 6	GEMIN6			
P15586	N-acetylglucosamine-6-sulfatase	GNS			
Q96IK5	Germ cell-less protein-like 1	GMCL1			
H3BSY2	Golgin subfamily A member 8M	GOLGA8M			
Q8N6U8	G-protein coupled receptor 161	GPR161			
Q99678	G-protein coupled receptor 20	GPR20			
Q96NT3	Protein GUCD1	GUCD1			
Q12849	G-rich sequence factor 1	GRSF1			
Q9UBP9	PTB domain-containing engulfment adapter protein 1	GULP1			
P09210	Glutathione S-transferase A2	GSTA2			
Q9H4Y5	Glutathione S-transferase omega-2	GSTO2			
Q9Y2Q3	Glutathione S-transferase kappa 1	GSTK1			
Q9BSH5	Haloacid dehalogenase-like hydrolase domain-containing protein 3	HDHD3			
Q9H6D7	HAUS augmin-like complex subunit 4	HAUS4			
O75146	Huntingtin-interacting protein 1-related protein	HIP1R			
A0A0C4DH30	Probable non-functional immunoglobulin heavy variable 3-16	IGHV3-16			
A0A0B4J1V6	Immunoglobulin heavy variable 3-73	IGHV3-73			
A6NJ69	IgA-inducing protein homolog	IGIP			

P01877	Immunoglobulin heavy constant alpha 2	IGHA2			
Q8N5X7	Eukaryotic translation initiation factor 4E type 3	EIF4E3			
A1L1A6	Immunoglobulin superfamily member 23	IGSF23			
Q6UXL0	Interleukin-20 receptor subunit beta	IL20RB			
Q9NPF7	Interleukin-23 subunit alpha	IL23A			
Q9UHF4	Interleukin-20 receptor subunit alpha	IL20RA			
P18510	Interleukin-1 receptor antagonist protein	IL1RN			
O14645	Axonemal dynein light intermediate polypeptide 1	DNALI1			
Q9UQF2	C-Jun-amino-terminal kinase-interacting protein 1	MAPK8IP1			
Q15652	Probable JmjC domain-containing histone demethylation protein 2C	JMJD1C			
Q8IYS2	Uncharacterized protein KIAA2013	KIAA2013			
B3KU38	IQCJ-SCHIP1 readthrough transcript protein	IQCJ-SCHIP1			
O60674	Tyrosine-protein kinase JAK2	JAK2			
Q9H9V9	2-oxoglutarate and iron-dependent oxygenase JMJD4	JMJD4			
Q75QN2	Integrator complex subunit 8	INTS8			
Q969F8	KiSS-1 receptor	KISS1R			
P08476	Inhibin beta A chain	INHBA			
P51460	Insulin-like 3	INSL3			
Q96T92	Insulinoma-associated protein 2	INSM2			
Q96CT2	Kelch-like protein 29	KLHL29			
Q9Y4H2	Insulin receptor substrate 2	IRS2			
O00522	Krev interaction trapped protein 1	KRIT1			
Q9BYS1	Keratin-associated protein 1-5	KRTAP1-5			
Q693B1	BTB/POZ domain-containing protein KCTD11	KCTD11			
Q96SI1	BTB/POZ domain-containing protein KCTD15	KCTD15			
Q68CQ1	Maestro heat-like repeat-containing protein family member 7	MROH7			
P23352	Anosmin-1	ANOS1			
Q9P0J7	E3 ubiquitin-protein ligase KCMF1	KCMF1			
Q8TAF5	Putative uncharacterized protein LQK1	FLVCR1-DT			
Q9NPC1	Leukotriene B4 receptor 2	LTB4R2			
Q14210	Lymphocyte antigen 6D	LY6D			
Q6IAA8	Ragulator complex protein LAMTOR1	LAMTOR1			
Q8NHL6	Leukocyte immunoglobulin-like receptor subfamily B member 1	LILRB1			
Q6PI73	Leukocyte immunoglobulin-like receptor subfamily A member 6	LILRA6			
Q6ZMQ8	Serine/threonine-protein kinase LMTK1	AATK			
Q6UWN0	Ly6/PLAUR domain-containing protein 4	LYPD4			
O14770	Homeobox protein Meis2	MEIS2			
Q96JQ5	Membrane-spanning 4-domains subfamily A member 4A	MS4A4A			
Q96HR3	Mediator of RNA polymerase II transcription subunit 30	MED30			
A6NDR6	Putative homeobox protein Meis3-like 1	MEIS3P1			
Q96JA4	Membrane-spanning 4-domains subfamily A member 14	MS4A14			
P08183	ATP-dependent translocase ABCB1	ABCB1			

Q6PJG2	Mitotic deacetylase-associated SANT domain protein	MIDEAS			
Q96T53	Ghrelin O-acyltransferase	MBOAT4			
Q9UBF1	Melanoma-associated antigen C2	MAGEC2			
P48740	Mannan-binding lectin serine protease 1	MASP1			
Q8WWC4	m-AAA protease-interacting protein 1, mitochondrial	MAIP1			
Q14696	LRP chaperone MESD	MESD			
Q9H8M7	Ubiquitin carboxyl-terminal hydrolase MINDY-3	MINDY3			
Q969V6	Myocardin-related transcription factor A	MRTFA			
Q9P0P8	Mitochondrial transcription rescue factor 1	MTRES1			
Q13613	Myotubularin-related protein 1	MTMR1			
P49914	5-formyltetrahydrofolate cyclo-ligase	MTHFS			
P42285	Exosome RNA helicase MTR4	MTREX			
Q9UKN1	Mucin-12	MUC12			
Q8N684	Cleavage and polyadenylation specificity factor subunit 7	CPSF7			
P57737	Coronin-7	CORO7			
Q6JBY9	CapZ-interacting protein	RCSD1			
Q14118	Dystroglycan	DAG1			
Q6IQ26	DENN domain-containing protein 5A	DENND5A			
Q9UMR2	ATP-dependent RNA helicase DDX19B	DDX19B			
P19113	Histidine decarboxylase	HDC			
Q96HY6	DDRGK domain-containing protein 1	DDRGK1			
Q92841	Probable ATP-dependent RNA helicase DDX17	DDX17			
Q5VTH9	Dynein intermediate chain 4, axonemal	DNAI4			
Q9Y222	Cyclin-D-binding Myb-like transcription factor 1	DMTF1			
P49366	Deoxyhypusine synthase	DHPS			
Q96M86	Dynein heavy chain domain-containing protein 1	DNHD1			
O60884	DnaJ homolog subfamily A member 2	DNAJA2			
Q8WW22	DnaJ homolog subfamily A member 4	DNAJA4			
Q9UBC3	DNA	DNMT3B			
P13765	HLA class II histocompatibility antigen, DO beta chain	HLA-DOB			
Q8NF50	Dedicator of cytokinesis protein 8	DOCK8			
Q9BZ29	Dedicator of cytokinesis protein 9	DOCK9			
Q9Y2E4	Disco-interacting protein 2 homolog C	DIP2C			
Q07687	Homeobox protein DLX-2	DLX2			
Q9NR33	DNA polymerase epsilon subunit	POLE4			
Q8NBI3	Draxin	DRAXIN			
Q9NP87	DNA-directed DNA/RNA polymerase mu	POLM			
O75822	Eukaryotic translation initiation factor 3 subunit J	EIF3J			
P54851	Epithelial membrane protein 2	EMP2			
P49770	Translation initiation factor eIF-2B subunit beta	EIF2B2			
Q96EB1	Elongator complex protein 4	ELP4			
P15976	Erythroid transcription factor	GATA1			
O60353	Frizzled-6	FZD6			
P04062	Lysosomal acid glucosylceramidase	GBA			
Q6ZNW5	GDP-D-glucose phosphorylase 1	GDPGP1			
Q9BZE0	Zinc finger protein GLIS2	GLIS2			

P62879	Guanine nucleotide-binding protein G	GNB2			
Q9H0R8	Gamma-aminobutyric acid receptor-associated protein-like 1	GABARAPL1			
Q96CN9	GRIP and coiled-coil domain-containing protein 1	GCC1			
P36915	Guanine nucleotide-binding protein-like 1	GNL1			
P48169	Gamma-aminobutyric acid receptor subunit alpha-4	GABRA4			
Q14409	Glycerol kinase 3	GK3P			
Q5JWF2	Guanine nucleotide-binding protein G	GNAS			
Q9BXC1	Probable G-protein coupled receptor 174	GPR174			
Q6DWJ6	Probable G-protein coupled receptor 139	GPR139			
Q8TBA6	Golgin subfamily A member 5	GOLGA5			
P78347	General transcription factor II-I	GTF2I			
Q8TAA5	GrpE protein homolog 2, mitochondrial	GRPEL2			
Q96HH9	GRAM domain-containing protein 2B	GRAMD2B			
Q701N4	Keratin-associated protein 5-2	KRTAP5-2			
Q7Z4V5	Hepatoma-derived growth factor-related protein 2	HDGFL2			
P36551	Oxygen-dependent coproporphyrinogen-III oxidase, mitochondrial	CPOX			
Q02575	Helix-loop-helix protein 1	NHLH1			
Q96QV1	Hedgehog-interacting protein	HHIP			
B2RPK0	Putative high mobility group protein B1-like 1	HMGB1P1			
Q9Y4D8	Probable E3 ubiquitin-protein ligase HECTD4	HECTD4			
P31270	Homeobox protein Hox-A11	HOXA11			
P28358	Homeobox protein Hox-D10	HOXD10			
P01768	Immunoglobulin heavy variable 3-30	IGHV3-30			
A0A0B4J1V1	Immunoglobulin heavy variable 3-21	IGHV3-21			
A0A075B6R2	Immunoglobulin heavy variable 4-4	IGHV4-4			
Q9NP08	Homeobox protein HMX1	HMX1			
P34931	Heat shock 70 kDa protein 1-like	HSPA1L			
P07910	Heterogeneous nuclear ribonucleoproteins C1/C2	HNRNPC			
Q96PD4	Interleukin-17F	IL17F			
Q9Y6M1	Insulin-like growth factor 2 mRNA-binding protein 2	IGF2BP2			
Q9UBC1	NF-kappa-B inhibitor-like protein 1	NFKBIL1			
Q9NPH9	Interleukin-26	IL26			
P35225	Interleukin-13	IL13			
O75054	Immunoglobulin superfamily member 3	IGSF3			
P22301	Interleukin-10	IL10			
Q9H665	IGF-like family receptor 1	IGFLR1			
P24593	Insulin-like growth factor-binding protein 5	IGFBP5			
Q9H1B7	Probable E3 ubiquitin-protein ligase IRF2BPL	IRF2BPL			
Q6UXV1	Izumo sperm-egg fusion protein 2	IZUMO2			
P02538	Keratin, type II cytoskeletal 6A	KRT6A			
P13647	Keratin, type II cytoskeletal 5	KRT5			
Q68E01	Integrator complex subunit 3	INTS3			
Q15058	Kinesin-like protein KIF14	KIF14			
Q8IXV7	Kelch domain-containing protein 8B	KLHDC8B			

P48549	G protein-activated inward rectifier potassium channel 1	KCNJ3			
A8MYU2	Potassium channel subfamily U member 1	KCNU1			
Q7Z4W3	Keratin-associated protein 19-3	KRTAP19-3			
A8MX34	Keratin-associated protein 29-1	KRTAP29-1			
P41229	Lysine-specific demethylase 5C	KDM5C			
Q9BQ66	Keratin-associated protein 4-12	KRTAP4-12			
Q12791	Calcium-activated potassium channel subunit alpha-1	KCNMA1			
Q9BQE6	LBH domain-containing protein 1	LBHD1			
O00515	Ladinin-1	LAD1			
Q8NC54	Keratinocyte-associated transmembrane protein 2	KCT2			
Q52LA3	Protein lin-52 homolog	LIN52			
O95278	Laforin	EPM2A			
Q96A84	EMI domain-containing protein 1	EMID1			
P51114	Fragile X mental retardation syndrome-related protein 1	FXR1			
Q9UF56	F-box/LRR-repeat protein 17	FBXL17			
Q12950	Forkhead box protein D4	FOXD4			
Q96CU9	FAD-dependent oxidoreductase domain-containing protein 1	FOXRED1			
P32314	Forkhead box protein N2	FOXN2			
P15407	Fos-related antigen 1	FOSL1			
Q8IVV7	Glucose-induced degradation protein 4 homolog	GID4			
E5RQL4	Formiminotransferase N-terminal subdomain-containing protein	FTCDNL1			
P08151	Zinc finger protein GLI1	GLI1			
Q9NXN4	Ganglioside-induced differentiation-associated protein 2	GDAP2			
P63211	Guanine nucleotide-binding protein G	GNGT1			
Q9H0R5	Guanylate-binding protein 3	GBP3			
Q92947	Glutaryl-CoA dehydrogenase, mitochondrial	GCDH			
P31644	Gamma-aminobutyric acid receptor subunit alpha-5	GABRA5			
Q96P66	Probable G-protein coupled receptor 101	GPR101			
Q9H2G9	Golgin-45	BLZF1			
Q8TED1	Probable glutathione peroxidase 8	GPX8			
X6R8D5	Putative uncharacterized protein GUCA1ANB	GUCA1ANB			
Q8TD20	Solute carrier family 2, facilitated glucose transporter member 12	SLC2A12			
P14317	Hematopoietic lineage cell-specific protein	HCLS1			
Q9NRM0	Solute carrier family 2, facilitated glucose transporter member 9	SLC2A9			
O15303	Metabotropic glutamate receptor 6	GRM6			
Q9HCP6	Protein-cysteine N-palmitoyltransferase HHAT-like protein	HHATL			
Q15751	Probable E3 ubiquitin-protein ligase HERC1	HERC1			
P04233	HLA class II histocompatibility antigen gamma chain	CD74			
P48723	Heat shock 70 kDa protein 13	HSPA13			
P04553	Sperm protamine P1	PRM1			
P0DP07	Immunoglobulin heavy variable 4-31	IGHV4-31			
A0A087WSY4	Immunoglobulin heavy variable 4-30-2	IGHV4-30-2			
A0A0C4DH42	Immunoglobulin heavy variable 3-66	IGHV3-66			

P0C7T4	Minor histocompatibility protein HMSD variant form	HMSD			
Q8NCD3	Holliday junction recognition protein	HJURP			
Q8IZT8	Heparan sulfate glucosamine 3-O-sulfotransferase 5	HS3ST5			
Q14527	Helicase-like transcription factor	HLTF			
P0CJ68	Humanin-like 1	MTRNR2L1			
Q9P2W1	Homologous-pairing protein 2 homolog	PSMC3IP			
Q9BYX4	Interferon-induced helicase C domain-containing protein 1	IFIH1			
Q12906	Interleukin enhancer-binding factor 3	ILF3			
Q9NV31	U3 small nucleolar ribonucleoprotein protein IMP3	IMP3			
Q96FT9	Intraflagellar transport protein 43 homolog	IFT43			
Q14213	Interleukin-27 subunit beta	EBI3			
Q13907	Isopentenyl-diphosphate Delta-isomerase 1	IDI1			
Q7Z4Q2	HEAT repeat-containing protein 3	HEATR3			
Q9P2H3	Intraflagellar transport protein 80 homolog	IFT80			
P52292	Importin subunit alpha-1	KPNA2			
Q96F46	Interleukin-17 receptor A	IL17RA			
Q6NSI8	Uncharacterized protein KIAA1841	KIAA1841			
Q5TCS8	Adenylate kinase 9	AK9			
Q9NV88	Integrator complex subunit 9	INTS9			
Q14003	Potassium voltage-gated channel subfamily C member 3	KCNC3			
P10721	Mast/stem cell growth factor receptor Kit	KIT			
Q9UIL4	Kinesin-like protein KIF25	KIF25			
Q3LI62	Putative keratin-associated protein 20-4	KRTAP20-4			
Q9NS61	Kv channel-interacting protein 2	KCNIP2			
Q9BQ13	BTB/POZ domain-containing protein KCTD14	KCTD14			
Q9P2E2	Kinesin-like protein KIF17	KIF17			
Q9BYR3	Keratin-associated protein 4-4	KRTAP4-4			
Q9BYR9	Keratin-associated protein 2-4	KRTAP2-4			
Q8NFY9	Kelch repeat and BTB domain-containing protein 8	KBTBD8			
Q9HAP6	Protein lin-7 homolog B	LIN7B			
Q6JVE5	Epididymal-specific lipocalin-12	LCN12			
Q8IV20	Purine nucleoside phosphorylase LACC1	LACC1			
Q9NRM7	Serine/threonine-protein kinase LATS2	LATS2			
Q5T5B0	Late cornified envelope protein 3E	LCE3E			
P41159	Leptin	LEP			
Q2I0M4	Leucine-rich repeat-containing protein 26	LRRC26			
Q8N4P6	Leucine-rich repeat-containing protein 71	LRRC71			
B2RXH2	Lysine-specific demethylase 4E	KDM4E			
O75096	Low-density lipoprotein receptor-related protein 4	LRP4			
Q14114	Low-density lipoprotein receptor-related protein 8	LRP8			
Q9ULH4	Leucine-rich repeat and fibronectin type-III domain-containing protein 2	LRFN2			
A4D1F6	Leucine-rich repeat and death domain-containing protein 1	LRRD1			
Q32MZ4	Leucine-rich repeat flightless-interacting protein 1	LRRFIP1			

Q5S007	Leucine-rich repeat serine/threonine-protein kinase 2	LRRK2			
Q643R3	Lysophospholipid acyltransferase LPCAT4	LPCAT4			
Q9Y6Y9	Lymphocyte antigen 96	LY96			
O95868	Lymphocyte antigen 6 complex locus protein G6d	LY6G6D			
Q96Q04	Serine/threonine-protein kinase LMTK3	LMTK3			
Q00325	Phosphate carrier protein, mitochondrial	SLC25A3			
P08118	Beta-microseminoprotein	MSMB			
Q13496	Myotubularin	MTM1			
P04732	Metallothionein-1E	MT1E			
Q7Z5P9	Mucin-19	MUC19			
Q7L1T6	Cytochrome b5 reductase 4	CYB5R4			
Q9Y3Q0	N-acetylated-alpha-linked acidic dipeptidase 2	NAALAD2			
P54849	Epithelial membrane protein 1	EMP1			
P15502	Elastin	ELN			
Q03828	Homeobox even-skipped homolog protein 2	EVX2			
Q05BU3	Putative protein FAM86JP	FAM86JP			
P24390	ER lumen protein-retaining receptor 1	KDELR1			
Q4G0M1	Erythroferrone	ERFE			
Q4VXF1	Putative protein FAM74A3	FAM74A3			
Q6ZV65	Protein FAM47E	FAM47E			
O14944	Proepiregulin	EREG			
P41212	Transcription factor ETV6	ETV6			
Q9NYF3	Protein FAM53C	FAM53C			
Q9NVM1	Protein eva-1 homolog B	EVA1B			
Q96BQ1	Protein FAM3D	FAM3D			
O00757	Fructose-1,6-bisphosphatase isozyme 2	FBP2			
Q8NEG4	Protein FAM83F	FAM83F			
Q9BQN1	Protein FAM83C	FAM83C			
A0FGR9	Extended synaptotagmin-3	ESYT3			
A6NE01	Protein FAM186A	FAM186A			
Q1W6H9	Protein FAM110C	FAM110C			
Q9NTX9	Protein FAM217B	FAM217B			
Q9H8W3	Protein FAM204A	FAM204A			
Q8NHP7	piRNA biogenesis protein EXD1	EXD1			
O95208	Epsin-2	EPN2			
A6NKX1	Protein FAM223B	FAM223B			
Q96GL9	Protein FAM163A	FAM163A			
Q96KS9	Protein FAM167A	FAM167A			
Q9BTA0	Protein FAM167B	FAM167B			
Q92800	Histone-lysine N-methyltransferase EZH1	EZH1			
P0DSO1	Protein FAM246C	FAM246C			
Q96HJ9	Protein FMC1 homolog	FMC1			
Q8IVF7	Formin-like protein 3	FMNL3			
Q99504	Eyes absent homolog 3	EYA3			
Q9NWM8	Peptidyl-prolyl cis-trans isomerase FKBP14	FKBP14			
O75344	Inactive peptidyl-prolyl cis-trans isomerase FKBP6	FKBP6			
O94952	F-box only protein 21	FBXO21			
Q969H0	F-box/WD repeat-containing protein 7	FBXW7			
Q92562	Polyphosphoinositide	FIG4			
Q6ZNL6	FYVE, RhoGEF and PH domain-containing protein 5	FGD5			
Q92915	Fibroblast growth factor 14	FGF14			
P09958	Furin	FURIN			
Q9BWH2	FUN14 domain-containing protein 2	FUNDC2			
Q9BZ68	Putative FERM domain-containing protein FRMD8P1	FRMD8P1			

Q9BYC5	Alpha-	FUT8			
Q6P050	F-box and leucine-rich protein 22	FBXL22			
Q13066	G antigen 2B/2C	GAGE2B			
Q9UKT6	Putative F-box/LRR-repeat protein 21	FBXL21P			
P63126	Endogenous retrovirus group K member 9 Gag polyprotein	ERVK-9			
Q12947	Forkhead box protein F2	FOXF2			
P23769	Endothelial transcription factor GATA-2	GATA2			
Q9NPG1	Frizzled-3	FZD3			
O43559	Fibroblast growth factor receptor substrate 3	FRS3			
Q8WWB7	Glycosylated lysosomal membrane protein	GLMP			
P23378	Glycine dehydrogenase	GLDC			
Q8ND71	GTPase IMAP family member 8	GIMAP8			
Q7Z4J2	Putative glycosyltransferase 6 domain-containing protein 1	GLT6D1			
Q99819	Rho GDP-dissociation inhibitor 3	ARHGDIG			
P33402	Guanylate cyclase soluble subunit alpha-2	GUCY1A2			
Q14642	Inositol polyphosphate-5-phosphatase A	INPP5A			
O00358	Forkhead box protein E1	FOXE1			
Q13461	Forkhead box protein E3	FOXE3			
A6NMD2	Golgin subfamily A member 8J	GOLGA8J			
Q5T7V8	RAB6-interacting golgin	GORAB			
O00461	Golgi integral membrane protein	GOLIM4			
Q9UHW5	GPN-loop GTPase 3	GPN3			
Q9UPC5	Probable G-protein coupled receptor 34	GPR34			
Q9BY41	Histone deacetylase 8	HDAC8			
Q9P0M6	Core histone macro-H2A.2	MACROH2A2			
Q4G0G2	Putative uncharacterized protein H1-10-AS1	H1-10-AS1			
P81172	Hepcidin	HAMP			
P00738	Haptoglobin	HP			
Q86YV9	Hermansky-Pudlak syndrome 6 protein	HPS6			
P35452	Homeobox protein Hox-D12	HOXD12			
O43719	HIV Tat-specific factor 1	HTATSF1			
Q8NHY5	Checkpoint protein HUS1B	HUS1B			
Q16082	Heat shock protein beta-2	HSPB2			
Q9NP66	High mobility group protein 20A	HMG20A			
Q9NSC5	Homer protein homolog 3	HOMER3			
Q96S86	Hyaluronan and proteoglycan link protein 3	HAPLN3			
Q01628	Interferon-induced transmembrane protein 3	IFITM3			
O75330	Hyaluronan mediated motility receptor	HMMR			
Q8IY31	Intraflagellar transport protein 20 homolog	IFT20			
Q9UHD0	Interleukin-19	IL19			
A0AVF1	Intraflagellar transport protein 56	TTC26			
P25963	NF-kappa-B inhibitor alpha	NFKBIA			
P08700	Interleukin-3	IL3			
P22304	Iduronate 2-sulfatase	IDS			
Q9Y6W8	Inducible T-cell costimulator	ICOS			
Q96ID5	Immunoglobulin superfamily member 21	IGSF21			
Q8N0W5	IQ domain-containing protein K	IQCK			
Q8WWA0	Intelectin-1	ITLN1			
Q14CN4	Keratin, type II cytoskeletal 72	KRT72			
Q9ULL0	Acrosomal protein KIAA1210	KIAA1210			
Q9NQS7	Inner centromere protein	INCENP			
Q96PE3	Inositol polyphosphate-4-phosphatase type I A	INPP4A			

Q9H0H3	Kelch-like protein 25	KLHL25			
Q9NPH2	Inositol-3-phosphate synthase 1	ISYNA1			
P78414	Iroquois-class homeodomain protein IRX-1	IRX1			
Q8IYD2	Kelch domain-containing protein 8A	KLHDC8A			
O15066	Kinesin-like protein KIF3B	KIF3B			
Q14722	Voltage-gated potassium channel subunit beta-1	KCNAB1			
P43628	Killer cell immunoglobulin-like receptor 2DL3	KIR2DL3			
Q92831	Histone acetyltransferase KAT2B	KAT2B			
Q9BR39	Junctophilin-2	JPH2			
Q5HYC2	Uncharacterized protein KIAA2026	KIAA2026			
P13645	Keratin, type I cytoskeletal 10	KRT10			
Q9Y6F6	Inositol 1,4,5-triphosphate receptor associated 1	IRAG1			
B1AKI9	Isthmin-1	ISM1			
Q1A5X6	IQ domain-containing protein J	IQCJ			
Q5XKE5	Keratin, type II cytoskeletal 79	KRT79			
O95373	Importin-7	IPO7			
P14316	Interferon regulatory factor 2	IRF2			
Q5JSQ8	Putative KHDC1-like protein	KHDC1L			
Q9UJ90	Potassium voltage-gated channel subfamily E regulatory beta subunit 5	KCNE5			
Q14954	Killer cell immunoglobulin-like receptor 2DS1	KIR2DS1			
Q96S38	Ribosomal protein S6 kinase delta-1	RPS6KC1			
O95251	Histone acetyltransferase KAT7	KAT7			
Q9BYQ2	Keratin-associated protein 9-4	KRTAP9-4			
Q86U70	LIM domain-binding protein 1	LDB1			
P18428	Lipopolysaccharide-binding protein	LBP			
Q9BYQ0	Keratin-associated protein 9-8	KRTAP9-8			
Q3B8N2	Galectin-9B	LGALS9B			
Q96PB8	Leucine-rich repeat-containing protein 3B	LRRC3B			
O75427	Leucine-rich repeat and calponin homology domain-containing protein 4	LRCH4			
Q3SXY7	Leucine-rich repeat, immunoglobulin-like domain and transmembrane domain-containing protein 3	LRIT3			
P09848	Lactase-phlorizin hydrolase	LCT			
Q6UX01	Protein LMBR1L	LMBR1L			
P11150	Hepatic triacylglycerol lipase	LIPC			
A0A075B6J2	Probable non-functional immunoglobulin lambda variable 2-33	IGLV2-33			
P04211	Immunoglobulin lambda variable 7-43	IGLV7-43			
O43148	mRNA cap guanine-N7 methyltransferase	RNMT			
Q99705	Melanin-concentrating hormone receptor 1	MCHR1			
Q99683	Mitogen-activated protein kinase kinase kinase 5	MAP3K5			
Q86UD3	E3 ubiquitin-protein ligase MARCHF3	MARCHF3			
Q96M61	Melanoma-associated antigen B18	MAGEB18			
O75586	Mediator of RNA polymerase II transcription subunit 6	MED6			
Q3KP22	Membrane-anchored junction protein	MAJIN			

A0A1B0GVZ6	Methyl-CpG-binding domain protein 3-like 2B	MBD3L2B			
Q7L5Y9	E3 ubiquitin-protein transferase MAEA	MAEA			
Q8TC57	Meiosis 1 arrest protein	M1AP			
O75951	Lysozyme-like protein 6	LYZL6			
Q9ULX9	Transcription factor MafF	MAFF			
P43360	Melanoma-associated antigen 6	MAGEA6			
O15481	Melanoma-associated antigen B4	MAGEB4			
Q9UPY8	Microtubule-associated protein RP/EB family member 3	MAPRE3			
O76050	E3 ubiquitin-protein ligase NEURL1	NEURL1			
Q9BR09	Neuralized-like protein 2	NEURL2			
Q96EH8	E3 ubiquitin-protein ligase NEURL3	NEURL3			
P51843	Nuclear receptor subfamily 0 group B member 1	NR0B1			
P49279	Natural resistance-associated macrophage protein 1	SLC11A1			
O60500	Nephrin	NPHS1			
Q9BYT8	Neurolysin, mitochondrial	NLN			
O75200	Nuclear pore complex-interacting protein family member B7	NPIPB7			
Q75NE6	Putative microRNA 17 host gene protein	MIR17HG			
P03971	Muellerian-inhibiting factor	AMH			
Q641Q3	Meteorin-like protein	METRNL			
P16455	Methylated-DNA--protein-cysteine methyltransferase	MGMT			
Q9UL63	Muskelin	MKLN1			
Q9HCE5	N6-adenosine-methyltransferase non-catalytic subunit	METTL14			
Q9Y5R2	Matrix metalloproteinase-24	MMP24			
P08581	Hepatocyte growth factor receptor	MET			
Q6ZSS7	Major facilitator superfamily domain-containing protein 6	MFSD6			
O43451	Maltase-glucoamylase, intestinal	MGAM			
Q15777	Metallophosphoesterase MPPED2	MPPED2			
P0DKB6	Mitochondrial pyruvate carrier 1-like protein	MPC1L			
Q15014	Mortality factor 4-like protein 2	MORF4L2			
Q9UJG1	Motile sperm domain-containing protein 1	MOSPD1			
Q99547	M-phase phosphoprotein 6	MPHOSPH			
Q86WG5	Myotubularin-related protein 13	SBF2			
Q9BYG7	Protein maestro	MRO			
Q86U44	N6-adenosine-methyltransferase catalytic subunit	METTL3			
Q15746	Myosin light chain kinase, smooth muscle	MYLK			
P12525	Putative myc-like protein MYCLP1	MYCLP1			
O60361	Putative nucleoside diphosphate kinase	NME2P1			
O00160	Unconventional myosin-If	MYO1F			
Q9UM54	Unconventional myosin-VI	MYO6			
P16435	NADPH--cytochrome P450 reductase	POR			
A0A087WUL8	Neuroblastoma breakpoint family member 19	NBPF19			
P13535	Myosin-8	MYH8			
Q8NCQ3	Putative uncharacterized protein encoded by LINC00301	LINC00301			
Q9NPP4	NLR family CARD domain-containing protein 4	NLRC4			
P0C0P6	Neuropeptide S	NPS			
Q9NX14	NADH dehydrogenase	NDUFB11			
Q8NG41	Neuropeptide B	NPB			

O95139	NADH dehydrogenase	NDUFB6			
O00590	Atypical chemokine receptor 2	ACKR2			
O94929	Actin-binding LIM protein 3	ABLIM3			
P02760	Protein AMBP	AMBP			
O14862	Interferon-inducible protein AIM2	AIM2			
Q9UKB5	Adherens junction-associated protein 1	AJAP1			
P05091	Aldehyde dehydrogenase, mitochondrial	ALDH2			
O43823	A-kinase anchor protein 8	AKAP8			
Q9UL18	Protein argonaute-1	AGO1			
O95394	Phosphoacetylglucosamine mutase	PGM3			
Q5VUY0	Arylacetamide deacetylase-like 3	AADACL3			
P48960	Adhesion G protein-coupled receptor E5	ADGRE5			
Q9UKU0	Long-chain-fatty-acid--CoA ligase 6	ACSL6			
O00253	Agouti-related protein	AGRP			
P63261	Actin, cytoplasmic 2	ACTG1			
Q13015	Protein AF1q	MLLT11			
O43506	Disintegrin and metalloproteinase domain-containing protein 20	ADAM20			
Q9HBW9	Adhesion G protein-coupled receptor L4	ADGRL4			
Q8IUX7	Adipocyte enhancer-binding protein 1	AEBP1			
Q6IWH7	Anoctamin-7	ANO7			
Q8N9V6	Ankyrin repeat domain-containing protein 53	ANKRD53			
Q8IVW6	AT-rich interactive domain-containing protein 3B	ARID3B			
Q49B96	Cytochrome c oxidase assembly protein COX19	COX19			
Q8NI60	Atypical kinase COQ8A, mitochondrial	COQ8A			
Q86YQ8	Copine-8	CPNE8			
P49747	Cartilage oligomeric matrix	COMP			
Q9Y6A2	Cholesterol 24-hydroxylase	CYP46A1			
P52943	Cysteine-rich protein 2	CRIP2			
Q16850	Lanosterol 14-alpha demethylase	CYP51A1			
Q8N1N5	Putative protein CRIPAK	CRIPAK			
A5YM72	Carnosine synthase 1	CARNS1			
P58512	Uncharacterized protein encoded by LINC01547	LINC01547			
Q2VPK5	Cytoplasmic tRNA 2-thiolation protein 2	CTU2			
Q4LDR2	Cortexin-3	CTXN3			
P59037	Putative uncharacterized protein encoded by LINC00313	LINC00313			
Q8WYA6	Beta-catenin-like protein 1	CTNNBL1			
P35221	Catenin alpha-1	CTNNA1			
Q86UF2	cTAGE family member 6	CTAGE6			
Q15438	Cytohesin-1	CYTH1			
Q9H2H0	CXXC-type zinc finger protein 4	CXXC4			
P59861	Beta-defensin 131A	DEFB131A			
O75064	DENN domain-containing protein 4B	DENND4B			
P53355	Death-associated protein kinase 1	DAPK1			
Q9UIK4	Death-associated protein kinase 2	DAPK2			
Q96NX9	Dachshund homolog 2	DACH2			
Q92564	DCN1-like protein 4	DCUN1D4			
Q8IXT1	DNA damage-induced apoptosis suppressor protein	DDIAS			
Q8TDD1	ATP-dependent RNA helicase DDX54	DDX54			
A6NGE4	DDB1- and CUL4-associated factor 8-like protein 1	DCAF8L1			

Q5H9U9	Probable ATP-dependent RNA helicase DDX60-like	DDX60L			
Q8WV16	DDB1- and CUL4-associated factor 4	DCAF4			
Q15392	Delta	DHCR24			
Q9NZJ0	Denticleless protein homolog	DTL			
Q8WTR2	Dual specificity protein phosphatase 19	DUSP19			
Q96F86	Enhancer of mRNA-decapping protein 3	EDC3			
Q69384	Endogenous retrovirus group K member 6 Env polyprotein	ERVK-6			
Q9BV81	ER membrane protein complex subunit 6	EMC6			
P43026	Growth/differentiation factor 5	GDF5			
P31150	Rab GDP dissociation inhibitor alpha	GDI1			
P50395	Rab GDP dissociation inhibitor beta	GDI2			
O95395	Beta-1,3-galactosyl-O-glycosyl-glycoprotein beta-1,6-N-acetylglucosaminyltransferase 3	GCNT3			
Q9BX10	GTP-binding protein 2	GTPBP2			
P49841	Glycogen synthase kinase-3 beta	GSK3B			
Q14831	Metabotropic glutamate receptor 7	GRM7			
Q9NVX0	HAUS augmin-like complex subunit 2	HAUS2			
Q96HZ4	Transcription cofactor HES-6	HES6			
P20155	Serine protease inhibitor Kazal-type 2	SPINK2			
Q6DN90	IQ motif and SEC7 domain-containing protein 1	IQSEC1			
Q96ST2	Protein IWS1 homolog	IWS1			
Q08881	Tyrosine-protein kinase ITK/TSK	ITK			
O95965	Integrin beta-like protein 1	ITGBL1			
Q9UL03	Integrator complex subunit 6	INTS6			
Q9H2U2	Inorganic pyrophosphatase 2, mitochondrial	PPA2			
Q9Y6H6	Potassium voltage-gated channel subfamily E member 3	KCNE3			
O95600	Krueppel-like factor 8	KLF8			
O95239	Chromosome-associated kinesin KIF4A	KIF4A			
P60014	Keratin-associated protein 10-10	KRTAP10-10			
A0A087WTH5	Potassium voltage-gated channel subfamily E member 1B	KCNE1B			
P56696	Potassium voltage-gated channel subfamily KQT member 4	KCNQ4			
O60282	Kinesin heavy chain isoform 5C	KIF5C			
Q16773	Kynurenine--oxoglutarate transaminase 1	KYAT1			
Q9BYQ6	Keratin-associated protein 4-11	KRTAP4-11			
Q92743	Serine protease HTRA1	HTRA1			
Q9P0W2	SWI/SNF-related matrix-associated actin-dependent regulator of chromatin subfamily E member 1-related	HMG20B			
O75031	Heat shock factor 2-binding protein	HSF2BP			
Q05925	Homeobox protein engrailed-1	EN1			
Q95460	Major histocompatibility complex class I-related gene protein	MR1			
P31943	Heterogeneous nuclear ribonucleoprotein H	HNRNPH1			
Q3SXM5	Inactive hydroxysteroid dehydrogenase-like protein 1	HSDL1			

P10915	Hyaluronan and proteoglycan link protein 1	HAPLN1			
Q86YZ3	Hornerin	HRNR			
Q6K0P9	Pyrin and HIN domain-containing protein 1	PYHIN1			
P0CJ75	Humanin-like 8	MTRNR2L8			
P09601	Heme oxygenase 1	HMOX1			
P01566	Interferon alpha-10	IFNA10			
Q96M15	Putative uncharacterized protein IGF2BP2-AS1	IGF2BP2-AS1			
Q01113	Interleukin-9 receptor	IL9R			
P31785	Cytokine receptor common subunit gamma	IL2RG			
Q6ZVW7	Putative interleukin-17 receptor E-like	IL17REL			
P18065	Insulin-like growth factor-binding protein 2	IGFBP2			
Q14602	Putative DNA-binding protein inhibitor ID-2B	ID2B			
O43301	Heat shock 70 kDa protein 12A	HSPA12A			
Q8TD10	Mirror-image polydactyly gene 1 protein	MIPOL1			
Q9H0C8	Integrin-linked kinase-associated serine/threonine phosphatase 2C	ILKAP			
P28356	Homeobox protein Hox-D9	HOXD9			
P51553	Isocitrate dehydrogenase	IDH3G			
Q9BUN1	Protein MENT	MENT			
O60268	Uncharacterized protein KIAA0513	KIAA0513			
Q6PHW0	Iodotyrosine deiodinase 1	IYD			
P35900	Keratin, type I cytoskeletal 20	KRT20			
O43736	Integral membrane protein 2A	ITM2A			
P48200	Iron-responsive element-binding protein 2	IREB2			
O95678	Keratin, type II cytoskeletal 75	KRT75			
Q8NI77	Kinesin-like protein KIF18A	KIF18A			
Q99706	Killer cell immunoglobulin-like receptor 2DL4	KIR2DL4			
Q9Y2K7	Lysine-specific demethylase 2A	KDM2A			
Q8N9T8	Protein KRI1 homolog	KRI1			
Q14952	Killer cell immunoglobulin-like receptor 2DS3	KIR2DS3			
Q8IU85	Calcium/calmodulin-dependent protein kinase type 1D	CAMK1D			
P03952	Plasma kallikrein	KLKB1			
Q86UP2	Kinectin	KTN1			
Q3LI72	Keratin-associated protein 19-5	KRTAP19-5			
Q9BYT5	Keratin-associated protein 2-2	KRTAP2-2			
O14686	Histone-lysine N-methyltransferase 2D	KMT2D			
A6NM11	Leucine-rich repeat-containing protein 37A2	LRRC37A2			
Q6ZUX7	LHFPL tetraspan subfamily member 2 protein	LHFPL2			
Q9BYE3	Late cornified envelope protein	LCE3D			
Q1L5Z9	LON peptidase N-terminal domain and RING finger protein 2	LONRF2			
Q0VAA2	Leucine-rich repeat-containing protein 74A	LRRC74A			
Q96CX6	Leucine-rich repeat-containing protein 58	LRRC58			
Q15722	Leukotriene B4 receptor 1	LTB4R			
Q14766	Latent-transforming growth factor beta-binding protein 1	LTBP1			
Q6UXK5	Leucine-rich repeat neuronal protein 1	LRRN1			
Q9HBG7	T-lymphocyte surface antigen Ly-9	LY9			
P29376	Leukocyte tyrosine kinase receptor	LTK			
P54317	Pancreatic lipase-related protein 2	PNLIPRP2			

P29536	Leiomodin-1	LMOD1			
Q92539	Phosphatidate phosphatase LPIN2	LPIN2			
P01229	Lutropin subunit beta	LHB			
Q8WWI1	LIM domain only protein 7	LMO7			
Q96RN5	Mediator of RNA polymerase II transcription subunit 15	MED15			
A0JLT2	Mediator of RNA polymerase II transcription subunit 19	MED19			
Q96KG7	Multiple epidermal growth factor-like domains protein 10	MEGF10			
O95460	Matrilin-4	MATN4			
Q9UGB7	Inositol oxygenase	MIOX			
Q9H2W2	Homeobox protein MIXL1	MIXL1			
O00566	U3 small nucleolar ribonucleoprotein protein MPP10	MPHOSPH 10			
Q7L1V2	Vacuolar fusion protein MON1 homolog B	MON1B			
Q9NYP9	Protein Mis18-alpha	MIS18A			
Q13163	Dual specificity mitogen-activated protein kinase kinase 5	MAP2K5			
O94776	Metastasis-associated protein MTA2	MTA2			
Q8IXL7	Methionine-R-sulfoxide reductase B3	MSRB3			
O15069	NAC-alpha domain-containing protein 1	NACAD			
Q15113	Procollagen C-endopeptidase enhancer 1	PCOLCE			
Q15274	Nicotinate-nucleotide pyrophosphorylase	QPRT			
Q96EZ4	Myeloma-overexpressed gene protein	MYEOV			
O00159	Unconventional myosin-Ic	MYO1C			
Q8N660	Neuroblastoma breakpoint family member 15	NBPF15			
P61601	Neurocalcin-delta	NCALD			
Q13772	Nuclear receptor coactivator 4	NCOA4			
Q9ULX3	RNA-binding protein NOB1	NOB1			
Q8N987	N-terminal EF-hand calcium-binding protein 1	NECAB1			
Q9C002	Normal mucosa of esophagus-specific gene 1 protein	NMES1			
Q99457	Nucleosome assembly protein 1-like 3	NAP1L3			
Q5H8A3	Neuromedin-S	NMS			
Q9Y4Z2	Neurogenin-3	NEUROG3			
Q6T4R5	Nance-Horan syndrome protein	NHS			
E5RHQ5	Nuclear pore complex-interacting protein family member B11	NPIPB11			
Q9NXB9	Elongation of very long chain fatty acids protein 2	ELOVL2			
Q9BX51	Glutathione hydrolase light chain	GGTLC1			
Q9Y223	Bifunctional UDP-N-acetylglucosamine 2-epimerase/N-acetylmannosamine kinase	GNE			
P35573	Glycogen debranching enzyme	AGL			
P06396	Gelsolin	GSN			
Q8N8V2	Guanylate-binding protein 7	GBP7			
P34896	Serine hydroxymethyltransferase, cytosolic	SHMT1			
P32456	Guanylate-binding protein 2	GBP2			
P09471	Guanine nucleotide-binding protein G	GNAO1			
P02724	Glycophorin-A	GYPA			
P68431	Histone H3.1	H3C1			
O15379	Histone deacetylase 3	HDAC3			
Q9UQL6	Histone deacetylase 5	HDAC5			

Q9BYW1	Solute carrier family 2, facilitated glucose transporter member 11	SLC2A11			
P34947	G protein-coupled receptor kinase 5	GRK5			
Q8IYU2	E3 ubiquitin-protein ligase HACE1	HACE1			
P10746	Uroporphyrinogen-III synthase	UROS			
Q9NQE9	Histidine triad nucleotide-binding protein 3	HINT3			
O95714	E3 ubiquitin-protein ligase HERC2	HERC2			
A8MVW5	HEPACAM family member 2	HEPACAM2			
Q8NDA2	Hemicentin-2	HMCN2			
Q9UGU5	HMG domain-containing protein	HMGXB4			
Q15651	High mobility group nucleosome-binding domain-containing protein 3	HMGN3			
Q12894	Interferon-related developmental regulator 2	IFRD2			
Q9NWB7	Intraflagellar transport protein 57 homolog	IFT57			
Q8NDH6	Islet cell autoantigen 1-like	ICA1L			
Q96LU5	Mitochondrial inner membrane protease subunit 1	IMMP1L			
Q9H293	Interleukin-25	IL25			
P05362	Intercellular adhesion molecule 1	ICAM1			
P0DOX8	Immunoglobulin lambda-1 light chain				
P32927	Cytokine receptor common subunit beta	CSF2RB			
Q14627	Interleukin-13 receptor subunit alpha-2	IL13RA2			
Q9UKS7	Zinc finger protein Helios	IKZF2			
Q16270	Insulin-like growth factor-binding protein 7	IGFBP7			
P08833	Insulin-like growth factor-binding protein 1	IGFBP1			
Q8NFU5	Inositol polyphosphate multikinase	IPMK			
Q8N5M9	Protein jagunal homolog 1	JAGN1			
Q9NVH2	Integrator complex subunit 7	INTS7			
Q9H511	Kelch-like protein 31	KLHL31			
Q6TDP4	Kelch-like protein 17	KLHL17			
Q587J8	KH domain-containing protein 3	KHDC3L			
P06870	Kallikrein-1	KLK1			
Q9Y2U9	Kelch domain-containing protein	KLHDC2			
O75037	Kinesin-like protein KIF21B	KIF21B			
Q68DU8	BTB/POZ domain-containing protein KCTD16	KCTD16			
P60372	Keratin-associated protein 10-4	KRTAP10-4			
A6NCN2	Putative keratin-87 protein	KRT87P			
Q13751	Laminin subunit beta-3	LAMB3			
P55268	Laminin subunit beta-2	LAMB2			
Q15334	Lethal	LLGL1			
A8MZ59	Paired-like homeodomain transcription factor LEUTX	LEUTX			
O14782	Kinesin-like protein KIF3C	KIF3C			
Q86X40	Leucine-rich repeat-containing protein 28	LRRC28			
Q5SZI1	Low-density lipoprotein receptor class A domain-containing protein 2	LDLRAD2			
Q8NAA5	Leucine-rich repeat-containing protein 75A	LRRC75A			
Q9H2I8	Leucine-rich melanocyte differentiation-associated protein	LRMDA			
O43504	Ragulator complex protein LAMTOR5	LAMTOR5			
Q08397	Lysyl oxidase homolog 1	LOXL1			
Q96NI6	Leucine-rich repeat and fibronectin type-III domain-containing protein 5	LRFN5			

Q9HBW0	Lysophosphatidic acid receptor 2	LPAR2			
O94898	Leucine-rich repeats and immunoglobulin-like domains protein 2	LRIG2			
Q9BQD3	KxDL motif-containing protein 1	KXD1			
Q96JM7	Lethal	L3MBTL3			
O95867	Lymphocyte antigen 6 complex locus protein G6c	LY6G6C			
Q9Y4Z0	U6 snRNA-associated Sm-like protein LSm4	LSM4			
Q8N6C8	Leukocyte immunoglobulin-like receptor subfamily A member 3	LILRA3			
Q8TAP4	LIM domain only protein 3	LMO3			
Q07820	Induced myeloid leukemia cell differentiation protein Mcl-1	MCL1			
Q99759	Mitogen-activated protein kinase kinase kinase 3	MAP3K3			
Q9H204	Mediator of RNA polymerase II transcription subunit 28	MED28			
Q9H2W1	Membrane-spanning 4-domains subfamily A member 6A	MS4A6A			
P40925	Malate dehydrogenase, cytoplasmic	MDH1			
Q96L34	MAP/microtubule affinity-regulating kinase 4	MARK4			
Q9Y5Y7	Lymphatic vessel endothelial hyaluronic acid receptor 1	LYVE1			
Q9UJH8	Meteorin	METRN			
Q86W50	RNA N6-adenosine-methyltransferase METTL16	METTL16			
Q13615	Myotubularin-related protein 3	MTMR3			
A6NDL7	Putative methyltransferase-like protein 21E pseudogene	METTL21EP			
Q8N387	Mucin-15	MUC15			
P28330	Long-chain specific acyl-CoA dehydrogenase, mitochondrial	ACADL			
Q9BYF1	Angiotensin-converting enzyme 2	ACE2			
O75027	ATP-binding cassette sub-family B member 7, mitochondrial	ABCB7			
Q09428	ATP-binding cassette sub-family C member 8	ABCC8			
P30542	Adenosine receptor A1	ADORA1			
Q719I0	Putative activator of 90 kDa heat shock protein ATPase homolog 2	AHSA2P			
Q9UH17	DNA dC->dU-editing enzyme APOBEC-3B	APOBEC3B			
Q9GZX7	Single-stranded DNA cytosine deaminase	AICDA			
P14060	3 beta-hydroxysteroid dehydrogenase/Delta 5-->4-isomerase type 1	HSD3B1			
P14621	Acylphosphatase-2	ACYP2			
Q86WK7	Amphoterin-induced protein 3	AMIGO3			
Q8TC94	Actin-like protein 9	ACTL9			
Q8TDG2	Actin-related protein T1	ACTRT1			
Q02952	A-kinase anchor protein 12	AKAP12			
P52895	Aldo-keto reductase family 1 member C2	AKR1C2			
Q3MIX3	Uncharacterized aarF domain-containing protein kinase 5	ADCK5			
Q9NWT8	Aurora kinase A-interacting protein	AURKAIP1			
Q68CK6	Acyl-coenzyme A synthetase ACSM2B, mitochondrial	ACSM2B			
Q9P2G1	Ankyrin repeat and IBR domain-containing protein 1	ANKIB1			
Q6UXT8	ALK and LTK ligand 1	ALKAL1			
P31749	RAC-alpha serine/threonine-protein kinase	AKT1			

P25100	Alpha-1D adrenergic receptor	ADRA1D				
Q86W34	Archaemetzincin-2	AMZ2				
O00189	AP-4 complex subunit mu-1	AP4M1				
Q9BWW9	Apolipoprotein L5	APOL5				
O14791	Apolipoprotein L1	APOL1				
Q96DR7	Rho guanine nucleotide exchange factor 26	ARHGEF26				
Q9Y4B4	Helicase ARIP4	RAD54L2				
Q15327	Ankyrin repeat domain-containing protein 1	ANKRD1				
O94817	Ubiquitin-like protein ATG12	ATG12				
O00148	ATP-dependent RNA helicase DDX39A	DDX39A				
Q9UDT6	CAP-Gly domain-containing linker protein 2	CLIP2				
Q9BSU1	UPF0183 protein C16orf70	C16orf70				
H3BN30	Uncharacterized protein C16orf97	C16orf97				
P42126	Enoyl-CoA delta isomerase 1, mitochondrial	ECI1				
Q96PZ7	CUB and sushi domain-containing protein 1	CSMD1				
Q1HG44	Dual oxidase maturation factor 2	DUOXA2				
Q9UKG1	DCC-interacting protein 13-alpha	APPL1				
Q9BY08	Emopamil-binding protein-like	EBPL				
Q5VYK3	Proteasome adapter and scaffold protein ECM29	ECPAS				
A0A0U1RQF7	DPEP2 neighbor protein	DPEP2NB				
Q8TB45	DEP domain-containing mTOR-interacting protein	DEPTOR				
P43003	Excitatory amino acid transporter 1	SLC1A3				
P43004	Excitatory amino acid transporter 2	SLC1A2				
P20036	HLA class II histocompatibility antigen, DP alpha 1 chain	HLA-DPA1				
P43005	Excitatory amino acid transporter 3	SLC1A1				
Q9C005	Protein dpy-30 homolog	DPY30				
P48664	Excitatory amino acid transporter 4	SLC1A6				
O00341	Excitatory amino acid transporter 5	SLC1A7				
Q12882	Dihydropyrimidine	DPYD				
P04440	HLA class II histocompatibility antigen, DP beta 1 chain	HLA-DPB1				
Q14194	Dihydropyrimidinase-related protein 1	CRMP1				
Q16555	Dihydropyrimidinase-related protein 2	DPYSL2				
Q14195	Dihydropyrimidinase-related protein 3	DPYSL3				
Q86SG4	Putative Dresden prostate carcinoma protein 2	HMGN2P46				
Q96JC9	ELL-associated factor 1	EAF1				
Q9BVM2	Protein DPCD	DPCD				
O14531	Dihydropyrimidinase-related protein 4	DPYSL4				
Q9BPU6	Dihydropyrimidinase-related protein 5	DPYSL5				
Q96CJ1	ELL-associated factor 2	EAF2				
Q14117	Dihydropyrimidinase	DPYS				
P01909	HLA class II histocompatibility antigen, DQ alpha 1 chain	HLA-DQA1				
P01906	HLA class II histocompatibility antigen, DQ alpha 2 chain	HLA-DQA2				
P01920	HLA class II histocompatibility antigen, DQ beta 1 chain	HLA-DQB1				

P05538	HLA class II histocompatibility antigen, DQ beta 2 chain	HLA-DQB2			
Q8TE96	ATP-dependent RNA helicase DQX1	DQX1			
P0CG22	Putative dehydrogenase/reductase SDR family member 4-like 1	DHRS4L1			
Q6PKH6	Dehydrogenase/reductase SDR family member 4-like 2	DHRS4L2			
Q0D2K5	Putative EGF-like and EMI domain-containing protein 1	EGFEM1P			
Q04741	Homeobox protein EMX1	EMX1			
Q04743	Homeobox protein EMX2	EMX2			
Q8IUX8	Epidermal growth factor-like protein 6	EGFL6			
Q902F9	Endogenous retrovirus group K member 113 Env polyprotein	HERVK_113			
Q9UHF1	Epidermal growth factor-like protein 7	EGFL7			
Q8N8S7	Protein enabled homolog	ENAH			
Q99944	Epidermal growth factor-like protein 8	EGFL8			
Q9NRM1	Enamelin	ENAM			
Q63HQ2	Pikachurin	EGFLAM			
Q8NFI3	Cytosolic endo-beta-N-acetylglucosaminidase	ENGASE			
O14682	Ectoderm-neural cortex protein 1	ENC1			
Q58FF3	Putative endoplasmin-like protein	HSP90B2P			
P14625	Endoplasmin	HSP90B1			
P22413	Ectonucleotide pyrophosphatase/phosphodiesterase family member 1	ENPP1			
Q13822	Ectonucleotide pyrophosphatase/phosphodiesterase family member 2	ENPP2			
O14638	Ectonucleotide pyrophosphatase/phosphodiesterase family member 3	ENPP3			
P53672	Beta-crystallin A2	CRYBA2			
Q8NF67	Putative ankyrin repeat domain-containing protein 20A12 pseudogene	ANKRD20A12P			
Q8IZY2	Phospholipid-transporting ATPase ABCA7	ABCA7			
Q66LE6	Serine/threonine-protein phosphatase 2A 55 kDa regulatory subunit B delta isoform	PPP2R2D			
Q15173	Serine/threonine-protein phosphatase 2A 56 kDa regulatory subunit beta isoform	PPP2R5B			
Q96AK3	DNA dC->dU-editing enzyme APOBEC-3D	APOBEC3D			
Q9HC16	DNA dC->dU-editing enzyme APOBEC-3G	APOBEC3G			
P07311	Acylphosphatase-1	ACYP1			
P30837	Aldehyde dehydrogenase X, mitochondrial	ALDH1B1			
P14550	Aldo-keto reductase family 1 member A1	AKR1A1			
O60641	Clathrin coat assembly protein AP180	SNAP91			
Q96QP1	Alpha-protein kinase 1	ALPK1			
Q09666	Neuroblast differentiation-associated protein AHNAK	AHNAK			
Q96PN6	Adenylate cyclase type 10	ADCY10			
Q8IWK6	Adhesion G protein-coupled receptor A3	ADGRA3			
Q9UHX3	Adhesion G protein-coupled receptor E2	ADGRE2			

Q96Q83	Alpha-ketoglutarate-dependent dioxygenase alkB homolog 3	ALKBH3			
O14514	Adhesion G protein-coupled receptor B1	ADGRB1			
Q99767	Amyloid-beta A4 precursor protein-binding family A member 2	APBA2			
Q96K21	Abscission/NoCut checkpoint regulator	ZFYVE19			
P19961	Alpha-amylase 2B	AMY2B			
P55064	Aquaporin-5	AQP5			
Q3SXY8	ADP-ribosylation factor-like protein 13B	ARL13B			
O75143	Autophagy-related protein 13	ATG13			
Q9NVI7	ATPase family AAA domain-containing protein 3A	ATAD3A			
P08243	Asparagine synthetase	ASNS			
A6NK59	Ankyrin repeat and SOCS box protein 14	ASB14			
Q9Y575	Ankyrin repeat and SOCS box protein 3	ASB3			
Q9Y284	Protein Asterix	WDR83OS			
Q5TC04	Putative uncharacterized protein ATP1A1-AS1	ATP1A1-AS1			
Q8TF62	Probable phospholipid-transporting ATPase IM	ATP8B4			
Q9H981	Actin-related protein 8	ACTR8			
Q96DT6	Cysteine protease ATG4C	ATG4C			
Q96A70	Antizyme inhibitor 2	AZIN2			
O75964	ATP synthase subunit g, mitochondrial	ATP5MG			
P36575	Arrestin-C	ARR3			
P51798	H	CLCN7			
P56749	Claudin-12	CLDN12			
Q9Y5I7	Claudin-16	CLDN16			
Q13825	Methylglutaconyl-CoA hydratase, mitochondrial	AUH			
P51164	Potassium-transporting ATPase subunit beta	ATP4B			
Q9BXY8	Protein BEX2	BEX2			
Q96NH3	Protein broad-minded	TBC1D32			
Q15582	Transforming growth factor-beta-induced protein ig-h3	TGFBI			
Q9UPA5	Protein bassoon	BSN			
O43505	Beta-1,4-glucuronyltransferase 1	B4GAT1			
Q8N9N5	Protein BANP	BANP			
O14503	Class E basic helix-loop-helix protein 40	BHLHE40			
Q9NPB3	Calcium-binding protein 2	CABP2			
Q96KX2	F-actin-capping protein subunit alpha-3	CAPZA3			
P17655	Calpain-2 catalytic subunit	CAPN2			
Q96LZ3	Calcineurin subunit B type 2	PPP3R2			
Q13698	Voltage-dependent L-type calcium channel subunit alpha-1S	CACNA1S			
Q8N5S3	Uncharacterized protein C2orf73	C2orf73			
Q13112	Chromatin assembly factor 1 subunit B	CHAF1B			
P19022	Cadherin-2	CDH2			
Q96LT6	UPF0739 protein C1orf74	C1orf74			
Q6TDU7	Dynein intermediate chain CFAP94, axonemal	CFAP94			
P29466	Caspase-1	CASP1			
A6NLJ0	C2 calcium-dependent domain-containing protein 4B	C2CD4B			
Q86UW7	Calcium-dependent secretion activator 2	CADPS2			
Q8TAB7	Putative coiled-coil domain-containing protein 26	CCDC26			

P15169	Carboxypeptidase N catalytic chain	CPN1			
P07451	Carbonic anhydrase 3	CA3			
Q52MB2	Coiled-coil domain-containing protein 184	CCDC184			
Q5JU67	Cilia- and flagella-associated protein 157	CFAP157			
P07858	Cathepsin B	CTSB			
Q8NA61	Protein chibby homolog 2	CBY2			
O43822	Cilia- and flagella-associated protein 410	CFAP410			
P51681	C-C chemokine receptor type 5	CCR5			
P35790	Choline kinase alpha	CHKA			
Q8TAL5	Uncharacterized protein C9orf43	C9orf43			
Q8N9H6	Putative uncharacterized protein C8orf31	C8orf31			
A6NFR6	Uncharacterized protein C5orf60	C5orf60			
P49918	Cyclin-dependent kinase inhibitor 1C	CDKN1C			
Q8WVX3	Uncharacterized protein C4orf3	C4orf3			
Q9UKY3	Putative inactive carboxylesterase 4	CES1P1			
Q00536	Cyclin-dependent kinase 16	CDK16			
G9CGD6	CNK3/IPCEF1 fusion protein	CNK3/IPCEF1			
Q8ND76	Cyclin-Y	CCNY			
Q9BWU1	Cyclin-dependent kinase 19	CDK19			
Q00534	Cyclin-dependent kinase 6	CDK6			
Q96M19	Putative transmembrane protein encoded by LINC00477	LINC00477			
Q15417	Calponin-3	CNN3			
Q6X4T0	Uncharacterized protein C12orf54	C12orf54			
Q9NY25	C-type lectin domain family 5 member A	CLEC5A			
Q5SQS8	Uncharacterized protein C10orf120	C10orf120			
Q96N53	Putative uncharacterized protein encoded by LINC00167	LINC00167			
Q9NQW8	Cyclic nucleotide-gated cation channel beta-3	CNGB3			
A6NMZ7	Collagen alpha-6	COL6A6			
Q13286	Battenin	CLN3			
Q5VST6	Alpha/beta hydrolase domain-containing protein 17B	ABHD17B			
Q6PCB6	Alpha/beta hydrolase domain-containing protein 17C	ABHD17C			
P11766	Alcohol dehydrogenase class-3	ADH5			
Q9NZD4	Alpha-hemoglobin-stabilizing protein	AHSP			
P54646	5'-AMP-activated protein kinase catalytic subunit alpha-2	PRKAA2			
Q3SY69	Mitochondrial 10-formyltetrahydrofolate dehydrogenase	ALDH1L2			
Q86SJ2	Amphoterin-induced protein 2	AMIGO2			
Q08043	Alpha-actinin-3	ACTN3			
Q53H64	Putative ANKRD40 C-terminal-like protein	ANKRD40CL			
Q6P461	Acyl-coenzyme A synthetase ACSM6, mitochondrial	ACSM6			
Q08828	Adenylate cyclase type 1	ADCY1			
Q8N6S4	Ankyrin repeat domain-containing protein 13C	ANKRD13C			
P62736	Actin, aortic smooth muscle	ACTA2			
Q9BVK2	Probable dolichyl pyrophosphate Glc1Man9GlcNAc2 alpha-1,3-glucosyltransferase	ALG8			
F7VJQ1	Alternative prion protein	PRNP			

Q8WVL7	Ankyrin repeat domain-containing protein 49	ANKRD49			
Q92625	Ankyrin repeat and SAM domain-containing protein 1A	ANKS1A			
P05549	Transcription factor AP-2-alpha	TFAP2A			
Q6UW56	All-trans retinoic acid-induced differentiation factor	ATRAID			
Q6UXH0	Angiopoietin-like protein 8	ANGPTL8			
Q86WX3	Active regulator of SIRT1	RPS19BP1			
Q8WXJ9	Ankyrin repeat and SOCS box protein 17	ASB17			
P18846	Cyclic AMP-dependent transcription factor ATF-1	ATF1			
Q16515	Acid-sensing ion channel 2	ASIC2			
Q7Z4Y8	Putative ATP synthase subunit g 2, mitochondrial	ATP5MGL			
Q7Z6K5	Arpin	ARPIN			
P05496	ATP synthase F	ATP5MC1			
O75508	Claudin-11	CLDN11			
A6NM45	Putative claudin-24	CLDN24			
P57739	Claudin-2	CLDN2			
H7C241	Claudin-34	CLDN34			
O15551	Claudin-3	CLDN3			
O95471	Claudin-7	CLDN7			
Q96GD4	Aurora kinase B	AURKB			
Q3ZCQ2	Annexin-2 receptor	ANXA2R			
Q8TE57	A disintegrin and metalloproteinase with thrombospondin motifs 16	ADAMTS16			
Q9NZS9	Bifunctional apoptosis regulator	BFAR			
Q00994	Protein BEX3	BEX3			
Q76B58	BMP/retinoic acid-inducible neural-specific protein 3	BRINP3			
Q5PSV4	Breast cancer metastasis-suppressor 1-like protein	BRMS1L			
Q9NSY1	BMP-2-inducible protein kinase	BMP2K			
Q32M84	BTB/POZ domain-containing protein 16	BTBD16			
P32247	Bombesin receptor subtype-3	BRS3			
P41223	Protein BUD31 homolog	BUD31			
P38398	Breast cancer type 1 susceptibility protein	BRCA1			
O43286	Beta-1,4-galactosyltransferase 5	B4GALT5			
O60512	Beta-1,4-galactosyltransferase 3	B4GALT3			
Q8TDM0	Breast carcinoma-amplified sequence 4	BCAS4			
Q6P575	Putative inactive beta-glucuronidase protein GUSBP11	GUSBP11			
O75531	Barrier-to-autointegration factor	BANF1			
Q8NFU0	Bestrophin-4	BEST4			
Q8N888	Putative BCoR-like protein 2	BCORP1			
Q16611	Bcl-2 homologous antagonist/killer	BAK1			
Q9BXL6	Caspase recruitment domain-containing protein 14	CARD14			
Q00975	Voltage-dependent N-type calcium channel subunit alpha-1B	CACNA1B			
Q9H0H9	Putative cytochrome P450 family member 4F30	CYP4F30P			
P04003	C4b-binding protein alpha chain	C4BPA			
P40121	Macrophage-capping protein	CAPG			
Q9NZP8	Complement C1r subcomponent-like protein	C1RL			
Q9H8G2	Caspase activity and apoptosis inhibitor 1	CAAP1			
Q96A33	Coiled-coil domain-containing protein 47	CCDC47			
Q14511	Enhancer of filamentation 1	NEDD9			

Q8IWP9	Coiled-coil domain-containing protein 28A	CCDC28A			
Q8NCX0	Coiled-coil domain-containing protein 150	CCDC150			
A6NNP5	Coiled-coil domain-containing protein 169	CCDC169			
O60359	Voltage-dependent calcium channel gamma-3 subunit	CACNG3			
P62955	Voltage-dependent calcium channel gamma-7 subunit	CACNG7			
B2RV13	Sperm axonemal maintenance protein CFAP97D1	CFAP97D1			
A0A096LP49	Coiled-coil domain-containing protein 187	CCDC187			
Q3KPI0	Carcinoembryonic antigen-related cell adhesion molecule 21	CEACAM21			
P16870	Carboxypeptidase E	CPE			
P08861	Chymotrypsin-like elastase family member 3B	CELA3B			
Q9Y6F7	Testis-specific chromodomain protein Y 2	CDY2A			
Q8NEG2	Uncharacterized protein C7orf57	C7orf57			
O00421	C-C chemokine receptor-like 2	CCRL2			
Q5W0N0	Uncharacterized protein C9orf57	C9orf57			
P00450	Ceruloplasmin	CP			
Q16589	Cyclin-G2	CCNG2			
Q6PRD7	Cementoblastoma-derived protein 1	CEMP1			
Q12798	Centrin-1	CETN1			
Q6P1J9	Parafibromin	CDC73			
P32320	Cytidine deaminase	CDA			
Q8N4M7	Putative uncharacterized protein C10orf126	C10orf126			
Q8NA66	Cyclic nucleotide-binding domain-containing protein 1	CNBD1			
Q86U37	Uncharacterized protein encoded by LINC01551	LINC01551			
P12110	Collagen alpha-2	COL6A2			
Q96NS8	Putative protein CLUHP3	CLUHP3			
Q16280	Cyclic nucleotide-gated olfactory channel	CNGA2			
Q9NPC3	E3 ubiquitin-protein ligase CCNB1IP1	CCNB1IP1			
Q53SF7	Cordon-bleu protein-like 1	COBLL1			
Q8IUL8	Cartilage intermediate layer protein 2	CILP2			
Q5TA50	Ceramide-1-phosphate transfer protein	CPTP			
O75177	Calcium-responsive transactivator	SS18L1			
P05181	Cytochrome P450 2E1	CYP2E1			
Q5T197	E3 ubiquitin-protein ligase DCST1	DCST1			
O43293	Death-associated protein kinase 3	DAPK3			
Q9UHI6	Probable ATP-dependent RNA helicase DDX20	DDX20			
Q9GZZ6	Neuronal acetylcholine receptor subunit alpha-10	CHRNA10			
P07108	Acyl-CoA-binding protein	DBI			
Q9H3Z7	Protein ABHD16B	ABHD16B			
O94911	ABC-type organic anion transporter ABCA8	ABCA8			
Q00005	Serine/threonine-protein phosphatase 2A 55 kDa regulatory subunit B beta isoform	PPP2R2B			
P43353	Aldehyde dehydrogenase family 3 member B1	ALDH3B1			
Q5VW22	Arf-GAP with GTPase, ANK repeat and PH domain-containing protein 6	AGAP6			
Q96Q42	Alsin	ALS2			

P68133	Actin, alpha skeletal muscle	ACTA1			
Q99996	A-kinase anchor protein 9	AKAP9			
P10323	Acrosin	ACR			
P00813	Adenosine deaminase	ADA			
P50052	Type-2 angiotensin II receptor	AGTR2			
Q9NP73	Putative bifunctional UDP-N-acetylglucosamine transferase and deubiquitinase ALG13	ALG13			
P08588	Beta-1 adrenergic receptor	ADRB1			
P0C7M7	Acyl-coenzyme A synthetase ACSM4, mitochondrial	ACSM4			
P08913	Alpha-2A adrenergic receptor	ADRA2A			
Q9HCE9	Anoctamin-8	ANO8			
Q8WXG9	Adhesion G-protein coupled receptor V1	ADGRV1			
O75179	Ankyrin repeat domain-containing protein 17	ANKRD17			
Q53RT3	Retroviral-like aspartic protease 1	ASPRV1			
Q92754	Transcription factor AP-2 gamma	TFAP2C			
Q9UJX3	Anaphase-promoting complex subunit 7	ANAPC7			
C9JTQ0	Ankyrin repeat domain-containing protein 63	ANKRD63			
P20073	Annexin A7	ANXA7			
Q9UPS8	Ankyrin repeat domain-containing protein 26	ANKRD26			
P25098	Beta-adrenergic receptor kinase 1	GRK2			
Q9Y5C1	Angiopoietin-related protein 3	ANGPTL3			
P23582	C-type natriuretic peptide	NPPC			
O15143	Actin-related protein 2/3 complex subunit 1B	ARPC1B			
Q9BQD7	Adenine nucleotide translocase lysine N-methyltransferase	ANTKMT			
P15336	Cyclic AMP-dependent transcription factor ATF-2	ATF2			
Q9Y2D1	Cyclic AMP-dependent transcription factor ATF-5	ATF5			
Q9UBL3	Set1/Ash2 histone methyltransferase complex subunit ASH2	ASH2L			
P78348	Acid-sensing ion channel 1	ASIC1			
Q96BY7	Autophagy-related protein 2 homolog B	ATG2B			
O60312	Phospholipid-transporting ATPase VA	ATP10A			
Q8TBH0	Arrestin domain-containing protein 2	ARRDC2			
O14977	Antizyme inhibitor 1	AZIN1			
Q99700	Ataxin-2	ATXN2			
Q9H3M9	Ataxin-3-like protein	ATXN3L			
Q9Y679	Lipid droplet-regulating VLDL assembly factor AUP1	AUP1			
Q76LX8	A disintegrin and metalloproteinase with thrombospondin motifs 13	ADAMTS13			
Q8N9R0	Putative uncharacterized protein encoded by LINC00304	LINC00304			
O43313	ATM interactor	ATMIN			
Q6NZY7	Cdc42 effector protein 5	CDC42EP5			
P18075	Bone morphogenetic protein 7	BMP7			
O00499	Myc box-dependent-interacting protein 1	BIN1			
O75815	Breast cancer anti-estrogen resistance protein 3	BCAR3			
Q96KC9	Calcium-binding and spermatid-specific protein 1	CABS1			
Q13145	BMP and activin membrane-bound inhibitor homolog	BAMBI			

Q9NR55	Basic leucine zipper transcriptional factor ATF-like 3	BATF3			
Q13938	Calcyphosin	CAPS			
P07339	Cathepsin D	CTSD			
P25774	Cathepsin S	CTSS			
Q08289	Voltage-dependent L-type calcium channel subunit beta-2	CACNB2			
O75052	Carboxyl-terminal PDZ ligand of neuronal nitric oxide synthase protein	NOS1AP			
Q86WR0	Coiled-coil domain-containing protein 25	CCDC25			
Q6UB35	Monofunctional C1-tetrahydrofolate synthase, mitochondrial	MTHFD1L			
Q8NCB2	CaM kinase-like vesicle-associated protein	CAMKV			
Q9H2F9	Coiled-coil domain-containing protein 68	CCDC68			
P0DP25	Calmodulin-3	CALM3			
Q8N813	Putative uncharacterized protein C3orf56	C3orf56			
P0DP23	Calmodulin-1	CALM1			
P78556	C-C motif chemokine 20	CCL20			
P10966	T-cell surface glycoprotein CD8 beta chain	CD8B			
P41180	Extracellular calcium-sensing receptor	CASR			
P57076	Cilia- and flagella-associated protein 298	CFAP298			
P42772	Cyclin-dependent kinase 4 inhibitor B	CDKN2B			
Q9BSY4	Coiled-coil-helix-coiled-coil-helix domain-containing protein 5	CHCHD5			
Q9H7U1	Serine-rich coiled-coil domain-containing protein 2	CCSER2			
Q14839	Chromodomain-helicase-DNA-binding protein 4	CHD4			
Q49MI3	Ceramide kinase-like protein	CERKL			
P30203	T-cell differentiation antigen CD6	CD6			
Q6ZV77	Uncharacterized protein C9orf139	C9orf139			
O95873	Uncharacterized protein C6orf47	C6orf47			
P48745	CCN family member 3	CCN3			
A8MTZ7	Uncharacterized protein C12orf71	C12orf71			
Q9BV87	Protein CNPPD1	CNPPD1			
Q9ULM6	CCR4-NOT transcription complex subunit 6	CNOT6			
Q96FZ5	CKLF-like MARVEL transmembrane domain-containing protein 7	CMTM7			
Q7Z7L8	Uncharacterized protein C11orf96	C11orf96			
Q05707	Collagen alpha-1	COL14A1			
O14734	Acyl-coenzyme A thioesterase 8	ACOT8			
Q9NUZ1	Acyl-coenzyme A oxidase-like protein	ACOXL			
Q4AC99	Probable inactive 1-aminocyclopropane-1-carboxylate synthase-like protein 2	ACCSL			
P49753	Acyl-coenzyme A thioesterase 2, mitochondrial	ACOT2			
P61221	ATP-binding cassette sub-family E member 1	ABCE1			
Q9Y235	C->U-editing enzyme APOBEC-2	APOBEC2			
Q9Y478	5'-AMP-activated protein kinase subunit beta-1	PRKAB1			
P30153	Serine/threonine-protein phosphatase 2A 65 kDa regulatory subunit A alpha isoform	PPP2R1A			

Q6ULP2	Aftiphilin	AFTPH			
Q13131	5'-AMP-activated protein kinase catalytic subunit alpha-1	PRKAA1			
O95831	Apoptosis-inducing factor 1, mitochondrial	AIFM1			
Q8N944	APC membrane recruitment protein 3	AMER3			
Q7L8J4	SH3 domain-binding protein 5-like	SH3BP5L			
Q9NVV5	Androgen-induced gene 1 protein	AIG1			
Q96B36	Proline-rich AKT1 substrate 1	AKT1S1			
Q15848	Adiponectin	ADIPOQ			
Q8TDY3	Actin-related protein T2	ACTRT2			
Q5T2L2	Putative aldo-keto reductase family 1 member C8	AKR1C8P			
P24588	A-kinase anchor protein 5	AKAP5			
Q9H9G7	Protein argonaute-3	AGO3			
O75689	Arf-GAP with dual PH domain-containing protein 1	ADAP1			
Q08462	Adenylate cyclase type 2	ADCY2			
Q9NUB1	Acetyl-coenzyme A synthetase 2-like, mitochondrial	ACSS1			
Q8NFM4	Adenylate cyclase type 4	ADCY4			
Q8IZF6	Adhesion G-protein coupled receptor G4	ADGRG4			
Q9NXR5	Ankyrin repeat domain-containing protein 10	ANKRD10			
Q13444	Disintegrin and metalloproteinase domain-containing protein 15	ADAM15			
Q6P6B7	Ankyrin repeat domain-containing protein 16	ANKRD16			
P17342	Atrial natriuretic peptide receptor 3	NPR3			
P63010	AP-2 complex subunit beta	AP2B1			
P56377	AP-1 complex subunit sigma-2	AP1S2			
Q8NBQ7	Aquaporin-11	AQP11			
Q9C0K3	Actin-related protein 3C	ACTR3C			
Q8WXK1	Ankyrin repeat and SOCS box protein 15	ASB15			
O95500	Claudin-14	CLDN14			
Q8WWM7	Ataxin-2-like protein	ATXN2L			
Q5T686	Arginine vasopressin-induced protein 1	AVPI1			
O75110	Probable phospholipid-transporting ATPase IIA	ATP9A			
O75947	ATP synthase subunit d, mitochondrial	ATP5PD			
Q13315	Serine-protein kinase ATM	ATM			
P54259	Atrophin-1	ATN1			
Q8TDC3	Serine/threonine-protein kinase BRSK1	BRSK1			
Q9NY30	Protein BTG4	BTG4			
Q9UBR1	Beta-ureidopropionase	UPB1			
Q6ZUJ8	Phosphoinositide 3-kinase adapter protein 1	PIK3AP1			
A8MW95	Beclin-2	BECN2			
Q6PL45	BRICHOS domain-containing protein 5	BRICD5			
Q13323	Bcl-2-interacting killer	BIK			
Q86Y30	B melanoma antigen 2	BAGE2			
Q9HC96	Calpain-10	CAPN10			
Q9P296	C5a anaphylatoxin chemotactic receptor 2	C5AR2			
Q8N5C1	Calcium homeostasis modulator protein 5	CALHM5			
Q8N8Q1	Cytochrome b561 domain-containing protein 1	CYB561D1			
P43155	Carnitine O-acetyltransferase	CRAT			
P0DPF5	Uncharacterized protein C2orf27A	C2orf27A			

P47756	F-actin-capping protein subunit beta	CAPZB			
Q8N0U7	Uncharacterized protein C1orf87	C1orf87			
Q9H425	Uncharacterized protein C1orf198	C1orf198			
Q6IMN6	Caprin-2	CAPRIN2			
Q9Y6R9	Centrosomal protein CCDC61	CCDC61			
P0DP24	Calmodulin-2	CALM2			
Q569K6	Coiled-coil domain-containing protein 157	CCDC157			
P14384	Carboxypeptidase M	CPM			
P80075	C-C motif chemokine 8	CCL8			
Q6UW01	Cerebellin-3	CBLN3			
P15085	Carboxypeptidase A1	CPA1			
P22681	E3 ubiquitin-protein ligase CBL	CBL			
Q96EP1	E3 ubiquitin-protein ligase CHFR	CHFR			
P31997	Carcinoembryonic antigen-related cell adhesion molecule 8	CEACAM8			
P17676	CCAAT/enhancer-binding protein beta	CEBPB			
O95992	Cholesterol 25-hydroxylase	CH25H			
P15812	T-cell surface glycoprotein CD1e, membrane-associated	CD1E			
P51861	Cerebellar degeneration-related antigen 1	CDR1			
Q9UNE7	E3 ubiquitin-protein ligase CHIP	STUB1			
Q96T59	CMT1A duplicated region transcript 15 protein	CDRT15			
Q9NPF0	CD320 antigen	CD320			
Q8N8E3	Centrosomal protein of 112 kDa	CEP112			
A6NKQ9	Choriogonadotropin subunit beta variant 1	CGB1			
P30281	G1/S-specific cyclin-D3	CCND3			
Q9ULY5	C-type lectin domain family 4 member E	CLEC4E			
P0C7P0	CDGSH iron-sulfur domain-containing protein 3, mitochondrial	CISD3			
P01024	Complement C3	C3			
P10643	Complement component C7	C7			
Q8IV77	Cyclic nucleotide-gated cation channel alpha-4	CNGA4			
Q9UBR5	Chemokine-like factor	CKLF			
Q9UPZ9	Serine/threonine-protein kinase ICK	CILK1			
P49759	Dual specificity protein kinase	CLK1			
Q99715	Collagen alpha-1	COL12A1			
Q92828	Coronin-2A	CORO2A			
Q9HBI6	Cytochrome P450 4F11	CYP4F11			
Q9UBL6	Copine-7	CPNE7			
P51589	Cytochrome P450 2J2	CYP2J2			
Q6GPI1	Chymotrypsinogen B2	CTRB2			
Q9H5V9	UPF0428 protein CXorf56	CXorf56			
P12821	Angiotensin-converting enzyme	ACE			
O14678	Lysosomal cobalamin transporter ABCD4	ABCD4			
P52209	6-phosphogluconate dehydrogenase, decarboxylating	PGD			
P43681	Neuronal acetylcholine receptor subunit alpha-4	CHRNA4			
P01011	Alpha-1-antichymotrypsin	SERPINA3			
Q9Y2T4	Serine/threonine-protein phosphatase 2A 55 kDa regulatory subunit B gamma isoform	PPP2R2C			
Q8TCU4	Alstrom syndrome protein 1	ALMS1			
Q8N302	Angiogenic factor with G patch and FHA domains 1	AGGF1			
P42330	Aldo-keto reductase family 1 member C3	AKR1C3			
Q8N157	Jouberin	AHI1			

P78325	Disintegrin and metalloproteinase domain-containing protein 8	ADAM8			
Q7Z7M1	Adhesion G-protein coupled receptor D2	ADGRD2			
Q96PE1	Adhesion G protein-coupled receptor A2	ADGRA2			
Q8IZF3	Adhesion G protein-coupled receptor F4	ADGRF4			
O95573	Long-chain-fatty-acid--CoA ligase 3	ACSL3			
Q92685	Dol-P-Man:Man	ALG3			
P78536	Disintegrin and metalloproteinase domain-containing protein 17	ADAM17			
Q13686	Nucleic acid dioxygenase ALKBH1	ALKBH1			
P07741	Adenine phosphoribosyltransferase	APRT			
O43299	AP-5 complex subunit zeta-1	AP5Z1			
P53680	AP-2 complex subunit sigma	AP2S1			
Q8IVJ8	AP20 region protein 1	APRG1			
O14617	AP-3 complex subunit delta-1	AP3D1			
P15144	Aminopeptidase N	ANPEP			
Q96KC2	ADP-ribosylation factor-like protein 5B	ARL5B			
P05089	Arginase-1	ARG1			
O95154	Aflatoxin B1 aldehyde reductase member 3	AKR7A3			
A6NL99	Putative aquaporin-7-like protein 3	AQP7P3			
Q86XL3	Ankyrin repeat and LEM domain-containing protein 2	ANKLE2			
Q969Q4	ADP-ribosylation factor-like protein 11	ARL11			
Q7LC44	Activity-regulated cytoskeleton-associated protein	ARC			
O94778	Aquaporin-8	AQP8			
P0C7Q2	Age-related maculopathy susceptibility protein 2	ARMS2			
Q9BXN1	Asporin	ASPN			
Q9Y574	Ankyrin repeat and SOCS box protein 4	ASB4			
Q99941	Cyclic AMP-dependent transcription factor ATF-6 beta	ATF6B			
Q9H672	Ankyrin repeat and SOCS box protein 7	ASB7			
Q7Z3C6	Autophagy-related protein 9A	ATG9A			
P54707	Potassium-transporting ATPase alpha chain 2	ATP12A			
Q92484	Acid sphingomyelinase-like phosphodiesterase 3a	SMPDL3A			
Q96QS3	Homeobox protein ARX	ARX			
O75882	Attractin	ATRN			
Q9UP79	A disintegrin and metalloproteinase with thrombospondin motifs 8	ADAMTS8			
Q8TE56	A disintegrin and metalloproteinase with thrombospondin motifs 17	ADAMTS17			
Q8TE59	A disintegrin and metalloproteinase with thrombospondin motifs 19	ADAMTS19			
P00846	ATP synthase subunit a	MT-ATP6			
Q6ZMM2	ADAMTS-like protein 5	ADAMTSL5			
O00244	Copper transport protein ATOX1	ATOX1			
Q6RI45	Bromodomain and WD repeat-containing protein 3	BRWD3			
A6NE02	BTB/POZ domain-containing protein 17	BTBD17			
Q9BX70	BTB/POZ domain-containing protein 2	BTBD2			

Q8NFJ9	Bardet-Biedl syndrome 1 protein	BBS1			
Q14137	Ribosome biogenesis protein BOP1	BOP1			
P08236	Beta-glucuronidase	GUSB			
Q13936	Voltage-dependent L-type calcium channel subunit alpha-1C	CACNA1C			
P09228	Cystatin-SA	CST2			
A6NHC0	Calpain-8	CAPN8			
Q86Y37	CDK2-associated and cullin domain-containing protein 1	CACUL1			
Q9NWW7	Uncharacterized protein C2orf42	C2orf42			
Q9UKL3	CASP8-associated protein 2	CASP8AP2			
P55289	Cadherin-12	CDH12			
Q9UJ99	Cadherin-22	CDH22			
Q99643	Succinate dehydrogenase cytochrome b560 subunit, mitochondrial	SDHC			
Q8TAB5	UPF0500 protein C1orf216	C1orf216			
Q3SY05	Putative uncharacterized protein encoded by LINC00303	LINC00303			
Q9BY67	Cell adhesion molecule 1	CADM1			
Q9H4Z3	mRNA	PCIF1			
Q13901	Nuclear nucleic acid-binding protein C1D	C1D			
Q9ULX7	Carbonic anhydrase 14	CA14			
Q9Y6J0	Calcineurin-binding protein cabin-1	CABIN1			
Q6ZSU1	Putative inactive cytochrome P450 2G1	CYP2G1P			
P49913	Cathelicidin antimicrobial peptide	CAMP			
Q2TAC2	Coiled-coil domain-containing protein 57	CCDC57			
P51636	Caveolin-2	CAV2			
Q9UFE4	Coiled-coil domain-containing protein 39	CCDC39			
Q9P0B6	Coiled-coil domain-containing protein 167	CCDC167			
O95273	Cyclin-D1-binding protein 1	CCNDBP1			
Q13939	Calicin	CCIN			
A0A1B0GVQ3	Coiled-coil domain-containing protein 200	CCDC200			
Q8IUK8	Cerebellin-2	CBLN2			
Q96LY2	Coiled-coil domain-containing protein 74B	CCDC74B			
A1A4V9	Coiled-coil domain-containing protein 189	CCDC189			
Q9NUG4	Cerebral cavernous malformations 2 protein-like	CCM2L			
A8K010	Putative transcriptional regulator encoded by LINC00473	LINC00473			
A6NHX0	Cytosolic arginine sensor for mTORC1 subunit 2	CASTOR2			
P49715	CCAAT/enhancer-binding protein alpha	CEBPA			
O43745	Calcineurin B homologous protein 2	CHP2			
Q9P2E5	Chondroitin sulfate glucuronyltransferase	CHPF2			
Q9Y232	Chromodomain Y-like protein	CDYL			
Q6W349	Putative uncharacterized protein encoded by LINC00575	LINC00575			
Q16880	2-hydroxyacylsphingosine 1-beta-galactosyltransferase	UGT8			
Q9Y4C5	Carbohydrate sulfotransferase 2	CHST2			
Q96LL4	Uncharacterized protein C8orf48	C8orf48			
Q9BYV8	Centrosomal protein of 41 kDa	CEP41			
P0DMB2	Uncharacterized protein C8orf88	C8orf88			
Q8N0S6	Centromere protein L	CENPL			
Q76N32	Centrosomal protein of 68 kDa	CEP68			

Q9Y592	Centrosomal protein of 83 kDa	CEP83			
Q8N111	Cell cycle exit and neuronal differentiation protein 1	CEND1			
Q13740	CD166 antigen	ALCAM			
P06493	Cyclin-dependent kinase 1	CDK1			
Q8IZL9	Cyclin-dependent kinase 20	CDK20			
Q9NYV4	Cyclin-dependent kinase 12	CDK12			
Q96MD2	KICSTOR complex protein C12orf66	C12orf66			
Q96IL0	Cytochrome c oxidase assembly factor 8	COA8			
Q9BXN2	C-type lectin domain family 7 member A	CLEC7A			
Q96JB5	CDK5 regulatory subunit-associated protein 3	CDK5RAP3			
Q9UJ71	C-type lectin domain family 4 member K	CD207			
Q6UVW9	C-type lectin domain family 2 member A	CLEC2A			
P29372	DNA-3-methyladenine glycosylase	MPG			
Q5FVE4	Long-chain-fatty-acid--CoA ligase ACSBG2	ACSBG2			
A7E2S9	Putative ankyrin repeat domain-containing protein 30B-like	ANKRD30BL			
Q14738	Serine/threonine-protein phosphatase 2A 56 kDa regulatory subunit delta isoform	PPP2R5D			
P21589	5'-nucleotidase	NT5E			
P29275	Adenosine receptor A2b	ADORA2B			
Q9Y3L3	SH3 domain-binding protein 1	SH3BP1			
Q9H2F3	3 beta-hydroxysteroid dehydrogenase type 7	HSD3B7			
P51648	Aldehyde dehydrogenase family 3 member A2	ALDH3A2			
P09972	Fructose-bisphosphate aldolase C	ALDOC			
Q12904	Aminoacyl tRNA synthase complex-interacting multifunctional protein 1	AIMP1			
Q8N556	Actin filament-associated protein 1	AFAP1			
A5PLL1	Ankyrin repeat domain-containing protein 34B	ANKRD34B			
Q9BXX3	Ankyrin repeat domain-containing protein 30A	ANKRD30A			
P36405	ADP-ribosylation factor-like protein 3	ARL3			
P56559	ADP-ribosylation factor-like protein 4C	ARL4C			
P21397	Amine oxidase	MAOA			
Q7Z6G8	Ankyrin repeat and sterile alpha motif domain-containing protein 1B	ANKS1B			
O95445	Apolipoprotein M	APOM			
Q12774	Rho guanine nucleotide exchange factor 5	ARHGEF5			
P55056	Apolipoprotein C-IV	APOC4			
P02656	Apolipoprotein C-III	APOC3			
Q86VW2	Rho guanine nucleotide exchange factor 25	ARHGEF25			
Q7RTU5	Achaete-scute homolog 5	ASCL5			
P09497	Clathrin light chain B	CLTB			
Q9NY37	Acid-sensing ion channel 5	ASIC5			
Q5FYB0	Arylsulfatase J	ARSJ			
Q9H7F0	Probable cation-transporting ATPase 13A3	ATP13A3			
Q8WYN0	Cysteine protease ATG4A	ATG4A			
P24539	ATP synthase F	ATP5PB			
P51788	Chloride channel protein 2	CLCN2			

P56134	ATP synthase subunit f, mitochondrial	ATP5MF			
Q9NY35	Claudin domain-containing protein 1	CLDND1			
Q13535	Serine/threonine-protein kinase ATR	ATR			
Q9P2N4	A disintegrin and metalloproteinase with thrombospondin motifs 9	ADAMTS9			
Q8NHH9	Atlastin-2	ATL2			
Q12934	Filensin	BFSP1			
Q13895	Bystin	BYSL			
Q86VB7	Scavenger receptor cysteine-rich type 1 protein M130	CD163			
Q96P09	Baculoviral IAP repeat-containing protein 8	BIRC8			
Q13867	Bleomycin hydrolase	BLMH			
Q8IWZ6	Bardet-Biedl syndrome 7 protein	BBS7			
Q9P2W7	Galactosylgalactosylxylosylprotein 3-beta-glucuronosyltransferase 1	B3GAT1			
O43825	Beta-1,3-galactosyltransferase 2	B3GALT2			
Q8NFL0	UDP-GlcNAc:betaGal beta-1,3-N-acetylglucosaminyltransferase 7	B3GNT7			
Q7RTU4	Class A basic helix-loop-helix protein 9	BHLHA9			
O75150	E3 ubiquitin-protein ligase BRE1B	RNF40			
O14569	Cytochrome b561 domain-containing protein 2	CYB561D2			
Q6ZSI9	Calpain-12	CAPN12			
Q9NYM9	BET1-like protein	BET1L			
O95999	B-cell lymphoma/leukemia 10	BCL10			
Q8TCZ2	CD99 antigen-like protein 2	CD99L2			
Q8WVQ1	Soluble calcium-activated nucleotidase 1	CANT1			
Q9BWL3	Protein C1orf43	C1orf43			
P43234	Cathepsin O	CTSO			
Q8NEL0	Coiled-coil domain-containing protein 54	CCDC54			
Q96HJ3	Coiled-coil domain-containing protein 34	CCDC34			
O15234	Protein CASC3	CASC3			
Q8IYK2	Coiled-coil domain-containing protein 105	CCDC105			
Q7Z3S7	Voltage-dependent calcium channel subunit alpha-2/delta-4	CACNA2D4			
Q6P1N0	Coiled-coil and C2 domain-containing protein 1A	CC2D1A			
H7C350	Coiled-coil domain-containing protein 188	CCDC188			
O15467	C-C motif chemokine 16	CCL16			
P0CW27	Coiled-coil domain-containing protein 166	CCDC166			
O00585	C-C motif chemokine 21	CCL21			
A0ZSE6	Cell cycle control protein 50C	TMEM30CP			
Q6ZVT6	Protein CFAP20DC	CFAP20DC			
O60729	Dual specificity protein phosphatase CDC14B	CDC14B			
O60826	Coiled-coil domain-containing protein 22	CCDC22			
P40199	Carcinoembryonic antigen-related cell adhesion molecule 6	CEACAM6			
Q5T0N1	Cilia- and flagella-associated protein 70	CFAP70			
Q6NT52	Choriogonadotropin subunit beta variant 2	CGB2			
O96017	Serine/threonine-protein kinase Chk2	CHEK2			
P06127	T-cell surface glycoprotein CD5	CD5			
Q6YHK3	CD109 antigen	CD109			

Q53FE4	Uncharacterized protein C4orf17	C4orf17			
A7E2U8	UPF0602 protein C4orf47	C4orf47			
P32970	CD70 antigen	CD70			
Q9GZS9	Carbohydrate sulfotransferase 5	CHST5			
Q96LT7	Guanine nucleotide exchange C9orf72	C9orf72			
Q9HA82	Ceramide synthase 4	CERS4			
Q12873	Chromodomain-helicase-DNA-binding protein 3	CHD3			
Q9ULG6	Cell cycle progression protein 1	CCPG1			
Q8N7H1	Putative uncharacterized protein encoded by LINC01465	LINC01465			
P29279	CCN family member 2	CCN2			
P41002	Cyclin-F	CCNF			
A6H8M9	Cadherin-related family member	CDHR4			
Q5VV42	Threonylcarbamoyladenosine tRNA methylthiotransferase	CDKAL1			
Q8IUN9	C-type lectin domain family 10 member A	CLEC10A			
Q9ULV3	Cip1-interacting zinc finger	CIZ1			
Q17RA5	Putative uncharacterized protein C21orf62-AS1	C21orf62-AS1			
Q494W8	CHRNA7-FAM7A fusion protein	CHRFAM7A			
Q9UKF6	Cleavage and polyadenylation specificity factor subunit 3	CPSF3			
Q9Y215	Acetylcholinesterase collagenic tail peptide	COLQ			
P0DML2	Chorionic somatomammotropin hormone 1	CSH1			
Q8N6N7	Acyl-CoA-binding domain-containing protein 7	ACBD7			
Q6UXT9	Protein ABHD15	ABHD15			
Q9UGM1	Neuronal acetylcholine receptor subunit alpha-9	CHRNA9			
P13798	Acylamino-acid-releasing enzyme	APEH			
Q2M2I8	AP2-associated protein kinase 1	AAK1			
P31946	14-3-3 protein beta/alpha	YWHAB			
P78314	SH3 domain-binding protein 2	SH3BP2			
Q969T7	7-methylguanosine phosphate-specific 5'-nucleotidase	NT5C3B			
Q04771	Activin receptor type-1	ACVR1			
Q15699	ALX homeobox protein 1	ALX1			
P0CW23	A-kinase anchor protein inhibitor	AKAIN1			
Q9BUB4	tRNA-specific adenosine deaminase 1	ADAT1			
O94910	Adhesion G protein-coupled receptor L1	ADGRL1			
Q8TC27	Disintegrin and metalloproteinase domain-containing protein 32	ADAM32			
Q9H553	Alpha-1,3/1,6-mannosyltransferase ALG2	ALG2			
Q9BXX2	Ankyrin repeat domain-containing protein 30B	ANKRD30B			
P49703	ADP-ribosylation factor-like protein 4D	ARL4D			
Q7Z6R9	Transcription factor AP-2-delta	TFAP2D			
P29972	Aquaporin-1	AQP1			
P01019	Angiotensinogen	AGT			
Q495Z4	Putative uncharacterized protein ASB16-AS1	ASB16-AS1			
Q5H913	ADP-ribosylation factor-like protein 13A	ARL13A			
Q6ZW76	Ankyrin repeat and SAM domain-containing protein 3	ANKS3			
P55345	Protein arginine N-methyltransferase 2	PRMT2			
A6ND91	Putative L-aspartate dehydrogenase	ASPDH			
Q9Y294	Histone chaperone ASF1A	ASF1A			

Q9UN42	Protein ATP1B4	ATP1B4			
Q8IXJ9	Polycomb group protein ASXL1	ASXL1			
Q5T6C5	Ataxin-7-like protein 2	ATXN7L2			
P32121	Beta-arrestin-2	ARRB2			
Q8N7P3	Claudin-22	CLDN22			
Q96B33	Claudin-23	CLDN23			
C9JDP6	Putative claudin-25	CLDN25			
Q6P387	Uncharacterized protein C16orf46	C16orf46			
Q8WXS8	A disintegrin and metalloproteinase with thrombospondin motifs 14	ADAMTS14			
P36542	ATP synthase subunit gamma, mitochondrial	ATP5F1C			
O15072	A disintegrin and metalloproteinase with thrombospondin motifs 3	ADAMTS3			
O75173	A disintegrin and metalloproteinase with thrombospondin motifs 4	ADAMTS4			
Q8N100	Protein atonal homolog 7	ATOH7			
Q5VW32	BRO1 domain-containing protein BROX	BROX			
Q53HL2	Borealin	CDCA8			
Q8WZ55	Barttin	BSND			
Q9Y5Z6	Beta-1,3-galactosyltransferase 1	B3GALT1			
Q9BZE3	BarH-like 1 homeobox protein	BARHL1			
Q6W2J9	BCL-6 corepressor	BCOR			
P10415	Apoptosis regulator Bcl-2	BCL2			
O00555	Voltage-dependent P/Q-type calcium channel subunit alpha-1A	CACNA1A			
Q5SVJ3	Uncharacterized protein C1orf100	C1orf100			
Q5XLA6	Caspase recruitment domain-containing protein 17	CARD17			
Q8WUZ0	B-cell CLL/lymphoma 7 protein family member C	BCL7C			
Q96HA4	Uncharacterized protein C1orf159	C1orf159			
P14091	Cathepsin E	CTSE			
Q86UU0	B-cell CLL/lymphoma 9-like protein	BCL9L			
Q9NPY3	Complement component C1q receptor	CD93			
Q2M243	Coiled-coil domain-containing protein 27	CCDC27			
P60827	Complement C1q tumor necrosis factor-related protein 8	C1QTNF8			
Q96L12	Calreticulin-3	CALR3			
Q6P1S2	Protein C3orf33	C3orf33			
Q502W7	Coiled-coil domain-containing protein 38	CCDC38			
Q96BT1	Putative uncharacterized protein C3orf49	C3orf49			
A6NC98	Coiled-coil domain-containing protein 88B	CCDC88B			
Q8N4T8	Carbonyl reductase family member 4	CBR4			
Q5TB80	Centrosomal protein of 162 kDa	CEP162			
P59074	Putative charged multivesicular body protein 4B-like protein CHMP4BP1	CHMP4BP1			
P10809	60 kDa heat shock protein, mitochondrial	HSPD1			
Q6NUI6	Chondroadherin-like protein	CHADL			
P00746	Complement factor D	CFD			
Q9UHN6	Cell surface hyaluronidase	CEMIP2			
P08218	Chymotrypsin-like elastase family member 2B	CELA2B			
O76039	Cyclin-dependent kinase-like 5	CDKL5			
Q49AR2	UPF0489 protein C5orf22	C5orf22			

Q49AH0	Cerebral dopamine neurotrophic factor	CDNF			
Q52M75	Putative uncharacterized protein encoded by LINC01554	LINC01554			
Q96LM5	Uncharacterized protein C4orf45	C4orf45			
O95813	Cerberus	CER1			
Q86X52	Chondroitin sulfate synthase 1	CHSY1			
P49336	Cyclin-dependent kinase 8	CDK8			
Q8WUJ3	Cell migration-inducing and hyaluronan-binding protein	CEMIP			
P41208	Centrin-2	CETN2			
P50750	Cyclin-dependent kinase 9	CDK9			
Q8N5K1	CDGSH iron-sulfur domain-containing protein 2	CISD2			
P20674	Cytochrome c oxidase subunit 5A, mitochondrial	COX5A			
P10635	Cytochrome P450 2D6	CYP2D6			
Q9UH73	Transcription factor COE1	EBF1			
P50238	Cysteine-rich protein 1	CRIP1			
P20815	Cytochrome P450 3A5	CYP3A5			
O75534	Cold shock domain-containing protein E1	CSDE1			
Q9BV23	Monoacylglycerol lipase ABHD6	ABHD6			
Q8NE71	ATP-binding cassette sub-family F member 1	ABCF1			
P05067	Amyloid-beta precursor protein	APP			
O14639	Actin-binding LIM protein 1	ABLIM1			
Q9UNQ0	Broad substrate specificity ATP-binding cassette transporter ABCG2	ABCG2			
Q8TF27	Arf-GAP with GTPase, ANK repeat and PH domain-containing protein 11	AGAP11			
Q96HD9	N-acyl-aromatic-L-amino acid amidohydrolase	ACY3			
P63267	Actin, gamma-enteric smooth muscle	ACTG2			
P35858	Insulin-like growth factor-binding protein complex acid labile subunit	IGFALS			
P04075	Fructose-bisphosphate aldolase A	ALDOA			
O95081	Arf-GAP domain and FG repeat-containing protein 2	AGFG2			
Q04828	Aldo-keto reductase family 1 member C1	AKR1C1			
Q6NVV9	Putative disintegrin and metalloproteinase domain-containing protein 5	ADAM5			
Q9BY15	Adhesion G protein-coupled receptor E3	ADGRE3			
Q8NEB7	Acrosin-binding protein	ACRBP			
Q6UX46	ALK and LTK ligand 2	ALKAL2			
Q8IZF2	Adhesion G protein-coupled receptor F5	ADGRF5			
Q9BT22	Chitobiosyldiphosphodolichol beta-mannosyltransferase	ALG1			
Q9UKF5	Disintegrin and metalloproteinase domain-containing protein 29	ADAM29			
Q9BV10	Dol-P-Man:Man	ALG12			
Q8NCL9	Protein APCDD1-like	APCDD1L			
Q6UB98	Ankyrin repeat domain-containing protein 12	ANKRD12			
Q9BXS5	AP-1 complex subunit mu-1	AP1M1			
Q06278	Aldehyde oxidase	AOX1			
Q9H6X2	Anthrax toxin receptor 1	ANTXR1			
A5YM69	Rho guanine nucleotide exchange factor 35	ARHGEF35			
Q16853	Membrane primary amine oxidase	AOC3			
Q96CW1	AP-2 complex subunit mu	AP2M1			

Q96IT6	Putative uncharacterized protein ARHGAP5-AS1	ARHGAP5-AS1			
Q14865	AT-rich interactive domain-containing protein 5B	ARID5B			
Q76L83	Putative Polycomb group protein ASXL2	ASXL2			
Q9NVP2	Histone chaperone ASF1B	ASF1B			
O15144	Actin-related protein 2/3 complex subunit 2	ARPC2			
Q9UBB4	Ataxin-10	ATXN10			
P56746	Claudin-15	CLDN15			
P56856	Claudin-18	CLDN18			
Q96IX5	ATP synthase membrane subunit DAPIT, mitochondrial	ATP5MD			
Q8WXX7	Autism susceptibility gene 2 protein	AUTS2			
Q6UWD8	Transmembrane protein C16orf54	C16orf54			
Q8NHY2	E3 ubiquitin-protein ligase COP1	COP1			
Q9UKP4	A disintegrin and metalloproteinase with thrombospondin motifs 7	ADAMTS7			
Q9UNA0	A disintegrin and metalloproteinase with thrombospondin motifs 5	ADAMTS5			
P54710	Sodium/potassium-transporting ATPase subunit gamma	FXYD2			
O00327	Aryl hydrocarbon receptor nuclear translocator-like protein 1	ARNTL			
Q9P203	BTB/POZ domain-containing protein 7	BTBD7			
Q14692	Ribosome biogenesis protein BMS1 homolog	BMS1			
P17213	Bactericidal permeability-increasing protein	BPI			
Q8WYA1	Aryl hydrocarbon receptor nuclear translocator-like protein 2	ARNTL2			
Q8ND07	Basal body-orientation factor 1	BBOF1			
Q9UMX3	Bcl-2-related ovarian killer protein	BOK			
Q6ZN30	Zinc finger protein basonuclin-2	BNC2			
O75936	Gamma-butyrobetaine dioxygenase	BBOX1			
O94766	Galactosylgalactosylxylosylprotein 3-beta-glucuronosyltransferase 3	B3GAT3			
Q9UHR4	Brain-specific angiogenesis inhibitor 1-associated protein 2-like protein 1	BAIAP2L1			
Q8IYS8	Biorientation of chromosomes in cell division protein 1-like 2	BOD1L2			
Q6MZZ7	Calpain-13	CAPN13			
O15155	BET1 homolog	BET1			
Q8N7W2	BEN domain-containing protein 7	BEND7			
A8MZ97	Uncharacterized protein C2orf74	C2orf74			
O15484	Calpain-5	CAPN5			
Q8IXH8	Cadherin-like protein 26	CDH26			
Q8N1Q1	Carbonic anhydrase 13	CA13			
Q14781	Chromobox protein homolog 2	CBX2			
A5D8W1	Cilia- and flagella-associated protein 69	CFAP69			
P15088	Mast cell carboxypeptidase A	CPA3			
P09093	Chymotrypsin-like elastase family member 3A	CELA3A			
P32248	C-C chemokine receptor type 7	CCR7			
Q9BZW8	Natural killer cell receptor 2B4	CD244			
Q8WUH1	Protein Churchill	CHURC1			
P31358	CAMPATH-1 antigen	CD52			
P06729	T-cell surface antigen CD2	CD2			
P08962	CD63 antigen	CD63			
P13987	CD59 glycoprotein	CD59			
Q12860	Contactin-1	CNTN1			

P40259	B-cell antigen receptor complex-associated protein beta chain	CD79B			
Q9Y6Z2	Uncharacterized protein encoded by LINC01558	LINC01558			
Q32Q52	Uncharacterized protein C12orf74	C12orf74			
P25067	Collagen alpha-2	COL8A2			
Q5U649	Uncharacterized protein C12orf60	C12orf60			
Q9NPU4	Uncharacterized protein C14orf132	C14orf132			
Q8N326	Putative uncharacterized protein RPP38-DT	RPP38-DT			
Q5VZT2	Putative uncharacterized protein C10orf113	C10orf113			
P18545	Retinal rod rhodopsin-sensitive cGMP 3',5'-cyclic phosphodiesterase subunit gamma	PDE6G			
P12109	Collagen alpha-1	COL6A1			
P0C7M8	C-type lectin domain family 2 member L	CLEC2L			
Q8N0X4	Citramalyl-CoA lyase, mitochondrial	CLYBL			
Q5K131	Chronic lymphocytic leukemia up-regulated protein 1	CLLU1			
O15516	Circadian locomoter output cycles protein kaput	CLOCK			
P09496	Clathrin light chain A	CLTA			
P61923	Coatomer subunit zeta-1	COPZ1			
P10176	Cytochrome c oxidase subunit 8A, mitochondrial	COX8A			
H7BZ55	Putative ciliary rootlet coiled-coil protein 2	CROCC2			
Q5T5F5	Uncharacterized protein ADAMTSL4-AS1	ADAMTSL4-AS1			
Q5TYW2	Ankyrin repeat domain-containing protein 20A1	ANKRD20A1			
A5X5Y0	5-hydroxytryptamine receptor 3E	HTR3E			
P32297	Neuronal acetylcholine receptor subunit alpha-3	CHRNA3			
Q9NRG9	Aladin	AAAS			
P17174	Aspartate aminotransferase, cytoplasmic	GOT1			
Q96EY9	Probable inactive tRNA-specific adenosine deaminase-like protein 3	ADAT3			
P63104	14-3-3 protein zeta/delta	YWHAZ			
P31941	DNA dC->dU-editing enzyme APOBEC-3A	APOBEC3A			
P55008	Allograft inflammatory factor 1	AIF1			
P36896	Activin receptor type-1B	ACVR1B			
P07327	Alcohol dehydrogenase 1A	ADH1A			
P48448	Aldehyde dehydrogenase family 3 member B2	ALDH3B2			
P08319	All-trans-retinol dehydrogenase	ADH4			
Q16352	Alpha-internexin	INA			
Q8TD30	Alanine aminotransferase 2	GPT2			
Q7Z591	Microtubule organization protein AKNA	AKNA			
Q12802	A-kinase anchor protein 13	AKAP13			
Q9NR19	Acetyl-coenzyme A synthetase, cytoplasmic	ACSS2			
P23109	AMP deaminase 1	AMPD1			
Q6P093	Arylacetamide deacetylase-like 2	AADACL2			
Q5T1N1	Protein AKNAD1	AKNAD1			
P51826	AF4/FMR2 family member 3	AFF3			
Q7Z695	Uncharacterized aarF domain-containing protein kinase 2	ADCK2			

Q96F25	UDP-N-acetylglucosamine transferase subunit ALG14 homolog	ALG14			
Q4VCS5	Angiomotin	AMOT			
P55198	Protein AF-17	MLLT6			
Q8IZF4	Adhesion G-protein coupled receptor G5	ADGRG5			
Q9BZZ5	Apoptosis inhibitor 5	API5			
P84085	ADP-ribosylation factor 5	ARF5			
Q6ZN18	Zinc finger protein AEBP2	AEBP2			
P58335	Anthrax toxin receptor 2	ANTXR2			
Q9H0F7	ADP-ribosylation factor-like protein 6	ARL6			
P29374	AT-rich interactive domain-containing protein 4A	ARID4A			
A8K4G0	CMRF35-like molecule 7	CD300LB			
Q8WXK3	Ankyrin repeat and SOCS box protein 13	ASB13			
Q93084	Sarcoplasmic/endoplasmic reticulum calcium ATPase 3	ATP2A3			
Q9H9F9	Actin-related protein 5	ACTR5			
P05023	Sodium/potassium-transporting ATPase subunit alpha-1	ATP1A1			
Q16720	Plasma membrane calcium-transporting ATPase 3	ATP2B3			
Q6PL18	ATPase family AAA domain-containing protein 2	ATAD2			
P48201	ATP synthase F	ATP5MC3			
P17735	Tyrosine aminotransferase	TAT			
Q99766	ATP synthase subunit s, mitochondrial	DMAC2L			
P0C7T5	Ataxin-1-like	ATXN1L			
O00501	Claudin-5	CLDN5			
Q8NBF6	Late secretory pathway protein AVL9 homolog	AVL9			
Q5T1B0	Axonemal dynein light chain domain-containing protein 1	AXDND1			
Q6E213	Acyl-CoA wax alcohol acyltransferase 2	AWAT2			
Q5VT79	Annexin A8-like protein 1	ANXA8L1			
Q9Y2T1	Axin-2	AXIN2			
Q8TE60	A disintegrin and metalloproteinase with thrombospondin motifs 18	ADAMTS18			
P56385	ATP synthase subunit e, mitochondrial	ATP5ME			
Q6QNY1	Biogenesis of lysosome-related organelles complex 1 subunit 2	BLOC1S2			
Q13873	Bone morphogenetic protein receptor type-2	BMPR2			
Q8NFQ5	BPI fold-containing family B member 6	BPIFB6			
Q8TDL5	BPI fold-containing family B member 1	BPIFB1			
Q8N1L9	Basic leucine zipper transcriptional factor ATF-like 2	BATF2			
Q9BUT1	3-hydroxybutyrate dehydrogenase type 2	BDH2			
Q8NFU1	Bestrophin-2	BEST2			
Q5VU97	VWFA and cache domain-containing protein 1	CACHD1			
O00305	Voltage-dependent L-type calcium channel subunit beta-4	CACNB4			
Q8N143	B-cell CLL/lymphoma 6 member B protein	BCL6B			
Q4VC05	B-cell CLL/lymphoma 7 protein family member A	BCL7A			
Q13634	Cadherin-18	CDH18			
P55291	Cadherin-15	CDH15			

Q8IYL3	UPF0688 protein C1orf174	C1orf174			
Q08AD1	Calmodulin-regulated spectrin-associated protein 2	CAMSAP2			
Q96F63	Coiled-coil domain-containing protein 97	CCDC97			
Q9H0W5	Coiled-coil domain-containing protein 8	CCDC8			
Q16204	Coiled-coil domain-containing protein 6	CCDC6			
Q8TD31	Coiled-coil alpha-helical rod protein 1	CCHCR1			
O14958	Calsequestrin-2	CASQ2			
Q8N9Z2	Coiled-coil domain-containing protein 71L	CCDC71L			
P13236	C-C motif chemokine 4	CCL4			
O94986	Centrosomal protein of 152 kDa	CEP152			
Q5TEZ4	Putative uncharacterized protein encoded by LINC01590	LINC01590			
Q9BY43	Charged multivesicular body protein 4a	CHMP4A			
P53567	CCAAT/enhancer-binding protein gamma	CEBPG			
P08217	Chymotrypsin-like elastase family member 2A	CELA2A			
P30260	Cell division cycle protein 27 homolog	CDC27			
Q9HCU0	Endosialin	CD248			
P26842	CD27 antigen	CD27			
Q92903	Phosphatidate cytidylyltransferase 1	CDS1			
Q8N6G5	Chondroitin sulfate N-acetylgalactosaminyltransferase 2	CSGALNACT2			
P42773	Cyclin-dependent kinase 4 inhibitor C	CDKN2C			
C9J302	Uncharacterized protein C4orf51	C4orf51			
A6NGY3	Uncharacterized protein C5orf52	C5orf52			
O60308	Centrosomal protein of 104 kDa	CEP104			
Q5T280	Putative methyltransferase C9orf114	SPOUT1			
Q6P047	Uncharacterized protein C8orf74	C8orf74			
A8MXV6	CMT1A duplicated region transcript 15 protein-like protein	CDRT15L2			
Q8N6U2	Putative uncharacterized protein encoded by LINC00612	LINC00612			
P08571	Monocyte differentiation antigen CD14	CD14			
Q9H3J6	Probable peptide chain release factor C12orf65, mitochondrial	C12orf65			
Q96LP6	Uncharacterized protein C12orf42	C12orf42			
P32246	C-C chemokine receptor type 1	CCR1			
Q5QGZ9	C-type lectin domain family 12 member A	CLEC12A			
P02748	Complement component C9	C9			
Q8NAA6	Putative uncharacterized protein encoded by LINC02694	LINC02694			
Q8N999	Uncharacterized protein C12orf29	C12orf29			
A8K5M9	Uncharacterized protein C15orf62, mitochondrial	C15orf62			
P16562	Cysteine-rich secretory protein 2	CRISP2			
Q8IUY3	GRAM domain-containing protein 2A	GRAMD2A			
Q58FF6	Putative heat shock protein HSP 90-beta 4	HSP90AB4P			
P09341	Growth-regulated alpha protein	CXCL1			
Q8IUC2	Keratin-associated protein 8-1	KRTAP8-1			
Q9Y241	HIG1 domain family member 1A, mitochondrial	HIGD1A			
Q93099	Homogentisate 1,2-dioxygenase	HGD			
Q9HCC6	Transcription factor HES-4	HES4			

Q14469	Transcription factor HES-1	HES1			
Q9H8Q6	Putative uncharacterized protein encoded by HEXA-AS1	HEXA-AS1			
P17509	Homeobox protein Hox-B6	HOXB6			
Q92826	Homeobox protein Hox-B13	HOXB13			
A0A0A0MS14	Immunoglobulin heavy variable 1-45	IGHV1-45			
P0DMV8	Heat shock 70 kDa protein 1A	HSPA1A			
P19622	Homeobox protein engrailed-2	EN2			
Q9UJC3	Protein Hook homolog 1	HOOK1			
P26583	High mobility group protein B2	HMGB2			
B2RXH8	Heterogeneous nuclear ribonucleoprotein C-like 2	HNRNPCL2			
Q8IZJ0	Interferon lambda-2	IFNL2			
O60573	Eukaryotic translation initiation factor 4E type 2	EIF4E2			
P01871	Immunoglobulin heavy constant mu	IGHM			
Q9GZX6	Interleukin-22	IL22			
Q969P0	Immunoglobulin superfamily member 8	IGSF8			
P22692	Insulin-like growth factor-binding protein 4	IGFBP4			
O60725	Protein-S-isoprenylcysteine O-methyltransferase	ICMT			
Q9UPT6	C-Jun-amino-terminal kinase-interacting protein 3	MAPK8IP3			
P13612	Integrin alpha-4	ITGA4			
Q96J02	E3 ubiquitin-protein ligase Itchy homolog	ITCH			
P04259	Keratin, type II cytoskeletal 6B	KRT6B			
Q2LD37	Transmembrane protein	KIAA1109			
Q8TEX9	Importin-4	IPO4			
P22459	Potassium voltage-gated channel subfamily A member 4	KCNA4			
Q96CX2	BTB/POZ domain-containing protein KCTD12	KCTD12			
Q9BY66	Lysine-specific demethylase 5D	KDM5D			
Q3SYF9	Keratin-associated protein 19-7	KRTAP19-7			
P07195	L-lactate dehydrogenase B chain	LDHB			
Q96FV0	Leucine-rich repeat-containing protein 46	LRRC46			
Q8TCA0	Leucine-rich repeat-containing protein 20	LRRC20			
Q15048	Leucine-rich repeat-containing protein 14	LRRC14			
Q8N485	Protein limb expression 1	LIX1			
Q86Y78	Ly6/PLAUR domain-containing protein 6	LYPD6			
Q8IXW0	Lamin tail domain-containing protein 2	LMNTD2			
Q99732	Lipopolysaccharide-induced tumor necrosis factor-alpha factor	LITAF			
Q6UB28	Methionine aminopeptidase 1D, mitochondrial	METAP1D			
P61968	LIM domain transcription factor LMO4	LMO4			
Q8IWU2	Serine/threonine-protein kinase LMTK2	LMTK2			
Q7Z434	Mitochondrial antiviral-signaling protein	MAVS			
C9JSJ3	Meiosis initiator protein	MEIOSIN			
Q16655	Melanoma antigen recognized by T-cells 1	MLANA			
P41279	Mitogen-activated protein kinase kinase kinase 8	MAP3K8			
Q96DS6	Membrane-spanning 4-domains subfamily A member 6E	MS4A6E			

Q9P0L2	Serine/threonine-protein kinase MARK1	MARK1			
Q8NDY4	Myelodysplastic syndrome 2 translocation-associated protein	MDS2			
Q86UE6	Leucine-rich repeat transmembrane neuronal protein 1	LRRTM1			
Q96JY0	Protein maelstrom homolog	MAEL			
A6NJ78	12S rRNA N4-methylcytidine	METTL15			
Q9BXW4	Microtubule-associated proteins 1A/1B light chain 3C	MAP1LC3C			
Q8N4P3	Guanosine-3',5'-bis	HDDC3			
Q6NVV0	Putative makorin-5	MKRN9P			
Q8TCB7	tRNA N	METTL6			
L0R8F8	MIEF1 upstream open reading frame protein	MIEF1			
Q8NCR3	Protein MFI	MFI			
Q504T8	Midnolin	MIDN			
Q5U5Q3	RNA-binding E3 ubiquitin-protein ligase MEX3C	MEX3C			
Q96T58	Msx2-interacting protein	SPEN			
O75121	Microfibrillar-associated protein 3-like	MFAP3L			
P20645	Cation-dependent mannose-6-phosphate receptor	M6PR			
O95297	Myelin protein zero-like protein 1	MPZL1			
Q96LB0	Mas-related G-protein coupled receptor member X3	MRGPRX3			
O15375	Monocarboxylate transporter 6	SLC16A5			
Q8IWI9	MAX gene-associated protein	MGA			
P15941	Mucin-1	MUC1			
O43312	Protein MTSS 1	MTSS1			
Q2M296	Methenyltetrahydrofolate synthase domain-containing protein	MTHFSD			
Q13505	Metaxin-1	MTX1			
A0A0U1RRE5	Negative regulator of P-body association	NBDY			
Q8IZF0	Sodium leak channel non-selective protein	NALCN			
Q6NZ67	Mitotic-spindle organizing protein 2B	MZT2B			
B0I1T2	Unconventional myosin-Ig	MYO1G			
Q14324	Myosin-binding protein C, fast-type	MYBPC2			
P0CAP1	Myocardial zonula adherens protein	MYZAP			
Q86YV6	Myosin light chain kinase family member 4	MYLK4			
Q17RQ9	NTPase KAP family P-loop domain-containing protein 1	NKPD1			
P01185	Vasopressin-neurophysin 2-copeptin	AVP			
Q86Y39	NADH dehydrogenase	NDUFA11			
Q8NG66	Serine/threonine-protein kinase Nek11	NEK11			
Q9GZP1	Neurensin-2	NRSN2			
Q9BSD7	Cancer-related nucleoside-triphosphatase	NTPCR			
Q7Z4S6	Kinesin-like protein KIF21A	KIF21A			
Q52LG2	Keratin-associated protein 13-2	KRTAP13-2			
P29622	Kallistatin	SERPINA4			
Q86UK5	Limbin	EVC2			
Q5T7P3	Late cornified envelope protein 1B	LCE1B			
O95970	Leucine-rich glioma-inactivated protein 1	LGI1			
Q569H4	Protein Largen	PRR16			
Q8N6Y2	Leucine-rich repeat-containing protein 17	LRRC17			

Q96QE4	Leucine-rich repeat-containing protein 37B	LRRC37B			
Q9HCJ2	Leucine-rich repeat-containing protein 4C	LRRC4C			
Q6PJG9	Leucine-rich repeat and fibronectin type-III domain-containing protein 4	LRFN4			
Q5JTD7	Leucine-rich repeat-containing protein 73	LRRC73			
Q9NQ29	Putative RNA-binding protein Luc7-like 1	LUC7L			
P16581	E-selectin	SELE			
P33241	Lymphocyte-specific protein 1	LSP1			
Q16706	Alpha-mannosidase 2	MAN2A1			
P53582	Methionine aminopeptidase 1	METAP1			
O60476	Mannosyl-oligosaccharide 1,2-alpha-mannosidase IB	MAN1A2			
Q6ZRQ5	Protein MMS22-like	MMS22L			
O15457	MutS protein homolog 4	MSH4			
Q10713	Mitochondrial-processing peptidase subunit alpha	PMPCA			
Q86VF5	2-acylglycerol O-acyltransferase 3	MOGAT3			
A0A3B3IT52	Putative uncharacterized protein MSANTD5	MSANTD5			
Q99583	Max-binding protein MNT	MNT			
P0C860	Putative male-specific lethal-3 protein-like 2	MSL3P1			
Q86SM5	Mas-related G-protein coupled receptor member G	MRGPRG			
Q9NZJ7	Mitochondrial carrier homolog 1	MTCH1			
Q9ULD2	Microtubule-associated tumor suppressor 1	MTUS1			
Q9Y4B5	Microtubule cross-linking factor 1	MTCL1			
Q6NSW7	Homeobox protein NANOGP8	NANOGP8			
Q9BTE0	N-acetyltransferase 9	NAT9			
P41271	Neuroblastoma suppressor of tumorigenicity 1	NBL1			
P10243	Myb-related protein A	MYBL1			
Q9Y6C9	Mitochondrial carrier homolog 2	MTCH2			
Q9H1M0	Nucleoporin-62 C-terminal-like protein	NUP62CL			
P50539	Max-interacting protein 1	MXI1			
Q13506	NGFI-A-binding protein 1	NAB1			
P23409	Myogenic factor 6	MYF6			
Q86UY6	N-alpha-acetyltransferase 40	NAA40			
Q8WX92	Negative elongation factor B	NELFB			
A0A5F9ZHS7	NFIL3 like protein	NFILZ			
P08651	Nuclear factor 1 C-type	NFIC			
Q00604	Norrin	NDP			
Q504Y2	Extracellular tyrosine-protein kinase PKDCC	PKDCC			
Q9NTG1	Polycystic kidney disease and receptor for egg jelly-related protein	PKDREJ			
Q16649	Nuclear factor interleukin-3-regulated protein	NFIL3			
P36873	Serine/threonine-protein phosphatase PP1-gamma catalytic subunit	PPP1CC			
Q9NWW9	Phospholipase A and acyltransferase 2	PLAAT2			
A8MXV4	Nucleoside diphosphate-linked moiety X motif 19	NUDT19			
Q96EP9	Sodium/bile acid cotransporter 4	SLC10A4			
Q96RS6	NudC domain-containing protein 1	NUDCD1			
Q9NWW6	Nicotinamide riboside kinase 1	NMRK1			
Q9Y2I2	Netrin-G1	NTNG1			

O60285	NUAK family SNF1-like kinase 1	NUAK1			
Q6X4W1	NMDA receptor synaptonuclear signaling and neuronal migration factor	NSMF			
Q8NGB6	Olfactory receptor 4M2	OR4M2			
Q8NGS2	Olfactory receptor 1J2	OR1J2			
Q8NH50	Olfactory receptor 8K5	OR8K5			
P0CE72	Oncomodulin-1	OCM			
A6NMU1	Olfactory receptor 52A4	OR52A4P			
Q969Q6	Serine/threonine-protein phosphatase 2A regulatory subunit B'' subunit gamma	PPP2R3C			
Q9H339	Olfactory receptor 51B5	OR51B5			
Q8NGS8	Olfactory receptor 13C5	OR13C5			
Q8NGJ8	Olfactory receptor 51S1	OR51S1			
Q6IFG1	Olfactory receptor 52E8	OR52E8			
Q8NGP9	Olfactory receptor 5AR1	OR5AR1			
Q8NGQ4	Olfactory receptor 10Q1	OR10Q1			
Q8N127	Olfactory receptor 5AS1	OR5AS1			
Q9GZK7	Olfactory receptor 11A1	OR11A1			
O95747	Serine/threonine-protein kinase OSR1	OXSR1			
P61366	Osteocrin	OSTN			
Q15257	Serine/threonine-protein phosphatase 2A activator	PTPA			
O00408	cGMP-dependent 3',5'-cyclic phosphodiesterase	PDE2A			
Q56P42	Pyrin domain-containing protein 2	PYDC2			
Q8NGH6	Putative olfactory receptor 52L2	OR52L2P			
Q8NCC3	Phospholipase A2 group XV	PLA2G15			
P40426	Pre-B-cell leukemia transcription factor 3	PBX3			
Q15366	Poly	PCBP2			
P25116	Proteinase-activated receptor 1	F2R			
O14832	Phytanoyl-CoA dioxygenase, peroxisomal	PHYH			
Q9BZM2	Group IIF secretory phospholipase A2	PLA2G2F			
Q9Y5E4	Protocadherin beta-5	PCDHB5			
Q9UN75	Protocadherin alpha-12	PCDHA12			
Q6KB66	Keratin, type II cytoskeletal 80	KRT80			
P46013	Proliferation marker protein Ki-67	MKI67			
Q9NQT8	Kinesin-like protein KIF13B	KIF13B			
Q96T54	Potassium channel subfamily K member 17	KCNK17			
Q9BYP8	Keratin-associated protein 17-1	KRTAP17-1			
P60411	Keratin-associated protein 10-9	KRTAP10-9			
Q9H2S1	Small conductance calcium-activated potassium channel protein 2	KCNN2			
Q3LI59	Keratin-associated protein 21-2	KRTAP21-2			
P23276	Kell blood group glycoprotein	KEL			
Q13887	Krueppel-like factor 5	KLF5			
O60303	Katanin-interacting protein	KATNIP			
Q8NEZ4	Histone-lysine N-methyltransferase 2C	KMT2C			
P06732	Creatine kinase M-type	CKM			
P42167	Lamina-associated polypeptide 2, isoforms beta/gamma	TMPO			
Q5T753	Late cornified envelope protein 1E	LCE1E			
Q9BRS8	La-related protein 6	LARP6			
Q5T754	Late cornified envelope protein 1F	LCE1F			
Q05C16	Leucine-rich repeat-containing protein 63	LRRC63			
Q8ND94	LRRN4 C-terminal-like protein	LRRN4CL			
Q8ND56	Protein LSM14 homolog A	LSM14A			
Q8ND30	Liprin-beta-2	PPFIBP2			
Q9NZU5	LIM and cysteine-rich domains protein 1	LMCD1			

Q68DH5	G-protein coupled receptor-associated protein LMBRD2	LMBRD2			
Q9BUE0	Mediator of RNA polymerase II transcription subunit 18	MED18			
Q6P9B6	MTOR-associated protein MEAK7	MEAK7			
Q9Y5V3	Melanoma-associated antigen D1	MAGED1			
O00187	Mannan-binding lectin serine protease 2	MASP2			
O95243	Methyl-CpG-binding domain protein 4	MBD4			
Q0VG99	Mesoderm posterior protein 2	MESP2			
Q9BUU2	Methyltransferase-like protein 22	METTL22			
P55082	Microfibril-associated glycoprotein 3	MFAP3			
O95396	Adenylyltransferase and sulfurtransferase MOCS3	MOCS3			
Q16653	Myelin-oligodendrocyte glycoprotein	MOG			
Q9Y5U8	Mitochondrial pyruvate carrier 1	MPC1			
O15403	Monocarboxylate transporter 7	SLC16A6			
P98088	Mucin-5AC	MUC5AC			
Q6IQ20	N-acyl-phosphatidylethanolamine-hydrolyzing phospholipase D	NAPEPLD			
Q9Y303	N-acetylglucosamine-6-phosphate deacetylase	AMDHD2			
Q8WX94	NACHT, LRR and PYD domains-containing protein 7	NLRP7			
Q99417	c-Myc-binding protein	MYCBP			
Q92802	NEDD4-binding protein 2-like 2	N4BP2L2			
Q9UHQ1	Nuclear prelamin A recognition factor	NARF			
P53602	Diphosphomevalonate decarboxylase	MVD			
Q6N069	N-alpha-acetyltransferase 16, NatA auxiliary subunit	NAA16			
P35580	Myosin-10	MYH10			
O43639	Cytoplasmic protein NCK2	NCK2			
Q86T75	Neuroblastoma breakpoint family member 11	NBPF11			
O00217	NADH dehydrogenase	NDUFS8			
Q8N8Q9	Magnesium transporter NIPA2	NIPA2			
Q86UT6	NLR family member X1	NLRX1			
O95096	Homeobox protein Nkx-2.2	NKX2-2			
P30414	NK-tumor recognition protein	NKTR			
P01138	Beta-nerve growth factor	NGF			
O00624	Sodium-dependent phosphate transport protein 3	SLC17A2			
P08949	Neuromedin-B	NMB			
O43795	Unconventional myosin-Ib	MYO1B			
Q7L592	Protein arginine methyltransferase NDUFAF7, mitochondrial	NDUFAF7			
O95298	NADH dehydrogenase	NDUFC2			
O43677	NADH dehydrogenase	NDUFC1			
P07196	Neurofilament light polypeptide	NEFL			
P55055	Oxysterols receptor LXR-beta	NR1H2			
A1L188	NADH dehydrogenase	NDUFAF8			
Q9Y466	Nuclear receptor subfamily 2 group E member 1	NR2E1			
Q9BZE4	Nucleolar GTP-binding protein 1	GTPBP4			
P0CG21	NHL-repeat-containing protein 4	NHLRC4			
Q12857	Nuclear factor 1 A-type	NFIA			
O00712	Nuclear factor 1 B-type	NFIB			
Q9H1E3	Nuclear ubiquitous casein and cyclin-dependent kinase substrate 1	NUCKS1			
P30989	Neurotensin receptor type 1	NTSR1			
Q9P2S2	Neurexin-2	NRXN2			
P58401	Neurexin-2-beta	NRXN2			
Q96AT1	Uncharacterized protein KIAA1143	KIAA1143			

O43187	Interleukin-1 receptor-associated kinase-like 2	IRAK2			
Q7Z794	Keratin, type II cytoskeletal 1b	KRT77			
Q4VXA5	KH homology domain-containing protein 1	KHDC1			
P38484	Interferon gamma receptor 2	IFNGR2			
Q9Y6J6	Potassium voltage-gated channel subfamily E member 2	KCNE2			
Q2M2Z5	Centrosomal protein kizuna	KIZ			
Q9UKQ9	Kallikrein-9	KLK9			
Q9H079	KATNB1-like protein 1	KATNBL1			
P01619	Immunoglobulin kappa variable 3-20	IGKV3-20			
P50748	Kinetochore-associated protein 1	KNTC1			
Q6ISS4	Leukocyte-associated immunoglobulin-like receptor 2	LAIR2			
P18627	Lymphocyte activation gene 3 protein	LAG3			
Q9H6V9	Lipid droplet-associated hydrolase	LDAH			
P15018	Leukemia inhibitory factor	LIF			
Q8WVC0	RNA polymerase-associated protein LEO1	LEO1			
O60711	Leupaxin	LPXN			
Q5T3J3	Ligand-dependent nuclear receptor-interacting factor 1	LRIF1			
Q5VYY2	Lipase member M	LIPM			
O94772	Lymphocyte antigen 6H	LY6H			
Q5VXJ0	Lipase member K	LIPK			
Q17RR3	Pancreatic lipase-related protein 3	PNLIPRP3			
P01715	Immunoglobulin lambda variable 3-1	IGLV3-1			
A0A075B6K2	Immunoglobulin lambda variable 3-12	IGLV3-12			
P46821	Microtubule-associated protein	MAP1B			
Q06413	Myocyte-specific enhancer factor 2C	MEF2C			
Q14680	Maternal embryonic leucine zipper kinase	MELK			
Q12852	Mitogen-activated protein kinase kinase kinase 12	MAP3K12			
Q15691	Microtubule-associated protein RP/EB family member 1	MAPRE1			
Q969Z3	Mitochondrial amidoxime reducing component 2	MTARC2			
Q15528	Mediator of RNA polymerase II transcription subunit 22	MED22			
Q9NQG1	Protein MANBAL	MANBAL			
Q9P2E8	E3 ubiquitin-protein ligase MARCHF4	MARCHF4			
Q9ULK4	Mediator of RNA polymerase II transcription subunit 23	MED23			
Q68D91	Metallo-beta-lactamase domain-containing protein 2	MBLAC2			
Q8IZK6	Mucolipin-2	MCOLN2			
Q9Y4K4	Mitogen-activated protein kinase kinase kinase kinase 5	MAP4K5			
Q96DN6	Methyl-CpG-binding domain protein 6	MBD6			
Q5JXC2	Migration and invasion-inhibitory protein	MIIP			
Q9ULZ9	Matrix metalloproteinase-17	MMP17			
Q00266	S-adenosylmethionine synthase isoform type-1	MAT1A			
Q7RTX9	Monocarboxylate transporter 14	SLC16A14			
Q14432	cGMP-inhibited 3',5'-cyclic phosphodiesterase A	PDE3A			
P04731	Metallothionein-1A	MT1A			
Q02505	Mucin-3A	MUC3A			

Q9NR99	Matrix-remodeling-associated protein 5	MXRA5			
Q00872	Myosin-binding protein C, slow-type	MYBPC1			
Q13402	Unconventional myosin-VIIa	MYO7A			
Q15596	Nuclear receptor coactivator 2	NCOA2			
P60660	Myosin light polypeptide 6	MYL6			
Q92982	Ninjurin-1	NINJ1			
Q6ZUT1	Uncharacterized protein NKAPD1	NKAPD1			
Q99801	Homeobox protein Nkx-3.1	NKX3-1			
Q6KC79	Nipped-B-like protein	NIPBL			
Q9UBE8	Serine/threonine-protein kinase NLK	NLK			
Q92692	Nectin-2	NECTIN2			
O95502	Neuronal pentraxin receptor	NPTXR			
Q15223	Nectin-1	NECTIN1			
O60287	Nucleolar pre-ribosomal-associated protein 1	URB1			
O95168	NADH dehydrogenase	NDUFB4			
Q9H7X0	N-alpha-acetyltransferase 60	NAA60			
Q9NV92	NEDD4 family-interacting protein 2	NDFIP2			
P49146	Neuropeptide Y receptor type 2	NPY2R			
Q8IXF0	Neuronal PAS domain-containing protein 3	NPAS3			
Q8N729	Neuropeptide W	NPW			
O43920	NADH dehydrogenase	NDUFS5			
Q14994	Nuclear receptor subfamily 1 group I member 3	NR1I3			
Q9Y5X4	Photoreceptor-specific nuclear receptor	NR2E3			
Q9Y239	Nucleotide-binding oligomerization domain-containing protein 1	NOD1			
Q8WUJ1	Neuferricin	CYB5D2			
Q53H76	Phospholipase A1 member A	PLA1A			
P14923	Junction plakoglobin	JUP			
Q96I34	Protein phosphatase 1 regulatory subunit 16A	PPP1R16A			
P48736	Phosphatidylinositol 4,5-bisphosphate 3-kinase catalytic subunit gamma isoform	PIK3CG			
Q5JTB6	Placenta-specific protein 9	PLAC9			
Q9NXH3	Protein phosphatase 1 regulatory subunit 14D	PPP1R14D			
Q6DJT9	Zinc finger protein PLAG1	PLAG1			
Q9Y263	Phospholipase A-2-activating protein	PLAA			
Q13018	Secretory phospholipase A2 receptor	PLA2R1			
P98161	Polycystin-1	PKD1			
E9PKD4	Nuclear pore complex-interacting protein family member A5	NPIPA5			
E9PJI5	Nuclear pore complex-interacting protein family member A7	NPIPA7			
P0DM63	Nuclear pore complex-interacting protein family member A8	NPIPA8			
Q8TB73	Protein NDNF	NDNF			
Q8WTR8	Netrin-5	NTN5			
Q6ZWH5	Serine/threonine-protein kinase Nek10	NEK10			
Q12968	Nuclear factor of activated T-cells, cytoplasmic 3	NFATC3			
P80303	Nucleobindin-2	NUCB2			
Q96IY1	Kinetochore-associated protein NSL1 homolog	NSL1			
Q9Y693	LHFPL tetraspan subfamily member 6 protein	LHFPL6			

O60309	Leucine-rich repeat-containing protein 37A3	LRRC37A3			
Q6UY18	Leucine-rich repeat and immunoglobulin-like domain-containing nogo receptor-interacting protein 4	LINGO4			
Q5T752	Late cornified envelope protein	LCE1D			
P36776	Lon protease homolog, mitochondrial	LONP1			
Q6UY01	Leucine-rich repeat-containing protein 31	LRRC31			
P16050	Polyunsaturated fatty acid lipoxygenase ALOX15	ALOX15			
A6NCL2	Leucine-rich colipase-like protein 1	LRCOL1			
Q9BTN0	Leucine-rich repeat and fibronectin type-III domain-containing protein 3	LRFN3			
O43300	Leucine-rich repeat transmembrane neuronal protein 2	LRRTM2			
Q92633	Lysophosphatidic acid receptor 1	LPAR1			
Q9Y468	Lethal	L3MBTL1			
Q5SQ64	Lymphocyte antigen 6 complex locus protein G6f	LY6G6F			
Q9NUN5	Lysosomal cobalamin transport escort protein LMBD1	LMBRD1			
Q538Z0	Leucine zipper protein 6	LUZP6			
A0A0B4J1U3	Immunoglobulin lambda variable 1-36	IGLV1-36			
Q5T7N2	LINE-1 type transposase domain-containing protein 1	L1TD1			
Q8N8F7	Leucine-rich single-pass membrane protein 1	LSMEM1			
P21941	Cartilage matrix protein	MATN1			
Q9H1U4	Multiple epidermal growth factor-like domains protein 9	MEGF9			
Q9BQ69	ADP-ribose glycohydrolase MACROD1	MACROD1			
Q9NX70	Mediator of RNA polymerase II transcription subunit 29	MED29			
Q6P2C8	Mediator of RNA polymerase II transcription subunit 27	MED27			
Q6ZNQ3	Leucine-rich repeat-containing protein 69	LRRC69			
A0A1B0GVS7	MyoD family inhibitor domain-containing protein 2	MDFIC2			
Q9P1T7	MyoD family inhibitor domain-containing protein	MDFIC			
A6NHS7	MANSC domain-containing protein 4	MANSC4			
Q9BSK0	MARVEL domain-containing protein 1	MARVELD1			
O60675	Transcription factor MafK	MAFK			
Q6UWQ5	Lysozyme-like protein 1	LYZL1			
P43365	Melanoma-associated antigen 12	MAGEA12			
Q02978	Mitochondrial 2-oxoglutarate/malate carrier protein	SLC25A11			
Q96LZ2	Melanoma-associated antigen B10	MAGEB10			
A2A368	Melanoma-associated antigen B16	MAGEB16			
P31153	S-adenosylmethionine synthase isoform type-2	MAT2A			
P61626	Lysozyme C	LYZ			
P55083	Microfibril-associated glycoprotein 4	MFAP4			
Q9H8L6	Multimerin-2	MMRN2			

P09237	Matrilysin	MMP7			
Q9H3U5	Major facilitator superfamily domain-containing protein 1	MFSD1			
Q6ZW33	MICAL C-terminal-like protein	MICALCL			
Q86XN8	RNA-binding protein MEX3D	MEX3D			
Q13368	MAGUK p55 subfamily member 3	MPP3			
Q9HD47	Ran guanine nucleotide release factor	RANGRF			
Q9UBU8	Mortality factor 4-like protein 1	MORF4L1			
Q68DK7	Male-specific lethal 1 homolog	MSL1			
Q8NFP9	Neurobeachin	NBEA			
Q15784	Neurogenic differentiation factor	NEUROD2			
Q86UW6	NEDD4-binding protein 2	N4BP2			
Q6P4D5	PABIR family member 1	PABIR3			
Q8N699	Myc target protein 1	MYCT1			
Q8IUG5	Unconventional myosin-XVIIIb	MYO18B			
Q53F19	Nuclear cap-binding protein subunit 3	NCBP3			
O60237	Protein phosphatase 1 regulatory subunit 12B	PPP1R12B			
P25189	Myelin protein P0	MPZ			
Q5VWK0	Neuroblastoma breakpoint family member 6	NBPF6			
B2RTY4	Unconventional myosin-IXa	MYO9A			
P13533	Myosin-6	MYH6			
P05976	Myosin light chain 1/3, skeletal muscle isoform	MYL1			
O75376	Nuclear receptor corepressor 1	NCOR1			
Q9Y221	60S ribosome subunit biogenesis protein NIP7 homolog	NIP7			
P23511	Nuclear transcription factor Y subunit alpha	NFYA			
Q12879	Glutamate receptor ionotropic, NMDA 2A	GRIN2A			
P30419	Glycylpeptide N-tetradecanoyltransferase 1	NMT1			
Q8NBZ9	Putative uncharacterized protein NEXN-AS1	NEXN-AS1			
Q969G9	Protein naked cuticle homolog 1	NKD1			
O75438	NADH dehydrogenase	NDUFB1			
Q9ULW6	Nucleosome assembly protein 1-like 2	NAP1L2			
Q5JPE7	Nodal modulator 2	NOMO2			
P14543	Nidogen-1	NID1			
Q9UMS0	NFU1 iron-sulfur cluster scaffold homolog, mitochondrial	NFU1			
Q99466	Neurogenic locus notch homolog protein 4	NOTCH4			
Q96PB7	Noelin-3	OLFM3			
Q02297	Pro-neuregulin-1, membrane-bound isoform	NRG1			
Q9NPD7	Neuritin	NRN1			
Q8NGX8	Olfactory receptor 6Y1	OR6Y1			
P35658	Nuclear pore complex protein Nup214	NUP214			
P11177	Pyruvate dehydrogenase E1 component subunit beta, mitochondrial	PDHB			
P47893	Olfactory receptor 3A2	OR3A2			
Q8TAK6	Oligodendrocyte transcription factor 1	OLIG1			
Q9BZK8	Ovarian cancer-related protein 1	OCR1			
Q8TAX0	Protein odd-skipped-related 1	OSR1			
Q8NH89	Putative olfactory receptor 5AK3	OR5AK3P			
A6NM76	Olfactory receptor 6C76	OR6C76			
Q8NH02	Olfactory receptor 2T29	OR2T29			
Q9H346	Olfactory receptor 52D1	OR52D1			
Q6IF36	Putative olfactory receptor 8G2	OR8G2P			
P0C646	Olfactory receptor 52Z1	OR52Z1			
Q9NWU1	3-oxoacyl-	OXSM			

Q8IZE3	Protein-associating with the carboxyl-terminal domain of ezrin	SCYL3			
A6NDB9	Paralemmin-3	PALM3			
Q6VY07	Phosphofurin acidic cluster sorting protein 1	PACS1			
O60245	Protocadherin-7	PCDH7			
Q86XP0	Cytosolic phospholipase A2 delta	PLA2G4D			
Q8NGK3	Olfactory receptor 52K2	OR52K2			
Q9Y5F6	Protocadherin gamma-C5	PCDHGC5			
Q9H3R0	Lysine-specific demethylase 4C	KDM4C			
P29375	Lysine-specific demethylase 5A	KDM5A			
Q3LHN0	Keratin-associated protein 25-1	KRTAP25-1			
P22694	cAMP-dependent protein kinase catalytic subunit beta	PRKACB			
Q8NG31	Kinetochore scaffold 1	KNL1			
A0A0C4DH25	Immunoglobulin kappa variable 3D-20	IGKV3D-20			
Q16363	Laminin subunit alpha-4	LAMA4			
O14633	Late cornified envelope protein 2B	LCE2B			
Q5T751	Late cornified envelope protein 1C	LCE1C			
O75335	Liprin-alpha-4	PPFIA4			
P07864	L-lactate dehydrogenase C chain	LDHC			
Q9BYZ2	L-lactate dehydrogenase A-like 6B	LDHAL6B			
P0CW19	LIM and senescent cell antigen-like-containing domain protein 3	LIMS3			
A6NIK2	Leucine-rich repeat-containing protein 10B	LRRC10B			
P51884	Lumican	LUM			
Q17RY6	Lymphocyte antigen 6K	LY6K			
Q9BU23	Lipase maturation factor 2	LMF2			
Q6UX82	Ly6/PLAUR domain-containing protein 8	LYPD8			
Q6UX53	Methyltransferase-like protein 7B	METTL7B			
Q9H000	Probable E3 ubiquitin-protein ligase makorin-2	MKRN2			
P31152	Mitogen-activated protein kinase 4	MAPK4			
Q8IYU8	Calcium uptake protein 2, mitochondrial	MICU2			
Q8N594	MPN domain-containing protein	MPND			
P55001	Microfibrillar-associated protein 2	MFAP2			
Q96JB8	MAGUK p55 subfamily member 4	MPP4			
Q96G30	Melanocortin-2 receptor accessory protein 2	MRAP2			
Q6IN84	rRNA methyltransferase 1, mitochondrial	MRM1			
P30307	M-phase inducer phosphatase 3	CDC25C			
O43439	Protein CBFA2T2	CBFA2T2			
Q9UBK8	Methionine synthase reductase	MTRR			
Q96BF6	Nucleus accumbens-associated protein 2	NACC2			
Q9UJ70	N-acetyl-D-glucosamine kinase	NAGK			
P35749	Myosin-11	MYH11			
P12883	Myosin-7	MYH7			
P13591	Neural cell adhesion molecule 1	NCAM1			
O15394	Neural cell adhesion molecule 2	NCAM2			
Q96P20	NACHT, LRR and PYD domains-containing protein 3	NLRP3			
A6NCS4	Homeobox protein Nkx-2.6	NKX2-6			
Q16617	Protein NKG7	NKG7			
Q8N5V2	Ephexin-1	NGEF			
O60391	Glutamate receptor ionotropic, NMDA 3B	GRIN3B			
Q8NFZ4	Neuroligin-2	NLGN2			
Q96PH1	NADPH oxidase 5	NOX5			
Q86X76	Deaminated glutathione amidase	NIT1			
O15522	Homeobox protein Nkx-2.8	NKX2-8			
Q13287	N-myc-interactor	NMI			

Q14916	Sodium-dependent phosphate transport protein 1	SLC17A1			
Q86UT5	Na	PDZD3			
Q5TEU4	Arginine-hydroxylase NDUFAF5, mitochondrial	NDUFAF5			
Q14978	Nucleolar and coiled-body phosphoprotein 1	NOLC1			
Q9UGY1	Nucleolar protein 12	NOL12			
Q86XR2	Protein Niban 3	NIBAN3			
Q5C9Z4	Nucleolar MIF4G domain-containing protein 1	NOM1			
Q9BSH3	Nicolin-1	NICN1			
Q13133	Oxysterols receptor LXR-alpha	NR1H3			
Q8NET5	NFAT activation molecule 1	NFAM1			
Q7Z3B1	Neuronal growth regulator 1	NEGR1			
C9JG80	Nuclear pore complex-interacting protein family member B4	NPIPB4			
O43678	NADH dehydrogenase	NDUFA2			
Q86SG6	Serine/threonine-protein kinase Nek8	NEK8			
Q8TDX7	Serine/threonine-protein kinase Nek7	NEK7			
Q5PT55	Sodium/bile acid cotransporter 5	SLC10A5			
Q9H0G5	Nuclear speckle splicing regulatory protein 1	NSRP1			
Q8NGQ2	Olfactory receptor 6Q1	OR6Q1			
O00330	Pyruvate dehydrogenase protein X component, mitochondrial	PDHX			
Q8NGL1	Olfactory receptor 5D18	OR5D18			
Q8NGL7	Olfactory receptor 4P4	OR4P4			
Q9H1P3	Oxysterol-binding protein-related protein 2	OSBPL2			
Q8N7R1	POM121-like protein 12	POM121L1			
Q58A45	PAN2-PAN3 deadenylation complex subunit PAN3	PAN3			
Q9Y5G4	Protocadherin gamma-A9	PCDHGA9			
I0J062	Proapoptotic nucleolar protein 1	PANO1			
Q9BZA8	Protocadherin-11 Y-linked	PCDH11Y			
Q9H361	Polyadenylate-binding protein 3	PABPC3			
Q9BYU1	Pre-B-cell leukemia transcription factor 4	PBX4			
Q9Y5I2	Protocadherin alpha-10	PCDHA10			
P14555	Phospholipase A2, membrane associated	PLA2G2A			
P52945	Pancreas/duodenum homeobox protein 1	PDX1			
Q9Y5I0	Protocadherin alpha-13	PCDHA13			
Q9Y5F3	Protocadherin beta-1	PCDHB1			
P51582	P2Y purinoceptor 4	P2RY4			
Q96IZ0	PRKC apoptosis WT1 regulator protein	PAWR			
A6NKB5	Pecanex-like protein 2	PCNX2			
Q86VZ1	P2Y purinoceptor 8	P2RY8			
O75747	Phosphatidylinositol 4-phosphate 3-kinase C2 domain-containing subunit gamma	PIK3C2G			
Q9Y5K3	Choline-phosphate cytidylyltransferase B	PCYT1B			
Q9Y6V0	Protein piccolo	PCLO			
Q8IVL6	Prolyl 3-hydroxylase 3	P3H3			
Q15118		PDK1			
Q9HCJ3	Ribonucleoprotein PTB-binding 2	RAVER2			
Q9NZQ7	Programmed cell death 1 ligand 1	CD274			
Q9Y616	Interleukin-1 receptor-associated kinase 3	IRAK3			
Q8WXH2	Junctophilin-3	JPH3			
P49895	Type I iodothyronine deiodinase	DIO1			
Q9NZM3	Intersectin-2	ITSN2			
P43405	Tyrosine-protein kinase SYK	SYK			

P48051	G protein-activated inward rectifier potassium channel 2	KCNJ6			
P63252	Inward rectifier potassium channel 2	KCNJ2			
Q8TBB5	Kelch domain-containing protein	KLHDC4			
Q15842	ATP-sensitive inward rectifier potassium channel 8	KCNJ8			
P43632	Killer cell immunoglobulin-like receptor 2DS4	KIR2DS4			
Q8N371	Bifunctional peptidase and arginyl-hydroxylase JMJD5	KDM8			
P06310	Immunoglobulin kappa variable 2-30	IGKV2-30			
Q15139	Serine/threonine-protein kinase D1	PRKD1			
O95232	Luc7-like protein 3	LUC7L3			
Q08380	Galectin-3-binding protein	LGALS3BP			
O75387	Large neutral amino acids transporter small subunit 3	SLC43A1			
Q9Y5L5	Lens epithelial cell protein LEP503	LENEP			
Q9P2M1	LRP2-binding protein	LRP2BP			
O15165	Low-density lipoprotein receptor class A domain-containing protein 4	LDLRAD4			
Q9UFC0	Leucine-rich repeat and WD repeat-containing protein 1	LRWD1			
Q9BVC4	Target of rapamycin complex subunit LST8	MLST8			
Q8N1E2	Lysozyme g-like protein 1	LYG1			
Q9BRA0	N-alpha-acetyltransferase 38, NatC auxiliary subunit	NAA38			
P23368	NAD-dependent malic enzyme, mitochondrial	ME2			
P49641	Alpha-mannosidase 2x	MAN2A2			
Q9NWA0	Mediator of RNA polymerase II transcription subunit 9	MED9			
Q5JSS6	Meiosis expressed gene 1 protein homolog	MEIG1			
P29966	Myristoylated alanine-rich C-kinase substrate	MARCKS			
Q9NPJ6	Mediator of RNA polymerase II transcription subunit 4	MED4			
Q9BRK4	Leucine zipper putative tumor suppressor 2	LZTS2			
Q9Y586	Protein mab-21-like 2	MAB21L2			
Q9NQ48	Leucine zipper transcription factor-like protein 1	LZTFL1			
Q92585	Mastermind-like protein 1	MAML1			
P33032	Melanocortin receptor 5	MC5R			
O95983	Methyl-CpG-binding domain protein 3	MBD3			
Q8NHW3	Transcription factor MafA	MAFA			
Q9UI17	Dimethylglycine dehydrogenase, mitochondrial	DMGDH			
Q7Z4W2	Lysozyme-like protein 2	LYZL2			
P43366	Melanoma-associated antigen B1	MAGEB1			
O15480	Melanoma-associated antigen B3	MAGEB3			
P45452	Collagenase 3	MMP13			
Q9NUT2	Mitochondrial potassium channel ATP-binding subunit	ABCB8			
O95568	Histidine protein methyltransferase 1 homolog	METTL18			
Q9BXP8	Pappalysin-2	PAPPA2			
Q5JR59	Microtubule-associated tumor suppressor candidate 2	MTUS2			
Q8TAX7	Mucin-7	MUC7			
Q96L15	Ecto-ADP-ribosyltransferase 5	ART5			
Q969H8	Myeloid-derived growth factor	MYDGF			

Q86XG9	Putative neuroblastoma breakpoint family member 5	NBPF5P			
Q9NZM1	Myoferlin	MYOF			
Q01658	Protein Dr1	DR1			
Q69YL0	Protein NCBP2AS2	NCBP2AS2			
Q9Y2A7	Nck-associated protein 1	NCKAP1			
Q6J4K2	Mitochondrial sodium/calcium exchanger protein	SLC8B1			
Q4KMZ8	Sodium/potassium-transporting ATPase subunit beta-1-interacting protein 1	NKAIN1			
Q9HAS0	Protein Njmu-R1	C17orf75			
O43908	NKG2-F type II integral membrane protein	KLRC4			
P80188	Neutrophil gelatinase-associated lipocalin	LCN2			
Q9C056	Homeobox protein Nkx-6.2	NKX6-2			
Q7Z6G3	N-terminal EF-hand calcium-binding protein 2	NECAB2			
Q9NQS3	Nectin-3	NECTIN3			
Q9Y2I1	Nischarin	NISCH			
Q9HBL8	NmrA-like family domain-containing protein 1	NMRAL1			
Q9Y639	Neuroplastin	NPTN			
O95436	Sodium-dependent phosphate transport protein 2B	SLC34A2			
Q8WU39	Marginal zone B- and B1-cell-specific protein	MZB1			
A8MTQ0	Homeobox protein notochord	NOTO			
Q9Y5X5	Neuropeptide FF receptor 2	NPFFR2			
Q8NCF5	NFATC2-interacting protein	NFATC2IP			
Q0ZGT2	Nexilin	NEXN			
Q8NC67	Neuropilin and tolloid-like protein 2	NETO2			
Q9Y4A8	Nuclear factor erythroid 2-related factor 3	NFE2L3			
O43869	Olfactory receptor 2T1	OR2T1			
Q8NH85	Olfactory receptor 8U3	OR8U3			
Q8NGL9	Olfactory receptor 4C16	OR4C16			
Q96R45	Olfactory receptor 2A7	OR2A7			
Q9UMX2	Ornithine decarboxylase antizyme 3	OAZ3			
Q96R30	Olfactory receptor 2V2	OR2V2			
Q8NGQ6	Olfactory receptor 9I1	OR9I1			
Q9ULD0	2-oxoglutarate dehydrogenase-like, mitochondrial	OGDHL			
Q9NPJ8	NTF2-related export protein 2	NXT2			
Q8NGE2	Olfactory receptor 2AP1	OR2AP1			
Q13516	Oligodendrocyte transcription factor 2	OLIG2			
A6NM03	Olfactory receptor 2AG2	OR2AG2			
Q9H344	Olfactory receptor 51I2	OR51I2			
Q8NGI8	Olfactory receptor 5AN1	OR5AN1			
A5D8V7	Outer dynein arm-docking complex subunit 3	ODAD3			
Q8NHC7	Olfactory receptor 14C36	OR14C36			
Q6GTS8	N-fatty-acyl-amino acid synthase/hydrolase PM20D1	PM20D1			
Q9BRF8	Serine/threonine-protein phosphatase CPPED1	CPPED1			
Q9Y6N1	Cytochrome c oxidase assembly protein COX11, mitochondrial	COX11			
Q08477	Cytochrome P450 4F3	CYP4F3			
Q9Y600	Cysteine sulfinic acid decarboxylase	CSAD			
P0C5K6	Putative tumor antigen NA88-A	VENTXP1			
Q96M42	Putative uncharacterized protein encoded by LINC00479	LINC00479			
Q9Y6H8	Gap junction alpha-3 protein	GJA3			

Q8NG35	Beta-defensin 105	DEFB105A			
A8MPP1	Putative ATP-dependent RNA helicase DDX11-like protein 8	DDX11L8			
Q7RTX7	Cation channel sperm-associated protein 4	CATSPER4			
Q9H175	Cysteine/serine-rich nuclear protein 2	CSRNP2			
P17538	Chymotrypsinogen B	CTRB1			
P39880	Homeobox protein cut-like 1	CUX1			
Q9H0L4	Cleavage stimulation factor subunit 2 tau variant	CSTF2T			
Q15828	Cystatin-M	CST6			
Q96C86	m7GpppX diphosphatase	DCPS			
P59666	Neutrophil defensin 3	DEFA3			
O43602	Neuronal migration protein doublecortin	DCX			
O95865	N	DDAH2			
Q9Y394	Dehydrogenase/reductase SDR family member 7	DHRS7			
O60832	H/ACA ribonucleoprotein complex subunit DKC1	DKC1			
P56937	3-keto-steroid reductase/17-beta-hydroxysteroid dehydrogenase 7	HSD17B7			
Q8N608	Inactive dipeptidyl peptidase 10	DPP10			
P28562	Dual specificity protein phosphatase 1	DUSP1			
P0CJ85	Double homeobox protein 4-like protein 2	DUX4L2			
Q8IZU8	Dermatan-sulfate epimerase-like protein	DSEL			
Q8TC92	Ecto-NOX disulfide-thiol exchanger 1	ENOX1			
Q9H0I2	Enkurin domain-containing protein 1	ENKD1			
Q6P531	Glutathione hydrolase 6	GGT6			
Q53GS7	Nucleoporin GLE1	GLE1			
P36268	Inactive glutathione hydrolase 2	GGT2			
Q8TDS4	Hydroxycarboxylic acid receptor 2	HCAR2			
Q4AE62	Glycosyltransferase-like domain-containing protein 1	GTDC1			
P22732	Solute carrier family 2, facilitated glucose transporter member 5	SLC2A5			
Q93079	Histone H2B type 1-H	H2BC9			
O60565	Gremlin-1	GREM1			
P33778	Histone H2B type 1-B	H2BC3			
Q9Y3R0	Glutamate receptor-interacting protein 1	GRIP1			
P39086	Glutamate receptor ionotropic, kainate 1	GRIK1			
P41594	Metabotropic glutamate receptor 5	GRM5			
P31275	Homeobox protein Hox-C12	HOXC12			
Q00444	Homeobox protein Hox-C5	HOXC5			
P07900	Heat shock protein HSP 90-alpha	HSP90AA1			
P0CJ77	Humanin-like 10	MTRNR2L10			
S4R3P1	Humanin-like 13	MTRNR2L13			
P0CJ72	Humanin-like 5	MTRNR2L5			
P09914	Interferon-induced protein with tetratricopeptide repeats 1	IFIT1			
Q8IU54	Interferon lambda-1	IFNL1			
P01569	Interferon alpha-5	IFNA5			
P30740	Leukocyte elastase inhibitor	SERPINB1			
O00425	Insulin-like growth factor 2 mRNA-binding protein 3	IGF2BP3			
Q8NAC3	Interleukin-17 receptor C	IL17RC			
Q9NZH8	Interleukin-36 gamma	IL36G			

P40933	Interleukin-15	IL15			
Q9H2X8	Interferon alpha-inducible protein 27-like protein 2	IFI27L2			
P41134	DNA-binding protein inhibitor ID-1	ID1			
P48668	Keratin, type II cytoskeletal 6C	KRT6C			
Q6UWN8	Serine protease inhibitor Kazal-type 6	SPINK6			
P54819	Adenylate kinase 2, mitochondrial	AK2			
P19012	Keratin, type I cytoskeletal 15	KRT15			
Q8NA54	IQ and ubiquitin-like domain-containing protein	IQUB			
Q9UI26	Importin-11	IPO11			
Q5VZ46	Uncharacterized protein KIAA1614	KIAA1614			
Q9Y581	Insulin-like peptide INSL6	INSL6			
Q96LW2	Ribosomal protein S6 kinase-related protein	RSKR			
Q9P0G3	Kallikrein-14	KLK14			
O15550	Lysine-specific demethylase 6A	KDM6A			
O75676	Ribosomal protein S6 kinase alpha-4	RPS6KA4			
Q9Y5K2	Kallikrein-4	KLK4			
Q9BYR5	Keratin-associated protein 4-2	KRTAP4-2			
Q6L8G8	Keratin-associated protein 5-7	KRTAP5-7			
Q9NPA1	Calcium-activated potassium channel subunit beta-3	KCNMB3			
P06312	Immunoglobulin kappa variable 4-1	IGKV4-1			
A0A0C4DH68	Immunoglobulin kappa variable 2-24	IGKV2-24			
A8MYP8	Outer dense fiber protein 3B	ODF3B			
P0C7T2	Olfactory receptor 2T7	OR2T7			
O95918	Olfactory receptor 2H2	OR2H2			
Q8N162	Olfactory receptor 8H2	OR8H2			
Q8NH76	Olfactory receptor 56B4	OR56B4			
Q8NH67	Olfactory receptor 52I2	OR52I2			
Q8NGX5	Olfactory receptor 10K1	OR10K1			
Q8NGK4	Olfactory receptor 52K1	OR52K1			
O15294	UDP-N-acetylglucosamine--peptide N-acetylglucosaminyltransferase 110 kDa subunit	OGT			
Q8NGC4	Olfactory receptor 10G3	OR10G3			
Q8NG94	Olfactory receptor 11H1	OR11H1			
Q9GZU5	Nyctalopin	NYX			
Q8NGS9	Olfactory receptor 13C2	OR13C2			
Q96HA1	Nuclear envelope pore membrane protein POM 121	POM121			
Q14681	BTB/POZ domain-containing protein KCTD2	KCTD2			
Q9H400	Lck-interacting transmembrane adapter 1	LIME1			
P0C6S8	Leucine-rich repeat and immunoglobulin-like domain-containing nogo receptor-interacting protein 3	LINGO3			
Q9Y546	Leucine-rich repeat-containing protein 42	LRRC42			
Q07954	Prolow-density lipoprotein receptor-related protein 1	LRP1			
Q9C0I9	Leucine-rich repeat-containing protein 27	LRRC27			
Q9BYS8	Leucine-rich repeat-containing protein 2	LRRC2			
Q14767	Latent-transforming growth factor beta-binding protein 2	LTBP2			
Q96LR2	Leucine rich adaptor protein 1	LURAP1			
P78559	Microtubule-associated protein 1A	MAP1A			

O60244	Mediator of RNA polymerase II transcription subunit 14	MED14			
Q8NI22	Multiple coagulation factor deficiency protein 2	MCFD2			
D6RGH6	Multicilin	MCIDAS			
P33991	DNA replication licensing factor MCM4	MCM4			
P27448	MAP/microtubule affinity-regulating kinase 3	MARK3			
Q8TCQ1	E3 ubiquitin-protein ligase MARCHF1	MARCHF1			
O95819	Mitogen-activated protein kinase kinase kinase kinase 4	MAP4K4			
Q9NS15	Latent-transforming growth factor beta-binding protein 3	LTBP3			
P0DP75	Putative uncharacterized protein MED14OS	MED14OS			
Q05BQ5	MBT domain-containing protein 1	MBTD1			
Q9Y2U8	Inner nuclear membrane protein Man1	LEMD3			
Q86U38	Nucleolar protein 9	NOP9			
Q8TDR0	TRAF3-interacting protein 1	TRAF3IP1			
Q99797	Mitochondrial intermediate peptidase	MIPEP			
Q8NFP7	Diphosphoinositol polyphosphate phosphohydrolase 3-alpha	NUDT10			
P58340	Myeloid leukemia factor 1	MLF1			
Q6P1R3	Myb/SANT-like DNA-binding domain-containing protein 2	MSANTD2			
Q6ZTZ1	Myb/SANT-like DNA-binding domain-containing protein 1	MSANTD1			
Q8NHV5	Modulator of smoothened protein	MOSMO			
Q6UVY6	DBH-like monooxygenase protein 1	MOXD1			
Q9C0I1	Myotubularin-related protein 12	MTMR12			
Q5VZV1	Protein-lysine methyltransferase METTL21C	METTL21C			
P60321	Nanos homolog 2	NANOS2			
Q9H9S0	Homeobox protein NANOG	NANOG			
Q8N9F0	N-acetylaspartate synthetase	NAT8L			
O00746	Nucleoside diphosphate kinase, mitochondrial	NME4			
P60201	Myelin proteolipid protein	PLP1			
Q86VE0	Myb-related transcription factor, partner of profilin	MYPOP			
P12829	Myosin light chain 4	MYL4			
Q8NFW9	Rab effector MyRIP	MYRIP			
Q13459	Unconventional myosin-IXb	MYO9B			
Q969V3	Nicalin	NCLN			
P25103	Substance-P receptor	TACR1			
O76041	Nebulette	NEBL			
Q9HBY0	NADPH oxidase 3	NOX3			
Q15843	NEDD8	NEDD8			
Q16517	Neuronatin	NNAT			
P47972	Neuronal pentraxin-2	NPTX2			
Q9HB89	Neuromedin-U receptor 1	NMUR1			
O43181	NADH dehydrogenase	NDUFS4			
O00567	Nucleolar protein 56	NOP56			
Q9BT67	NEDD4 family-interacting protein 1	NDFIP1			
Q8IZ57	Neurensin-1	NRSN1			
P78549	Endonuclease III-like protein 1	NTHL1			
Q3KNT7	Putative NOL1/NOP2/Sun domain family member 5B	NSUN5P1			
Q6IF42	Olfactory receptor 2A2	OR2A2			
Q8NHA8	Olfactory receptor 1F12	OR1F12			
A6NFC9	Putative olfactory receptor 2W5 pseudogene	OR2W5P			
Q8N0Y3	Olfactory receptor 4N4	OR4N4			

Q9BW72	HIG1 domain family member 2A, mitochondrial	HIGD2A			
P02790	Hemopexin	HPX			
Q14526	Hypermethylated in cancer 1 protein	HIC1			
P20702	Integrin alpha-X	ITGAX			
P11215	Integrin alpha-M	ITGAM			
P17535	Transcription factor jun-D	JUND			
Q8NI35	InaD-like protein	PATJ			
P0DMQ5	Putative transmembrane protein INAFM2	INAFM2			
Q9P2N7	Kelch-like protein 13	KLHL13			
Q9NXS3	Kelch-like protein 28	KLHL28			
Q15726	Metastasis-suppressor KiSS-1	KISS1			
Q8N109	Killer cell immunoglobulin-like receptor 2DL5A	KIR2DL5A			
P60370	Keratin-associated protein 10-5	KRTAP10-5			
Q3LI73	Keratin-associated protein 19-4	KRTAP19-4			
Q3LI61	Keratin-associated protein 20-2	KRTAP20-2			
P16150	Leukosialin	SPN			
Q6MZP7	Protein lin-54 homolog	LIN54			
A9UHW6	MIF4G domain-containing protein	MIF4GD			
Q9NPA2	Matrix metalloproteinase-25	MMP25			
O15264	Mitogen-activated protein kinase 13	MAPK13			
Q16539	Mitogen-activated protein kinase 14	MAPK14			
Q96AX9	E3 ubiquitin-protein ligase MIB2	MIB2			
Q9BPX6	Calcium uptake protein 1, mitochondrial	MICU1			
Q99549	M-phase phosphoprotein 8	MPHOSPH			
Q8N3R9	MAGUK p55 subfamily member 5	MPP5			
O60669	Monocarboxylate transporter 2	SLC16A7			
Q6DT37	Serine/threonine-protein kinase MRCK gamma	CDC42BPG			
Q9H8S9	MOB kinase activator 1A	MOB1A			
Q685J3	Mucin-17	MUC17			
Q96NY9	Crossover junction endonuclease MUS81	MUS81			
P80294	Metallothionein-1H	MT1H			
Q6W4X9	Mucin-6	MUC6			
P56278	Protein p13 MTCP-1	MTCP1			
Q8N339	Metallothionein-1M	MT1M			
Q86W26	NACHT, LRR and PYD domains-containing protein 10	NLRP10			
P0DI83	Ras-related protein Rab-34, isoform NARR	RAB34			
P13349	Myogenic factor 5	MYF5			
Q8WXR4	Myosin-IIIb	MYO3B			
Q32MK0	Myosin light chain kinase 3	MYLK3			
Q86WI3	Protein NLRC5	NLRC5			
Q13952	Nuclear transcription factor Y subunit gamma	NFYC			
Q86WB0	Nuclear-interacting partner of	ZC3HC1			
O60721	Sodium/potassium/calcium exchanger 1	SLC24A1			
Q6P499	NIPA-like protein 3	NIPAL3			
Q99733	Nucleosome assembly protein 1-like 4	NAP1L4			
Q15761	Neuropeptide Y receptor type 5	NPY5R			
P50391	Neuropeptide Y receptor type 4	NPY4R			
Q14995	Nuclear receptor subfamily 1 group D member 2	NR1D2			
P46531	Neurogenic locus notch homolog protein 1	NOTCH1			
Q8IW45	ATP-dependent	NAXD			
Q15155	Nodal modulator 1	NOMO1			
Q9UHC9	NPC1-like intracellular cholesterol transporter 1	NPC1L1			

P43354	Nuclear receptor subfamily 4 group A member 2	NR4A2			
O75251	NADH dehydrogenase	NDUFS7			
Q96S42	Nodal homolog	NODAL			
P22736	Nuclear receptor subfamily 4 group A member 1	NR4A1			
A6NJ64	Putative nuclear pore complex-interacting protein family member B2	NPIPB2			
O94856	Neurofascin	NFASC			
Q96DE0	U8 snoRNA-decapping enzyme	NUDT16			
Q9BRQ3	Uridine diphosphate glucose pyrophosphatase NUDT22	NUDT22			
P20783	Neurotrophin-3	NTF3			
Q496H8	Neuritin-like protein	NRN1L			
P61970	Nuclear transport factor 2	NUTF2			
Q9NRX3	NADH dehydrogenase	NDUFA4L2			
Q96CW9	Netrin-G2	NTNG2			
Q8TEA1	tRNA	NSUN6			
Q8NGG1	Olfactory receptor 8J2	OR8J2			
P48741	Putative heat shock 70 kDa protein 7	HSPA7			
A0A0C4DH33	Immunoglobulin heavy variable 1-24	IGHV1-24			
A0A0C4DH36	Probable non-functional immunoglobulin heavy variable 3-38	IGHV3-38			
P14866	Heterogeneous nuclear ribonucleoprotein L	HNRNPL			
A0A1B0GWH4	Heat shock transcription factor, X-linked member 3	HSFX3			
Q9Y661	Heparan sulfate glucosamine 3-O-sulfotransferase 4	HS3ST4			
Q8WW32	High mobility group protein B4	HMGB4			
Q12766	HMG domain-containing protein	HMGXB3			
Q8TCB0	Interferon-induced protein 44	IFI44			
P01562	Interferon alpha-1/13	IFNA1;			
P01563	Interferon alpha-2	IFNA2			
P13164	Interferon-induced transmembrane protein 1	IFITM1			
Q6IS14	Eukaryotic translation initiation factor 5A-1-like	EIF5AL1			
P05015	Interferon alpha-16	IFNA16			
Q9BW83	Intraflagellar transport protein 27 homolog	IFT27			
P01880	Immunoglobulin heavy constant delta	IGHD			
P0DOX6	Immunoglobulin mu heavy chain				
P08069	Insulin-like growth factor 1 receptor	IGF1R			
Q9BZV3	Interphotoreceptor matrix proteoglycan 2	IMPG2			
Q53TQ3	INO80 complex subunit D	INO80D			
P23229	Integrin alpha-6	ITGA6			
Q9H8X2	Inositol-pentakisphosphate 2-kinase	IPPK			
A8MXQ7	IQ motif and ankyrin repeat domain-containing protein 1	IQANK1			
O75525	KH domain-containing, RNA-binding, signal transduction-associated protein 3	KHDRBS3			
O15054	Lysine-specific demethylase 6B	KDM6B			
O60928	Inward rectifier potassium channel 13	KCNJ13			
Q8N4N8	Kinesin-like protein KIF2B	KIF2B			
Q9H7Z6	Histone acetyltransferase KAT8	KAT8			
P17612	cAMP-dependent protein kinase catalytic subunit alpha	PRKACA			

A0A0U1RRK4	LBH domain-containing protein 2	LBHD2			
P50458	LIM/homeobox protein Lhx2	LHX2			
Q96B96	Lipid droplet assembly factor 1	TMEM159			
Q6UX15	Layilin	LAYN			
Q71RC2	La-related protein 4	LARP4			
Q6ZMR3	L-lactate dehydrogenase A-like 6A	LDHAL6A			
P53667	LIM domain kinase 1	LIMK1			
Q2VYF4	LETM1 domain-containing protein LETM2, mitochondrial	LETM2			
Q9H1C0	Lysophosphatidic acid receptor 5	LPAR5			
Q9HAT1	Protein ERGIC-53-like	LMAN1L			
Q9BS40	Latexin	LXN			
H3BQJ8	Lymphocyte antigen 6L	LY6L			
O15296	Polyunsaturated fatty acid lipoxygenase ALOX15B	ALOX15B			
Q86SG7	Lysozyme g-like protein 2	LYG2			
P33908	Mannosyl-oligosaccharide 1,2-alpha-mannosidase IA	MAN1A1			
Q5U5X0	Complex III assembly factor LYRM7	LYRM7			
Q9HD34	LYR motif-containing protein 4	LYRM4			
Q8N112	Leucine-rich single-pass membrane protein 2	LSMEM2			
Q9NVC6	Mediator of RNA polymerase II transcription subunit 17	MED17			
A2RUB1	Meiosis-specific coiled-coil domain-containing protein MEIOC	MEIOC			
Q96PG2	Membrane-spanning 4-domains subfamily A member 10	MS4A10			
Q8IZ02	Leucine-rich repeat-containing protein 34	LRRC34			
Q9UEW3	Macrophage receptor MARCO	MARCO			
Q12851	Mitogen-activated protein kinase kinase kinase kinase 2	MAP4K2			
Q5T0T0	E3 ubiquitin-protein ligase MARCHF8	MARCHF8			
Q9Y3C7	Mediator of RNA polymerase II transcription subunit 31	MED31			
Q5SRI9	Glycoprotein endo-alpha-1,2-mannosidase	MANEA			
Q9HCI5	Melanoma-associated antigen E1	MAGEE1			
P20916	Myelin-associated glycoprotein	MAG			
Q9H213	Melanoma-associated antigen H1	MAGEH1			
A2RUH7	Myosin-binding protein H-like	MYBPHL			
Q9BXT6	RNA helicase Mov10l1	MOV10L1			
P0DM35	Metallothionein 1H-like protein 1	MT1HL1			
Q9P267	Methyl-CpG-binding domain protein 5	MBD5			
P43357	Melanoma-associated antigen 3	MAGEA3			
Q8WV92	MIT domain-containing protein 1	MITD1			
Q7Z3K6	Mesoderm induction early response protein 3	MIER3			
Q13361	Microfibrillar-associated protein 5	MFAP5			
A1L020	RNA-binding protein MEX3A	MEX3A			
Q9NXB0	Meckel syndrome type 1 protein	MKS1			
P11717	Cation-independent mannose-6-phosphate receptor	IGF2R			
Q6UWV2	Myelin protein zero-like protein 3	MPZL3			
Q5VZR4	Hippocampus abundant transcript-like protein 2	MFSD14C			
Q96BX8	MOB kinase activator 3A	MOB3A			
Q96J65	ATP-binding cassette sub-family C member 12	ABCC12			
P45985	Dual specificity mitogen-activated protein kinase kinase 4	MAP2K4			
Q49AM1	Transcription termination factor 2, mitochondrial	MTERF2			

P04733	Metallothionein-1F	MT1F			
O75431	Metaxin-2	MTX2			
Q86W25	NACHT, LRR and PYD domains-containing protein 13	NLRP13			
Q9NX02	NACHT, LRR and PYD domains-containing protein 2	NLRP2			
Q8IZQ8	Myocardin	MYOCD			
Q9NQX4	Unconventional myosin-Vc	MYO5C			
Q5VTT5	Myomesin-3	MYOM3			
Q5M9Q1	NKAP-like protein	NKAPL			
Q8NHV4	Protein NEDD1	NEDD1			
Q8N5F7	NF-kappa-B-activating protein	NKAP			
Q8NC96	Adaptin ear-binding coat-associated protein 1	NECAP1			
Q15270	NK1 transcription factor-related protein 1	NKX1-1			
A6NJ46	Homeobox protein Nkx-6.3	NKX6-3			
Q99743	Neuronal PAS domain-containing protein 2	NPAS2			
Q8TAU0	Homeobox protein Nkx-2.3	NKX2-3			
Q8N2Q7	Neuroligin-1	NLGN1			
Q9H8H0	Nucleolar protein 11	NOL11			
Q86SE8	Nucleoplasmin-2	NPM2			
P03905	NADH-ubiquinone oxidoreductase chain 4	MT-ND4			
Q7Z2Y5	Nik-related protein kinase	NRK			
Q9BW60	Elongation of very long chain fatty acids protein 1	ELOVL1			
P61570	Endogenous retrovirus group K member 25 Env polyprotein	ERVK-25			
P60002	Transcription elongation factor 1 homolog	ELOF1			
Q9UFP1	Golgi-associated kinase 1A	GASK1A			
Q13467	Frizzled-5	FZD5			
Q9H706	GRB2-associated and regulator of MAPK protein 1	GAREM1			
Q01415	N-acetylgalactosamine kinase	GALK2			
Q96N19	Integral membrane protein GPR137	GPR137			
P47211	Galanin receptor type 1	GALR1			
Q8N4A0	Polypeptide N-acetylgalactosaminyltransferase 4	GALNT4			
P27469	G0/G1 switch protein 2	G0S2			
Q4G0N0	Inactive N-acetyllactosaminide alpha-1,3-galactosyltransferase	GGTA1			
P07902	Galactose-1-phosphate uridylyltransferase	GALT			
H0YM25	Golgin subfamily A member 6-like protein 22	GOLGA6L22			
Q8N6F7	Germinal center-associated signaling and motility protein	GCSAM			
Q06210	Glutamine--fructose-6-phosphate aminotransferase	GFPT1			
P10070	Zinc finger protein GLI2	GLI2			
P32455	Guanylate-binding protein 1	GBP1			
Q6IC98	GRAM domain-containing protein 4	GRAMD4			
Q6ZS86	Putative glycerol kinase 5	GK5			
P14672	Solute carrier family 2, facilitated glucose transporter member 4	SLC2A4			
Q96P88	Putative gonadotropin-releasing hormone II receptor	GNRHR2			
Q8NGU9	Probable G-protein coupled receptor 150	GPR150			
Q3V6T2	Girdin	CCDC88A			
P08631	Tyrosine-protein kinase HCK	HCK			
Q9NYZ3	G2 and S phase-expressed protein 1	GTSE1			
P09488	Glutathione S-transferase Mu 1	GSTM1			

Q7Z2G1	Histone H2B type W-T	H2BW1			
Q9UBK5	Hematopoietic cell signal transducer	HCST			
P02100	Hemoglobin subunit epsilon	HBE1			
O00291	Huntingtin-interacting protein 1	HIP1			
O43248	Homeobox protein Hox-C11	HOXC11			
P09067	Homeobox protein Hox-B5	HOXB5			
Q4G112	Heat shock factor protein 5	HSF5			
Q8N7B1	HORMA domain-containing protein 2	HORMAD2			
Q6PI97	Cilia- and flagella-associated protein HOATZ	HOATZ			
P41235	Hepatocyte nuclear factor 4-alpha	HNF4A			
K9M1U5	Interferon lambda-4	IFNL4			
Q9Y366	Intraflagellar transport protein 52 homolog	IFT52			
Q12905	Interleukin enhancer-binding factor 2	ILF2			
Q0D2I5	Non-homologous end joining factor IFFO1	IFFO1			
Q9NQC8	Intraflagellar transport protein 46 homolog	IFT46			
Q8WYA0	Intraflagellar transport protein 81 homolog	IFT81			
Q08334	Interleukin-10 receptor subunit beta	IL10RB			
P01857	Immunoglobulin heavy constant gamma 1	IGHG1			
Q8NFR9	Interleukin-17 receptor E	IL17RE			
Q86Y46	Keratin, type II cytoskeletal 73	KRT73			
P08648	Integrin alpha-5	ITGA5			
O14498	Immunoglobulin superfamily containing leucine-rich repeat protein	ISLR			
Q00978	Interferon regulatory factor 9	IRF9			
P50053	Ketohexokinase	KHK			
Q9ULS6	Potassium voltage-gated channel subfamily S member 2	KCNS2			
Q8N7A1	Kelch domain-containing protein	KLHDC1			
Q14953	Killer cell immunoglobulin-like receptor 2DS5	KIR2DS5			
O43525	Potassium voltage-gated channel subfamily KQT member 3	KCNQ3			
Q9BVG8	Kinesin-like protein KIFC3	KIFC3			
Q9ULI4	Kinesin-like protein KIF26A	KIF26A			
Q6PID8	Kelch domain-containing protein 10	KLHDC10			
Q8NGV7	Olfactory receptor 5H2	OR5H2			
Q6ZSK4	Putative uncharacterized protein NTM-AS1	NTM-AS1			
Q6GQQ9	OTU domain-containing protein 7B	OTUD7B			
Q96PU9	Outer dense fiber protein 3	ODF3			
Q8NGD1	Olfactory receptor 4N2	OR4N2			
P14920	D-amino-acid oxidase	DAO			
Q8NFH5	Nucleoporin NUP35	NUP35			
Q92621	Nuclear pore complex protein Nup205	NUP205			
P48539	Calmodulin regulator protein	PCP4			
Q99453	Paired mesoderm homeobox protein 2B	PHOX2B			
A4D2B8	Putative postmeiotic segregation increased 2-like protein 1	PMS2P1			
Q03181	Peroxisome proliferator-activated receptor delta	PPARD			
P63136	Endogenous retrovirus group K member 25 Pol protein	ERVK-25			
P10696	Alkaline phosphatase, germ cell type	ALPG			

Q8TBY8	Polyamine-modulated factor 1-binding protein 1	PMFBP1			
Q15166	Serum paraoxonase/lactonase 3	PON3			
Q15149	Plectin	PLEC			
Q9H3Y8	Pancreatic progenitor cell differentiation and proliferation factor	PPDPF			
Q9NZM6	Polycystic kidney disease 2-like 2 protein	PKD2L2			
Q9UQ26	Regulating synaptic membrane exocytosis protein 2	RIMS2			
Q9HB21	Pleckstrin homology domain-containing family A member 1	PLEKHA1			
Q96QC0	Serine/threonine-protein phosphatase 1 regulatory subunit 10	PPP1R10			
Q9H478	KCNQ1 downstream neighbor protein	KCNQ1DN			
Q8N135	Leucine-rich repeat LGI family member 4	LGI4			
Q99538	Legumain	LGMN			
Q9UIC8	Leucine carboxyl methyltransferase 1	LCMT1			
Q6ZQY2	Leucine-rich repeat-containing protein 74B	LRRC74B			
Q8N1G4	Leucine-rich repeat-containing protein 47	LRRC47			
P09960	Leukotriene A-4 hydrolase	LTA4H			
Q6UXM1	Leucine-rich repeats and immunoglobulin-like domains protein 3	LRIG3			
Q8WUT4	Leucine-rich repeat neuronal protein 4	LRRN4			
Q9C0E8	Endoplasmic reticulum junction formation protein lunapark	LNPK			
Q8NHJ6	Leukocyte immunoglobulin-like receptor subfamily B member 4	LILRB4			
Q03252	Lamin-B2	LMNB2			
Q9H9Z2	Protein lin-28 homolog A	LIN28A			
P80748	Immunoglobulin lambda variable 3-21	IGLV3-21			
Q14582	Max dimerization protein 4	MXD4			
P43358	Melanoma-associated antigen 4	MAGEA4			
O15525	Transcription factor MafG	MAFG			
Q9H063	Repressor of RNA polymerase III transcription MAF1 homolog	MAF1			
Q8N7X4	Melanoma-associated antigen B6	MAGEB6			
Q8IWC1	MAP7 domain-containing protein 3	MAP7D3			
P15529	Membrane cofactor protein	CD46			
Q8TDD5	Mucolipin-3	MCOLN3			
Q71SY5	Mediator of RNA polymerase II transcription subunit 25	MED25			
Q96JN8	Neuralized-like protein 4	NEURL4			
Q15466	Nuclear receptor subfamily 0 group B member 2	NR0B2			
P49281	Natural resistance-associated macrophage protein 2	SLC11A2			
O15259	Nephrocystin-1	NPHP1			
P17677	Neuromodulin	GAP43			
P20393	Nuclear receptor subfamily 1 group D member 1	NR1D1			
Q7Z494	Nephrocystin-3	NPHP3			
Q99519	Sialidase-1	NEU1			
O75161	Nephrocystin-4	NPHP4			
P48681	Nestin	NES			
Q9Y3R4	Sialidase-2	NEU2			
Q9UND3	Nuclear pore complex-interacting protein family member A1	NPIPA1			

E9PIF3	Nuclear pore complex-interacting protein family member A2	NPIPA2			
F8WFD2	Nuclear pore complex-interacting protein family member A3	NPIPA3			
A8MRT5	Nuclear pore complex-interacting protein family member B5	NPIPB5			
E9PJ23	Nuclear pore complex-interacting protein family member B6	NPIPB6			
Q8WWR8	Sialidase-4	NEU4			
Q9NP71	Carbohydrate-responsive element-binding protein	MLXIPL			
E9PQR5	Nuclear pore complex-interacting protein family member B8	NPIPB8			
F8W1W9	Nuclear pore complex-interacting protein family member B9	NPIPB9			
Q99574	Neuroserpin	SERPINI1			
Q9H9Q4	Non-homologous end-joining factor 1	NHEJ1			
Q86UQ8	Transcription factor NF-E4	NFE4			
P52849	Bifunctional heparan sulfate N-deacetylase/N-sulfotransferase 2	NDST2			
O14524	Nuclear envelope integral membrane protein 1	NEMP1			
O95803	Bifunctional heparan sulfate N-deacetylase/N-sulfotransferase 3	NDST3			
A6NFY4	Nuclear envelope integral membrane protein 2	NEMP2			
Q9H3R1	Bifunctional heparan sulfate N-deacetylase/N-sulfotransferase 4	NDST4			
Q9Y5Y2	Cytosolic Fe-S cluster assembly factor NUBP2	NUBP2			
Q9Y4U1	Cyanocobalamin reductase / alkylcobalamin dealkylase	MMACHC			
O75030	Microphthalmia-associated transcription factor	MITF			
P28482	Mitogen-activated protein kinase 1	MAPK1			
Q5VZR2	NUT family member 2G	NUTM2G			
Q8NHB8	Olfactory receptor 5K2	OR5K2			
Q8NGW6	Olfactory receptor 6K6	OR6K6			
O15504	Nucleoporin NUP42	NUP42			
Q9Y5G8	Protocadherin gamma-A5	PCDHGA5			
Q5XG87	Terminal nucleotidyltransferase 4A	TENT4A			
Q9UN71	Protocadherin gamma-B4	PCDHGB4			
Q9BWT3	Poly	PAPOLG			
O60330	Protocadherin gamma-A12	PCDHGA12			
Q8NGK5	Olfactory receptor 52M1	OR52M1			
P40424	Pre-B-cell leukemia transcription factor 1	PBX1			
A6NKN8	Purkinje cell protein 4-like protein 1	PCP4L1			
A6NGG8	Photoreceptor cilium actin regulator	PCARE			
Q9UNK4	Group IID secretory phospholipase A2	PLA2G2D			
P05121	Plasminogen activator inhibitor 1	SERPINE1			
Q9Y5E1	Protocadherin beta-9	PCDHB9			
Q9Y5F1	Protocadherin beta-12	PCDHB12			
Q9Y5F0	Protocadherin beta-13	PCDHB13			
P09874	Poly	PARP1			
Q8WYR1	Phosphoinositide 3-kinase regulatory subunit 5	PIK3R5			
O43692	Peptidase inhibitor 15	PI15			
Q96LB9	Peptidoglycan recognition protein 3	PGLYRP3			
Q9BRR3	Post-GPI attachment to proteins factor 4	PGAP4			
Q92769	Histone deacetylase 2	HDAC2			

Q9BSE4	Homocysteine-responsive endoplasmic reticulum-resident ubiquitin-like domain member 2 protein	HERPUD2			
Q9BW71	HIRA-interacting protein 3	HIRIP3			
Q8TDG4	Helicase POLQ-like	HELQ			
P31274	Homeobox protein Hox-C9	HOXC9			
P23083	Immunoglobulin heavy variable 1-2	IGHV1-2			
P01763	Immunoglobulin heavy variable 3-48	IGHV3-48			
Q9Y5N1	Histamine H3 receptor	HRH3			
A8MTL9	Serpin-like protein HMSD	HMSD			
P05113	Interleukin-5	IL5			
P08887	Interleukin-6 receptor subunit alpha	IL6R			
O00479	High mobility group nucleosome-binding domain-containing protein 4	HMGN4			
Q9Y547	Intraflagellar transport protein 25 homolog	HSPB11			
P27352	Cobalamin binding intrinsic factor	CBLIF			
P32942	Intercellular adhesion molecule 3	ICAM3			
O15131	Importin subunit alpha-6	KPNA5			
Q9Y2F5	Little elongation complex subunit 1	ICE1			
P26951	Interleukin-3 receptor subunit alpha	IL3RA			
Q02363	DNA-binding protein inhibitor ID-2	ID2			
Q14164	Inhibitor of nuclear factor kappa-B kinase subunit epsilon	IKBKE			
P47928	DNA-binding protein inhibitor ID-4	ID4			
O60841	Eukaryotic translation initiation factor 5B	EIF5B			
Q5JU85	IQ motif and SEC7 domain-containing protein 2	IQSEC2			
Q3MIP1	Inositol 1,4,5-trisphosphate receptor-interacting protein-like 2	ITPRIPL2			
Q96SY0	Integrator complex subunit 14	INTS14			
Q92952	Small conductance calcium-activated potassium channel protein 1	KCNN1			
Q8N7Y1	Putative uncharacterized protein KIRREL3-AS3	KIRREL3-AS3			
P14618	Pyruvate kinase PKM	PKM			
Q9NYG8	Potassium channel subfamily K member 4	KCNK4			
Q86Y91	Kinesin-like protein KIF18B	KIF18B			
Q96E29	Transcription termination factor 3, mitochondrial	MTERF3			
Q13330	Metastasis-associated protein MTA1	MTA1			
P47944	Metallothionein-4	MT4			
A1L3X4	Putative metallothionein MT1DP	MT1DP			
Q8IVL1	Neuron navigator 2	NAV2			
P16333	Cytoplasmic protein NCK1	NCK1			
Q9UI40	Sodium/potassium/calcium exchanger 2	SLC24A2			
Q8WY64	E3 ubiquitin-protein ligase MYLIP	MYLIP			
Q6ZUF6	Putative uncharacterized protein encoded by LINC00336	LINC00336			
Q08AG7	Mitotic-spindle organizing protein 1	MZT1			
Q9Y6Q9	Nuclear receptor coactivator 3	NCOA3			
Q5VXU1	Sodium/potassium-transporting ATPase subunit beta-1-interacting protein 2	NKAIN2			

Q9UD57	NK1 transcription factor-related protein 2	NKX1-2			
Q6ZNB6	NF-X1-type zinc finger protein NFXL1	NFXL1			
Q9NVZ3	Adaptin ear-binding coat-associated protein 2	NECAP2			
Q96PU5	E3 ubiquitin-protein ligase NEDD4-like	NEDD4L			
Q9UK39	Nocturnin	NOCT			
Q96IV0	Peptide-N	NGLY1			
Q9UJF2	Ras GTPase-activating protein nGAP	RASAL2			
Q6W5P4	Neuropeptide S receptor	NPSR1			
Q8IUM7	Neuronal PAS domain-containing protein 4	NPAS4			
Q06495	Sodium-dependent phosphate transport protein 2A	SLC34A1			
Q9UMY1	Nucleolar protein 7	NOL7			
Q5SY16	Polynucleotide 5'-hydroxyl-kinase NOL9	NOL9			
A6NHN6	Nuclear pore complex-interacting protein family member B15	NPIPB15			
P25929	Neuropeptide Y receptor type 1	NPY1R			
P01303	Pro-neuropeptide Y	NPY			
Q96FI4	Endonuclease 8-like 1	NEIL1			
O95167	NADH dehydrogenase	NDUFA3			
Q8WY50	Placenta-specific protein 4	PLAC4			
Q8NGR4	Olfactory receptor 5C1	OR5C1			
Q8NGP6	Olfactory receptor 5M8	OR5M8			
Q8NH06	Olfactory receptor 1P1	OR1P1			
Q8NGQ1	Olfactory receptor 9G4	OR9G4			
Q9H0X9	Oxysterol-binding protein-related protein 5	OSBPL5			
Q9NZ53	Podocalyxin-like protein 2	PODXL2			
Q9H4B4	Serine/threonine-protein kinase PLK3	PLK3			
C9JH25	Proline-rich transmembrane protein 4	PRRT4			
Q86WI1	Fibrocystin-L	PKHD1L1			
P62191	26S proteasome regulatory subunit 4	PSMC1			
O43172	U4/U6 small nuclear ribonucleoprotein Prp4	PRPF4			
Q5T8A7	Protein phosphatase 1 regulatory subunit 26	PPP1R26			
A3QJZ7	PRAME family member 27	PRAMEF27			
Q8NCQ7	Protein PROCA1	PROCA1			
P51888	Prolargin	PRELP			
Q9UBK2	Peroxisome proliferator-activated receptor gamma coactivator 1-alpha	PPARGC1A			
Q9Y248	DNA replication complex GINS protein PSF2	GINS2			
P25105	Platelet-activating factor receptor	PTAFR			
A5PKW4	PH and SEC7 domain-containing protein 1	PSD			
P31939	Bifunctional purine biosynthesis protein ATIC	ATIC			
O14649	Potassium channel subfamily K member 3	KCNK3			
P20151	Kallikrein-2	KLK2			
Q9UQM7	Calcium/calmodulin-dependent protein kinase type II subunit alpha	CAMK2A			
Q14657	EKC/KEOPS complex subunit LAGE3	LAGE3			
Q9UNZ5	Leydig cell tumor 10 kDa protein homolog	C19orf53			
P53671	LIM domain kinase 2	LIMK2			

O75473	Leucine-rich repeat-containing G-protein coupled receptor 5	LGR5			
A6NJI9	Leucine-rich repeat-containing protein 72	LRRC72			
Q96DD0	Leucine-rich repeat-containing protein 39	LRRC39			
A6NIV6	Leucine-rich repeat and IQ domain-containing protein 4	LRRIQ4			
Q969R5	Lethal	L3MBTL2			
Q16553	Lymphocyte antigen 6E	LY6E			
Q8WVP7	Limb region 1 protein homolog	LMBR1			
Q96KR4	Leishmanolysin-like peptidase	LMLN			
P49901	Sperm mitochondrial-associated cysteine-rich protein	SMCP			
Q9Y2E5	Epididymis-specific alpha-mannosidase	MAN2B2			
Q9NWR8	Calcium uniporter regulatory subunit MCUb, mitochondrial	MCUB			
Q96N66	Lysophospholipid acyltransferase	MBOAT7			
Q3V5L5	Alpha-1,6-mannosylglycoprotein 6-beta-N-acetylglucosaminyltransferase B	MGAT5B			
Q7RTP6		MICAL3			
Q9UQ53	Alpha-1,3-mannosyl-glycoprotein 4-beta-N-acetylglucosaminyltransferase B	MGAT4B			
Q16891	MICOS complex subunit MIC60	IMMT			
O43196	MutS protein homolog 5	MSH5			
P69208	Morphogenetic neuropeptide				
P00540	Proto-oncogene serine/threonine-protein kinase mos	MOS			
Q5T089	MORN repeat-containing protein 1	MORN1			
E2RYF6	Mucin-22	MUC22			
Q969V5	Mitochondrial ubiquitin ligase activator of NFKB 1	MUL1			
P24844	Myosin regulatory light polypeptide 9	MYL9			
Q3BBV0	Neuroblastoma breakpoint family member 1	NBPF1			
Q15788	Nuclear receptor coactivator 1	NCOA1			
Q7RTR2	NLR family CARD domain-containing protein 3	NLRC3			
Q9ULJ8	Neurabin-1	PPP1R9A			
O14513	Nck-associated protein 5	NCKAP5			
Q86UR1	NADPH oxidase activator 1	NOXA1			
Q9Y5S8	NADPH oxidase 1	NOX1			
P41219	Peripherin	PRPH			
P61581	Endogenous retrovirus group K member 24 Np9 protein	ERVK-24			
Q14938	Nuclear factor 1 X-type	NFIX			
Q05586	Glutamate receptor ionotropic, NMDA 1	GRIN1			
P07197	Neurofilament medium polypeptide	NEFM			
Q99463	Putative neuropeptide Y receptor type 6	NPY6R			
P10588	Nuclear receptor subfamily 2 group F member 6	NR2F6			
Q9GZT8	NIF3-like protein 1	NIF3L1			
Q8TDF5	Neuropilin and tolloid-like protein 1	NETO1			
Q9UGV2	Protein NDRG3	NDRG3			
Q8WVJ2	NudC domain-containing protein 2	NUDCD2			
P34982	Olfactory receptor 1D2	OR1D2			
Q8NHC8	Olfactory receptor 2T6	OR2T6			

P29803	Pyruvate dehydrogenase E1 component subunit alpha, testis-specific form, mitochondrial	PDHA2			
Q96PE5	Opalin	OPALIN			
Q8NGG0	Olfactory receptor 8J3	OR8J3			
Q8NH10	Olfactory receptor 8U1	OR8U1			
Q8N8J0	Putative inactive phosphatidylinositol 4-kinase alpha-like protein P1	PI4KAP1			
Q7Z7B1	Phosphatidylinositol-glycan biosynthesis class W protein	PIGW			
A6NFU8	Pyroglutamyl-peptidase 1-like protein	PGPEP1L			
Q53GA4	Pleckstrin homology-like domain family A member 2	PHLDA2			
Q9Y5J5	Pleckstrin homology-like domain family A member 3	PHLDA3			
Q96GD0	Pyridoxal phosphate phosphatase	PDXP			
P63128	Endogenous retrovirus group K member 9 Pol protein	ERVK-9			
P02776	Platelet factor 4	PF4			
Q8N819	Probable protein phosphatase 1N	PPM1N			
Q13522	Protein phosphatase 1 regulatory subunit 1A	PPP1R1A			
P13797	Plastin-3	PLS3			
Q6ZMI0	Protein phosphatase 1 regulatory subunit 21	PPP1R21			
Q8N3E9	1-phosphatidylinositol 4,5-bisphosphate phosphodiesterase delta-3	PLCD3			
Q6ZR37	Pleckstrin homology domain-containing family G member 7	PLEKHG7			
Q15111	Inactive phospholipase C-like protein 1	PLCL1			
O75569	Interferon-inducible double-stranded RNA-dependent protein kinase activator A	PRKRA			
Q8IYP2	Serine protease 58	PRSS58			
Q5VTL8	Pre-mRNA-splicing factor 38B	PRPF38B			
P86478	Proline-rich protein 20E	PRR20E			
Q96S44	EKC/KEOPS complex subunit TP53RK	TP53RK			
Q5VWM6	Putative PRAME family member	PRAMEF13			
O43272	Proline dehydrogenase 1, mitochondrial	PRODH			
Q9Y520	Protein PRRC2C	PRRC2C			
Q13162	Peroxiredoxin-4	PRDX4			
Q6H9L7	Isthmin-2	ISM2			
Q7Z3Z0	Keratin, type I cytoskeletal 25	KRT25			
P57087	Junctional adhesion molecule B	JAM2			
Q6PF15	Kelch-like protein 35	KLHL35			
Q2WGJ6	Kelch-like protein 38	KLHL38			
Q9Y6M4	Casein kinase I isoform gamma-3	CSNK1G3			
Q13555	Calcium/calmodulin-dependent protein kinase type II subunit gamma	CAMK2G			
Q86Z14	Beta-klotho	KLB			
A0A0U1RQI7	Kruppel-like factor 18	KLF18			
Q9BQ90	Kelch domain-containing protein	KLHDC3			
O43790	Keratin, type II cuticular Hb6	KRT86			
P01597	Immunoglobulin kappa variable 1-39	IGKV1-39			
P01042	Kininogen-1	KNG1			
Q9BUR5	MICOS complex subunit MIC26	APOO			
Q8N3J2	N	METTL4			
Q9Y4C4	Malignant fibrous histiocytoma-amplified sequence 1	MFHAS1			

Q9UKD2	mRNA turnover protein 4 homolog	MRTO4			
Q9HC36	rRNA methyltransferase 3, mitochondrial	MRM3			
P07438	Metallothionein-1B	MT1B			
Q3MIW9	Mucin-like protein 3	MUCL3			
P46734	Dual specificity mitogen-activated protein kinase kinase 3	MAP2K3			
Q96RE7	Nucleus accumbens-associated protein 1	NACC1			
P52961	GPI-linked NAD	ART1			
A2RRP1	Neuroblastoma-amplified sequence	NBAS			
Q9UHQ9	NADH-cytochrome b5 reductase 1	CYB5R1			
Q9H094	Neuroblastoma breakpoint family member 3	NBPF3			
Q9BTX1	Nucleoporin NDC1	NDC1			
Q13203	Myosin-binding protein H	MYBPH			
P10244	Myb-related protein B	MYBL2			
Q9UKZ9	Procollagen C-endopeptidase enhancer 2	PCOLCE2			
Q15119		PDK2			
Q9BQ51	Programmed cell death 1 ligand 2	PDCD1LG2			
Q8IVA1	Purkinje cell protein 2 homolog	PCP2			
Q9Y5Y4	Prostaglandin D2 receptor 2	PTGDR2			
Q9Y5N5	Methyltransferase N6AMT1	N6AMT1			
P20591	Interferon-induced GTP-binding protein Mx1	MX1			
Q99836	Myeloid differentiation primary response protein MyD88	MYD88			
Q71RS6	Sodium/potassium/calcium exchanger 5	SLC24A5			
P51957	Serine/threonine-protein kinase Nek4	NEK4			
P51956	Serine/threonine-protein kinase Nek3	NEK3			
Q92859	Neogenin	NEO1			
Q16718	NADH dehydrogenase	NDUFA5			
Q9P0J0	NADH dehydrogenase	NDUFA13			
Q8NGY6	Olfactory receptor 6N2	OR6N2			
Q14990	Outer dense fiber protein 1	ODF1			
Q9BRP0	Transcription factor Ovo-like 2	OVOL2			
Q9Y530	ADP-ribose glycohydrolase OARD1	OARD1			
Q8NGP8	Olfactory receptor 5M1	OR5M1			
Q9H649	tRNA	NSUN3			
Q12908	Ileal sodium/bile acid cotransporter	SLC10A2			
Q8NGJ7	Olfactory receptor 51A2	OR51A2			
Q8NH70	Olfactory receptor 4A16	OR4A16			
Q8NH95	Putative olfactory receptor 13C6	OR13C6P			
Q99571	P2X purinoceptor 4	P2RX4			
Q96KK4	Olfactory receptor 10C1	OR10C1			
Q8NGS7	Olfactory receptor 13C8	OR13C8			
O15547	P2X purinoceptor 6	P2RX6			
P10451	Osteopontin	SPP1			
P21953	2-oxoisovalerate dehydrogenase subunit beta, mitochondrial	BCKDHB			
Q15645	Pachytene checkpoint protein 2 homolog	TRIP13			
Q4VXU2	Polyadenylate-binding protein 1-like	PABPC1L			
Q5JQF8	Polyadenylate-binding protein 1-like 2	PABPC1L2A			
Q9NRJ5	Poly	PAPOLB			
Q8NG83	Olfactory receptor 2M3	OR2M3			
Q9Y5E5	Protocadherin beta-4	PCDHB4			

Q6P996	Pyridoxal-dependent decarboxylase domain-containing protein 1	PDXDC1			
Q9BY77	Polymerase delta-interacting protein 3	POLDIP3			
Q8N8D1	Programmed cell death protein 7	PDCD7			
Q8NBR0	Tumor protein p53-inducible protein 13	TP53I13			
Q9UIL8	PHD finger protein 11	PHF11			
Q8IV76	Circadian clock protein PASD1	PASD1			
P10619	Lysosomal protective protein	CTSA			
O95397	Putative protein PLEKHA9	PLEKHA8P1			
P0CW18	Serine protease 56	PRSS56			
P78386	Keratin, type II cuticular Hb5	KRT85			
P51787	Potassium voltage-gated channel subfamily KQT member 1	KCNQ1			
O60870	DNA/RNA-binding protein KIN17	KIN			
P59991	Keratin-associated protein 12-2	KRTAP12-2			
Q99712	ATP-sensitive inward rectifier potassium channel 15	KCNJ15			
Q16774	Guanylate kinase	GUK1			
Q8IUG1	Keratin-associated protein 1-3	KRTAP1-3			
P26371	Keratin-associated protein 5-9	KRTAP5-9			
A0AUZ9	KAT8 regulatory NSL complex subunit 1-like protein	KANSL1L			
Q7L273	BTB/POZ domain-containing protein KCTD9	KCTD9			
Q9Y6C7	Putative uncharacterized protein encoded by LINC00312	LINC00312			
Q13753	Laminin subunit gamma-2	LAMC2			
P06239	Tyrosine-protein kinase Lck	LCK			
Q96PV6	Leukocyte receptor cluster member 8	LENG8			
A6PVS8	Leucine-rich repeat and IQ domain-containing protein 3	LRRIQ3			
P58215	Lysyl oxidase homolog 3	LOXL3			
Q9Y608	Leucine-rich repeat flightless-interacting protein 2	LRRFIP2			
P20774	Mimecan	OGN			
Q9NQG6	Mitochondrial dynamics protein MID51	MIEF1			
Q14165	Malectin	MLEC			
A0A1B0GUY1	MARCO-like protein	MARCOL			
Q9UBG0	C-type mannose receptor 2	MRC2			
Q8N3F0	Maturin	MTURN			
A4FU01	Myotubularin-related protein 11	MTMR11			
Q9UHE5	N-acetyltransferase 8	NAT8			
Q13765	Nascent polypeptide-associated complex subunit alpha	NACA			
Q9UK23	N-acetylglucosamine-1-phosphodiester alpha-N-acetylglucosaminidase	NAGPA			
O96009	Napsin-A	NAPSA			
P20592	Interferon-induced GTP-binding protein Mx2	MX2			
Q5TI25	Neuroblastoma breakpoint family member 14	NBPF14			
P52298	Nuclear cap-binding protein subunit 2	NCBP2			
O00541	Pescadillo homolog	PES1			
Q9HCH0	Nck-associated protein 5-like	NCKAP5L			
P26718	NKG2-D type II integral membrane protein	KLRK1			
P21452	Substance-K receptor	TACR2			
Q969F2	Protein naked cuticle homolog 2	NKD2			
Q8IVV8	Sodium/potassium-transporting ATPase subunit beta-1-interacting protein 4	NKAIN4			

P29120	Neuroendocrine convertase 1	PCSK1			
P78426	Homeobox protein Nkx-6.1	NKX6-1			
Q6NVV3	Magnesium transporter NIPA3	NIPAL1			
O15118	NPC intracellular cholesterol transporter 1	NPC1			
O94818	Nucleolar protein 4	NOL4			
P40261	Nicotinamide N-methyltransferase	NNMT			
P48145	Neuropeptides B/W receptor type 1	NPBWR1			
Q8N183	NADH dehydrogenase	NDUFAF2			
Q9Y3C1	Nucleolar protein 16	NOP16			
P08473	Neprilysin	MME			
Q6PJE2	POM121 and ZP3 fusion protein	POMZP3			
P30990	Neurotensin/neuromedin N	NTS			
O95897	Noelin-2	OLFM2			
Q9GZK4	Olfactory receptor 2H1	OR2H1			
Q8WZ84	Olfactory receptor 8D1	OR8D1			
Q8IYS1	Peptidase M20 domain-containing protein 2	PM20D2			
Q16822	Phosphoenolpyruvate carboxykinase	PCK2			
Q9BRP1	Programmed cell death protein 2-like	PDCD2L			
Q9NPJ4	Proline-rich nuclear receptor coactivator 2	PNRC2			
Q9UNP9	Peptidyl-prolyl cis-trans isomerase E	PPIE			
O96001	Protein phosphatase 1 regulatory subunit 17	PPP1R17			
Q9NYT0	Pleckstrin-2	PLEK2			
P12277	Creatine kinase B-type	CKB			
Q99542	Matrix metalloproteinase-19	MMP19			
P08254	Stromelysin-1	MMP3			
Q8IWD5	Major facilitator superfamily domain-containing protein 6-like	MFSD6L			
Q10469	Alpha-1,6-mannosyl-glycoprotein 2-beta-N-acetylglucosaminyltransferase	MGAT2			
P39210	Protein Mpv17	MPV17			
O43482	Protein Mis18-beta	OIP5			
P12872	Promotilin	MLN			
Q9ULC0	Endomucin	EMCN			
Q9NZV6	Methionine-R-sulfoxide reductase B1	MSRB1			
P49286	Melatonin receptor type 1B	MTNR1B			
Q9UPR5	Sodium/calcium exchanger 2	SLC8A2			
Q96HR8	H/ACA ribonucleoprotein complex non-core subunit NAF1	NAF1			
Q8TBE9	N-acylneuraminate-9-phosphatase	NANP			
Q8WY41	Nanos homolog 1	NANOS1			
Q8N159	N-acetylglutamate synthase, mitochondrial	NAGS			
O95613	Pericentrin	PCNT			
Q69YI7	Nuclear apoptosis-inducing factor 1	NAIF1			
Q96QE2	Proton myo-inositol cotransporter	SLC2A13			
Q9UIF7	Adenine DNA glycosylase	MUTYH			
Q96JP2	Unconventional myosin-XVB	MYO15B			
P32418	Sodium/calcium exchanger 1	SLC8A1			
O95544	NAD kinase	NADK			
P19878	Neutrophil cytosol factor 2	NCF2			
Q5TAG4	Neuroblastoma breakpoint family member 12	NBPF12			
Q15080	Neutrophil cytosol factor 4	NCF4			
P19404	NADH dehydrogenase	NDUFV2			
P52179	Myomesin-1	MYOM1			
Q9NP64	Nucleolar protein of 40 kDa	ZCCHC17			
Q9BPW8	Protein NipSnap homolog 1	NIPSNAP1			
P46934	E3 ubiquitin-protein ligase NEDD4	NEDD4			

Q96P71	N-terminal EF-hand calcium-binding protein 3	NECAB3			
Q8NFZ3	Neuroligin-4, Y-linked	NLGN4Y			
P48146	Neuropeptides B/W receptor type 2	NPBWR2			
O96000	NADH dehydrogenase	NDUFB10			
O60551	Glycylpeptide N-tetradecanoyltransferase 2	NMT2			
Q330K2	NADH dehydrogenase	NDUFAF6			
O75306	NADH dehydrogenase	NDUFS2			
Q14207	Protein NPAT	NPAT			
Q14494	Endoplasmic reticulum membrane sensor NFE2L1	NFE2L1			
P21359	Neurofibromin	NF1			
Q8IXH7	Negative elongation factor C/D	NELFCD			
Q9ULP0	Protein NDRG4	NDRG4			
Q9NQ35	Nuclear receptor-interacting protein 3	NRIP3			
P0C025	Nucleoside diphosphate-linked moiety X motif 17	NUDT17			
Q13416	Origin recognition complex subunit 2	ORC2			
O60412	Olfactory receptor 7C2	OR7C2			
P41143	Delta-type opioid receptor	OPRD1			
A6NL26	Olfactory receptor 5B21	OR5B21			
Q8NH87	Olfactory receptor 9G1	OR9G1			
Q8NGV0	Olfactory receptor 2Y1	OR2Y1			
Q8NFH4	Nucleoporin Nup37	NUP37			
Q9NZP5	Olfactory receptor 5AC2	OR5AC2			
A0A096LPK9	Olfactory receptor 4N4C	OR4N4C			
O95206	Protocadherin-8	PCDH8			
Q3KNV8	Polycomb group RING finger protein 3	PCGF3			
Q9H4B0	Probable tRNA N6-adenosine threonylcarbamoyltransferase, mitochondrial	OSGEPL1			
Q86WK9	Membrane progestin receptor alpha	PAQR7			
P35227	Polycomb group RING finger protein 2	PCGF2			
Q9NPB6	Partitioning defective 6 homolog alpha	PARD6A			
Q9Y5F2	Protocadherin beta-11	PCDHB11			
Q9BYG4	Partitioning defective 6 homolog gamma	PARD6G			
Q9UN74	Protocadherin alpha-4	PCDHA4			
Q8NC51	Plasminogen activator inhibitor 1 RNA-binding protein	SERBP1			
Q96RV3	Pecanex-like protein 1	PCNX1			
Q9Y5H5	Protocadherin alpha-9	PCDHA9			
Q9NXG6	Transmembrane prolyl 4-hydroxylase	P4HTM			
Q14554	Protein disulfide-isomerase A5	PDIA5			
Q9P2J9		PDP2			
Q9Y2S7	Polymerase delta-interacting protein 2	POLDIP2			
Q96GU1	P antigen family member 5	PAGE5			
O15460	Prolyl 4-hydroxylase subunit alpha-2	P4HA2			
Q99447	Ethanolamine-phosphate cytidylyltransferase	PCYT2			
Q8NBM8	Prenylcysteine oxidase-like	PCYOX1L			
Q58A44	Prostate collagen triple helix protein	PCOTH			
Q8NDX5	Polyhomeotic-like protein 3	PHC3			
P80108	Phosphatidylinositol-glycan-specific phospholipase D	GPLD1			

Q96BK5	PIN2/TERF1-interacting telomerase inhibitor 1	PINX1			
P05164	Myeloperoxidase	MPO			
P54278	Mismatch repair endonuclease PMS2	PMS2			
Q8NBV4	Inactive phospholipid phosphatase 7	PLPP7			
Q7Z6Z6	Patatin-like phospholipase domain-containing protein 5	PNPLA5			
P01602	Immunoglobulin kappa variable 1-5	IGKV1-5			
Q8NC56	LEM domain-containing protein 2	LEMD2			
Q9Y4W2	Ribosomal biogenesis protein LAS1L	LAS1L			
P04180	Phosphatidylcholine-sterol acyltransferase	LCAT			
Q9UIQ6	Leucyl-cystinyl aminopeptidase	LNPEP			
Q9Y4X4	Krueppel-like factor 12	KLF12			
Q9HBW1	Leucine-rich repeat-containing protein 4	LRRC4			
O43325	LYR motif-containing protein 1	LYRM1			
P01705	Immunoglobulin lambda variable 2-23	IGLV2-23			
P59942	Mitochondrial coiled-coil domain protein 1	MCCD1			
Q96A72	Protein mago nashi homolog 2	MAGOHB			
Q9NRN9	rRNA N6-adenosine-methyltransferase METTL5	METTL5			
Q6UXV4	MICOS complex subunit MIC27	APOOL			
Q9UNW1	Multiple inositol polyphosphate phosphatase 1	MINPP1			
P50219	Motor neuron and pancreas homeobox protein 1	MNX1			
O15439	Multidrug resistance-associated protein 4	ABCC4			
Q96BY2	Modulator of apoptosis 1	MOAP1			
P35548	Homeobox protein MSX-2	MSX2			
P55157	Microsomal triglyceride transfer protein large subunit	MTTP			
Q9Y4I5	Tesmin	TESMIN			
Q9H3R2	Mucin-13	MUC13			
Q6ZS30	Neurobeachin-like protein 1	NBEAL1			
Q9H0A0	RNA cytidine acetyltransferase	NAT10			
Q96NK8	Neurogenic differentiation factor	NEUROD6			
O15049	NEDD4-binding protein 3	N4BP3			
Q9BXJ9	N-alpha-acetyltransferase 15, NatA auxiliary subunit	NAA15			
Q9P2K5	Myelin expression factor 2	MYEF2			
Q9BSU3	N-alpha-acetyltransferase 11	NAA11			
Q9HB07	MYG1 exonuclease	MYG1			
Q7Z406	Myosin-14	MYH14			
Q15742	NGFI-A-binding protein 2	NAB2			
P02689	Myelin P2 protein	PMP2			
P12036	Neurofilament heavy polypeptide	NEFH			
Q9HC98	Serine/threonine-protein kinase Nek6	NEK6			
Q8TAT5	Endonuclease 8-like 3	NEIL3			
Q9Y585	Olfactory receptor 1A2	OR1A2			
A6NET4	Olfactory receptor 5K3	OR5K3			
P23760	Paired box protein Pax-3	PAX3			
P14859	POU domain, class 2, transcription factor 1	POU2F1			
Q9UD71	Protein phosphatase 1 regulatory subunit 1B	PPP1R1B			
Q9H875	PRKR-interacting protein 1	PRKRIP1			
Q5SGD2	Protein phosphatase 1L	PPM1L			
P67775	Serine/threonine-protein phosphatase 2A catalytic subunit alpha isoform	PPP2CA			

P42336	Phosphatidylinositol 4,5-bisphosphate 3-kinase catalytic subunit alpha isoform	PIK3CA			
Q96T49	Protein phosphatase 1 regulatory inhibitor subunit 16B	PPP1R16B			
O60240	Perilipin-1	PLIN1			
Q16512	Serine/threonine-protein kinase N1	PKN1			
A6NEC2	Puromycin-sensitive aminopeptidase-like protein	NPEPPSL1			
Q9NW61	Pleckstrin homology domain-containing family J member 1	PLEKHJ1			
Q96S07	Proline-rich protein 25	PRR25			
O43464	Serine protease HTRA2, mitochondrial	HTRA2			
Q9Y251	Heparanase	HPSE			
P01762	Immunoglobulin heavy variable 3-11	IGHV3-11			
A0A0C4DH32	Immunoglobulin heavy variable 3-20	IGHV3-20			
Q9UBY9	Heat shock protein beta-7	HSPB7			
Q03933	Heat shock factor protein 2	HSF2			
P35914	Hydroxymethylglutaryl-CoA lyase, mitochondrial	HMGCL			
P82970	High mobility group nucleosome-binding domain-containing protein 5	HMGN5			
Q9P2X3	Protein IMPACT	IMPACT			
O15111	Inhibitor of nuclear factor kappa-B kinase subunit alpha	CHUK			
Q9NYY1	Interleukin-20	IL20			
Q9UBP8	Kidney-associated antigen 1	KAAG1			
P05783	Keratin, type I cytoskeletal 18	KRT18			
Q5JT82	Krueppel-like factor 17	KLF17			
A0A0A0MT89	Immunoglobulin kappa joining 1	IGKJ1			
Q9ULH0	Kinase D-interacting substrate of 220 kDa	KIDINS220			
O14901	Krueppel-like factor 11	KLF11			
O60333	Kinesin-like protein KIF1B	KIF1B			
Q96RP8	Potassium voltage-gated channel subfamily A member 7	KCNA7			
P04433	Immunoglobulin kappa variable 3-11	IGKV3-11			
Q3ZCW2	Galectin-related protein	LGALSL			
Q9UHB6	LIM domain and actin-binding protein 1	LIMA1			
A8MUM7	Galectin-16	LGALS16			
Q96GY3	Protein lin-37 homolog	LIN37			
Q5T749	Keratinocyte proline-rich protein	KPRP			
F2Z398	LMO7 downstream neighbor protein	LMO7DN			
Q9P127	Leucine zipper protein 4	LUZP4			
Q6UXB3	Ly6/PLAUR domain-containing protein 2	LYPD2			
Q9NR34	Mannosyl-oligosaccharide 1,2-alpha-mannosidase IC	MAN1C1			
Q8NB16	Mixed lineage kinase domain-like protein	MLKL			
Q99707	Methionine synthase	MTR			
P08253	72 kDa type IV collagenase	MMP2			
Q86YR7	Probable guanine nucleotide exchange factor MCF2L2	MCF2L2			
Q86VX9	Vacuolar fusion protein MON1 homolog A	MON1A			
P34949	Mannose-6-phosphate isomerase	MPI			
A6NHM9	Putative DBH-like monooxygenase protein 2	MOXD2P			
Q6ZSM3	Monocarboxylate transporter 12	SLC16A12			

P03891	NADH-ubiquinone oxidoreductase chain 2	MT-ND2			
Q3ZCN5	Otogelin-like protein	OTOGL			
O43612	Orexin	HCRT			
Q8NHB1	Olfactory receptor 2V1	OR2V1			
A8MW92	PHD finger protein 20-like protein 1	PHF20L1			
Q6TGC4	Protein-arginine deiminase type-	PADI6			
Q15004	PCNA-associated factor	PCLAF			
Q8N7B6	PACRG-like protein	PACRGL			
Q86V24	Adiponectin receptor protein 2	ADIPOR2			
Q7Z2X7	P antigen family member 2	PAGE2			
Q9Y5I1	Protocadherin alpha-11	PCDHA11			
Q9NRA1	Platelet-derived growth factor C	PDGFC			
Q9NQU5	Serine/threonine-protein kinase PAK 6	PAK6			
Q9H6A9	Pecanex-like protein 3	PCNX3			
Q96LB8	Peptidoglycan recognition protein 4	PGLYRP4			
Q6IN85	Serine/threonine-protein phosphatase 4 regulatory subunit 3A	PPP4R3A			
Q13608	Peroxisome assembly factor 2	PEX6			
Q9ULL4	Plexin-B3	PLXNB3			
Q5EBL8	PDZ domain-containing protein 11	PDZD11			
Q9UL42	Paraneoplastic antigen Ma2	PNMA2			
Q6S8J3	POTE ankyrin domain family member E	POTEE			
Q6PEZ8	Podocan-like protein 1	PODNL1			
Q9H237	Protein-serine O-palmitoleoyltransferase porcupine	PORCN			
A8MPX8	Protein phosphatase 2C-like domain-containing protein 1	PP2D1			
Q8IVE3	Pleckstrin homology domain-containing family H member 2	PLEKHH2			
Q7Z443	Polycystic kidney disease protein 1-like 3	PKD1L3			
Q9BQR3	Serine protease 27	PRSS27			
O95084	Serine protease 23	PRSS23			
P54821	Paired mesoderm homeobox protein 1	PRRX1			
Q15735	Phosphatidylinositol 4,5-bisphosphate 5-phosphatase A	INPP5J			
P23759	Paired box protein Pax-7	PAX7			
Q9BUH6	Protein PAXX	PAXX			
O75381	Peroxisomal membrane protein PEX14	PEX14			
Q96T60	Bifunctional polynucleotide phosphatase/kinase	PNKP			
P35408	Prostaglandin E2 receptor EP4 subtype	PTGER4			
O43808	Peroxisomal membrane protein PMP34	SLC25A17			
A2A3K4	Protein tyrosine phosphatase domain-containing protein 1	PTPDC1			
Q9Y6J8	Serine/threonine/tyrosine-interacting-like protein 1	STYXL1			
Q16831	Uridine phosphorylase 1	UPP1			
Q96RI9	Trace amine-associated receptor 9	TAAR9			
Q9P1P5	Trace amine-associated receptor 2	TAAR2			
Q8N3H0	Chemokine-like protein TAFA-2	TAFA2			
Q5QJ74	Tubulin-specific chaperone cofactor E-like protein	TBCEL			
Q86TM6	E3 ubiquitin-protein ligase synoviolin	SYVN1			
Q6XYQ8	Synaptotagmin-10	SYT10			

Q9P016	Thymocyte nuclear protein 1	THYN1			
B4DZS4	T-complex protein 11 X-linked protein 1	TCP11X1			
Q7L7X3	Serine/threonine-protein kinase TAO1	TAOK1			
Q6PEY2	Tubulin alpha-3E chain	TUBA3E			
Q969V4	Tektin-1	TEKT1			
Q9BSF4	Mitochondrial import inner membrane translocase subunit Tim29	TIMM29			
Q8IUR0	Trafficking protein particle complex subunit 5	TRAPPC5			
Q9NRJ4	Tubby-related protein 4	TULP4			
Q8TAM2	Tetratricopeptide repeat protein 8	TTC8			
A0A087WT02	T cell receptor alpha variable 9-2	TRAV9-2			
Q96PP4	Testis-specific gene 13 protein	TSGA13			
D6RBM5	Putative ubiquitin carboxyl-terminal hydrolase 17-like protein 23	USP17L23			
P07911	Uromodulin	UMOD			
Q15029	116 kDa U5 small nuclear ribonucleoprotein component	EFTUD2			
P10599	Thioredoxin	TXN			
Q9Y4G6	Talin-2	TLN2			
Q8IY95	Transmembrane protein 192	TMEM192			
P37173	TGF-beta receptor type-2	TGFBR2			
Q8IYM9	E3 ubiquitin-protein ligase	TRIM22			
Q5T2D2	Trem-like transcript 2 protein	TREML2			
Q9NZQ9	Tropomodulin-4	TMOD4			
Q9BX59	Tapasin-related protein	TAPBPL			
P0DPR3	T cell receptor delta diversity 1	TRDD1			
Q6PJ69	Tripartite motif-containing protein 65	TRIM65			
Q8N7C3	Probable E3 ubiquitin-protein ligase TRIML2	TRIML2			
Q969Z4	Tumor necrosis factor receptor superfamily member 19L	RELT			
Q9Y606	tRNA pseudouridine synthase A	PUS1			
Q8NDW8	Tetratricopeptide repeat protein 21A	TTC21A			
A8MYJ7	Tetratricopeptide repeat protein 34	TTC34			
A6PVC2	Protein monoglycylase TTLL8	TTLL8			
A0A0B4J26	T cell receptor alpha variable 39	TRAV39			
Q9BY10	Thymic stromal cotransporter homolog	SLC46A2			
Q6PGP7	Tetratricopeptide repeat protein 37	TTC37			
P30536	Translocator protein	TSPO			
Q12792	Twinfilin-1	TWF1			
O94759	Transient receptor potential cation channel subfamily M member 2	TRPM2			
P61086	Ubiquitin-conjugating enzyme E2 K	UBE2K			
Q16881	Thioredoxin reductase 1, cytoplasmic	TXNRD1			
Q9P2D8	Protein unc-79 homolog	UNC79			
O75795	UDP-glucuronosyltransferase	UGT2B17			
O14795	Protein unc-13 homolog B	UNC13B			
P35504	UDP-glucuronosyltransferase 1A5	UGT1A5			
O60701	UDP-glucose 6-dehydrogenase	UGDH			
O00124	UBX domain-containing protein 8	UBXN8			
Q6IPR3	tRNA wybutosine-synthesizing protein 3 homolog	TYW3			
Q96NG5	Zinc finger protein 558	ZNF558			
Q9UJU3	Zinc finger protein 112	ZNF112			
Q969N4	Trace amine-associated receptor 8	TAAR8			

Q9BQ87	F-box-like/WD repeat-containing protein TBL1Y	TBL1Y			
P57105	Synaptojanin-2-binding protein	SYNJ2BP			
Q92609	TBC1 domain family member 5	TBC1D5			
A0A087WVF3	TBC1 domain family member 3D	TBC1D3D			
O15119	T-box transcription factor TBX3	TBX3			
Q495A1	T-cell immunoreceptor with Ig and ITIM domains	TIGIT			
Q3YBM2	Transmembrane protein 176B	TMEM176B			
P31314	T-cell leukemia homeobox protein 1	TLX1			
Q9BSI4	TERF1-interacting nuclear factor 2	TINF2			
P59533	Taste receptor type 2 member 38	TAS2R38			
Q8IUE0	Homeobox protein TGIF2LY	TGIF2LY			
Q9Y5M6	Oculomedin	OCLM			
A0A0A0MT70	T cell receptor beta joining 2-6	TRBJ2-6			
Q5JRA6	Transport and Golgi organization protein 1 homolog	MIA3			
O60704	Protein-tyrosine sulfotransferase 2	TPST2			
Q16890	Tumor protein D53	TPD52L1			
Q9UL33	Trafficking protein particle complex subunit 2-like protein	TRAPPC2L			
Q5JU69	Torsin-2A	TOR2A			
A0JD37	T cell receptor delta variable 3	TRDV3			
Q63HK5	Teashirt homolog 3	TSHZ3			
A0A599	T cell receptor beta variable 5-6	TRBV5-6			
C9J2P7	Ubiquitin carboxyl-terminal hydrolase 17-like protein 15	USP17L15			
P0C7H9	Inactive ubiquitin carboxyl-terminal hydrolase 17-like protein 7	USP17L7			
Q8WVZ1	Palmitoyltransferase ZDHHC19	ZDHHC19			
Q8IYB9	Zinc finger protein 595	ZNF595			
Q9H7X3	Zinc finger protein 696	ZNF696			
A6NN14	Zinc finger protein 729	ZNF729			
Q9Y519	Transmembrane protein 184B	TMEM184B			
Q9Y2I9	TBC1 domain family member 30	TBC1D30			
P0DP42	Transmembrane protein 225B	TMEM225B			
Q9H0A3	Transmembrane protein 191A	TMEM191A			
Q16143	Beta-synuclein	SNCB			
Q16635	Tafazzin	TAZ			
Q93075	Putative deoxyribonuclease TATDN2	TATDN2			
Q9UQP3	Tenascin-N	TNN			
Q9BW92	Threonine--tRNA ligase, mitochondrial	TARS2			
O14817	Tetraspanin-4	TSPAN4			
P53804	E3 ubiquitin-protein ligase TTC3	TTC3			
Q9BXA7	Testis-specific serine/threonine-protein kinase 1	TSSK1B			
A0A1B0GXF2	T cell receptor beta variable 7-2	TRBV7-2			
A0A0A0MS04	Probable non-functional T cell receptor beta variable 6-7	TRBV6-7			
A0A1B0GX31	T cell receptor beta variable 7-6	TRBV7-6			
A0A1B0GX49	T cell receptor beta variable 6-4	TRBV6-4			
Q969E3	Urocortin-3	UCN3			
Q96K76	Ubiquitin carboxyl-terminal hydrolase 47	USP47			
H0YL09	Putative ubiquitin-conjugating enzyme E2Q2-like protein	UBE2Q2L			
O75691	Small subunit processome component 20 homolog	UTP20			
Q9Y5K8	V-type proton ATPase subunit D	ATP6V1D			

P50552	Vasodilator-stimulated phosphoprotein	VASP			
Q9Y2T7	Y-box-binding protein 2	YBX2			
A6NHS1	Putative uncharacterized protein ENSP00000347057				
Q6ZRX8	Putative uncharacterized protein FLJ45999				
A6NJY4	Putative transmembrane protein LOC100289255				
Q9Y5A9	YTH domain-containing family protein 2	YTHDF2			
Q9H4Z2	Zinc finger protein 335	ZNF335			
Q6ZN79	Zinc finger protein 705A	ZNF705A			
Q9Y330	Zinc finger and BTB domain-containing protein 12	ZBTB12			
Q5TFG8	Zinc finger C2HC domain-containing protein 1B	ZC2HC1B			
Q9UDY2	Tight junction protein ZO-2	TJP2			
Q8N567	Zinc finger CCHC domain-containing protein 9	ZCCHC9			
P49910	Zinc finger protein 165	ZNF165			
P35789	Zinc finger protein 93	ZNF93			
Q6P9A3	Zinc finger protein 549	ZNF549			
Q8TBF4	Zinc finger CCHC-type and RNA-binding motif-containing protein 1	ZCRB1			
Q6NZY4	Zinc finger CCHC domain-containing protein 8	ZCCHC8			
Q15973	Zinc finger protein 124	ZNF124			
O75346	Zinc finger protein 253	ZNF253			
Q9HCG1	Zinc finger protein 160	ZNF160			
Q6AZW8	Zinc finger protein 660	ZNF660			
O14628	Zinc finger protein 195	ZNF195			
O14709	Zinc finger protein 197	ZNF197			
O95947	T-box transcription factor TBX6	TBX6			
Q9P2M4	TBC1 domain family member 14	TBC1D14			
Q9BRU2	Transcription elongation factor A protein-like 7	TCEAL7			
Q9ULZ0	TP53-target gene 3 protein	TP53TG3			
O95988	T-cell leukemia/lymphoma protein 1B	TCL1B			
A6NM43	Putative T-complex protein 1 subunit theta-like 1	CCT8L1P			
Q8IYN2	Transcription elongation factor A protein-like 8	TCEAL8			
Q6ZMB5	Transmembrane protein 184A	TMEM184A			
O75529	TAF5-like RNA polymerase II p300/CBP-associated factor-associated factor 65 kDa subunit 5L	TAF5L			
A6NC51	Modulator of macroautophagy TMEM150B	TMEM150B			
Q86WV5	CST complex subunit TEN1	TEN1			
Q9P273	Teneurin-3	TENM3			
Q86UV7	Tripartite motif-containing protein 73	TRIM73			
O15344	E3 ubiquitin-protein ligase Midline-1	MID1			
Q9C040	Tripartite motif-containing protein 2	TRIM2			
Q12931	Heat shock protein 75 kDa, mitochondrial	TRAP1			
Q9UET6	Putative tRNA	FTSJ1			
P41273	Tumor necrosis factor ligand superfamily member 9	TNFSF9			
Q96ET8	Golgi apparatus membrane protein TVP23 homolog C	TVP23C			
A0A087X0M5	T cell receptor beta variable 18	TRBV18			
A0A0B4J23	T cell receptor alpha variable 2	TRAV2			

P13051	Uracil-DNA glycosylase	UNG			
Q969M7	NEDD8-conjugating enzyme UBE2F	UBE2F			
Q96MP5	Zinc finger SWIM domain-containing protein 3	ZSWIM3			
Q9C0D7	Probable ribonuclease ZC3H12C	ZC3H12C			
Q9C0G0	Zinc finger protein 407	ZNF407			
Q9H8G1	Zinc finger protein 430	ZNF430			
O14978	Zinc finger protein 263	ZNF263			
P00749	Urokinase-type plasminogen activator	PLAU			
Q8N8C0	Zinc finger protein 781	ZNF781			
A6NP11	Zinc finger protein 716	ZNF716			
Q7L945	Zinc finger protein 627	ZNF627			
Q13336	Urea transporter 1	SLC14A1			
O15240	Neurosecretory protein VGF	VGF			
O95670	V-type proton ATPase subunit G 2	ATP6V1G2			
Q9NNW5	WD repeat-containing protein 6	WDR6			
P51811	Membrane transport protein XK	XK			
Q8N0Z9	V-set and immunoglobulin domain-containing protein 10	VSIG10			
P63131	Endogenous retrovirus group K member 7 Pro protein	ERVK-7			
Q14191	Werner syndrome ATP-dependent helicase	WRN			
Q8WTP9	X antigen family member 3	XAGE3			
Q9H8V8	Putative uncharacterized protein FLJ13197				
Q8N9G6	Putative UPF0607 protein				
Q6UXQ8	Putative uncharacterized protein UNQ6190/PRO20217	UNQ6190/ PRO20217			
A6NLC8	Putative TAF11-like protein ENSP00000332601				
P0C6A0	GATA-type zinc finger protein 1	ZGLP1			
Q6ZVL8	Putative uncharacterized protein FLJ42384				
P0C7U3	Probable palmitoyltransferase ZDHHC11B	ZDHHC11B			
Q6ZRU5	Putative uncharacterized protein FLJ46089				
O60315	Zinc finger E-box-binding homeobox 2	ZEB2			
P15822	Zinc finger protein 40	HIVEP1			
Q96JP5	E3 ubiquitin-protein ligase ZFP91	ZFP91			
Q6ZMS4	Zinc finger protein 852	ZNF852			
Q5JNZ3	Zinc finger protein 311	ZNF311			
Q9C0D4	Zinc finger protein 518B	ZNF518B			
Q8WW36	Zinc finger CCHC domain-containing protein 13	ZCCHC13			
Q86UZ6	Zinc finger and BTB domain-containing protein 46	ZBTB46			
Q9H6B1	Zinc finger protein 385D	ZNF385D			
Q15326	Zinc finger MYND domain-containing protein 11	ZMYND11			
Q8N2G6	Zinc finger CCHC domain-containing protein 24	ZCCHC24			
A0A1B0GTU1	Zinc finger CCCH domain-containing protein 11B	ZC3H11B			
Q0IIN9	Putative uncharacterized protein ZNF252P-AS1	ZNF252P-AS1			
P17023	Zinc finger protein 19	ZNF19			
O75312	Zinc finger protein ZPR1	ZPR1			
Q66K41	Zinc finger protein 385C	ZNF385C			
P60852	Zona pellucida sperm-binding protein 1	ZP1			
Q6P9G9	Zinc finger protein 449	ZNF449			
Q9C0B9	Zinc finger CCHC domain-containing protein 2	ZCCHC2			

Q53FD0	Zinc finger C2HC domain-containing protein 1C	ZC2HC1C			
P52747	Zinc finger protein 143	ZNF143			
Q6PF04	Zinc finger protein 613	ZNF613			
Q9UIJ5	Palmitoyltransferase ZDHHC2	ZDHHC2			
Q5SVZ6	Zinc finger MYM-type protein 1	ZMYM1			
Q7Z340	Zinc finger protein 551	ZNF551			
Q8NBB4	Zinc finger and SCAN domain-containing protein 1	ZSCAN1			
Q8N8L2	Zinc finger protein 491	ZNF491			
Q5TEC3	Zinc finger protein 697	ZNF697			
Q9P0T4	Zinc finger protein 581	ZNF581			
Q86W42	THO complex subunit 6 homolog	THOC6			
A8MWL7	Transmembrane protein 14DP	TMEM14DP			
Q7Z6K1	THAP domain-containing protein 5	THAP5			
Q8IWB6	Inactive serine/threonine-protein kinase TEX14	TEX14			
H3BV60	Transforming growth factor-beta receptor type 3-like protein	TGFBR3L			
Q53FP2	Transmembrane protein 35A	TMEM35A			
Q9NWC5	Transmembrane protein 45A	TMEM45A			
Q7L0X0	TLR4 interactor with leucine rich repeats	TRIL			
Q7Z4K8	Tripartite motif-containing protein 46	TRIM46			
P0DSE2	M1-specific T cell receptor beta chain	TRB			
Q9H0U9	Testis-specific Y-encoded-like protein 1	TSPYL1			
Q7Z7G8	Vacuolar protein sorting-associated protein 13B	VPS13B			
Q0WX57	Ubiquitin carboxyl-terminal hydrolase 17-like protein 24	USP17L24			
Q68DE3	Basic helix-loop-helix domain-containing protein USF3	USF3			
Q3KQV9	UDP-N-acetylhexosamine pyrophosphorylase-like protein 1	UAP1L1			
Q6QN14	Ubiquitin carboxyl-terminal hydrolase 17-like protein 6	USP17L6P			
Q14135	Transcription cofactor vestigial-like protein 4	VGLL4			
Q70EK8	Inactive ubiquitin carboxyl-terminal hydrolase 53	USP53			
B4DXR9	Zinc finger protein 732	ZNF732			
Q6ZT12	E3 ubiquitin-protein ligase UBR3	UBR3			
Q9BRX9	WD repeat domain-containing protein 83	WDR83			
Q9H1F0	WAP four-disulfide core domain protein 10A	WFDC10A			
Q96N03	V-set and transmembrane domain-containing protein 2-like protein	VSTM2L			
Q5TIE3	von Willebrand factor A domain-containing protein 5B1	VWA5B1			
O43592	Exportin-T	XPOT			
P12956	X-ray repair cross-complementing protein 6	XRCC6			
O75695	Protein XRP2	RP2			
Q9UBH6	Xenotropic and polytropic retrovirus receptor 1	XPR1			
Q9P2L0	WD repeat-containing protein 35	WDR35			
Q86VZ2	WD repeat-containing protein 5B	WDR5B			
Q6GPH4	XIAP-associated factor 1	XAF1			
Q8IY57	YY1-associated factor 2	YAF2			
A8MUA0	Putative UPF0607 protein ENSP00000381514				
Q8N9L1	Zinc finger protein ZIC 4	ZIC4			
P62699	Protein yippee-like 5	YPEL5			

Q6UXU0	Putative uncharacterized protein UNQ9165/PRO28630	UNQ9165/ PRO28630			
Q8IYH5	ZZ-type zinc finger-containing protein 3	ZZZ3			
A8MUN3	Putative uncharacterized protein ENSP00000381830				
Q5W150	Putative uncharacterized protein MGC163334				
Q5T1R4	Transcription factor HIVEP3	HIVEP3			
Q9P243	Zinc finger protein ZFAT	ZFAT			
Q5T742	Uncharacterized protein ZNF22-AS1	ZNF22-AS1			
O75123	Zinc finger protein 623	ZNF623			
Q5HY98	Zinc finger protein 766	ZNF766			
Q8N966	Palmitoyltransferase ZDHHC22	ZDHHC22			
Q6PML9	Zinc transporter 9	SLC30A9			
Q96N38	Zinc finger protein 714	ZNF714			
Q9BU19	Zinc finger protein 692	ZNF692			
Q969W1	Palmitoyltransferase ZDHHC16	ZDHHC16			
Q6NUN9	Zinc finger protein 746	ZNF746			
P62380	TATA box-binding protein-like 1	TBPL1			
P26640	Valine--tRNA ligase	VARS1			
P48775	Tryptophan 2,3-dioxygenase	TDO2			
Q9Y3D7	Mitochondrial import inner membrane translocase subunit TIM16	PAM16			
Q8NBV8	Synaptotagmin-8	SYT8			
P59539	Taste receptor type 2 member 45	TAS2R45			
Q86VP1	Tax1-binding protein 1	TAX1BP1			
Q9H2G4	Testis-specific Y-encoded-like protein 2	TSPYL2			
P01737	T cell receptor alpha variable 8-4	TRAV8-4			
Q5SRH9	Tetratricopeptide repeat protein 39A	TTC39A			
Q96FV3	Tetraspanin-17	TSPAN17			
Q5I0X7	Tetratricopeptide repeat protein 32	TTC32			
A0A0B4J27	T cell receptor alpha variable 34	TRAV34			
Q9HCJ0	Trinucleotide repeat-containing gene 6C protein	TNRC6C			
P63121	Endogenous retrovirus group K member 113 Pro protein	HERVK_113			
Q9H0E7	Ubiquitin carboxyl-terminal hydrolase 44	USP44			
Q15904	V-type proton ATPase subunit S1	ATP6AP1			
P21283	V-type proton ATPase subunit C 1	ATP6V1C1			
Q8N976	Putative uncharacterized protein FLJ38264				
A8MXK9	Uncharacterized protein ENSP00000382033				
Q9UJL9	Zinc finger protein 69 homolog B	ZFP69B			
Q15911	Zinc finger homeobox protein 3	ZFHX3			
Q9NTW7	Zinc finger protein 64	ZFP64			
Q86UP3	Zinc finger homeobox protein 4	ZFHX4			
Q96PM9	Zinc finger protein 385A	ZNF385A			
P17041	Zinc finger protein 32	ZNF32			
Q9H0M4	Zinc finger CW-type PWWP domain protein 1	ZCWPW1			
Q8IZ26	Zinc finger protein 34	ZNF34			
Q8N587	Zinc finger protein 561	ZNF561			
O15060	Zinc finger and BTB domain-containing protein 39	ZBTB39			
Q7RTV3	Zinc finger protein 367	ZNF367			
Q8IUH4	Palmitoyltransferase ZDHHC13	ZDHHC13			
Q96E35	Zinc finger MYND domain-containing protein 19	ZMYND19			
Q96H40	Zinc finger protein 486	ZNF486			
P52738	Zinc finger protein 140	ZNF140			
Q8IZC7	Zinc finger protein 101	ZNF101			
Q86YE8	Zinc finger protein 573	ZNF573			

Q14593	Zinc finger protein 273	ZNF273			
Q96JC4	Zinc finger protein 479	ZNF479			
A8K8V0	Zinc finger protein 785	ZNF785			
Q9BY31	Zinc finger protein 717	ZNF717			
Q6NX49	Zinc finger protein 544	ZNF544			
Q9NXF8	Palmitoyltransferase ZDHHC7	ZDHHC7			
P52739	Zinc finger protein 131	ZNF131			
Q9GZM7	Tubulointerstitial nephritis antigen-like	TINAGL1			
Q9Y6G1	Transmembrane protein 14A	TMEM14A			
Q6ZP80	Transmembrane protein 182	TMEM182			
Q15025	TNFAIP3-interacting protein 1	TNIP1			
Q96LD4	E3 ubiquitin-protein ligase	TRIM47			
Q8NDY8	Transmembrane protein 52	TMEM52			
Q8N6N2	Tetratricopeptide repeat protein 9B	TTC9B			
A0A0B4J26	T cell receptor alpha variable 38-1	TRAV38-1			
Q5TAA0	Tetratricopeptide repeat protein 22	TTC22			
P04435	T cell receptor beta variable 7-9	TRBV7-9			
P47985	Cytochrome b-c1 complex subunit Rieske, mitochondrial	UQCRFS1			
Q7RTZ2	Ubiquitin carboxyl-terminal hydrolase 17-like protein 1	USP17L1			
Q15696	U2 small nuclear ribonucleoprotein auxiliary factor 35 kDa subunit-related protein 2	ZRSR2			
Q8WVN8	Ubiquitin-conjugating enzyme E2 Q2	UBE2Q2			
P52743	Putative zinc finger protein 137	ZNF137P			
O95379	Tumor necrosis factor alpha-induced protein 8	TNFAIP8			
P34981	Thyrotropin-releasing hormone receptor	TRHR			
Q9HCM9	E3 ubiquitin-protein ligase	TRIM39			
Q9BYV6	Tripartite motif-containing protein 55	TRIM55			
P36941	Tumor necrosis factor receptor superfamily member 3	LTBR			
Q9NXH8	Torsin-4A	TOR4A			
Q8TDI8	Transmembrane channel-like protein 1	TMC1			
P19237	Troponin I, slow skeletal muscle	TNNI1			
Q309B1	Tripartite motif-containing protein 16-like protein	TRIM16L			
O14657	Torsin-1B	TOR1B			
Q96MH6	Transmembrane protein 68	TMEM68			
Q9BUZ4	TNF receptor-associated factor 4	TRAF4			
Q69YG0	Transmembrane protein 42	TMEM42			
Q9UPV9	Trafficking kinesin-binding protein 1	TRAK1			
Q12933	TNF receptor-associated factor 2	TRAF2			
O14545	TRAF-type zinc finger domain-containing protein 1	TRAFD1			
A0A5A2	T cell receptor beta variable 5-8	TRBV5-8			
Q8N5M4	Tetratricopeptide repeat protein 9C	TTC9C			
P50607	Tubby protein homolog	TUB			
Q92890	Ubiquitin recognition factor in ER-associated degradation protein 1	UFD1			
P61081	NEDD8-conjugating enzyme Ubc12	UBE2M			
P57075	Ubiquitin-associated and SH3 domain-containing protein A	UBASH3A			
P06133	UDP-glucuronosyltransferase 2B4	UGT2B4			
D6R9N7	Ubiquitin carboxyl-terminal hydrolase 17-like protein 18	USP17L18			
Q8N8G2	Transcription cofactor vestigial-like protein 2	VGLL2			

Q9H582	Zinc finger protein 644	ZNF644			
Q7Z7K2	Zinc finger protein 467	ZNF467			
Q96MW7	Tigger transposable element-derived protein 1	TIGD1			
P55072	Transitional endoplasmic reticulum ATPase	VCP			
Q6UXF1	Transmembrane protein 108	TMEM108			
Q04206	Transcription factor p65	RELA			
Q6ZMP0	Thrombospondin type-1 domain-containing protein 4	THSD4			
A0A1B0GUV7	Testis-expressed protein 48	TEX48			
Q53QW1	Testis-expressed protein 44	TEX44			
A0A1W2PP97	Thrombospondin type-1 domain-containing protein 8	THSD8			
P24557	Thromboxane-A synthase	TBXAS1			
Q9P2Z0	THAP domain-containing protein 10	THAP10			
Q9Y584	Mitochondrial import inner membrane translocase subunit Tim22	TIMM22			
H3BTG2	Testis-expressed protein 46	TEX46			
Q6UXP3	Transmembrane protein 14EP	TMEM14EP			
Q6ZYL4	General transcription factor IIH subunit 5	GTF2H5			
A0A075B700	T cell receptor alpha joining 31	TRAJ31			
P0C7T8	Transmembrane protein 253	TMEM253			
Q0VDI3	Transmembrane protein 267	TMEM267			
P61812	Transforming growth factor beta-2 proprotein	TGFB2			
Q86XT9	Insulin-like growth factor-binding protein 3 receptor	TMEM219			
P60174	Triosephosphate isomerase	TPI1			
Q96GJ1	tRNA	TRMT2B			
Q9BYJ4	Tripartite motif-containing protein 34	TRIM34			
A0A0C4DH27	T cell receptor gamma variable 8	TRGV8			
Q15785	Mitochondrial import receptor subunit TOM34	TOMM34			
Q86UF1	Tetraspanin-33	TSPAN33			
Q14166	Tubulin--tyrosine ligase-like protein 12	TTLL12			
A0A1B0GX78	T cell receptor beta variable 12-5	TRBV12-5			
Q5VY80	UL16-binding protein 6	RAET1L			
Q9H1D0	Transient receptor potential cation channel subfamily V member 6	TRPV6			
P02585	Troponin C, skeletal muscle	TNNC2			
Q9C0B0	RING finger protein unkempt homolog	UNK			
Q86T82	Ubiquitin carboxyl-terminal hydrolase 37	USP37			
O15391	Transcription factor YY2	YY2			
C9JLJ4	Ubiquitin carboxyl-terminal hydrolase 17-like protein 13	USP17L13			
Q9Y6I4	Ubiquitin carboxyl-terminal hydrolase 3	USP3			
Q86V25	Tubulinyl-Tyr carboxypeptidase 2	VASH2			
Q96KV7	WD repeat-containing protein 90	WDR90			
A6NE52	WD repeat-containing protein 97	WDR97			
Q8WWM1	X antigen family member 5	XAGE5			
Q5T9L3	Protein wntless homolog	WLS			
Q9UF83	Uncharacterized protein DKFZp434B061				
Q96J92	Serine/threonine-protein kinase WNK4	WNK4			
A8MPS7	Carbohydrate deacetylase	YDJC			

Q6ZT77	Putative zinc finger protein 826	ZNF826P			
P59817	Zinc finger protein 280A	ZNF280A			
P0CG31	Putative zinc finger protein 286B	ZNF286B			
P17030	Zinc finger protein 25	ZNF25			
Q9P1Z0	Zinc finger and BTB domain-containing protein 4	ZBTB4			
Q5TC79	Zinc finger and BTB domain-containing protein 37	ZBTB37			
Q05481	Zinc finger protein 91	ZNF91			
P17027	Zinc finger protein 23	ZNF23			
Q9GZX5	Zinc finger protein 350	ZNF350			
Q9H5U6	rRNA N6-adenosine-methyltransferase ZCCHC4	ZCCHC4			
Q8IWR0	Zinc finger CCCH domain-containing protein 7A	ZC3H7A			
A1YPR0	Zinc finger and BTB domain-containing protein 7C	ZBTB7C			
Q96GC6	Neurotrophin receptor-interacting factor homolog	ZNF274			
O95201	Zinc finger protein 205	ZNF205			
O75362	Zinc finger protein 217	ZNF217			
Q96NC0	Zinc finger matrin-type protein 2	ZMAT2			
P0CB33	Putative zinc finger protein 735	ZNF735			
O14776	Transcription elongation regulator 1	TCERG1			
Q53R12	Transmembrane 4 L6 family member 20	TM4SF20			
Q9HBM6	Transcription initiation factor TFIID subunit 9B	TAF9B			
Q9UL49	Transcription factor-like 5 protein	TCFL5			
O75333	T-box transcription factor TBX10	TBX10			
Q8IXB3	Trafficking regulator of GLUT4 1	TRARG1			
Q8IZW8	Tensin-4	TNS4			
Q96QS1	Tetraspanin-32	TSPAN32			
P49746	Thrombospondin-3	THBS3			
Q9BZA0	Putative transcript Y 10 protein	TTTY10			
Q86WB7	Protein unc-93 homolog A	UNC93A			
Q9BXU7	Ubiquitin carboxyl-terminal hydrolase 26	USP26			
A6NN06	Putative UPF0633 protein MGC21881				
Q9BZF9	Uveal autoantigen with coiled-coil domains and ankyrin repeats	UACA			
P55916	Mitochondrial uncoupling protein 3	UCP3			
Q8TAS1	Serine/threonine-protein kinase Kist	UHMK1			
A6NCW7	Inactive ubiquitin carboxyl-terminal hydrolase 17-like protein 4	USP17L4			
Q9NY84	Vascular non-inflammatory molecule 3	VNN3			
Q9H598	Vesicular inhibitory amino acid transporter	SLC32A1			
Q8N9P0	Putative uncharacterized protein FLJ36797				
Q9HAA7	Putative uncharacterized protein FLJ11871				
O60481	Zinc finger protein ZIC 3	ZIC3			
Q6ZS49	Putative uncharacterized protein FLJ45831				
Q6FIF0	AN1-type zinc finger protein 6	ZFAND6			
P0CJ79	Zinc finger protein 888	ZNF888			
Q96DA0	Zymogen granule protein 16 homolog B	ZG16B			
Q96GE5	Zinc finger protein 799	ZNF799			
Q9NSJ1	Putative zinc finger protein 355P	ZNF355P			
Q8WYQ9	Zinc finger CCHC domain-containing protein 14	ZCCHC14			

Q9UC07	Zinc finger protein 69	ZNF69			
O75132	Zinc finger BED domain-containing protein 4	ZBED4			
Q9UJN7	Zinc finger protein 391	ZNF391			
Q7Z398	Zinc finger protein 550	ZNF550			
Q8IVC4	Zinc finger protein 584	ZNF584			
Q96SR6	Zinc finger protein 382	ZNF382			
Q9Y473	Zinc finger protein 175	ZNF175			
Q96N22	Zinc finger protein 681	ZNF681			
Q8TF50	Zinc finger protein 526	ZNF526			
O43296	Zinc finger protein 264	ZNF264			
Q9Y397	Palmitoyltransferase ZDHHC9	ZDHHC9			
Q6ZMY9	Zinc finger protein 517	ZNF517			
Q5FWF6	Zinc finger protein 789	ZNF789			
Q9BVA1	Tubulin beta-2B chain	TUBB2B			
P37840	Alpha-synuclein	SNCA			
Q96HP8	Transmembrane protein 176A	TMEM176A			
Q12893	Transmembrane protein 115	TMEM115			
A0A0J9YX0	T cell receptor beta joining 1-2	TRBJ1-2			
P24752	Acetyl-CoA acetyltransferase, mitochondrial	ACAT1			
Q9P0S9	Transmembrane protein 14C	TMEM14C			
Q6ZRR5	TLC domain-containing protein 5	TLCD5			
Q2M3C6	Transmembrane protein 266	TMEM266			
Q03167	Transforming growth factor beta receptor type 3	TGFBR3			
O43548	Protein-glutamine gamma-glutamyltransferase 5	TGM5			
Q6NUS6	Tectonic-3	TCTN3			
Q9NX00	Transmembrane protein 160	TMEM160			
Q9BQ50	Three prime repair exonuclease 2	TREX2			
C9JQI7	Transmembrane protein 232	TMEM232			
Q96RU8	Tribbles homolog 1	TRIB1			
Q9UJA5	tRNA	TRMT6			
Q6UXU6	Transmembrane protein 92	TMEM92			
Q8NHX9	Two pore calcium channel protein 2	TPCN2			
P01375	Tumor necrosis factor	TNF			
P11387	DNA topoisomerase 1	TOP1			
P45378	Troponin T, fast skeletal muscle	TNNT3			
O14668	Transmembrane gamma-carboxyglutamic acid protein 1	PRRG1			
P19438	Tumor necrosis factor receptor superfamily member 1A	TNFRSF1A			
Q96DC7	Transmembrane and coiled-coil domain-containing protein 6	TMCO6			
Q9Y5L0	Transportin-3	TNPO3			
Q92973	Transportin-1	TNPO1			
Q7L2K0	Tubulin epsilon and delta complex protein 2	TEDC2			
Q8N4P2	Tetratricopeptide repeat protein 30B	TTC30B			
P98066	Tumor necrosis factor-inducible gene 6 protein	TNFAIP6			
Q8TE23	Taste receptor type 1 member 2	TAS1R2			
A0A075B6N3	T cell receptor beta variable 24-1	TRBV24-1			
Q2TAM9	Tumor suppressor candidate gene 1 protein	TUSC1			
A0A0A6YYG3	T cell receptor beta variable 6-8	TRBV6-8			
Q9BZ98	Putative transcript Y 12 protein	TTTY12			
P22310	UDP-glucuronosyltransferase 1A4	UGT1A4			
P11441	Ubiquitin-like protein 4A	UBL4A			
P68543	UBX domain-containing protein 2A	UBXN2A			
Q9H3U1	Protein unc-45 homolog A	UNC45A			
Q5XG85	Putative UPF0633 protein LOC554249				
Q9NWZ5	Uridine-cytidine kinase-like 1	UCKL1			

Q7Z2F6	Putative protein ZNF720	ZNF720			
Q9NWS9	Zinc finger protein 446	ZNF446			
P55851	Mitochondrial uncoupling protein 2	UCP2			
P15498	Proto-oncogene vav	VAV1			
Q9UKW4	Guanine nucleotide exchange factor VAV3	VAV3			
Q96NZ8	WAP, Kazal, immunoglobulin, Kunitz and NTR domain-containing protein 1	WFIKKN1			
Q7Z5U6	WD repeat-containing protein 53	WDR53			
P17861	X-box-binding protein 1	XBP1			
Q5BKY6	Putative uncharacterized protein DKFZp434K191				
Q8N9L7	Putative uncharacterized protein FLJ36925				
P61236	Protein yippee-like 3	YPEL3			
Q6ZRG5	Putative uncharacterized protein FLJ43944				
Q8N3U1	Putative uncharacterized protein LOC400692				
Q8WTZ3	Zinc finger protein ENSP00000375192				
Q96MU7	YTH domain-containing protein 1	YTHDC1			
Q9BTK2	Putative uncharacterized protein LOC642776				
Q8NHY6	Zinc finger protein 28 homolog	ZFP28			
Q8WV99	AN1-type zinc finger protein 2B	ZFAND2B			
Q8N141	Zinc finger protein 82 homolog	ZFP82			
B7Z6K7	Zinc finger protein 814	ZNF814			
A8K554	Putative protein ZNF815	ZNF815P			
Q5JPB2	Zinc finger protein 831	ZNF831			
Q96NB3	Zinc finger protein 830	ZNF830			
B2RXF5	Zinc finger and BTB domain-containing protein 42	ZBTB42			
Q96N77	Zinc finger protein 641	ZNF641			
A6NK75	Zinc finger protein 98	ZNF98			
Q9Y5V0	Zinc finger protein 706	ZNF706			
Q8NAP8	Zinc finger and BTB domain-containing protein 8B	ZBTB8B			
Q8N0Y2	Zinc finger protein 444	ZNF444			
Q8NEW0	Zinc transporter 7	SLC30A7			
P21754	Zona pellucida sperm-binding protein 3	ZP3			
Q9UK11	Zinc finger protein 223	ZNF223			
Q9UKE5	TRAF2 and NCK-interacting protein kinase	TNIK			
Q6ZMU5	Tripartite motif-containing protein 72	TRIM72			
Q4KMQ1	Taperin	TPRN			
Q8N6Q1	Transmembrane and coiled-coil domain-containing protein 5A	TMCO5A			
Q6PI78	Transmembrane protein 65	TMEM65			
Q6IBS0	Twinfilin-2	TWF2			
P33981	Dual specificity protein kinase TTK	TTK			
Q9NYZ1	Golgi apparatus membrane protein TVP23 homolog B	TVP23B			
Q8NET8	Transient receptor potential cation channel subfamily V member 3	TRPV3			
Q9UJT2	Testis-specific serine kinase substrate	TSKS			
Q8NEE8	Tetratricopeptide repeat protein 16	TTC16			
P49459	Ubiquitin-conjugating enzyme E2 A	UBE2A			
P32241	Vasoactive intestinal polypeptide receptor 1	VIPR1			
Q9H0D2	Zinc finger protein 541	ZNF541			
O75820	Zinc finger protein 189	ZNF189			

Q6IV72	Zinc finger protein 425	ZNF425			
Q8TB69	Zinc finger protein 519	ZNF519			
P61421	V-type proton ATPase subunit d 1	ATP6V0D1			
P49767	Vascular endothelial growth factor C	VEGFC			
P27449	V-type proton ATPase 16 kDa proteolipid subunit	ATP6V0C			
Q8TF74	WAS/WASL-interacting protein family member 2	WIPF2			
Q8IYB0	Putative uncharacterized protein MGC39545				
Q9BW85	Splicing factor YJU2	YJU2			
Q6ZSR9	Uncharacterized protein FLJ45252				
Q8IZM8	Zinc finger protein 654	ZNF654			
Q9ULD5	Zinc finger protein 777	ZNF777			
Q12901	Zinc finger protein 155	ZNF155			
P52742	Zinc finger protein 135	ZNF135			
Q8NDP4	Zinc finger protein 439	ZNF439			
Q86UQ0	Zinc finger protein 589	ZNF589			
Q9UK13	Zinc finger protein 221	ZNF221			
Q8NEK5	Zinc finger protein 548	ZNF548			
Q9NVV0	Trimeric intracellular cation channel type B	TMEM38B			
Q9NUR3	Transmembrane protein 74B	TMEM74B			
P0DI81	Trafficking protein particle complex subunit 2	TRAPPC2			
Q15388	Mitochondrial import receptor subunit TOM20 homolog	TOMM20			
A6NNM8	Tubulin polyglutamylase TTLL13P	TTLL13P			
Q6ZT98	Tubulin polyglutamylase TTLL7	TTLL7			
Q8NG11	Tetraspanin-14	TSPAN14			
P63279	SUMO-conjugating enzyme UBC9	UBE2I			
P10074	Telomere zinc finger-associated protein	ZBTB48			
P35125	Ubiquitin carboxyl-terminal hydrolase 6	USP6			
P62837	Ubiquitin-conjugating enzyme E2 D2	UBE2D2			
Q86TS7	Putative UPF0730 protein encoded by LINC00643	LINC00643			
Q9UHD9	Ubiquilin-2	UBQLN2			
Q9H8Y1	Vertnin	VRTN			
Q8IW00	V-set and transmembrane domain-containing protein 4	VSTM4			
Q9NZR4	Visual system homeobox 1	VSX1			
Q12768	WASH complex subunit 5	WASHC5			
Q8IV35	WD repeat-containing protein 49	WDR49			
Q93098	Protein Wnt-8b	WNT8B			
Q9ULM3	YEATS domain-containing protein 2	YEATS2			
Q9H379	Putative uncharacterized protein PRO3102	PRO3102			
A6NC05	Glutaredoxin-like protein C5orf63	C5orf63			
Q49AG3	Zinc finger BED domain-containing protein 5	ZBED5			
Q8N8H1	Putative protein ZNF321	ZNF321P			
P17020	Zinc finger protein 16	ZNF16			
P0CJ78	Zinc finger protein 865	ZNF865			
Q6AW86	Zinc finger protein 324B	ZNF324B			
Q6P3V2	Zinc finger protein 585A	ZNF585A			
Q6S9Z5	Zinc finger protein 474	ZNF474			
Q8N883	Zinc finger protein 614	ZNF614			
Q96BV0	Zinc finger protein 775	ZNF775			
Q86V71	Zinc finger protein 429	ZNF429			
Q15649	Zinc finger HIT domain-containing protein 3	ZNHIT3			
Q70EL2	Ubiquitin carboxyl-terminal hydrolase 45	USP45			
Q9UIE0	Zinc finger protein 230	ZNF230			
Q6ZSS3	Zinc finger protein 621	ZNF621			

Q8IW36	Zinc finger protein 695	ZNF695			
Q8IZN3	Palmitoyltransferase ZDHHC14	ZDHHC14			
Q9NV72	Zinc finger protein 701	ZNF701			
Q2M3W8	Zinc finger protein 181	ZNF181			
Q9NYG2	Palmitoyltransferase ZDHHC3	ZDHHC3			
Q14966	Zinc finger protein 638	ZNF638			
Q32M78	Zinc finger protein 699	ZNF699			
O14771	Zinc finger protein 213	ZNF213			
Q86T29	Zinc finger protein 605	ZNF605			
Q9BQB6	Vitamin K epoxide reductase complex subunit 1	VKORC1			
O14607	Histone demethylase UTY	UTY			
Q8TF39	Zinc finger protein 483	ZNF483			
Q9BR84	Zinc finger protein 559	ZNF559			
Q96BR6	Zinc finger protein 669	ZNF669			
Q9UDV6	Zinc finger protein 212	ZNF212			
Q14587	Zinc finger protein 268	ZNF268			
Q70EK9	Ubiquitin carboxyl-terminal hydrolase 51	USP51			
Q96QD9	UAP56-interacting factor	FYTTD1			
Q93008	Probable ubiquitin carboxyl-terminal hydrolase FAF-X	USP9X			
Q5GH76	XK-related protein 4	XKR4			
Q93097	Protein Wnt-2b	WNT2B			
Q9Y2W2	WW domain-binding protein 11	WBP11			
Q9ULE0	Protein WWC3	WWC3			
P46937	Transcriptional coactivator YAP1	YAP1			
Q9GZM5	Protein YIPF3	YIPF3			
Q969M3	Protein YIPF5	YIPF5			
A8MT66	Putative uncharacterized protein ENSP00000383407				
Q96MM3	Zinc finger protein 42 homolog	ZFP42			
B4DU55	Zinc finger protein 879	ZNF879			
Q9HCX3	Zinc finger protein 304	ZNF304			
A8K0R7	Zinc finger protein 839	ZNF839			
O43829	Zinc finger and BTB domain-containing protein 14	ZBTB14			
Q96PM5	RING finger and CHY zinc finger domain-containing protein 1	RCHY1			
P08048	Zinc finger Y-chromosomal protein	ZFY			
Q9BUG6	Zinc finger and SCAN domain-containing protein 5A	ZSCAN5A			
Q569K4	Zinc finger protein 385B	ZNF385B			
Q9HCK1	DBF4-type zinc finger-containing protein 2	ZDBF2			
A8MWA4	Putative zinc finger protein 705E	ZNF705E			
Q86Y25	Zinc finger protein 354C	ZNF354C			
Q504Y3	Zinc finger CW-type PWWP domain protein 2	ZCWPW2			
Q8N184	Zinc finger protein 567	ZNF567			
Q8NF99	Zinc finger protein 397	ZNF397			
P17036	Zinc finger protein 3	ZNF3			
Q96JG9	Zinc finger protein 469	ZNF469			
Q9C0F3	Zinc finger protein 436	ZNF436			
Q63HK3	Zinc finger protein with KRAB and SCAN domains 2	ZKSCAN2			
P58317	Zinc finger protein 121	ZNF121			
Q96NL3	Zinc finger protein 599	ZNF599			
Q7L2R6	Zinc finger protein 765	ZNF765			
Q7Z7L9	Zinc finger and SCAN domain-containing protein 2	ZSCAN2			
Q08AN1	Zinc finger protein 616	ZNF616			
Q99727	Metalloproteinase inhibitor 4	TIMP4			
Q17R31	Putative deoxyribonuclease TATDN3	TATDN3			
O00206	Toll-like receptor 4	TLR4			
P59551	Taste receptor type 2 member 60	TAS2R60			
Q9BYE2	Transmembrane protease serine 13	TMPRSS13			

Q8TEL6	Short transient receptor potential channel 4-associated protein	TRPC4AP			
Q8N394	Protein O-mannosyl-transferase TMTC2	TMTC2			
Q6UXN7	TOMM20-like protein 1	TOMM20L			
P51580	Thiopurine S-methyltransferase	TPMT			
Q9H2K2	Poly	TNKS2			
Q5T0D9	Tumor protein p63-regulated gene 1-like protein	TPRG1L			
Q9BZD6	Transmembrane gamma-carboxyglutamic acid protein 4	PRRG4			
Q9H2L4	Transmembrane protein 60	TMEM60			
Q8N0U2	Transmembrane protein 61	TMEM61			
O00463	TNF receptor-associated factor 5	TRAF5			
O75069	Transmembrane and coiled-coil domains protein 2	TMCC2			
Q6YI46	Transmembrane protein 64	TMEM64			
P0DPE3	Transmembrane and death domain protein 1	TMDD1			
Q6PKC3	Thioredoxin domain-containing protein 11	TXNDC11			
Q9NUQ3	Gamma-taxilin	TXLNG			
Q8NBP0	Tetratricopeptide repeat protein 13	TTC13			
P59090	Putative uncharacterized protein TSPEAR-AS2	TSPEAR-AS2			
P01858	Phagocytosis-stimulating peptide				
A0A075B6N1	T cell receptor beta variable 19	TRBV19			
O43657	Tetraspanin-6	TSPAN6			
Q92574	Hamartin	TSC1			
O95922	Probable tubulin polyglutamylase TTLL1	TTLL1			
Q5DID0	Uromodulin-like 1	UMODL1			
O95231	Homeobox protein VENTX	VENTX			
Q7Z5H4	Vomeronasal type-1 receptor 5	VN1R5			
O60504	Vinexin	SORBS3			
Q05940	Synaptic vesicular amine transporter	SLC18A2			
O60763	General vesicular transport factor p115	USO1			
O15342	V-type proton ATPase subunit e 1	ATP6V0E1			
Q9BRU9	rRNA-processing protein UTP23 homolog	UTP23			
P10265	Endogenous retrovirus group K member 10 Pro protein	ERVK-10			
Q8IUB3	Protein WFDC10B	WFDC10B			
Q9HC57	WAP four-disulfide core domain protein 1	WFDC1			
Q8NEX5	Protein WFDC9	WFDC9			
Q9UID3	Vacuolar protein sorting-associated protein 51 homolog	VPS51			
P55060	Exportin-2	CSE1L			
Q9Y334	von Willebrand factor A domain-containing protein 7	VWA7			
Q64LD2	WD repeat-containing protein 25	WDR25			
C4AMC7	Putative WAS protein family homolog 3	WASH3P			
Q96S55	ATPase WRNIP1	WRNIP1			
Q7L0L9	Transmembrane protein LOC653160				
Q9BWQ6	Protein YIPF2	YIPF2			
Q6ZRM9	Putative uncharacterized protein FLJ46235				
Q6ZRF7	Putative zinc finger protein 818	ZNF818P			
Q96H86	Zinc finger protein 764	ZNF764			
Q96ME7	Zinc finger protein 512	ZNF512			
Q9H6R6	Palmitoyltransferase ZDHHC6	ZDHHC6			
Q8TC21	Zinc finger protein 596	ZNF596			

Q9NUM4	Transmembrane protein 106B	TMEM106B			
P35590	Tyrosine-protein kinase receptor Tie-1	TIE1			
Q6P1K8	General transcription factor IIH subunit 2-like protein	GTF2H2C;			
Q8N9M5	Transmembrane protein 102	TMEM102			
Q68D42	Transmembrane protein 215	TMEM215			
Q08188	Protein-glutamine gamma-glutamyltransferase E	TGM3			
Q96K49	Transmembrane protein 87B	TMEM87B			
P0DSE1	M1-specific T cell receptor alpha chain	TRA			
Q32P41	tRNA	TRMT5			
Q969Q1	E3 ubiquitin-protein ligase	TRIM63			
Q8N9V2	Probable E3 ubiquitin-protein ligase TRIML1	TRIML1			
P13805	Troponin T, slow skeletal muscle	TNNT1			
O94876	Transmembrane and coiled-coil domains protein 1	TMCC1			
Q8N609	Translocating chain-associated membrane protein 1-like 1	TRAM1L1			
Q9BWF2	E3 ubiquitin-protein ligase TRAIP	TRAIP			
Q9UPX0	Protein turtle homolog B	IGSF9B			
A0A0B4J27	T cell receptor alpha variable 25	TRAV25			
Q5TAX3	Terminal uridylyltransferase 4	TUT4			
O15205	Ubiquitin D	UBD			
A0A0B4J2E	T cell receptor beta variable 12-4	TRBV12-4			
Q9BZL1	Ubiquitin-like protein 5	UBL5			
P0C7P4	Putative cytochrome b-c1 complex subunit Rieske-like protein 1	UQCRFS1P 1			
P0C7I0	Inactive ubiquitin carboxyl-terminal hydrolase 17-like protein 8	USP17L8			
Q8WU68	Splicing factor U2AF 26 kDa subunit	U2AF1L4			
Q9Y6N9	Harmonin	USH1C			
Q9HBM0	Vezatin	VEZT			
Q6ZS92	Putative uncharacterized protein FLJ45721				
Q6ZRP5	Putative uncharacterized protein FLJ46204				
A8MUI8	Putative UPF0607 protein ENSP00000383783				
Q6ZVH6	Putative uncharacterized protein FLJ42569				
Q9BSR8	Protein YIPF4	YIPF4			
Q9P1D8	Putative uncharacterized protein PRO2289	PRO2289			
Q2QGD7	Zinc finger protein ZXDC	ZXDC			
Q6ZN11	Zinc finger protein 793	ZNF793			
Q9UL58	Zinc finger protein 215	ZNF215			
Q96B54	Zinc finger protein 428	ZNF428			
Q96MN9	Zinc finger protein 488	ZNF488			
Q7Z4V0	Zinc finger protein 438	ZNF438			
Q8TBC5	Zinc finger and SCAN domain-containing protein 18	ZSCAN18			
Q9ULM2	Zinc finger protein 490	ZNF490			
Q8NB42	Zinc finger protein 527	ZNF527			
Q86UE3	Zinc finger protein 546	ZNF546			
Q9H7S9	Zinc finger protein 703	ZNF703			
Q6P9A1	Zinc finger protein 530	ZNF530			
O43361	Zinc finger protein 749	ZNF749			
P21281	V-type proton ATPase subunit B, brain isoform	ATP6V1B2			
Q15836	Vesicle-associated membrane protein 3	VAMP3			
Q8N5D0	WD and tetratricopeptide repeats protein 1	WDTC1			

Q8N402	Putative uncharacterized protein LOC388882				
Q96NS1	Protein yippee-like 4	YPEL4			
Q96FA7	ZBED6 C-terminal-like protein	ZBED6CL			
Q6ZQV5	Putative KRAB domain-containing protein ZNF788	ZNF788P			
Q96DT7	Zinc finger and BTB domain-containing protein 10	ZBTB10			
Q05996	Zona pellucida sperm-binding protein 2	ZP2			
P51522	Zinc finger protein 83	ZNF83			
Q9UK55	Protein Z-dependent protease inhibitor	SERPINA10			
A6NHJ4	Zinc finger protein 860	ZNF860			
O43309	Zinc finger and SCAN domain-containing protein 12	ZSCAN12			
Q9UFB7	Zinc finger and BTB domain-containing protein 47	ZBTB47			
P0CG00	Putative zinc finger and SCAN domain-containing protein 5D	ZSCAN5DP			
Q02386	Zinc finger protein 45	ZNF45			
O95780	Zinc finger protein 682	ZNF682			
Q9UJW8	Zinc finger protein 180	ZNF180			
Q9NZL3	Zinc finger protein 224	ZNF224			
Q8N9Q2	Protein SREK1IP1	SREK1IP1			
P23381	Tryptophan--tRNA ligase, cytoplasmic	WARS1			
Q99426	Tubulin-folding cofactor B	TBCB			
Q96EM0	Trans-3-hydroxy-L-proline dehydratase	L3HYPDH			
Q5H9J9	T-complex protein 11 X-linked protein 2	TCP11X2			
Q7RTR8	Taste receptor type 2 member 42	TAS2R42			
Q9NZ63	Telomere length and silencing protein 1 homolog	C9orf78			
Q9UPZ6	Thrombospondin type-1 domain-containing protein 7A	THSD7A			
O43711	T-cell leukemia homeobox protein 3	TLX3			
P48023	Tumor necrosis factor ligand superfamily member 6	FASLG			
Q6ICL3	Transport and Golgi organization protein 2 homolog	TANGO2			
Q12767	Transmembrane protein 94	TMEM94			
Q13507	Short transient receptor potential channel 3	TRPC3			
A0A0K0K1A3	T cell receptor beta variable 10-1	TRBV10-1			
H0UI37	Thiosulfate sulfurtransferase/rhodanese-like domain-containing protein 3	TSTD3			
P60604	Ubiquitin-conjugating enzyme E2 G2	UBE2G2			
Q4G0F5	Vacuolar protein sorting-associated protein 26B	VPS26B			
Q96FK6	WD repeat-containing protein 89	WDR89			
Q86VR7	V-set and immunoglobulin domain-containing protein 10-like	VSIG10L			
Q86Y07	Serine/threonine-protein kinase VRK2	VRK2			
P63124	Endogenous retrovirus group K member 104 Pro protein	HERV-K104			
P12018	Immunoglobulin iota chain	VPREB1			
O15213	WD repeat-containing protein 46	WDR46			
Q2M389	WASH complex subunit 4	WASHC4			
Q6ZTI0	Putative uncharacterized protein FLJ44636				
Q9BRP9	Putative uncharacterized protein MGC13053				

Q9H521	Putative uncharacterized protein LOC645739				
Q96NJ1	Uncharacterized protein FLJ30774				
Q9UHT4	Putative uncharacterized protein PRO1854	PRO1854			
Q92536	Y+L amino acid transporter 2	SLC7A6			
Q96M85	Putative uncharacterized protein FLJ32756				
Q96M66	Putative uncharacterized protein FLJ32790				
A8MUU9	Putative uncharacterized protein ENSP00000383309				
Q6ZPA2	Putative uncharacterized protein FLJ26174				
Q9HBF4	Zinc finger FYVE domain-containing protein 1	ZFYVE1			
A6NP61	ZAR1-like protein	ZAR1L			
Q8NHG8	E3 ubiquitin-protein ligase ZNRF2	ZNRF2			
Q86XU0	Zinc finger protein 677	ZNF677			
P17019	Zinc finger protein 708	ZNF708			
Q8N988	Zinc finger protein 557	ZNF557			
Q9Y2Q1	Zinc finger protein 257	ZNF257			
Q9H963	Putative zinc finger protein 702	ZNF702P			
Q96CV9	Optineurin	OPTN			
Q8NGE1	Olfactory receptor 6C4	OR6C4			
A6NKK0	Olfactory receptor 5H1	OR5H1			
Q8NH43	Olfactory receptor 4L1	OR4L1			
Q8NGA2	Putative olfactory receptor 7A2	OR7A2P			
Q02218	2-oxoglutarate dehydrogenase, mitochondrial	OGDH			
P49790	Nuclear pore complex protein Nup153	NUP153			
Q6IF82	Olfactory receptor 4A47	OR4A47			
Q15743	Ovarian cancer G-protein coupled receptor 1	GPR68			
Q8TAD7	Overexpressed in colon carcinoma 1 protein	OCC1			
Q8NH60	Olfactory receptor 52J3	OR52J3			
Q9BXI2	Mitochondrial ornithine transporter 2	SLC25A2			
Q9NP74	Palmdelphin	PALMD			
Q9UKX7	Nuclear pore complex protein Nup50	NUP50			
O75459	P antigen family member 1	PAGE1			
Q9Y5H7	Protocadherin alpha-5	PCDHA5			
Q8TEW0	Partitioning defective 3 homolog	PARD3			
Q96JQ0	Protocadherin-16	DCHS1			
Q96TA0	Putative protocadherin beta-18	PCDHB18P			
Q15077	P2Y purinoceptor 6	P2RY6			
Q96A73	Putative monooxygenase p33MONOX	KIAA1191			
Q5R387	Putative inactive group IIC secretory phospholipase A2	PLA2G2C			
Q9NRJ7	Protocadherin beta-16	PCDHB16			
Q9NWS1	PCNA-interacting partner	PARPBP			
Q9H832	Ubiquitin-conjugating enzyme E2 Z	UBE2Z			
Q15750	TGF-beta-activated kinase 1 and MAP3K7-binding protein 1	TAB1			
Q12962	Transcription initiation factor TFIID subunit 10	TAF10			
Q15814	Tubulin-specific chaperone C	TBCC			
Q14DG7	Transmembrane protein 132B	TMEM132B			
P29083	General transcription factor IIE subunit 1	GTF2E1			
P24821	Tenascin	TNC			
O60220	Mitochondrial import inner membrane translocase subunit Tim8 A	TIMM8A			

Q68CZ2	Tensin-3	TNS3			
Q9H6X4	Transmembrane protein 134	TMEM134			
Q9UI38	Probable threonine protease PRSS50	PRSS50			
Q9UJK0	18S rRNA aminocarboxypropyltransferase	TSR3			
Q86VQ6	Thioredoxin reductase 3	TXNRD3			
Q7RTX0	Taste receptor type 1 member 3	TAS1R3			
P19224	UDP-glucuronosyltransferase 1-6	UGT1A6			
P0DTE5	UDP-glucuronosyltransferase 2A2	UGT2A2			
Q9NYU2	UDP-glucose:glycoprotein glucosyltransferase 1	UGGT1			
Q86UV5	Ubiquitin carboxyl-terminal hydrolase 48	USP48			
P25490	Transcriptional repressor protein YY1	YY1			
P35916	Vascular endothelial growth factor receptor 3	FLT4			
O15231	Zinc finger protein 185	ZNF185			
Q8N859	Zinc finger protein 713	ZNF713			
Q6ZS27	Zinc finger protein 662	ZNF662			
Q9BS31	Zinc finger protein 649	ZNF649			
Q9UK12	Zinc finger protein 222	ZNF222			
Q86YJ6	Threonine synthase-like 2	THNSL2			
Q969F1	General transcription factor 3C polypeptide 6	GTF3C6			
Q8WZ71	Transmembrane protein 158	TMEM158			
Q99805	Transmembrane 9 superfamily member 2	TM9SF2			
P02788	Lactotransferrin	LTF			
Q5T6R2	Putative phosphatidylinositol 3,4,5-trisphosphate 3-phosphatase TPTE2P1	TPTE2P1			
P25942	Tumor necrosis factor receptor superfamily member 5	CD40			
Q9BRZ2	E3 ubiquitin-protein ligase	TRIM56			
P25445	Tumor necrosis factor receptor superfamily member 6	FAS			
Q9P0U1	Mitochondrial import receptor subunit TOM7 homolog	TOMM7			
Q96SJ8	Tetraspanin-18	TSPAN18			
A0A075B6W5	T cell receptor alpha variable 23/delta variable 6	TRAV23DV6			
A0A0B4J27	T cell receptor alpha variable 24	TRAV24			
A0A539	T cell receptor beta variable 4-2	TRBV4-2			
Q9BXA6	Testis-specific serine/threonine-protein kinase 6	TSSK6			
Q9BSA4	Protein tweety homolog 2	TTYH2			
P0CG47	Polyubiquitin-B	UBB			
Q9BZV1	UBX domain-containing protein 6	UBXN6			
P0CG48	Polyubiquitin-C	UBC			
Q8IWV8	E3 ubiquitin-protein ligase UBR2	UBR2			
Q13107	Ubiquitin carboxyl-terminal hydrolase 4	USP4			
Q6RSH7	von Hippel-Lindau-like protein	VHLL			
O75348	V-type proton ATPase subunit G 1	ATP6V1G1			
Q8N9X3	Putative uncharacterized protein encoded by LINC01356	LINC01356			
Q9NRH1	Protein YAE1 homolog	YAE1			
Q8N377	Putative uncharacterized protein LOC387726				
Q9NSD4	Zinc finger protein 275	ZNF275			
Q8WV37	Zinc finger protein 480	ZNF480			
Q6ZMW2	Zinc finger protein 782	ZNF782			
O15015	Zinc finger protein 646	ZNF646			
Q9UII5	Zinc finger protein 107	ZNF107			
Q5T619	Zinc finger protein 648	ZNF648			
Q9NR00	Transcriptional and immune response regulator	TCIM			
Q70YC4	Talanin	ZNF365			

P00734	Prothrombin	F2			
Q6UX40	Transmembrane protein 107	TMEM107			
Q15554	Telomeric repeat-binding factor 2	TERF2			
Q5JUR7	Testis-expressed protein 30	TEX30			
Q00059	Transcription factor A, mitochondrial	TFAM			
Q8IYF3	Testis-expressed protein 11	TEX11			
A0A0B4J20	T cell receptor beta joining 2-3	TRBJ2-3			
Q9UNN4	TFIIA-alpha and beta-like factor	GTF2A1L			
P25325	3-mercaptopyruvate sulfurtransferase	MPST			
P42765	3-ketoacyl-CoA thiolase, mitochondrial	ACAA2			
C9J3V5	Testis-expressed protein 22	TEX22			
P01135	Protransforming growth factor alpha	TGFA			
Q9NWH2	Transmembrane protein 242	TMEM242			
O15050	TPR and ankyrin repeat-containing protein 1	TRANK1			
Q5T215	Trafficking protein particle complex subunit 3-like protein	TRAPPC3L			
Q9H3N1	Thioredoxin-related transmembrane protein 1	TMX1			
Q6XPS3	Phosphatidylinositol 3,4,5-trisphosphate 3-phosphatase TPTE2	TPTE2			
P07951	Tropomyosin beta chain	TPM2			
Q8NHH1	Tubulin polyglutamylase TTLL11	TTLL11			
A0A075B6L6	Probable non-functional T cell receptor beta variable 7-3	TRBV7-3			
Q93009	Ubiquitin carboxyl-terminal hydrolase 7	USP7			
Q01081	Splicing factor U2AF 35 kDa subunit	U2AF1			
Q9Y2X8	Ubiquitin-conjugating enzyme E2 D4	UBE2D4			
O00534	von Willebrand factor A domain-containing protein 5A	VWA5A			
A1L167	Ubiquitin-conjugating enzyme E2Q-like protein 1	UBE2QL1			
Q99986	Serine/threonine-protein kinase VRK1	VRK1			
Q8IWA0	WD repeat-containing protein 75	WDR75			
Q9Y6I0	Endogenous retrovirus group K member 6 Pro protein	ERVK-6			
Q13488	V-type proton ATPase 116 kDa subunit a3	TCIRG1			
P0DPA2	V-set and immunoglobulin domain-containing protein 8	VSIG8			
P63122	Endogenous retrovirus group K member 8 Pro protein	ERVK-8			
Q9UBV4	Protein Wnt-16	WNT16			
Q8N9V3	WD repeat, SAM and U-box domain-containing protein 1	WDSUB1			
O00308	NEDD4-like E3 ubiquitin-protein ligase WWP2	WWP2			
Q6AWC8	Putative uncharacterized protein LOC100129027				
O95070	Protein YIF1A	YIF1A			
A6NL46	Putative UPF0607 protein ENSP00000332738				
Q8NFD4	Uncharacterized protein FLJ76381				
Q6UWF5	Putative uncharacterized protein UNQ5815/PRO19632	UNQ5815/PRO19632			
A4D1N5	Putative uncharacterized protein FLJ40288				
Q6ZQT7	Putative uncharacterized protein FLJ44672				

Q6ZSR6	Putative uncharacterized protein FLJ45256				
Q96T25	Zinc finger protein ZIC 5	ZIC5			
O95409	Zinc finger protein ZIC 2	ZIC2			
O60281	Zinc finger protein 292	ZNF292			
E7ETH6	Zinc finger protein 587B	ZNF587B			
Q9HC78	Zinc finger and BTB domain-containing protein 20	ZBTB20			
Q17R98	Zinc finger protein 827	ZNF827			
O75800	Zinc finger MYND domain-containing protein 10	ZMYND10			
Q9BWM5	Zinc finger protein 416	ZNF416			
Q9H609	Zinc finger protein 576	ZNF576			
O95789	Zinc finger MYM-type protein 6	ZMYM6			
Q96AP4	Zinc finger-containing ubiquitin peptidase 1	ZUP1			
Q9Y2A4	Zinc finger protein 443	ZNF443			
Q8TD23	Zinc finger protein 675	ZNF675			
Q9Y4E5	E3 SUMO-protein ligase ZNF451	ZNF451			
Q8N972	Zinc finger protein 709	ZNF709			
Q9H7M6	Zinc finger SWIM domain-containing protein 4	ZSWIM4			
Q70EL4	Ubiquitin carboxyl-terminal hydrolase 43	USP43			
Q3ZAQ7	Vacuolar ATPase assembly integral membrane protein VMA21	VMA21			
Q96GC9	Vacuole membrane protein 1	VMP1			
Q96IQ7	V-set and immunoglobulin domain-containing protein 2	VSIG2			
Q5GH73	XK-related protein 6	XKR6			
Q9NRW7	Vacuolar protein sorting-associated protein 45	VPS45			
Q9C0E2	Exportin-4	XPO4			
O43895	Xaa-Pro aminopeptidase 2	XPNPEP2			
Q9BRG1	Vacuolar protein-sorting-associated protein 25	VPS25			
Q8IZU2	WD repeat-containing protein 17	WDR17			
Q5JTN6	WD repeat-containing protein 38	WDR38			
Q9GZL7	Ribosome biogenesis protein WDR12	WDR12			
O00755	Protein Wnt-7a	WNT7A			
Q9NS37	CREB/ATF bZIP transcription	CREBZF			
O60688	Protein yippee-like 1	YPEL1			
A2RRD8	Zinc finger protein 320	ZNF320			
Q8N3Z6	Zinc finger CCHC domain-containing protein 7	ZCCHC7			
P0CI00	Putative zinc finger protein 705B	ZNF705B			
Q5T200	Zinc finger CCCH domain-containing protein 13	ZC3H13			
Q8NCP5	Zinc finger and BTB domain-containing protein 44	ZBTB44			
Q96N95	Zinc finger protein 396	ZNF396			
Q9BYN7	Zinc finger protein 341	ZNF341			
Q09FC8	Zinc finger protein 415	ZNF415			
Q6V9R5	Zinc finger protein 562	ZNF562			
Q14202	Zinc finger MYM-type protein 3	ZMYM3			
P15621	Zinc finger protein 44	ZNF44			
Q401N2	Zinc-activated ligand-gated ion channel	ZACN			
P52744	Zinc finger protein 138	ZNF138			
Q06730	Zinc finger protein 33A	ZNF33A			
Q6ZSB9	Zinc finger and BTB domain-containing protein 49	ZBTB49			
Q76KX8	Zinc finger protein 534	ZNF534			
P52741	Zinc finger protein 134	ZNF134			
O95125	Zinc finger protein 202	ZNF202			
A7E2V4	Zinc finger SWIM domain-containing protein 8	ZSWIM8			
Q9H8N7	Zinc finger protein 395	ZNF395			
Q96I27	Zinc finger protein 625	ZNF625			

Q96K58	Zinc finger protein 668	ZNF668			
Q9NVE5	Ubiquitin carboxyl-terminal hydrolase 40	USP40			
O15195	Villin-like protein	VILL			
P41587	Vasoactive intestinal polypeptide receptor 2	VIPR2			
Q5T4S7	E3 ubiquitin-protein ligase UBR4	UBR4			
P55808	Glycoprotein Xg	XG			
P51809	Vesicle-associated membrane protein 7	VAMP7			
Q6P2C0	WD repeat-containing protein 93	WDR93			
Q9UBQ0	Vacuolar protein sorting-associated protein 29	VPS29			
A8K0Z3	WASH complex subunit 1	WASHC1			
Q92558	Wiskott-Aldrich syndrome protein family member 1	WASF1			
Q6UX27	V-set and transmembrane domain-containing protein 1	VSTM1			
A5D8V6	Vacuolar protein sorting-associated protein 37C	VPS37C			
P47992	Lymphotactin	XCL1			
Q8NEZ2	Vacuolar protein sorting-associated protein 37A	VPS37A			
Q9UBD3	Cytokine SCM-1 beta	XCL2			
Q9UPY5	Cystine/glutamate transporter	SLC7A11			
Q6UY13	Putative uncharacterized protein UNQ5830/PRO19650/PRO19816	UNQ5830/ PRO19650/ PRO19816			
A8MZ25	Putative uncharacterized protein FLJ38767				
Q9UI54	Putative uncharacterized protein PRO0628	PRO0628			
Q8TCF1	AN1-type zinc finger protein 1	ZFAND1			
Q6ZTB9	Putative zinc finger protein 833	ZNF833P			
P17022	Zinc finger protein 18	ZNF18			
Q5T4F4	Protrudin	ZFYVE27			
P17028	Zinc finger protein 24	ZNF24			
P17031	Zinc finger protein 26	ZNF26			
Q96JM2	Zinc finger protein 462	ZNF462			
Q9NUD5	Zinc finger CCHC domain-containing protein 3	ZCCHC3			
Q75MW2	Protein ZNF767	ZNF767P			
Q86XF7	Zinc finger protein 575	ZNF575			
Q5VV52	Zinc finger protein 691	ZNF691			
Q8NEM1	Zinc finger protein 680	ZNF680			
Q9P217	Zinc finger SWIM domain-containing protein 5	ZSWIM5			
Q14592	Zinc finger protein 460	ZNF460			
Q14584	Zinc finger protein 266	ZNF266			
Q14586	Zinc finger protein 267	ZNF267			
B4DX44	Zinc finger protein 736	ZNF736			
O75373	Zinc finger protein 737	ZNF737			
O15533	Tapasin	TAPBP			
P32971	Tumor necrosis factor ligand superfamily member 8	TNFSF8			
Q13641	Trophoblast glycoprotein	TPBG			
P62995	Transformer-2 protein homolog beta	TRA2B			
Q13595	Transformer-2 protein homolog alpha	TRA2A			
Q86W33	Transmembrane protein adipocyte-associated 1	TPRA1			
P45379	Troponin T, cardiac muscle	TNNT2			
Q9P0T7	Proton-transporting V-type ATPase complex assembly regulator TMEM9	TMEM9			
P57088	Transmembrane protein 33	TMEM33			
Q6P5X7	Transmembrane protein 71	TMEM71			

Q8N427	Thioredoxin domain-containing protein 3	NME8			
Q6P7N7	Transmembrane protein 81	TMEM81			
Q9BQJ4	Transmembrane protein 47	TMEM47			
Q6UWJ1	Transmembrane and coiled-coil domain-containing protein 3	TMCO3			
P17152	Transmembrane protein 11, mitochondrial	TMEM11			
Q15672	Twist-related protein 1	TWIST1			
A0A0B4J24	T cell receptor alpha variable 3	TRAV3			
O00322	Uroplakin-1a	UPK1A			
Q9BZM5	UL16-binding protein 2	ULBP2			
Q96S82	Ubiquitin-like protein 7	UBL7			
Q96LR5	Ubiquitin-conjugating enzyme E2 E2	UBE2E2			
Q96AW1	Vesicular, overexpressed in cancer, prosurvival protein 1	VOPP1			
Q9P2H5	Ubiquitin carboxyl-terminal hydrolase 35	USP35			
Q8N2K1	Ubiquitin-conjugating enzyme E2 J2	UBE2J2			
Q8TDU5	Putative vomeronasal receptor-like protein 4	VN1R17P			
O14598	Testis-specific basic protein Y 1	VCY			
P23763	Vesicle-associated membrane protein 1	VAMP1			
Q9Y277	Voltage-dependent anion-selective channel protein 3	VDAC3			
Q9Y487	V-type proton ATPase 116 kDa subunit a2	ATP6V0A2			
Q9UKI3	Pre-B lymphocyte protein 3	VPREB3			
Q96DN2	von Willebrand factor C and EGF domain-containing protein	VWCE			
A8MXK1	V-set and transmembrane domain-containing protein 5	VSTM5			
Q9NX94	WW domain binding protein 1-	WBP1L			
Q9Y6I7	WD repeat and SOCS box-containing protein 1	WSB1			
Q8N8F6	Protein YIPF7	YIPF7			
Q6UXV3	Uncharacterized protein UNQ6126/PRO20091	UNQ6126/ PRO20091			
Q96IU2	Zinc finger BED domain-containing protein 3	ZBED3			
Q8N2I2	Zinc finger protein 619	ZNF619			
Q86WZ6	Zinc finger protein 227	ZNF227			
Q7L3S4	Zinc finger protein 771	ZNF771			
Q6NX45	Zinc finger protein 774	ZNF774			
Q96HQ0	Zinc finger protein 419	ZNF419			
O75437	Zinc finger protein 254	ZNF254			
Q8NAM6	Zinc finger and SCAN domain-containing protein 4	ZSCAN4			
A1L4H1	Soluble scavenger receptor cysteine-rich domain-containing protein SSC5D	SSC5D			
O75478	Transcriptional adapter 2-alpha	TADA2A			
Q96BZ9	TBC1 domain family member 20	TBC1D20			
Q9UJT1	Tubulin delta chain	TUBD1			
Q13207	T-box transcription factor TBX2	TBX2			
P10636	Microtubule-associated protein tau	MAPT			
Q14188	Transcription factor Dp-2	TFDP2			
Q6NXR4	TELO2-interacting protein 2	TTI2			
A6NIH7	Protein unc-119 homolog B	UNC119B			
Q6ZUT4	Putative uncharacterized protein FLJ43343				
Q96MT0	Putative uncharacterized protein FLJ31958				
Q9BZS9	Putative uncharacterized protein PNAS-138	PNAS-138			

Q6WRX3	Protein zyg-11 homolog A	ZYG11A			
P98168	Zinc finger X-linked protein ZXDA	ZXDA			
Q15915	Zinc finger protein ZIC 1	ZIC1			
Q9H5H4	Zinc finger protein 768	ZNF768			
Q96F45	Zinc finger protein 503	ZNF503			
O95229	ZW10 interactor	ZWINT			
Q5JVG8	Zinc finger protein 506	ZNF506			
Q9H9D4	Zinc finger protein 408	ZNF408			
Q8IZ20	Tissue-resident T-cell transcription regulator protein ZNF683	ZNF683			
Q8N1G0	Zinc finger protein 687	ZNF687			
Q8NGB2	Olfactory receptor 4C5	OR4C5			
Q13607	Olfactory receptor 2F1	OR2F1			
Q6IEU7	Olfactory receptor 5M10	OR5M10			
P0DN78	Medium-wave-sensitive opsin 3	OPN1MW3			
Q8NGE8	Olfactory receptor 4D9	OR4D9			
Q96MR7	Putative uncharacterized protein OBSCN-AS1	OBSCN-AS1			
Q8N0Y5	Olfactory receptor 8I2	OR8I2			
A4D2G3	Olfactory receptor 2A25	OR2A25			
P23515	Oligodendrocyte-myelin glycoprotein	OMG			
Q8NH61	Olfactory receptor 51F2	OR51F2			
Q5VZ03	Nucleoredoxin-like protein 2	NXNL2			
Q01968	Inositol polyphosphate 5-phosphatase OCRL	OCRL			
O15527	N-glycosylase/DNA lyase	OGG1			
Q9Y236	Oxidative stress-induced growth inhibitor 2	OSGIN2			
Q8TF05	Serine/threonine-protein phosphatase 4 regulatory subunit 1	PPP4R1			
Q9UKF7	Cytoplasmic phosphatidylinositol transfer protein 1	PITPNC1			
Q01970	1-phosphatidylinositol 4,5-bisphosphate phosphodiesterase beta-3	PLCB3			
E5RG02	Putative serine protease 46	PRSS46P			
Q96NZ9	Proline-rich acidic protein 1	PRAP1			
O60811	PRAME family member 2	PRAMEF2			
Q6MZM9	Proline-rich protein 27	PRR27			
Q5T6V5	Queuosine salvage protein	C9orf64			
Q06141	Regenerating islet-derived protein 3-alpha	REG3A			
A6NC62	Putative RBAK downstream neighbor protein	RBAKDN			
P29558	RNA-binding motif, single-stranded-interacting protein 1	RBMS1			
Q96D21	GTP-binding protein Rhes	RASD2			
Q9H0Z9	RNA-binding protein 38	RBM38			
Q8N264	Rho GTPase-activating protein 24	ARHGAP24			
Q6NW40	RGM domain family member B	RGMB			
Q9UGC7	Peptide chain release factor 1-like, mitochondrial	MTRF1L			
P49802	Regulator of G-protein signaling 7	RGS7			
P51606	N-acylglucosamine 2-epimerase	RENBP			
P31350	Ribonucleoside-diphosphate reductase subunit M2	RRM2			
P35249	Replication factor C subunit 4	RFC4			
Q9BYD1	39S ribosomal protein L13, mitochondrial	MRPL13			
Q96E39	RNA binding motif protein, X-linked-like-1	RBMXL1			
Q9NRX2	39S ribosomal protein L17, mitochondrial	MRPL17			
Q9Y6G3	39S ribosomal protein L42, mitochondrial	MRPL42			
Q9H9J2	39S ribosomal protein L44, mitochondrial	MRPL44			
P10155	60 kDa SS-A/Ro	RO60			

Q5M7Z0	E3 ubiquitin-protein ligase RNFT1	RNFT1			
Q86X10	Ral GTPase-activating protein subunit beta	RALGAPB			
Q96AT9	Ribulose-phosphate 3-epimerase	RPE			
Q8N6D2	E3 ubiquitin-protein ligase	RNF182			
Q2M238	Putative RRN3-like protein RRN3P1	RRN3P1			
E7ERA6	RING finger protein 223	RNF223			
O15027	Protein transport protein Sec16A	SEC16A			
P51172	Amiloride-sensitive sodium channel subunit delta	SCNN1D			
A3KN83	Protein strawberry notch homolog 1	SBNO1			
Q6BEB4	Transcription factor Sp5	SP5			
Q86U17	Serpin A11	SERPINA11			
Q9Y2E8	Sodium/hydrogen exchanger 8	SLC9A8			
Q9H7B4	Histone-lysine N-methyltransferase SMYD3	SMYD3			
Q92581	Sodium/hydrogen exchanger 6	SLC9A6			
P57768	Sorting nexin-16	SNX16			
Q12824	SWI/SNF-related matrix-associated actin-dependent regulator of chromatin subfamily B member 1	SMARCB1			
Q14190	Single-minded homolog 2	SIM2			
Q9NS26	Sperm protein associated with the nucleus on the X chromosome A	SPANXA1			
Q13103	Secreted phosphoprotein 24	SPP2			
P0DI80	Small integral membrane protein	SMIM6			
Q9Y5X2	Sorting nexin-8	SNX8			
P01286	Somatoliberin	GHRH			
A6NLX3	Speedy protein E4	SPDYE4			
A6NJR5	Putative speedy protein-like protein 3				
Q92185	Alpha-N-acetylneuraminide alpha-2,8-sialyltransferase	ST8SIA1			
Q5JUX0	Spindlin-3	SPIN3			
P50452	Serpin B8	SERPINB8			
Q08AF3	Schlafen family member 5	SLFN5			
A0MZ66	Shootin-1	SHTN1			
Q15772	Striated muscle preferentially expressed protein kinase	SPEG			
Q7RTT4	Putative protein SSX8	SSX8P			
Q8IZU3	Synaptonemal complex protein 3	SYCP3			
P0DMN0	Sulfotransferase 1A4	SULT1A4			
Q9Y6E0	Serine/threonine-protein kinase	STK24			
Q7KZF4	Staphylococcal nuclease domain-containing protein 1	SND1			
P51687	Sulfite oxidase, mitochondrial	SUOX			
O43805	Sjoegren syndrome nuclear autoantigen 1	SSNA1			
Q96FJ0	AMSH-like protease	STAMBPL1			
Q9P289	Serine/threonine-protein kinase	STK26			
O43815	Striatin	STRN			
Q9BXU3	Testis-expressed protein 13A	TEX13A			
Q9BZA5	Putative gamma-taxilin 2	TXLNGY			
Q8N4V2	Synaptic vesicle 2-related protein	SVOP			
Q8WVY7	Ubiquitin-like domain-containing CTD phosphatase 1	UBLCP1			
Q9UPU5	Ubiquitin carboxyl-terminal hydrolase 24	USP24			
P09936	Ubiquitin carboxyl-terminal hydrolase isozyme L1	UCHL1			
Q6ZMZ3	Nesprin-3	SYNE3			
Q8N3V7	Synaptopodin	SYNPO			
Q5T5C0	Syntaxin-binding protein 5	STXBP5			
Q8WXF0	Serine/arginine-rich splicing factor 12	SRSF12			
Q7L0J3	Synaptic vesicle glycoprotein 2A	SV2A			

O43818	U3 small nucleolar RNA-interacting protein 2	RRP9			
Q9NYW1	Taste receptor type 2 member 9	TAS2R9			
O95935	T-box transcription factor TBX18	TBX18			
P0DSN6	Putative taste receptor type 2 member 33	TAS2R33			
P26639	Threonine--tRNA ligase 1, cytoplasmic	TARS1			
P59540	Taste receptor type 2 member 46	TAS2R46			
Q8NA31	Telomere repeats-binding bouquet formation protein 1	TERB1			
Q8N6V9	Testis-expressed protein 9	TEX9			
O43247	Testis-expressed protein 33	TEX33			
P54274	Telomeric repeat-binding factor 1	TERF1			
Q96AN5	Transmembrane protein 143	TMEM143			
A0A0J9YWP8	T cell receptor beta joining 1-3	TRBJ1-3			
Q01664	Transcription factor AP-4	TFAP4			
Q96AQ2	Transmembrane protein 125	TMEM125			
Q5VWP2	Terminal nucleotidyltransferase 5C	TENT5C			
Q9H808	Transducin-like enhancer protein	TLE6			
Q96RS0	Trimethylguanosine synthase	TGS1			
Q96Q45	Transmembrane protein 237	TMEM237			
Q5SV17	Transmembrane protein 240	TMEM240			
Q5HYJ1	Trans-2,3-enoyl-CoA reductase-like	TECRL			
A0A0B4J1U4	T cell receptor gamma variable 5	TRGV5			
Q7Z2V1	Protein TNT	C16orf82			
Q86WT6	E3 ubiquitin-protein ligase	TRIM69			
Q68CL5	Tubulin polyglutamylase complex subunit 2	TPGS2			
O14773	Tripeptidyl-peptidase 1	TPP1			
Q8IWU9	Tryptophan 5-hydroxylase 2	TPH2			
Q96B49	Mitochondrial import receptor subunit TOM6 homolog	TOMM6			
Q6P589	Tumor necrosis factor alpha-induced protein 8-like protein 2	TNFAIP8L2			
Q5SWH9	Transmembrane protein 69	TMEM69			
Q9Y6Q6	Tumor necrosis factor receptor superfamily member 11A	TNFRSF11A			
Q93038	Tumor necrosis factor receptor superfamily member 25	TNFRSF25			
Q8WWA1	Transmembrane protein 40	TMEM40			
O75386	Tubby-related protein 3	TULP3			
P40126	L-dopachrome tautomerase	DCT			
A0A0B4J27	T cell receptor alpha variable 22	TRAV22			
A0JD32	T cell receptor alpha variable 38-2/delta variable 8	TRAV38-2DV8			
A0A075B6X5	T cell receptor alpha variable 18	TRAV18			
A0A5B6	T cell receptor beta variable 28	TRBV28			
Q9H489	Putative testis-specific Y-encoded-like protein 3	TSPY26P			
A0A0A0MS06	Probable non-functional T cell receptor beta variable 23-1	TRBV23-1			
Q8WU66	Thrombospondin-type laminin G domain and EAR repeat-containing protein	TSPEAR			
A0A5B7	T cell receptor beta variable 29-1	TRBV29-1			
Q9BX84	Transient receptor potential cation channel subfamily M member 6	TRPM6			
O00762	Ubiquitin-conjugating enzyme E2 C	UBE2C			
P22309	UDP-glucuronosyltransferase 1A1	UGT1A1			
Q5T124	UBX domain-containing protein 11	UBXN11			
P54855	UDP-glucuronosyltransferase	UGT2B15			

Q9HAC8	Ubiquitin domain-containing protein 1	UBTD1			
P0DTE4	UDP-glucuronosyltransferase 2A1	UGT2A1			
Q9H321	Variable charge X-linked protein 3B	VCX3B			
Q9NZ43	Vesicle transport protein USE1	USE1			
Q9Y462	Zinc finger protein 711	ZNF711			
Q86XI8	Uncharacterized protein ZSWIM9	ZSWIM9			
Q9P0L1	Zinc finger protein with KRAB and SCAN domains 7	ZKSCAN7			
Q5T5D7	Zinc finger protein 684	ZNF684			
Q9Y2D9	Zinc finger protein 652	ZNF652			
Q6P280	Zinc finger protein 529	ZNF529			
Q9NR12	PDZ and LIM domain protein 7	PDLIM7			
O95453	Poly	PARN			
Q9UHJ9	Post-GPI attachment to proteins factor 2	PGAP2			
P78424	POU domain, class 6, transcription factor 2	POU6F2			
Q06830	Peroxiredoxin-1	PRDX1			
Q5JSZ5	Protein PRRC2B	PRRC2B			
P13727	Bone marrow proteoglycan	PRG2			
Q9HCU5	Prolactin regulatory element-binding protein	PREB			
Q8IYL9	Psychosine receptor	GPR65			
Q8N4Q0	Prostaglandin reductase 3	ZADH2			
O00233	26S proteasome non-ATPase regulatory subunit 9	PSMD9			
Q9UPQ7	E3 ubiquitin-protein ligase PDZRN3	PDZRN3			
P11216	Glycogen phosphorylase, brain form	PYGB			
P12931	Proto-oncogene tyrosine-protein kinase Src	SRC			
Q96BN2	Transcriptional adapter 1	TADA1			
Q6ZT07	TBC1 domain family member 9	TBC1D9			
Q8N1K5	Protein THEMIS	THEMIS			
Q9BVX2	Transmembrane protein 106C	TMEM106C			
Q6PEX7	Testis-expressed protein 38	TEX38			
C9JI98	Transmembrane protein 238	TMEM238			
Q9BU79	Transmembrane protein 243	TMEM243			
Q96B77	Transmembrane protein 186	TMEM186			
Q8IZ69	tRNA	TRMT2A			
Q9UK28	Transmembrane protein 59-like	TMEM59L			
Q15185	Prostaglandin E synthase 3	PTGES3			
Q2MV58	Tectonic-1	TCTN1			
Q96Q11	CCA tRNA nucleotidyltransferase 1, mitochondrial	TRNT1			
Q9BQ61	Telomerase RNA component interacting RNase	TRIR			
P55327	Tumor protein D52	TPD52			
Q8N816	Transmembrane protein 99	TMEM99			
Q3KNT9	Sperm-egg fusion protein TMEM95	TMEM95			
P00750	Tissue-type plasminogen activator	PLAT			
Q9Y2Y6	Transmembrane protein 98	TMEM98			
O43557	Tumor necrosis factor ligand superfamily member 14	TNFSF14			
O15016	Tripartite motif-containing protein 66	TRIM66			
Q9H3S4	Thiamin pyrophosphokinase 1	TPK1			
Q7Z7H5	Transmembrane emp24 domain-containing protein 4	TMED4			
Q9Y3A6	Transmembrane emp24 domain-containing protein 5	TMED5			
A0PJX8	Transmembrane protein 82	TMEM82			
A0A0B4J28	T cell receptor alpha variable 40	TRAV40			
Q02223	Tumor necrosis factor receptor superfamily member 17	TNFRSF17			

A0A087WT03	T cell receptor alpha variable 26-1	TRAV26-1			
O43914	TYRO protein tyrosine kinase-binding protein	TYROBP			
P54578	Ubiquitin carboxyl-terminal hydrolase 14	USP14			
O94874	E3 UFM1-protein ligase 1	UFL1			
Q96LJ8	UBX domain-containing protein 10	UBXN10			
Q6NUS8	UDP-glucuronosyltransferase 3A1	UGT3A1			
A6NNY8	Ubiquitin carboxyl-terminal hydrolase 27	USP27X			
O75604	Ubiquitin carboxyl-terminal hydrolase 2	USP2			
Q70EL1	Inactive ubiquitin carboxyl-terminal hydrolase 54	USP54			
Q6PFW1	Inositol hexakisphosphate and diphosphoinositol-pentakisphosphate kinase 1	PPIP5K1			
Q9NRM2	Zinc finger protein 277	ZNF277			
Q8N8E2	Zinc finger protein 513	ZNF513			
Q9P2Y4	Zinc finger protein 219	ZNF219			
A6NK53	Zinc finger protein 233	ZNF233			
Q07955	Serine/arginine-rich splicing factor 1	SRSF1			
Q8WXH0	Nesprin-2	SYNE2			
Q8N0U8	Vitamin K epoxide reductase complex subunit 1-like protein 1	VKORC1L1			
Q9BUF5	Tubulin beta-6 chain	TUBB6			
P54577	Tyrosine--tRNA ligase, cytoplasmic	YARS1			
Q9BQE3	Tubulin alpha-1C chain	TUBA1C			
A0A1B0GUA7	Testis-expressed protein 51	TEX51			
Q9H5L6	DNA transposase THAP9	THAP9			
Q96EK4	THAP domain-containing protein 11	THAP11			
Q99757	Thioredoxin, mitochondrial	TXN2			
Q86UE8	Serine/threonine-protein kinase tousled-like 2	TLK2			
Q9NU19	TBC1 domain family member 22B	TBC1D22B			
Q9UIK5	Tomoregulin-2	TMEFF2			
Q9BRN9	TM2 domain-containing protein 3	TM2D3			
Q5QJE6	Deoxynucleotidyltransferase terminal-interacting protein 2	DNTTIP2			
O95807	Transmembrane protein 50A	TMEM50A			
P21580	Tumor necrosis factor alpha-induced protein 3	TNFAIP3			
Q96J77	Tumor protein D55	TPD52L3			
Q9UI30	Multifunctional methyltransferase subunit TRM112-like protein	TRMT112			
Q86SZ2	Trafficking protein particle complex subunit 6B	TRAPPC6B			
Q6ZUI0	Tumor protein p63-regulated gene 1 protein	TPRG1			
A8MYB1	Transmembrane and coiled-coil domain-containing protein 5B	TMCO5B			
Q86XW9	Thioredoxin domain-containing protein 6	NME9			
P53007	Tricarboxylate transport protein, mitochondrial	SLC25A1			
A0A0B4J26	T cell receptor alpha variable 26-2	TRAV26-2			
P01733	T cell receptor beta variable 12-3	TRBV12-3			
O75317	Ubiquitin carboxyl-terminal hydrolase 12	USP12			
O95847	Mitochondrial uncoupling protein 4	SLC25A27			
P40818	Ubiquitin carboxyl-terminal hydrolase 8	USP8			

Q13106	Zinc finger protein 154	ZNF154			
Q3SXZ3	Zinc finger protein 718	ZNF718			
Q3ZCT1	Zinc finger protein 260	ZNF260			
A8MUV8	Putative zinc finger protein 727	ZNF727			
Q16600	Zinc finger protein 239	ZNF239			
O94892	Zinc finger protein 432	ZNF432			
O43929	Origin recognition complex subunit 4	ORC4			
Q96R08	Olfactory receptor 5B12	OR5B12			
Q8NGS1	Olfactory receptor 1J4	OR1J4			
Q9UH64	Putative protein CDKN2A-DT	CDKN2A-DT			
Q8NGU2	Olfactory receptor 9A4	OR9A4			
Q8NH92	Olfactory receptor 1S1	OR1S1			
Q16633	POU domain class 2-associating factor 1	POU2AF1			
Q96E52	Metalloendopeptidase OMA1, mitochondrial	OMA1			
Q5JRS4	Olfactory receptor 10J3	OR10J3			
Q9H2C5	Olfactory receptor 52A5	OR52A5			
Q9P2P1	Protein NYNRIN	NYNRIN			
Q8NGY1	Olfactory receptor 10Z1	OR10Z1			
Q96RD2	Olfactory receptor 52B2	OR52B2			
Q9H343	Olfactory receptor 51I1	OR51I1			
Q8NH57	Putative olfactory receptor 52P1	OR52P1P			
Q8NHC6	Putative olfactory receptor 14L1	OR14L1P			
Q9BVG4	Protein PBDC1	PBDC1			
O60331	Phosphatidylinositol 4-phosphate 5-kinase type-1 gamma	PIP5K1C			
P07202	Thyroid peroxidase	TPO			
P11678	Eosinophil peroxidase	EPX			
Q8N0Y7	Probable phosphoglycerate mutase 4	PGAM4			
Q9HB75	p53-induced death domain-containing protein 1	PIDD1			
Q15319	POU domain, class 4, transcription factor 3	POU4F3			
Q99811	Paired mesoderm homeobox protein 2	PRRX2			
Q16827	Receptor-type tyrosine-protein phosphatase O	PTPRO			
P49590	Histidine--tRNA ligase, mitochondrial	HARS2			
Q15833	Syntaxin-binding protein 2	STXBP2			
O00268	Transcription initiation factor TFIID subunit 4	TAF4			
P29084	Transcription initiation factor IIE subunit beta	GTF2E2			
Q9UIF3	Tektin-2	TEKT2			
Q9NV29	Transmembrane protein 100	TMEM100			
P01033	Metalloproteinase inhibitor 1	TIMP1			
Q9BVC6	Transmembrane protein 109	TMEM109			
Q6UE05	Transmembrane protein 270	TMEM270			
Q969M1	Mitochondrial import receptor subunit TOM40B	TOMM40L			
Q9H0R3	Transmembrane protein 222	TMEM222			
Q9BXT4	Tudor domain-containing protein 1	TDRD1			
O75443	Alpha-tectorin	TECTA			
Q9GZU3	Transmembrane protein 39B	TMEM39B			
P07204	Thrombomodulin	THBD			
Q8WVP5	Tumor necrosis factor alpha-induced protein 8-like protein 1	TNFAIP8L1			
O15393	Transmembrane protease serine 2	TMPRSS2			
O95271	Poly	TNKS			
O14933	Ubiquitin/ISG15-conjugating enzyme E2 L6	UBE2L6			
Q8N6Y0	Usher syndrome type-1C protein-binding protein 1	USHBP1			

P13693	Translationally-controlled tumor protein	TPT1			
Q9BZK7	F-box-like/WD repeat-containing protein TBL1XR1	TBL1XR1			
Q9UK61	Protein TASOR	TASOR			
Q9P0N9	TBC1 domain family member 7	TBC1D7			
Q9UNS1	Protein timeless homolog	TIMELESS			
Q8TBZ6	tRNA methyltransferase 10 homolog A	TRMT10A			
A6NGC4	TLC domain-containing protein 2	TLCD2			
Q99603	T cell receptor gamma variable 9	TRGV9			
P28908	Tumor necrosis factor receptor superfamily member 8	TNFRSF8			
Q06643	Lymphotoxin-beta	LTB			
P63316	Troponin C, slow skeletal and cardiac muscles	TNNC1			
Q9Y2W1	Thyroid hormone receptor-associated protein 3	THRAP3			
Q9HCN2	p53-regulated apoptosis-inducing protein 1	TP53AIP1			
Q9BRA2	Thioredoxin domain-containing protein 17	TXNDC17			
Q9UL62	Short transient receptor potential channel 5	TRPC5			
A0A0B4J26	T cell receptor alpha variable 8-6	TRAV8-6			
Q9NNX1	Tuftelin	TUFT1			
Q8N831	Testis-specific Y-encoded-like protein 6	TSPYL6			
Q00341	Vigilin	HDLBP			
P01282	VIP peptides	VIP			
P51965	Ubiquitin-conjugating enzyme E2 E1	UBE2E1			
Q9UN37	Vacuolar protein sorting-associated protein 4A	VPS4A			
Q7Z7D3	V-set domain-containing T-cell activation inhibitor 1	VTCN1			
Q14508	WAP four-disulfide core domain protein 2	WFDC2			
Q9UEU0	Vesicle transport through interaction with t-SNAREs homolog 1B	VTI1B			
Q8TAG5	V-set and transmembrane domain-containing protein 2A	VSTM2A			
Q92889	DNA repair endonuclease XPF	ERCC4			
Q96JG6	Syndetin	VPS50			
Q9BTM9	Ubiquitin-related modifier 1	URM1			
Q9P1Q0	Vacuolar protein sorting-associated protein 54	VPS54			
Q8NFZ6	Vomeronasal type-1 receptor 2	VN1R2			
Q15906	Vacuolar protein sorting-associated protein 72 homolog	VPS72			
Q8N398	von Willebrand factor A domain-containing protein 5B2	VWA5B2			
P13010	X-ray repair cross-complementing protein 5	XRCC5			
Q6PJI9	GATOR complex protein WDR59	WDR59			
Q96GT9	X antigen family member 2	XAGE2			
Q9P202	Whirlin	WHRN			
Q8IXZ2	Zinc finger CCCH domain-containing protein 3	ZC3H3			
P52746	Zinc finger protein 142	ZNF142			
Q8N895	Zinc finger protein 366	ZNF366			
Q5EBL2	Zinc finger protein 628	ZNF628			
A6NNF4	Zinc finger protein 726	ZNF726			
Q92610	Zinc finger protein 592	ZNF592			
Q3MII6	TBC1 domain family member 25	TBC1D25			
Q9Y2Z4	Tyrosine--tRNA ligase, mitochondrial	YARS2			
Q9UI15	Transgelin-3	TAGLN3			

Q9Y3Q8	TSC22 domain family protein 4	TSC22D4			
Q8WUA4	General transcription factor 3C polypeptide 2	GTF3C2			
Q9Y5J7	Mitochondrial import inner membrane translocase subunit Tim9	TIMM9			
Q9NYW4	Taste receptor type 2 member 5	TAS2R5			
Q5BVD1	TPA-induced transmembrane protein	TTMP			
Q96AE7	Tetratricopeptide repeat protein 17	TTC17			
O95858	Tetraspanin-15	TSPAN15			
P62328	Thymosin beta-4	TMSB4X			
Q8TD43	Transient receptor potential cation channel subfamily M member 4	TRPM4			
A0A576	T cell receptor beta variable 3-1	TRBV3-1			
A0A075B6N4	T cell receptor beta variable 25-1	TRBV25-1			
Q6ZU65	Ubinuclein-2	UBN2			
Q9UMW8	Ubl carboxyl-terminal hydrolase 18	USP18			
Q9BZX2	Uridine-cytidine kinase 2	UCK2			
P45974	Ubiquitin carboxyl-terminal hydrolase 5	USP5			
P35968	Vascular endothelial growth factor receptor 2	KDR			
Q5T750	Skin-specific protein 32	XP32			
P63120	Endogenous retrovirus group K member 19 Pro protein	ERVK-19			
Q86VN1	Vacuolar protein-sorting-associated protein 36	VPS36			
Q8IZQ1	WD repeat and FYVE domain-containing protein 3	WDFY3			
Q969T9	WW domain-binding protein 2	WBP2			
P0DP72	V-set and immunoglobulin domain-containing protein 10-like 2	VSIG10L2			
Q9H1J5	Protein Wnt-8a	WNT8A			
P58557	Endoribonuclease YbeY	YBEY			
Q8TAT8	Putative uncharacterized protein LOC644613				
Q0VFX4	Putative uncharacterized protein LOC100128554				
Q9Y6X8	Zinc fingers and homeoboxes protein 2	ZHX2			
A8MWP4	Putative uncharacterized protein ENSP00000401716				
Q9H6S0	3'-5' RNA helicase YTHDC2	YTHDC2			
O60384	Putative zinc finger protein 861	ZNF861P			
O43167	Zinc finger and BTB domain-containing protein 24	ZBTB24			
Q07157	Tight junction protein ZO-1	TJP1			
P21506	Zinc finger protein 10	ZNF10			
Q6ZTR6	Putative uncharacterized protein ZNF516-DT	ZNF516-DT			
Q9P2F9	Zinc finger protein 319	ZNF319			
Q96K80	Zinc finger CCCH domain-containing protein 10	ZC3H10			
P17038	Zinc finger protein 43	ZNF43			
Q8N8Z8	Zinc finger protein 441	ZNF441			
Q9Y3M9	Zinc finger protein 337	ZNF337			
P17035	Zinc finger protein 28	ZNF28			
Q5VIY5	Zinc finger protein 468	ZNF468			
Q8NA42	Zinc finger protein 383	ZNF383			
Q96NG8	Zinc finger protein 582	ZNF582			
Q03924	Zinc finger protein 117	ZNF117			
Q96ND8	Zinc finger protein 583	ZNF583			
P52736	Zinc finger protein 133	ZNF133			

Q16670	Zinc finger and SCAN domain-containing protein 26	ZSCAN26			
Q14929	Zinc finger protein 169	ZNF169			
Q8N680	Zinc finger and BTB domain-containing protein 2	ZBTB2			
Q8TBZ5	Zinc finger protein 502	ZNF502			
Q8IYI8	Zinc finger protein 440	ZNF440			
Q9BVV6	Protein TALPID3	KIAA0586			
Q6I9Y2	THO complex subunit 7 homolog	THOC7			
Q9HBL0	Tensin-1	TNS1			
Q96A25	Transmembrane protein 106A	TMEM106A			
Q9NYK1	Toll-like receptor 7	TLR7			
Q9Y6M0	Testisin	PRSS21			
P52655	Transcription initiation factor IIA subunit 1	GTF2A1			
P62072	Mitochondrial import inner membrane translocase subunit Tim10	TIMM10			
A0A1B0GTY4	Testis-expressed protein 50	TEX50			
P10828	Thyroid hormone receptor beta	THRB			
Q13889	General transcription factor IIH subunit 3	GTF2H3			
Q8NDG6	ATP-dependent RNA helicase TDRD9	TDRD9			
O43493	Trans-Golgi network integral membrane protein 2	TGOLN2			
Q9NWD8	Transmembrane protein 248	TMEM248			
Q9HD45	Transmembrane 9 superfamily member 3	TM9SF3			
P21980	Protein-glutamine gamma-glutamyltransferase 2	TGM2			
Q9BTX3	Transmembrane protein 208	TMEM208			
O43715	TP53-regulated inhibitor of apoptosis 1	TRIAP1			
Q9C037	E3 ubiquitin-protein ligase TRIM4	TRIM4			
Q9BVT8	Transmembrane and ubiquitin-like domain-containing protein 1	TMUB1			
Q96BQ3	Tripartite motif-containing protein 43	TRIM43			
Q9BYV2	Tripartite motif-containing protein 54	TRIM54			
Q9P272	Probable tRNA methyltransferase 9B	TRMT9B			
Q9HAV5	Tumor necrosis factor receptor superfamily member 27	EDA2R			
Q9NUQ7	Ufm1-specific protease 2	UFSP2			
P17480	Nucleolar transcription factor 1	UBTF			
Q13432	Protein unc-119 homolog A	UNC119			
Q8IWV7	E3 ubiquitin-protein ligase UBR1	UBR1			
P51668	Ubiquitin-conjugating enzyme E2 D1	UBE2D1			
O43915	Vascular endothelial growth factor D	VEGFD			
Q9H1J7	Protein Wnt-5b	WNT5B			
A1L4Q6	Putative uncharacterized protein FLJ41423				
Q6ZR54	Putative uncharacterized protein FLJ46641				
P07947	Tyrosine-protein kinase Yes	YES1			
Q96MF0	Putative uncharacterized protein LOC100506887				
Q86U90	YrdC domain-containing protein, mitochondrial	YRDC			
Q96PE6	Zinc finger imprinted 3	ZIM3			
Q8N393	Zinc finger protein 786	ZNF786			
A6NM28	Zinc finger protein 92 homolog	ZFP92			
Q8IZ13	Protein ZBED8	ZBED8			
Q96LW1	Zinc finger protein 354B	ZNF354B			

P86452	Zinc finger BED domain-containing protein 6	ZBED6			
P51814	Zinc finger protein 41	ZNF41			
Q96RE9	Zinc finger protein 300	ZNF300			
Q9Y2Y4	Zinc finger and BTB domain-containing protein 32	ZBTB32			
Q8NB15	Zinc finger protein 511	ZNF511			
Q96N58	Zinc finger protein 578	ZNF578			
Q96N20	Zinc finger protein 75A	ZNF75A			
A8MXY4	Zinc finger protein 99	ZNF99			
Q9P2J8	Zinc finger protein 624	ZNF624			
O43264	Centromere/kinetochore protein zw10 homolog	ZW10			
Q8N7M2	Zinc finger protein 283	ZNF283			
Q8WWF5	E3 ubiquitin-protein ligase ZNRF4	ZNRF4			
Q6ZMV8	Putative zinc finger protein 730	ZNF730			
Q99614	Tetratricopeptide repeat protein 1	TTC1			
Q6ZVT0	Inactive polyglycylase TTLL10	TTLL10			
A0A075B6N2	T cell receptor beta variable 20-1	TRBV20-1			
P0CB48	Putative upstream-binding factor 1-like protein 6	UBTFL6			
O95185	Netrin receptor UNC5C	UNC5C			
Q14139	Ubiquitin conjugation factor E4 A	UBE4A			
O14562	Ubiquitin domain-containing protein UBFD1	UBFD1			
P38435	Vitamin K-dependent gamma-carboxylase	GGCX			
Q6UXN9	WD repeat-containing protein 82	WDR82			
Q9UK41	Vacuolar protein sorting-associated protein 28 homolog	VPS28			
Q9ULK5	Vang-like protein 2	VANGL2			
P30291	Wee1-like protein kinase	WEE1			
Q5SQQ9	Ventral anterior homeobox 1	VAX1			
Q9NQH7	Xaa-Pro aminopeptidase 3	XPNPEP3			
Q8NI36	WD repeat-containing protein 36	WDR36			
Q9Y4E1	WASH complex subunit 2C	WASHC2C			
Q6ZS81	WD repeat- and FYVE domain-containing protein 4	WDFY4			
P56705	Protein Wnt-4	WNT4			
Q9H977	WD repeat-containing protein 54	WDR54			
P19544	Wilms tumor protein	WT1			
P09544	Protein Wnt-2	WNT2			
A8MV72	Putative UPF0607 protein ENSP00000382826				
Q96EC8	Protein YIPF6	YIPF6			
Q96TA2	ATP-dependent zinc metalloprotease YME1L1	YME1L1			
Q8NA96	Putative uncharacterized protein FLJ35723				
A8MU10	Putative uncharacterized protein ENSP00000381562				
Q6ZWC4	Putative uncharacterized protein LOC100128429				
Q6ZTC4	Putative uncharacterized protein FLJ44790				
Q6ZVQ6	Putative uncharacterized protein FLJ42213				
Q96NJ6	Zinc finger protein 3 homolog	ZFP3			
Q0VGE8	Zinc finger protein 816	ZNF816			
Q8WU90	Zinc finger CCCH domain-containing protein 15	ZC3H15			
A8MT65	Zinc finger protein 891	ZNF891			
Q9UPT8	Zinc finger CCCH domain-containing protein 4	ZC3H4			
Q9UL40	Zinc finger protein 346	ZNF346			
Q8IWY8	Zinc finger and SCAN domain-containing protein 29	ZSCAN29			

Q15776	Zinc finger protein with KRAB and SCAN domains 8	ZKSCAN8			
Q9BTP6	Zinc finger BED domain-containing protein 2	ZBED2			
Q53GI3	Zinc finger protein 394	ZNF394			
Q6PEW1	Zinc finger CCHC domain-containing protein 12	ZCCHC12			
Q9NW07	Zinc finger protein 358	ZNF358			
Q8IWU4	Zinc transporter 8	SLC30A8			
Q8TAQ5	Zinc finger protein 420	ZNF420			
Q969J2	Zinc finger protein with KRAB and SCAN domains 4	ZKSCAN4			
Q70YC5	Protein ZNF365	ZNF365			
Q99726	Zinc transporter 3	SLC30A3			
Q96GY0	Zinc finger C2HC domain-containing protein 1A	ZC2HC1A			
Q9H4T2	Zinc finger and SCAN domain-containing protein 16	ZSCAN16			
Q92670	Putative zinc finger protein 75C	ZNF75CP			
Q8NAF0	Zinc finger protein 579	ZNF579			
Q03938	Zinc finger protein 90	ZNF90			
Q9BRH9	Zinc finger protein 251	ZNF251			
Q9UK33	Zinc finger protein 580	ZNF580			
Q96IT1	Zinc finger protein 496	ZNF496			
Q4KMZ1	IQ domain-containing protein C	IQCC			
Q6IE81	Protein Jade-1	JADE1			
P05556	Integrin beta-1	ITGB1			
P07476	Involucrin	IVL			
Q9P206	Uncharacterized protein KIAA1522	KIAA1522			
Q14643	Inositol 1,4,5-trisphosphate receptor type 1	ITPR1			
Q92813	Type II iodothyronine deiodinase	DIO2			
Q9NXR8	Inhibitor of growth protein 3	ING3			
O60662	Kelch-like protein 41	KLHL41			
Q9NTI7	PAK4-inhibitor INKA2	INKA2			
A6NCF5	Kelch-like protein 33	KLHL33			
Q9Y2M5	Kelch-like protein 20	KLHL20			
Q2T9L4	Inhibitory synaptic factor 1	INSYN1			
Q8N4N3	Kelch-like protein 36	KLHL36			
Q9P2K6	Kelch-like protein 42	KLHL42			
Q9UIX4	Potassium voltage-gated channel subfamily G member 1	KCNG1			
Q8TAE7	Potassium voltage-gated channel subfamily G member 3	KCNG3			
O95198	Kelch-like protein 2	KLHL2			
Q9C0H6	Kelch-like protein 4	KLHL4			
Q3KP66	Innate immunity activator protein	INAVA			
Q9BW62	Katanin p60 ATPase-containing subunit A-like 1	KATNAL1			
Q96RR4	Calcium/calmodulin-dependent protein kinase kinase 2	CAMKK2			
Q99685	Monoglyceride lipase	MGLL			
A6NNL0	NUT family member 2B	NUTM2B			
Q8NH37	Olfactory receptor 4C3	OR4C3			
Q7RTS5	Proton channel OTOP3	OTOP3			
O95371	Olfactory receptor 2C1	OR2C1			
Q07869	Peroxisome proliferator-activated receptor alpha	PPARA			
P98160	Basement membrane-specific heparan sulfate proteoglycan core protein	HSPG2			
Q8WY54	Protein phosphatase 1E	PPM1E			
Q6S8J7	POTE ankyrin domain family member A	POTEA			
P19174	1-phosphatidylinositol 4,5-bisphosphate phosphodiesterase gamma-1	PLCG1			
Q9UNA4	DNA polymerase iota	POLI			
Q86UA1	Pre-mRNA-processing factor 39	PRPF39			
Q5VWM5	PRAME family member 9/15	PRAMEF9;			

P48634	Protein PRRC2A	PRRC2A			
P08575	Receptor-type tyrosine-protein phosphatase C	PTPRC			
Q8NFP0	Peroxisomal testis-specific protein 1	PXT1			
O14949	Cytochrome b-c1 complex subunit 8	UQCRQ			
O43374	Ras GTPase-activating protein 4	RASA4			
Q9BYZ8	Regenerating islet-derived protein 4	REG4			
Q7Z5J4	Retinoic acid-induced protein 1	RAI1			
P48382	DNA-binding protein RFX5	RFX5			
P63173	60S ribosomal protein L38	RPL38			
Q969X0	RILP-like protein 2	RILPL2			
P09001	39S ribosomal protein L3, mitochondrial	MRPL3			
Q9BYD2	39S ribosomal protein L9, mitochondrial	MRPL9			
Q8N983	39S ribosomal protein L43, mitochondrial	MRPL43			
Q8WVZ7	E3 ubiquitin-protein ligase	RNF133			
P05387	60S acidic ribosomal protein P2	RPLP2			
P51991	Heterogeneous nuclear ribonucleoprotein A3	HNRNPA3			
Q9BZX4	Ropporin-1B	ROPN1B			
P34096	Ribonuclease 4	RNASE4			

Supplemental table 2 the docking setting

gene	number	molecule	center_x	center_y	center_z	size_x	size_y	size_z
AKT1	A1	kaempferol	-26.573	2.825	16.243	67.55	67.55	67.55
AKT1	HQ17	quercetin	-26.573	2.825	16.243	65.45	65.45	65.45
ICAM1	A1	kaempferol	-5.202	-12.754	36.188	112.35	112.35	112.35
ICAM1	HQ17	quercetin	-5.202	-12.754	36.188	126	126	126
JUN	A1	kaempferol	-16.523	23.084	26.257	84.35	84.35	84.35
JUN	HQ11	formononetin	-16.523	23.084	26.257	75.95	75.95	75.95
JUN	HQ17	quercetin	-16.523	23.084	26.257	78.75	78.75	78.75
PPARG	A1	kaempferol	46.402	9.632	18.521	61.25	61.25	61.25
PPARG	HQ11	formononetin	46.402	9.632	18.521	64.75	64.75	64.75
PPARG	HQ17	quercetin	46.402	9.632	18.521	71.05	71.05	71.05
PPARG	RS10	Frutinone A	46.402	9.632	18.521	66.15	66.15	66.15
PTGS2	A1	kaempferol	35.631	25.018	217.817	126	126	126
PTGS2	HQ11	formononetin	35.631	25.018	217.817	99.75	99.75	99.75
PTGS2	HQ17	quercetin	35.631	25.018	217.817	126	126	126
PTGS2	RS10	Frutinone A	35.631	25.018	217.817	126	126	126
PPTGS2	RS11	ginsenoside rh2	35.631	25.018	217.817	126	126	126
TNF	A1	kaempferol	45.304	52.906	13.785	59.85	59.85	59.85
TNF	HQ17	quercetin	45.304	52.906	13.785	75.25	75.25	75.25
TNF	RS11	ginsenoside rh2	45.304	52.906	13.785	68.95	68.95	68.95
